# Dual Catalytic Enantioconvergent Carbamoylation of Aziridines

**DOI:** 10.1021/jacs.5c15873

**Published:** 2025-11-20

**Authors:** Liangliang Zhang, Huilin Liu, Tomás G. Santiago, Ruben Martin

**Affiliations:** † 202569 Institute of Chemical Research of Catalonia (ICIQ), The Barcelona Institute of Science and Technology, Av. Països Catalans 16, 43007 Tarragona, Spain; ‡ Universitat Rovira i Virgili, Departament de Química Orgànica, 43007 Tarragona, Spain; § ICREA, Passeig Lluís Companys, 23, 08010 Barcelona, Spain

## Abstract

Herein, we disclose an enantioconvergent carbamoylation of racemic aziridines enabled by a dual catalytic strategy. The method offers a new entry point to highly enantioenriched β-amino amides from simple precursors and is characterized by its broad applicability, even with challenging combinations. Preliminary studies with well-defined nickel complexes support a scenario via carbamoyl organometallic intermediates.

## Graphical abstract

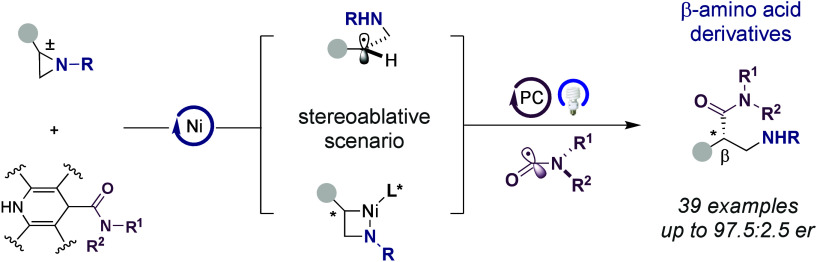

Over the past years, Ni-catalyzed enantioconvergent reactions of racemic alkyl electrophiles have gained momentum in synthetic organic chemistry (Scheme 1, *path a*).^1^ Such interest arises not only from the inherent flexibility of Ni catalysts in one- or two-electron manifolds^2^ but also from the ability to convert two enantiomers into prochiral species via stereoablative processes.^3^ Among other scenarios, enantioconvergent reactions of racemic aziridines have attracted significant attention given that these techniques offer a direct, yet valuable, entry point to enantioenriched β-functionalized aliphatic amine architectures (*path b*),^4^ prevalent motifs in a myriad of molecules that display important biological activities.^5^ While elegant advances have been realized, Ni-catalyzed enantioconvergent couplings of racemic aziridines beyond alkenylation,^6^ arylation,^7^ or alkylation^8^ events still remain an elusive endeavor, yet have potential to open up a broad range of transformations while streamlining the access to valuable enantioenriched β-functionalized amine-containing building blocks.^9,10^

**Scheme 1:**
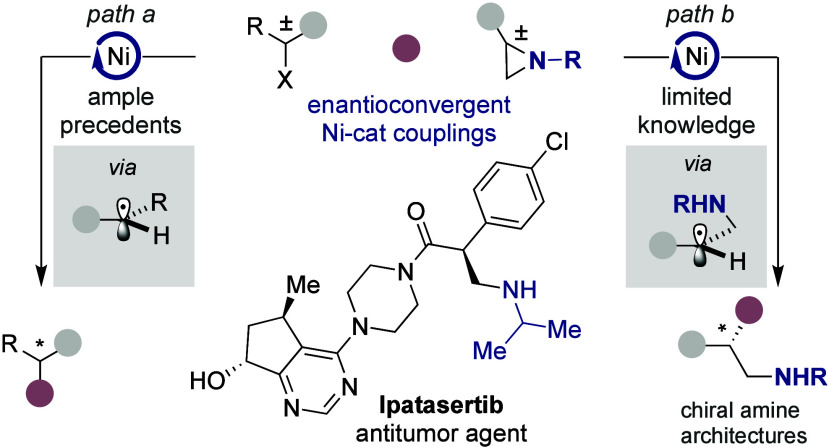
Ni-Catalyzed Enantioconvergent Reactions

Driven by the emerging demand for β-amino acid derivatives in medicinal chemistry settings^11^—molecules that offer greater chemical diversity and higher resistance to hydrolysis by enzymes than peptides containing α-amino acids^12^—a particularly rewarding scenario would consist of designing a Ni-catalyzed enantioconvergent carbamoylation of readily available racemic aziridines (Scheme 2). As part of our ongoing interest in Ni-catalyzed enantioselective reactions,^8c,13^ we wondered whether we could design a blueprint that merges the modularity of nickel catalysts for bond formation^2^ with the flexibility of photoinduced processes for generating open-shell species under mild conditions.^14^ At present, however, enantioconvergent carbamoylations of racemic alkyl electrophiles constitute an uncharted cartography in the cross-coupling arena.^15^ If successful, the design of such a technique would not only offer an unrecognized opportunity to expand our ever-growing arsenal in enantioconvergent cross-coupling reactions^1^ but also complement existing synthetic routes for preparing valuable β-amino acid architectures.^11,12^ Specifically, we anticipated that β-amino amides could be easily within reach by intercepting carbamoyl radical species **I** generated in situ from 1,4-dihydropyridines (DHP) via photoinduced single-electron transfer (SET) processes^16^ with Ni­(II) azanickelacycles **III** ^17^ arising from a stereoablative event via β-amino alkyl radical intermediates **II** (Scheme 2). Herein, we report the successful realization of this goal. The protocol is distinguished by its broad applicability, excellent chemoselectivity pattern, and exquisite enantioinduction, even with challenging substrate combinations.

**Scheme 2:**
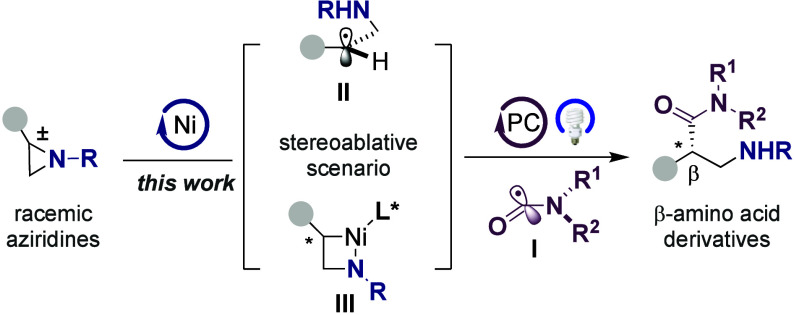
Enantioconvergent Aziridine Carbamoylation

Our study began by evaluating the Ni-catalyzed enantioconvergent reaction of racemic **1a** with **2a**, readily available in bulk from 2-oxoacetic acid (Table 1).^18^ After considerable optimization,^18^ a combination of **PC1** (1.5 mol %), NiBr_2_·diglyme (10 mol %), **L1** (15 mol %), BnBu_3_NI (10 mol %), KClO_4_ (50 mol %), and 4-CF_3_C_6_H_4_CO_2_Na (50 mol %) in MeCN/PhCF_3_ (1:4, 0.028 M) under blue-LED (451 nm) irradiation at 30 °C for 16 h delivered the best results, leading to **3a** in 75% yield and 96:4 enantiomeric ratio (er) (entry 1). As anticipated, the nature of the ligand is critical for success. As shown, the tether at the *C*_2_-symmetric bis-oxazoline backbone had a deleterious effect on both the enantioselectivity and reactivity (entries 2–4). While bis­(imidazoline) ligands of type **L5** have been employed with success in other aziridine enantioconvergent events,^7b,7c,8c^ their utilization resulted in a negligible yield of **3a** and a markedly lower enantioinduction (entry 5). Intriguingly, the utilization of otherwise related Ni­(cod)_2_, NiCl_2_·glyme, or **PC2** resulted in marginal yields and lower er values (entries 6, 8, and 9). In contrast, traces of **3a**, if any, were found by utilizing Ir­[(dtbpy)­(ppy)_2_]­PF_6_ instead of **PC1** (entry 7). Control experiments confirmed that the absence of KClO_4_ (entry 10) and the presence of TBAB or PhCO_2_Na instead of 4-CF_3_C_6_H_4_CO_2_Na and BnBu_3_NI (entries 11 and 12) led to a decrease in yield and enantioinduction. Equally striking was the observation that low yields were accomplished in MeCN or PhCF_3_ as the solvent, thus confirming that the combination of both was optimal for the reaction to occur (entries 13 and 14). As anticipated, the absence of either light or photocatalyst resulted in no conversion to **3a**.^18^

**Table 1:**
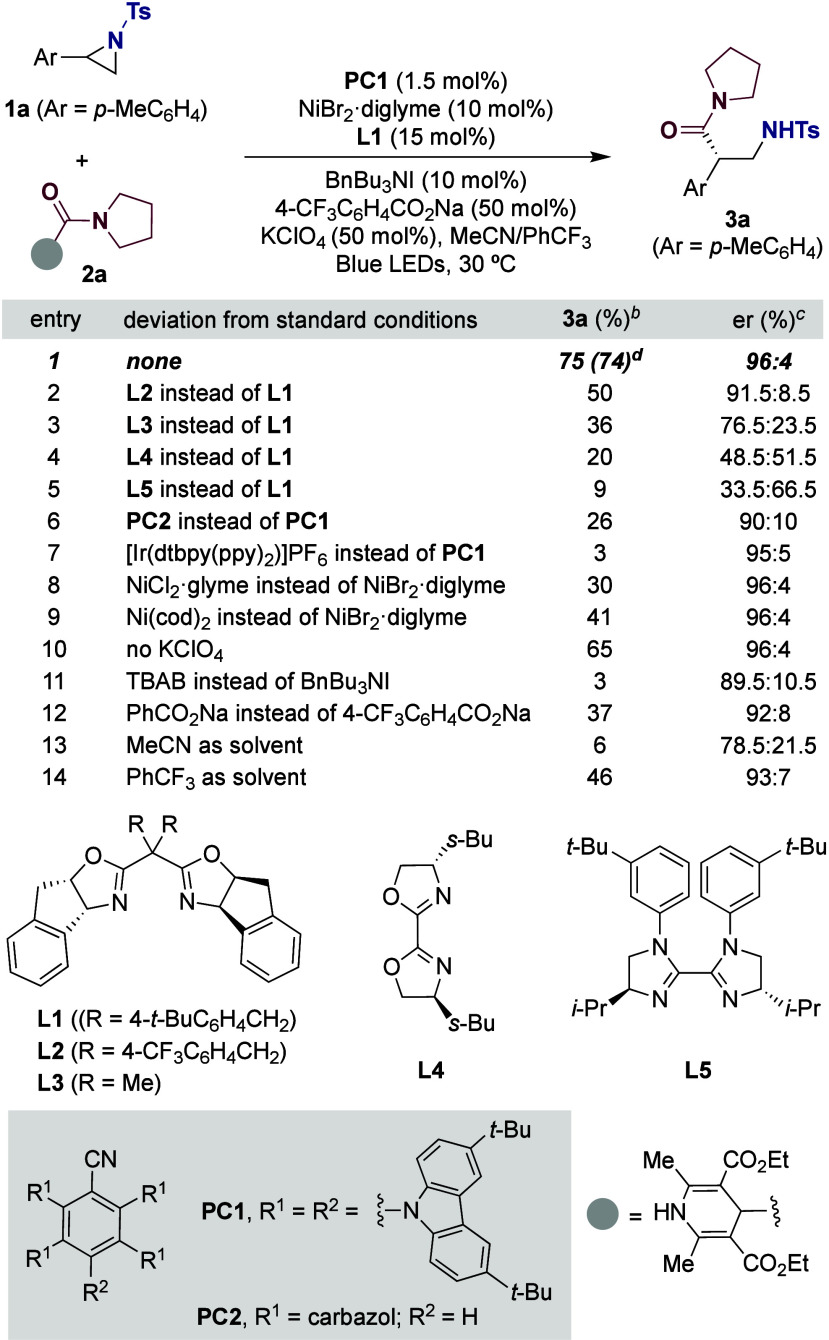
Optimization of Reaction Conditions^b^

a Conditions: **1a** (0.10 mmol), **2a** (0.20 mmol), **PC1** (1.5 mol %), NiBr_2_·diglyme (10 mol %), **L1** (15 mol %), BnBu_3_NI (10 mol %), KClO_4_ (0.050 mmol), 4-CF_3_C_6_H_4_CO_2_Na (0.050 mmol), CH_3_CN:PhCF_3_ (1:4, 0.028 M), blue LED (451 nm), 30 °C, 16 h.

b GC yield using dodecane as internal standard.

c er values were determined by SFC analysis.

d Isolated yield, average of two independent runs.

With optimized conditions in hand, we next turned our attention to studying the generality of our Ni-catalyzed enantioconvergent carbamoylation of aziridines. As shown in Table 2, the protocol was found to be equally applicable to a variety of aziridines regardless of whether electron-rich or electron-deficient substituents were incorporated at the arene motif.^19^ The chemoselectivity of the reaction was illustrated by the observation that amides (**3i**), nitriles (**3j**), or esters (**3m**) were well accommodated, obtaining the targeted compounds in good yields and high stereochemical fidelity. The absolute stereochemistry of the reaction was univocally confirmed by X-ray diffraction of **3g**.^18^ It is worth noting that aziridines bearing indolyl and benzofuran groups could also be equally compatible, delivering targeted compounds **3k** and **3o** with high efficiency and stereocontrol. More importantly, the method could be applied to advanced synthetic intermediates derived from estrone (**3s**), indomethacin (**3t**), naproxen (**3u**), or (*S*)- ibuprofen (**3v**) in good yields and high stereoselectivity, thus holding promise for the implementation of this protocol in medicinal chemistry programs. In addition, the utilization of *ent*-**L1** as ligand resulted in the formation of *epi-* **3s**, *epi-* **3u**, and *epi*-**3v**, tacitly indicating that the Ni-catalyzed enantioconvergent carbamoylation of aziridines is ligand- rather than substrate-controlled. Moreover, this strategy could be applied across a number of different 1,4-dihydropyridine building blocks. Thus, the method could incorporate acyclic **4a** or cyclic amide derivatives with similar ease, including compounds bearing four- (**4b**–**4e**), five- (**4f**, **4g**), and six-membered rings (**4h**, **4i**). Even spirocyclic motifs could be within reach under otherwise identical reaction conditions (**4j**–**4m**). While the inclusion of heteroaryl moieties posed no problems (**4i**), it is worth mentioning that the presence of benzyl substituents at the carbamoyl fragment resulted in lower enantioselectivities (**4n**). Furthermore, the presence of esters (**4e**) or amides (**4g**, **4l**) did not interfere with our Ni-catalyzed enantioconvergent carbamoylation, thus giving access to the targeted compounds in good yields and excellent levels of enantioselectivity.

**Table 2:**
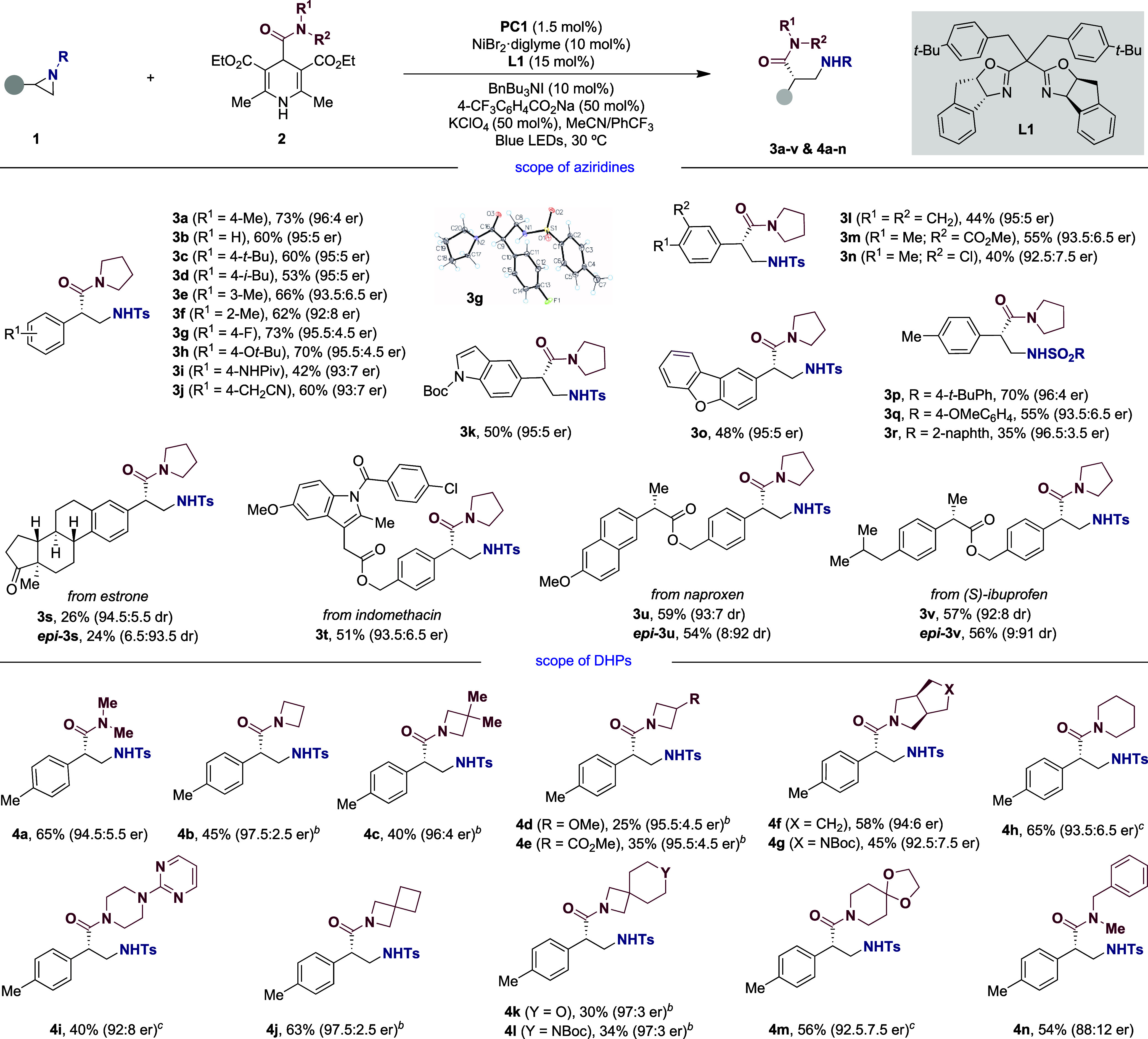
Scope of the Ni-Catalyzed Enantioselective Carbamoylation of Aziridines^a^

a Conditions: as for Table 1 (entry 1). Isolated yields, average of two independent runs.

b Utilizing carbamoyl DHP (1.50 equiv).

c MeCN:PhCF_3_ (0.05 M).

To further illustrate the versatility of our protocol, we decided to assess the synthetic applicability of the carbamoyl products arising from our Ni-catalyzed enantioconvergent coupling event. As shown in Scheme 3, simple exposure of **3a** to formaldehyde under acidic conditions cleanly delivered the tetrahydroisoquinoline core **5** in good yields.^18^ On the other hand, a tandem Pd-catalyzed C–N bond-formation/intramolecular C–H arylation en route to thiazepine **6** could be easily within reach from **3a**, whereas a simple reduction with LiAlH_4_ resulted in the formation of the 1,3-diamine motif **7**.^18^ It is worth noting that in all cases analyzed, **5**–**7** were obtained with complete retention of the stereochemical integrity at the benzylic site.

**Scheme 3:**
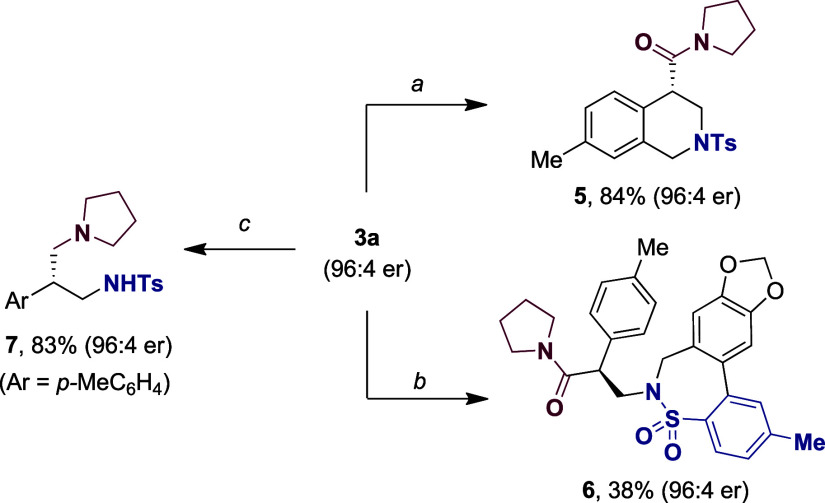
Synthetic Applicability

a (HCHO)*_n_* (3.0 equiv), H_3_[PW_12_O_40_] (10 mol %), CH_3_CN, 100 °C.
b 5-Bromo-6-(bromomethyl)­benzo­[*d*]­[1,3]­dioxole (1.2 equiv), Pd­(OAc)_2_ (10 mol %), PPh_3_ (20 mol %), Cs_2_CO_3_ (2.5 equiv), 1,4-dioxane, 110 °C, 16 h.
c LiAlH_4_ (2.0 equiv), THF, 100 °C, 16 h.

Encouraged by the results shown in Table 2, we conducted a series of control experiments to understand the enantioconvergent nature of our Ni-catalyzed carbamoylation of aziridines. As shown in Scheme 4 (*top*), enantioconvergency could be substantiated by obtaining **3b** in otherwise identical yields and enantioselectivities regardless of whether *R-* or *S-* **1b** was utilized as substrate, thus showing that the stereochemical fidelity exclusively arises from **L1**. On the other hand, not even traces of **3b** were observed with β-halo sulfonamides **8a** and **8b** as substrates (Scheme 4, *bottom*). It is worth noting that substantial amounts of homocoupling products were observed in the crude mixtures, thus confirming that an appropriate concentration of radical species is critical for success.^20^

**Scheme 4:**
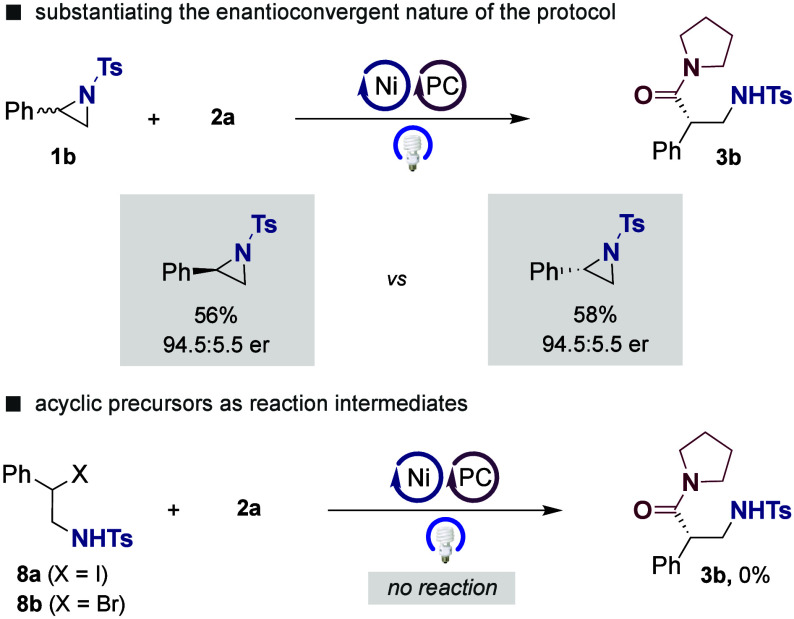
Control Experiments

Aiming at understanding the mechanistic intricacies of the reaction, we prepared **L1**NiBr_2_ (**Ni-1**) by simple exposure of NiBr_2_ to **L1** in THF at 40 °C, the structure of which was univocally characterized by X-ray diffraction (Scheme 5, *right*).^18^ As judged by our cyclic voltammetry (CV) data (Scheme 5, *bottom*), the reduced form of **PC1** (*E*_1/2_ PC1/PC1^·–^ = −1.99 V vs Fc^+^/Fc) might easily promote two consecutive single-electron transfer (SET) reductions of **Ni-1** to Ni(0) (**Ni-1**; *E*_1/2_ Ni­(II)/Ni­(I) = −0.94 V and *E*_1/2_ Ni­(I)/Ni(0) = −1.89 V vs Fc^+^/Fc).^18^ Although other mechanistic pathways might a priori be conceivable,^18^ we tentatively favor a pathway consisting of oxidative addition of **1** to Ni(0)­L_*n*_ en route to **III** (Scheme 5, *left*). Subsequently, reaction with a carbamoyl radical **I** obtained by SET oxidation^18^ from **2** generates **IV**, which upon reductive elimination gives rise to **3** or **4** and Ni­(I)­L_*n*_ **V**.^21^ The latter could a priori be in equilibrium with **VI** by interception with **I—** formally making **VI** a reservoir of **I** via light-induced C–Ni bond homolysis—or evolve via SET to regenerate the propagating Ni(0)­L_*n*_. While the synthesis of **III** and **VI** bearing **L1** proved particularly problematic, we turned our attention to the synthesis of Ni/**L6** (**L6** = 4,4′-di-*tert*-butyl-2,2′-bipyridine) analogues given that the reaction of **1a** with **2a** under a Ni/**L6** regime results in 45% yield of *rac-* **3a**. Gratifyingly, oxidative addition reaction of **1g** or pyrrolidine-1-carbonyl chloride to Ni­(cod)_2_/**L6** cleanly delivered **Ni-2** ^22^ and **Ni-3** ^23^ (Scheme 5, *right*), whose structures were characterized by X-ray diffraction.

**Scheme 5:**
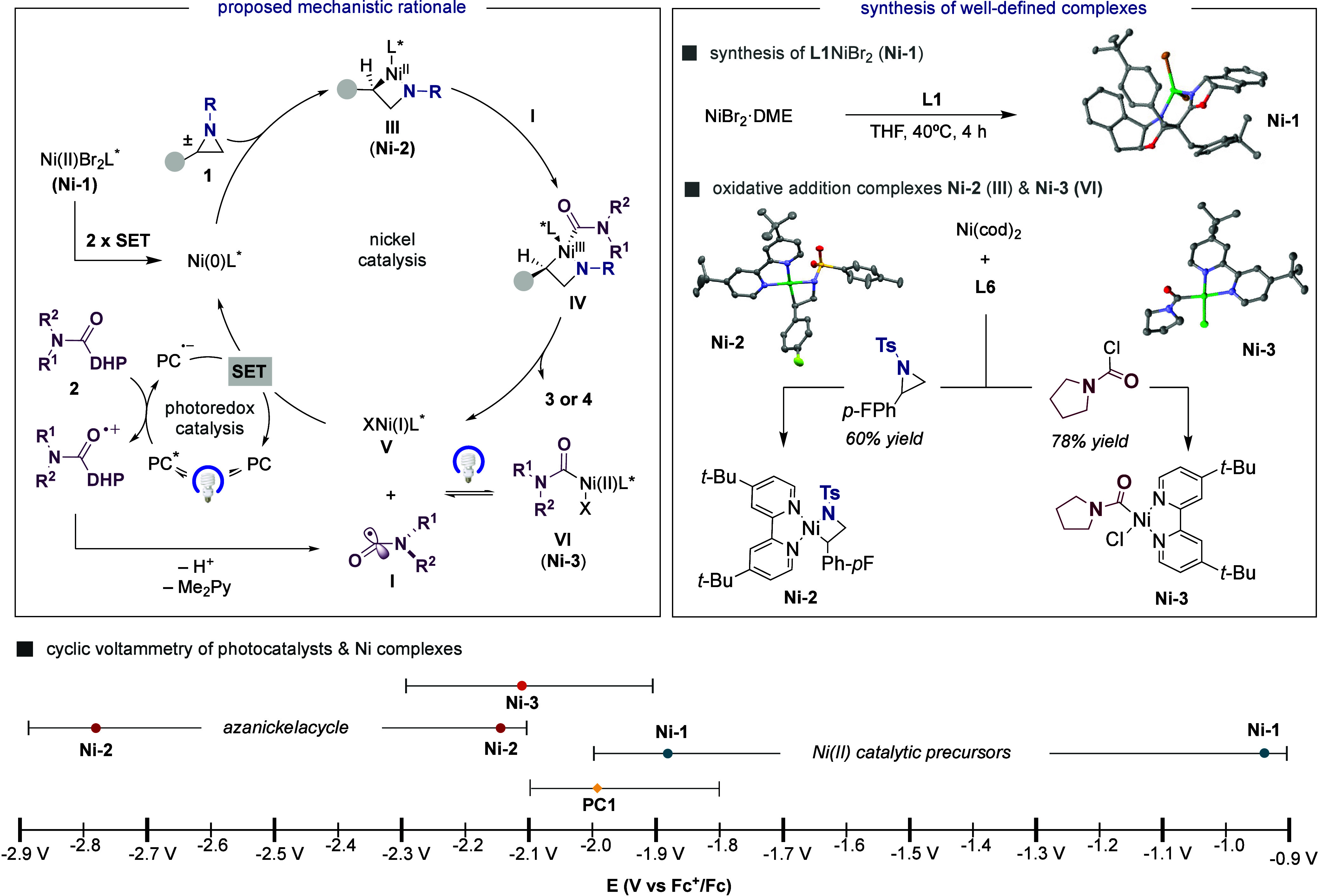
Mechanistic Interpretation and Synthesis of Well-Defined Ni Complexes

As expected, **Ni-1** was found to be catalytically competent en route to **3a**, obtaining the targeted compound with 93.5:6.5 er (Scheme 6, *top*).^24^ Notably, a 38% yield of **3g** was obtained when exposing **Ni-2** to **2a** under light irradiation with stoichiometric **PC1** (Scheme 6, *middle*), with non-negligible amounts of carbamoyl homocoupling byproducts being observed in the crude reaction mixture.^18^ Given that SET reduction of **Ni-2** by **PC1** is highly unlikely according to our CV data (Scheme 5, *bottom*), all these observations (a) raise a reasonable doubt about a pathway consisting of single-electron reduction of **Ni-2** prior to reaction with a carbamoyl radical **I** ^18^ and (b) strongly suggest that an appropriate concentration of **I** is critical for the reaction to occur while avoiding formation of parasitic homocoupling events. On the other hand, **3a** and **9** were obtained in moderate yields by exposure of **Ni-3** to either 2-phenyl acrylate or **1a** (Scheme 6, *bottom*).^25^ Notably, no reaction occurred in the dark, thus confirming that photolysis of the C–Ni bond precedes C–C bond-formation.^26^ These results reinforce the perception that **Ni-3** might act as a reservoir of carbamoyl radical species **I**, keeping an appropriate concentration of the latter to be intercepted by **Ni-2**.^27^ This hypothesis gained credence by observing a 40% yield of **3g** upon reaction of **Ni-3** and **Ni-2** (1:1 ratio) under light irradiation. Taken together, our data suggests the intervention of organometallic carbamoyl complexes as reservoirs of open-shell species upon photolysis, thus offering new opportunities to design enantioconvergent coupling scenarios.

**Scheme 6:**
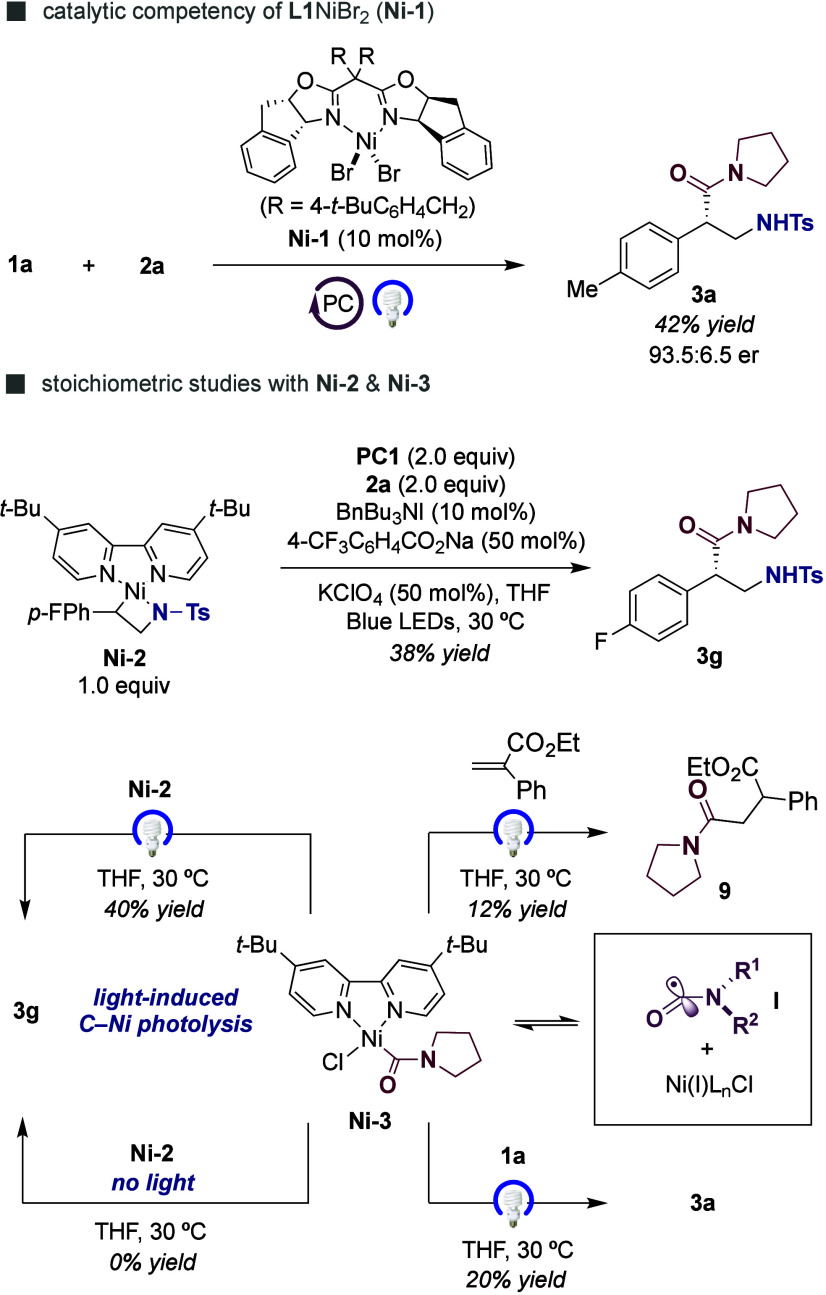
Studies with Well-Defined Ni Complexes

In summary, we have developed a dual catalytic enantioconvergent carbamoylation of aziridines that streamlines access to enantioenriched β-amino amides. This strategy is characterized by its mild conditions, broad applicability, including late-stage diversification of advanced intermediates, and high enantioinduction. Mechanistic studies revealed the competency of well-defined carbamoyl nickel complexes as reservoirs of carbamoyl radicals upon photolysis. We expect that the broader consequences of our protocol might foster the discovery of new enantioconvergent scenarios.

## Supplementary Material



## Notes

Experimental details, compound characterization data, SFC chromatographs, NMR spectra, and crystallographic data (PDF)

## References

[1] Yus M., Nájera C., Foubelo F., Sansano J. M. (2023). Metal-Catalyzed Enantioconvergent
Transformations. Chem. Rev..

[2] a Nickel Catalysis in Organic Synthesis: Methods and Reactions, 1st ed.; Ogoshi, S. , Ed.; Wiley, 2020.

[3] Mohr J. T., Ebner D. C., Stoltz B. M. (2007). Catalytic
Enantioselective
Stereoablative Reactions: An Unexploited Approach to Enantioselective
Catalysis. Org. Biomol. Chem..

[4] a Aziridines and Epoxides in Organic Synthesis, 1st ed.; Yudin, A. K. , Ed.; Wiley, 2006.

[5] a Lawrence, S. A. Amines: Synthesis, Properties, and Applications; Cambridge University Press: Cambridge, 2004.

[6] Hu X., Cheng-Sánchez I., Cuesta-Galisteo S., Nevado C. (2023). Nickel-Catalyzed Enantioselective
Electrochemical Reductive Cross-Coupling of Aryl Aziridines with Alkenyl
Bromides. J. Am. Chem. Soc..

[7] Woods B. P., Orlandi M., Huang C.-Y., Sigman M. S., Doyle A. G. (2017). Nickel-Catalyzed Enantioselective
Reductive Cross-Coupling
of Styrenyl Aziridines. J. Am. Chem. Soc..

[8] Huang C.-Y., Doyle A. G. (2015). Electron-Deficient
Olefin Ligands
Enable Generation of Quaternary Carbons by Ni-Catalyzed Cross-Coupling. J. Am. Chem. Soc..

[9] Trost B. M., Osipov M., Dong G. (2010). Palladium-Catalyzed Dynamic Kinetic
Asymmetric Transformations of Vinyl Aziridines with Nitrogen Heterocycles:
Rapid Access to Biologically Active Pyrroles and Indoles. J. Am. Chem. Soc..

[10] Wang R., Fang Y., Wang C. (2024). Regioselective
and Enantioselective
Acylative Ring Opening of Aziridines via Cooperative Nickel Photocatalysis. Cell Rep. Phys. Sci..

[11] a Amino Acids, Peptides and Proteins in Organic Chemistry: Origins and Synthesis of Amino Acids, 1st ed.; Hughes, A. B. , Ed.; Wiley, 2009.

[12] Cabrele C., Martinek T. A., Reiser O., Berlicki L. (2014). Peptides containing
β-amino acid patterns: challenges
and successes in medicinal chemistry. J. Med.
Chem..

[13] Sun S.-Z., Cai Y.-M., Zhang D.-L., Wang J.-B., Yao H.-Q., Rui X.-Y., Martin R., Shang M. (2022). Enantioselective
Deaminative Alkylation of Amino Acid Derivatives with Unactivated
Olefins. J. Am. Chem. Soc..

[14] Prier C. K., Rankic D. A., MacMillan D. W. C. (2013). Visible
Light Photoredox Catalysis with Transition Metal Complexes: Applications
in Organic Synthesis. Chem. Rev..

[15] Li L., Hu Z., Ren S., Arndtsen B. A., Chu L. (2025). Enantioselective
Carbonylative Coupling Reactions: Merging Nickel-Based Selectivity
and Photoredox Reactivity. J. Am. Chem. Soc..

[16] Alandini N., Buzzetti L., Favi G., Schulte T., Candish L., Collins K. D., Melchiorre P. (2020). Amide Synthesis
by Nickel/Photoredox-Catalyzed Direct Carbamoylation of (Hetero)­Aryl
Bromides. Angew. Chem., Int. Ed..

[17] Lin B. L., Clough C. R., Hillhouse G. L. (2002). Interactions
of Aziridines with Nickel
Complexes: Oxidative-Addition and Reductive-Elimination Reactions
that Break and Make C–N Bonds. J. Am.
Chem. Soc..

[18] See Supporting Information for details.

[19] Mass balance predominantly accounts for the formation of homocoupling products arising from either **I** or the intermediacy of benzyl radicals generated in situ.

[20] No reaction was observed when utilizing BnBu_3_NI (1.0 equiv), suggesting that the concentration of iodide species and radical intermediates is critical for success.

[21] Cyclic voltammetry of **Ni-2** reveals that its reduction potential is significantly more negative than that of the photocatalyst, making a SET reduction en route to Ni(I) rather unlikely. Thus, direct radical capture is the most plausible avenue for this intermediate.

[22] Dongbang S., Doyle A. G. (2022). Ni/Photoredox-Catalyzed
C­(sp^3^)–C­(sp^3^) Coupling between Aziridines
and Acetals as Alcohol-Derived
Alkyl Radical Precursors. J. Am. Chem. Soc..

[23] See Supporting Information for details of the photophysical characterization of **Ni-3**, including UV–vis spectroscopy and quantum yield analysis.

[24] This observation suggests the intervention of monomeric nickel species bearing **L1**, an assumption that was corroborated by nonlinear effects. See Supporting Information for details.

[25] 40% of **3a** was observed when exposing **8a** to **Ni-3**. This result is in sharp contrast to that shown in Scheme 4 (*bottom*), thus advocating the notion that **Ni-3** might serve as a reservoir of carbamoyl radicals, keeping an appropriate concentration of **I** in solution for the reaction to occur.

[26] Cagan D. A., Bím D., Silva B., Kazmierczak N. P., McNicholas B. J., Hadt R. G. (2022). Elucidating the Mechanism of Excited-State Bond Homolysis
in Nickel–Bipyridine Photoredox Catalysts. J. Am. Chem. Soc..

[27] In line with this notion, we found a metal-to-ligand charge transfer (MLCT) band centered in **Ni-3** at 485 nm. This is in excellent alignment with the wavelength of the applied blue LEDs, thus reinforcing the assumption for a C–Ni homolysis upon irradiation. See Supporting Information for details.

