# Supplementary material for: Dual Catalytic Enantioconvergent Carbamoylation of Aziridines
Source: J Am Chem Soc. 2025 Nov 20;147(48):44048–54. doi: 10.1021/jacs.5c15873 (PMC12679628; doi:10.1021/jacs.5c15873)
Supplement: Supplementary file 1 [file ja5c15873_si_001.pdf]

## Supporting Information

# Dual Catalytic Enantioconvergent Carbamoylation of Aziridines

Liangliang Zhang,†§ Huilin Liu,†§¶ Tomás G. Santiago,† and Ruben Martin*†£

† Institute of Chemical Research of Catalonia (ICIQ), The Barcelona Institute of Science and Technology, Av. Països Catalans 16, 43007 Tarragona, Spain

¶ Departament de Química Analítica i Química Orgànica, Universitat Rovira i Virgili, c/Marcel·lí Domingo, 1, 43007 Tarragona, Spain

£ ICREA, Passeig Lluís Companys, 23, 08010 Barcelona, Spain

§ These authors contributed equally to this work

E-mail: rmartinromo@iciq.es

## Table of Contents

***1. General Considerations*** ........ ***3***

***2. Optimization of the Reaction Conditions*** ........ ***5***

***3. Synthesis of Starting Materials*** ........ ***7***

3.1 Synthesis of aziridines ........ 7

3.2 Synthesis of 1,4-Dihydropyridines (DHP) ........ **13**

***4. Ni-Catalyzed Enantioselective Carbamoylation of Aziridines*** ........ ***19***

***5. Scale up Reactions and Synthetic Applications*** ........ ***67***

5.1 Scale up Reaction of **1a** and **2a** ........ **67**

5.2 Cyclization of **3a** with $(HCHO)_n$ ........ **67**

5.3 Cyclization of **3a** with 5-bromo-6-(bromomethyl)benzo[*d*][1,3]dioxole (**S2**) ........ **69**

5.4 Hydrogenation of **3a** with $LiAlH_4$ ........ **70**

***6. Mechanistic Experiments*** ........ ***72***

6.1 Evidence for stereoconvergent scenarios ........ **72**

6.2 Intermediacy of open-shell species ........ **74**

6.3 Utilization of β-halo sulfonamides as substrates ........ **76**

6.4 Non-linear effect studies ........ **77**

6.5 Reactivity of **Ni-1** (**L1**$NiBr_2$) complex ........ **77**

6.6 Cyclic voltammetry (CV) analysis ....................79
6.7 Fluorescence Quenching Studies ....................81
6.8 Synthesis and reactivity studies of **Ni-2** and **Ni-3** complexes ....................82
6.9 Stability tests of **Ni-2** complex ....................86
6.10 Discussion on the reactivity of **Ni-2** under both stoichiometric and catalytic conditions ....................87
***7. Photophysical Characterization of Ni-3: UV–Vis Spectroscopy and Quantum Yield Analysis 88***
7.1 UV–Vis Spectroscopic Analysis ....................88
7.2 Photostability and Kinetic Analysis of **Ni-3** under 451 nm Irradiation ....................89
7.3 Quantum yield ....................90
7.4 EPR characterization of photogenerated Ni(I) species from **Ni-3** ....................90
***8. Alternative Mechanistic Scenario: Proposed Ni(I)/Ni(III)/Ni(II)/Ni(III) Manifold (path B) 92***
***9. X-Ray Crystallographic Data ....................93***
***10. References ....................97***
***11. NMR Spectra ....................98***

## 1. General Considerations

**Reagents.** Commercially available materials were used as received without further purification. $NiBr_2$·diglyme, benzyltributylammonium ($Bn(nBu)_3NI$), anhydrous *α*,*α*,*α*-Trifluorotoluene (≥99% purity) were purchased from *Aldrich*. Sodium 4-(trifluoromethyl)benzoate (4-$CF_3$PhCOONa) was purchased from *Fluorochem*. Potassium perchlorate ($KClO_4$) was purchased from *Merck*. Anhydrous acetoneitrile ($CH_3CN$) was purchased from *Thermo Fisher Scientific*.

**Analytical methods.** $^1H$ and $^{13}C$ NMR spectra were recorded on Bruker 400 MHz and 500 MHz at 20 °C, unless otherwise indicated. All $^1H$ NMR spectra are reported in parts per million (ppm) downfield of TMS and were calibrated using the residual solvent peak of $CHCl_3$ (7.26 ppm), unless otherwise indicated. All $^{13}C$ NMR spectra are reported in ppm relative to TMS, were calibrated using the signal of residual $CHCl_3$ (77.16 ppm), $^{19}F$ NMR was obtained with $^1H$ decoupling unless otherwise indicated. Coupling constants, *J* are reported in Hertz. Melting points were measured using open glass capillaries in a Büchi B540 apparatus. Infrared spectra (FT-IR) measurements were carried out on a Bruker Optics FT-IR Alpha spectrometer equipped with a DTGS detector, KBr beamsplitter at 4 $cm^{-1}$ resolution using a one bounce ATR accessory with diamond windows. Mass spectra were recorded on a Waters LCT Premier spectrometer or in a MicroTOF Focus, Bruker Daltonics spectrometer. Gas chromatographic analyses were performed on Hewlett-Packard 6890 gas chromatography instrument with FID detector. Flash chromatography was performed with EM Science silica gel 60 (230-400 mesh) using potassium permanganate as the TLC stain. Enantiomeric excesses were determined by supercritical fluid chromatography (SFC) analysis on an Agilent Technologies 1260 Infinity II instrument with a chiral stationary phase using a Chiralpak IC-3, ID-3, IE, or IH-3 column. The yields reported refer to isolated yields and represent an average of at least two independent runs. The procedures described in this section are representative. Thus, the yields may differ slightly from those given in the tables of the manuscript.

**Light Source**. All reactions were performed with 451 nm LEDs (OSRAM Oslon® SSL 80 royal- blue LEDs), which were installed at the bottom of a custom-made 8 flat-bottom Schlenk tubes holder (the distance between the flat-bottom Schlenk tube and the light source was measured to be ~7 mm), equipped with chiller cooling system (the thermostat was set at 30 °C) and a magnetic stirrer (850 rpm). The reactions were run in LLG-headspace-vials (ND20, 7 ml, 22 x 38.20 mm) capped with aluminium crimp seals (ND20, septa butyl grey / PTFE grey, hardness 50° shore A, thickness 3 mm), both purchased from Carlo Erba.

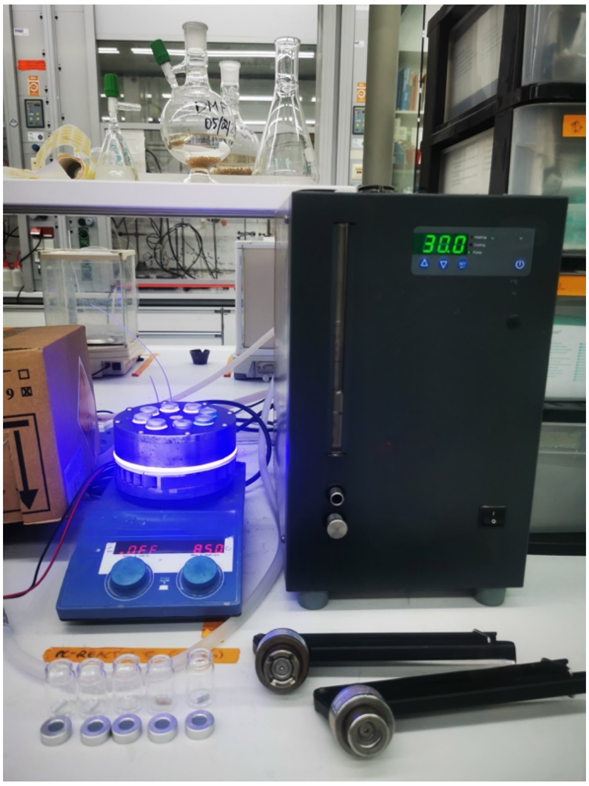

## 2. Optimization of the Reaction Conditions

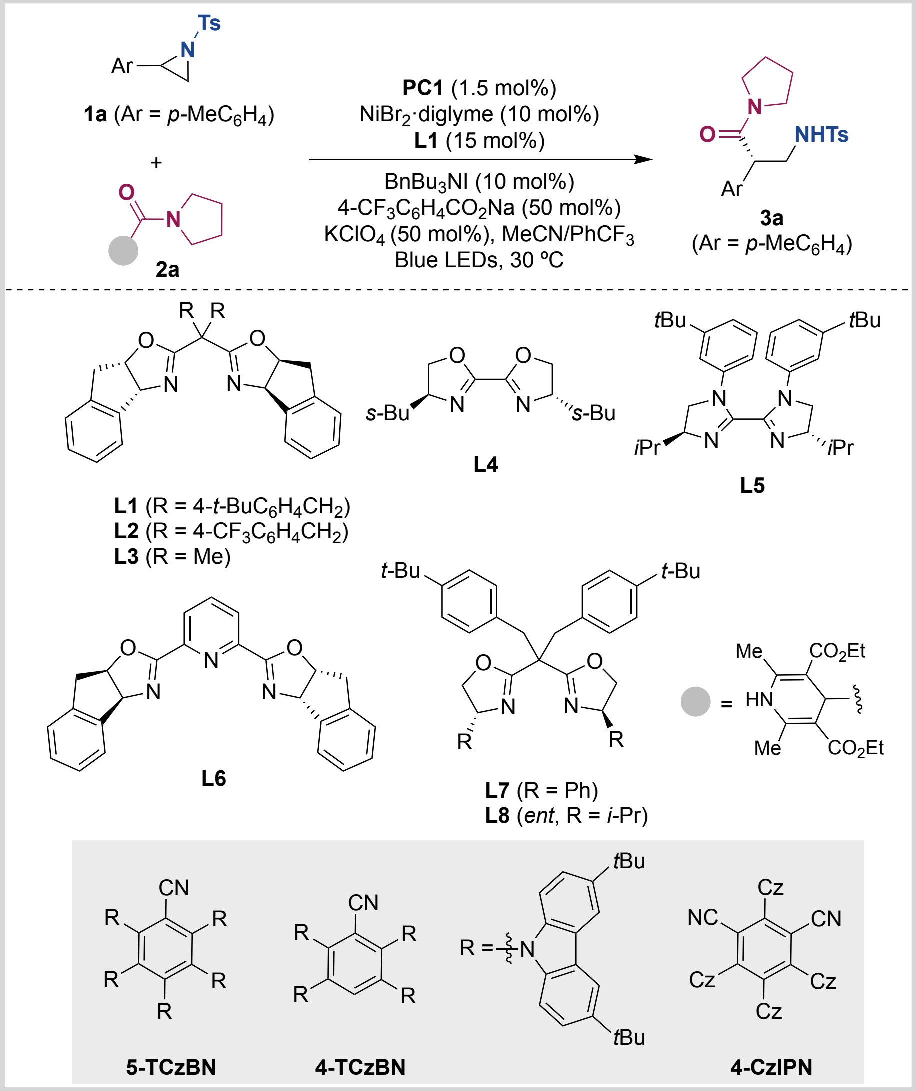


**General procedure:** To a 7 mL vial equipped with a magnetic stir bar, the photocatalyst (1.5 mol%), nickel salt (10 mol%), chiral ligand (15 mol%), ammonium salt (10 mol%), base (0.5 equiv), additive (0.5 equiv), 2-(4-methylphenyl)-1-tosylaziridine (**1a**, 28.7 mg, 0.10 mmol, 1.0 equiv), and diethyl 2,6-dimethyl-4-(pyrrolidine-1-carbonyl)-1,4-dihydropyridine-3,5-dicarboxylate (**2a**, 70.0 mg, 0.20 mmol, 2.0 equiv) were subsequently added. The vial was sealed with an aluminum crimp, evacuated, and backfilled with argon three times. Anhydrous α,α,α-trifluorotoluene (2.8 mL) and $CH_3CN$ (0.7 mL) were then added via syringe under an argon atmosphere to the preassembled Ni/L manifold. The reaction mixture was stirred at 850 rpm and irradiated at 451 nm in a blue LED photoreactor at 30 °C for 16 hours. Upon completion, the mixture was filtered through a short column of silica gel using ethyl acetate as the eluent. An internal standard (dodecane) was introduced to determine the GC yield. The filtrate was concentrated and purified by column chromatography on silica gel (*i*PrOAc/acetone = 20:1). The enantiomeric ratio was determined by SFC analysis using a chiral stationary phase.

**Table S1: Optimization of the Reaction Conditions**

| entry | deviation from standard conditions | **3a** yield (%)[a] | er[b] |
|---|---|---|---|
| 1 | none | 75(74)[c] | 96:4 |
| 2 | no $KClO_4$ | 65 | 96:4 |
| 3 | no 5-TCzBN or in the darkness | 0 | - |
| 4 | **L2** instead of **L1** | 50 | 91.5:8.5 |
| 5 | **L3** instead of **L1** | 36 | 76.5:23.5 |
| 6 | **L4** instead of **L1** | 20 | 48.5:51.5 |
| 7 | **L5** instead of **L1** | 9 | 33.5:66.5 |
| 8 | **L6** instead of **L1** | 7 | 62.5:37.5 |
| 9 | **L7** instead of **L1** | 41 | 89.5:10.5 |
| 10 | **L8** instead of **L1** | 23 | 11.5:88.5 |
| 11 | 4-CzIPN instead of 5-TCzBN | 26 | 90:10 |
| 12 | 4-TCzBN instead of 5-TCzBN | 37 | 93:7 |
| 13 | $[Ir(dtbpy(ppy)_2)]PF_6$ instead of 5-TCzBN | 3 | 95:5 |
| 14 | $NiCl_2$·glyme instead of $NiBr_2$·diglyme | 30 | 96:4 |
| 15 | $Ni(cod)_2$ instead of $NiBr_2$·diglyme | 41 | 96:4 |
| 16 | TBAB instead of $BnBu_3NI$ | 3 | 89.5:10.5 |
| 17 | TPAI instead of $BnBu_3NI$ | 65 | 95:5 |
| 18 | $Na_2CO_3$ instead of 4-$CF_3C_6H_4COONa$ | 14 | 77.5:22.5 |
| 19 | PhCOONa instead of 4-$CF_3C_6H_4COONa$ | 37 | 92:8 |
| 20 | 4-$OMeC_6H_4COONa$ instead of 4-$CF_3C_6H_4COONa$ | 38 | 95.5:4.5 |
| 21 | KCl instead of $KClO_4$ | 25 | 85.5:14.5 |
| 22 | $KPF_6$ instead of $KClO_4$ | 41 | 83.5:16.5 |
| 23 | $CH_3CN$ as solvent | 6 | 78.5:21.5 |
| 24 | $PhCF_3$ as solvent | 46 | 93:7 |
| 25 | 0.05 M instead of 0.028 M | 14 | 95:5 |

Conditions: **1a** (0.10 mmol), **2a** (0.2 mmol), $NiBr_2$·diglyme (10 mol%), **L1** (15 mol%), $BnBu_3NI$ (10 mol%), 4-$CF_3C_6H_4COONa$ (0.5 equiv), $KClO_4$ (0.5 equiv), $PhCF_3/CH_3CN$(3/1)(0.028 M), blue LED (451 nm), 30 °C, 16 h. [a]GC yield using 1-dodecane as internal standard. [b]er values were determined by SFC analysis. [c]Isolated yield, average of two independent runs. $BnBu_3NI$ = Benzyltributylammonium iodide. TPAI = Tetrapentylammonium iodide. TBAB = Tetrabutylammonium bromide. Cz = carbazole.

## 3. Synthesis of Starting Materials

### 3.1 Synthesis of aziridines

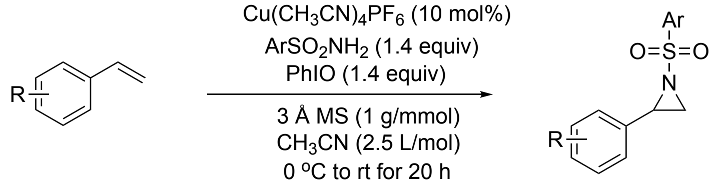


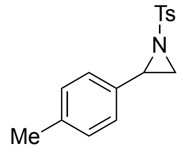

**1a**

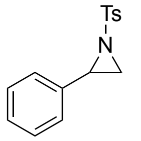

**1b**

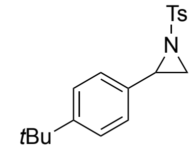

**1c**

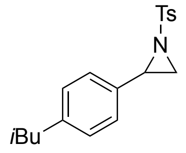

**1d**

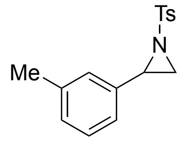

**1e**

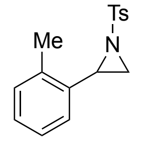

**1f**

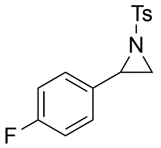

**1g**

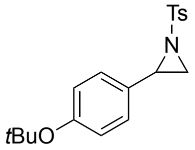

**1h**

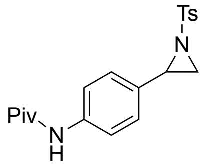

**1i**

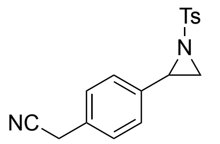

**1j**

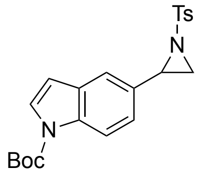

**1k**

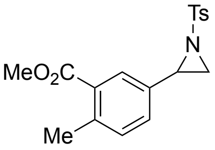

**1l**

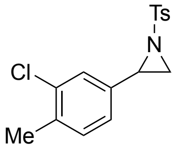

**1m**

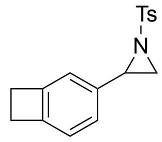

**1n**

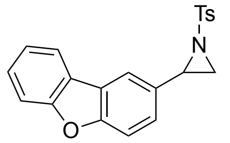

**1o**

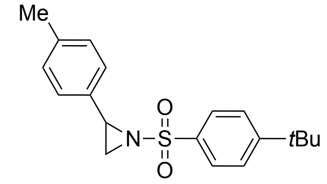

**1p**

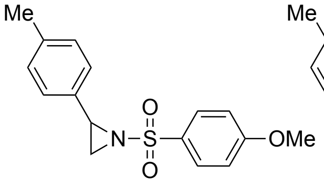

**1q**

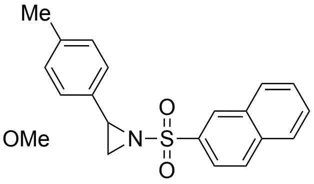

**1r**

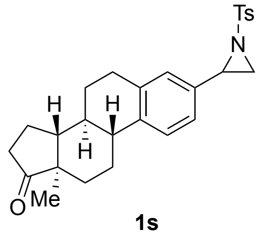

**1s**

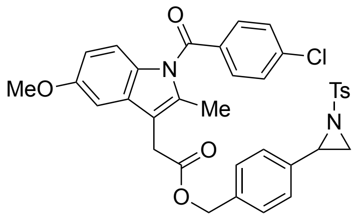

**1t**

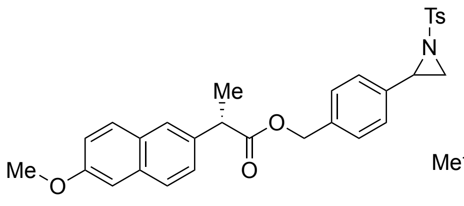

**1u**

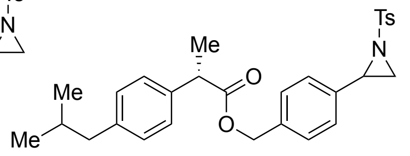

**1v**

**General procedure A for aziridine synthesis**. An oven-dried flask equipped with a stir bar was charged with $ArSO_2NH_2$ (1.4 equiv), $Cu(MeCN)_4PF_6$ (10 mol%), and activated 3 Å molecular sieves (***Note:*** 1 g/mmol alkene). After the addition of the alkene (1.0 equiv) and MeCN (2.5 mL/mmol alkene), the mixture was cooled to 0 °C, and iodosylbenzene (1.4 equiv) was added in one portion. The reaction mixture was then warmed to room temperature and stirred for 20 hours. Upon completion, the mixture

was filtered through a short column of silica gel using ethyl acetate as the eluent. The filtrate was concentrated and purified by column chromatography on silica gel (using hexane/ethyl acetate) to afford the corresponding aziridines. **1a**,[1] **1b**,[3] **1c**,[4] **1e**,[2] **1f**,[3] **1g**,[3] **1k**,[4] and **1s**[3] were prepared according to the above procedure, and all spectroscopic data were in agreement with previously reported data. **1h**[5] and **1i**[3] were prepared according to known literature procedures and their analytical data are in agreement with the reported data.

**2-(4-isobutylphenyl)-1-tosylaziridine (1d):** Following the General procedure A, 1-isobutyl-4-vinylbenzene (801 mg, 5.0 mmol), iodosylbenzene (1.54 g, 7.0 mmol), and $TsNH_2$ (1.20 g, 7.0 mmol) were used, affording the title compound as a yellow oil (550 mg, 33% yield) by using Hexane/EtOAc (8:1 to 5:1) as eluent. **$^{1}$H NMR** (400 MHz, $CDCl_3$) δ 7.89 – 7.85 (m, 2H), 7.35 – 7.31 (m, 2H), 7.15 – 7.09 (m, 2H), 7.10 – 7.03 (m, 2H), 3.76 (dd, *J* = 7.2, 4.5 Hz, 1H), 2.96 (d, *J* = 7.2 Hz, 1H), 2.46 – 2.40 (m, 5H), 2.38 (d, *J* = 4.5 Hz, 1H), 1.89 – 1.74 (m, 1H), 0.87 (d, *J* = 6.6 Hz, 6H). **$^{13}$C NMR** (101 MHz, $CDCl_3$) δ 144.7, 142.1, 135.2, 132.4, 129.9, 129.4, 128.1, 126.5, 45.2, 41.2, 36.0, 30.3, 22.4, 21.8. **HRMS** (ESI) calcd. for ($C_{19}H_{24}NO_2S$) $[M+H]^+$: 330.1522, found 330.1522. **IR (neat)** 2958, 2923, 2868, 1958, 1453, 1384, 1327, 1186, 1162, 1094, 913, 816, 722, 695, 664, 575, 552.

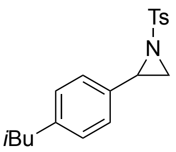

**1d**

**2-(4-(1-tosylaziridin-2-yl)phenyl)acetonitrile (1j):** Following General procedure A, 2-(4-vinylphenyl)acetonitrile (716 mg, 5.0 mmol), iodosylbenzene (1.54 g, 7.0 mmol), and $TsNH_2$ (1.20 g, 7.0 mmol) were used, affording the title compound as a yellow solid (581 mg, 37% yield) by using Hexane/EtOAc (5:1 to 3:1) as eluent. **M.p.**: 77-81 °C. **$^{1}$H NMR** (400 MHz, $CDCl_3$) δ 7.88 – 7.84 (m, 2H), 7.36 – 7.32 (m, 2H), 7.30 – 7.20 (m, 4H), 3.76 (dd, *J* = 7.2, 4.5 Hz, 1H), 3.71 (s, 2H), 2.99 (d, *J* = 7.2 Hz, 1H), 2.44 (s, 3H), 2.36 (d, *J* = 4.4 Hz, 1H). **$^{13}$C NMR** (101 MHz, $CDCl_3$) δ 145.0, 135.4, 135.0, 130.2, 123.0, 128.4, 128.1, 127.5, 117.7, 40.6, 36.2, 23.5, 21.8. **HRMS** (ESI) calcd. for ($C_{17}H_{16}N_2NaO_2S$) $[M+Na]^+$: 335.0825, found 335.0823. **IR (neat)**: 3359, 3273, 1597, 1518, 1454, 1417, 1383, 1324, 1186, 1160, 1094, 912, 815, 724, 694, 666, 571, 555, 541.

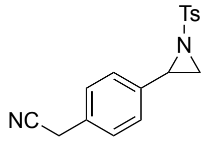

**1j**

**2-(bicyclo[4.2.0]octa-1(6),2,4-trien-3-yl)-1-tosylaziridine (1l):** Following General procedure A, 2-3-vinylbicyclo[4.2.0]octa-1(6),2,4-triene (650 mg, 5.0 mmol), iodosylbenzene (1.54 g, 7.0 mmol), and $TsNH_2$ (1.20 g, 7.0 mmol) were used, affording the title compound as a yellow solid (924 mg, 62% yield) by using Hexane/EtOAc (8:1 to 5:1) as eluent. **M.p.**: 45-48 °C. **$^{1}$H NMR** (400 MHz, $CDCl_3$) δ 7.88 – 7.85 (m, 2H), 7.34 – 7.31 (m, 2H), 7.06 (dd, *J* = 7.5, 1.4 Hz, 1H), 6.96 (d, *J* = 7.6 Hz, 1H), 6.90 (s, 1H), 3.75 (dd, *J* = 7.2, 4.5 Hz, 1H), 3.17 – 3.07 (m, 4H), 2.96 (d, *J* = 7.2 Hz, 1H), 2.43 (s, 3H), 2.37 (d, *J* = 4.5 Hz, 1H). **$^{13}$C NMR** (101 MHz, $CDCl_3$) δ 146.3, 146.2, 144.7, 135.3, 133.7, 129.9, 128.1, 125.6, 122.7, 120.7, 41.9, 36.1, 29.6,

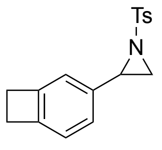

**1l**

29.4, 21.8. **HRMS** (ESI) calcd. for ($C_{17}H_{18}NO_2S$) $[M+H]^+$: 300.1053, found 300.1041. **IR (neat)**: 3028, 2998, 2966, 2926, 1597, 1454, 1324, 1292, 1204, 1094, 928, 897, 878, 816, 722, 693, 666, 578.

**methyl 2-methyl-5-(1-tosylaziridin-2-yl)benzoate (1m):** Following General procedure A, 2-3-methyl 2-methyl-5-vinylbenzoate (880 mg, 5.0 mmol), iodosylbenzene (1.54 g, 7.0 mmol), and $TsNH_2$ (1.20 g, 7.0 mmol) were used, affording the title compound as a colorless solid (1.11 g, 64% yield) by using Hexane/EtOAc (8:1 to 5:1) as eluent. **M.p.**: 69-74 °C. **$^1$H NMR** (400 MHz, $CDCl_3$) δ 7.88 – 7.84 (m, 2H), 7.75 (d, *J* = 2.0 Hz, 1H), 7.34 – 7.31 (m, 2H), 7.23 (dd, *J* = 7.9, 2.0 Hz, 1H), 7.16 (d, *J* = 7.9 Hz, 1H), 3.87 (s, 3H), 3.75 (dd, *J* = 7.2, 4.4 Hz, 1H), 2.98 (d, *J* = 7.2 Hz, 1H), 2.54 (s, 3H), 2.43 (s, 3H), 2.39 (d, *J* = 4.4 Hz, 1H). **$^{13}$C NMR** (101 MHz, $CDCl_3$) δ 167.6, 144.9, 140.5, 135.0, 132.9, 132.2, 130.0, 129.92, 129.90, 128.9, 128.1, 52.0, 40.5, 36.0, 21.8, 21.5. **HRMS** (ESI) calcd. for ($C_{18}H_{19}NNaO_4S$) $[M+Na]^+$: 368.0927, found 368.0912. **IR (neat)**: 3033, 2953, 2926, 2855, 1721, 1597, 1498, 1437, 1381, 1325, 1304, 1255, 1229, 1193, 1161, 1083, 971, 923, 871, 817, 785, 724, 693, 665, 584, 558.

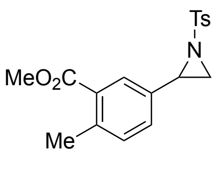


**1m**

**2-(3-chloro-4-methylphenyl)-1-tosylaziridine (1n):** Following General procedure A, 2-chloro-1-methyl-4-vinylbenzene (760 mg, 5.0 mmol), iodosylbenzene (1.54 g, 7.0 mmol), and $TsNH_2$ (1.20 g, 7.0 mmol) were used, affording the title compound as a colorless solid (825 mg, 51% yield) by using Hexane/EtOAc (8:1 to 5:1) as eluent. **M.p.**: 87-90 °C. **$^1$H NMR** (400 MHz, $CDCl_3$) δ 7.88 – 7.84 (m, 2H), 7.34 (d, *J* = 8.1 Hz, 2H), 7.18 – 7.12 (m, 2H), 7.01 (dd, *J* = 7.9, 1.8 Hz, 1H), 3.70 (dd, *J* = 7.2, 4.4 Hz, 1H), 2.96 (d, *J* = 7.2 Hz, 1H), 2.44 (s, 3H), 2.34 (d, *J* = 4.4 Hz, 1H), 2.32 (s, 3H). **$^{13}$C NMR** (101 MHz, $CDCl_3$) δ 144.9, 136.3, 135.0, 134.7, 134.5, 131.2, 129.9, 128.1, 127.1, 125.0, 40.3, 36.1, 21.8, 19.9. **HRMS** (ESI) calcd. for ($C_{16}H_{17}ClNO_2S$) $[M+H]^+$: 322.0633, found 322.0661. **IR (neat)**: 3062, 2995, 2952, 2924, 1597, 1568, 1550, 1453, 1406, 1374, 1325, 1306, 1291, 1234, 1121, 1161, 1093, 1051, 992, 920, 857, 815, 723, 694, 669, 593, 556.

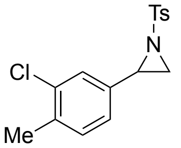


**1n**

**2-(dibenzo[*b,d*]furan-2-yl)-1-tosylaziridine (1o):** Following General procedure A, 2-vinyldibenzo[*b,d*]furan (970 mg, 5.0 mmol), iodosylbenzene (1.54 g, 7.0 mmol), and $TsNH_2$ (1.20 g, 7.0 mmol) were used, affording the title compound as a white solid (1.10 g, 61% yield) by using Hexane/EtOAc (5:1 to 3:1) as eluent. **M.p.**: 160-164 °C. **$^1$H NMR** (400 MHz, $CDCl_3$) δ 7.95 – 7.75 (m, 4H), 7.57 – 7.27 (m, 7H), 3.94 (dd, *J* = 7.3, 4.3 Hz, 1H), 3.06 (d, *J* = 7.1 Hz, 1H), 2.49 (d, *J* = 4.4 Hz, 1H), 2.43 (s, 3H). **$^{13}$C NMR** (101 MHz, $CDCl_3$) δ 156.7, 156.2, 144.8, 135.2, 129.9, 129.8, 128.1, 127.7, 125.8, 124.7, 123.8, 123.0, 120.9, 118.9, 111.9, 111.8, 41.3, 36.5, 21.8. **HRMS** (ESI) calcd. for ($C_{21}H_{18}NO_3S$) $[M+H]^+$: 364.1002, found 364.0998. **IR (neat)**: 3059, 1710, 1597, 1483, 1450, 1324, 1197, 1160, 1093, 1022, 921, 871, 841, 814, 750, 737, 716, 672, 612, 570, 557.

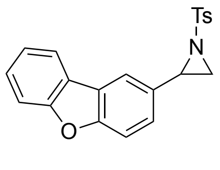


**1o**

**1-((4-(tert-butyl)phenyl)sulfonyl)-2-(4-fluorophenyl)aziridine (1p):** Following General procedure A, 1-methyl-4-vinylbenzene (590 mg, 5.0 mmol), iodosylbenzene (1.54 g, 7.0 mmol), and 4-(tert-butyl)benzenesulfonamide (1.49 g, 7.0 mmol) were used, affording the title compound as a white solid (605 mg, 36% yield) by using Hexane/EtOAc (8:1 to 5:1) as eluent. **M.p.**: 95-99 °C. **$^1$H NMR** (400 MHz, $CDCl_3$) δ 7.93 – 7.89 (m, 2H), 7.57 – 7.52 (m, 2H), 7.13 – 7.09 (m, 4H), 3.78 (dd, *J* = 7.2, 4.5 Hz, 1H), 2.97 (d, *J* = 7.2 Hz, 1H), 2.39 (d, *J* = 4.5 Hz, 1H), 2.32 (s, 3H), 1.35 (s, 9H). **$^{13}$C NMR** (101 MHz, $CDCl_3$) δ 157.6, 138.3, 135.2, 132.2, 129.4, 127.9, 126.7, 126.2, 41.1, 36.0, 35.4, 31.2, 21.3. **HRMS** (ESI) calcd. for ($C_{19}H_{24}NO_2S$) $[M+H]^+$: 330.1522, found 330.1529. **IR (neat)**: 2964, 2870, 1595, 1518, 1461, 1399, 1381, 1326, 1293, 1189, 1163, 1111, 1090, 979, 912, 840, 819, 763, 689, 644, 633, 579, 554.

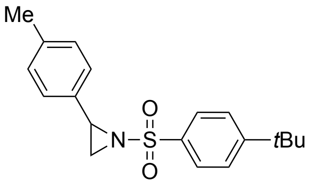


**1p**

**2-(4-fluorophenyl)-1-((4-methoxyphenyl)sulfonyl)aziridine (1q):** Following General procedure A, 1-methyl-4-vinylbenzene (590 mg, 5.0 mmol), iodosylbenzene (1.54 g, 7.0 mmol), and 4-methoxybenzenesulfonamide (1.31 g, 7.0 mmol) were used, affording the title compound as a white solid (500 mg, 33% yield) by using Hexane/EtOAc (5:1 to 3:1) as eluent. **M.p.**: 133-136 °C. **$^1$H NMR** (400 MHz, $CDCl_3$) δ 7.93 – 7.89 (m, 2H), 7.10 (s, 4H), 7.01 – 6.97 (m, 2H), 3.86 (s, 3H), 3.72 (dd, *J* = 7.2, 4.5 Hz, 1H), 2.95 (d, *J* = 7.2 Hz, 1H), 2.37 (d, *J* = 4.4 Hz, 1H), 2.31 (s, 3H). **$^{13}$C NMR** (101 MHz, $CDCl_3$) δ 163.8, 138.3, 132.2, 130.2, 129.7, 129.4, 126.6, 114.5, 55.8, 41.2, 35.9, 21.3. **HRMS** (ESI) calcd. for ($C_{16}H_{18}NO_3S$) $[M+H]^+$: 304.1002, found 304.1001. **IR (neat)**: 3014, 2983, 1596, 1579, 1498, 1461, 1326, 1311, 1262, 1184, 1156, 1095, 1023, 911, 823, 807, 734, 697, 577.

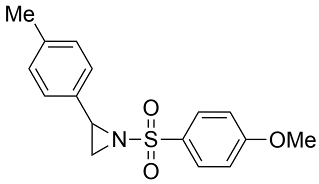


**1q**

**2-(4-fluorophenyl)-1-(naphthalen-2-ylsulfonyl)aziridine (1r):** Following General procedure A, 1-methyl-4-vinylbenzene (590 mg, 5.0 mmol), iodosylbenzene (1.54 g, 7.0 mmol), and naphthalene-2-sulfonamide (1.45 g, 7.0 mmol) were used, affording the title compound as a white solid (725 mg, 44% yield) by using Hexane/EtOAc (8:1 to 5:1) as eluent. **M.p.**: 144-147 °C. **$^1$H NMR** (400 MHz, $CDCl_3$) δ 8.55 (s, 1H), 8.01 – 7.94 (m, 3H), 7.93 – 7.90 (m, 1H), 7.70 – 7.57 (m, 2H), 7.13 – 7.07 (m, 4H), 3.85 (dd, *J* = 7.2, 4.5 Hz, 1H), 3.06 (d, *J* = 7.2 Hz, 1H), 2.43 (d, *J* = 4.5 Hz, 1H), 2.30 (s, 3H). **$^{13}$C NMR** (101 MHz, $CDCl_3$) δ 138.4, 135.4, 135.1, 132.1, 132.0, 129.6, 129.53, 129.50, 129.4, 129.3, 128.1, 127.7, 126.6, 123.0, 41.4, 36.1, 21.2. **HRMS** (ESI) calcd. for ($C_{19}H_{18}NO_2S$) $[M+H]^+$: 324.1053, found 324.1058. **IR (neat)**: 3056, 3024, 1519, 1457, 1382, 1348, 1322, 1189, 1159, 1131, 1080, 911, 860, 816, 750, 697, 659, 567, 554, 477.

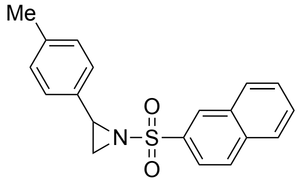


**1r**

**4-(1-tosylaziridin-2-yl)benzyl 2-(1-(4-chlorobenzoyl)-5-methoxy-2-methyl-1H-indol-3-yl)acetate (1t):** Following General procedure A, 4-vinylbenzyl 2-(1-(4-chlorobenzoyl)-5-methoxy-2-methyl-1H-indol-3-yl)acetate (2.37 g, 5.0 mmol), iodosylbenzene (1.54 g, 7.0 mmol), and $TsNH_2$ (1.20 g, 7.0 mmol) were used, affording the title compound as a yellow oil (699 mg, 22% yield) by using Hexane/EtOAc (8:1 to 5:1) as eluent. **$^1$H NMR** (400 MHz, $CDCl_3$) δ 7.88 – 7.85 (m, 2H), 7.67 – 7.63 (m, 2H), 7.48 – 7.44 (m, 2H), 7.35 – 7.32 (m, 2H), 7.26 – 7.15 (m, 4H), 6.90 – 6.85 (m, 2H), 6.70 – 6.62 (m, 1H), 5.09 (s, 2H), 3.77 (dd, *J* = 7.2, 4.5 Hz, 1H), 3.73 (s, 3H), 3.69 (s, 2H), 2.98 (d, *J* = 7.2 Hz, 1H), 2.44 (s, 3H), 2.38 – 2.34 (m, 4H). **$^{13}$C NMR** (101 MHz, $CDCl_3$) δ 170.7, 168.4, 156.2, 144.9, 139.5, 136.1, 135.4, 135.1, 134.0, 131.3, 130.9, 130.7, 130.0, 129.3, 128.5, 128.1, 126.9, 115.1, 112.5, 111.9, 101.4, 66.4, 55.8, 40.8, 36.2, 30.5, 21.8, 13.5. **HRMS** (ESI) calcd. for ($C_{35}H_{32}ClN_2O_6S$) $[M+H]^+$: 642.1664, found 643.1669. **IR (neat)**: 3356, 3263, 3061, 2929, 2836, 1736, 1681, 1597, 1478, 1456, 1400, 1356, 1323, 1262, 1223, 1161, 1091, 1068, 1036, 1015, 911, 815, 755, 712, 693, 666, 573, 557.

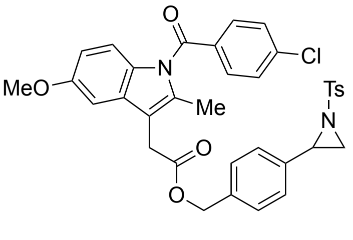


1t

**4-(1-tosylaziridin-2-yl)benzyl (2*S*)-2-(6-methoxynaphthalen-2-yl)propanoate (1u):** Following General procedure A, 4-vinylbenzyl (*S*)-2-(6-methoxynaphthalen-2-yl)propanoate (3.46 g, 10.0 mmol), iodosylbenzene (1.54 g, 7.0 mmol), and $TsNH_2$ (1.20 g, 7.0 mmol) were used, affording the title compound as a yellow oil (2.09 g, 41% yield) by using Hexane/EtOAc (8:1 to 5:1) as eluent. **$^1$H NMR** (400 MHz, $CDCl_3$) δ 7.88 – 7.85 (m, 2H), 7.71 – 7.62 (m, 3H), 7.38 (dd, *J* = 8.5, 1.9 Hz, 1H), 7.34 – 7.31 (m, 2H), 7.17 – 7.12 (m, 6H), 5.11 – 5.03 (m, 2H), 3.92 (s, 3H), 3.90 – 3.86 (m, 1H), 3.74 (dd, *J* = 7.2, 4.4 Hz, 1H), 2.97 (d, *J* = 7.2 Hz, 1H), 2.43 (s, 3H), 2.34 (dd, *J* = 4.4, 2.1 Hz, 1H), 1.58 (d, *J* = 7.1 Hz, 3H). **$^{13}$C NMR** (101 MHz, $CDCl_3$) δ 174.4, 157.8, 144.8, 136.3, 135.5, 135.04, 135.01, 133.8, 129.9, 129.3, 129.0, 128.2, 128.0, 127.2, 126.8, 126.3, 126.1, 119.1, 105.7, 66.0, 55.4, 45.5, 40.8, 36.0, 21.7, 18.6. **HRMS** (ESI) calcd. for ($C_{30}H_{29}NNaO_5S$) $[M+Na]^+$: 538.1659, found 538.1659. **IR (neat)**: 2977, 2937, 1732, 1606, 1453, 1392, 1325, 1265, 1231, 1161, 1092, 911, 814, 712, 573.

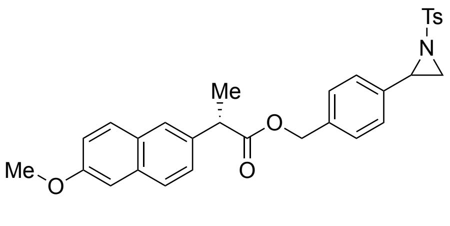


1u

**4-(1-tosylaziridin-2-yl)benzyl (2*S*)-2-(4-isobutylphenyl)propanoate (1v):** Following General procedure A, 4-vinylbenzyl (*S*)-2-(4-isobutylphenyl)propanoate (1.61 g, 5.0 mmol), iodosylbenzene (1.54 g, 7.0 mmol), and $TsNH_2$ (1.20 g, 7.0 mmol) were used, affording the title compound as a colorless oil (1.28 g, 52% yield) by using Hexane/EtOAc (8:1 to 5:1) as eluent. **$^1$H NMR** (400 MHz, $CDCl_3$) δ 7.88 – 7.85 (m, 2H), 7.35 – 7.32 (m, 2H), 7.20 – 7.17 (m, 2H), 7.15 (s, 4H), 7.10 – 7.07 (m, 2H), 5.06 (d, *J* = 2.0 Hz, 2H), 3.77 – 3.70 (m, 2H), 2.98 (d, *J* = 7.2

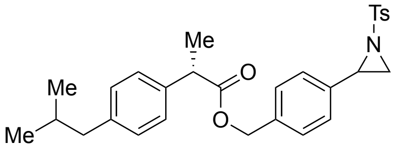


1v

Hz, 1H), 2.46 (d, $J$ = 7.2 Hz, 2H), 2.44 (s, 3H), 2.35 (d, $J$ = 4.4 Hz, 1H), 1.93 – 1.78 (m, 1H), 1.50 (d, $J$ = 7.2 Hz, 3H), 0.91 (d, $J$ = 6.6 Hz, 6H). **$^{13}$C NMR** (101 MHz, $CDCl_3$) δ 174.5, 144.8, 140.7, 137.6, 136.5, 135.1, 135.0, 129.9, 129.4, 128.1, 128.0, 127.3, 126.8, 65.9, 45.2, 45.1, 40.8, 36.1, 30.3, 22.5, 21.8, 18.5. **HRMS** (ESI) calcd. for ($C_{29}H_{34}NO_4S$) $[M+H]^+$: 492.2203, found 492.2186. **IR (neat)**: 3027, 2953, 2933, 2868, 1735, 1598, 1513, 1453, 1381, 1327, 1186, 1162, 1093, 1020, 981, 912, 815, 723, 694, 668, 574, 554.

## 3.2 Synthesis of 1,4-Dihydropyridines (DHP)

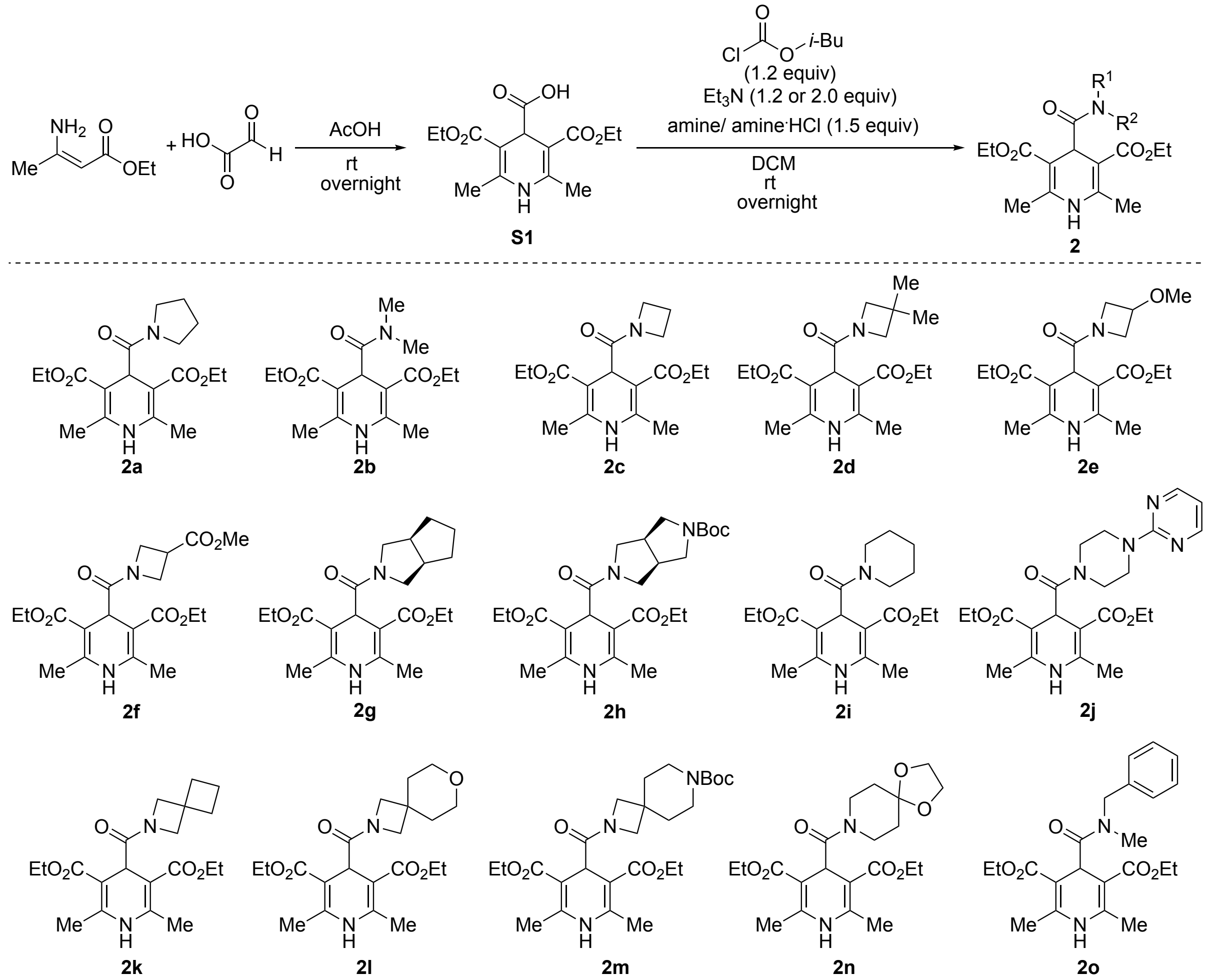


**General procedure B for the preparation of 1,4-Dihydropyridines (DHP)**: Glyoxylic acid (50% wt, 14.8 g, 100 mmol, 1.0 equiv) was added portion wise at 0 °C to a solution of ethyl-3-aminocrotonate (25.8 g, 200 mmol, 2.0 equiv) in 50 mL of glacial acetic acid. The reaction mixture rapidly became yellow, and a precipitate deposited. After the evolution of heat had ceased, the reaction mixture was left stirring overnight at rt. The solid was collected by filtration and washed with acetic acid and water. The solids were dried overnight under reduced pressure to obtain the pure acid **S1** as a light-yellow powder (13.2 g, 44% yield). In a round bottom flask, the carboxylic acid **S1** (1.0 equiv) was suspended in DCM (0.2 M) followed by the addition of triethylamine (1.2 equiv or 2.0 equiv when amine hydrochloride salts are used). The resulting yellow solution was cooled down to 0 °C and isobutyl chloroformate (1.2 equiv) was then added dropwise. After 10 minutes, the mixture was allowed to warm up to ambient temperature and stirred for 20 minutes. Next, the amine (1.5 equiv) was added and the resulting solution stirred at ambient temperature overnight. After completion of the reaction (as judged by TLC, EtOAc), the solution was concentrated. The remaining residue was purified by flash column chromatography (EtOAc/MeOH (10:1) and recrystallized from DCM and hexane to provide the desired 1,4-dihydropyridines **2**. **2a,**[6] **2b,**[7] **2i,**[6] and **2o**[6] were synthesized according to reported procedures and all spectroscopic data were in agreement with those reported.

**diethyl 4-(azetidine-1-carbonyl)-2,6-dimethyl-1,4-dihydropyridine-3,5-dicarboxylate (2c):** Following General Procedure B, 3,5-bis(ethoxycarbonyl)-2,6-dimethyl-1,4-dihydropyridine-4-carboxylic acid (2.00 g, 6.72 mmol), azetidine (574 mg, 10.1 mmol), affording the title compound as a white solid (1.62 g, 72% yield) by using EtOAc/MeOH (10:1) as eluent. **M.p.**: 202-205 °C. **$^{1}$H NMR** (400 MHz, $CDCl_3$) δ 8.56 (s, 1H), 4.61 (t, $J$ = 7.8 Hz, 2H), 4.54 (s, 1H), 4.20 (q, $J$ = 7.1 Hz, 4H), 3.98 (t, $J$ = 7.8 Hz, 2H), 2.27 – 2.19 (m, 2H), 2.18 (s, 6H), 1.30 (t, $J$ = 7.1 Hz, 6H). **$^{13}$C NMR** (101 MHz, $CDCl_3$) δ 174.5, 167.8, 148.2, 97.9, 59.9, 51.2, 48.2, 36.8, 19.4, 15.1, 14.7. **HRMS** (ESI) calcd. for ($C_{17}H_{25}N_2O_5$) $[M+H]^+$: 337.1758, found 337.1764. **IR (neat)**: 3282, 3200, 3088, 3075, 2977, 1697, 1679, 1620, 1503, 1461, 1443, 1384, 1368, 1329, 1293, 1268, 1212, 1115, 1093, 1052, 1024.

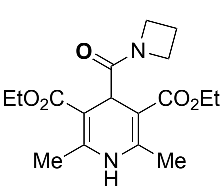

**2c**

**diethyl 4-(3,3-dimethylazetidine-1-carbonyl)-2,6-dimethyl-1,4-dihydropyridine-3,5-dicarboxylate (2d):** Following General Procedure B, 3,5-bis(ethoxycarbonyl)-2,6-dimethyl-1,4-dihydropyridine-4-carboxylic acid (2.00 g, 6.72 mmol), 3,3-dimethylazetidine hydrochloride (1.23 g, 10.08 mmol), affording the title compound as a yellow solid (1.78 g, 70% yield) by using EtOAc/MeOH (10:1) as eluent. **M.p.**: 237-242 °C. **$^{1}$H NMR** (400 MHz, $CDCl_3$) δ 8.77 (s, 1H), 4.56 (s, 1H), 4.30 – 4.07 (m, 6H), 3.61 (s, 2H), 2.17 (s, 6H), 1.33 – 1.23 (m, 12H). **$^{13}$C NMR** (101 MHz, $CDCl_3$) δ 174.9, 167.7, 148.4, 97.7, 63.1, 60.1, 59.9, 36.9, 30.3, 27.1, 19.4, 14.8. **HRMS** (ESI) calcd. for ($C_{19}H_{29}N_2O_5$) $[M+H]^+$: 365.2071, found 365.2074. **IR (neat)**: 3283, 3203, 3084, 2984, 2949, 2873, 1698, 1673, 1639, 1614, 1502, 1455, 1369, 1325, 1294, 1262, 1205, 1152, 1119, 1109, 1096, 1054, 1024, 763.

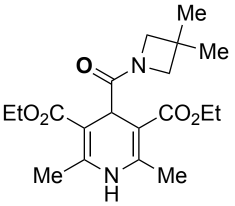

**2d**

**diethyl 4-(3-methoxyazetidine-1-carbonyl)-2,6-dimethyl-1,4-dihydropyridine-3,5-dicarboxylate (2e):** Following General Procedure B, 3,5-bis(ethoxycarbonyl)-2,6-dimethyl-1,4-dihydropyridine-4-carboxylic acid (2.00 g, 6.72 mmol), 3-methoxyazetidine hydrochloride (1.39 g, 10.08 mmol), affording the title compound as a yellow solid (617.3 mg, 25% yield) by using EtOAc/MeOH (10:1) as eluent. **M.p.**: 193-196 °C. **$^{1}$H NMR** (400 MHz, $CDCl_3$) δ 8.37 (s, 1H), 4.82 – 4.73 (m, 1H), 4.55 (s, 1H), 4.44 – 4.40 (m, 1H), 4.24 – 4.15 (m, 5H), 4.16 – 4.07 (m, 1H), 3.85 – 3.80 (m, 1H), 3.30 (s, 3H), 2.18 (d, $J$ = 5.1 Hz, 6H), 1.35 – 1.25 (m, 6H). **$^{13}$C NMR** (101 MHz, $CDCl_3$) δ 174.9, 167.9, 167.5, 148.2, 98.0, 97.9, 68.9, 60.04, 60.01, 58.3, 56.2, 55.1, 37.3, 19.5, 19.3, 14.8, 14.7. **$^{1}$H NMR** (500 MHz, DMSO-$d_6$, 393K) δ 8.29 (s, 1H), 4.43 (s, 1H), 4.36 – 4.09 (m, 7H), 3.87 (brs, 2H), 3.25 (s, 3H), 2.21 (s, 6H), 1.25 (t, $J$ = 7.1 Hz, 6H). **$^{13}$C NMR** (126 MHz, DMSO-$d_6$, 393K) δ 173.0, 166.1, 146.2, 97.3, 68.0, 58.5, 54.6, 36.5, 17.6, 13.6. **Note:** At 393 K, the rotameric signals coalesce and the alkyl carbon adjacent to the amide nitrogen is broadened beyond detection. **HRMS** (ESI) calcd. for ($C_{18}H_{27}N_2O_6$) $[M+H]^+$: 367.1864, found 367.1857. **IR (neat)**: 3291, 3200, 3090,

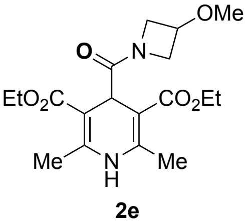

**2e**

2983, 2955, 2874, 2018, 1699, 1678, 1625, 1501, 1458, 1385, 1368, 1330, 1299, 1267, 1209, 1120, 1091, 1051, 1024.

**diethyl 4-(3-(methoxycarbonyl)azetidine-1-carbonyl)-2,6-dimethyl-1,4-dihydropyridine-3,5-dicarboxylate (2f):** Following General Procedure B, 3,5-bis(ethoxycarbonyl)-2,6-dimethyl-1,4-dihydropyridine-4-carboxylic acid (2.00 g, 6.72 mmol), methyl azetidine-3-carboxylate hydrochloride (1.52 g, 10.08 mmol), affording the title compound as a white solid (1.06 g, 40% yield) by using EtOAc/MeOH (10:1) as eluent. **M.p.**: 184-189 °C. **$^1$H NMR** (400 MHz, $CDCl_3$) δ 8.43 (s, 1H), 4.87 – 4.78 (m, 1H), 4.76 – 4.68 (m, 1H), 4.53 (s, 1H), 4.29 – 4.09 (m, 6H), 3.75 (s, 3H), 3.46 – 3.34 (m, 1H), 2.17 (dd, *J* = 5.8, 2.6 Hz, 6H), 1.30 (t, *J* = 7.1 Hz, 6H). **$^{13}$C NMR** (101 MHz, $CDCl_3$) δ 174.8, 173.0, 167.8, 167.3, 148.3, 148.2, 97.8, 97.7, 60.1, 53.5, 52.4, 50.6, 37.0, 31.7, 19.5, 19.3, 14.73, 14.69. **$^1$H NMR** (500 MHz, DMSO-$d_6$, 393K) δ 8.33 (s, 1H), 4.42 (s, 1H), 4.40 – 3.85 (m, 8H), 3.70 (s, 3H), 3.50 – 3.40 (m, 1H), 2.21 (s, 6H), 1.25 (t, *J* = 7.1 Hz, 6H). **$^{13}$C NMR** (126 MHz, DMSO-$d_6$, 393K) δ 172.9, 172.0, 166.1, 146.3, 97.1, 58.6, 51.1, 36.2, 30.9, 17.6, 13.6. **Note:** At 393 K, the rotameric signals coalesce and the alkyl carbon adjacent to the amide nitrogen is broadened beyond detection. **HRMS** (ESI) calcd. for ($C_{19}H_{27}N_2O_7$) $[M+H]^+$: 395.1813, found 395.1816. **IR (neat)**: 3278, 3212, 3085, 2979, 1741, 1698, 1677, 1627, 1501, 1460, 1368, 1329, 1297, 1268, 1208, 1116, 1092, 1052, 1022.

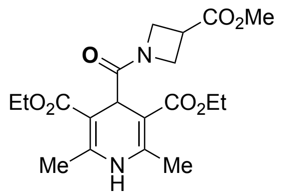

**2f**

**diethyl 2,6-dimethyl-4-((3aR,6aS)-octahydrocyclopenta[c]pyrrole-2-carbonyl)-1,4-dihydropyridine-3,5-dicarboxylate (2g):** Following General Procedure B, 3,5-bis(ethoxycarbonyl)-2,6-dimethyl-1,4-dihydropyridine-4-carboxylic acid (2.00 g, 6.72 mmol), (3a*R*,6a*S*)-octahydrocyclopenta[*c*]pyrrole hydrochloride (1.49 g, 10.08 mmol), affording the title compound as a white solid (808 mg, 31% yield) by using EtOAc/MeOH (10:1) as eluent. **M.p.**: 217-221 °C. **$^1$H NMR** (400 MHz, $CDCl_3$) δ 8.47 (s, 1H), 4.81 (s, 1H), 4.26 – 4.07 (m, 5H), 3.75 – 3.64 (m, 2H), 3.18 – 3.09 (m, 1H), 2.76 – 2.52 (m, 2H), 2.20 (s, 6H), 1.85 – 1.71 (m, 3H), 1.67 – 1.39 (m, 3H), 1.27 (td, *J* = 7.1, 1.0 Hz, 6H). **$^{13}$C NMR** (101 MHz, $CDCl_3$) δ 173.8, 167.91, 167.85, 148.2, 148.0, 98.6, 98.5, 59.9, 52.6, 52.4, 44.0, 41.9, 39.2, 32.0, 31.3, 25.5, 19.52, 19.50, 14.74, 14.69. **$^1$H NMR** (500 MHz, DMSO-$d_6$, 393K) δ 8.27 (s, 1H), 4.65 (s, 1H), 4.18 – 4.05 (m, 4H), 3.72 (s, 2H), 3.32 (s, 2H), 2.65 – 2.55 (m, 2H), 2.20 (s, 6H), 1.82 – 1.75 (m, 2H), 1.75 – 1.65 (m, 1H), 1.64 – 1.52 (m, 1H), 1.47 – 1.37 (m, 2H), 1.23 (t, *J* = 7.1 Hz, 6H). **$^{13}$C NMR** (126 MHz, DMSO-$d_6$, 393K) δ 171.8, 166.4, 145.9, 97.9, 58.4, 51.2, 41.8, 38.4, 30.9, 24.5, 17.6, 13.6. **Note:** At 393 K, the rotameric signals coalesce and the alkyl carbon adjacent to the amide nitrogen is broadened beyond detection. **HRMS** (ESI) calcd. for ($C_{21}H_{31}N_2O_5$) $[M+H]^+$: 391.2227, found 391.2222. **IR (neat)**: 3283, 3205, 3091, 2948, 2869, 1698, 1678, 1644, 1613, 1502, 1443, 1385, 1367, 1328, 1307, 1265, 1205, 1113, 1091, 1051, 1022.

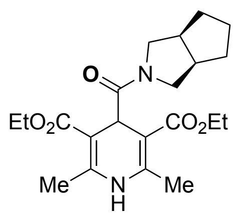

**2g**

**diethyl 4-((3aR,6aS)-5-(*tert*-butoxycarbonyl)octahydropyrrolo[3,4-*c*]pyrrole-2-carbonyl)-2,6-dimethyl-1,4-dihydropyridine-3,5-dicarboxylate (2h):** Following General Procedure B, 3,5-bis(ethoxycarbonyl)-2,6-dimethyl-1,4-dihydropyridine-4-carboxylic acid (2.00 g, 6.72 mmol), *tert*-butyl (3aR,6aS)-hexahydropyrrolo[3,4-*c*]pyrrole-2(1H)-carboxylate (2.14 g, 10.08 mmol), affording the title compound as a yellow solid (1.24 g, 38% yield) by using EtOAc/MeOH (10:1) as eluent. **M.p.**: 197-199 °C. **$^{1}$H NMR** (400 MHz, $CDCl_3$) δ 8.24 (s, 1H), 4.75 (s, 1H), 4.38 – 4.22 (m, 1H), 4.23 – 4.06 (m, 4H), 4.03 – 3.91 (m, 1H), 3.71 – 3.53 (m, 3H), 3.34 – 3.14 (m, 3H), 2.97 – 2.85 (m, 1H), 2.85 – 2.77 (m, 1H), 2.20 (d, *J* = 1.9 Hz, 6H), 1.44 (s, 9H), 1.29 – 1.25 (m, 6H). **$^{13}$C NMR** (101 MHz, $CDCl_3$) δ 174.5, 168.0, 167.6, 154.6, 148.0, 98.6, 79.5, 60.1, 60.0, 50.2, 50.0, 49.8, 49.5, 49.2, 43.0, 42.1, 40.9, 40.0, 39.6, 28.6, 19.7, 14.7. **$^{1}$H NMR** (500 MHz, DMSO-$d_6$, 393K) δ 8.29 (s, 1H), 4.62 (s, 1H), 4.17 – 4.07 (m, 4H), 3.95 – 3.25 (m, 6H), 3.15 – 3.09 (m, 2H), 2.84 (s, 2H), 2.21 (s, 6H), 1.42 (s, 9H), 1.22 (t, *J* = 7.1 Hz, 6H). **$^{13}$C NMR** (126 MHz, DMSO-$d_6$, 393K) δ 172.3, 166.3, 153.1, 146.1, 97.9, 77.8, 58.5, 49.21, 49.15, 38.6, 27.6, 17.7, 13.6. **Note:** At 393 K, the rotameric signals coalesce and the alkyl carbon adjacent to the amide nitrogen is broadened beyond detection. **HRMS** (ESI) calcd. for ($C_{25}H_{38}N_3O_7$) $[M+H]^+$: 492.2704, found 492.2712. **IR (neat)**: 3292, 3215, 3090, 2976, 2878, 1696, 1678, 1616, 1500, 1400, 1366, 1329, 1265, 1207, 1112, 1022, 964, 769.

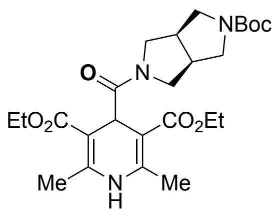


**2h**

**diethyl 2,6-dimethyl-4-(4-(pyrimidin-2-yl)piperazine-1-carbonyl)-1,4-dihydropyridine-3,5-dicarboxylate (2j):** Following General Procedure B, 3,5-bis(ethoxycarbonyl)-2,6-dimethyl-1,4-dihydropyridine-4-carboxylic acid (2.00 g, 6.72 mmol), 2-(piperazin-1-yl)pyrimidine (1.65 g, 10.08 mmol), affording the title compound as a white solid (410 mg, 14% yield) by using EtOAc/MeOH (10:1) as eluent. **M.p.**: 217-222 °C. **$^{1}$H NMR** (400 MHz, $CDCl_3$) δ 8.32 (d, *J* = 4.7 Hz, 2H), 7.49 (s, 1H), 6.51 (t, *J* = 4.7 Hz, 1H), 5.09 (s, 1H), 4.24 – 4.09 (m, 4H), 4.05 – 3.97 (m, 2H), 3.96 – 3.92 (m, 2H), 3.84 – 3.77 (m, 2H), 3.70 – 3.66 (m, 2H), 2.25 (s, 6H), 1.27 (t, *J* = 7.1 Hz, 6H). **$^{13}$C NMR** (101 MHz, $CDCl_3$) δ 174.7, 167.6, 161.7, 157.9, 147.7, 110.3, 99.1, 60.1, 46.6, 44.3, 43.6, 42.3, 36.8, 19.7, 14.8. **HRMS** (ESI) calcd. for ($C_{22}H_{30}N_5O_5$) $[M+H]^+$: 444.2241, found 444.2250. **IR (neat)**: 3277, 3188, 3085, 3074, 2980, 2948, 1701, 1675, 1640, 1617, 1500, 1461, 1370, 1326, 1297, 1261, 1205, 1119, 1094, 1053, 1025.

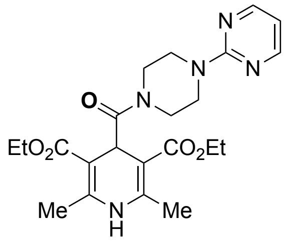


**2j**

**diethyl 2,6-dimethyl-4-(2-azaspiro[3.3]heptane-2-carbonyl)-1,4-dihydropyridine-3,5-dicarboxylate (2k):** Following General Procedure B, 3,5-bis(ethoxycarbonyl)-2,6-dimethyl-1,4-dihydropyridine-4-carboxylic acid (2.00 g, 6.72 mmol), 2-azaspiro[3.3]heptane hydrochloride (1.35 g,

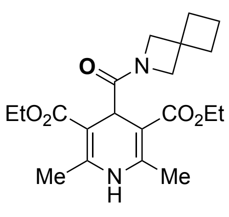

**2k**

10.08 mmol), affording the title compound as a yellow solid (2.11 g, 83% yield) by using EtOAc/MeOH (10:1) as eluent. **M.p.**: 227-229 °C. **$^1$H NMR** (400 MHz, $CDCl_3$) δ 8.74 (s, 1H), 4.53 (s, 1H), 4.51 (s, 2H), 4.23 – 4.11 (m, 4H), 3.89 (s, 2H), 2.22 – 2.11 (m, 10H), 1.89 – 1.79 (m, 2H), 1.28 (t, $J$ = 7.1 Hz, 6H).**$^{13}$C NMR** (101 MHz, $CDCl_3$) δ 174.5, 167.7, 148.4, 97.7, 63.3, 60.3, 59.9, 37.4, 36.9, 33.2, 19.3, 16.3, 14.8. **HRMS** (ESI) calcd. for ($C_{20}H_{29}N_2O_5$) $[M+H]^+$: 377.2071, found 377.2079. **IR (neat)**: 3283, 3195, 3077, 2980, 2953, 2874, 1701, 1676, 1640, 1618, 1502, 1460, 1370, 1327, 1296, 1261, 1204, 1119, 1094, 1053, 1026.

**diethyl 2,6-dimethyl-4-(7-oxa-2-azaspiro[3.5]nonane-2-carbonyl)-1,4-dihydropyridine-3,5-dicarboxylate (2l):** Following General Procedure B, 3,5-bis(ethoxycarbonyl)-2,6-dimethyl-1,4-dihydropyridine-4-carboxylic acid (1.21 g, 4.07 mmol) 7-oxa-2-azaspiro[3.5]nonane hydrochloride

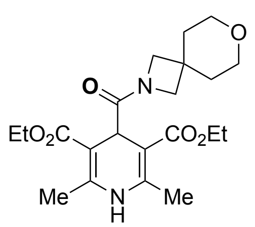

**2l**

(1.00 g, 6.11 mmol), affording the title compound as a white solid (1.48 g, 89% yield) by using EtOAc/MeOH (10:1) as eluent. **M.p.**: 256-260 °C. **$^1$H NMR** (400 MHz, $CDCl_3$) δ 8.73 (s, 1H), 4.56 (s, 1H), 4.32 (s, 2H), 4.27 – 4.07 (m, 4H), 3.70 – 3.54 (m, 6H), 2.16 (d, $J$ = 1.4 Hz, 6H), 1.83 – 1.72 (m, 4H), 1.30 – 1.26 (m, 6H). **$^{13}$C NMR** (101 MHz, $CDCl_3$) δ 175.0, 167.7, 148.3, 97.8, 65.0, 61.2, 59.9, 58.1, 37.1, 36.2, 32.3, 19.4, 14.8. **HRMS** (ESI) calcd. for ($C_{21}H_{31}N_2O_6$) $[M+H]^+$: 407.2177, found 407.2185. **IR (neat)**: 3271, 3203, 3070, 2978, 2963, 2940, 2842, 1695, 1662, 1500, 1461, 1384, 1368, 1295, 1269, 1208, 1195, 1110, 1092, 1050, 1023.

**diethyl 2,6-dimethyl-4-(7-oxa-2-azaspiro[3.5]nonane-2-carbonyl)-1,4-dihydropyridine-3,5-dicarboxylate (2m):** Following General Procedure B, 3,5-bis(ethoxycarbonyl)-2,6-dimethyl-1,4-dihydropyridine-4-carboxylic acid (2.00 g, 6.73 mmol), *tert*-butyl 2,7-diazaspiro[3.5]nonane-7-

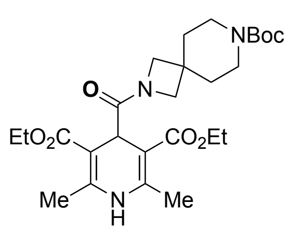

**2m**

carboxylate hydrochloride (2.65 g, 10.1 mmol), affording the title compound as a white solid (2.58 g, 76% yield) by using EtOAc/MeOH (10:1) as eluent. **M.p.**: 224-228 °C. **$^1$H NMR** (400 MHz, $CDCl_3$) δ 8.39 (s, 1H), 4.56 (s, 1H), 4.30 (s, 2H), 4.26 – 4.10 (m, 4H), 3.66 (s, 2H), 3.49 – 3.38 (m, 2H), 3.36 – 3.25 (m, 2H), 2.18 (s, 6H), 1.72 (t, $J$ = 5.6 Hz, 4H), 1.45 (s, 9H), 1.29 (t, $J$ = 7.1 Hz, 6H). **$^{13}$C NMR** (101 MHz, $CDCl_3$) δ 175.0, 167.7, 154.9, 148.1, 97.9, 79.8, 60.7, 60.0, 57.5, 37.0, 35.3, 33.2, 28.6, 19.5, 14.8. **HRMS** (ESI) calcd. for ($C_{26}H_{40}N_3O_7$) $[M+H]^+$: 506.2861, found 506.2852. **IR (neat)**: 3287, 3215, 3087, 2977, 2932, 1696, 1625, 1500, 1459, 1423, 1366, 1270, 1241, 1209, 1169, 1113, 1052, 1022, 763.

**diethyl 2,6-dimethyl-4-(1,4-dioxa-8-azaspiro[4.5]decane-8-carbonyl)-1,4-dihydropyridine-3,5-dicarboxylate (2n):** Following General Procedure B, 3,5-bis(ethoxycarbonyl)-2,6-dimethyl-1,4-mmol), affording the title compound as a white solid (366 mg, 16% yield) by using EtOAc/MeOH

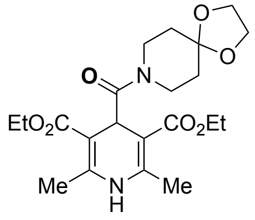


**2n**

(10:1) as eluent. **M.p.**: 225-231 °C. **$^1$H NMR** (400 MHz, $CDCl_3$) δ 7.32 (s, 1H), 5.12 (s, 1H), 4.24 – 4.08 (m, 4H), 4.00 – 3.92 (m, 6H), 3.67 (t, *J* = 5.9 Hz, 2H), 2.24 (s, 6H), 1.83 (t, *J* = 5.7 Hz, 2H), 1.65 (t, *J* = 5.6 Hz, 2H), 1.27 (t, *J* = 7.1 Hz, 6H). **$^{13}$C NMR** (101 MHz, $CDCl_3$) δ 174.2, 167.6, 147.5, 107.4, 99.2, 64.5, 60.0, 44.9, 40.6, 36.5, 35.9, 34.9, 19.8, 14.7. **HRMS** (ESI) calcd. for ($C_{21}H_{31}N_2O_7$) $[M+H]^+$: 423.2126, found 423.2133. **IR (neat)**: 3285, 3215, 3093, 2977, 2892, 1698, 1676, 1612, 1500, 1446, 1367, 1327, 1306, 1263, 1204, 1101, 1022.

## 4. Ni-Catalyzed Enantioselective Carbamoylation of Aziridines

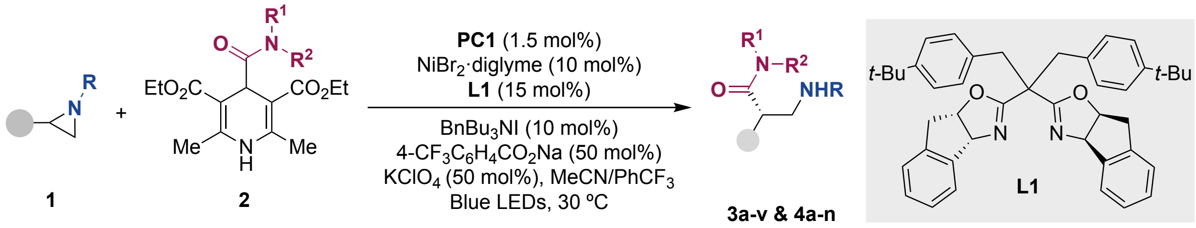


**General Procedure C**: In a 7 mL vial equipped with a magnetic stir bar, 5-TCzBN (3.3 mg), $NiBr_2$·diglyme (5.3 mg, 10 mol%), **L1** (14.0 mg, 15 mol%), $BnBu_3NI$ (6.1 mg, 10 mol%), 4-$CF_3C_6H_4CO_2Na$ (15.9 mg, 0.075 mmol, 0.5 equiv), and $KClO_4$ (10.4 mg, 0.075 mmol, 0.5 equiv), 4-carbamoyl 1,4-dihydropyridine (0.30 mmol, 2.0 equiv), aziridine (0.15 mmol, 1.0 equiv) were added. The vial was sealed with an aluminium crimp, evacuated, and backfilled with argon at least three times. Subsequently, anhydrous α,α,α-trifluorotoluene (4.2 mL) and $CH_3CN$ (1.05 mL) were added via syringe to the flask containing the Ni/L manifold under an argon atmosphere. The reaction mixture was stirred at 850 rpm and irradiated at 451 nm in a blue LED photoreactor at 30 °C for 16 hours. The mixture was then filtered through a short column of silica gel using ethyl acetate as the eluent. The filtrate was concentrated and subsequently purified by column chromatography on silica gel (using $^i$PrOAc/acetone as eluent). The enantiomeric ratio was determined through SFC analysis on a chiral stationary phase.

**(*S*)-4-methyl-*N*-(3-oxo-3-(pyrrolidin-1-yl)-2-(*p*-tolyl)propyl)benzenesulfonamide (3a):** Following General Procedure C, 2-(*p*-tolyl)-1-tosylaziridine (**1a**) (43.1 mg, 0.15 mmol), and diethyl 2,6-dimethyl-4-(pyrrolidine-1-carbonyl)-1,4-dihydropyridine-3,5-dicarboxylate (**2a**) (105.1 mg, 0.30 mmol) were

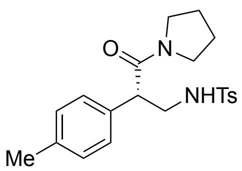


**3a**

used, affording the title compound as a white solid (41.6 mg, 72% yield) by using $^{i}$PrOAc/Acetone (20:1) as eluent. In an independent experiment, 43.2 mg (75% yield) were obtained, giving an average yield of 73% with 96:4 er. **M.p.**: 106-110 °C. $^{1}$**H NMR** (400 MHz, $CDCl_3$) δ 7.73 – 7.67 (m, 2H), 7.28 – 7.23 (m, 2H), 7.12 – 7.04 (m, 4H), 5.40 (dd, $J$ = 9.1, 4.6 Hz, 1H), 3.83 (dd, $J$ = 9.8, 4.8 Hz, 1H), 3.52 – 3.45 (m, 1H), 3.42 – 3.24 (m, 3H), 3.23 – 3.12 (m, 1H), 2.95 – 2.86 (m, 1H), 2.39 (s, 3H), 2.30 (s, 3H), 1.89 – 1.64 (m, 4H). $^{13}$**C NMR (101 MHz, $CDCl_3$)** δ 170.1, 143.3, 137.6, 137.5, 133.1, 129.84, 129.78, 128.1, 127.1, 51.5, 47.3, 46.2, 46.1, 26.0, 24.2, 21.6, 21.2. **HRMS** (ESI) calcd. for ($C_{21}H_{27}N_2O_3S$) $[M+H]^+$: 387.1737, found 387.1735. **IR** (neat): 3200, 2972, 2952, 2926, 2876, 1623, 1513, 1449, 1329, 1158, 1093, 816, 754, 662, 550. **Optical rotation**: $[\alpha]^{26}_{D}$ = 13.4 (c 0.3, $CHCl_3$, 96:4 er). The enantiomeric excess of **3a** was determined by SFC analysis on a Chiralpak ID-3 column ($CO_2$/MeOH with a gradient from 100% to 70% $CO_2$ in 5 min, then maintained at 70% $CO_2$, column temperature 35 °C, flow rate 1.2 mL/min) with retention time 6.57 min (major) and 8.69 min (minor).

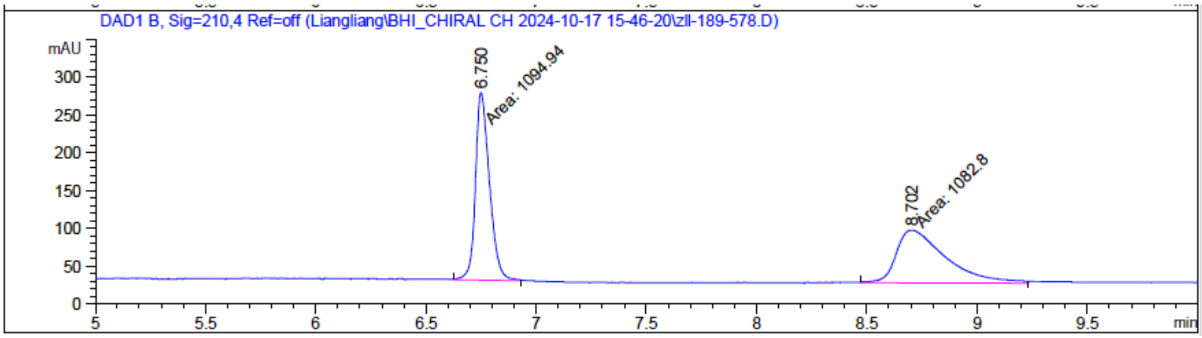


Signal 2: DAD1 B, Sig=210,4 Ref=off

| Peak # | RetTime [min] | Type | Width [min] | Area [mAU*s] | Height [mAU] | Area % |
|---|---|---|---|---|---|---|
| 1 | 6.750 | MM | 0.0736 | 1094.93738 | 248.06291 | 50.2786 |
| 2 | 8.702 | MF | 0.2587 | 1082.80481 | 69.75238 | 49.7214 |

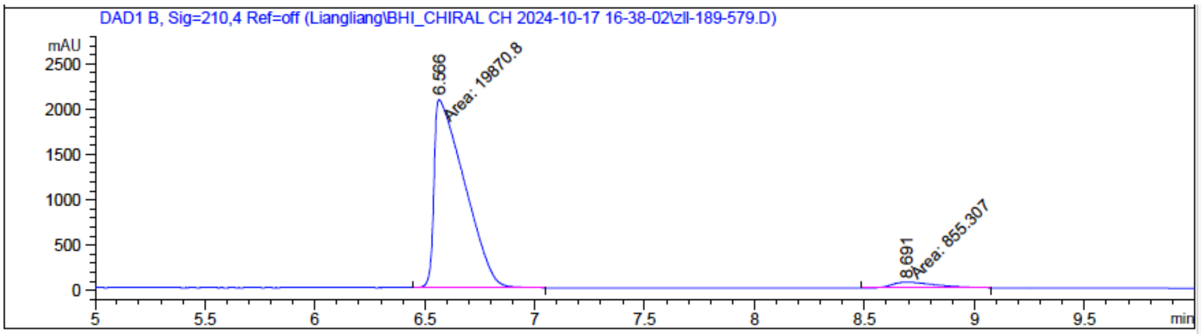

Signal 2: DAD1 B, Sig=210,4 Ref=off

| Peak # | RetTime [min] | Type | Width [min] | Area [mAU*s] | Height [mAU] | Area % |
|---|---|---|---|---|---|---|
| 1 | 6.566 | MM | 0.1599 | 1.98708e4 | 2071.20239 | 95.8733 |
| 2 | 8.691 | MM | 0.2289 | 855.30750 | 62.28825 | 4.1267 |

**(*S*)-4-methyl-*N*-(3-oxo-2-phenyl-3-(pyrrolidin-1-yl)propyl)benzenesulfonamide (3b):** Following General Procedure C, 2-phenyl-1-tosylaziridine (**1b**) (41.0 mg, 0.15 mmol), and diethyl 2,6-dimethyl-4-(pyrrolidine-1-carbonyl)-1,4-dihydropyridine-3,5-dicarboxylate (**2a**) (78.8 mg, 0.225 mmol) were used, affording the title compound as a white solid (32.9 mg, 59% yield) by using $^{i}$PrOAc/Acetone (20:1) as eluent. In an independent experiment, 34.5 mg (62% yield) were obtained, giving an average yield of 60% with 95:5 er. **M.p.**: 53-58 °C. **$^{1}$H NMR** (400 MHz, $CDCl_3$) δ 7.72 – 7.69 (m, 2H), 7.34 – 7.21 (m, 5H), 7.21 – 7.17 (m, 2H), 5.33 (dd, $J$ = 9.2, 4.6 Hz, 1H), 3.88 (dd, $J$ = 9.8, 4.8 Hz, 1H), 3.56 – 3.46 (m, 1H), 3.43 – 3.26 (m, 3H), 3.26 – 3.15 (m, 1H), 2.92 – 2.86 (m, 1H), 2.40 (s, 3H), 1.88 – 1.68 (m, 4H). **$^{13}$C NMR** (101 MHz, $CDCl_3$) δ 169.9, 143.3, 137.5, 136.2, 129.8, 129.2, 128.2, 127.8, 127.0, 51.9, 47.20, 46.17, 46.16, 26.0, 24.1, 21.6. **HRMS** (ESI) calcd. for ($C_{20}H_{25}N_2O_3S$) $[M+H]^+$: 373.1580, found 373.1579. **IR** (neat): 3177, 2970, 2875, 1623, 1442, 1324, 1157, 1093, 816, 753, 702, 660, 551. **Optical rotation**: $[\alpha]^{26}_{D}$ = 9.6 (c 0.3, $CHCl_3$, 95:5 er). The enantiomeric excess of **3b** was determined by SFC analysis on a Chiralpak ID-3 column ($CO_2$/MeOH with a gradient from 100% to 70% $CO_2$ in 5 min, then maintained at 70% $CO_2$, column temperature 35 °C, flow rate 1.2 mL/min) with retention time 6.32 min (major) and 8.24 min (minor).

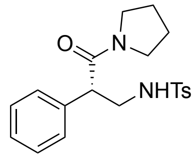

**3b**

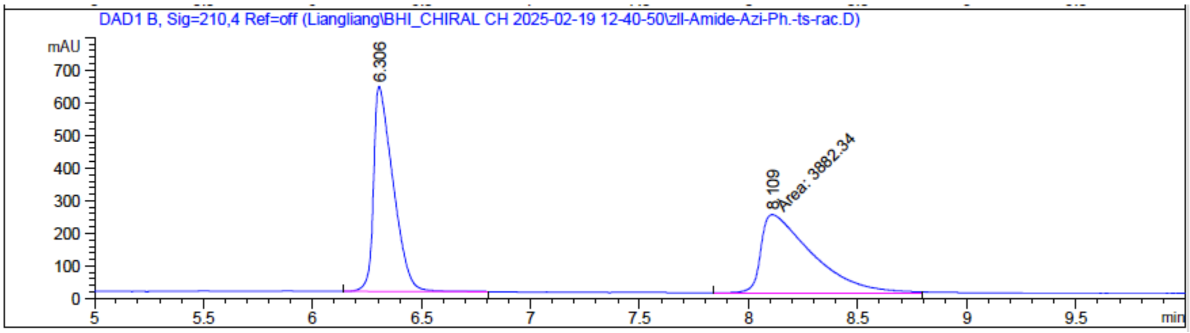


Signal 2: DAD1 B, Sig=210,4 Ref=off

| Peak # | RetTime [min] | Type | Width [min] | Area [mAU*s] | Height [mAU] | Area % |
|---|---|---|---|---|---|---|
| 1 | 6.306 | VV R | 0.0909 | 3925.38721 | 628.19049 | 50.2757 |
| 2 | 8.109 | MF | 0.2705 | 3882.34229 | 239.24518 | 49.7243 |

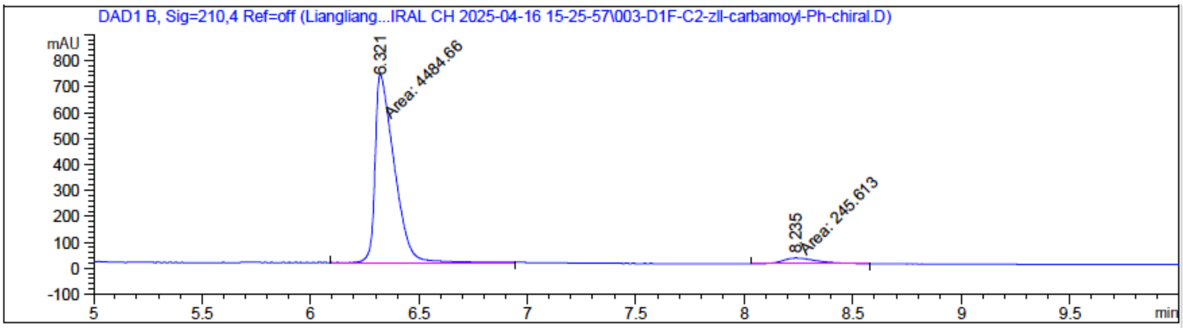


Signal 2: DAD1 B, Sig=210,4 Ref=off

| Peak # | RetTime [min] | Type | Width [min] | Area [mAU*s] | Height [mAU] | Area % |
|---|---|---|---|---|---|---|
| 1 | 6.321 | MM | 0.1031 | 4484.65576 | 725.12262 | 94.8076 |
| 2 | 8.235 | MM | 0.1857 | 245.61334 | 22.04919 | 5.1924 |

**(*S*)-*N*-(2-(4-(*tert*-butyl)phenyl)-3-oxo-3-(pyrrolidin-1-yl)propyl)-4-methylbenzenesulfonamide (3c):** Following General Procedure C, 2-(4-(*tert*-butyl)phenyl)-1-tosylaziridine (**1c**) (49.4 mg, 0.15 mmol), and diethyl 2,6-dimethyl-4-(pyrrolidine-1-carbonyl)-1,4-dihydropyridine-3,5-dicarboxylate (**2a**) (78.8 mg, 0.225 mmol) were used, affording the title compound as a yellow oil (37.1 mg, 58% yield) by using $^{i}$PrOAc/Acetone (20:1) as eluent. In an independent experiment, 39.7 mg (62% yield) were obtained, giving an average yield of 60% with 95:5 er. **$^{1}$H NMR** (400 MHz, $CDCl_3$) δ 7.72 – 7.69 (m, 2H), 7.33 – 7.23 (m, 4H), 7.12 – 7.09 (m, 2H), 5.35 (dd, *J* = 9.2, 4.6 Hz, 1H), 3.85 (dd, *J* = 9.9, 4.8 Hz, 1H), 3.56 – 3.45 (m, 1H), 3.42 – 3.27 (m, 3H), 3.24 – 3.12 (m, 1H), 2.98 – 2.88 (m, 1H), 2.39 (s, 3H), 1.90 – 1.70 (m, 4H), 1.28 (s, 9H). **$^{13}$C NMR** (101 MHz, $CDCl_3$) δ 170.1, 150.7, 143.3, 137.5, 133.0, 129.8, 127.8, 127.1, 126.1, 51.4, 47.2, 46.2, 46.1, 34.6, 31.4, 26.0, 24.2, 21.6. **HRMS** (ESI) calcd. for ($C_{24}H_{33}N_2O_3S$) $[M+H]^+$: 429.2206, found 429.2208. **IR** (neat): 3178, 2962, 2873, 1624, 1450, 1330, 1159, 1094, 837, 816, 754, 663, 565, 552. **Optical rotation**: $[\alpha]^{26}_{D}$ = 17.8 (c 0.8, $CHCl_3$, 95:5 er). The enantiomeric excess of **3c** was determined by SFC analysis on a Chiralpak ID-3 column ($CO_2$/MeOH with a gradient from 100% to 70% $CO_2$ in 5 min, then maintained at 70% $CO_2$, column temperature 35 °C, flow rate 1.2 mL/min) with retention time 6.55 min (major) and 7.65 min (minor).

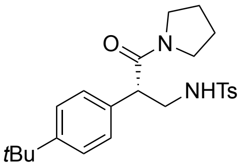

**3c**

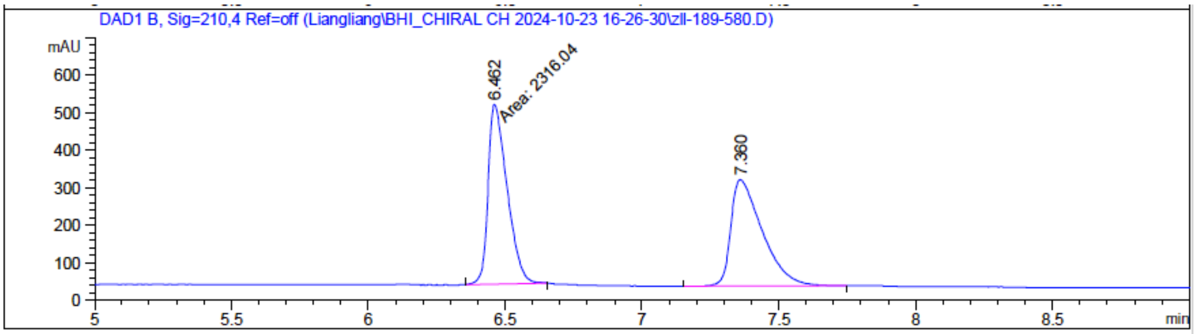

Signal 2: DAD1 B, Sig=210,4 Ref=off

| Peak # | RetTime [min] | Type | Width [min] | Area [mAU*s] | Height [mAU] | Area % |
|---|---|---|---|---|---|---|
| 1 | 6.462 | MM | 0.0804 | 2316.03662 | 480.01010 | 50.4742 |
| 2 | 7.360 | VV R | 0.1199 | 2272.52075 | 283.82935 | 49.5258 |

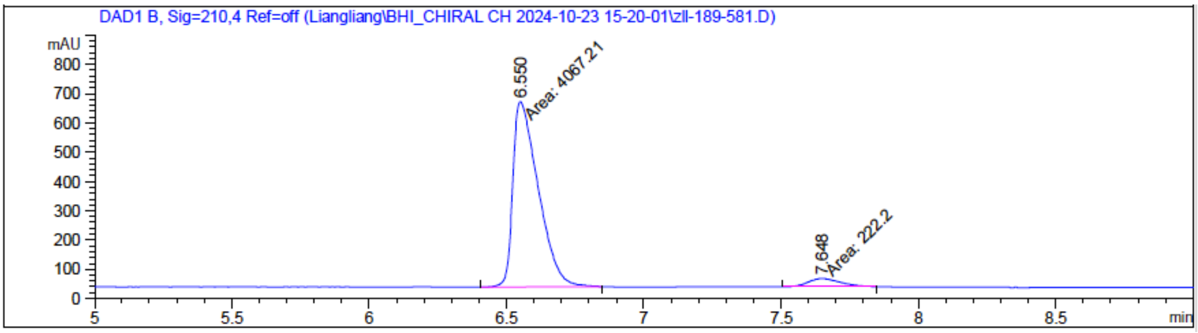


Signal 2: DAD1 B, Sig=210,4 Ref=off

| Peak # | RetTime [min] | Type | Width [min] | Area [mAU*s] | Height [mAU] | Area % |
|---|---|---|---|---|---|---|
| 1 | 6.550 | MM | 0.1070 | 4067.20581 | 633.47833 | 94.8198 |
| 2 | 7.648 | MM | 0.1350 | 222.19994 | 27.42247 | 5.1802 |

**(*S*)-*N*-(2-(4-isobutylphenyl)-3-oxo-3-(pyrrolidin-1-yl)propyl)-4-methylbenzenesulfonamide (3d):** Following General Procedure C, 2-(4-isobutylphenyl)-1-tosylaziridine (**1d**) (49.4 mg, 0.15 mmol), and diethyl 2,6-dimethyl-4-(pyrrolidine-1-carbonyl)-1,4-dihydropyridine-3,5-dicarboxylate (**2a**) (105.1mg, 0.30 mmol) were used, affording the title compound as a yellow oil (35.6 mg, 55% yield) by using $^{i}$PrOAc/Acetone (20:1) as eluent. In an independent experiment, 33.0 mg (51% yield) were obtained, giving an average yield of 53% with 95:5 er.

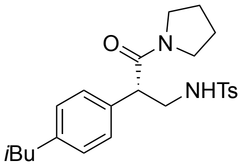


**3d**

$^{1}$**H NMR** (400 MHz, $CDCl_3$) δ 7.75 – 7.68 (m, 2H), 7.26 (d, $J$ = 8.1 Hz, 2H), 7.11 – 7.02 (m, 4H), 5.42 (dd, $J$ = 9.2, 4.5 Hz, 1H), 3.84 (dd, $J$ = 9.9, 4.7 Hz, 1H), 3.55 – 3.44 (m, 1H), 3.44 – 3.23 (m, 3H), 3.24 – 3.13 (m, 1H), 2.95 – 2.85 (m, 1H), 2.41 (d, $J$ = 7.2 Hz, 2H), 2.39 (s, 3H), 1.87 – 1.67 (m, 5H), 0.87 (d, $J$ = 6.6 Hz, 6H). $^{13}$**C NMR** (101 MHz, $CDCl_3$) δ 170.1, 143.3, 141.3, 137.6, 133.3, 129.9, 129.8, 127.9, 127.1, 51.5, 47.2, 46.2, 46.1, 45.1, 30.2, 26.0, 24.1, 22.5, 21.6. **HRMS** (ESI) calcd. for ($C_{24}H_{33}N_2O_3S$) $[M+H]^+$: 429.2206, found 429.2212. **IR** (neat): 3189, 2953, 2925, 2870, 1624, 1450, 1331, 1159, 1094, 815, 659, 552. **Optical rotation**: $[\alpha]^{26}_{D}$ = 14.3 (c 0.3, $CHCl_3$, 95:5 er). The enantiomeric excess of **3d** was determined by SFC analysis on a Chiralpak ID-3 column ($CO_2$/MeOH with a gradient from 100% to 70% $CO_2$ in 5 min, then maintained at 70% $CO_2$, column temperature 35 °C, flow rate 1.2 mL/min) with retention time 6.37 min (major) and 7.71 min (minor).

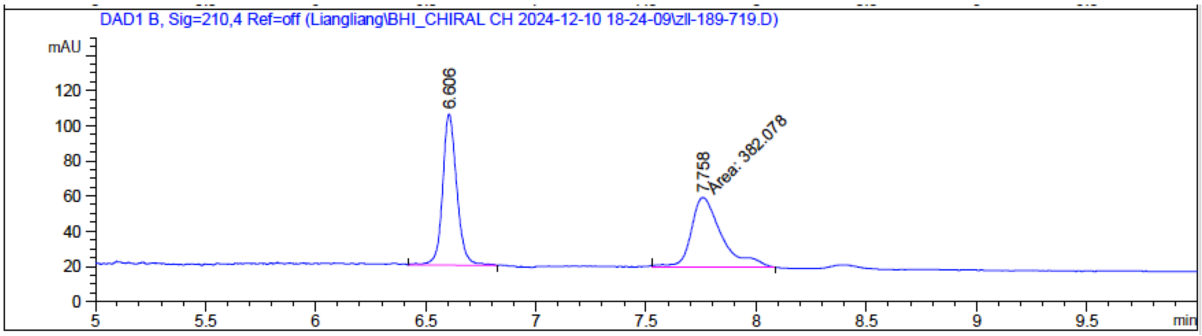


Signal 2: DAD1 B, Sig=210,4 Ref=off

| Peak # | RetTime [min] | Type | Width [min] | Area [mAU*s] | Height [mAU] | Area % |
|---|---|---|---|---|---|---|
| 1 | 6.606 | VV R | 0.0691 | 384.68478 | 86.14333 | 50.1700 |
| 2 | 7.758 | MM | 0.1610 | 382.07776 | 39.55965 | 49.8300 |

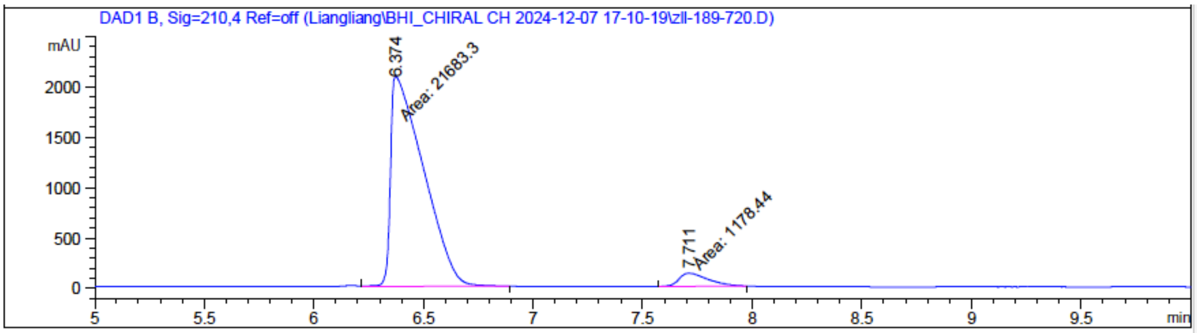


Signal 2: DAD1 B, Sig=210,4 Ref=off

| Peak # | RetTime [min] | Type | Width [min] | Area [mAU*s] | Height [mAU] | Area % |
|---|---|---|---|---|---|---|
| 1 | 6.374 | MM | 0.1736 | 2.16833e4 | 2082.21265 | 94.8453 |
| 2 | 7.711 | MM | 0.1521 | 1178.44373 | 129.13676 | 5.1547 |

**(*S*)-4-methyl-*N*-(3-oxo-3-(pyrrolidin-1-yl)-2-(*m*-tolyl)propyl)benzenesulfonamide (3e):** Following General Procedure C, 2-(*m*-tolyl)-1-tosylaziridine (**1e**) (43.1 mg, 0.15 mmol), and diethyl 2,6-dimethyl-4-(pyrrolidine-1-carbonyl)-1,4-dihydropyridine-3,5-dicarboxylate (**2a**) (105.1 mg, 0.3 mmol) were used, affording the title compound as a yellow oil (37.1 mg, 64% yield) by using $^{i}$PrOAc/Acetone (20:1) as eluent. In an independent experiment, 38.9 mg (67% yield) were obtained, giving an average yield of 66% with 93.5:6.5 er. **$^{1}$H NMR** (400 MHz, $CDCl_3$) δ 7.74 – 7.67 (m, 2H), 7.26 (d, $J$ = 8.0 Hz, 2H), 7.17 (t, $J$ = 7.5 Hz, 1H), 7.05 (d, $J$ = 7.6 Hz, 1H), 7.02 – 6.95 (m, 2H), 5.42 (dd, $J$ = 9.2, 4.4 Hz, 1H), 3.83 (dd, $J$ = 9.8, 4.7 Hz, 1H), 3.56 – 3.45 (m, 1H), 3.42 – 3.25 (m, 3H), 3.24 – 3.13 (m, 1H), 2.97 – 2.88 (m, 1H), 2.39 (s, 3H), 2.29 (s, 3H), 1.87 – 1.70 (m, 4H). **$^{13}$C NMR** (101 MHz, $CDCl_3$) δ 170.0, 143.3, 139.0, 137.5, 136.1, 129.8, 129.0, 128.8, 128.6, 127.0, 125.3, 51.9, 47.3, 46.18, 46.16, 26.0, 24.2, 21.6, 21.5. **HRMS** (ESI) calcd. for ($C_{21}H_{27}N_2O_3S$) $[M+H]^+$: 387.1737, found 387.1739. **IR** (neat): 3179, 2953, 2924, 2875, 1624, 1449, 1329, 1159, 1093, 816, 758, 702, 660, 551. **Optical**

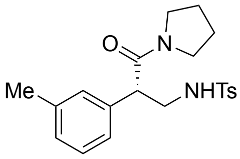


**3e**

**rotation**: $[\alpha]^{26}_{D}$ = 10.2 (c 0.6, $CHCl_3$, 93.5:6.5 er). The enantiomeric excess of **3e** was determined by SFC analysis on a Chiralpak ID-3 column ($CO_2$/MeOH with a gradient from 100% to 70% $CO_2$ in 5 min, then maintained at 70% $CO_2$, column temperature 35 °C, flow rate 1.2 mL/min) with retention time 6.67 min (major) and 7.92 min (minor).

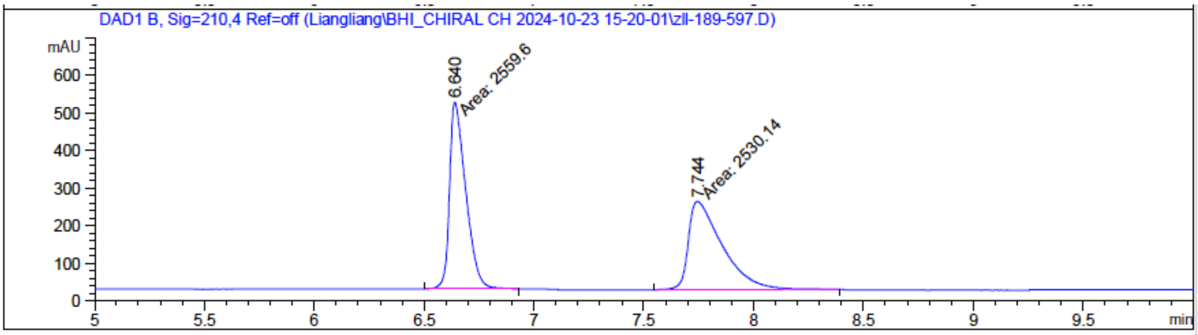


Signal 2: DAD1 B, Sig=210,4 Ref=off

| Peak # | RetTime [min] | Type | Width [min] | Area [mAU*s] | Height [mAU] | Area % |
|---|---|---|---|---|---|---|
| 1 | 6.640 | MM | 0.0858 | 2559.60278 | 496.93814 | 50.2894 |
| 2 | 7.744 | MM | 0.1794 | 2530.14038 | 235.06894 | 49.7106 |

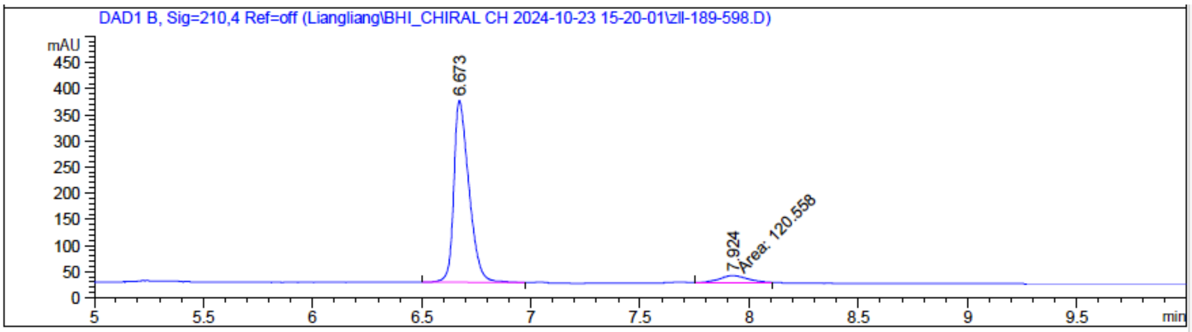


Signal 2: DAD1 B, Sig=210,4 Ref=off

| Peak # | RetTime [min] | Type | Width [min] | Area [mAU*s] | Height [mAU] | Area % |
|---|---|---|---|---|---|---|
| 1 | 6.673 | VV R | 0.0729 | 1682.45496 | 347.90906 | 93.3135 |
| 2 | 7.924 | MM T | 0.1509 | 120.55756 | 13.31358 | 6.6865 |

**(*S*)-4-methyl-*N*-(3-oxo-3-(pyrrolidin-1-yl)-2-(*o*-tolyl)propyl)benzenesulfonamide (3f):** Following General Procedure C, 2-(*o*-tolyl)-1-tosylaziridine (**1f**) (43.1 mg, 0.15 mmol), and diethyl 2,6-dimethyl-4-(pyrrolidine-1-carbonyl)-1,4-dihydropyridine-3,5-dicarboxylate (**2a**) (105.1mg, 0.30 mmol) were used, affording the title compound as a white solid (35.4 mg, 61% yield) by using $^{i}$PrOAc/Acetone (20:1) as eluent. In an independent experiment, 36.4 mg (63% yield) were obtained, giving an average yield of 62% with 92:8 er. **M.p.**: 57-59 °C. **$^{1}$H NMR** (400 MHz, $CDCl_3$) δ 7.74 – 7.70 (m, 2H), 7.26 (d, *J* = 8.1 Hz, 2H), 7.18 – 7.06 (m, 3H), 7.05 – 7.01 (m, 1H), 5.53 (dd, *J* = 9.6, 4.1 Hz, 1H), 4.01 (dd, *J* = 10.2, 4.0 Hz, 1H), 3.54 – 3.47 (m, 1H), 3.44 – 3.37 (m, 1H), 3.30 – 3.23 (m, 1H), 3.17 – 3.08 (m, 2H), 2.68 –

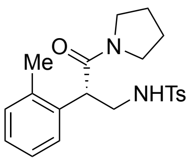


**3f**

2.61 (m, 1H), 2.40 (d, $J$ = 2.4 Hz, 6H), 1.84 – 1.64 (m, 4H). **$^{13}$C NMR** (101 MHz, $CDCl_3$) δ 170.6, 143.3, 137.9, 136.1, 134.6, 130.9, 129.8, 127.8, 127.01, 126.98, 48.5, 46.2, 45.8, 45.5, 25.9, 24.2, 21.6, 19.5. **HRMS** (ESI) calcd. for ($C_{21}H_{27}N_2O_3S$) $[M+H]^+$: 387.1737, found 387.1734. **IR** (neat): 3190, 2971, 2952, 2874, 1624, 1446, 1328, 1157, 1093, 816, 755, 659, 544. **Optical rotation**: $[\alpha]^{26}_D$ = 3.9 (c 0.3, $CHCl_3$, 92:8 er). The enantiomeric excess of **3f** was determined by SFC analysis on a Chiralpak ID-3 column ($CO_2$/MeOH with a gradient from 100% to 70% $CO_2$ in 5 min, then maintained at 70% $CO_2$, column temperature 35 °C, flow rate 1.2 mL/min) with retention time 6.89 min (major) and 8.11 min (minor).

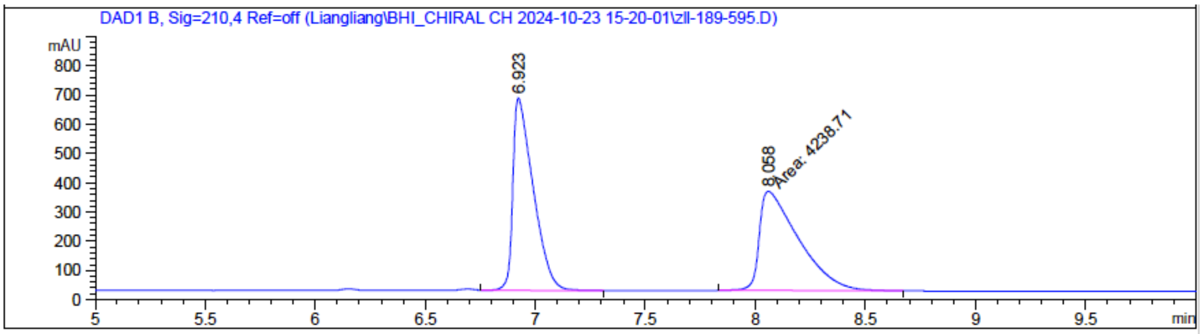


Signal 2: DAD1 B, Sig=210,4 Ref=off

| Peak # | RetTime [min] | Type | Width [min] | Area [mAU*s] | Height [mAU] | Area % |
|---|---|---|---|---|---|---|
| 1 | 6.923 | VV R | 0.0932 | 4238.41748 | 657.82062 | 49.9983 |
| 2 | 8.058 | MM | 0.2081 | 4238.71338 | 339.42316 | 50.0017 |

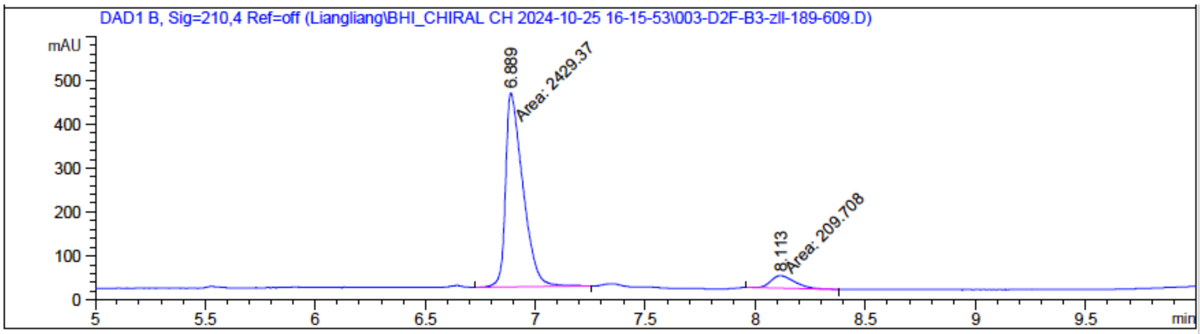


Signal 2: DAD1 B, Sig=210,4 Ref=off

| Peak # | RetTime [min] | Type | Width [min] | Area [mAU*s] | Height [mAU] | Area % |
|---|---|---|---|---|---|---|
| 1 | 6.889 | MM | 0.0915 | 2429.37158 | 442.45993 | 92.0538 |
| 2 | 8.113 | MM | 0.1248 | 209.70781 | 28.00015 | 7.9462 |

**(*S*)-*N*-(2-(4-fluorophenyl)-3-oxo-3-(pyrrolidin-1-yl)propyl)-4-methylbenzenesulfonamide (3g):** Following General Procedure C, 2-(4-fluorophenyl)-1-tosylaziridine (**1g**) (43.7 mg, 0.15 mmol), and diethyl 2,6-dimethyl-4-(pyrrolidine-1-carbonyl)-1,4-dihydropyridine-3,5-dicarboxylate (**2a**) (105.1mg, 0.30 mmol) were used, affording the title compound as a white solid (42.0 mg, 72% yield) by using $^{i}$PrOAc/Acetone (20:1) as eluent. In an independent experiment, 43.6 mg (75% yield) were obtained, giving an average yield of 73% with 95.5:4.5 er. **M.p.**: 143-145 °C. **$^{1}$H NMR** (300 MHz, $CDCl_3$) δ 7.72 – 7.68 (m, 2H), 7.32 – 7.22 (m, 2H), 7.21 – 7.14 (m, 2H), 7.02 – 6.94 (m, 2H), 5.36 (dd, *J* = 8.9, 4.8 Hz, 1H), 3.88 (dd, *J* = 9.6, 4.8 Hz, 1H), 3.55 – 3.46 (m, 1H), 3.43 – 3.27 (m, 3H), 3.25 – 3.10 (m, 1H), 2.97 – 2.83 (m, 1H), 2.40 (s, 3H), 1.89 – 1.71 (m, 4H). **$^{13}$C NMR** (101 MHz, $CDCl_3$) δ 169.8, 162.4 (d, *J* = 246.8 Hz), 143.4, 137.5, 132.0 (d, *J* = 3.3 Hz), 129.86 (d, *J* = 8.1 Hz), 129.85, 127.0, 116.1 (d, *J* = 21.5 Hz), 51.1, 47.2, 46.2, 26.0, 24.2, 21.6. **$^{19}$F NMR** (282 MHz, $CDCl_3$) δ -114.27. **HRMS** (ESI) calcd. for ($C_{20}H_{24}FN_2O_3S$) $[M+H]^+$: 391.1486, found 391.1484. **IR** (neat): 3183, 2972, 2926, 2876, 1623, 1508, 1449, 1327, 1224, 1158, 1093, 842, 815, 754, 661, 550. **Optical rotation**: $[\alpha]^{26}_{D}$ = 6.8 (c 0.3, $CHCl_3$, 95.5:4.5 er). The enantiomeric excess of **3g** was determined by SFC analysis on a Chiralpak ID-3 column ($CO_2$/MeOH with a gradient from 100% to 70% $CO_2$ in 5 min, then maintained at 70% $CO_2$, column temperature 35 °C, flow rate 1.2 mL/min) with retention time 6.06 min (major) and 7.79 min (minor).

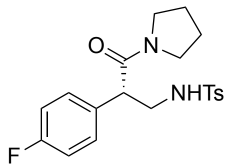

3g

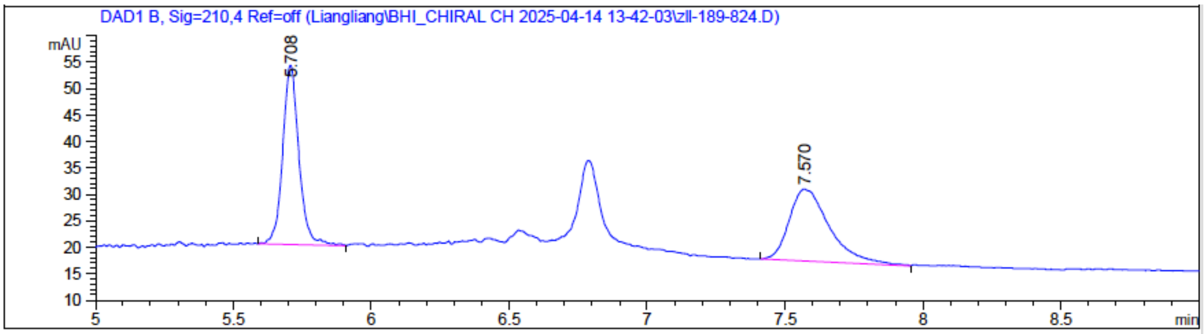


Signal 2: DAD1 B, Sig=210,4 Ref=off

| Peak # | RetTime [min] | Type | Width [min] | Area [mAU*s] | Height [mAU] | Area % |
|---|---|---|---|---|---|---|
| 1 | 5.708 | VV R | 0.0616 | 138.31348 | 33.90717 | 50.0437 |
| 2 | 7.570 | BB | 0.1368 | 138.07187 | 13.61445 | 49.9563 |

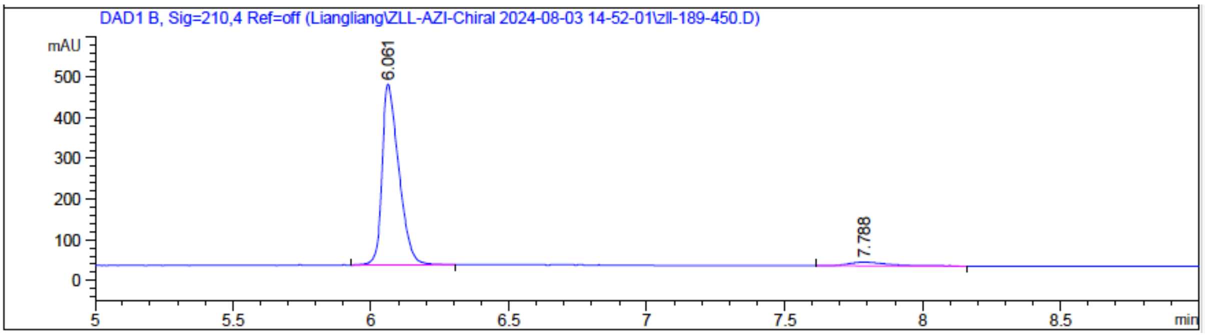

Signal 2: DAD1 B, Sig=210,4 Ref=off

| Peak # | RetTime [min] | Type | Width [min] | Area [mAU*s] | Height [mAU] | Area % |
|---|---|---|---|---|---|---|
| 1 | 6.061 | BV R | 0.0627 | 1874.06140 | 444.79584 | 95.3238 |
| 2 | 7.788 | BB | 0.1328 | 91.93470 | 9.10307 | 4.6762 |

**(*S*)-*N*-(2-(4-(*tert*-butoxy)phenyl)-3-oxo-3-(pyrrolidin-1-yl)propyl)-4-methylbenzenesulfonamide (3h):** Following General Procedure C, 2-(4-(*tert*-butoxy)phenyl)-1-tosylaziridine (**1h**) (51.8 mg, 0.15 mmol), and diethyl 2,6-dimethyl-4-(pyrrolidine-1-carbonyl)-1,4-dihydropyridine-3,5-dicarboxylate

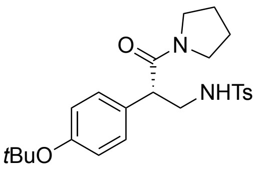

**3h**

(**2a**) (105.1mg, 0.30 mmol) were used, affording the title compound as a yellow oil (45.3 mg, 68% yield) by using $^i$PrOAc/Acetone (20:1) as eluent. In an independent experiment, 47.7 mg (72% yield) were obtained, giving an average yield of 70% with 95.5:4.5 er. **$^1$H NMR** (400 MHz, $CDCl_3$) δ 7.73 – 7.70 (m, 2H), 7.26 (d, *J* = 8.0 Hz, 2H), 7.08 – 7.04 (m, 2H), 6.90 – 6.87 (m, 2H), 5.45 (dd, *J* = 8.9, 4.3 Hz, 1H), 3.82 (dd, *J* = 9.8, 4.7 Hz, 1H), 3.54 – 3.43 (m, 1H), 3.43 – 3.21 (m, 3H), 3.23 – 3.12 (m, 1H), 2.94 – 2.84 (m, 1H), 2.39 (s, 3H), 1.86 – 1.67 (m, 4H), 1.31 (s, 9H). **$^{13}$C NMR** (101 MHz, $CDCl_3$) δ 155.1, 143.3, 137.6, 130.7, 129.8, 128.7, 127.0, 124.5, 78.7, 51.2, 47.2, 46.2, 46.1, 29.0, 26.0, 24.2, 21.6. **HRMS** (ESI) calcd. for ($C_{24}H_{33}N_2O_4S$) $[M+H]^+$: 445.2156, found 445.2148. **IR** (neat): 3177, 2976, 2876, 1623, 1505, 1449, 1330, 1238, 1159, 1094, 897, 855, 816, 753, 660, 552. **Optical rotation**: $[\alpha]^{26}_{D}$ = 13.4 (c 0.3, $CHCl_3$, 95.5:4.5 er). The enantiomeric excess of **3h** was determined by SFC analysis on a Chiralpak ID-3 column ($CO_2$/MeOH with a gradient from 100% to 70% $CO_2$ in 5 min, then maintained at 70% $CO_2$, column temperature 35 °C, flow rate 1.2 mL/min) with retention time 6.78 min (major) and 8.72 min (minor).

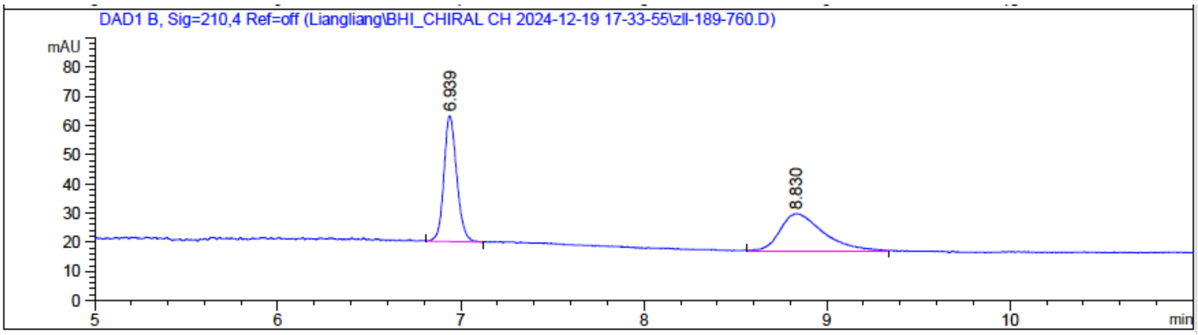


Signal 2: DAD1 B, Sig=210,4 Ref=off

| Peak # | RetTime [min] | Type | Width [min] | Area [mAU*s] | Height [mAU] | Area % |
|---|---|---|---|---|---|---|
| 1 | 6.939 | BV R | 0.0743 | 209.72061 | 43.03953 | 50.3978 |
| 2 | 8.830 | BV R | 0.1961 | 206.40971 | 12.66905 | 49.6022 |

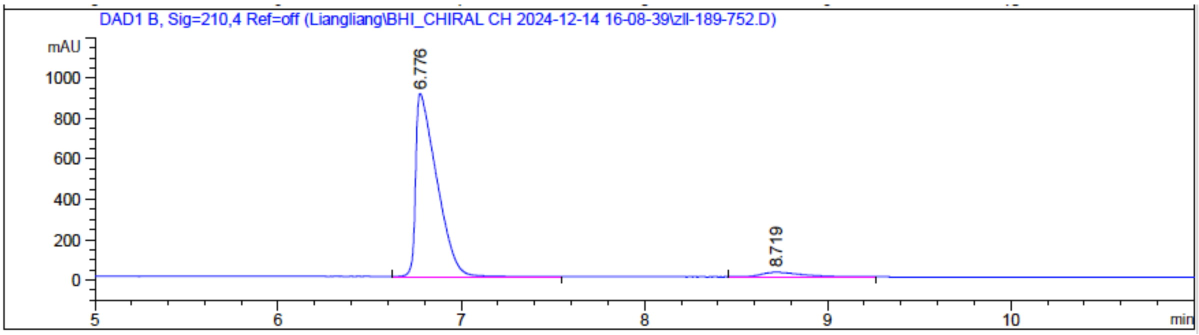


Signal 2: DAD1 B, Sig=210,4 Ref=off

| Peak # | RetTime [min] | Type | Width [min] | Area [mAU*s] | Height [mAU] | Area % |
|---|---|---|---|---|---|---|
| 1 | 6.776 | BV R | 0.1179 | 7212.93555 | 906.01630 | 95.2588 |
| 2 | 8.719 | BV R | 0.1885 | 358.99731 | 23.07057 | 4.7412 |

**(*S*)-*N*-(4-(3-((4-methylphenyl)sulfonamido)-1-oxo-1-(pyrrolidin-1-yl)propan-2-yl)phenyl)pivalamide (3i):** Following General Procedure C, *N*-(4-(1-tosylaziridin-2-yl)phenyl)pivalamide (**1i**) (55.9 mg, 0.15 mmol), and diethyl 2,6-dimethyl-4-(pyrrolidine-1-carbonyl)-1,4-dihydropyridine-3,5-dicarboxylate (**2a**) (105.1mg, 0.30 mmol) were used, affording the title compound as a yellow oil (27.9 mg, 39% yield) by using $^i$PrOAc/Acetone (20:1) as eluent. In an independent experiment, 31.7 mg (45% yield) were obtained, giving an average yield of 42% with 93:7 er. **$^1$H NMR** (400 MHz, $CDCl_3$) δ 7.71 – 7.68 (m, 2H), 7.48 – 7.44 (m, 2H), 7.33 (s, 1H), 7.26 (d, $J$ = 8.1 Hz, 2H), 7.16 – 7.12 (m, 2H), 5.36 (dd, $J$ = 7.9, 4.1 Hz, 1H), 3.83 (dd, $J$ = 9.7, 4.8 Hz, 1H), 3.54 – 3.43 (m, 1H), 3.40 – 3.23 (m, 3H), 3.22 – 3.11 (m, 1H), 2.93 – 2.87 (m, 1H), 2.40 (s, 3H), 1.86 – 1.67 (m, 4H), 1.29 (s, 9H). **$^{13}$C NMR** (101 MHz, $CDCl_3$) δ 176.8, 169.9, 143.4, 137.7, 137.4, 131.9, 129.8, 128.8, 127.1, 120.8, 51.2, 47.2, 46.2, 39.7, 27.7, 26.0, 24.2, 21.6. **HRMS** (ESI) calcd. for ($C_{25}H_{34}N_3O_4S$) $[M+H]^+$: 472.2265, found 472.2253. **IR** (neat): 3375, 2969, 2927, 2874, 1662, 1515, 1449, 1409, 1318, 1157, 1093, 816, 754, 661, 551. **Optical rotation**: $[\alpha]^{26}_D$ = 7.7 (c 0.2, $CHCl_3$, 93:7 er). The enantiomeric excess of **3i** was determined by SFC analysis on a Chiralpak IC-3 column ($CO_2$/MeOH = 70/30, column temperature 35 °C, flow rate 1.2 mL/min) with retention time 4.79 min (major) and 7.72 min (minor).

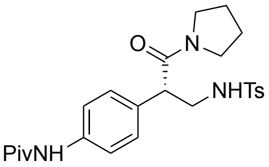

**3i**

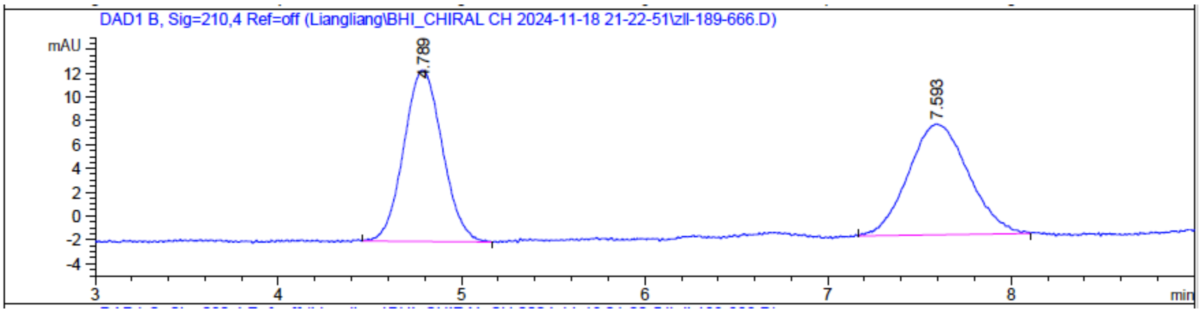

Signal 2: DAD1 B, Sig=210,4 Ref=off

| Peak # | RetTime [min] | Type | Width [min] | Area [mAU*s] | Height [mAU] | Area % |
|---|---|---|---|---|---|---|
| 1 | 4.789 | VV R | 0.1775 | 210.74870 | 14.37927 | 49.8559 |
| 2 | 7.593 | VV R | 0.2714 | 211.96666 | 9.31718 | 50.1441 |

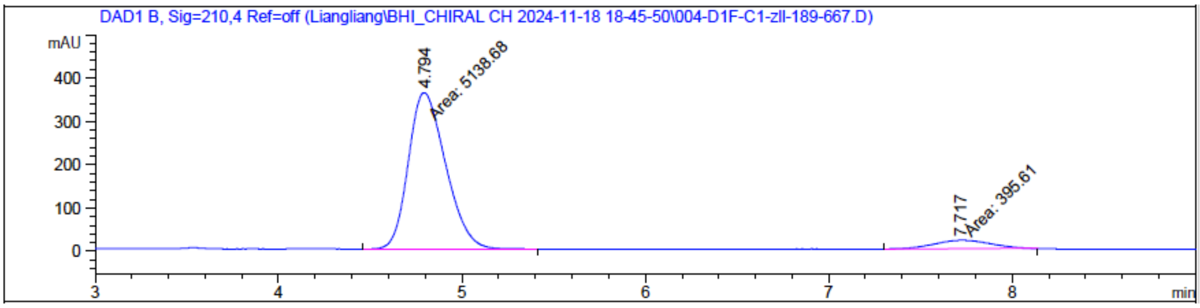


Signal 2: DAD1 B, Sig=210,4 Ref=off

| Peak # | RetTime [min] | Type | Width [min] | Area [mAU*s] | Height [mAU] | Area % |
|---|---|---|---|---|---|---|
| 1 | 4.794 | MM | 0.2368 | 5138.68457 | 361.65356 | 92.8517 |
| 2 | 7.717 | MM | 0.3479 | 395.61029 | 18.95156 | 7.1483 |

**(*S*)-*N*-(2-(4-(cyanomethyl)phenyl)-3-oxo-3-(pyrrolidin-1-yl)propyl)-4-methylbenzenesulfonamide(3j):** Following General Procedure C, 2-(4-(1-tosylaziridin-2-yl)phenyl)acetonitrile (**1j**) (46.8 mg, 0.15 mmol), and diethyl 2,6-dimethyl-4-(pyrrolidine-1-carbonyl)-1,4-dihydropyridine-3,5-dicarboxylate (**2a**) (105.1mg, 0.30 mmol) were used, affording the title compound as a white solid (36.8 mg, 60% yield) by using $^{i}$PrOAc/Acetone (20:1) as eluent. In an independent experiment, 39.8 mg (64% yield) were obtained, giving an average yield of 62% with 93:7 er. **M.p.**: 63-67 °C. **$^{1}$H NMR** (400 MHz, $CDCl_3$) δ 7.72 – 7.68 (m, 2H), 7.30 – 7.24 (m, 4H), 7.26 – 7.19 (m, 2H), 5.40 (dd, $J$ = 8.9, 4.8 Hz, 1H), 3.90 (dd, $J$ = 9.7, 4.7 Hz, 1H), 3.70 (s, 2H), 3.56 – 3.45 (m, 1H), 3.43 – 3.27 (m, 3H), 3.23 – 3.12 (m, 1H), 2.92 – 2.85 (m, 1H), 2.40 (s, 3H), 1.89 – 1.70 (m, 4H). **$^{13}$C NMR** (101 MHz, $CDCl_3$) δ 169.6, 143.4, 137.4, 136.3, 129.9, 129.6, 129.1, 128.9, 127.0, 117.7, 51.4, 47.1, 46.2, 26.0, 24.2, 23.4, 21.6. **HRMS** (ESI) calcd. for ($C_{22}H_{25}N_3NaO_3S$) $[M+Na]^+$: 434.1509, found 434.1504. **IR** (neat): 3170, 2954, 2926, 2877, 1624, 1513, 1449, 1421, 1328, 1158, 1093, 816, 753, 660, 552. **Optical rotation**: $[\alpha]^{26}_{D}$ = 4.3 (c 0.3, $CHCl_3$, 93:7 er). The enantiomeric excess of **3j** was determined by SFC analysis on a Chiralpak IH-3 column ($CO_2$/*i*PrOH with a gradient from 100% to 80% $CO_2$ in 5 min, then maintained at 80% $CO_2$, column temperature 35 °C, flow rate 1.2 mL/min) with retention time 13.62 min (minor) and 14.55 min (major).

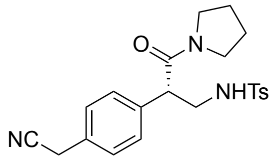


**3j**

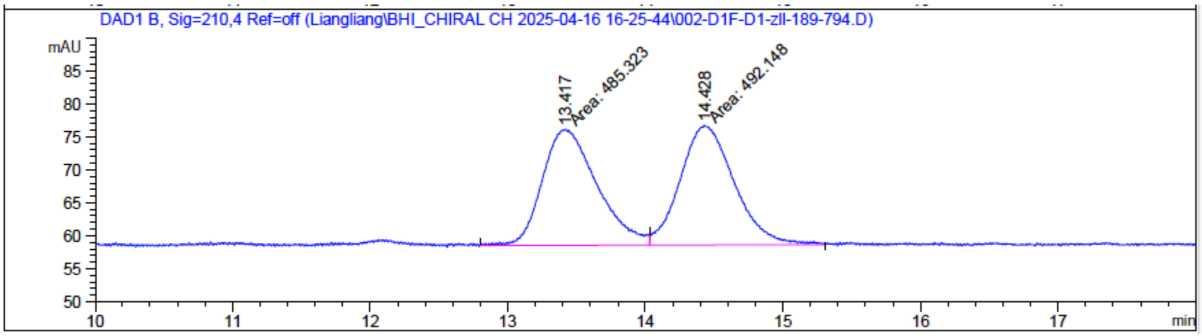


Signal 2: DAD1 B, Sig=210,4 Ref=off

| Peak # | RetTime [min] | Type | Width [min] | Area [mAU*s] | Height [mAU] | Area % |
|---|---|---|---|---|---|---|
| 1 | 13.417 | MF | 0.4591 | 485.32251 | 17.61970 | 49.6509 |
| 2 | 14.428 | FM | 0.4528 | 492.14801 | 18.11439 | 50.3491 |

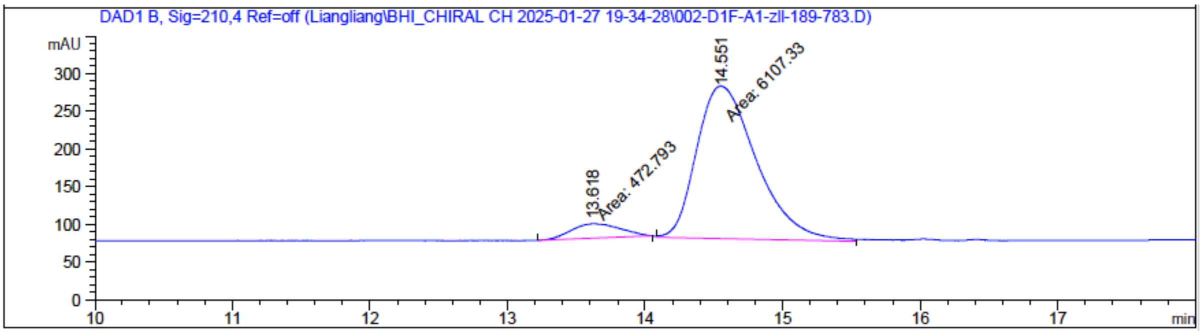


Signal 2: DAD1 B, Sig=210,4 Ref=off

| Peak # | RetTime [min] | Type | Width [min] | Area [mAU*s] | Height [mAU] | Area % |
|---|---|---|---|---|---|---|
| 1 | 13.618 | MM | 0.4100 | 472.79315 | 19.21840 | 7.1852 |
| 2 | 14.551 | MM | 0.5030 | 6107.32910 | 202.37830 | 92.8148 |

***tert*-butyl (*S*)-5-(3-((4-methylphenyl)sulfonamido)-1-oxo-1-(pyrrolidin-1-yl)propan-2-yl)-1*H*-indole-1-carboxylate (3k):** Following General Procedure C, *tert*-butyl 5-(1-tosylaziridin-2-yl)-1*H*-indole-1-carboxylate (**1k**) (61.8mg, 0.15 mmol), and diethyl 2,6-dimethyl-4-(pyrrolidine-1-carbonyl)-1,4-dihydropyridine-3,5-dicarboxylate (**2a**) (105.1mg, 0.30 mmol) were used, affording the title compound as a yellow oil (38.9 mg, 51% yield) by using $^{i}$PrOAc/Acetone (20:1) as eluent. In an independent experiment, 37.5 mg (49% yield) were obtained, giving an average yield of 50% with 95:5 er. **$^{1}$H NMR** (400 MHz, $CDCl_3$) δ 8.05 (d, $J$ = 8.5 Hz, 1H), 7.71 – 7.67 (m, 2H), 7.58 (d, $J$ = 3.7 Hz, 1H), 7.38 (d, $J$ = 1.7 Hz, 1H), 7.24 (d, $J$ = 8.1 Hz, 2H), 7.13 (dd, $J$ = 8.5, 1.8 Hz, 1H), 6.49 (dd, $J$ = 3.7, 0.8 Hz, 1H), 5.40 (dd, $J$ = 8.9, 4.6 Hz, 1H), 3.95 (dd, $J$ = 9.7, 4.8 Hz, 1H), 3.56 – 3.45 (m, 1H), 3.43 – 3.21 (m, 4H), 2.95 – 2.84 (m, 1H), 2.38 (s, 3H), 1.86 – 1.67 (m, 4H), 1.65 (s, 9H). **$^{13}$C NMR** (101 MHz, $CDCl_3$) δ 170.3, 149.7, 143.3, 137.5, 134.7, 131.2, 130.5, 129.8, 127.0, 126.8, 124.4, 120.5, 115.8, 107.2, 84.2, 51.7, 47.5, 46.19, 46.16, 28.3, 26.0, 24.2, 21.6. **HRMS** (ESI) calcd. for

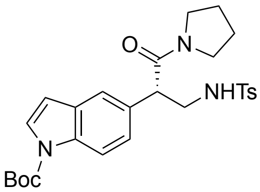


**3k**

($C_{27}H_{34}N_3O_5S$) [M+H]$^+$: 512.2214, found 512.2208. **IR** (neat): 3155, 2975, 2929, 2875, 1732, 1623, 1445, 1372, 1330, 1258, 1159, 1131, 1084, 1024, 815, 752, 662, 551. **Optical rotation**: $[\alpha]^{26}_{D}$ = 11.9 (c 0.3, $CHCl_3$, 95:5 er). The enantiomeric excess of **3k** was determined by SFC analysis on a Chiralpak IC-3 column ($CO_2$/MeOH = 70/30, column temperature 35 °C, flow rate 1.2 mL/min) with retention time 5.79 min (major) and 7.36 min (minor).

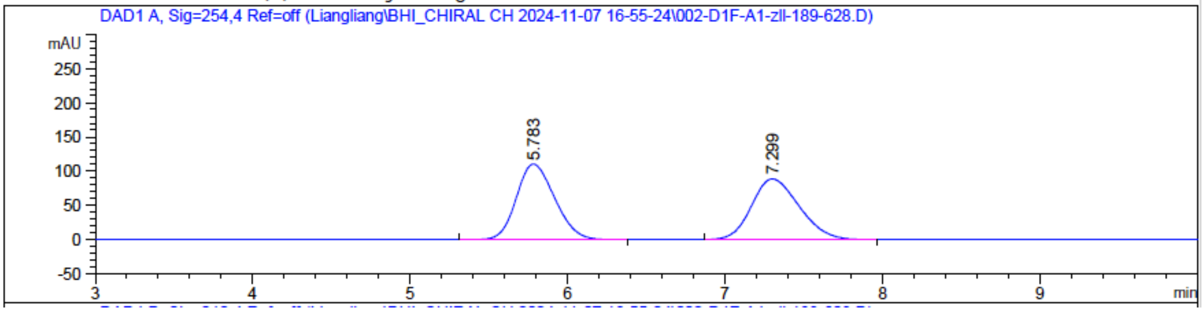


Signal 1: DAD1 A, Sig=254,4 Ref=off

| Peak # | RetTime [min] | Type | Width [min] | Area [mAU*s] | Height [mAU] | Area % |
|---|---|---|---|---|---|---|
| 1 | 5.783 | VV R | 0.2493 | 1859.32556 | 110.25118 | 50.0481 |
| 2 | 7.299 | BV R | 0.2779 | 1855.74805 | 88.41538 | 49.9519 |

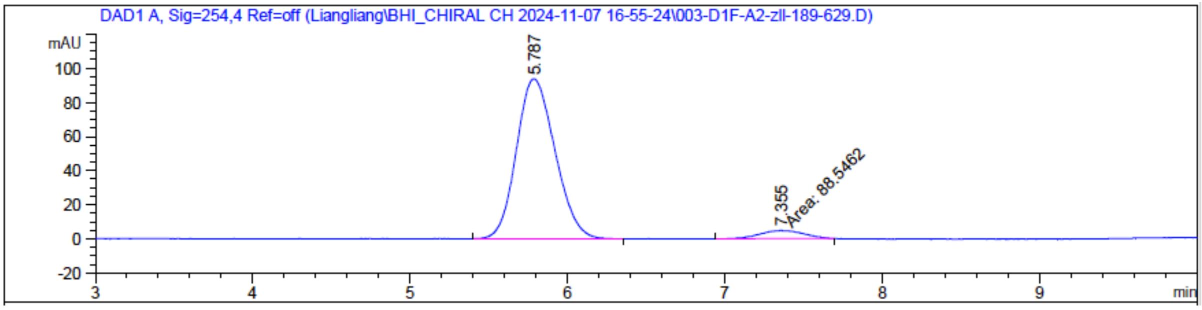


Signal 1: DAD1 A, Sig=254,4 Ref=off

| Peak # | RetTime [min] | Type | Width [min] | Area [mAU*s] | Height [mAU] | Area % |
|---|---|---|---|---|---|---|
| 1 | 5.787 | VV R | 0.2521 | 1602.81055 | 93.90415 | 94.7648 |
| 2 | 7.355 | MM | 0.3140 | 88.54623 | 4.69933 | 5.2352 |

**(*S*)-*N*-(2-(bicyclo[4.2.0]octa-1(6),2,4-trien-3-yl)-3-oxo-3-(pyrrolidin-1-yl)propyl)-4-methylbenzenesulfonamide (3l):** Following General Procedure C, 2-(bicyclo[4.2.0]octa-1(6),2,4-trien-3-yl)-1-tosylaziridine (**1l**) (34.4 mg, 0.15 mmol), and diethyl 2,6-dimethyl-4-(pyrrolidine-1-carbonyl)-1,4-dihydropyridine-3,5-dicarboxylate (**2a**) (105.1mg, 0.30 mmol) were used, affording the title compound as a yellow oil (25.0 mg, 42% yield) by using $^i$PrOAc/Acetone (20:1) as eluent. In an independent experiment, 27.8 mg (47% yield) were obtained, giving an average yield of 44% with 95:5 er. **$^1$H NMR** (400 MHz, $CDCl_3$) δ 7.72 – 7.68 (m, 2H), 7.26 (d, *J* = 8.1 Hz, 2H), 7.02 – 6.95 (m, 2H), 6.88 (s, 1H), 5.35 (dd, *J* = 9.2, 4.6 Hz, 1H), 3.81 (dd, *J* = 9.8, 4.8 Hz, 1H), 3.55 – 3.44 (m, 1H),

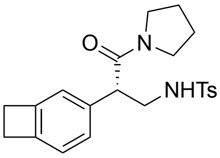


**3l**

3.41 – 3.25 (m, 3H), 3.24 – 3.12 (m, 1H), 3.11 (s, 4H), 2.98 – 2.88 (m, 1H), 2.40 (s, 3H), 1.88 – 1.69 (m, 4H). **$^{13}$C NMR** (101 MHz, $CDCl_3$) δ 170.2, 146.7, 145.4, 143.3, 137.5, 134.7, 129.8, 127.1, 126.9, 123.2, 122.3, 52.4, 47.6, 46.18, 46.15, 29.53, 29.48, 26.0, 24.2, 21.6. **HRMS** (ESI) calcd. for ($C_{22}H_{27}N_2O_3S$) $[M+H]^+$: 399.1737, found 399.1735. **IR** (neat): 3193, 2969, 2927, 2878, 1623, 1447, 1329, 1158, 1093, 816, 753, 661, 551. **Optical rotation**: $[\alpha]^{26}_{D}$ = 13.8 (c 0.3, $CHCl_3$, 95:5 er). The enantiomeric excess of **3l** was determined by SFC analysis on a Chiralpak ID-3 column ($CO_2$/MeOH with a gradient from 100% to 70% $CO_2$ in 5 min, then maintained at 70% $CO_2$, column temperature 35 °C, flow rate 1.2 mL/min) with retention time 7.38 min (major) and 10.29 min (minor).

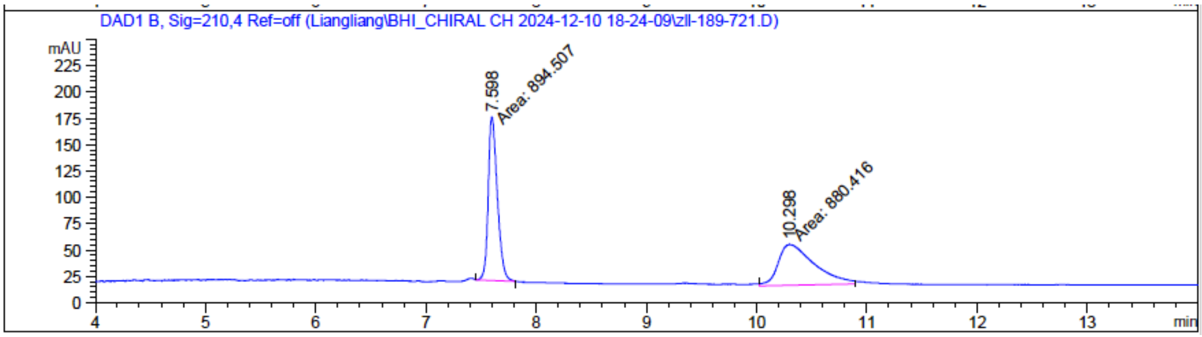


Signal 2: DAD1 B, Sig=210,4 Ref=off

| Peak # | RetTime [min] | Type | Width [min] | Area [mAU*s] | Height [mAU] | Area % |
|---|---|---|---|---|---|---|
| 1 | 7.598 | MM | 0.0958 | 894.50696 | 155.63892 | 50.3969 |
| 2 | 10.298 | MM | 0.3791 | 880.41614 | 38.71000 | 49.6031 |

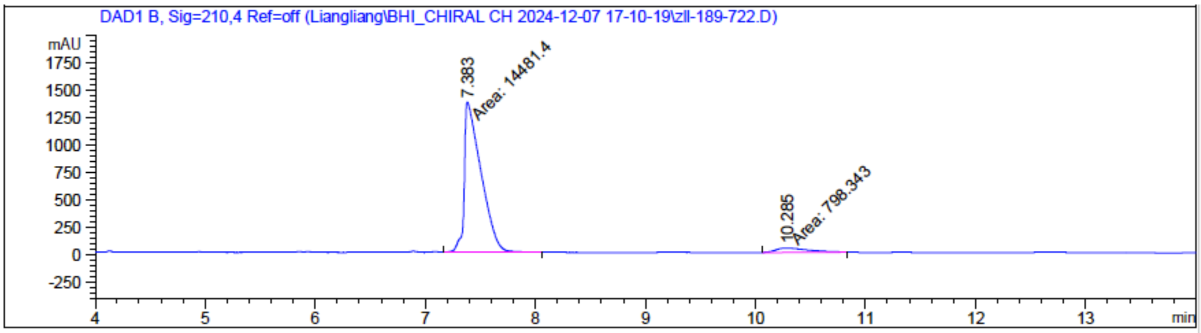


Signal 2: DAD1 B, Sig=210,4 Ref=off

| Peak # | RetTime [min] | Type | Width [min] | Area [mAU*s] | Height [mAU] | Area % |
|---|---|---|---|---|---|---|
| 1 | 7.383 | MM | 0.1765 | 1.44814e4 | 1367.31665 | 94.7751 |
| 2 | 10.285 | MM | 0.3383 | 798.34344 | 39.33279 | 5.2249 |

**methyl (*S*)-2-methyl-5-(3-((4-methylphenyl)sulfonamido)-1-oxo-1-(pyrrolidin-1-yl)propan-2-yl)benzoate (3m):** Following General Procedure C, methyl 2-methyl-5-(1-tosylaziridin-2-yl)benzoate (**1m**) (51.8 mg, 0.15 mmol), and diethyl 2,6-dimethyl-4-(pyrrolidine-1-carbonyl)-1,4-dihydropyridine-3,5-dicarboxylate (**2a**) (105.1mg, 0.30 mmol) were used, affording the title compound as a yellow oil (37.5 mg, 56% yield) by using $^i$PrOAc/Acetone (20:1) as eluent. In an independent

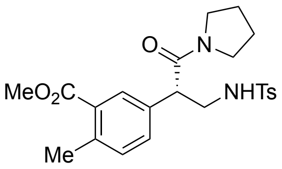


**3m**

experiment, 35.9 mg (54% yield) were obtained, giving an average yield of 55% with 93.5:6.5 er. **$^{1}$H NMR** (400 MHz, $CDCl_3$) δ 7.71 (d, *J* = 2.0 Hz, 1H), 7.71 – 7.67 (m, 2H), 7.29 – 7.24 (m, 2H), 7.24 – 7.21 (m, 1H), 7.17 (d, *J* = 7.9 Hz, 1H), 5.41 (dd, *J* = 9.0, 4.7 Hz, 1H), 3.91 – 3.88 (m, 1H), 3.87 (s, 3H), 3.55 – 3.45 (m, 1H), 3.42 – 3.27 (m, 3H), 3.25 – 3.13 (m, 1H), 2.92 – 2.86 (m, 1H), 2.54 (s, 3H), 2.39 (s, 3H), 1.89 – 1.69 (m, 4H). **$^{13}$C NMR** (101 MHz, $CDCl_3$) δ 169.6, 167.7, 143.4, 139.9, 137.5, 133.8, 132.6, 131.5, 130.4, 130.3, 129.8, 127.0, 52.1, 51.3, 47.1, 46.3, 46.2, 26.0, 24.2, 21.6, 21.4. **HRMS** (ESI) calcd. for ($C_{23}H_{29}N_2O_5S$) $[M+H]^+$: 445.1792, found 445.1788. **IR** (neat): 3207, 2951, 2876, 1722, 1624, 1438, 1328, 1304, 1264, 1159, 1084, 816, 755, 661, 551. **Optical rotation**: $[\alpha]^{26}_{D}$ = 5.8 (c 0.3, $CHCl_3$, 93.5:6.5 er). The enantiomeric excess of **3m** was determined by SFC analysis on a Chiralpak ID-3 column ($CO_2$/MeOH with a gradient from 100% to 70% $CO_2$ in 5 min, then maintained at 70% $CO_2$, column temperature 35 °C, flow rate 1.2 mL/min) with retention time 7.18 min (major) and 9.81 min (minor).

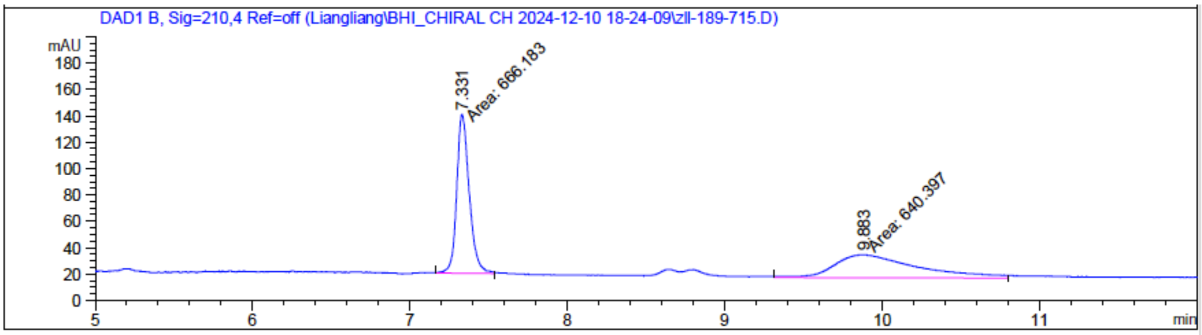


Signal 2: DAD1 B, Sig=210,4 Ref=off

| Peak # | RetTime [min] | Type | Width [min] | Area [mAU*s] | Height [mAU] | Area % |
|---|---|---|---|---|---|---|
| 1 | 7.331 | MM | 0.0922 | 666.18317 | 120.39897 | 50.9868 |
| 2 | 9.883 | MM | 0.6142 | 640.39691 | 17.37876 | 49.0132 |

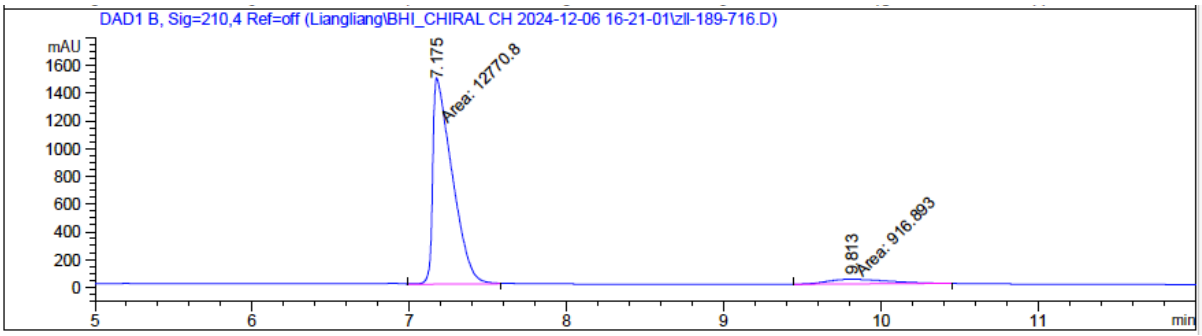


Signal 2: DAD1 B, Sig=210,4 Ref=off

| Peak # | RetTime [min] | Type | Width [min] | Area [mAU*s] | Height [mAU] | Area % |
|---|---|---|---|---|---|---|
| 1 | 7.175 | MF | 0.1435 | 1.27708e4 | 1483.39075 | 93.3013 |
| 2 | 9.813 | MM | 0.4588 | 916.89337 | 33.31081 | 6.6987 |

**(*S*)-*N*-(2-(3-chloro-4-methylphenyl)-3-oxo-3-(pyrrolidin-1-yl)propyl)-4-methylbenzenesulfonamide (3n):** Following General Procedure C, 2-(3-chloro-4-methylphenyl)-1-tosylaziridine (**1n**) (63.1 mg, 0.15 mmol), and diethyl 2,6-dimethyl-4-(pyrrolidine-1-carbonyl)-1,4-dihydropyridine-3,5-dicarboxylate (**2a**) (105.1mg, 0.30 mmol) were used, affording the title compound as a yellow oil (23.9 mg, 38% yield) by using $^{i}$PrOAc/Acetone (20:1) as eluent. In an independent experiment, 26.9 mg (43% yield) were obtained, giving an average yield of 40% with 92.5:7.5 er. **$^{1}$H NMR** (400 MHz, $CDCl_3$) δ 7.71 – 7.68 (m, 2H), 7.26 (d, *J* = 8.1 Hz, 2H), 7.17 (d, *J* = 1.8 Hz, 1H), 7.14 (d, *J* = 7.8 Hz, 1H), 6.99 (dd, *J* = 7.8, 1.9 Hz, 1H), 5.42 (dd, *J* = 9.1, 4.8 Hz, 1H), 3.82 (dd, *J* = 9.7, 4.7 Hz, 1H), 3.55 – 3.44 (m, 1H), 3.42 – 3.27 (m, 3H), 3.22 – 3.11 (m, 1H), 2.96 – 2.90 (m, 1H), 2.40 (s, 3H), 2.31 (s, 3H), 1.89 – 1.71 (m, 4H). **$^{13}$C NMR** (101 MHz, $CDCl_3$) δ 169.5, 143.4, 137.4, 135.8, 135.4, 135.0, 131.6, 129.8, 128.6, 127.0, 126.5, 51.1, 47.1, 46.3, 46.2, 26.0, 24.2, 21.6, 19.8. **HRMS** (ESI) calcd. for ($C_{21}H_{25}ClN_2NaO_3S$) $[M+Na]^+$: 443.1167, found 443.1169. IR (neat): 3198, 2971, 2925, 2877, 1625, 1446, 1330, 1159, 1093, 815, 756, 662, 551. **Optical rotation**: $[\alpha]^{26}_{D}$ = 9.6 (c 0.1, $CHCl_3$, 92.5:7.5 er). The enantiomeric excess of **3n** was determined by SFC analysis on a Chiralpak ID-3 column ($CO_2$/MeOH with a gradient from 100% to 70% $CO_2$ in 5 min, then maintained at 70% $CO_2$, column temperature 35 °C, flow rate 1.2 mL/min) with retention time 6.93 min (major) and 8.60 min (minor).

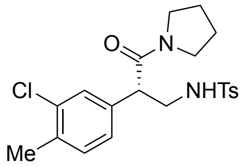

**3n**

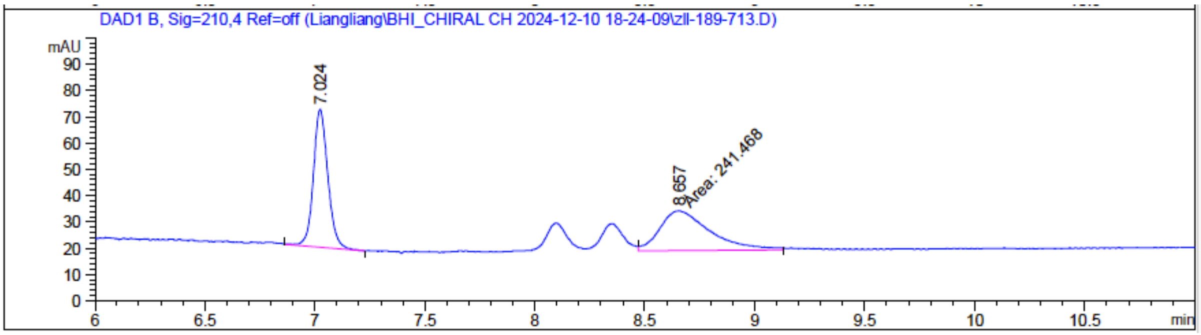


Signal 2: DAD1 B, Sig=210,4 Ref=off

| Peak # | RetTime [min] | Type | Width [min] | Area [mAU*s] | Height [mAU] | Area % |
|---|---|---|---|---|---|---|
| 1 | 7.024 | VV R | 0.0694 | 239.77086 | 52.38353 | 49.8237 |
| 2 | 8.657 | MM | 0.2659 | 241.46771 | 15.13455 | 50.1763 |

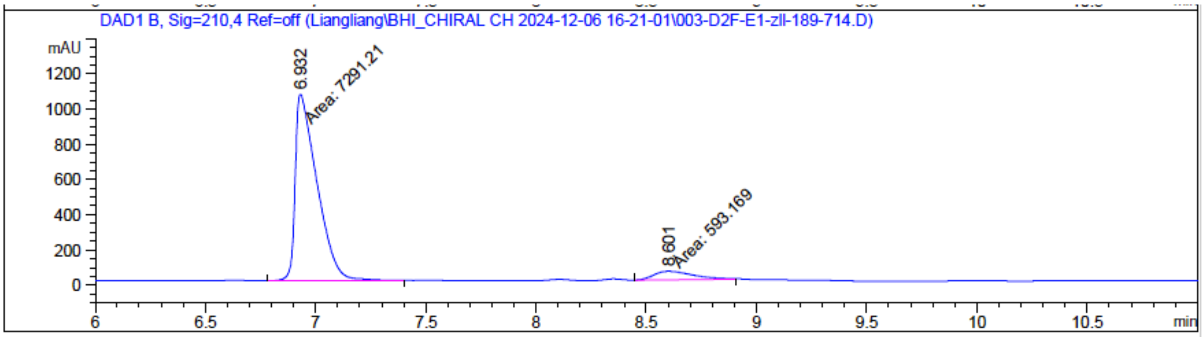

Signal 2: DAD1 B, Sig=210,4 Ref=off

| Peak # | RetTime [min] | Type | Width [min] | Area [mAU*s] | Height [mAU] | Area % |
|---|---|---|---|---|---|---|
| 1 | 6.932 | FM | 0.1146 | 7291.21338 | 1060.52039 | 92.4767 |
| 2 | 8.601 | MM | 0.2052 | 593.16876 | 48.17446 | 7.5233 |

**(*S*)-*N*-(2-(dibenzo[*b*,*d*]furan-2-yl)-3-oxo-3-(pyrrolidin-1-yl)propyl)-4-methylbenzenesulfonamide (3o):** Following General Procedure C, 2-(dibenzo[*b,d*]furan-2-yl)-1-tosylaziridine (**1o**) (54.5mg, 0.15 mmol), and diethyl 2,6-dimethyl-4-(pyrrolidine-1-carbonyl)-1,4-dihydropyridine-3,5-dicarboxylate (**2a**) (105.1mg, 0.30 mmol) were used, affording the title compound as a yellow oil (31.8 mg, 46% yield) by using $^{i}$PrOAc/Acetone (20:1) as eluent. In an independent experiment, 34.4 mg (50% yield) were obtained, giving an average yield of 48% with 95:5 er. **$^{1}$H NMR** (400 MHz, $CDCl_3$) δ 7.91 (d, *J* = 7.7 Hz, 1H), 7.78 (d, *J* = 1.9 Hz, 1H), 7.71 – 7.68 (m, 2H), 7.55 (d, *J* = 8.2 Hz, 1H), 7.51 – 7.42 (m, 2H), 7.36 – 7.27 (m, 2H), 7.22 (d, *J* = 8.1 Hz, 2H), 5.50 (dd, *J* = 9.0, 4.7 Hz, 1H), 4.05 (dd, *J* = 9.6, 4.8 Hz, 1H), 3.59 – 3.48 (m, 1H), 3.48 – 3.27 (m, 4H), 2.99 – 2.89 (m, 1H), 2.35 (s, 3H), 1.88 – 1.66 (m, 4H). **$^{13}$C NMR** (101 MHz, $CDCl_3$) δ 170.2, 156.7, 155.7, 143.3, 137.5, 130.7, 129.8, 127.7, 127.4, 127.0, 125.1, 123.8, 123.1, 121.0, 120.2, 112.2, 111.9, 51.7, 47.6, 46.3, 46.2, 26.0, 24.2, 21.5. **HRMS** (ESI) calcd. for ($C_{26}H_{26}N_2NaO_4S$) $[M+Na]^+$: 485.1505, found 485.1502. **IR** (neat): 3169, 2974, 2925, 2876, 1622, 1479, 1448, 1328, 1197, 1157, 1093, 815, 751, 662, 551. **Optical rotation**: $[\alpha]^{26}_{D}$ = 1.4 (c 0.1, $CHCl_3$, 95:5 er). The enantiomeric excess of **3o** was determined by SFC analysis on a Chiralpak ID-3 column ($CO_2$/MeOH with a gradient from 100% to 70% $CO_2$ in 5 min, then maintained at 70% $CO_2$, column temperature 35 °C, flow rate 1.2 mL/min) with retention time 8.99 min (major) and 12.12 min (minor).

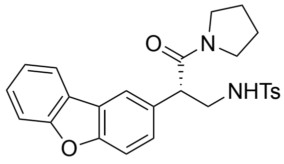


3o

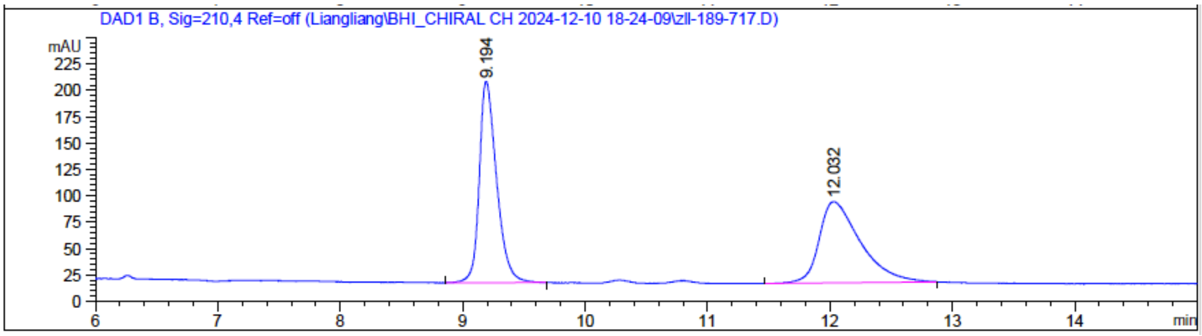


Signal 2: DAD1 B, Sig=210,4 Ref=off

| Peak # | RetTime [min] | Type | Width [min] | Area [mAU*s] | Height [mAU] | Area % |
|---|---|---|---|---|---|---|
| 1 | 9.194 | VV R | 0.1465 | 1881.75073 | 190.87582 | 51.0974 |
| 2 | 12.032 | VV R | 0.2884 | 1800.92603 | 76.84158 | 48.9026 |

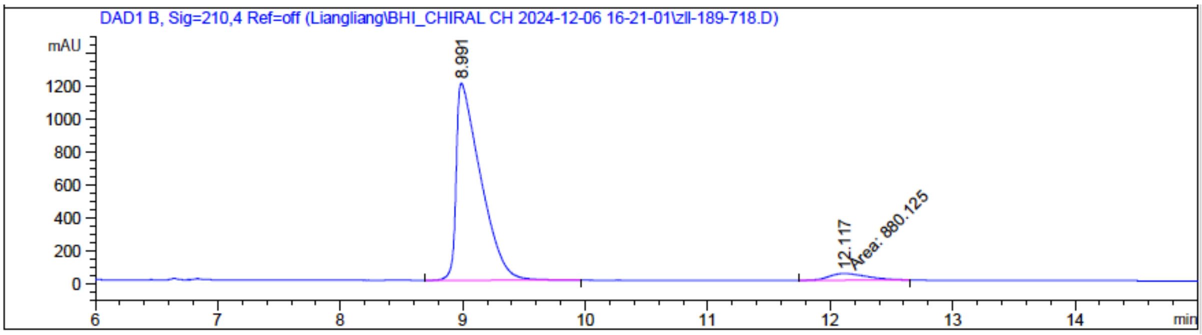


Signal 2: DAD1 B, Sig=210,4 Ref=off

| Peak # | RetTime [min] | Type | Width [min] | Area [mAU*s] | Height [mAU] | Area % |
|---|---|---|---|---|---|---|
| 1 | 8.991 | BV R | 0.1971 | 1.65973e4 | 1194.67676 | 94.9642 |
| 2 | 12.117 | MM | 0.3566 | 880.12537 | 41.13509 | 5.0358 |

**(*S*)-4-(*tert*-butyl)-*N*-(3-oxo-3-(pyrrolidin-1-yl)-2-(*p*-tolyl)propyl)benzenesulfonamide (3p):** Following General Procedure C, 1-((4-(*tert*-butyl)phenyl)sulfonyl)-2-(4-fluorophenyl)aziridine (**1p**) (50.0mg, 0.15 mmol), and diethyl 2,6-dimethyl-4-(pyrrolidine-1-carbonyl)-1,4-dihydropyridine-3,5-dicarboxylate (**2a**) (105.1mg, 0.30 mmol) were used, affording the title compound as a yellow oil (42.8 mg, 67% yield) by using $^i$PrOAc/Acetone (20:1) as eluent. In an independent experiment, 46.8 mg (73% yield) were obtained, giving an average yield of 70% with 96:4 er.

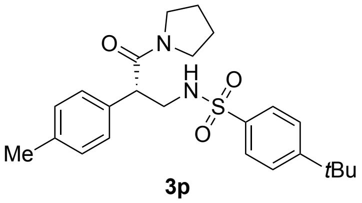


**3p**

**$^1$H NMR** (400 MHz, $CDCl_3$) δ 7.76 – 7.72 (m, 2H), 7.49 – 7.45 (m, 2H), 7.13 – 7.04 (m, 4H), 5.48 (dd, *J* = 9.1, 4.6 Hz, 1H), 3.84 (dd, *J* = 9.8, 4.8 Hz, 1H), 3.55 – 3.44 (m, 1H), 3.42 – 3.25 (m, 3H), 3.22 – 3.15 (m, 1H), 2.97 – 2.86 (m, 1H), 2.30 (s, 3H), 1.87 – 1.67 (m, 4H), 1.31 (s, 9H). **$^{13}$C NMR** (101 MHz, $CDCl_3$) δ 170.1, 156.2, 137.5, 137.4, 133.2, 129.8, 128.1, 126.9, 126.2, 51.5, 47.2, 46.1, 35.2, 31.2, 26.0, 24.1, 21.1. **HRMS** (ESI) calcd. for ($C_{24}H_{33}N_2O_3S$) $[M+H]^+$: 429.2206, found 429.2195. **IR** (neat): 3180, 2963, 2873, 1625, 1449, 1331, 1163, 1113, 1088, 834, 753, 625, 566, 551. **Optical rotation**: $[\alpha]^{26}_D$ = 13.2 (c 0.3, $CHCl_3$, 96:4 er). The enantiomeric excess of **3p** was determined by SFC analysis on a Chiralpak ID-3 column ($CO_2$/MeOH with a gradient from 100% to 70% $CO_2$ in 5 min, then maintained at 70% $CO_2$, column temperature 35 °C, flow rate 1.2 mL/min) with retention time 8.99 min (major) and 12.12 min (minor).

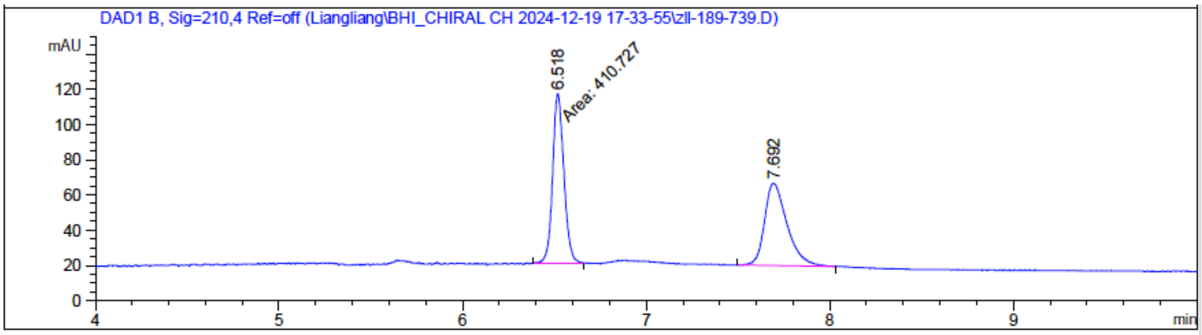

Signal 2: DAD1 B, Sig=210,4 Ref=off

| Peak # | RetTime [min] | Type | Width [min] | Area [mAU*s] | Height [mAU] | Area % |
|---|---|---|---|---|---|---|
| 1 | 6.518 | MM | 0.0710 | 410.72675 | 96.44205 | 50.5926 |
| 2 | 7.692 | VB R | 0.1275 | 401.10519 | 46.80896 | 49.4074 |

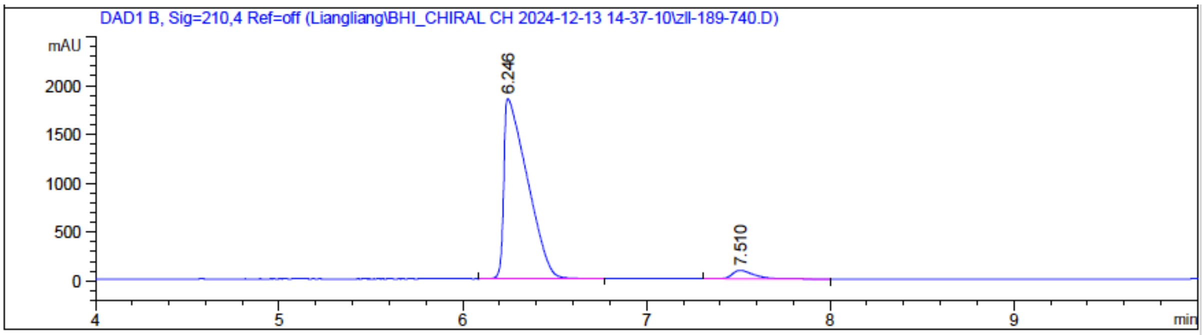


Signal 2: DAD1 B, Sig=210,4 Ref=off

| Peak # | RetTime [min] | Type | Width [min] | Area [mAU*s] | Height [mAU] | Area % |
|---|---|---|---|---|---|---|
| 1 | 6.246 | VV R | 0.1296 | 1.63727e4 | 1844.29492 | 95.7860 |
| 2 | 7.510 | BV R | 0.1224 | 720.29517 | 87.13743 | 4.2140 |

**(*S*)-4-methoxy-*N*-(3-oxo-3-(pyrrolidin-1-yl)-2-(*p*-tolyl)propyl)benzenesulfonamide (3q):** Following General Procedure C, 1-((4-methoxyphenyl)sulfonyl)-2-(*p*-tolyl)aziridine (**1q**) (45.5mg, 0.15 mmol), and diethyl 2,6-dimethyl-4-(pyrrolidine-1-carbonyl)-1,4-dihydropyridine-3,5-dicarboxylate (**2a**) (105.1mg, 0.30 mmol) were used, affording the title compound as a yellow oil (34.6 mg, 57% yield) by using $^{i}$PrOAc/Acetone (20:1) as eluent. In an independent experiment, 32.2 mg (53% yield) were obtained, giving an average yield of 55% with 93.5:6.5 er.

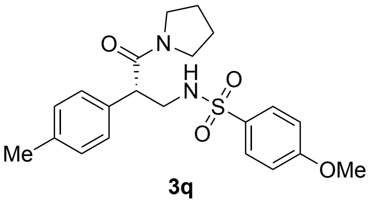


**3q**

**$^{1}$H NMR** (400 MHz, $CDCl_3$) δ 7.76 – 7.73 (m, 2H), 7.12 – 7.06 (m, 4H), 6.95 – 6.91 (m, 2H), 5.33 (dd, *J* = 9.2, 4.6 Hz, 1H), 3.86 – 3.83 (m, 4H), 3.53 – 3.45 (m, 1H), 3.42 – 3.26 (m, 3H), 3.22 – 3.11 (m, 1H), 2.96 – 2.86 (m, 1H), 2.30 (s, 3H), 1.87 – 1.68 (m, 4H). **$^{13}$C NMR** (101 MHz, $CDCl_3$) δ 170.1, 162.9, 137.6, 133.2, 132.1, 129.9, 129.2, 128.1, 114.4, 55.7, 51.5, 47.3, 46.19, 46.15, 26.0, 24.2, 21.2. **HRMS** (ESI) calcd. for ($C_{21}H_{27}N_2O_4S$) $[M+H]^+$: 403.1686, found 403.1669. **IR** (neat): 3163, 2972, 2876, 1625, 1597, 1498, 1449, 1330, 1259, 1156, 1095, 1023, 836, 566. **Optical rotation**: $[\alpha]^{26}_{D}$ = 14.9 (c 0.1, $CHCl_3$, 93.5:6.5 er). The enantiomeric excess of **3q** was determined by SFC analysis on a Chiralpak ID-3 column ($CO_2$/MeOH with a gradient from 100% to 70% $CO_2$ in 5 min, then maintained at 70% $CO_2$, column temperature 35 °C, flow rate 1.2 mL/min) with retention time 6.97 min (major) and 9.12 min (minor).

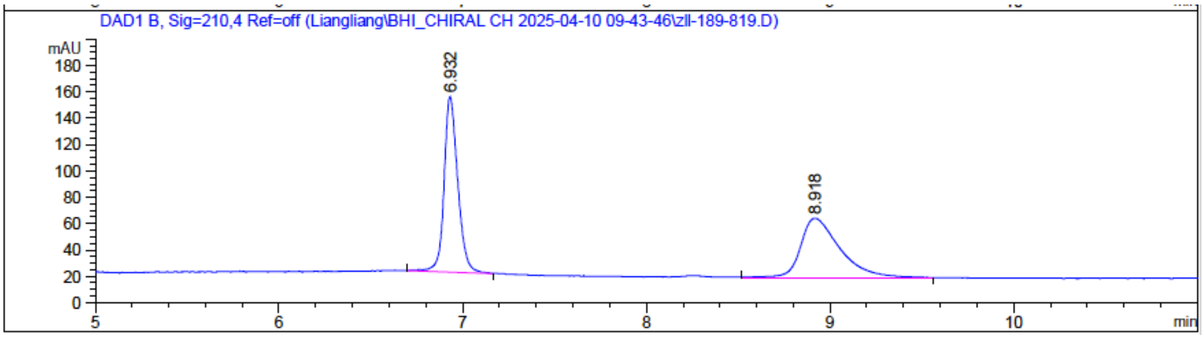


Signal 2: DAD1 B, Sig=210,4 Ref=off

| Peak # | RetTime [min] | Type | Width [min] | Area [mAU*s] | Height [mAU] | Area % |
|---|---|---|---|---|---|---|
| 1 | 6.932 | VB R | 0.0780 | 690.29565 | 133.13177 | 50.7730 |
| 2 | 8.918 | BB | 0.2123 | 669.27771 | 45.04617 | 49.2270 |

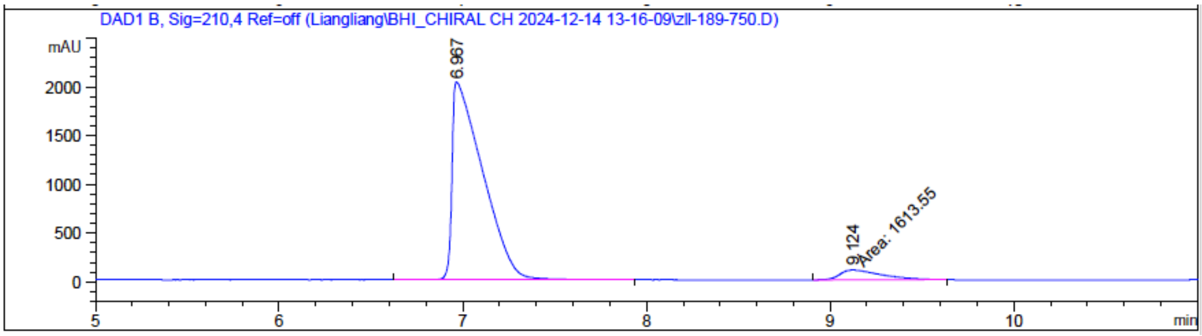


Signal 2: DAD1 B, Sig=210,4 Ref=off

| Peak # | RetTime [min] | Type | Width [min] | Area [mAU*s] | Height [mAU] | Area % |
|---|---|---|---|---|---|---|
| 1 | 6.967 | VV R | 0.1531 | 2.34501e4 | 2025.53516 | 93.5622 |
| 2 | 9.124 | MM | 0.2671 | 1613.55005 | 100.69684 | 6.4378 |

**(*S*)-*N*-(3-oxo-3-(pyrrolidin-1-yl)-2-(*p*-tolyl)propyl)naphthalene-2-sulfonamide (3r):** Following General Procedure C, 1-(naphthalen-2-ylsulfonyl)-2-(*p*-tolyl)aziridine (**1r**) (48.5mg, 0.15 mmol), and diethyl 2,6-dimethyl-4-(pyrrolidine-1-carbonyl)-1,4-dihydropyridine-3,5-dicarboxylate (**2a**) (105.1mg, 0.30 mmol) were used, affording the title compound as a yellow oil (24.0 mg, 38% yield) by using $^{i}$PrOAc/Acetone (20:1) as eluent. In an independent experiment, 20.0 mg (32% yield) were obtained, giving an average yield of 35% with 96.5:3.5 er. **$^{1}$H NMR** (400 MHz, $CDCl_3$) δ 8.39 (d, *J* = 1.4 Hz, 1H), 7.96 – 7.85 (m, 3H), 7.81 (dd, *J* = 8.7, 1.9 Hz, 1H), 7.66 – 7.54 (m, 2H), 7.10 – 7.01 (m, 4H), 5.61 (dd, *J* = 9.0, 4.7 Hz, 1H), 3.81 (dd, *J* = 9.7, 4.8 Hz, 1H), 3.52 – 3.41 (m, 1H), 3.42 – 3.30 (m, 2H), 3.32 – 3.21 (m, 1H), 3.18 – 3.08 (m, 1H), 2.89 – 2.79 (m, 1H), 2.27 (s, 3H), 1.76 – 1.61 (m, 4H). **$^{13}$C NMR** (101 MHz, $CDCl_3$) δ 170.1, 137.6, 137.5, 134.9, 133.0, 132.3, 129.9, 129.5, 129.3, 128.8, 128.2, 128.1, 128.0, 127.6, 122.5, 51.4, 47.3, 46.13, 46.11, 25.9, 24.1, 21.2. **HRMS** (ESI) calcd. for ($C_{24}H_{27}N_2O_3S$) $[M+H]^+$: 423.1737, found 423.1729. **IR**

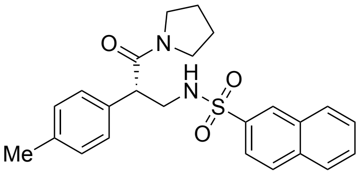


**3r**

(neat): 3175, 2971, 2951, 2925, 2875, 1621, 1512, 1447, 1328, 1227, 1157, 1131, 1075, 863, 819, 751, 656, 550, 479. **Optical rotation**: $[\alpha]^{26}_{D}$ = 5.7 (c 0.2, $CHCl_3$, 96.5:3.5 er). The enantiomeric excess of **3r** was determined by SFC analysis on a Chiralpak ID-3 column ($CO_2$/MeOH with a gradient from 100% to 70% $CO_2$ in 5 min, then maintained at 70% $CO_2$, column temperature 35 °C, flow rate 1.2 mL/min) with retention time 8.01 min (major) and 11.12 min (minor).

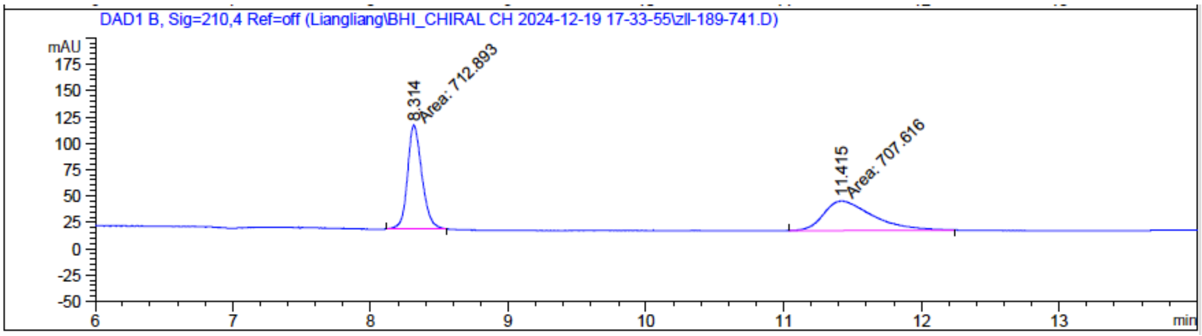


Signal 2: DAD1 B, Sig=210,4 Ref=off

| Peak # | RetTime [min] | Type | Width [min] | Area [mAU*s] | Height [mAU] | Area % |
|---|---|---|---|---|---|---|
| 1 | 8.314 | MM | 0.1210 | 712.89343 | 98.22937 | 50.1858 |
| 2 | 11.415 | MM | 0.4194 | 707.61584 | 28.12207 | 49.8142 |

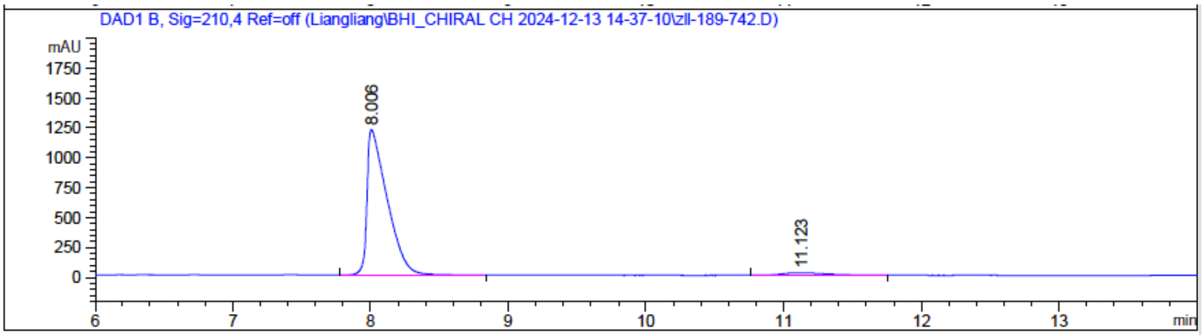


Signal 2: DAD1 B, Sig=210,4 Ref=off

| Peak # | RetTime [min] | Type | Width [min] | Area [mAU*s] | Height [mAU] | Area % |
|---|---|---|---|---|---|---|
| 1 | 8.006 | BV R | 0.1461 | 1.23226e4 | 1211.95471 | 96.4817 |
| 2 | 11.123 | VV R | 0.2615 | 449.35760 | 20.35649 | 3.5183 |

**4-methyl-*N*-((*S*)-2-((8*S*,9*R*,13*R*,14*R*)-13-methyl-17-oxo-7,8,9,11,12,13,14,15,16,17-decahydro-6*H*-cyclopenta[*a*]phenanthren-3-yl)-3-oxo-3-(pyrrolidin-1-yl)propyl)benzenesulfonamide (3s):** Following General Procedure C, (8*S*,9*R*,13*R*,14*R*)-13-methyl-3-(1-tosylaziridin-2-yl)-6,7,8,9,11,12,13,14,15,16-decahydro-17*H*-cyclopenta[*a*]phenanthren-17-one (**1s**) (67.4 mg, 0.15 mmol), and diethyl 2,6-dimethyl-4-(pyrrolidine-1-carbonyl)-1,4-dihydropyridine-3,5-dicarboxylate (**2a**) (105.1mg, 0.30 mmol) were used, affording the title compound as a yellow oil (20.1 mg, 24% yield) by using $^{i}$PrOAc/Acetone (20:1) as

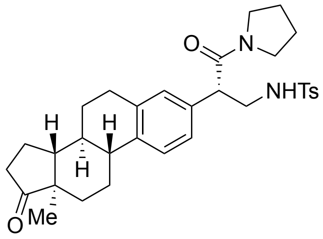


**3s**

eluent. In an independent experiment, 22.3 mg (27% yield) were obtained, giving an average yield of 26% with 94.5:5.5 dr. $^{1}$**H NMR** (400 MHz, $CDCl_3$) δ 7.73 – 7.68 (m, 2H), 7.30 – 7.23 (m, 2H), 7.20 (d, $J$ = 8.0 Hz, 1H), 6.97 – 6.91 (m, 2H), 5.28 (dd, $J$ = 9.2, 4.5 Hz, 1H), 3.82 (dd, $J$ = 9.8, 4.8 Hz, 1H), 3.57 – 3.46 (m, 1H), 3.42 – 3.28 (m, 3H), 3.42 – 3.14 (m, 1H), 3.02 – 2.94 (m, 1H), 2.89 – 2.81 (m, 2H), 2.55 – 2.46 (m, 1H), 2.40 (s, 3H), 2.30 – 2.22 (m, 1H), 2.19 – 1.71 (m, 9H), 1.67 – 1.35 (m, 6H), 0.90 (s, 3H). $^{13}$**C NMR** (101 MHz, $CDCl_3$) δ 220.8, 170.0, 143.4, 139.4, 137.5, 137.4, 133.5, 129.8, 128.7, 127.1, 126.1, 125.5, 51.5, 50.7, 48.1, 47.3, 46.22, 46.17, 44.5, 38.2, 36.0, 31.7, 29.5, 26.5, 26.0, 25.7, 24.2, 21.7, 21.6, 14.0. **HRMS** (ESI) calcd. for ($C_{32}H_{41}N_2O_4S$) $[M+H]^+$: 549.2782, found 549.2775. **IR** (neat): 3179, 2929, 2873, 1737, 1625, 1452, 1326, 1160, 1093, 817, 755, 662, 551. **Optical rotation**: $[\alpha]^{26}_{D}$ = 97.6 (c 0.1, $CHCl_3$, 94.5:5.5 dr). The enantiomeric excess of **3s** was determined by SFC analysis on a Chiralpak ID-3 column ($CO_2$/MeOH with a gradient from 100% to 70% $CO_2$ in 5 min, then maintained at 70% $CO_2$, column temperature 35 °C, flow rate 1.2 mL/min) with retention time 6.80 min (major) and 7.75 min (minor).

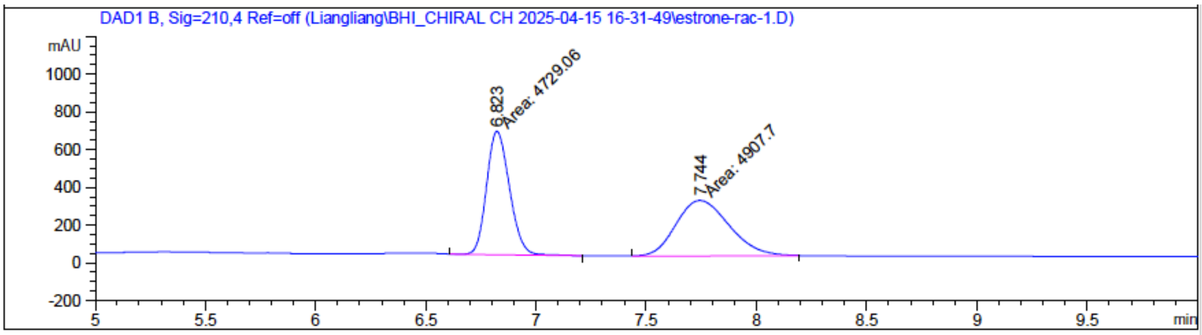


Signal 2: DAD1 B, Sig=210,4 Ref=off

| Peak # | RetTime [min] | Type | Width [min] | Area [mAU*s] | Height [mAU] | Area % |
|---|---|---|---|---|---|---|
| 1 | 6.823 | MM | 0.1204 | 4729.06104 | 654.41962 | 49.0732 |
| 2 | 7.744 | MM | 0.2788 | 4907.69629 | 293.39801 | 50.9268 |

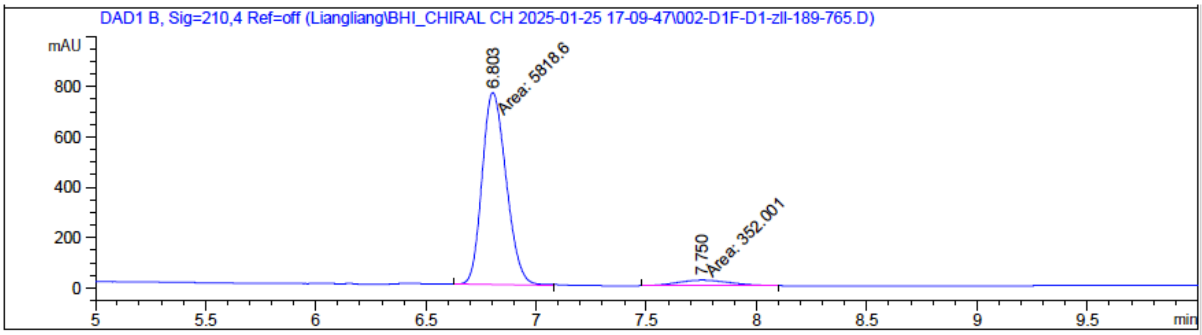


Signal 2: DAD1 B, Sig=210,4 Ref=off

| Peak # | RetTime [min] | Type | Width [min] | Area [mAU*s] | Height [mAU] | Area % |
|---|---|---|---|---|---|---|
| 1 | 6.803 | MF | 0.1268 | 5818.59619 | 764.72528 | 94.2955 |
| 2 | 7.750 | MM | 0.2666 | 352.00061 | 22.00354 | 5.7045 |

**4-methyl-*N*-((*R*)-2-((8*S*,9*R*,13*R*,14*R*)-13-methyl-17-oxo-7,8,9,11,12,13,14,15,16,17-decahydro-6*H*-cyclopenta[*a*]phenanthren-3-yl)-3-oxo-3-(pyrrolidin-1-yl)propyl)benzenesulfonamide (*epi*-3s):** Following General Procedure C, (8*S*,9*R*,13*R*,14*R*)-13-methyl-3-(1-tosylaziridin-2-yl)-6,7,8,9,11,12,13,14,15,16-decahydro-17*H*-cyclopenta[*a*]phenanthren-17-one (**1s**) (67.4 mg, 0.15 mmol), and diethyl 2,6-dimethyl-4-(pyrrolidine-1-carbonyl)-1,4-dihydropyridine-3,5-dicarboxylate (**2a**) (105.1mg, 0.30 mmol) and *ent*-**L1** (3a*S*,3a'*S*,8a*R*,8a'*R*)-2,2'-(1,3-bis(4-(*tert*-butyl)phenyl)propane-2,2-diyl)bis(3a,8a-dihydro-8*H*-indeno[1,2-*d*]oxazole) (14.0 mg, 0.0225 mol) were used, affording the title compound as a yellow oil (20.0 mg, 24% yield) by using $^{i}$PrOAc/Acetone (20:1) as eluent. In an independent experiment, 20.2 mg (25% yield) were obtained, giving an average yield of 24% with 93.5:6.5 dr. **$^{1}$H NMR** (400 MHz, $CDCl_3$) δ 7.72 – 7.68 (m, 2H), 7.30 – 7.23 (m, 2H), 7.23 – 7.17 (m, 1H), 7.00 – 6.93 (m, 1H), 6.95 – 6.89 (m, 1H), 5.29 (dd, *J* = 9.2, 4.6 Hz, 1H), 3.82 (dd, *J* = 9.8, 4.8 Hz, 1H), 3.56 – 3.46 (m, 1H), 3.42 – 3.27 (m, 3H), 3.24 – 3.13 (m, 1H), 3.00 – 2.94 (m, 1H), 2.87 – 2.82 (m, 2H), 2.54 – 2.46 (m, 1H), 2.40 (s, 3H), 2.24 (t, *J* = 10.5 Hz, 1H), 2.17 – 1.72 (m, 9H), 1.67 – 1.37 (m, 6H), 0.90 (s, 3H). **$^{13}$C NMR** (101 MHz, $CDCl_3$) $^{13}$C NMR (101 MHz, $CDCl_3$) δ 220.8, 170.0, 143.4, 139.4, 137.51, 137.47, 133.5, 129.8, 128.5, 127.1, 126.1, 125.8, 51.5, 50.7, 48.1, 47.3, 46.21, 46.17, 44.5, 38.1, 36.0, 31.7, 29.4, 26.5, 26.0, 25.7, 24.2, 21.7, 21.6, 14.0. **HRMS** (ESI) calcd. for ($C_{32}H_{40}N_2NaO_4S$) $[M+Na]^+$: 571.2601, found 571.2622. **IR** (neat): 3175, 2925, 2873, 1737, 1624, 1451, 1326, 1159, 1093, 817, 754, 662, 551. **Optical rotation**: $[\alpha]^{26}_{D}$ = 44.5 (c 0.1, $CHCl_3$, 93.5:6.5 dr). The enantiomeric excess of *epi*-**3s** was determined by SFC analysis on a Chiralpak ID-3 column ($CO_2$/MeOH with a gradient from 100% to 70% $CO_2$ in 5 min, then maintained at 70% $CO_2$, column temperature 35 °C, flow rate 1.2 mL/min) with retention time 6.82 min (minor) and 7.70 min (major).

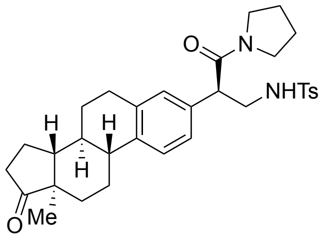


*epi*-**3s**

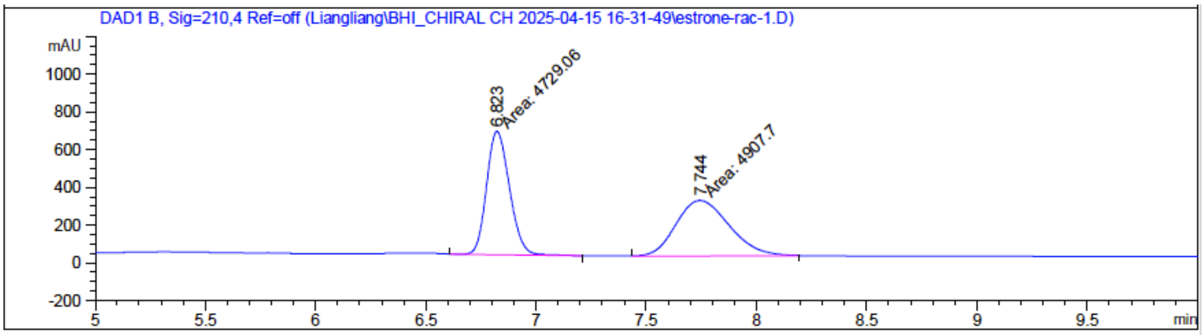


Signal 2: DAD1 B, Sig=210,4 Ref=off

| Peak # | RetTime [min] | Type | Width [min] | Area [mAU*s] | Height [mAU] | Area % |
|---|---|---|---|---|---|---|
| 1 | 6.823 | MM | 0.1204 | 4729.06104 | 654.41962 | 49.0732 |
| 2 | 7.744 | MM | 0.2788 | 4907.69629 | 293.39801 | 50.9268 |

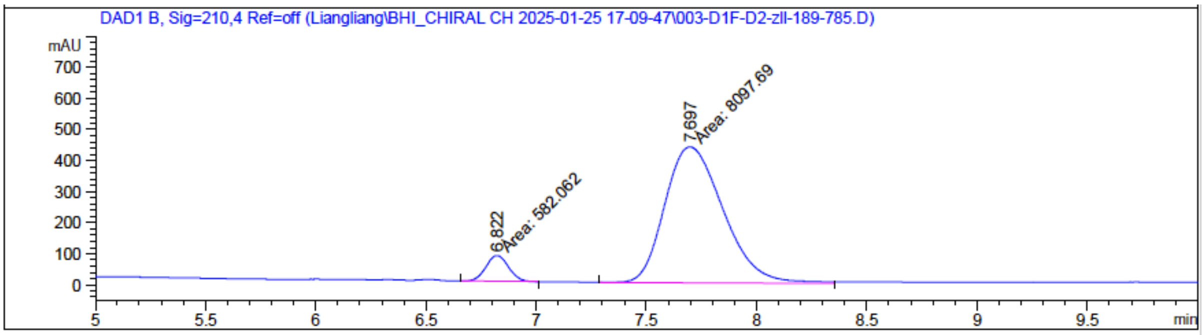


```
Signal 2: DAD1 B, Sig=210,4 Ref=off

Peak RetTime Type  Width     Area      Height     Area
  #   [min]        [min]   [mAU*s]     [mAU]        %
----|-------|----|-------|----------|----------|--------|
   1   6.822 MM    0.1175  582.06201   82.56398   6.7060
   2   7.697 MM    0.3078 8097.69092  438.43970  93.2940
```

**(*S*)-4-(3-((4-methylphenyl)sulfonamido)-1-oxo-1-(pyrrolidin-1-yl)propan-2-yl)benzyl 2-(1-(4-chlorobenzoyl)-5-methoxy-2-methyl-1*H*-indol-3-yl)acetate (3t):** Following General Procedure C, 4-(1-tosylaziridin-2-yl)benzyl 2-(1-(4-chlorobenzoyl)-5-methoxy-2-methyl-1*H*-indol-3-yl)acetate (**1t**) (96.3 mg, 0.15 mmol), and diethyl 2,6-dimethyl-4-(pyrrolidine-1-carbonyl)-1,4-dihydropyridine-3,5-dicarboxylate (**2a**) (105.1mg, 0.30 mmol) were used, affording the title compound as a yellow oil (54.6 mg, 49% yield) by using $^i$PrOAc/Acetone (20:1) as eluent. In an independent experiment, 58.0 mg (52% yield) were obtained, giving an average yield of 51% with 93:7 er. $^1$**H NMR** (400 MHz, $CDCl_3$) δ 7.72 – 7.69 (m, 2H), 7.67 – 7.63 (m, 2H), 7.49 – 7.45 (m, 2H), 7.30 – 7.25 (m, 2H), 7.24 – 7.20 (m, 2H), 7.19 – 7.14 (m, 2H), 6.91 (d, *J* = 2.5 Hz, 1H), 6.85 (d, *J* = 9.0 Hz, 1H), 6.65 (dd, *J* = 9.0, 2.5 Hz, 1H), 5.39 (dd, *J* = 9.0, 4.7 Hz, 1H), 5.09 (s, 2H), 3.89 (dd, *J* = 9.7, 4.8 Hz, 1H), 3.75 (s, 3H), 3.71 (s, 2H), 3.56 – 3.45 (m, 1H), 3.41 – 3.27 (m, 3H), 3.23 – 3.12 (m, 1H), 2.92 – 2.84 (m, 1H), 2.40 (s, 3H), 2.37 (s, 3H), 1.91 – 1.66 (m, 4H). $^{13}$**C NMR** (101 MHz, $CDCl_3$) δ 170.7, 169.7, 168.4, 156.1, 143.4, 139.5, 137.5, 136.3, 136.1, 135.5, 134.0, 131.3, 130.9, 130.7, 129.8, 129.3, 128.8, 128.4, 127.0, 115.1, 112.5, 111.8, 101.5, 66.3, 55.8, 51.6, 47.1, 46.21, 46.20, 30.5, 26.0, 24.2, 21.6, 13.5. **HRMS** (ESI) calcd. for ($C_{40}H_{40}ClN_3NaO_7S$) $[M+Na]^+$: 764.2168, found 764.2193. **IR** (neat): 3203, 2933, 2874, 1736, 1681, 1625, 1477, 1453, 1357, 1323, 1224, 1159, 1090, 1067, 1016, 835, 754, 660, 554. **Optical rotation**: $[\alpha]^{26}_D$ = -0.9 (c 0.2, $CHCl_3$, 93:7 er). The enantiomeric excess of **3t** was determined by SFC analysis on a Chiralpak ID-3 column ($CO_2$/MeOH with a gradient from 100% to 50% $CO_2$ in 5 min, then maintained at 50% $CO_2$, column temperature 35 °C, flow rate 1.2 mL/min) with retention time 15.76 min (major) and 24.62 min (minor).

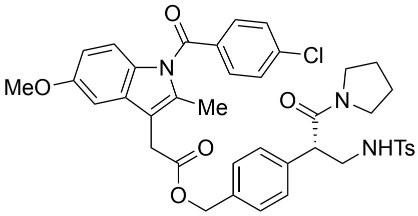


**3t**

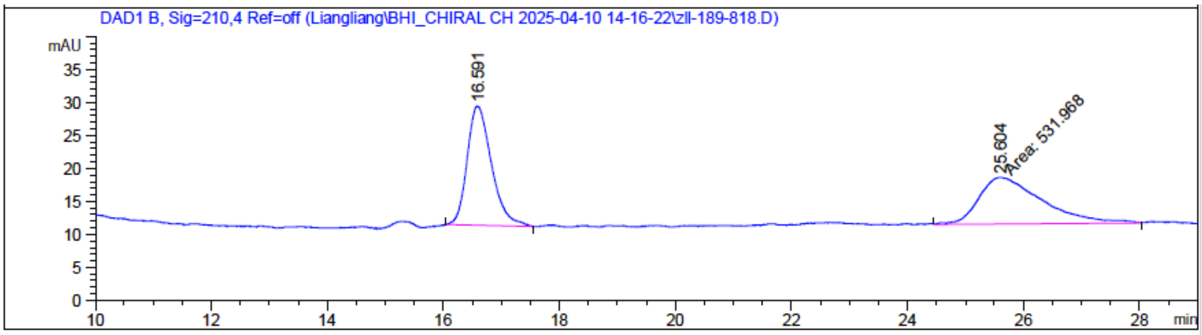


Signal 2: DAD1 B, Sig=210,4 Ref=off

| Peak # | RetTime [min] | Type | Width [min] | Area [mAU*s] | Height [mAU] | Area % |
|---|---|---|---|---|---|---|
| 1 | 16.591 | BV R | 0.3438 | 527.19739 | 18.09905 | 49.7748 |
| 2 | 25.604 | MM | 1.2592 | 531.96759 | 7.04113 | 50.2252 |

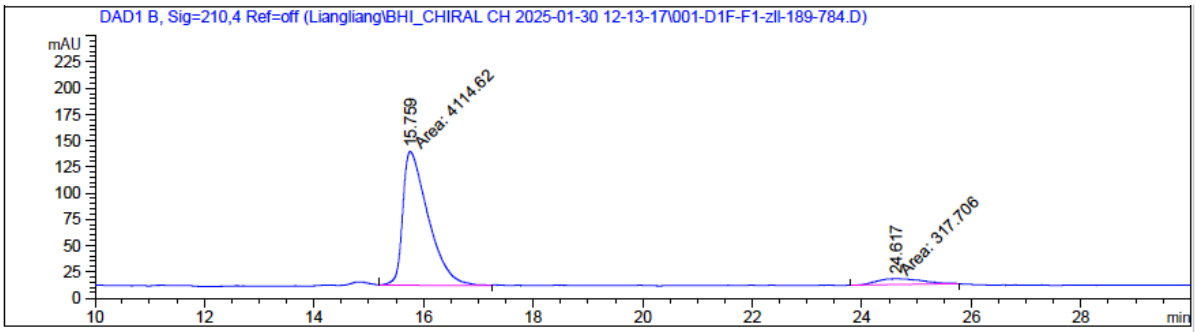


Signal 2: DAD1 B, Sig=210,4 Ref=off

| Peak # | RetTime [min] | Type | Width [min] | Area [mAU*s] | Height [mAU] | Area % |
|---|---|---|---|---|---|---|
| 1 | 15.759 | MM | 0.5380 | 4114.61768 | 127.47490 | 92.8321 |
| 2 | 24.617 | MM | 0.9558 | 317.70615 | 5.53976 | 7.1679 |

**4-((*S*)-3-((4-methylphenyl)sulfonamido)-1-oxo-1-(pyrrolidin-1-yl)propan-2-yl)benzyl (*S*)-2-(6-methoxynaphthalen-2-yl)propanoate (3u):** Following General Procedure C, 4-(1-tosylaziridin-2-yl)benzyl (2*S*)-2-(6-methoxynaphthalen-2-yl)propanoate (**1u**) (77.3 mg, 0.15 mmol), and diethyl 2,6-dimethyl-4-(pyrrolidine-1-carbonyl)-1,4-dihydropyridine-3,5-dicarboxylate (**2a**) (105.1mg, 0.30 mmol) were used, affording the title compound as a yellow oil (55.7 mg, 60% yield) by using $^{i}$PrOAc/Acetone (20:1) as eluent. In an independent experiment, 52.5 mg (57% yield) were obtained, giving an average yield of 59% with 93:7 dr.

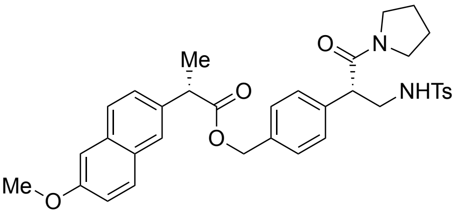


**3u**

**$^{1}$H NMR** (400 MHz, $CDCl_3$) δ 7.72 – 7.64 (m, 5H), 7.38 (dd, *J* = 8.5, 1.9 Hz, 1H), 7.27 – 7.24 (m, 2H), 7.16 – 7.10 (m, 6H), 5.44 (dd, *J* = 9.0, 4.7 Hz, 1H), 5.11 – 5.02 (m, 2H), 3.93 – 3.83 (m, 5H), 3.54 – 3.43 (m, 1H), 3.40 – 3.22 (m, 3H), 3.22 – 3.10 (m, 1H), 2.88 – 2.81 (m, 1H), 2.39 (s, 3H), 1.86 – 1.65 (m, 4H), 1.58 (d, *J* = 7.1 Hz, 3H). **$^{13}$C NMR** (101 MHz, $CDCl_3$) δ 174.5, 169.7, 157.8, 143.3, 137.5, 136.0, 135.7, 135.5,

133.8, 129.8, 129.3, 129.0, 128.5, 128.3, 127.3, 127.0, 126.3, 126.1, 119.1, 105.7, 66.0, 55.4, 51.5, 47.1, 46.2, 45.5, 25.9, 24.1, 21.6, 18.6. **HRMS** (ESI) calcd. for ($C_{35}H_{39}N_2O_6S$) $[M+H]^+$: 615.2523, found 615.2509. **IR** (neat): 3198, 2950, 1733, 1628, 1450, 1327, 1267, 1229, 1159, 1092, 815, 760, 662, 553. **Optical rotation**: $[\alpha]^{26}_{D}$ = -0.5 (c 0.2, $CHCl_3$, 93:7 dr). The enantiomeric excess of **3u** was determined by SFC analysis on a Chiralpak ID-3 column ($CO_2$/MeOH with a gradient from 100% to 70% $CO_2$ in 5 min, then maintained at 70% $CO_2$, column temperature 35 °C, flow rate 1.2 mL/min) with retention time 16.32 min (major) and 24.28 min (minor).

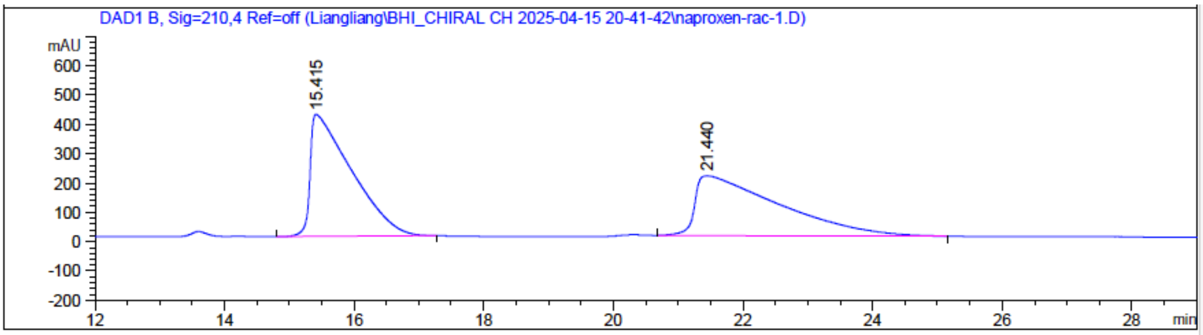


Signal 2: DAD1 B, Sig=210,4 Ref=off

| Peak # | RetTime [min] | Type | Width [min] | Area [mAU*s] | Height [mAU] | Area % |
|---|---|---|---|---|---|---|
| 1 | 15.415 | BV R | 0.5575 | 1.84900e4 | 416.44766 | 50.1928 |
| 2 | 21.440 | BV R | 1.0565 | 1.83479e4 | 203.57065 | 49.8072 |

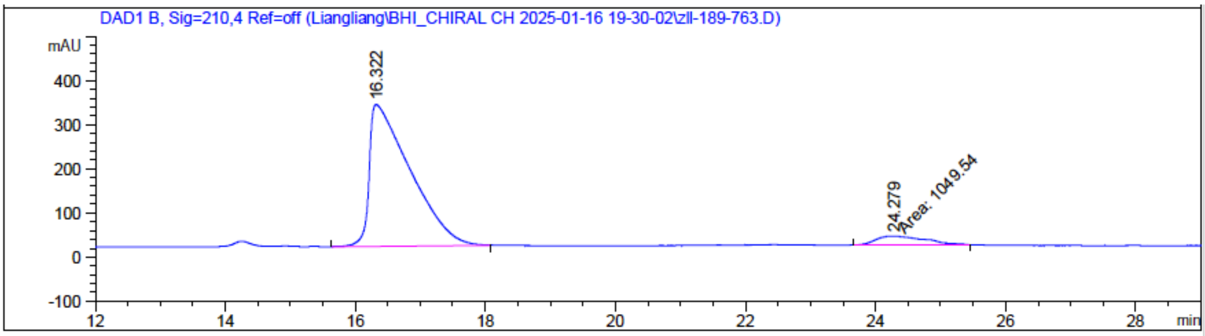


Signal 2: DAD1 B, Sig=210,4 Ref=off

| Peak # | RetTime [min] | Type | Width [min] | Area [mAU*s] | Height [mAU] | Area % |
|---|---|---|---|---|---|---|
| 1 | 16.322 | VV R | 0.5460 | 1.40473e4 | 321.33273 | 93.0480 |
| 2 | 24.279 | MM | 0.8706 | 1049.53638 | 20.09160 | 6.9520 |

**4-((*R*)-3-((4-methylphenyl)sulfonamido)-1-oxo-1-(pyrrolidin-1-yl)propan-2-yl)benzyl (*S*)-2-(6-methoxynaphthalen-2-yl)propanoate (*epi*-3u):** Following General Procedure C, 4-(1-tosylaziridin-2-yl)benzyl (2*S*)-2-(6-methoxynaphthalen-2-yl)propanoate (**1u**) (77.3 mg, 0.15 mmol), and diethyl 2,6-dimethyl-4-(pyrrolidine-1-carbonyl)-1,4-dihydropyridine-3,5-dicarboxylate (**2a**) (105.1mg, 0.30 mmol) and *ent*-**L1** (3a*S*,3a'*S*,8a*R*,8a'*R*)-2,2'-(1,3-bis(4-(*tert*-

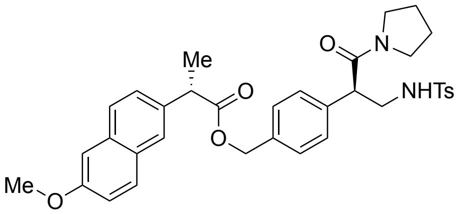


*epi*-**3u**

butyl)phenyl)propane-2,2-diyl)bis(3a,8a-dihydro-8*H*-indeno[1,2-*d*]oxazole) (14.0 mg, 0.0225 mol) were used, affording the title compound as a yellow oil (48.5 mg, 53% yield) by using $^{i}$PrOAc/Acetone (20:1) as eluent. In an independent experiment, 51.7 mg (56% yield) were obtained, giving an average yield of 54% with 92.5:7.5 dr. **$^{1}$H NMR** (400 MHz, $CDCl_3$) δ 7.71 – 7.64 (m, 5H), 7.38 (dd, *J* = 8.5, 1.9 Hz, 1H), 7.29 – 7.22 (m, 2H), 7.16 – 7.09 (m, 6H), 5.38 (dd, *J* = 9.1, 4.6 Hz, 1H), 5.11 – 5.03 (m, 2H), 3.93 – 3.83 (m, 5H), 3.54 – 3.44 (m, 1H), 3.40 – 3.23 (m, 3H), 3.21 – 3.10 (m, 1H), 2.88 – 2.82 (m, 1H), 2.39 (s, 3H), 1.85 – 1.67 (m, 4H), 1.59 (d, *J* = 7.1 Hz, 3H). **$^{13}$C NMR** (101 MHz, $CDCl_3$) δ 174.5, 169.7, 157.8, 143.4, 137.5, 136.0, 135.7, 135.5, 133.9, 129.8, 129.3, 129.0, 128.5, 128.3, 127.3, 127.0, 126.3, 126.1, 119.2, 105.8, 66.0, 55.4, 51.6, 47.1, 46.2, 45.6, 25.9, 24.1, 21.6, 18.6. **HRMS** (ESI) calcd. for ($C_{35}H_{39}N_2O_6S$) $[M+H]^+$: 615.2523, found 615.2530. **IR** (neat): 3208, 2972, 1732, 1627, 1452, 1330, 1265, 1217, 1159, 1093, 1031, 815, 755, 665, 550. **Optical rotation**: $[\alpha]^{26}_{D}$ = -7.7 (c 0.2, $CHCl_3$, 92.5:7.5 dr). The enantiomeric excess of *epi*-**3u** was determined by SFC analysis on a Chiralpak ID-3 column ($CO_2$/MeOH with a gradient from 100% to 70% $CO_2$ in 5 min, then maintained at 70% $CO_2$, column temperature 35 °C, flow rate 1.2 mL/min) with retention time 16.55 min (minor) and 22.59 min (major).

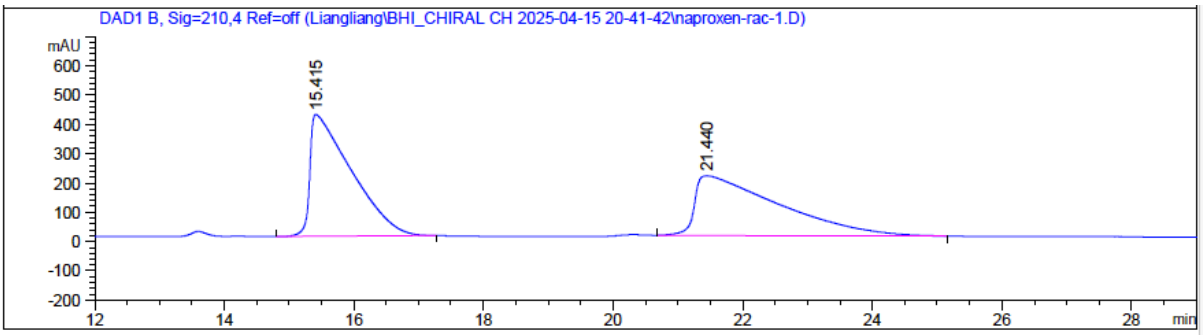


Signal 2: DAD1 B, Sig=210,4 Ref=off

| Peak # | RetTime [min] | Type | Width [min] | Area [mAU*s] | Height [mAU] | Area % |
|---|---|---|---|---|---|---|
| 1 | 15.415 | BV R | 0.5575 | 1.84900e4 | 416.44766 | 50.1928 |
| 2 | 21.440 | BV R | 1.0565 | 1.83479e4 | 203.57065 | 49.8072 |

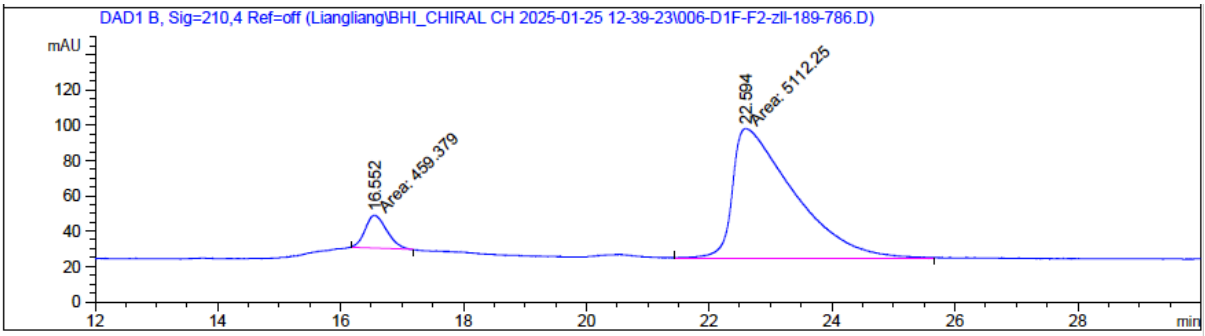

Signal 2: DAD1 B, Sig=210,4 Ref=off

| Peak # | RetTime [min] | Type | Width [min] | Area [mAU*s] | Height [mAU] | Area % |
|---|---|---|---|---|---|---|
| 1 | 16.552 | MM | 0.4106 | 459.37885 | 18.64509 | 8.2450 |
| 2 | 22.594 | MM | 1.1590 | 5112.25000 | 73.51508 | 91.7550 |

**4-((*S*)-3-((4-methylphenyl)sulfonamido)-1-oxo-1-(pyrrolidin-1-yl)propan-2-yl)benzyl (*S*)-2-(4-isobutylphenyl)propanoate (3v):** Following General Procedure C, 4-(1-tosylaziridin-2-yl)benzyl (2*S*)-2-(4-isobutylphenyl)propanoate (**1v**) (73.7 mg, 0.15 mmol), and diethyl 2,6-dimethyl-4-(pyrrolidine-1-carbonyl)-1,4-dihydropyridine-3,5-dicarboxylate (**2a**) (105.1mg, 0.30 mmol) were used, affording the title compound as a yellow oil (51.9 mg, 59% yield) by using *i*PrOAc/Acetone (20:1) as eluent. In an independent experiment, 48.1 mg (54% yield) were obtained, giving an average yield of 57% with 92:8 dr. $^1$**H NMR** (400 MHz, $CDCl_3$) δ 7.72 – 7.68 (m, 2H), 7.29 – 7.23 (m, 2H), 7.21 – 7.16 (m, 2H), 7.18 – 7.11 (m, 4H), 7.10 – 7.06 (m, 2H), 5.46 (dd, $J$ = 9.0, 4.7 Hz, 1H), 5.10 – 5.01 (m, 2H), 3.91 – 3.83 (m, 1H), 3.79 – 3.69 (m, 1H), 3.55 – 3.44 (m, 1H), 3.41 – 3.25 (m, 3H), 3.22 – 3.10 (m, 1H), 2.93 – 2.82 (m, 1H), 2.45 (d, $J$ = 7.2 Hz, 2H), 2.39 (s, 3H), 1.90 – 1.68 (m, 5H), 1.50 (d, $J$ = 7.2 Hz, 3H), 0.89 (d, $J$ = 6.6 Hz, 6H). $^{13}$**C NMR** (101 MHz, $CDCl_3$) δ 174.6, 169.7, 143.3, 140.7, 137.6, 137.5, 135.9, 135.8, 129.8, 129.4, 128.33, 128.30, 127.3, 127.0, 65.8, 51.5, 47.1, 46.2, 45.2, 45.1, 30.3, 25.9, 24.1, 22.5, 21.6, 18.5. **HRMS** (ESI) calcd. for ($C_{34}H_{43}N_2O_5S$) $[M+H]^+$: 591.2887, found 591.2874. **IR** (neat): 3201, 2954, 2871, 1735, 1626, 1450, 1331, 1159, 1093, 816, 660, 552. **Optical rotation**: $[\alpha]^{26}_D$ = 9.6 (c 0.7, $CHCl_3$, 92:8 dr). The enantiomeric excess of **3v** was determined by SFC analysis on a Chiralpak ID-3 column ($CO_2$/MeOH with a gradient from 100% to 70% $CO_2$ in 5 min, then maintained at 70% $CO_2$, column temperature 35 °C, flow rate 1.2 mL/min) with retention time 6.19 min (major) and 7.29 min (minor).

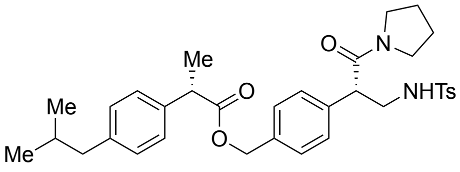


**3v**

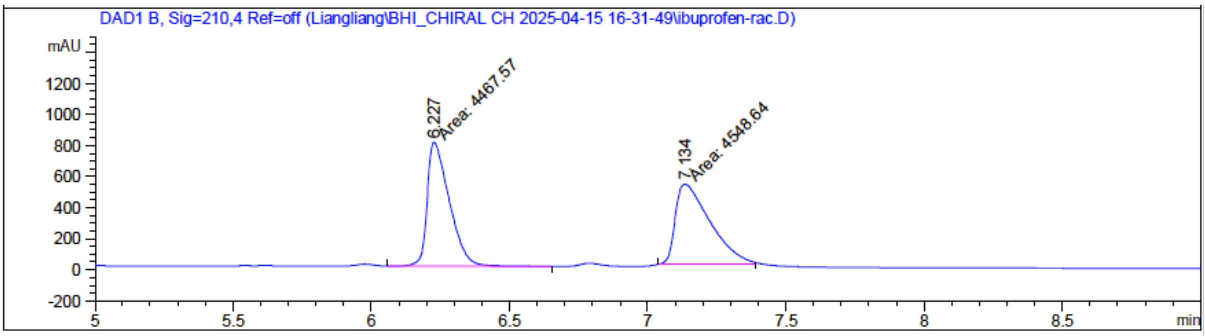


Signal 2: DAD1 B, Sig=210,4 Ref=off

| Peak # | RetTime [min] | Type | Width [min] | Area [mAU*s] | Height [mAU] | Area % |
|---|---|---|---|---|---|---|
| 1 | 6.227 | MM | 0.0933 | 4467.57129 | 798.04266 | 49.5504 |
| 2 | 7.134 | MM | 0.1465 | 4548.64111 | 517.45154 | 50.4496 |

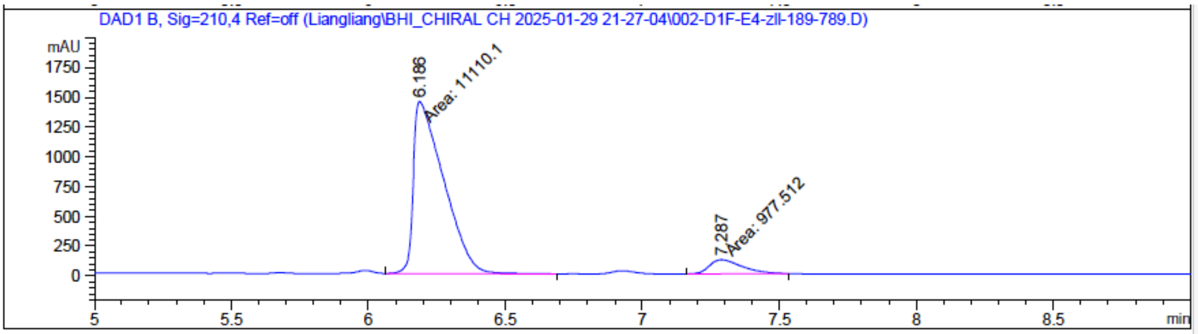


Signal 2: DAD1 B, Sig=210,4 Ref=off

| Peak # | RetTime [min] | Type | Width [min] | Area [mAU*s] | Height [mAU] | Area % |
|---|---|---|---|---|---|---|
| 1 | 6.186 | FM | 0.1283 | 1.11101e4 | 1443.44128 | 91.9131 |
| 2 | 7.287 | MM | 0.1383 | 977.51227 | 117.80498 | 8.0869 |

**4-((*S*)-3-((4-methylphenyl)sulfonamido)-1-oxo-1-(pyrrolidin-1-yl)propan-2-yl)benzyl (*S*)-2-(4-isobutylphenyl)propanoate (*epi*-3v):** Following General Procedure C, 4-(1-tosylaziridin-2-yl)benzyl (2*S*)-2-(4-isobutylphenyl)propanoate (**1v**) (73.7 mg, 0.15 mmol), and diethyl 2,6-dimethyl-4-(pyrrolidine-1-carbonyl)-1,4-dihydropyridine-3,5-dicarboxylate (**2a**) (105.1mg, 0.30 mmol) and *ent*-**L1** (3a*S*,3a'*S*,8a*R*,8a'*R*)-2,2'-(1,3-bis(4-(*tert*-butyl)phenyl)propane-2,2-diyl)bis(3a,8a-dihydro-8*H*-indeno[1,2-*d*]oxazole) (14.0 mg, 0.0225 mol) were used, affording the title compound as a yellow oil (50.6 mg, 57% yield) by using $^{i}$PrOAc/Acetone (20:1) as eluent. In an independent experiment, 49.0 mg (55% yield) were obtained, giving an average yield of 56% with 91:9 dr.

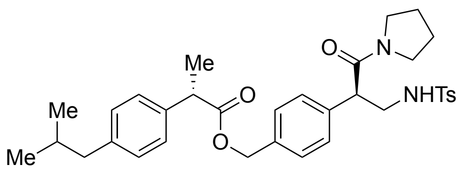


*epi*-**3v**

**$^{1}$H NMR** (400 MHz, $CDCl_3$) δ 7.72 – 7.68 (m, 2H), 7.30 – 7.23 (m, 2H), 7.21 – 7.17 (m, 2H), 7.18 – 7.09 (m, 4H), 7.10 – 7.06 (m, 2H), 5.37 (dd, *J* = 9.1, 4.6 Hz, 1H), 5.05 (d, *J* = 2.9 Hz, 2H), 3.91 – 3.83 (m, 1H), 3.79 – 3.69 (m, 1H), 3.55 – 3.45 (m, 1H), 3.41 – 3.25 (m, 3H), 3.22 – 3.11 (m, 1H), 2.93 – 2.82 (m, 1H), 2.45 (d, *J* = 7.2 Hz, 2H), 2.40 (s, 3H), 1.90 – 1.69 (m, 5H), 1.50 (d, *J* = 7.2 Hz, 3H), 0.89 (d, *J* = 6.6 Hz, 6H). **$^{13}$C NMR** (101 MHz, $CDCl_3$) δ 174.6, 169.7, 143.4, 140.8, 137.6, 137.5, 135.90, 135.85, 129.8, 129.5, 128.4, 128.3, 127.3, 127.1, 65.8, 51.6, 47.2, 46.2, 45.2, 45.1, 30.3, 26.0, 24.2, 22.5, 21.6, 18.6. **HRMS** (ESI) calcd. for ($C_{34}H_{43}N_2O_5S$) $[M+H]^+$: 591.2887, found 591.2890. **IR** (neat): 2955, 2871, 1735, 1627, 1451, 1330, 1160, 1093. **Optical rotation**: $[\alpha]^{26}_{D}$ = -4.4 (c 0.3, $CHCl_3$, 91:9 dr). The enantiomeric excess of *epi*-**3v** was determined by SFC analysis on a Chiralpak ID-3 column ($CO_2$/MeOH with a gradient from 100% to 70% $CO_2$ in 5 min, then maintained at 70% $CO_2$, column temperature 35 °C, flow rate 1.2 mL/min) with retention time 6.32 min (minor) and 7.20 min (major).

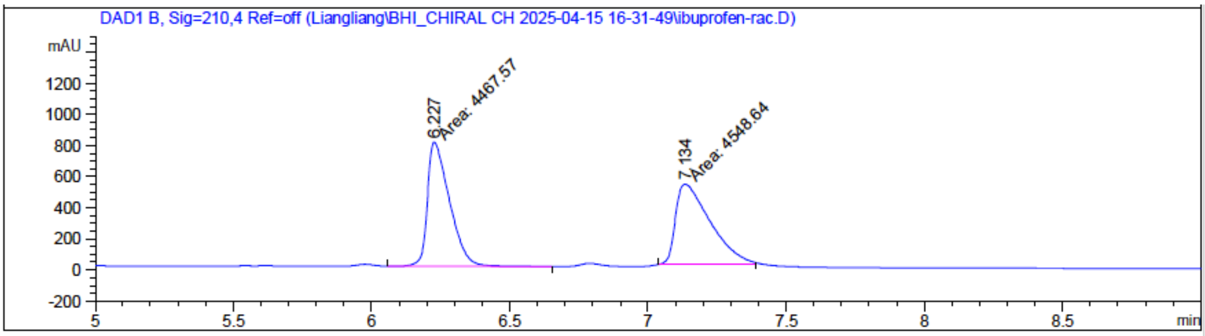


Signal 2: DAD1 B, Sig=210,4 Ref=off

| Peak # | RetTime [min] | Type | Width [min] | Area [mAU*s] | Height [mAU] | Area % |
|---|---|---|---|---|---|---|
| 1 | 6.227 | MM | 0.0933 | 4467.57129 | 798.04266 | 49.5504 |
| 2 | 7.134 | MM | 0.1465 | 4548.64111 | 517.45154 | 50.4496 |

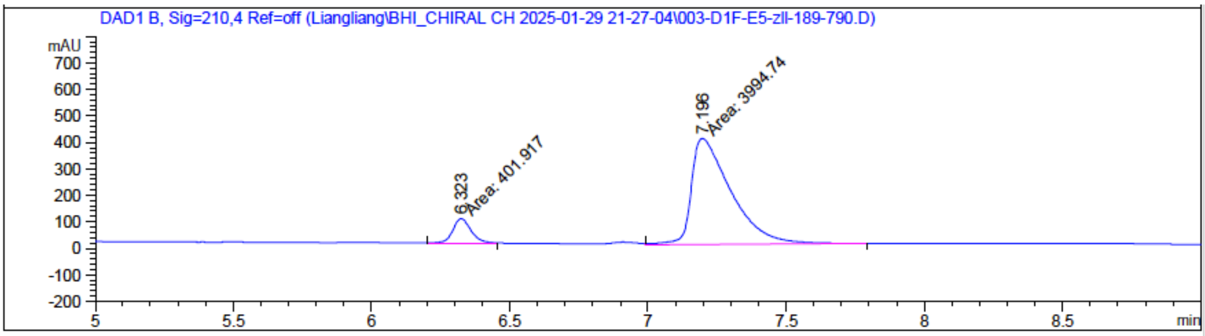


Signal 2: DAD1 B, Sig=210,4 Ref=off

| Peak # | RetTime [min] | Type | Width [min] | Area [mAU*s] | Height [mAU] | Area % |
|---|---|---|---|---|---|---|
| 1 | 6.323 | MM | 0.0736 | 401.91684 | 91.01128 | 9.1414 |
| 2 | 7.196 | FM | 0.1664 | 3994.73535 | 400.06198 | 90.8586 |

**(*S*)-*N*,*N*-dimethyl-3-((4-methylphenyl)sulfonamido)-2-(*p*-tolyl)propanamide (4a):** Following General Procedure C, 2-(*p*-tolyl)-1-tosylaziridine (**1a**) (43.1 mg, 0.15 mmol), and diethyl 4-(dimethylcarbamoyl)-2,6-dimethyl-1,4-dihydropyridine-3,5-dicarboxylate (**2b**) (97.3 mg, 0.30 mmol) were used, affording the title compound as a yellow oil (33.8 mg, 63% yield) by using $^{i}$PrOAc/Acetone (20:1) as eluent. In an independent experiment, 36.0 mg (67% yield) were obtained, giving an average yield of 65% with 94.5:5.5 er. **$^{1}$H NMR** (400 MHz, $CDCl_3$) δ 7.71 – 7.67 (m, 2H), 7.29 – 7.23 (m, 2H), 7.11 (d, $J$ = 8.1 Hz, 2H), 7.05 (d, $J$ = 8.1 Hz, 2H), 5.28 (dd, $J$ = 9.2, 4.6 Hz, 1H), 3.97 (dd, $J$ = 9.7, 4.7 Hz, 1H), 3.34 – 3.23 (m, 1H), 3.23 – 3.12 (m, 1H), 2.92 (s, 3H), 2.74 (s, 3H), 2.40 (s, 3H), 2.30 (s, 3H). **$^{13}$C NMR** (101 MHz, $CDCl_3$) δ 171.7, 143.4, 137.6, 137.5, 133.3, 123.0, 129.8, 127.9, 127.1, 50.2, 47.5, 37.1, 35.9, 21.6, 21.2. **HRMS** (ESI) calcd. for ($C_{19}H_{24}N_2NaO_3S$) $[M+Na]^+$: 383.1400, found 383.1407. **IR** (neat): 3208, 2925, 2872, 1631, 1495, 1400, 1329, 1159, 816, 661, 557. **Optical rotation**: $[\alpha]^{26}_{D}$ = 10.0 (c 0.3, $CHCl_3$, 94.5:5.5 er). The enantiomeric excess of **4a** was determined by

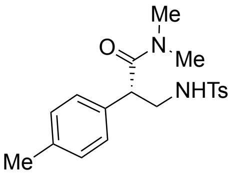


**4a**

SFC analysis on a Chiralpak ID-3 column ($CO_2$/MeOH with a gradient from 100% to 70% $CO_2$ in 5 min, then maintained at 70% $CO_2$, column temperature 35 °C, flow rate 1.2 mL/min) with retention time 5.70 min (major) and 7.57 min (minor).

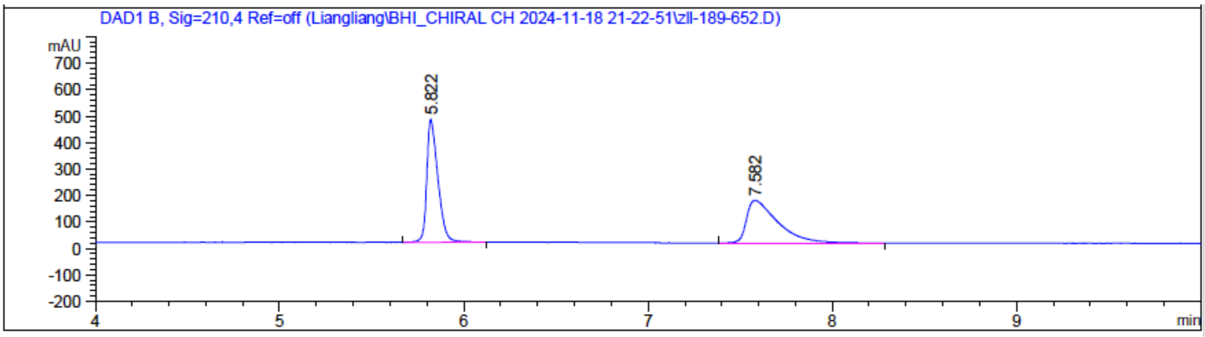


Signal 2: DAD1 B, Sig=210,4 Ref=off

| Peak # | RetTime [min] | Type | Width [min] | Area [mAU*s] | Height [mAU] | Area % |
|---|---|---|---|---|---|---|
| 1 | 5.822 | VV R | 0.0626 | 1951.96545 | 463.88635 | 50.7465 |
| 2 | 7.582 | BV R | 0.1664 | 1894.53674 | 160.84697 | 49.2535 |

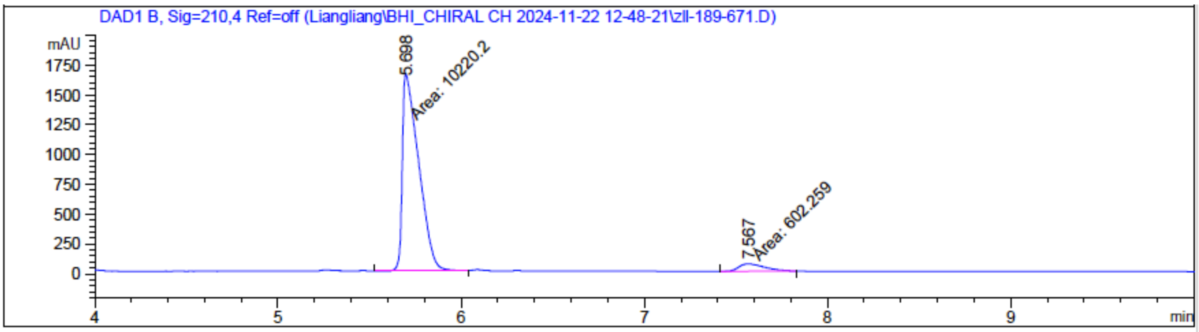


Signal 2: DAD1 B, Sig=210,4 Ref=off

| Peak # | RetTime [min] | Type | Width [min] | Area [mAU*s] | Height [mAU] | Area % |
|---|---|---|---|---|---|---|
| 1 | 5.698 | MM | 0.1035 | 1.02202e4 | 1645.78442 | 94.4351 |
| 2 | 7.567 | MM | 0.1626 | 602.25861 | 61.72256 | 5.5649 |

**(*S*)-*N*-(3-(azetidin-1-yl)-3-oxo-2-(*p*-tolyl)propyl)-4-methylbenzenesulfonamide (4b):** Following General Procedure C, 2-(p-tolyl)-1-tosylaziridine (**1a**) (43.1 mg, 0.15 mmol), and diethyl 4-(azetidine-1-carbonyl)-2,6-dimethyl-1,4-dihydropyridine-3,5-dicarboxylate (**2c**) (75.7mg, 0.225 mmol) were

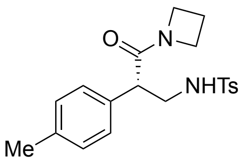


**4b**

used, affording the title compound as a yellow oil (25.6 mg, 46% yield) by two-step column chromatography using $^i$PrOAc/Acetone (20:1), then Hexane/Acetone (2:1) as eluents. In an independent experiment, 25.0 mg (45% yield) were obtained, giving an average yield of 45% with 97:3 er. **$^1$H NMR** (400 MHz, $CDCl_3$) δ 7.71 – 7.68 (m, 2H), 7.27 (d, *J* = 9.1 Hz, 2H), 7.14 – 7.03 (m, 4H), 5.26 (dd, *J* = 8.8, 4.8 Hz, 1H), 4.06 – 3.98 (m, 2H), 3.98 – 3.87 (m, 1H), 3.70 – 3.56 (m, 2H), 3.36 – 3.24 (m, 1H), 3.21 – 3.10 (m, 1H), 2.40 (s, 3H), 2.31 (s, 3H), 2.27 – 2.00 (m, 2H). **$^{13}$C NMR**

(101 MHz, $CDCl_3$) δ 171.2, 143.4, 137.7, 137.4, 132.9, 129.9, 129.8, 128.1, 127.1, 50.3, 48.6, 48.3, 46.5, 21.6, 21.2, 15.4. **HRMS** (ESI) calcd. for ($C_{20}H_{25}N_2O_3S$) $[M+H]^+$: 373.1580, found 373.1579. **IR** (neat): 3183, 2953, 2882, 1631, 1442, 1328, 1159, 1094, 816, 753, 661, 553. **Optical rotation**: $[\alpha]^{26}_D$ = 5.4 (c 0.1, $CHCl_3$, 97:3 er). The enantiomeric excess of **4b** was determined by SFC analysis on a Chiralpak ID-3 column ($CO_2$/MeOH with a gradient from 100% to 70% $CO_2$ in 5 min, then maintained at 70% $CO_2$, column temperature 35 °C, flow rate 1.2 mL/min) with retention time 6.80 min (major) and 9.45 min (minor).

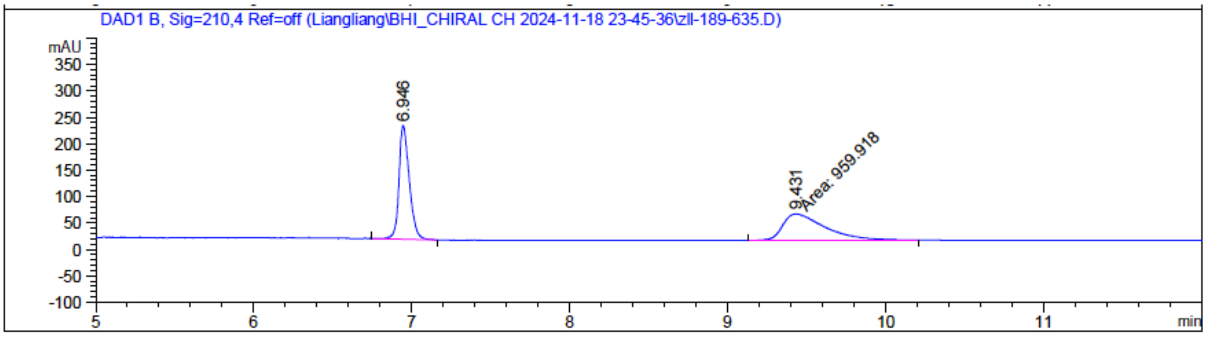


Signal 2: DAD1 B, Sig=210,4 Ref=off

| Peak # | RetTime [min] | Type | Width [min] | Area [mAU*s] | Height [mAU] | Area % |
|---|---|---|---|---|---|---|
| 1 | 6.946 | VB R | 0.0681 | 980.11902 | 215.33945 | 50.5206 |
| 2 | 9.431 | MM | 0.3188 | 959.91772 | 50.18590 | 49.4794 |

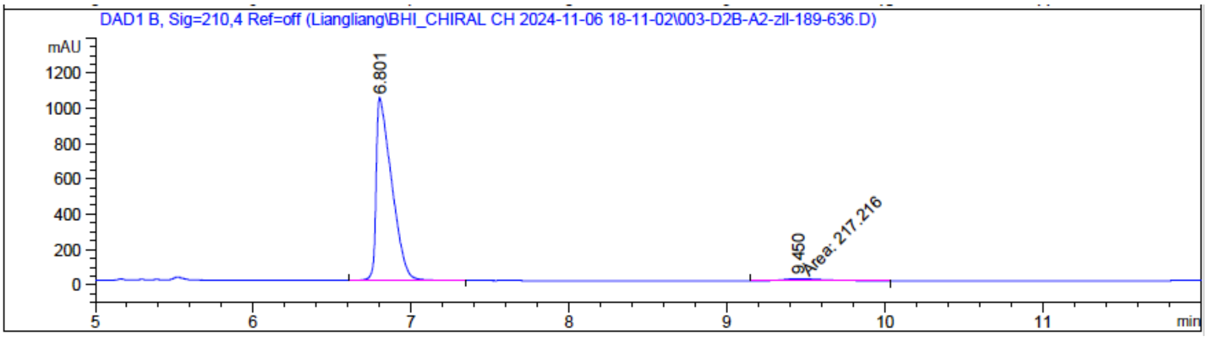


Signal 2: DAD1 B, Sig=210,4 Ref=off

| Peak # | RetTime [min] | Type | Width [min] | Area [mAU*s] | Height [mAU] | Area % |
|---|---|---|---|---|---|---|
| 1 | 6.801 | VV R | 0.0978 | 7080.42139 | 1035.29614 | 97.0235 |
| 2 | 9.450 | MM | 0.3030 | 217.21555 | 11.94796 | 2.9765 |

**(*S*)-*N*-(3-(3,3-dimethylazetidin-1-yl)-3-oxo-2-(*p*-tolyl)propyl)-4-methylbenzenesulfonamide (4c):**

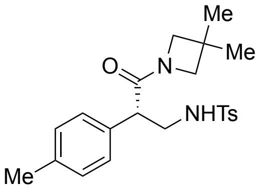


**4c**

Following General Procedure C, 2-(p-tolyl)-1-tosylaziridine (**1a**) (43.1 mg, 0.15 mmol), and diethyl 4-(3,3-dimethylazetidine-1-carbonyl)-2,6-dimethyl-1,4-dihydropyridine-3,5-dicarboxylate (**2d**) (82.0 mg, 0.225 mmol) were used, affording the title compound as a yellow oil (22.6 mg, 38% yield) by two-step column chromatography using $^i$PrOAc/Acetone (20:1), then Hexane/Acetone

(2:1) as eluents. In an independent experiment, 25.8 mg (43% yield) were obtained, giving an average yield of 40% with 96:4 er. **$^1$H NMR** (400 MHz, $CDCl_3$) δ 7.71 – 7.68 (m, 2H), 7.30 – 7.24 (m, 2H), 7.13 – 7.02 (m, 4H), 5.25 (dd, $J$ = 8.8, 4.8 Hz, 1H), 3.70 – 3.62 (m, 3H), 3.63 – 3.56 (m, 1H), 3.35 – 3.23 (m, 1H), 3.23 – 3.13 (m, 2H), 2.40 (s, 3H), 2.31 (s, 3H), 1.24 (s, 3H), 1.05 (s, 3H). **$^{13}$C NMR** (101 MHz, $CDCl_3$) δ 171.7, 143.4, 137.7, 137.4, 133.0, 129.9, 129.8, 128.0, 127.1, 62.3, 60.3, 48.8, 46.6, 31.3, 27.0, 26.9, 21.6, 21.2. **HRMS** (ESI) calcd. for ($C_{22}H_{28}N_2NaO_3S$) $[M+Na]^+$: 4423.1713, found 423.1707. **IR** (neat): 3186, 2956, 2873, 1634, 1455, 1331, 1160, 1094, 816, 661, 551. **Optical rotation**: $[\alpha]^{26}{}_{D}$ = 20.3 (c 0.1, $CHCl_3$, 96:4 er). The enantiomeric excess of **4c** was determined by SFC analysis on a Chiralpak ID-3 column ($CO_2$/MeOH with a gradient from 100% to 70% $CO_2$ in 5 min, then maintained at 70% $CO_2$, column temperature 35 °C, flow rate 1.2 mL/min) with retention time 5.91 min (major) and 7.02 min (minor).

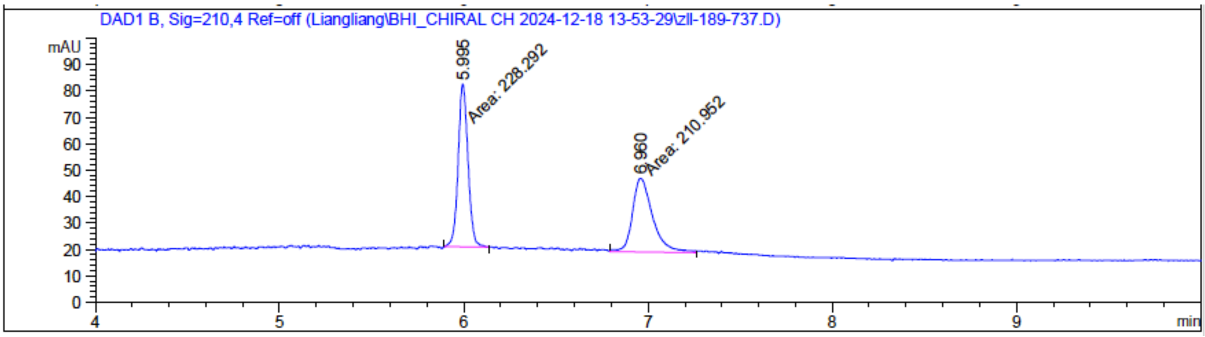


Signal 2: DAD1 B, Sig=210,4 Ref=off

| Peak # | RetTime [min] | Type | Width [min] | Area [mAU*s] | Height [mAU] | Area % |
|---|---|---|---|---|---|---|
| 1 | 5.995 | MM | 0.0618 | 228.29210 | 61.57153 | 51.9738 |
| 2 | 6.960 | MM | 0.1263 | 210.95230 | 27.83033 | 48.0262 |

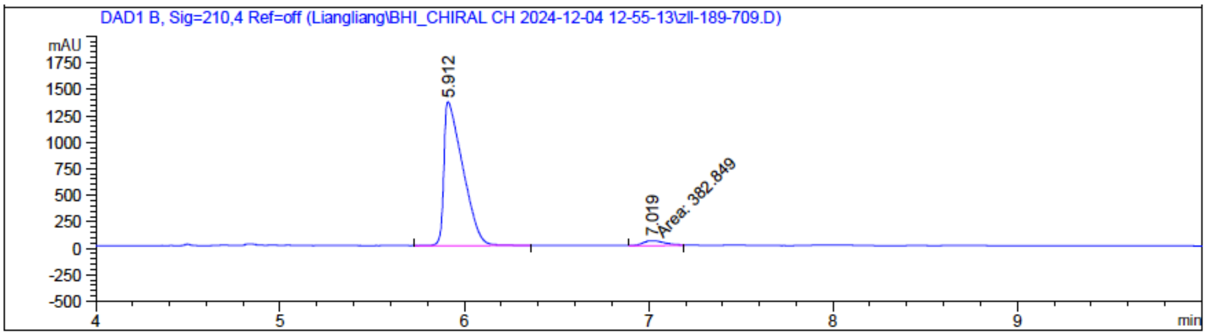


Signal 2: DAD1 B, Sig=210,4 Ref=off

| Peak # | RetTime [min] | Type | Width [min] | Area [mAU*s] | Height [mAU] | Area % |
|---|---|---|---|---|---|---|
| 1 | 5.912 | VV R | 0.1050 | 9656.68945 | 1357.03271 | 96.1866 |
| 2 | 7.019 | MM | 0.1374 | 382.84897 | 46.44598 | 3.8134 |

**(*S*)-*N*-(3-(3-methoxyazetidin-1-yl)-3-oxo-2-(*p*-tolyl)propyl)-4-methylbenzenesulfonamide (4d):** Following General Procedure C, 2-(p-tolyl)-1-tosylaziridine (**1a**) (43.1 mg, 0.15 mmol), and diethyl 4-(3-methoxyazetidine-1-carbonyl)-2,6-dimethyl-1,4-dihydropyridine-3,5-dicarboxylate (**2e**) (82.4 mg, 0.225 mmol) were used, affording the title compound as a colorless oil (16.0 mg, 27% yield) two-step column chromatography using $^i$PrOAc/Acetone (20:1), then Hexane/acetone (2:1) as eluents. In an independent experiment, 14.6 mg (24% yield) were obtained, giving an average yield of 25% with 95.5:4.5 er. **$^1$H NMR** (400 MHz, $CDCl_3$) δ 7.72 – 7.67 (m, 2H), 7.30 – 7.27 (m, 2H), 7.11 (d, $J$ = 7.9 Hz, 2H), 7.08 – 7.01 (m, 2H), 5.25 – 5.16 (m, 1H), 4.21 – 4.12 (m, 1.5H), 4.09 – 4.04 (m, 0.5H), 4.02 – 3.92 (m, 0.5H), 3.90 – 3.57 (m, 3H), 3.45 (q, $J$ = 6.4 Hz, 0.5H), 3.35 – 3.11 (m, 5H), 2.41 (s, 3H), 2.31 (s, 3H). **$^{13}$C NMR** (101 MHz, $CDCl_3$) δ 143.5, 137.83, 137.81, 137.5, 137.3, 132.8, 132.6, 130.0, 129.93, 129.88, 129.85, 128.04, 128.00, 127.12, 127.09, 69.0, 68.9, 57.4, 57.1, 56.4, 56.3, 55.3, 49.3, 48.8, 46.6, 46.5, 21.6, 21.2. **$^1$H NMR** (500 MHz, DMSO-$d_6$, 393K) δ 7.67 – 7.63 (m, 2H), 7.37 – 7.34 (m, 2H), 7.12 – 7.06 (m, 5H), 4.20 – 3.90 (m, 3H), 3.68 – 3.57 (m, 2H), 3.40 – 3.31 (m, 1H), 3.19 (s, 3H), 3.00 – 2.91 (m, 2H), 2.40 (s, 3H), 2.28 (s, 3H). **$^{13}$C NMR** (126 MHz, DMSO-$d_6$, 393K) δ 170.3, 141.9, 137.6, 135.8, 133.5, 128.8, 128.5, 127.2, 125.9, 67.9, 54.7, 46.4, 45.2, 20.2, 19.8. **Note:** At 393 K, the rotameric signals coalesce and the alkyl carbon adjacent to the amide nitrogen is broadened beyond detection. **HRMS** (ESI) calcd. for ($C_{21}H_{26}N_2NaO_4S$) $[M+Na]^+$: 425.1505, found 425.1517. **IR** (neat): 3194, 2926, 1636, 1513, 1457, 1330, 1223, 1159, 1093, 817, 552. **Optical rotation**: $[\alpha]^{26}{}_D$ = 11.7 (c 0.2, $CHCl_3$, 95.5:4.5 er). The enantiomeric excess of **4d** was determined by SFC analysis on a Chiralpak ID-3 column ($CO_2$/MeOH with a gradient from 100% to 70% $CO_2$ in 5 min, then maintained at 70% $CO_2$, column temperature 35 °C, flow rate 1.2 mL/min) with retention time 7.16 min (major) and 8.41 min (minor).

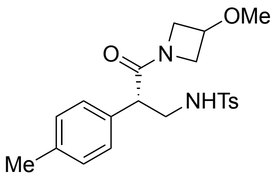


4d

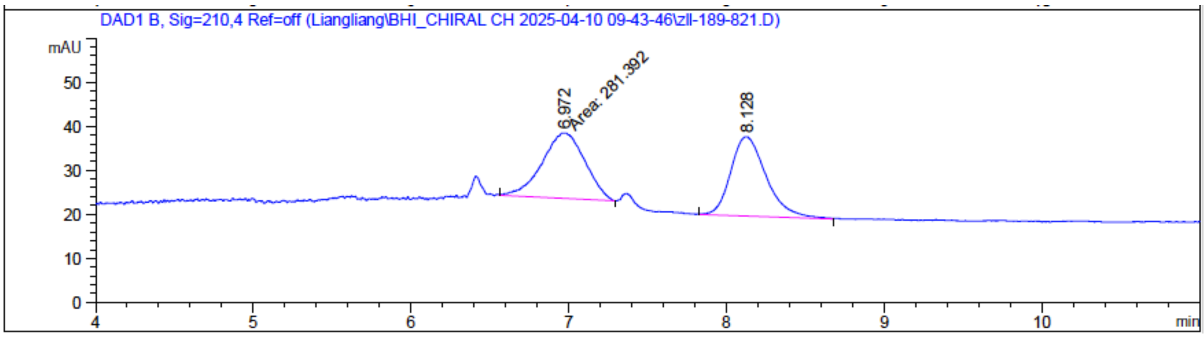


Signal 2: DAD1 B, Sig=210,4 Ref=off

| Peak # | RetTime [min] | Type | Width [min] | Area [mAU*s] | Height [mAU] | Area % |
|---|---|---|---|---|---|---|
| 1 | 6.972 | MM | 0.3158 | 281.39157 | 14.85118 | 50.1614 |
| 2 | 8.128 | BB | 0.1965 | 279.58075 | 18.02374 | 49.8386 |

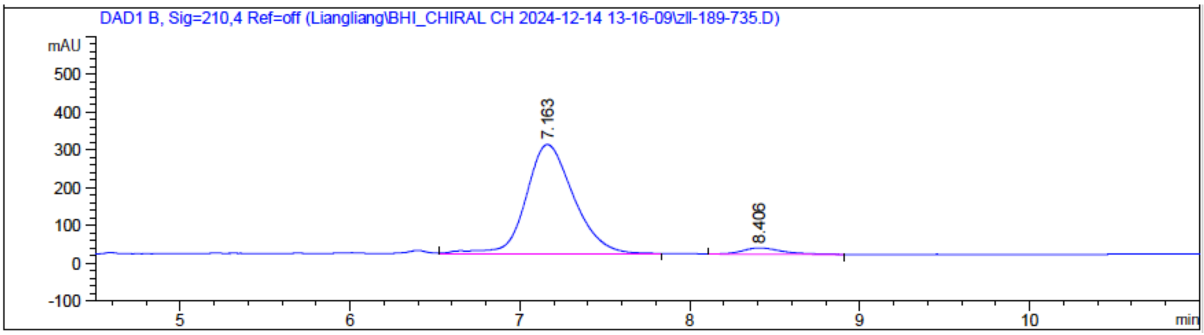


Signal 2: DAD1 B, Sig=210,4 Ref=off

| Peak # | RetTime [min] | Type | Width [min] | Area [mAU*s] | Height [mAU] | Area % |
|---|---|---|---|---|---|---|
| 1 | 7.163 | VB R | 0.2812 | 5599.28564 | 288.89499 | 95.4284 |
| 2 | 8.406 | VV R | 0.1950 | 268.23828 | 16.55727 | 4.5716 |

**methyl (*S*)-1-(3-((4-methylphenyl)sulfonamido)-2-(*p*-tolyl)propanoyl)azetidine-3-carboxylate (4e):** Following General Procedure C, 2-(p-tolyl)-1-tosylaziridine (**1a**) (43.1 mg, 0.15 mmol), and diethyl 4-(3-(methoxycarbonyl)azetidine-1-carbonyl)-2,6-dimethyl-1,4-dihydropyridine-3,5-dicarboxylate (**2f**) (88.7 mg, 0.225 mmol) were used, affording the title compound as a colorless oil (23.2 mg, 36% yield) by two-step column chromatography using $^{i}$PrOAc/Acetone (20:1), then Hexane/Acetone (2:1) as eluents. In an independent experiment, 21.8 mg (34% yield) were obtained, giving an average yield of 35% with 95.5:4.5 er.

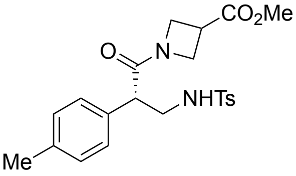


**4e**

$^{1}$**H NMR** (400 MHz, $CDCl_3$) δ 7.73 – 7.66 (m, 2H), 7.30 – 7.26 (m, 2H), 7.11 (d, $J$ = 7.9 Hz, 2H), 7.08 – 7.01 (m, 2H), 5.20 (dd, $J$ = 7.3, 4.6 Hz, 1H), 4.23 – 4.05 (m, 3H), 3.79 – 3.75 (m, 2H), 3.73 – 3.68 (m, 1H), 3.65 (s, 1.5H), 3.65 – 3.57 (m, 0.5H), 3.44 – 3.37 (m, 0.5H), 3.36 – 3.27 (m, 1H), 3.27 – 3.09 (m, 1.5H), 2.41 (s, 3H), 2.31 (s, 3H). $^{13}$**C NMR** (101 MHz, $CDCl_3$) δ 172.3, 171.5, 171.3, 143.5, 138.0, 137.9, 137.4, 137.3, 132.6, 132.3, 130.0, 129.88, 129.85, 128.1, 128.0, 127.1, 52.64, 52.62, 52.50, 52.47, 50.9, 50.8, 48.9, 48.8, 46.5, 46.4, 32.2, 32.1, 21.6, 21.2. $^{1}$**H NMR** (500 MHz, DMSO-$d_6$, 393K) δ 7.67 – 7.63 (m, 2H), 7.37 – 7.33 (m, 2H), 7.12 – 7.06 (m, 5H), 4.08 – 3.85 (m, 3H), 3.71 – 3.61 (m, 4H), 3.46 – 3.39 (m, 1H), 3.40 – 3.31 (m, 1H), 3.00 – 2.92 (m, 2H), 2.39 (s, 3H), 2.28 (s, 3H). $^{13}$**C NMR** (126 MHz, DMSO-$d_6$, 393K) δ 171.6, 170.3, 141.9, 137.6, 135.8, 133.3, 128.8, 128.5, 127.2, 125.9, 51.1, 46.2, 45.1, 30.9, 20.2, 19.8. **Note:** At 393 K, the rotameric signals coalesce and the alkyl carbon adjacent to the amide nitrogen is broadened beyond detection. **HRMS** (ESI) calcd. for ($C_{22}H_{27}N_2O_5S$) $[M+H]^+$: 431.1635, found 431.1647. **IR** (neat): 3250, 2954, 2923, 1738, 1639, 1455, 1436, 1330, 1207, 1160, 1093, 816, 661, 551. **Optical rotation**: $[\alpha]^{26}_{D}$ = 11.7 (c 0.1, $CHCl_3$, 95.5:4.5 er). The enantiomeric excess of **4e** was determined by SFC analysis on a Chiralpak ID-3 column ($CO_2$/MeOH with a gradient from 100% to 70% $CO_2$ in 5 min, then maintained at 70% $CO_2$, column temperature 35 °C, flow rate 1.2 mL/min) with retention time 7.12 min (major) and 8.28 min (minor).

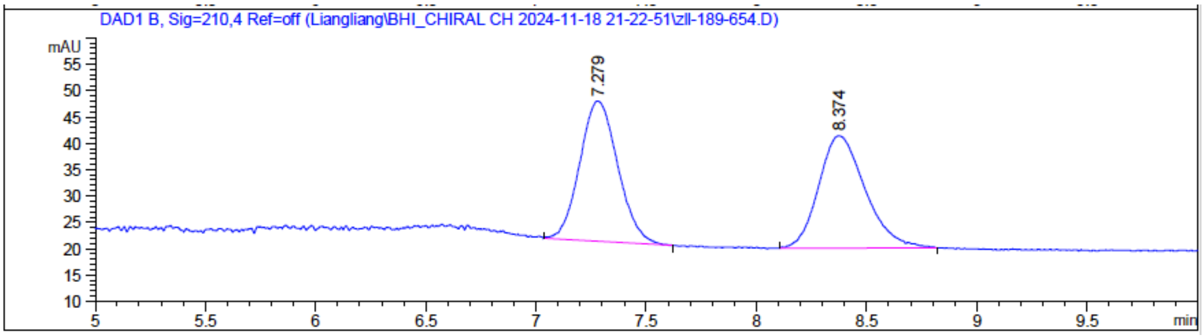


Signal 2: DAD1 B, Sig=210,4 Ref=off

| Peak # | RetTime [min] | Type | Width [min] | Area [mAU*s] | Height [mAU] | Area % |
|---|---|---|---|---|---|---|
| 1 | 7.279 | BV R | 0.1605 | 319.29443 | 26.60192 | 50.4418 |
| 2 | 8.374 | VV R | 0.1815 | 313.70081 | 21.33823 | 49.5582 |

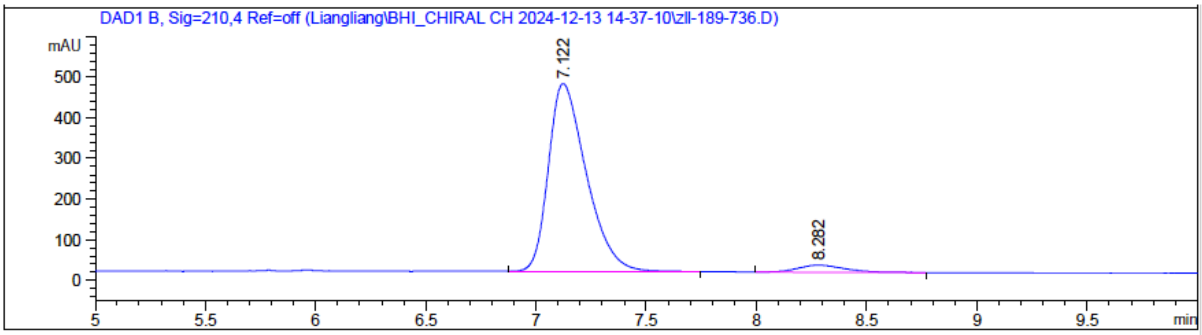


Signal 2: DAD1 B, Sig=210,4 Ref=off

| Peak # | RetTime [min] | Type | Width [min] | Area [mAU*s] | Height [mAU] | Area % |
|---|---|---|---|---|---|---|
| 1 | 7.122 | BB | 0.1788 | 5545.96143 | 462.70514 | 95.5638 |
| 2 | 8.282 | BV R | 0.1770 | 257.45212 | 17.56227 | 4.4362 |

**N-((S)-3-((3aR,6aS)-hexahydrocyclopenta[c]pyrrol-2(1H)-yl)-3-oxo-2-(p-tolyl)propyl)-4-methylbenzenesulfonamide(4f):** Following General Procedure C, 2-(p-tolyl)-1-tosylaziridine (**1a**) (43.1 mg, 0.15 mmol), and diethyl 2,6-dimethyl-4-((3a*R*,6a*S*)-octahydrocyclopenta[*c*]pyrrole-2-carbonyl)-1,4-dihydropyridine-3,5-dicarboxylate (**2g**) (117.1 mg, 0.30 mmol) were used, affording the title compound as a yellow oil (39.2 mg, 61% yield) by using $^i$PrOAc/Acetone (20:1) as eluent. In an independent experiment, 34.8 mg (54% yield) were obtained, giving an average yield of 58% with 94:6 er. **$^1$H NMR** (400 MHz, $CDCl_3$) δ 7.71 – 7.67 (m, 2H), 7.29 – 7.22 (m, 2H), 7.12 – 7.01 (m, 4H), 5.38 – 5.29 (m, 1H), 3.85 – 3.76 (m, 1H), 3.70 – 3.54 (m, 1H), 3.49 – 3.41 (m, 0.5H), 3.35 – 3.14 (m, 3H), 3.14 – 3.00 (m, 1H), 2.78 – 2.70 (m, 0.5H), 2.60 – 2.42 (m, 2H), 2.39 (s, 3H), 2.30 (s, 3H), 1.84 – 1.68 (m, 2H), 1.63 – 1.53 (m, 1H), 1.51 – 1.34 (m, 2H), 1.32 – 1.25 (m, 0.5H), 0.94 – 0.87 (m, 0.5H). **$^{13}$C NMR** (101 MHz, $CDCl_3$) δ 170.0, 169.9, 143.31, 143.29, 137.6, 137.49, 137.46, 133.3, 133.2, 129.9, 129.83, 129.82, 129.79, 128.1, 128.0, 127.08, 127.06, 52.4, 52.1, 51.9, 51.39, 51.37,

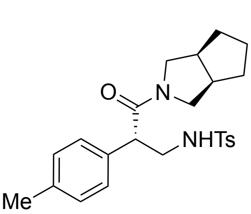


**4f**

47.27, 47.26, 43.6, 43.5, 41.7, 32.17, 32.16, 32.0, 31.7, 25.6, 25.4, 21.6, 21.20, 21.18. **$^1$H NMR** (500 MHz, DMSO-d$_6$, 393K) δ 7.67 – 7.63 (m, 2H), 7.36 – 7.32 (m, 2H), 7.11 – 7.06 (m, 4H), 7.02 (t, $J$ = 5.9 Hz, 1H), 3.83 (dd, $J$ = 8.0, 5.8 Hz, 1H), 3.53 – 3.35 (m, 3H), 3.19 – 2.93 (m, 3H), 2.57 – 2.51 (m, 2H), 2.39 (s, 3H), 2.27 (s, 3H), 1.81 – 0.94 (m, 6H). **$^{13}$C NMR** (126 MHz, DMSO-d$_6$, 393K) δ 168.5, 141.8, 137.8, 135.7, 133.8, 128.8, 128.5, 127.2, 125.9, 51.0, 48.8, 45.9, 31.0, 30.9, 24.4, 20.2, 19.8. **Note:** At 393 K, the rotameric signals coalesce and the alkyl carbon adjacent to the amide nitrogen is broadened beyond detection. **HRMS** (ESI) calcd. for ($C_{24}H_{31}N_2O_3S$) $[M+H]^+$: 427.2050, found 427.2051. **IR** (neat): 3184, 2947, 2869, 1623, 1513, 1448, 1330, 1159, 1093, 816, 753, 661, 551. **Optical rotation**: $[\alpha]^{26}_D$ = 25.2 (c 0.2, $CHCl_3$, 94:6 er). The enantiomeric excess of **4f** was determined by SFC analysis on a Chiralpak ID-3 column ($CO_2$/MeOH with a gradient from 100% to 70% $CO_2$ in 5 min, then maintained at 70% $CO_2$, column temperature 35 °C, flow rate 1.2 mL/min) with retention time 7.08 min (major) and 8.98 min (minor).

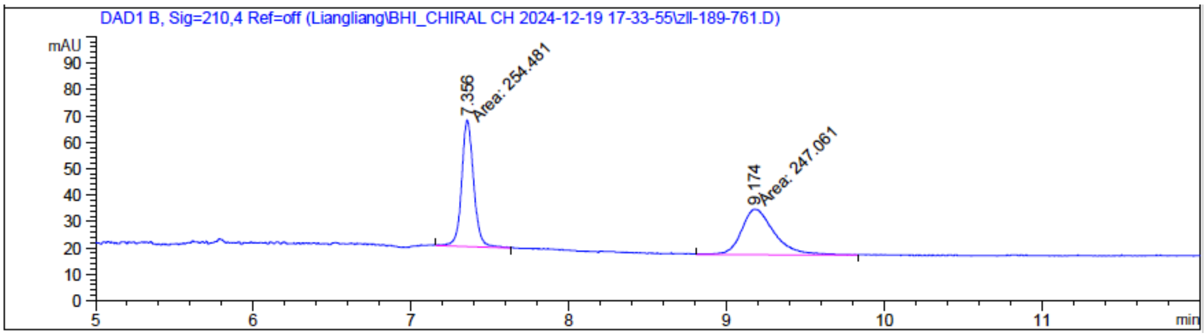


Signal 2: DAD1 B, Sig=210,4 Ref=off

| Peak # | RetTime [min] | Type | Width [min] | Area [mAU*s] | Height [mAU] | Area % |
|---|---|---|---|---|---|---|
| 1 | 7.356 | MM | 0.0885 | 254.48077 | 47.90375 | 50.7397 |
| 2 | 9.174 | MM | 0.2400 | 247.06050 | 17.15961 | 49.2603 |

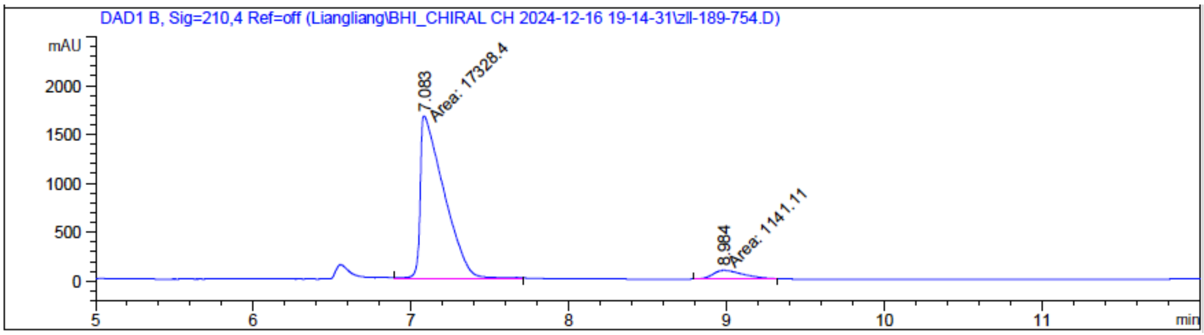


Signal 2: DAD1 B, Sig=210,4 Ref=off

| Peak # | RetTime [min] | Type | Width [min] | Area [mAU*s] | Height [mAU] | Area % |
|---|---|---|---|---|---|---|
| 1 | 7.083 | MM | 0.1739 | 1.73284e4 | 1661.17029 | 93.8217 |
| 2 | 8.984 | MM | 0.2194 | 1141.10889 | 86.68951 | 6.1783 |

***tert*-butyl (3a*R*,6a*S*)-5-((*S*)-3-((4-methylphenyl)sulfonamido)-2-(*p*-tolyl)propanoyl)hexahydropyrrolo[3,4-*c*]pyrrole-2(1*H*)-carboxylate (4g):** Following General Procedure C, 2-(p-tolyl)-1-tosylaziridine (**1a**) (43.1 mg, 0.15 mmol), and diethyl 4-((3a*R*,6a*S*)-5-(*tert*-butoxycarbonyl)octahydropyrrolo[3,4-*c*]pyrrole-2-carbonyl)-2,6-dimethyl-1,4-dihydropyridine-3,5-dicarboxylate (**2h**) (147.5 mg, 0.30 mmol) were used, affording the title compound as a yellow oil (34.2 mg, 43% yield) by using $^{i}$PrOAc/Acetone (20:1) as eluent. In an independent experiment, 36.6 mg (46% yield) were obtained, giving an average yield of 45% with 92.5:7.5 er. $^{1}$**H NMR** (400 MHz, $CDCl_3$) δ 7.71 – 7.67 (m, 2H), 7.29 – 7.22 (m, 2H), 7.10 (d, $J$ = 7.8 Hz, 2H), 7.04 (d, $J$ = 8.0 Hz, 2H), 5.36 – 5.28 (m, 1H), 3.87 – 3.67 (m, 2H), 3.61 – 3.02 (m, 8H), 2.86 – 2.66 (m, 3H), 2.39 (d, $J$ = 3.0 Hz, 3H), 2.30 (d, $J$ = 2.6 Hz, 3H), 1.47 (s, 4.5H), 1.39 (s, 4.5H). $^{13}$**C NMR** (101 MHz, $CDCl_3$) δ 170.6, 170.5, 154.5, 154.4, 143.42, 143.37, 137.81, 137.78, 137.5, 137.4, 132.8, 132.6, 130.04, 129.98, 129.9, 129.8, 128.0, 127.9, 127.08, 127.05, 79.9, 79.7, 69.62, 54.0, 51.6, 51.5, 51.3, 50.0, 49.9, 49.8, 49.6, 49.3, 49.0, 47.2, 47.1, 42.7, 41.8, 40.7, 39.8, 31.9, 29.8, 29.4, 28.6, 28.5, 21.6, 21.2. $^{1}$**H NMR** (500 MHz, DMSO-$d_6$, 393K) δ 7.68 – 7.62 (m, 2H), 7.37 – 7.32 (m, 2H), 7.12 – 7.05 (m, 4H), 7.04 (t, $J$ = 5.9 Hz, 1H), 3.82 (dd, $J$ = 8.1, 5.7 Hz, 1H), 3.63 – 3.15 (m, 7H), 3.02 – 2.93 (m, 3H), 2.81 – 2.74 (m, 2H), 2.39 (s, 3H), 2.27 (s, 3H), 1.40 (s, 9H). $^{13}$**C NMR** (126 MHz, DMSO-$d_6$, 393K) δ 169.0, 153.0, 141.8, 137.8, 135.8, 133.6, 128.8, 128.5, 127.2, 125.9, 77.9, 55.4, 49.1, 49.1, 48.8, 45.9, 27.6, 20.2, 19.8. **Note:** At 393 K, the rotameric signals coalesce and the alkyl carbon adjacent to the amide nitrogen is broadened beyond detection. **HRMS** (ESI) calcd. for ($C_{28}H_{37}N_3NaO_5S$) $[M+Na]^+$: 550.2346, found 550.2345. **IR** (neat): 33191, 2973, 2925, 2876, 1693, 1629, 1448, 1402, 1366, 1332, 1160, 1130, 1095, 816, 754, 662, 556. **Optical rotation**: $[\alpha]^{26}_{D}$ = 19.6 (c 0.1, $CHCl_3$, 92.5:7.5 er). The enantiomeric excess of **4g** was determined by SFC analysis on a Chiralpak ID-3 column ($CO_2$/MeOH with a gradient from 100% to 70% $CO_2$ in 5 min, then maintained at 70% $CO_2$, column temperature 35 °C, flow rate 1.2 mL/min) with retention time 7.08 min (major) and 8.98 min (minor).

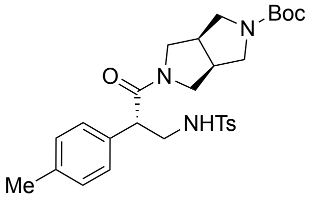

**4g**

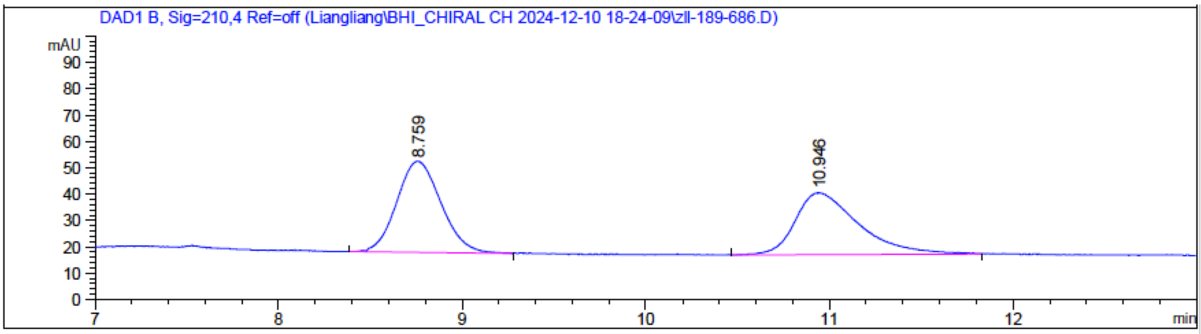


Signal 2: DAD1 B, Sig=210,4 Ref=off

| Peak # | RetTime [min] | Type | Width [min] | Area [mAU*s] | Height [mAU] | Area % |
|---|---|---|---|---|---|---|
| 1 | 8.759 | VV R | 0.2024 | 579.45129 | 34.59475 | 50.8030 |
| 2 | 10.946 | VV R | 0.2862 | 561.13251 | 23.40782 | 49.1970 |

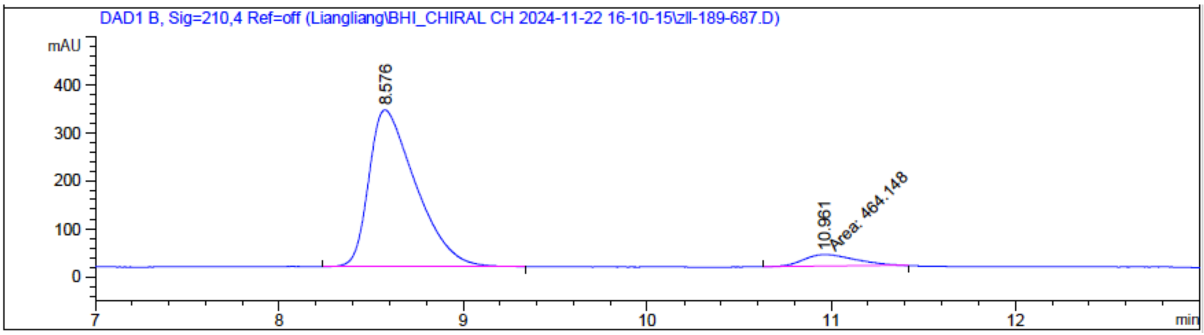


Signal 2: DAD1 B, Sig=210,4 Ref=off

| Peak # | RetTime [min] | Type | Width [min] | Area [mAU*s] | Height [mAU] | Area % |
|---|---|---|---|---|---|---|
| 1 | 8.576 | BV R | 0.2538 | 5770.70459 | 326.41129 | 92.5556 |
| 2 | 10.961 | MM | 0.3240 | 464.14783 | 23.87385 | 7.4444 |

**(S)-4-methyl-*N*-(3-oxo-3-(piperidin-1-yl)-2-(*p*-tolyl)propyl)benzenesulfonamide (4h):** Following General Procedure C, 2-(p-tolyl)-1-tosylaziridine (**1a**) (43.1 mg, 0.15 mmol), and diethyl 2,6-dimethyl-4-(piperidine-1-carbonyl)-1,4-dihydropyridine-3,5-dicarboxylate (**2i**) (109.3mg, 0.30 mmol) were used, affording the title compound as a colorless oil (40.7 mg, 68% yield) by two-step column chromatography using Hexane/EtOAc (2:1), then Hexane/Acetone (2:1) as eluents. In an independent experiment, 36.9 mg (62% yield) were obtained, giving an average yield of 65% with 93.5:6.5 er. $^{1}$**H NMR** (400 MHz, $CDCl_3$) δ 7.71 – 7.67 (m, 2H), 7.25 (d, $J$ = 8.2 Hz, 2H), 7.09 (d, $J$ = 8.0 Hz, 2H), 7.04 (d, $J$ = 8.1 Hz, 2H), 5.34 (dd, $J$ = 9.3, 4.5 Hz, 1H), 3.96 (dd, $J$ = 9.6, 4.7 Hz, 1H), 3.73 – 3.63 (m, 1H), 3.39 – 3.25 (m, 2H), 3.21 – 3.12 (m, 3H), 2.39 (s, 3H), 2.30 (s, 3H), 1.56 – 1.25 (m, 5H), 0.95 – 0.83 (m, 1H). $^{13}$**C NMR** (101 MHz, $CDCl_3$) δ 169.7, 143.3, 137.5, 133.8, 129.9, 129.8, 127.8, 127.1, 50.0, 47.6, 46.6, 43.2, 25.7, 25.6, 24.4, 21.6, 21.2. **HRMS** (ESI) calcd. for ($C_{22}H_{29}N_2O_3S$) $[M+H]^+$: 401.1893, found 401.1902. **IR** (neat): 3229, 2935, 2856, 1622, 145, 1329, 1160, 1093, 815, 661, 558. **Optical rotation**: $[\alpha]^{26}_{D}$ = 16.0 (c 0.3, $CHCl_3$, 93.5:6.5 er). The enantiomeric excess of **4h** was determined by SFC analysis on a Chiralpak ID-3 column ($CO_2$/MeOH with a gradient from 100% to 70% $CO_2$ in 5 min, then maintained at 70% $CO_2$, column temperature 35 °C, flow rate 1.2 mL/min) with retention time 6.25 min (major) and 7.74 min (minor).

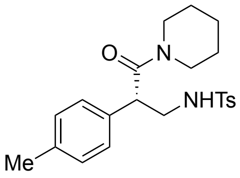


**4h**

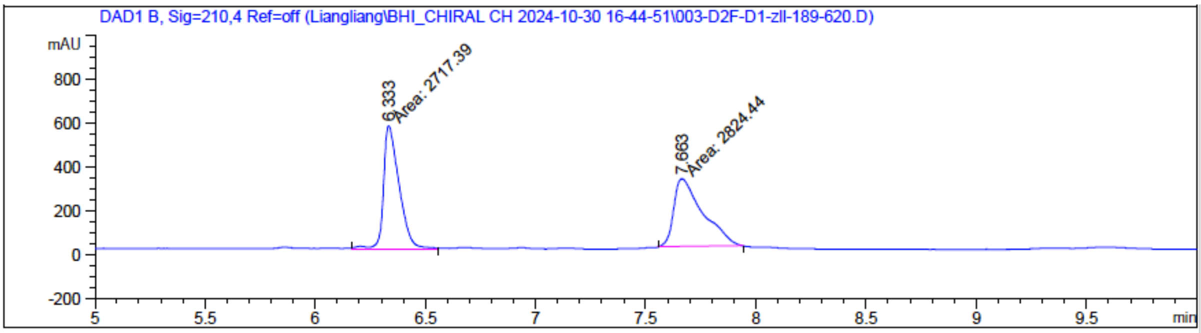

Signal 2: DAD1 B, Sig=210,4 Ref=off

| Peak # | RetTime [min] | Type | Width [min] | Area [mAU*s] | Height [mAU] | Area % |
|---|---|---|---|---|---|---|
| 1 | 6.333 | MM | 0.0810 | 2717.38721 | 559.22327 | 49.0342 |
| 2 | 7.663 | MM | 0.1527 | 2824.43604 | 308.37802 | 50.9658 |

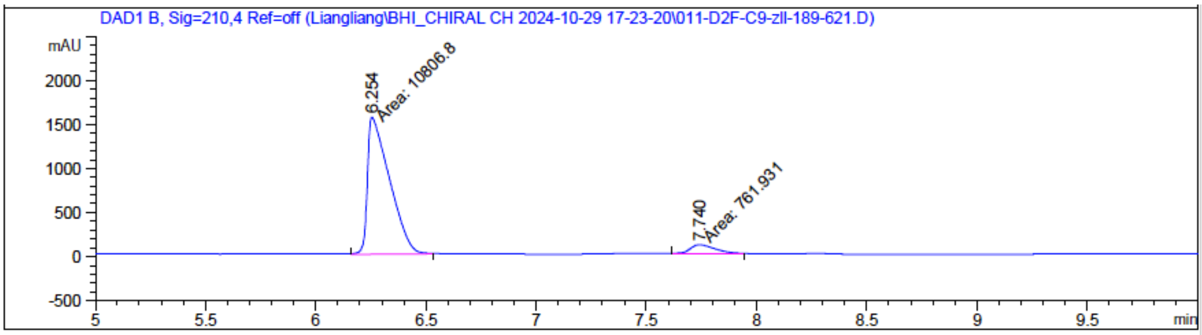


Signal 2: DAD1 B, Sig=210,4 Ref=off

| Peak # | RetTime [min] | Type | Width [min] | Area [mAU*s] | Height [mAU] | Area % |
|---|---|---|---|---|---|---|
| 1 | 6.254 | MM | 0.1157 | 1.08068e4 | 1557.22778 | 93.4139 |
| 2 | 7.740 | MM | 0.1297 | 761.93134 | 97.93722 | 6.5861 |

**(*S*)-4-methyl-*N*-(3-oxo-3-(4-(pyrimidin-2-yl)piperazin-1-yl)-2-(*p*-tolyl)propyl)benzenesulfonamide (4i):** Following General Procedure C, 2-(*p*-tolyl)-1-tosylaziridine (**1a**) (43.1 mg, 0.15 mmol), and diethyl 2,6-dimethyl-4-(4-(pyrimidin-2-yl)piperazine-1-carbonyl)-1,4-dihydropyridine-3,5-dicarboxylate (**2j**) (133.1 mg, 0.30 mmol) were used, affording the title compound

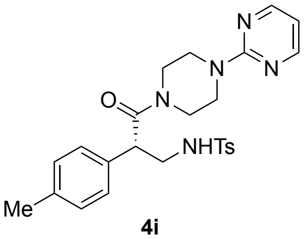


**4i**

as a colorless oil (27.8 mg, 39% yield) by two-step column chromatography using Hexane/EtOAc (2:1), then Hexane/Acetone (2:1) as eluents. In an independent experiment, 30.0 mg (42% yield) were obtained, giving an average yield of 40% with 92:8 er. **$^1$H NMR** (400 MHz, $CDCl_3$) δ 8.27 (d, $J$ = 4.8 Hz, 2H), 7.72 – 7.69 (m, 2H), 7.26 (d, $J$ = 8.0 Hz, 2H), 7.12 – 7.05 (m, 4H), 6.50 (t, $J$ = 4.7 Hz, 1H), 5.30 (dd, $J$ = 9.1, 4.7 Hz, 1H), 4.06 – 3.96 (m, 2H), 3.92 – 3.87 (m, 1H), 3.83 – 3.73 (m, 1H), 3.53 – 3.42 (m, 2H), 3.40 – 3.16 (m, 4H), 3.03 – 2.92 (m, 1H), 2.39 (s, 3H), 2.29 (s, 3H). **$^{13}$C NMR** (101 MHz, $CDCl_3$) δ 170.4, 161.2, 157.8, 143.4, 137.9, 137.5, 133.4, 130.2, 129.9, 127.8, 127.1, 110.5, 50.3, 47.4, 45.3, 43.5, 43.3, 41.9, 21.6, 21.2. **HRMS** (ESI) calcd. for ($C_{25}H_{29}N_5NaO_3S$) $[M+Na]^+$: 502.1883, found 502.1901. **IR** (neat): 3243, 3023, 2924, 2858, 1631, 1585, 1550, 1495, 1441, 1356, 1329, 1225, 1159, 1093, 983, 815, 757, 662, 559. **Optical rotation**: $[\alpha]^{26}_D$ = 27.4 (c 0.2, $CHCl_3$, 92:8 er). The enantiomeric excess of **4i** was determined by SFC analysis on a Chiralpak ID-3 column ($CO_2$/MeOH with a gradient from 100% to 70% $CO_2$ in 5 min, then maintained at 70% $CO_2$, column temperature 35 °C, flow rate 1.2 mL/min) with retention time 9.98 min (major) and 12.48 min (minor).

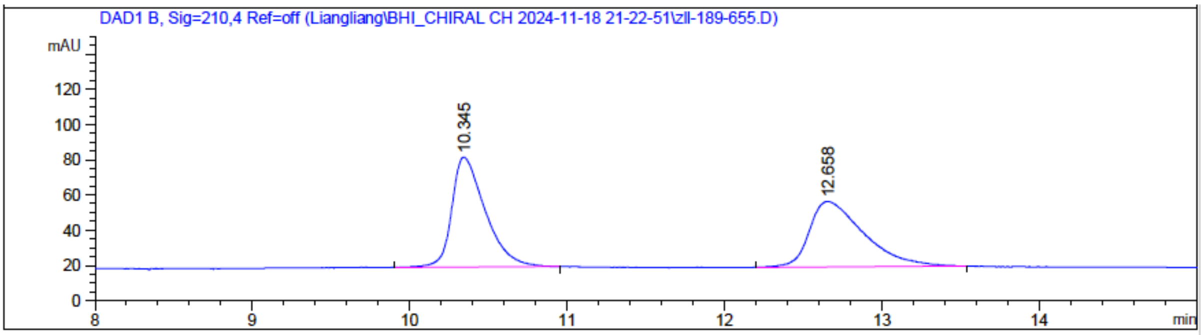


Signal 2: DAD1 B, Sig=210,4 Ref=off

| Peak # | RetTime [min] | Type | Width [min] | Area [mAU*s] | Height [mAU] | Area % |
|---|---|---|---|---|---|---|
| 1 | 10.345 | VV R | 0.2136 | 926.30176 | 62.42361 | 50.9168 |
| 2 | 12.658 | VV R | 0.2838 | 892.94543 | 37.16135 | 49.0832 |

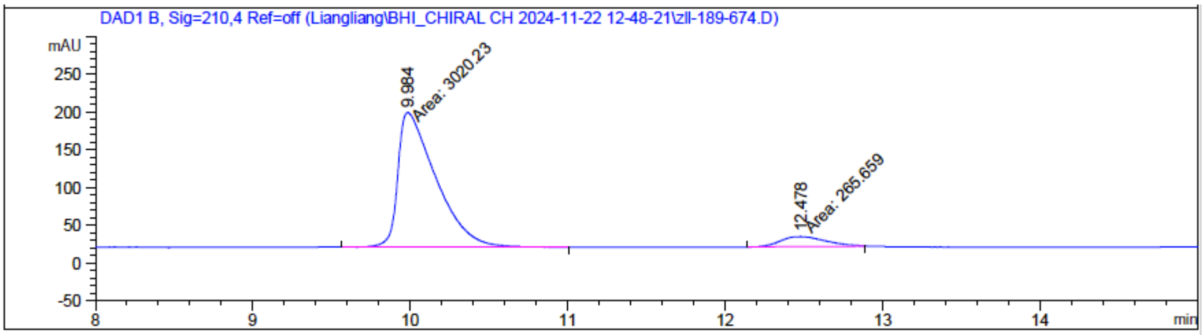


Signal 2: DAD1 B, Sig=210,4 Ref=off

| Peak # | RetTime [min] | Type | Width [min] | Area [mAU*s] | Height [mAU] | Area % |
|---|---|---|---|---|---|---|
| 1 | 9.984 | MM | 0.2815 | 3020.23071 | 178.81689 | 91.9152 |
| 2 | 12.478 | MM | 0.3290 | 265.65866 | 13.45799 | 8.0848 |

**(*S*)-4-methyl-*N*-(3-oxo-3-(2-azaspiro[3.3]heptan-2-yl)-2-(*p*-tolyl)propyl)benzenesulfonamide (4j):** Following General Procedure C, 2-(p-tolyl)-1-tosylaziridine (**1a**) (43.1 mg, 0.15 mmol), and diethyl 2,6-dimethyl-4-(2-azaspiro[3.3]heptane-2-carbonyl)-1,4-dihydropyridine-3,5-dicarboxylate (**2k**) (84.6 mg, 0.225 mmol) were used, affording the title compound as a white solid (38.8 mg, 63% yield) by two-step column chromatography using $^{i}$PrOAc/Acetone (20:1), then Hexane/Acetone (2:1) as eluents. In an independent experiment, 39.4 mg (64% yield) were obtained, giving an average yield of 63% with 97.5:2.5 er. **M.p.**: 46-49 °C. **$^{1}$H NMR** (400 MHz, $CDCl_3$) δ 7.71 – 7.67 (m, 2H), 7.26 (d, *J* = 8.0 Hz, 2H), 7.10 (d, *J* = 7.9 Hz, 2H), 7.05 (d, *J* = 8.1 Hz, 2H), 5.32 (s, 1H), 3.95 – 3.91 (m, 2H), 3.85 (d, *J* = 10.1 Hz, 1H), 3.64 (dd, *J* = 9.7, 5.0 Hz, 1H), 3.51 (d, *J* = 8.8 Hz, 1H), 3.34 – 3.24 (m, 1H), 3.19 – 3.10 (m, 1H), 2.40 (s, 3H), 2.31 (s, 3H), 2.19 – 2.08 (m, 2H), 2.05 – 1.98 (m, 1H), 1.97 – 1.86 (m, 1H), 1.85 – 1.69 (m, 2H). **$^{13}$C NMR** (101 MHz, $CDCl_3$) δ 171.1, 143.3, 137.6, 137.4, 133.0, 129.8, 128.0, 127.1, 62.4, 60.5, 48.8, 46.6, 37.9, 33.3, 32.8, 21.6, 21.2, 16.1. **HRMS** (ESI) calcd. for

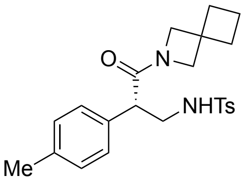


**4j**

($C_{23}H_{29}N_2O_3S$) $[M+H]^+$: 413.1893, found 413.1881. **IR** (neat): 3186, 2934, 2866, 1631, 1457, 1330, 1158, 1093, 815, 753, 660, 554. **Optical rotation**: $[\alpha]^{26}_{D}$ = 23.8 (c 0.3, $CHCl_3$, 97.5:2.5 er). The enantiomeric excess of **4j** was determined by SFC analysis on a Chiralpak ID-3 column ($CO_2$/MeOH with a gradient from 100% to 70% $CO_2$ in 5 min, then maintained at 70% $CO_2$, column temperature 35 °C, flow rate 1.2 mL/min) with retention time 6.82 min (major) and 8.19 min (minor).

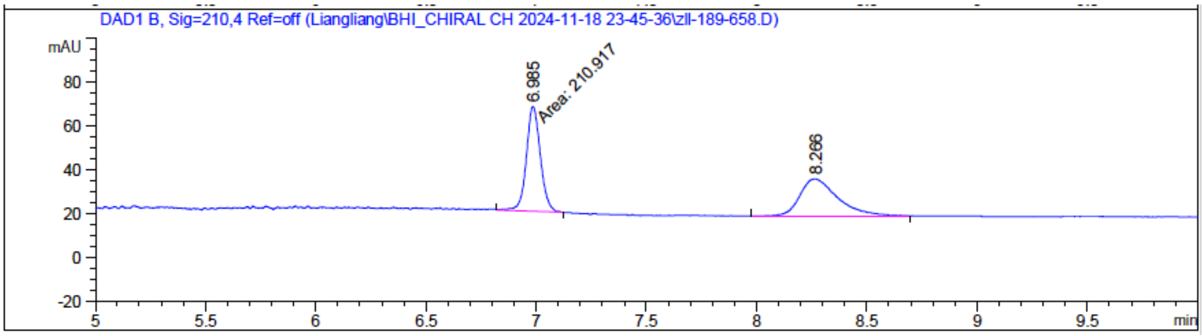


Signal 2: DAD1 B, Sig=210,4 Ref=off

| Peak # | RetTime [min] | Type | Width [min] | Area [mAU*s] | Height [mAU] | Area % |
|---|---|---|---|---|---|---|
| 1 | 6.985 | MM | 0.0736 | 210.91711 | 47.76458 | 50.7872 |
| 2 | 8.266 | VV R | 0.1556 | 204.37907 | 17.01607 | 49.2128 |

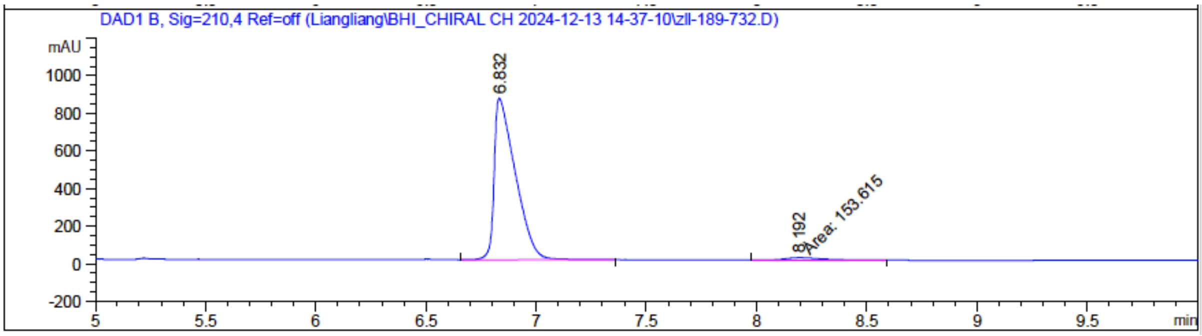


Signal 2: DAD1 B, Sig=210,4 Ref=off

| Peak # | RetTime [min] | Type | Width [min] | Area [mAU*s] | Height [mAU] | Area % |
|---|---|---|---|---|---|---|
| 1 | 6.832 | BV R | 0.0977 | 5718.99756 | 858.60132 | 97.3842 |
| 2 | 8.192 | MM | 0.1996 | 153.61505 | 12.82374 | 2.6158 |

**(*S*)-4-methyl-*N*-(3-oxo-3-(7-oxa-2-azaspiro[3.5]nonan-2-yl)-2-(*p*-tolyl)propyl)benzenesulfonamide (4k):** Following General Procedure C, 2-(p-tolyl)-1-tosylaziridine (**1a**) (43.1 mg, 0.15 mmol), and diethyl 2,6-dimethyl-4-(7-oxa-2-azaspiro[3.5]nonane-2-carbonyl)-1,4-dihydropyridine-3,5-dicarboxylate (**2l**) (91.4 mg, 0.225 mmol) were used, affording the title compound as a colorless oil (19.6 mg, 30% yield) by two-step column chromatography using $^i$PrOAc/Acetone (20:1), then Hexane/Acetone (2:1) as eluents. In an independent experiment, 20.4 mg (31% yield) were obtained, giving an average yield of 30% with 97:3 er. **$^1$H NMR** (400 MHz, $CDCl_3$) δ 7.71 –

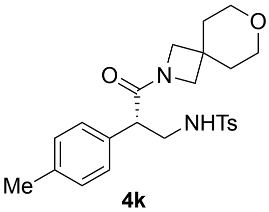

**4k**

7.68 (m, 2H), 7.27 (d, $J = 7.0$ Hz, 2H), 7.14 – 7.03 (m, 4H), 5.22 (dd, $J = 8.7$, 4.9 Hz, 1H), 3.77 – 3.64 (m, 4H), 3.59 (t, $J = 5.2$ Hz, 2H), 3.55 – 3.41 (m, 2H), 3.34 – 3.27 (m, 2H), 3.21 – 3.10 (m, 1H), 2.40 (s, 3H), 2.31 (s, 3H), 1.78 – 1.71 (m, 2H), 1.58 – 1.47 (m, 2H). **$^{13}$C NMR** (101 MHz, $CDCl_3$) δ 171.7, 143.4, 137.8, 137.4, 132.8, 129.91, 129.86, 128.0, 127.1, 64.8, 64.7, 60.3, 58.3, 48.9, 46.6, 35.98, 35.95, 33.3, 21.6, 21.2. **HRMS** (ESI) calcd. for ($C_{24}H_{30}N_2NaO_4S$) $[M+Na]^+$: 465.1818, found 465.1826. **IR** (neat): 3169, 2923, 2852, 1634, 1459, 1330, 1159, 1106, 816, 754, 659, 552. **Optical rotation**: $[\alpha]^{26}_D$ = 32.0 (c 0.1, $CHCl_3$, 97:3 er). The enantiomeric excess of **4k** was determined by SFC analysis on a Chiralpak ID-3 column ($CO_2$/MeOH with a gradient from 100% to 70% $CO_2$ in 5 min, then maintained at 70% $CO_2$, column temperature 35 °C, flow rate 1.2 mL/min) with retention time 7.10 min (major) and 8.82 min (minor).

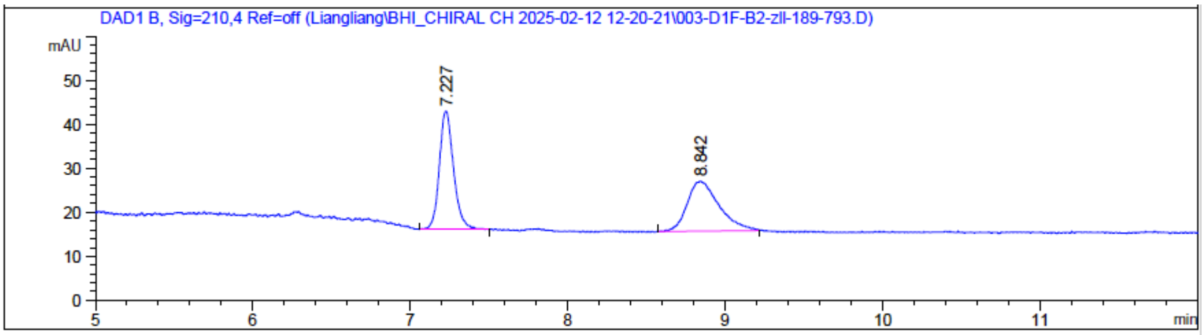


Signal 2: DAD1 B, Sig=210,4 Ref=off

| Peak # | RetTime [min] | Type | Width [min] | Area [mAU*s] | Height [mAU] | Area % |
|---|---|---|---|---|---|---|
| 1 | 7.227 | VV R | 0.0974 | 169.51357 | 26.92720 | 51.4438 |
| 2 | 8.842 | VV R | 0.1676 | 159.99846 | 11.37818 | 48.5562 |

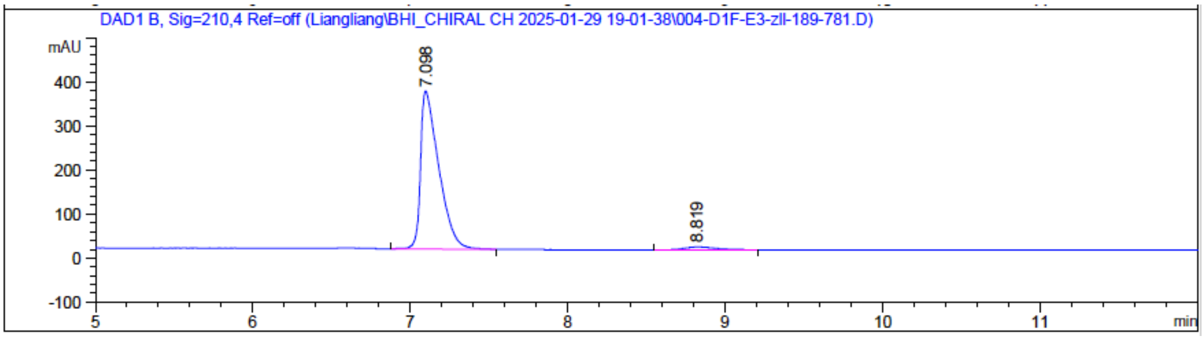


Signal 2: DAD1 B, Sig=210,4 Ref=off

| Peak # | RetTime [min] | Type | Width [min] | Area [mAU*s] | Height [mAU] | Area % |
|---|---|---|---|---|---|---|
| 1 | 7.098 | VB R | 0.1195 | 2888.19409 | 358.52188 | 96.8607 |
| 2 | 8.819 | VV R | 0.1718 | 93.60697 | 6.60613 | 3.1393 |

***tert*-butyl (*S*)-2-(3-((4-methylphenyl)sulfonamido)-2-(*p*-tolyl)propanoyl)-2,7-diazaspiro[3.5]nonane-7-carboxylate (4l):** Following General Procedure C, 2-(p-tolyl)-1-tosylaziridine (**1a**) (43.1 mg, 0.15 mmol), and diethyl 4-(7-(*tert*-butoxycarbonyl)-2,7-diazaspiro[3.5]nonane-2-carbonyl)-2,6-dimethyl-1,4-dihydropyridine-3,5-dicarboxylate (**2m**) (113.7 mg, 0.225 mmol) were used, affording the title compound as a colorless oil (29.1 mg, 36% yield) by two-step column chromatography using $^{i}$PrOAc/Acetone (20:1), then Hexane/Acetone (2:1) as eluents. In an independent experiment, 26.7 mg (33% yield) were obtained, giving an average yield of 34% with 97:3 er.

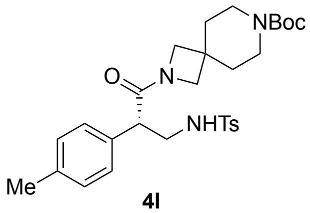


**4l**

**$^{1}$H NMR** (400 MHz, $CDCl_3$) δ 7.71 – 7.67 (m, 2H), 7.30 – 7.23 (m, 2H), 7.14 – 7.07 (m, 2H), 7.09 – 7.02 (m, 2H), 5.22 (dd, $J$ = 8.7, 4.9 Hz, 1H), 3.76 – 3.66 (m, 3H), 3.63 (d, $J$ = 9.7 Hz, 1H), 3.39 – 3.12 (m, 7H), 2.40 (s, 3H), 2.31 (s, 3H), 1.72 – 1.64 (m, 2H), 1.52 – 1.45 (m, 2H), 1.43 (s, 9H). **$^{13}$C NMR** (101 MHz, $CDCl_3$) δ 171.8, 154.8, 143.4, 137.8, 137.3, 132.8, 129.91, 129.85, 128.0, 127.1, 79.9, 59.8, 57.7, 48.9, 46.6, 40.8, 35.1, 34.1, 28.5, 21.6, 21.2. **HRMS** (ESI) calcd. for ($C_{29}H_{40}N_3O_5S$) $[M+H]^+$: 542.2683, found 542.2694. **IR** (neat): 3171, 2970, 2926, 2870, 1692, 1636, 1455, 1423, 1366, 1330, 1242, 1159, 1093, 816, 755, 663, 556. **Optical rotation**: $[\alpha]^{26}_{D}$ = 20.1 (c 0.2, $CHCl_3$, 97:3 er). The enantiomeric excess of **4l** was determined by SFC analysis on a Chiralpak ID-3 column ($CO_2$/MeOH with a gradient from 100% to 70% $CO_2$ in 5 min, then maintained at 70% $CO_2$, column temperature 35 °C, flow rate 1.2 mL/min) with retention time 7.80 min (major) and 10.04 min (minor).

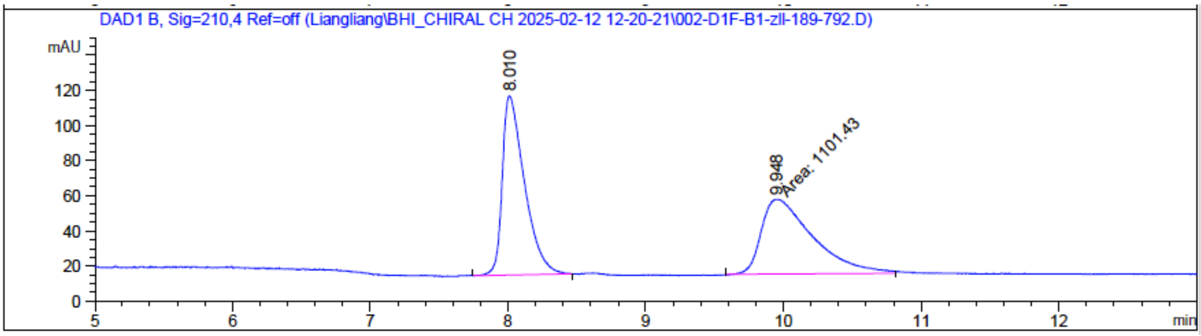


Signal 2: DAD1 B, Sig=210,4 Ref=off

| Peak # | RetTime [min] | Type | Width [min] | Area [mAU*s] | Height [mAU] | Area % |
|---|---|---|---|---|---|---|
| 1 | 8.010 | VV R | 0.1647 | 1143.50403 | 102.05436 | 50.9372 |
| 2 | 9.948 | MM | 0.4310 | 1101.42725 | 42.59563 | 49.0628 |

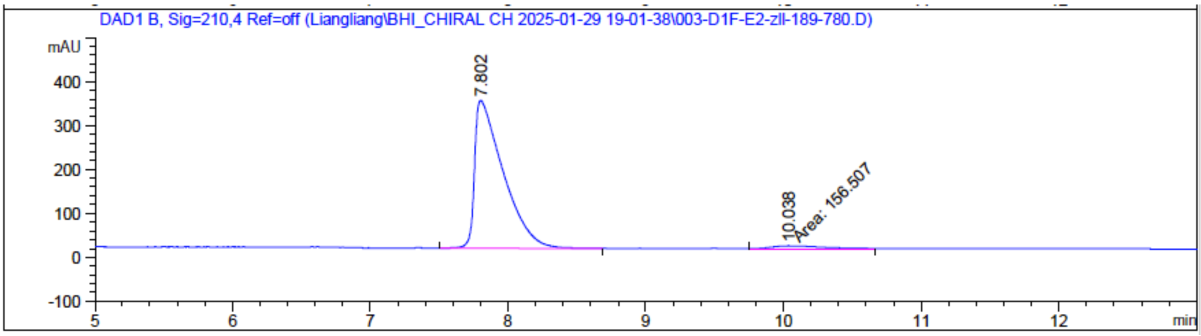

Signal 2: DAD1 B, Sig=210,4 Ref=off

| Peak # | RetTime [min] | Type | Width [min] | Area [mAU*s] | Height [mAU] | Area % |
|---|---|---|---|---|---|---|
| 1 | 7.802 | BV R | 0.2046 | 4882.03955 | 336.87546 | 96.8938 |
| 2 | 10.038 | MM | 0.4002 | 156.50664 | 6.51707 | 3.1062 |

**(*S*)-4-methyl-*N*-(3-oxo-3-(1,4-dioxa-8-azaspiro[4.5]decan-8-yl)-2-(*p*-tolyl)propyl)benzenesulfonamide (4m):** Following General Procedure C, 2-(p-tolyl)-1-tosylaziridine

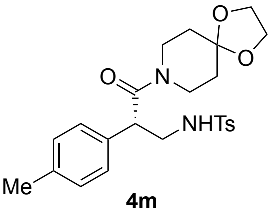

**4m**

(**1a**) (43.1 mg, 0.15 mmol), and diethyl 2,6-dimethyl-4-(1,4-dioxa-8-azaspiro[4.5]decane-8-carbonyl)-1,4-dihydropyridine-3,5-dicarboxylate (**2n**) (126.7 mg, 0.30 mmol) were used, affording the title compound as a colorless oil (37.8 mg, 55% yield) by two-step column chromatography using Hexane/EtOAc (2:1), then Hexane/Acetone (2:1) as eluents. In an independent experiment, 38.8 mg (56% yield) were obtained, giving an average yield of 56% with 92.5:7.5 er. **$^1$H NMR** (400 MHz, $CDCl_3$) δ 7.73 – 7.65 (m, 2H), 7.26 (d, *J* = 8.0 Hz, 2H), 7.10 (d, *J* = 8.0 Hz, 2H), 7.03 (d, *J* = 8.1 Hz, 2H), 5.28 (dd, *J* = 9.2, 4.6 Hz, 1H), 4.02 – 3.80 (m, 6H), 3.43 – 3.13 (m, 5H), 2.40 (s, 3H), 2.29 (s, 3H), 1.68 – 1.62 (m, 1H), 1.58 – 1.46 (m, 1H), 1.41 – 1.31 (m, 1H), 1.00 – 0.88 (m, 1H). **$^{13}$C NMR** (101 MHz, $CDCl_3$) δ 169.9, 143.4, 137.7, 137.5, 133.6, 130.1, 129.8, 127.7, 127.1, 106.8, 64.5, 50.1, 47.5, 43.5, 40.2, 34.73, 34.67, 21.6, 21.2. **HRMS** (ESI) calcd. for ($C_{24}H_{31}N_2O_5S$) $[M+H]^+$: 459.1948, found 459.1952. **IR** (neat): 3224, 2959, 2926, 2882, 1628, 1445, 1327, 1160, 1095, 945, 816, 662, 554. **Optical rotation**: $[\alpha]^{26}_D$ = 19.9 (c 0.2, $CHCl_3$, 92.5:7.5 er). The enantiomeric excess of **4m** was determined by SFC analysis on a Chiralpak ID-3 column ($CO_2$/MeOH with a gradient from 100% to 70% $CO_2$ in 5 min, then maintained at 70% $CO_2$, column temperature 35 °C, flow rate 1.2 mL/min) with retention time 7.47 min (major) and 9.47 min (minor).

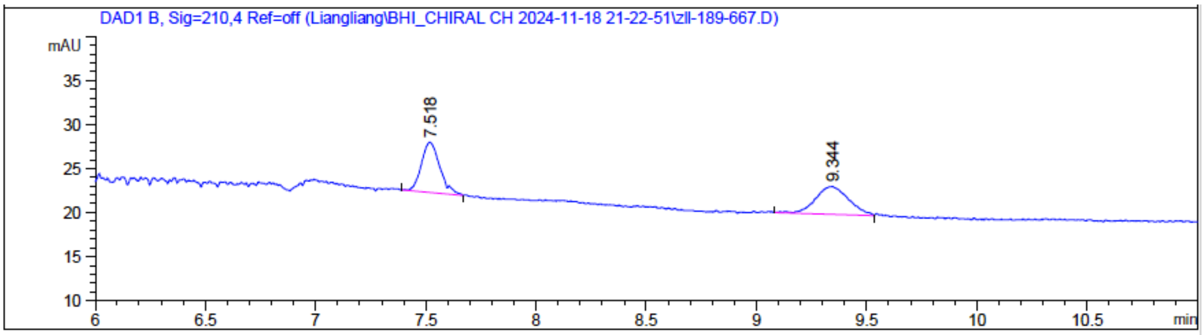


Signal 2: DAD1 B, Sig=210,4 Ref=off

| Peak # | RetTime [min] | Type | Width [min] | Area [mAU*s] | Height [mAU] | Area % |
|---|---|---|---|---|---|---|
| 1 | 7.518 | VV R | 0.0872 | 33.11778 | 5.70561 | 49.9417 |
| 2 | 9.344 | VB R | 0.1259 | 33.19514 | 3.16322 | 50.0583 |

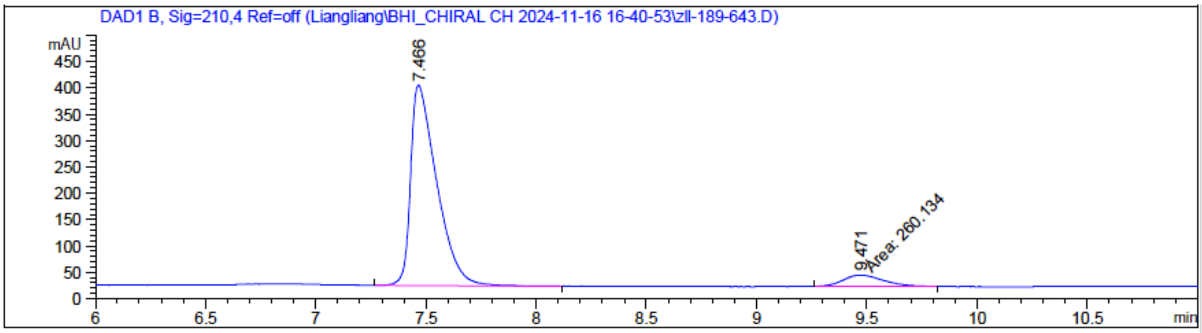


Signal 2: DAD1 B, Sig=210,4 Ref=off

| Peak # | RetTime [min] | Type | Width [min] | Area [mAU*s] | Height [mAU] | Area % |
|---|---|---|---|---|---|---|
| 1 | 7.466 | BB | 0.1214 | 3142.66553 | 380.49142 | 92.3553 |
| 2 | 9.471 | MM | 0.2050 | 260.13403 | 21.14608 | 7.6447 |

**(*S*)-*N*-benzyl-*N*-methyl-3-((4-methylphenyl)sulfonamido)-2-(*p*-tolyl)propanamide (4n):** Following General Procedure C, 2-(p-tolyl)-1-tosylaziridine (**1a**) (43.1 mg, 0.15 mmol), and diethyl 4-(benzyl(methyl)carbamoyl)-2,6-dimethyl-1,4-dihydropyridine-3,5-dicarboxylate (**2o**) (120.1 mg, 0.30 mmol) were used, affording the title compound as a colorless oil (33.3 mg, 51% yield) by two-step column chromatography using Hexane/EtOAc (2:1), then Hexane/Acetone (2:1) as eluents. In an independent experiment, 36.9 mg (56% yield) were obtained, giving an average yield of 54% with 88:12 er. $^{1}$**H NMR** (400 MHz, $CDCl_3$) δ 7.73 – 7.65 (m, 2H), 7.32 – 7.22 (m, 5H), 7.16 – 7.13 (m, 1.3H), 7.12 – 7.05 (m, 4H), 6.96 – 6.93 (m, 0.7H), 5.32 (ddd, *J* = 13.8, 9.1, 4.7 Hz, 1H), 4.65 (d, *J* = 14.7 Hz, 0.7H), 4.51 – 4.46 (m, 1H), 4.13 (d, *J* = 16.3 Hz, 0.3H), 4.10 – 3.96 (m, 1H), 3.42 – 3.30 (m, 1H), 3.28 – 3.14 (m, 1H), 2.86 (s,1H), 2.65 (s, 2H), 2.39 (s, 3H), 2.31 (s, 3H). $^{13}$**C NMR** (101 MHz, $CDCl_3$) δ 172.1, 171.9, 143.4, 143.3, 137.8, 137.7, 137.5, 137.3, 136.9, 135.8, 133.5, 133.1, 130.03, 129.97, 129.83, 129.82, 129.0, 128.8, 127.94, 127.90, 127.8, 127.6, 127.08, 127.06, 126.8, 52.9, 51.3, 50.3, 50.1, 47.6, 34.7, 33.8, 21.6, 21.2. $^{1}$**H NMR** (500 MHz, DMSO-$d_6$, 393K) δ 7.67 – 7.61 (m, 2H), 7.35 – 7.19 (m, 5H), 7.14 – 7.02 (m, 7H), 4.58 – 4.50 (m, 1H), 4.46 – 4.35 (m, 1H), 4.11 – 4.04 (m, 1H), 3.48 – 3.39 (m, 1H), 3.05 – 2.97 (m, 1H), 2.75 (s, 3H), 2.38 (s, 3H), 2.28 (s, 3H). $^{13}$**C NMR** (126 MHz, DMSO-$d_6$, 393K) δ 171.7, 150.2, 142.9, 138.7, 136.9, 134.8, 129.9, 129.7, 128.8, 128.3, 127.8, 127.5, 127.0, 48.4, 47.4, 21.2, 20.9. **Note:** At 393 K, the rotameric signals coalesce and the alkyl carbon adjacent to the amide nitrogen is broadened beyond detection. **HRMS** (ESI) calcd. for ($C_{25}H_{28}N_2NaO_3S$) $[M+Na]^+$: 459.1713, found 459.1724. **IR** (neat): 3226, 3028, 2924, 2863, 1629, 1495, 1452, 1405, 1330, 1159, 1094, 816, 702, 663, 558. **Optical rotation**: $[\alpha]^{26}_{D}$ = 28.5 (c 0.1, $CHCl_3$, 88:12 er). The enantiomeric excess of **4n** was determined by SFC analysis on a Chiralpak ID-3 column ($CO_2$/MeOH with a gradient from 100% to 70% $CO_2$ in 5 min, then maintained at 70% $CO_2$, column temperature 35 °C, flow rate 1.2 mL/min) with retention time 6.70 min (major) and 8.29 min (minor).

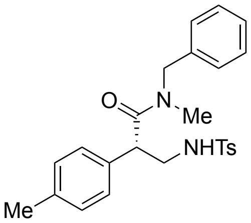


4n

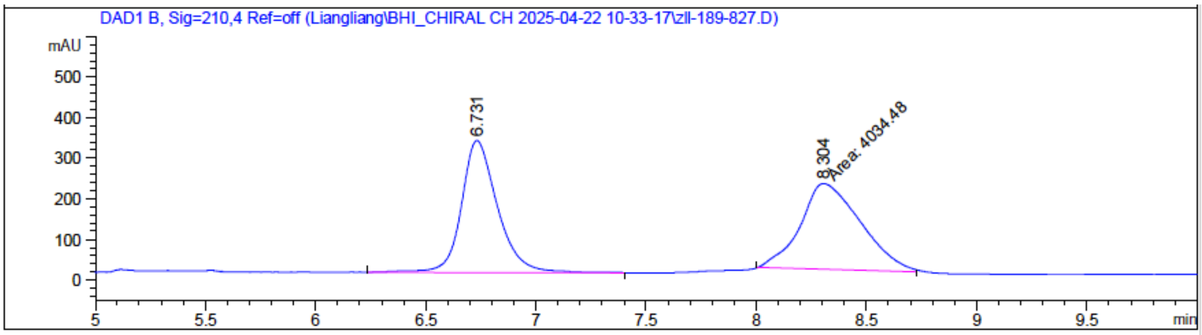


Signal 2: DAD1 B, Sig=210,4 Ref=off

| Peak # | RetTime [min] | Type | Width [min] | Area [mAU*s] | Height [mAU] | Area % |
|---|---|---|---|---|---|---|
| 1 | 6.731 | VV R | 0.1698 | 3728.20435 | 325.31180 | 48.0273 |
| 2 | 8.304 | MM | 0.3179 | 4034.47803 | 211.50108 | 51.9727 |

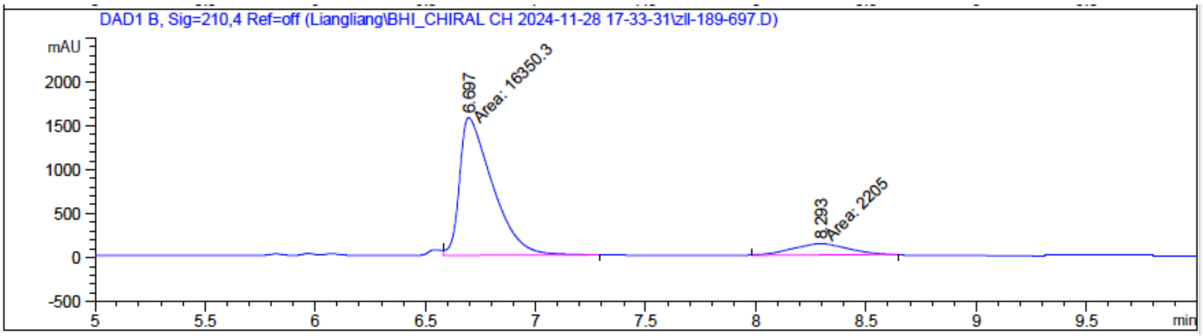


Signal 2: DAD1 B, Sig=210,4 Ref=off

| Peak # | RetTime [min] | Type | Width [min] | Area [mAU*s] | Height [mAU] | Area % |
|---|---|---|---|---|---|---|
| 1 | 6.697 | MM | 0.1737 | 1.63503e4 | 1568.76709 | 88.1166 |
| 2 | 8.293 | MM | 0.2878 | 2205.00220 | 127.67866 | 11.8834 |

## ***Substrates Showing Low or No reactivity***

**Substrates showing little (if any) conversion**

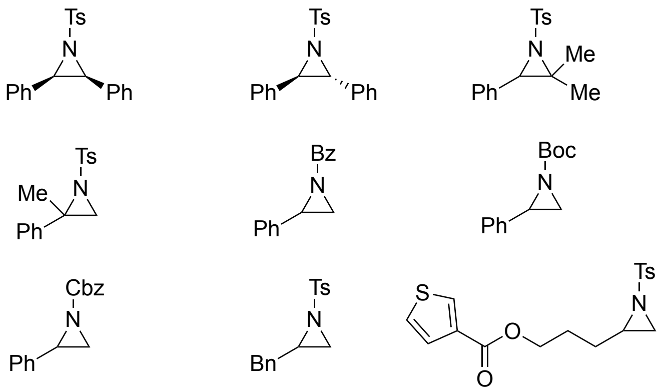

**substrates bearing *N*-alkyl sulfonates**

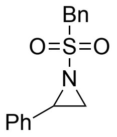

44%, 79% ee

## 5. Scale up Reactions and Synthetic Applications

### 5.1 Scale up Reaction of 1a and 2a

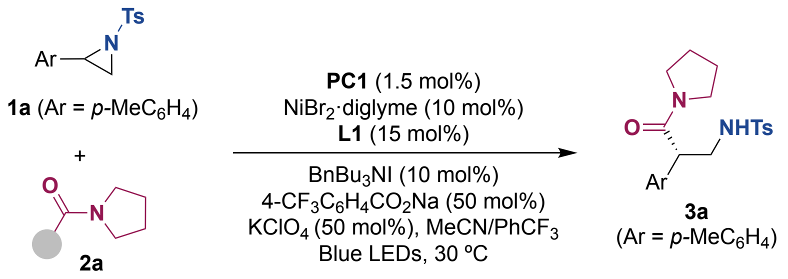


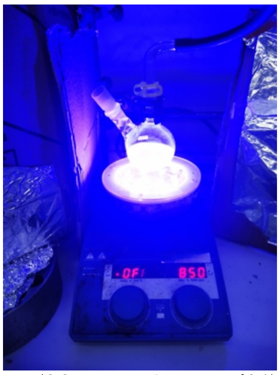

**Procesure:** In a 7 mL vial equipped with a magnetic stir bar, 5-TCzBN (22 mg, 1.5 mol%), $NiBr_2$·diglyme (35 mg, 10 mol%), **L1** (93 mg, 15 mol%), $BnBu_3NI$ (41 mg, 10 mol%), 4-$CF_3C_6H_4CO_2Na$ (106 mg, 0.5 mmol, 0.5 equiv), and $KClO_4$ (69 mg, 0.5 mmol, 0.5 equiv), **2a** (700 mg, 2.0 mmol, 2.0 equiv), **1a** (287 mg, 1.0 mmol, 1.0 equiv) were added. The vial was sealed with an aluminium crimp, evacuated, and backfilled with argon at least three times. Subsequently, anhydrous α,α,α-trifluorotoluene (28 mL) and $CH_3CN$ (7 mL) were added via syringe to the flask containing the Ni/L manifold under an argon atmosphere. The reaction mixture was stirred at 850 rpm and irradiated at 451 nm in a blue LED photoreactor at 30 °C for 40 hours. The mixture was filtered through a short column of silica gel using ethyl acetate as the eluent, concentrated and purified by column chromatography on silica gel (using $^i$PrOAc/Acetone acetate as eluent) affording the title compound as a colorless oil (146.9 mg, 38% yield, 96:4 er).

### 5.2 Cyclization of 3a with $(HCHO)_n$[8]

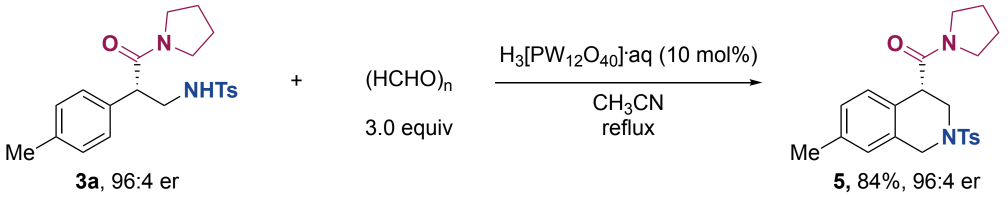


**(*S*)-(7-methyl-2-tosyl-1,2,3,4-tetrahydroisoquinolin-4-yl)(pyrrolidin-1-yl)methanone (5).** In a round bottom flask equipped with a magnetic stir bar and reflux condenser was added a mixture of (*S*)-4-methyl-*N*-(3-oxo-3-(pyrrolidin-1-yl)-2-(*p*-tolyl)propyl)benzenesulfonamide (**3a**, 96:4 er**)** (38.6 mg, 0.10 mmol, 1.0 equiv), paraformaldehyde (9.0 mg, 0.30 mmol, 3.0 equiv), and $H_3[PW_{12}O_{40}]$aq (28.8 mg, 10 mol%) in acetonitrile (2.0 mL) under argon. The mixture was heated to reflux at 100°C for 48 h. The mixture was filtered through a short column of silica gel using ethyl acetate as the eluent, concentrated and purified by column chromatography on silica gel (using $^i$PrOAc/Acetone acetate as eluent) affording the title compound as a colorless oil (33.3 mg, 84% yield, 96:4 er). **$^1$H NMR** (400 MHz, $CDCl_3$) δ 7.72 – 7.68 (m, 2H), 7.33 – 7.28 (m, 2H), 6.95 (dd, *J* = 8.0, 1.9 Hz, 1H), 6.86 – 6.84 (m, 1H), 6.80 (d, *J* = 7.9 Hz, 1H), 4.62 (d, *J* = 14.9 Hz, 1H), 4.16 (dd, *J* = 10.6, 5.2 Hz, 1H), 4.06 (ddd,

$J$ = 11.6, 5.2, 1.5 Hz, 1H), 3.84 (d, $J$ = 14.9 Hz, 1H), 3.64 (t, $J$ = 6.8 Hz, 2H), 3.60 – 3.50 (m, 2H), 3.02 – 2.92 (m, 1H), 2.41 (s, 3H), 2.25 (s, 3H), 2.10 – 1.99 (m, 2H), 1.99 – 1.88 (m, 2H). **$^{13}$C NMR** (101 MHz, $CDCl_3$) δ 170.3, 144.0, 137.0, 133.0, 131.8, 129.9, 129.3, 128.2, 127.9, 127.3, 126.7, 47.8, 47.4, 46.7, 46.1, 43.8, 26.3, 24.5, 21.6, 21.1. **HRMS** (ESI) calcd. for ($C_{22}H_{27}N_2O_3S$) $[M+H]^+$: 399.1737, found 399.1736. **IR** (neat): 2975, 2932, 2873, 1640, 1439, 1343, 1165, 1092, 961, 818, 659, 555. **Optical rotation**: $[\alpha]^{26}_D$ = -22.9 (c 0.2, $CHCl_3$, 96:4 er). The enantiomeric excess of **5** was determined by SFC analysis on a Chiralpak ID-3 column ($CO_2$/MeOH with a gradient from 100% to 50% $CO_2$ in 5 min, then maintained at 50% $CO_2$, column temperature 35 °C, flow rate 1.2 mL/min) with retention time 6.48 min (major) and 9.67 min (minor).

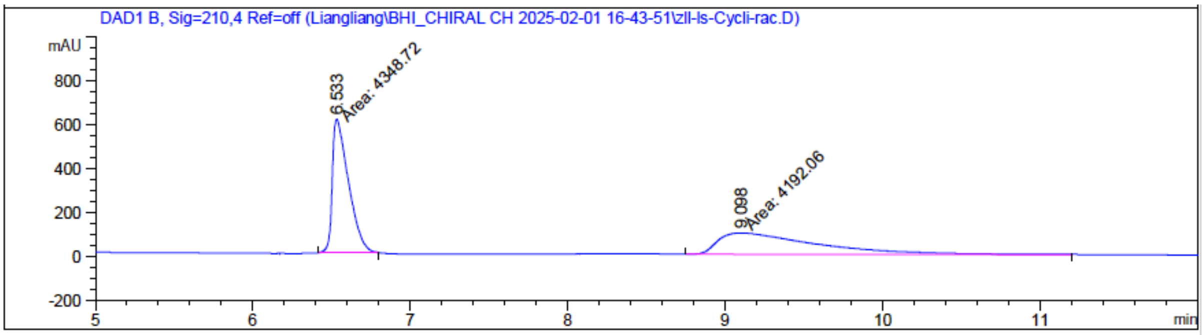


Signal 2: DAD1 B, Sig=210,4 Ref=off

| Peak # | RetTime [min] | Type | Width [min] | Area [mAU*s] | Height [mAU] | Area % |
|---|---|---|---|---|---|---|
| 1 | 6.533 | MM | 0.1194 | 4348.71875 | 607.03705 | 50.9171 |
| 2 | 9.098 | MM | 0.7197 | 4192.06152 | 97.08392 | 49.0829 |

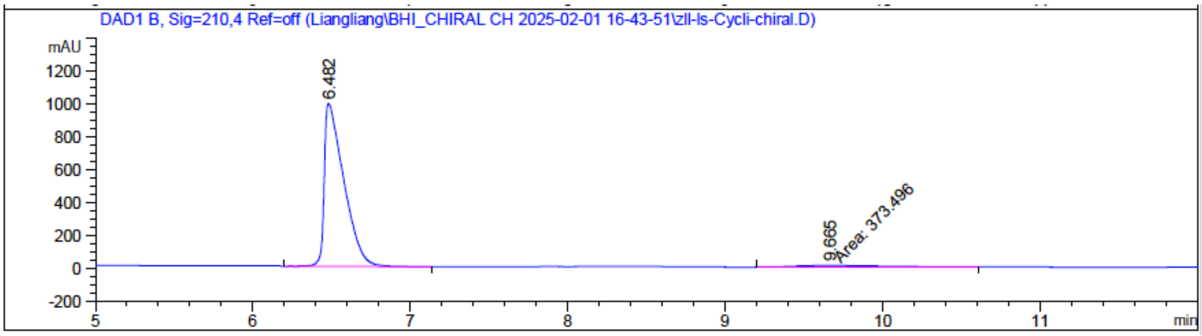


Signal 2: DAD1 B, Sig=210,4 Ref=off

| Peak # | RetTime [min] | Type | Width [min] | Area [mAU*s] | Height [mAU] | Area % |
|---|---|---|---|---|---|---|
| 1 | 6.482 | VB R | 0.1277 | 8529.31543 | 988.58917 | 95.8047 |
| 2 | 9.665 | MM | 0.6367 | 373.49554 | 9.77643 | 4.1953 |

### 5.3 Cyclization of 3a with 5-bromo-6-(bromomethyl)benzo[*d*][1,3]dioxole (S2)[9]

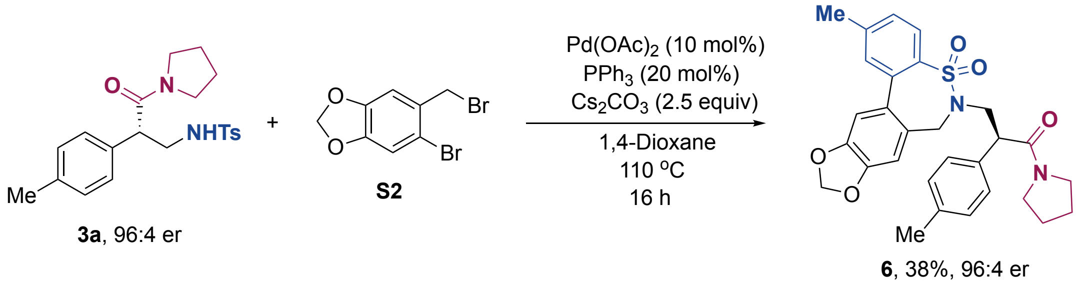


**(*S*)-3-(2-methyl-5,5-dioxido-[1,3]dioxolo[4',5':4,5]benzo[1,2-*d*]benzo[*f*][1,2]thiazepin-6(7*H*)-yl)-1-(pyrrolidin-1-yl)-2-(*p*-tolyl)propan-1-one** (**6**). A screw-cap sealed reaction tube equipped with a magnetic stir bar was charged with (*S*)-4-methyl-*N*-(3-oxo-3-(pyrrolidin-1-yl)-2-(*p*-tolyl)propyl)benzenesulfonamide (**3a**, 96:4 er) (38.6 mg, 0.10 mmol, 1.0 equiv), $Pd(OAc)_2$ (2.2 mg, 10 mol%), $PPh_3$ (5.2 mg, 20 mol%), and $Cs_2CO_3$ (97.7 mg, 0.30 mmol, 3.0 equiv) in dioxane (1.5 mL). The mixture was purged with argon for 5−10 min, and 5-bromo-6-(bromomethyl)benzo[*d*][1,3]dioxole (**S2**) (35.3 mg, 0.12 mmol, 1.2 equiv) was added. The reaction was heated at 110°C for 18 h. The mixture was filtered through a short column of silica gel using ethyl acetate as the eluent, concentrated and purified by column chromatography on silica gel (using DCM/MeOH as eluent), affording the title compound as a colorless oil (19.9 mg, 38% yield, 96:4 er) by using DCM/MeOH (20:1) as eluent. **$^1$H NMR** (400 MHz, $CDCl_3$) δ 7.82 (d, *J* = 7.9 Hz, 1H), 7.30 – 7.26 (m, 4H), 7.15 (d, *J* = 7.9 Hz, 2H), 6.88 (s, 1H), 6.72 (s, 1H), 6.01 (dd, *J* = 10.8, 1.5 Hz, 2H), 4.17 – 4.10 (m, 2H), 3.77 – 3.68 (m, 1H), 3.63 – 3.51 (m, 2H), 3.50 – 3.40 (m, 3H), 3.21 – 3.13 (m, 1H), 2.46 (s, 3H), 2.34 (s, 3H), 1.94 – 1.71 (m, 4H). **$^{13}$C NMR** (101 MHz, $CDCl_3$) δ 170.4, 148.5, 148.1, 143.8, 139.2, 137.3, 134.8, 134.0, 133.5, 130.3, 129.7, 128.7, 128.4, 128.1, 126.8, 110.9, 108.7, 101.7, 56.0, 55.4, 52.7, 46.4, 46.2, 26.0, 24.3, 21.7, 21.2. **HRMS** (ESI) calcd. for ($C_{29}H_{31}N_2O_5S$) $[M+H]^+$: 519.1948, found 519.1939. **IR** (neat): 2971, 2922, 2876, 1634, 1505, 1487, 1445, 1336, 1227, 1161, 1038, 929, 756, 552. **Optical rotation**: $[\alpha]^{26}_D$ = 40.2 (c 0.1, $CHCl_3$, 96:4 er). The enantiomeric excess of **6** was determined by SFC analysis on a Chiralpak ID-3 column ($CO_2$/MeOH with a gradient from 100% to 70% $CO_2$ in 5 min, then maintained at 70% $CO_2$, column temperature 35 °C, flow rate 1.2 mL/min) with retention time 10.15 min (major) and 11.31 min (minor).

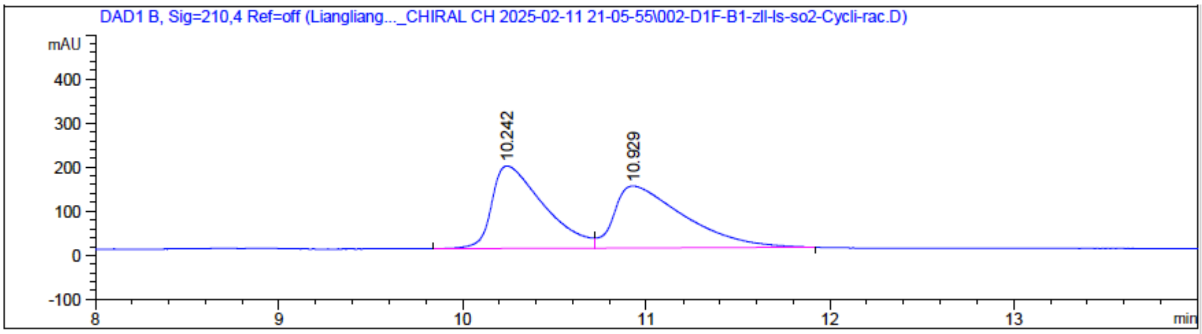

Signal 2: DAD1 B, Sig=210,4 Ref=off

| Peak # | RetTime [min] | Type | Width [min] | Area [mAU*s] | Height [mAU] | Area % |
|---|---|---|---|---|---|---|
| 1 | 10.242 | BV | 0.2750 | 3679.72314 | 187.49483 | 49.6041 |
| 2 | 10.929 | VV R | 0.3549 | 3738.46655 | 140.73270 | 50.3959 |

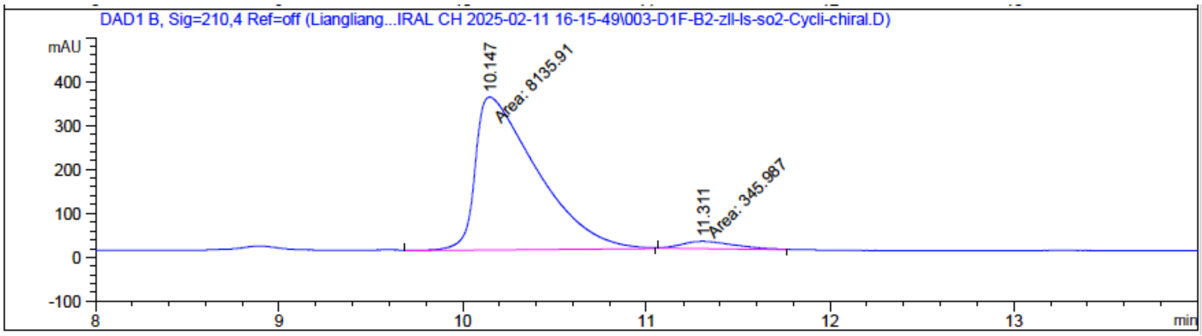


Signal 2: DAD1 B, Sig=210,4 Ref=off

| Peak # | RetTime [min] | Type | Width [min] | Area [mAU*s] | Height [mAU] | Area % |
|---|---|---|---|---|---|---|
| 1 | 10.147 | MM | 0.3896 | 8135.90869 | 348.04761 | 95.9209 |
| 2 | 11.311 | MM | 0.3393 | 345.98734 | 16.99432 | 4.0791 |

### 5.4 Hydrogenation of 3a with $LiAlH_4$

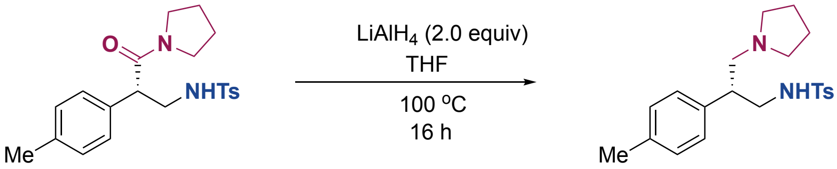


**3a**, 92% ee  **7**, 83%, 96:4 er

**(*R*)-4-methyl-*N*-(3-(pyrrolidin-1-yl)-2-(*p*-tolyl)propyl)benzenesulfonamide** (**7**). In a tube equipped with a magnetic stirring bar, (*S*)-4-methyl-*N*-(3-oxo-3-(pyrrolidin-1-yl)-2-(*p*-tolyl)propyl)benzenesulfonamide (**3a**, 96:4 er) (38.6 mg, 0.1 mmol, 1 equiv) was dissolved in THF (1.5 mL, 0.07 M) under a nitrogen atmosphere. After the solution was cooled to 0 °C, $LiAlH_4$ (2.4 M in THF, 67 µL, 0.16 mmol, 1.6 equiv) was added dropwise over 10 min. The mixture was heated at 100 °C for 12 h. The reaction was diluted with THF (5 mL) and quenched with NaOH 15% aq. solution (0.5 mL). The aqueous phase was extracted with EtOAc (3 x 10 mL) and the combined organic layers were washed with brine, dried over anhydrous $Na2SO_4$, and concentrated under reduced pressure. The mixture was filtered through a short column of silica gel using ethyl acetate as the eluent, concentrated and purified by column chromatography on silica gel (using DCM/MeOH as eluent), affording the title compound as a colorless oil( 30.8 mg, 83% yield, 96:4 er) by using DCM/MeOH (20:1) as eluent. **$^1$H NMR** (400 MHz, $CDCl_3$) δ 7.74 – 7.70 (m, 2H), 7.30 (d, *J* = 8.0 Hz, 2H), 7.08 (d, *J* = 7.8 Hz, 2H), 6.96 – 6.93 (m, 2H), 3.30 – 3.25 (m, 1H), 3.17 – 3.11 (m, 1H), 3.03 – 2.94 (m, 2H), 2.71 – 2.62 (m, 2H), 2.47 – 2.39 (m, 6H), 2.30 (s, 3H), 1.85 – 1.76 (m, 4H). **$^{13}$C NMR** (101 MHz, $CDCl_3$) δ 143.1, 137.9,

137.5, 136.9, 129.7, 129.5, 127.17, 127.16, 62.8, 54.3, 50.5, 41.9, 23.6, 21.6, 21.1. **HRMS** (ESI) calcd. for ($C_{21}H_{29}N_2O_2S$) $[M+H]^+$: 373.1944, found 373.1950. **IR** (neat): 2962, 2926, 2875, 2802, 1958, 1515, 1449, 1327, 1159, 1094, 815, 660, 549. **Optical rotation**: $[\alpha]^{26}{}_D$ = 23.4 (c 0.3, $CHCl_3$, 96:4 er). The enantiomeric excess of **7** was determined by SFC analysis on a Chiralpak IE column ($CO_2$/EtOH = 70:30, ***Note:*** EtOH was mixed with 0.3% diethylamine, column temperature 35 °C, flow rate 2 mL/min) with retention time 4.11 min (major) and 4.73 min (minor).

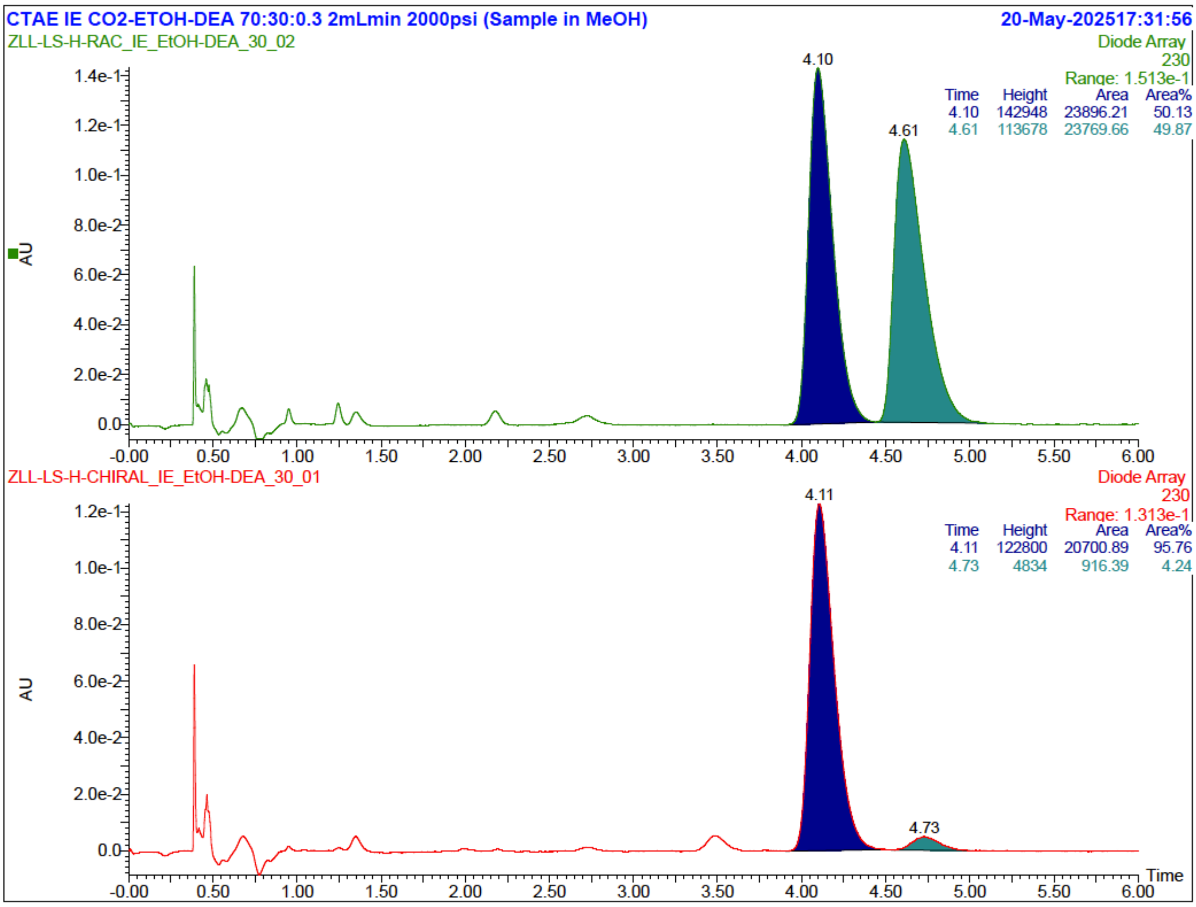

## 6. Mechanistic Experiments

### 6.1 Evidence for stereoconvergent scenarios

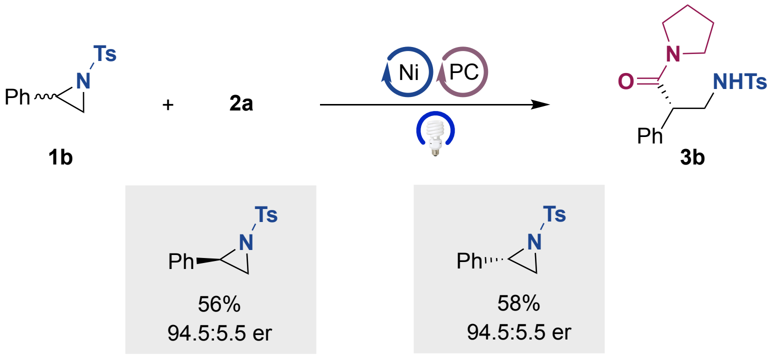


Following the General Procedure, 2-phenyl-1-tosylaziridine (**1b**) (41.0 mg, 0.15 mmol), and diethyl 2,6-dimethyl-4-(pyrrolidine-1-carbonyl)-1,4-dihydropyridine-3,5-dicarboxylate (**2a**) (78.8 mg, 0.225 mmol) were used, affording the title compound as a white solid by using $^{i}$PrOAc/Acetone (20:1) as eluent. From (*R*)-**1b**: 20.8 mg, 56% yield, 94.5:5.5 er; from (*S*)-**1b**: 21.4 mg, 58% yield, 94.5:5.5 er. The enantiomeric excess of **3b** was determined by SFC analysis on a Chiralpak ID-3 column ($CO_2$/MeOH with a gradient from 100% to 70% $CO_2$ in 5 min, then maintained at 70% $CO_2$, column temperature 35 °C, flow rate 1.2 mL/min).

*With dtbpy as the ligand*:

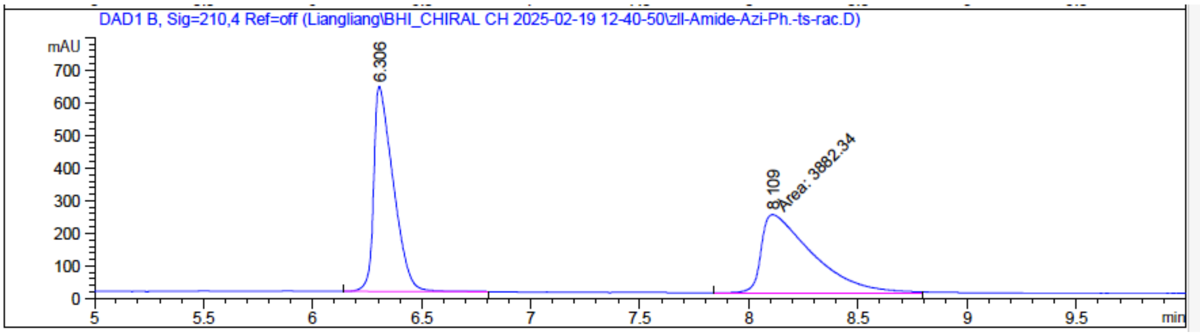


Signal 2: DAD1 B, Sig=210,4 Ref=off

| Peak # | RetTime [min] | Type | Width [min] | Area [mAU*s] | Height [mAU] | Area % |
|---|---|---|---|---|---|---|
| 1 | 6.306 | VV R | 0.0909 | 3925.38721 | 628.19049 | 50.2757 |
| 2 | 8.109 | MF | 0.2705 | 3882.34229 | 239.24518 | 49.7243 |

From (*R*)-**1b**:

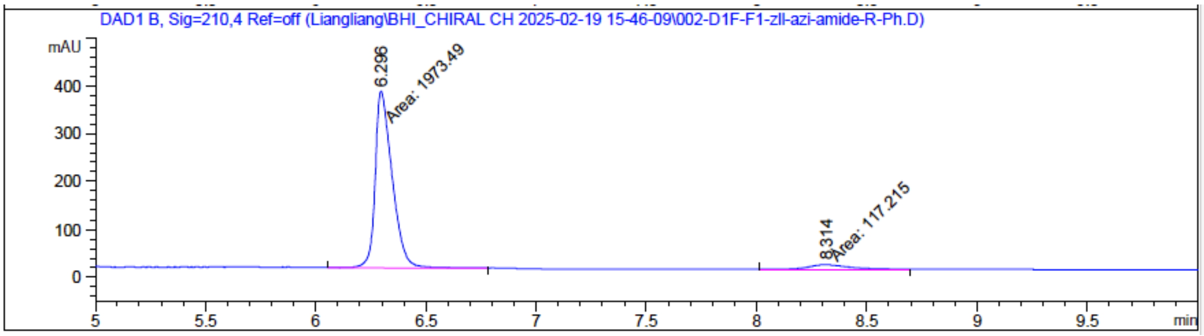

Signal 2: DAD1 B, Sig=210,4 Ref=off

| Peak # | RetTime [min] | Type | Width [min] | Area [mAU*s] | Height [mAU] | Area % |
|---|---|---|---|---|---|---|
| 1 | 6.296 | MM | 0.0893 | 1973.48779 | 368.47891 | 94.3935 |
| 2 | 8.314 | MM | 0.2075 | 117.21459 | 9.41691 | 5.6065 |

From (*S*)-**1b**:

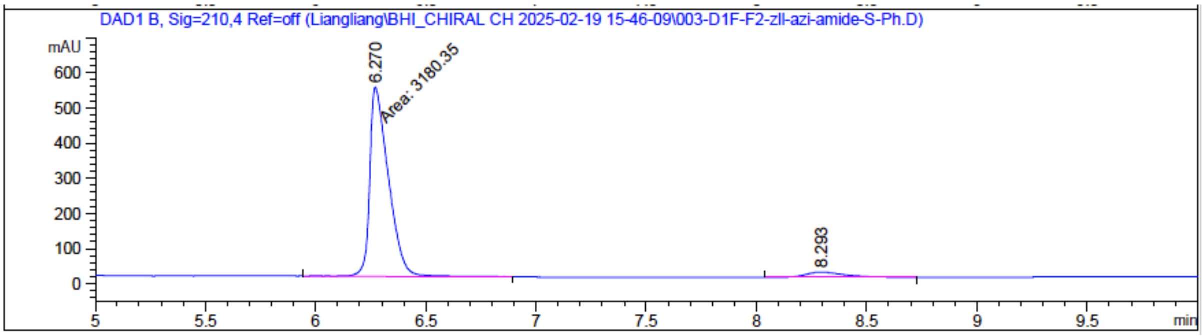


Signal 2: DAD1 B, Sig=210,4 Ref=off

| Peak # | RetTime [min] | Type | Width [min] | Area [mAU*s] | Height [mAU] | Area % |
|---|---|---|---|---|---|---|
| 1 | 6.270 | MM | 0.0984 | 3180.34546 | 538.69946 | 94.3051 |
| 2 | 8.293 | VV R | 0.1641 | 192.05394 | 15.23032 | 5.6949 |

## 6.2 Intermediacy of open-shell species

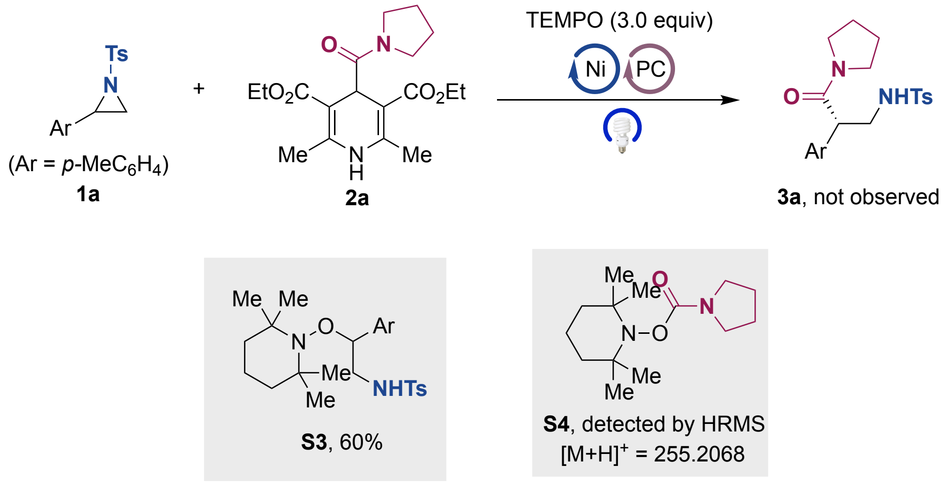


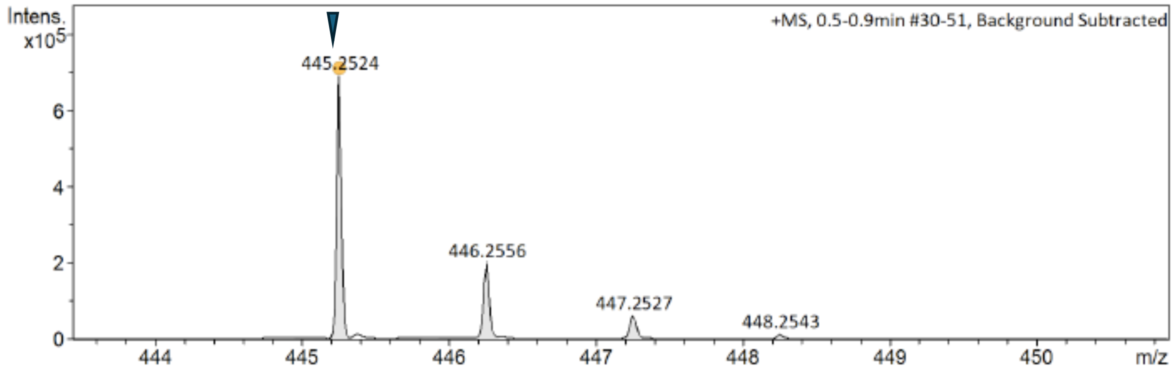


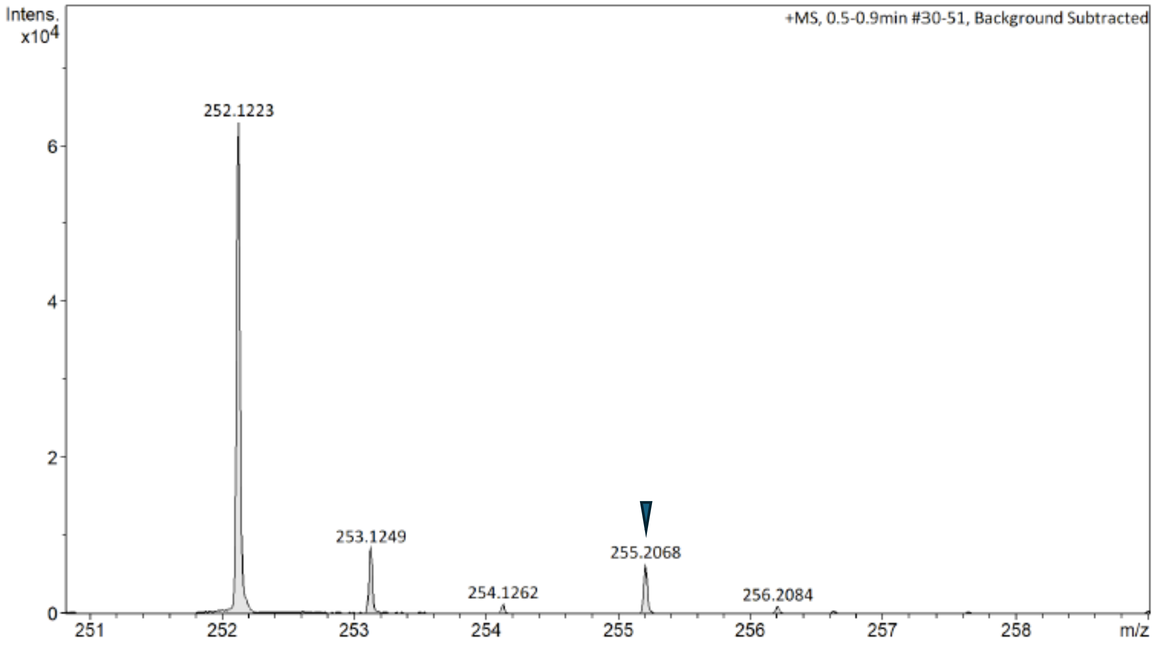

**4-methyl-*N*-(2-((2,2,6,6-tetramethylpiperidin-1-yl)oxy)-2-(*p*-tolyl)ethyl)benzenesulfonamide (S3).**

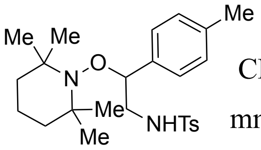

**S3**

In a 7 mL vial equipped with a magnetic stir bar, 5-TCzBN (2.2 mg, 1.5 mol%), $NiBr_2$·diglyme (3.5 mg, 10 mol%), **L1** (9.3 mg, 15 mol%), $BnBu_3NI$ (4.0 mg, 10 mol%), 4-$CF_3C_6H_4CO_2Na$ (10.6 mg, 0.050 mmol, 0.50 equiv), and $KClO_4$ (6.9 mg, 0.050 mmol, 0.50 equiv), **2a** (70.0 mg, 0.20 mmol, 2.0 equiv), **1a** (28.7 mg, 0.10 mmol, 1.0 equiv) and TEMPO (54.7 mg, 0.35 mmol, 3.5 equiv) were added. The vial was sealed with an aluminium crimp, evacuated, and backfilled with argon at least three times. Subsequently, anhydrous α,α,α-trifluorotoluene (2.8 mL) and $CH_3CN$ (0.7 mL) were added via syringe to the flask containing the Ni/L manifold under an argon atmosphere. The reaction mixture was stirred at 850 rpm and irradiated at 451 nm in a blue LED photoreactor at 30 °C for 16 hours. The reaction mixture was analyzed by HRMS. It was found that the reaction was completely suppressed by adding TEMPO (3.5 equiv). Interestingly, both **S3** and **S4** were observed, indicating the generation of open-shell species in both the 4-carbamoyl 1,4-dihydropyridine and the aziridine. The mixture was then filtered through a short column of silica gel using ethyl acetate as the eluent. The filtrate was concentrated and subsequently purified by column chromatography on silica gel, obtaining the title compound as a colorless oil (26.7 mg, 60% yield) by using Hexane/EtOAc (10/1-5/1) as eluent. **$^1$H NMR** (400 MHz, $CDCl_3$) δ 7.67 – 7.64 (m, 2H), 7.29 – 7.23 (m, 2H), 7.14 – 7.05 (m, 4H), 5.69 (t, *J* = 5.9 Hz, 1H), 4.90 (dd, *J* = 7.0, 5.3 Hz, 1H), 3.56 – 3.45 (m, 1H), 3.28 – 3.17 (m, 1H), 2.42 (s, 3H), 2.33 (s, 3H), 1.53 – 1.31 (m, 6H), 1.14 – 0.90 (m, 12H). **$^{13}$C NMR** (101 MHz, $CDCl_3$) δ 143.3, 137.9, 137.2, 136.8, 129.7, 129.2, 127.2, 127.1, 83.1, 60.2, 48.8, 40.5, 34.2, 33.4, 21.7, 21.3, 20.8, 17.2. **HRMS** (ESI) calcd. for ($C_{25}H_{37}N_2O_3S$) $[M+H]^+$: 445.2519, found 445.2524. **IR** (neat): 3285, 2972, 2928, 1599, 1453, 1331, 1161, 1094, 814, 662, 552.

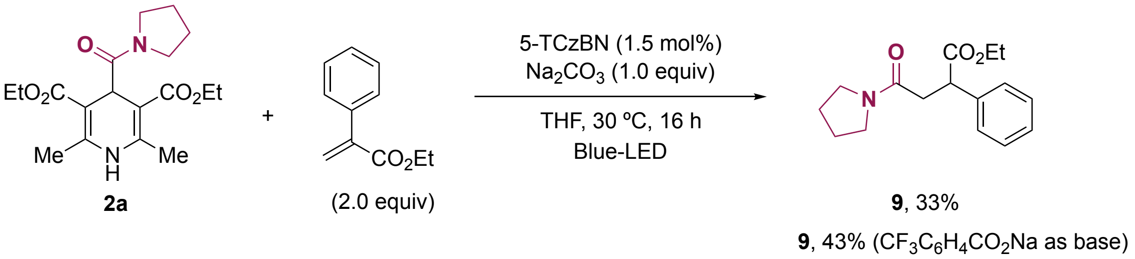


**ethyl 4-oxo-2-phenyl-4-(pyrrolidin-1-yl)butanoate (9).** In a glovebox, a 7 mL vial equipped with a magnetic stir bar was charged with 5-TCzBN (2.2 mg, 1.5 mol%), $Na_2CO_3$ or 4-$CF_3C_6H_4COONa$ (10.6 mg or 21.2 mg, 0.10 mmol, 1.0 equiv), **2a** (70.0 mg, 0.20 mmol, 2.0 equiv), and ethyl 2-phenylacrylate (35.2 mg, 0.20 mmol, 2.0 equiv). Anhydrous THF (3.0 mL) was then added via syringe, and the vial was sealed with an aluminum crimp. The vial was removed from the glovebox, and the reaction mixture was stirred at 850 rpm while being irradiated at 451 nm using a blue LED photoreactor at 30 °C for 16 hours. Upon completion, the reaction was quenched with 1 M HCl (3.0 mL) and extracted with ethyl acetate (3 × 5.0 mL). The combined organic layers were dried over $Na_2SO_4$ and concentrated under reduced pressure. The crude

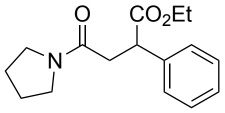

**9**

product was purified by silica gel column chromatography, obtaining the title compound as a colorless oil (9.1 mg or 11.8 mg, 33% yield or 43% yield) by using Hexane/EtOAc (2/1) as eluent. **$^{1}$H NMR** (400 MHz, $CDCl_3$) δ 7.35 – 7.23 (m, 5H), 4.26 – 4.04 (m, 3H), 3.54 – 3.37 (m, 3H), 3.33 – 3.23 (m, 1H), 3.15 (dd, $J$ = 16.2, 10.5 Hz, 1H), 2.51 (dd, $J$ = 16.2, 4.4 Hz, 1H), 1.99 – 1.74 (m, 4H), 1.19 (t, $J$ = 7.1 Hz, 3H). **$^{13}$C NMR** (101 MHz, $CDCl_3$) δ 173.9, 169.2, 139.0, 128.9, 127.9, 127.5, 61.1, 47.4, 46.6, 45.8, 39.0, 26.2, 24.5, 14.2. **HRMS** (ESI) calcd. for ($C_{16}H_{13}NO_3$) $[M+H]^+$: 276.1594, found 276.1595.

### 6.3 Utilization of β-halo sulfonamides as substrates

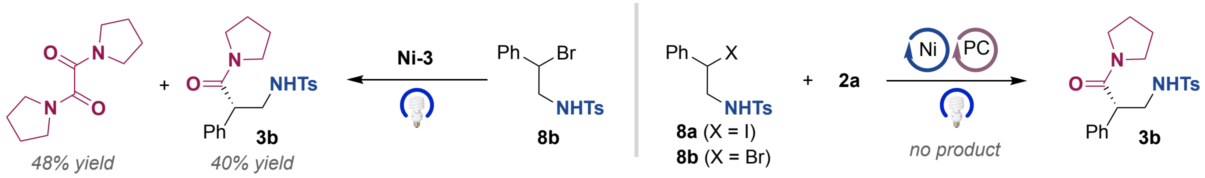


The carbamoylation of β-halo sulfonamides (**8a** and **8b**) was examined under both catalytic and stoichiometric conditions:

*Standard photocatalytic conditions*: In a 7 mL vial equipped with a magnetic stir bar, 5-TCzBN (2.2 mg, 1.5 mol%), $NiBr_2$·diglyme (3.5 mg, 10 mol%), **L1** (9.3 mg, 15 mol%), $BnBu_3NI$ (4.0 mg, 10 mol%), 4-$CF_3C_6H_4CO_2Na$ (10.6 mg, 0.050 mmol, 0.50 equiv), and $KClO_4$ (6.9 mg, 0.050 mmol, 0.50 equiv), *N*-(2-iodo-2-phenylethyl)-4-methylbenzenesulfonamide or *N*-(2-bromo-2-phenylethyl)-4-methylbenzenesulfonamide (**8a** or **8b**, 35.4 mg or 40.1 mg, 0.10 mmol, 1.0 equiv), and **2a** (70.0 mg, 0.20 mmol, 2.0 equiv) were added. The vial was sealed with an aluminium crimp, evacuated, and backfilled with argon at least three times. Subsequently, anhydrous α,α,α-trifluorotoluene (2.8 mL) and $CH_3CN$ (0.7 mL) were added via syringe to the flask containing the Ni/L manifold under an argon atmosphere. The reaction mixture was stirred at 850 rpm and irradiated at 451 nm in a blue LED photoreactor at 30 °C for 16 hours. Upon completion, the mixture was filtered through a short column of silica gel using ethyl acetate as the eluent. An internal standard (dodecane) was introduced to determine the GC yield.

*Stoichiometric conditions:* stoichiometric experiment was next conducted employing isolated **Ni-3**–as a carbamoyl-radical precursor–and aryl sulfonamide **8b**. Thus, inside a nitrogen-filled glove box, a 5 mL vial equipped with a magnetic stirring bar was charged with 11.6 mg of **Ni-3** (0.025 mmol, 1 equiv), 17.1 mg of **8b** (0.050 mmol, 2 equiv) and 2 mL of THF at room temperature. The vial was sealed with an aluminum crimp, removed from the glovebox and irradiated at 451 nm in a blue LED photoreactor at 30 °C for 16 hours. Upon completion, the mixture was exposed to air, filtered through a short column of silica gel using ethyl acetate as the eluent, and the solvent removed under reduced pressure. Yields were determined by $^{1}$H NMR with 1,3,5-trimethoxybenzene as internal standard, affording 40% of the desired product **3b** together with carbamoyl radical recombination in 48% yield.

### 6.4 Non-linear effect studies

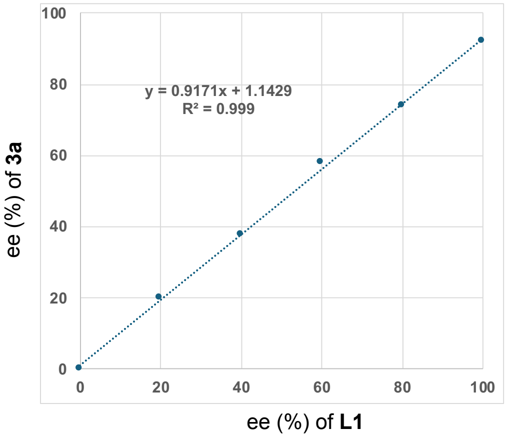


***Note:*** we investigated the nonlinear effect to assess the nature of the active catalyst. A perfect linear relationship was observed, suggesting that a monomeric nickel complex bearing a single ligand is involved in the enantioselectivity-determining step.

### 6.5 Reactivity of Ni-1 (L1NiBr$_2$) complex

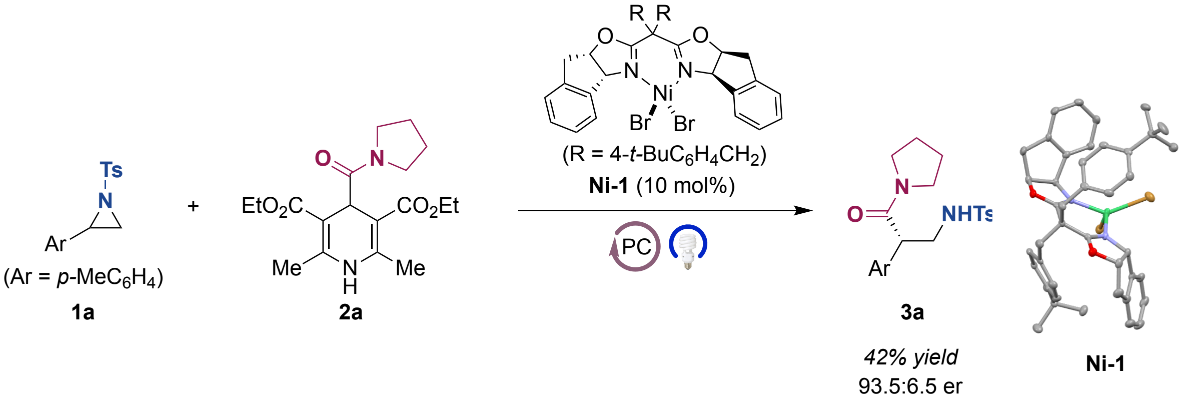


*Procedure for the Synthesis of Ni-I Complex*: In an argon-filled glovebox, $NiBr_2$·DME (155.0 mg, 0.50 mmol, 1.0 equiv), ligand **L1** (311.4 mg, 0.50 mmol, 1.0 equiv), and THF (5 mL) were added to a 10 mL Schlenk flask. The reaction mixture was stirred at 40 °C for 4 h. Afterwards, the mixture was concentrated under vacuum to afford a purple-red solid.

In a 7 mL vial equipped with a magnetic stir bar, 5-TCzBN (2.2 mg, 1.5 mol%), **Ni-1** (8.4 mg, 10 mol%), $BnBu_3NI$ (4.0 mg, 10 mol%), 4-$CF_3C_6H_4CO_2Na$ (10.6 mg, 0.050 mmol, 0.50 equiv), and $KClO_4$ (6.9 mg, 0.050 mmol, 0.50 equiv), **1a** (28.7 mg, 0.10 mmol, 1.0 equiv), and **2a** (70.0 mg, 0.20 mmol, 2.0 equiv) were added. The vial was sealed with an aluminium crimp, evacuated, and backfilled with argon at least three times. Subsequently, anhydrous α,α,α-trifluorotoluene (2.8 mL) and $CH_3CN$ (0.7 mL) were added via syringe to the flask containing the Ni/L manifold under an argon atmosphere. The reaction mixture was stirred at 850 rpm and irradiated at 451 nm in a blue LED photoreactor at 30 °C for 16 hours. The mixture was then filtered through a short column of silica gel using ethyl acetate as

the eluent. The filtrate was concentrated and subsequently purified by column chromatography on silica gel. The title compound **3a** was obtained as a white solid (16.2 mg, 42% yield, 93.5:6.5 er) by using $^{i}$PrOAc/Acetone (20:1) as eluent. The enantiomeric excess of **3a** was determined by SFC analysis on a Chiralpak ID-3 column ($CO_2$/MeOH with a gradient from 100% to 70% $CO_2$ in 5 min, then maintained at 70% $CO_2$, column temperature 35 °C, flow rate 1.2 mL/min) with retention time 6.41 min (major) and 8.69 min (minor).

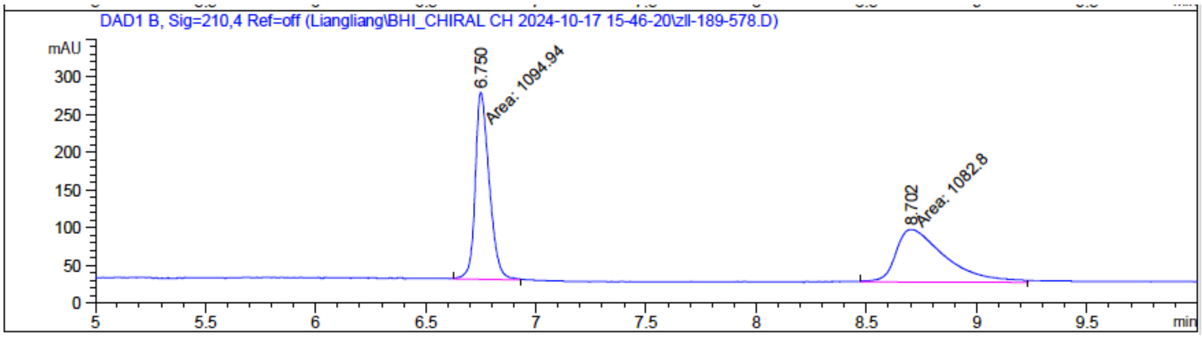


Signal 2: DAD1 B, Sig=210,4 Ref=off

| Peak # | RetTime [min] | Type | Width [min] | Area [mAU*s] | Height [mAU] | Area % |
|---|---|---|---|---|---|---|
| 1 | 6.750 | MM | 0.0736 | 1094.93738 | 248.06291 | 50.2786 |
| 2 | 8.702 | MF | 0.2587 | 1082.80481 | 69.75238 | 49.7214 |

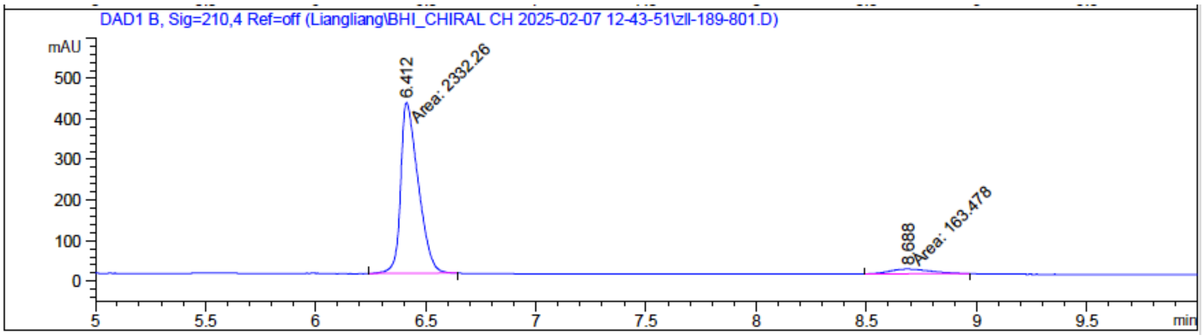


Signal 2: DAD1 B, Sig=210,4 Ref=off

| Peak # | RetTime [min] | Type | Width [min] | Area [mAU*s] | Height [mAU] | Area % |
|---|---|---|---|---|---|---|
| 1 | 6.412 | MM | 0.0923 | 2332.25977 | 421.17618 | 93.4497 |
| 2 | 8.688 | MM | 0.2270 | 163.47795 | 12.00510 | 6.5503 |

## 6.6 Cyclic voltammetry (CV) analysis

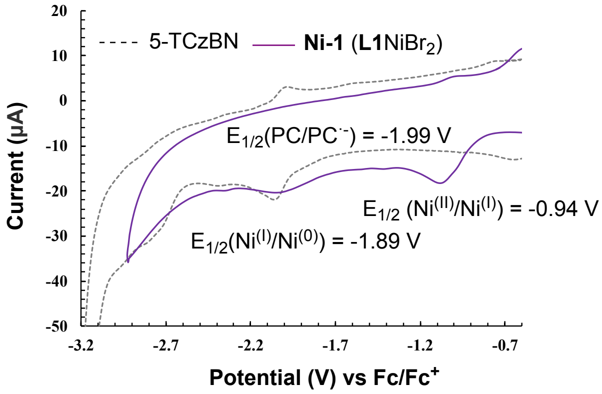


Cyclic voltammetry (CV) data of the mixture of **Ni-1** (**L1**$NiBr_2$) and 5-TCzBN. Solvent = THF, temperature = 25 °C, concentration of [Ni] = 1.0 mM and [PC] = 1.0 mM. Fc was added as internal standard. Electrolyte = $TBABF_6$ (0.1 M), scan rate = 100 mV/s. $E_{1/2}(Ni^{(II)}/Ni^{(I)})$ = -0.94 V vs $Fc/Fc^+$ and $E_{1/2}(Ni^{(I)}/Ni^{(0)})$ = -1.89 V vs $Fc/Fc^+$. $E_{1/2}(PC/PC^{\bullet-})$ = –1.99 V vs $Fc/Fc^+$.

***Note:*** we found the presence of quasi-reversible peaks for $Ni^{(II)}/Ni^{(I)}$, irreversible peak for $Ni^{(I)}/Ni^{(0)}$, and quasi-reversible peaks for $PC/PC^{\bullet-}$. This experiment suggests that the photocatalyst 5-TCzBN can promote the single-electron transfer reduction en route to Ni(I), and is also capable to generate Ni(0).

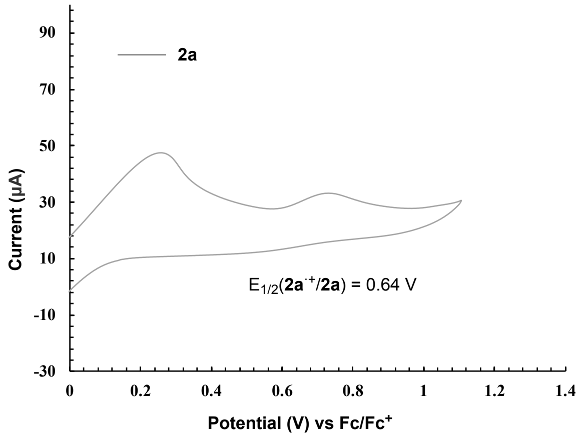


Cyclic voltammetry (CV) data of **2a**. Solvent = THF, temperature = 25 °C, concentration of **2a** = 1.0 mM. Fc was added as internal standard. Electrolyte = $TBABF_6$ (0.1 M), scan rate = 100 mV/s. $E_{1/2}$(**2a**$^{\bullet+}$/**2a**) = +0.64 V vs $Fc/Fc^+$.

***Note:*** we found the presence of quasi-reversible peaks for **2a**$^{\bullet+}$/**2a**. This finding indicates that the photocatalyst 5-TCzBN ($E_{1/2}(PC^*/PC^{\bullet-})$ = +0.82 V vs $Fc/Fc^+$) possesses a sufficiently high oxidation potential to oxidize carbamoyl DHP **2a**. The excited-state oxidation potential was estimated using the equation $E_{1/2}(PC^*/PC^{\bullet-}) = E_{1/2}(PC/PC^{\bullet-}) + E_{0\text{-}0}$. $E_{0\text{-}0} = hc/\lambda$. $E_{0\text{-}0}$, the zero-zero vibrational state excitation energy, was estimated using the medium wavelengths between the lowest fluorescence excitation peak (excitation $\lambda_{max}$ = 339 nm) and the fluorescence peak (emission $\lambda_{max}$ = 543 nm). [10]

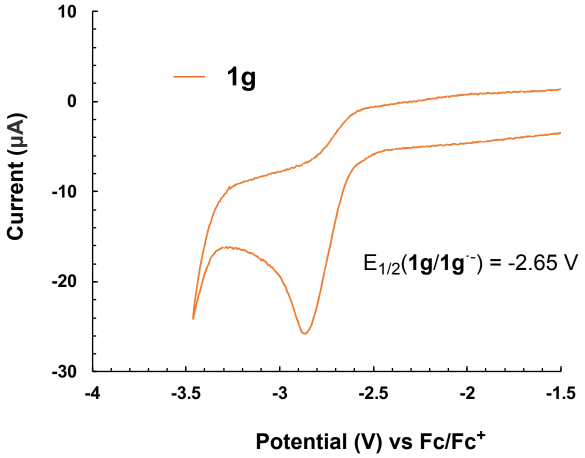


Cyclic voltammetry (CV) data of the mixture of **1g**. Solvent = THF, temperature = 25 °C, concentration of [**1g**] = 1.0 mM. Fc was added as internal standard. Electrolyte = TBABF$_6$ (0.1 M), scan rate = 100 mV/s. $E_{1/2}$(**1g**/**1g**$^{\bullet-}$) = –2.65 V vs Fc/Fc$^+$.

***Note:*** we found the presence of quasi-reversible peaks for **1g**/**1g**$^{\bullet-}$. This experiment suggests that a direct SET from either Ni or **PC1** to the aziridine can be excluded based on electrochemical data ($E_{1/2}$ [Ni(II)/Ni(I)] = –0.94 V and $E_{1/2}$ [Ni(I)/Ni(0)] = –1.89 V vs Fc$^+$/Fc; $E_{1/2}$ [**1g**/**1g**$^{\bullet-}$] = –2.65 V vs Fc$^+$/Fc; $E_{1/2}$(PC/PC$^{\bullet-}$) = –1.99 V vs Fc/Fc$^+$).

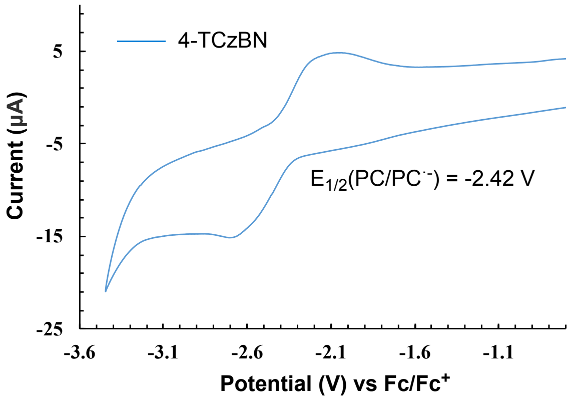


Cyclic voltammetry (CV) data of the mixture of 4-TCzBN. Solvent = THF, temperature = 25 °C, concentration of [PC] = 1.0 mM. Fc was added as internal standard. Electrolyte = TBABF$_6$ (0.1 M), scan rate = 100 mV/s. $E_{1/2}$(PC/PC$^{\bullet-}$) = –2.42 V vs Fc/Fc$^+$.

***Note:*** we found the presence of quasi-reversible peaks for PC/PC$^{\bullet-}$. This experiment suggests that the photocatalyst 4-TCzBN can promote the single-electron transfer reduction en route to Ni(I), and is also capable to generate Ni(0).

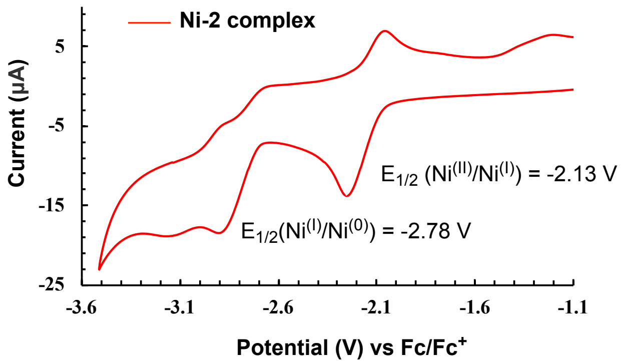


Cyclic voltammetry (CV) data of the mixture of **Ni-2** complex. Solvent = THF, temperature = 25 °C, concentration of [Ni] = 1.0 mM and [PC] = 1.0 mM. Fc was added as internal standard. Electrolyte = $TBABF_6$ (0.1 M), scan rate = 100 mV/s. $E_{1/2}(Ni^{(II)}/Ni^{(I)})$ = -2.13 V vs $Fc/Fc^+$ and $E_{1/2}(Ni^{(I)}/Ni^{(0)})$ = -2.78 V vs $Fc/Fc^+$.

***Note:*** we observed quasi-reversible redox peaks corresponding to the Ni(II)/Ni(I) and Ni(I)/Ni(0) couples. These results suggest that the photocatalyst 5-TCzBN [$E_{1/2}(PC/PC^{\bullet-})$ = –1.99 V vs $Fc/Fc^+$] is not sufficiently reducing to promote the single-electron transfer required to generate Ni(I).

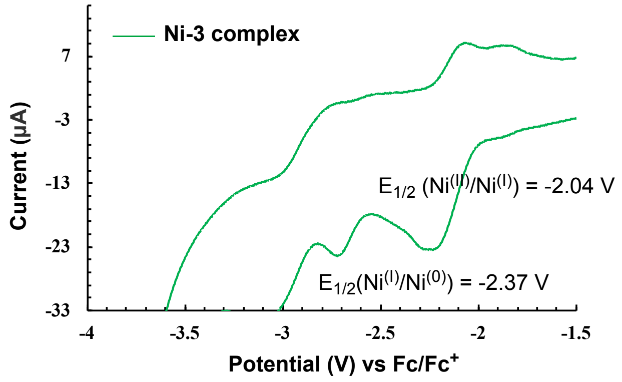


Cyclic voltammetry (CV) data of the mixture of **Ni-3** complex. Solvent = THF, temperature = 25 °C, concentration of [Ni] = 1.0 mM and [PC] = 1.0 mM. Fc was added as internal standard. Electrolyte = $TBABF_6$ (0.1 M), scan rate = 100 mV/s. $E_{1/2}(Ni^{(II)}/Ni^{(I)})$ = -2.04 V vs $Fc/Fc^+$ and $E_{1/2}(Ni^{(I)}/Ni^{(0)})$ = -2.37 V vs $Fc/Fc^+$.

***Note:*** we observed quasi-reversible redox peaks corresponding to the Ni(II)/Ni(I) and Ni(I)/Ni(0) couples. These results suggest that the photocatalyst 5-TCzBN [$E_{1/2}(PC/PC^{\bullet-})$ = –1.99 V vs $Fc/Fc^+$] is not sufficiently reducing to promote the single-electron transfer required to generate Ni(I).

### 6.7 Fluorescence Quenching Studies

A 1.0 x $10^{-6}$ M solution of PC (5-TCzBN) in anhydrous and degassed DCM was prepared in a nitrogen filled glovebox. PC (5-TCzBN) solution (4.0 mL) was transferred to a 4 mL quartz cuvette (path length: $l$ = 1.0 cm) under an atmosphere of nitrogen, where upon irradiation at 451 nm an emission at maximum at 558 nm was observed. Aliquots of the quencher solutions were added to the solution of PC (5-

TCzBN) contained in a quartz cuvette (path length: $l$ = 1.0 cm) under an atmosphere of nitrogen, followed by recording of the emission spectra.

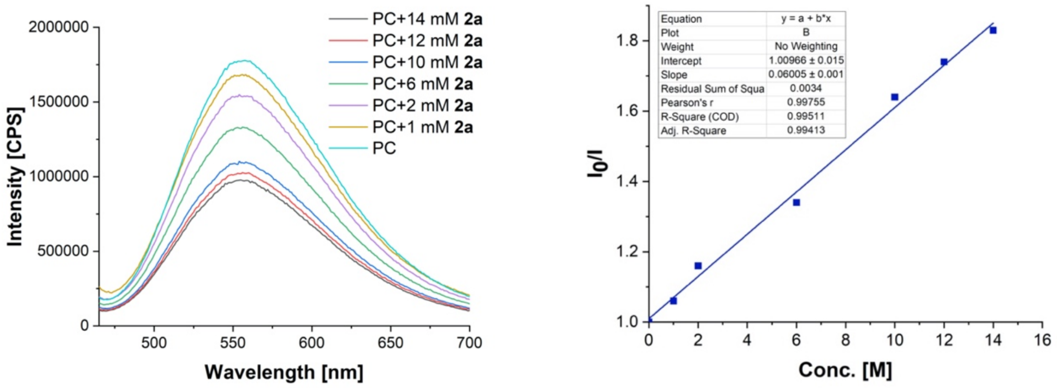


**Figure S1.** Fluorescence quenching studies of PC (5-TCzBN) with **2a**

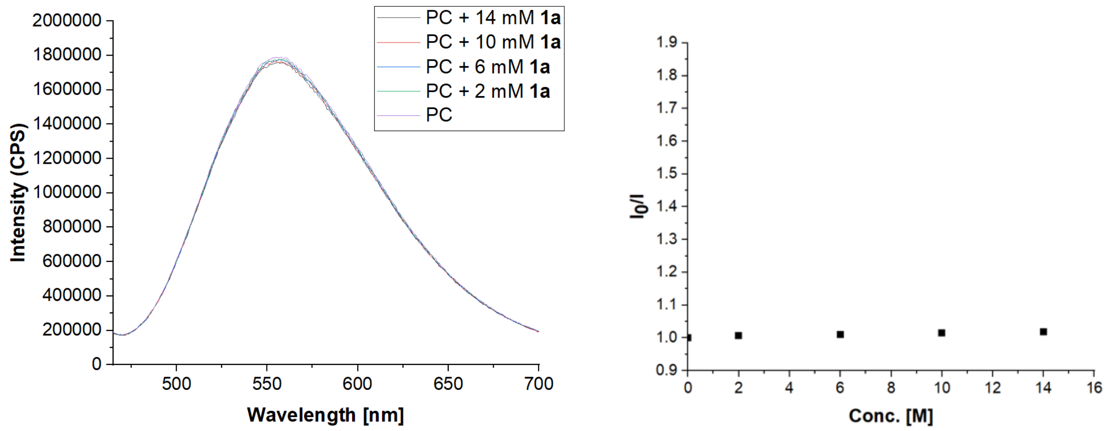


**Figure S2.** Fluorescence quenching studies of PC (5-TCzBN) with **1a**

## 6.8 Synthesis and reactivity studies of Ni-2 and Ni-3 complexes

- *Stoichiometric and catalytic studies of* ***Ni-2*** *complex with* ***2a***

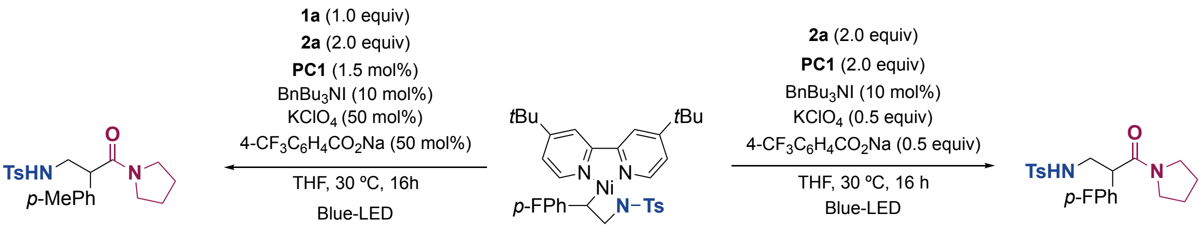


**3a**, 75% **Ni-2** **3g**, 38%

***Synthesis of Ni-2 complex:*** The **Ni-2** complex was prepared according to a slightly modified reported procedure[11] and its analytical data were consistent with the literature. In a nitrogen-filled glovebox, a 20 vial equipped with a magnetic stirring bar is charged with Ni(cod)$_2$ (150.0 mg, 0.545 mmol, 1.00 equiv), 4,4'-di-*tert*-butyl-2,2'-bipyridine (146.4 mg, 0.545 mmol 1.00 equiv), and THF (6 mL). At room temperature, the mixture was allowed to stir for 30 min until it turned into a homogeneous dark-purple

solution. To the latter a solution containing 158.8 mg of 2-(4-fluorophenyl)-1-(*p*-tolylsulfonyl)aziridine (0.545 mmol, 1.00 equiv) in THF (3 mL) was added at room temperature, followed by stirring for 18 h. The resulting mixture was concentrated under reduced pressure to afford a sticky red residue, which was extracted with 8 mL of THF, filtered through a 0.45 μm PTFE syringe filter and dried under vacuum. The resulting residue was washed with a 1:3 mixture of diethyl ether/pentane, and the so formed dark ruby colored solid dried on vacuum. The desired product was isolated as an analytically pure red solid in 59 % yield (200 mg, 0.323 mmol) without further purification protocol. It was stored in the glovebox freezer at –35 ºC for further use. Crystals suitable for X-ray diffraction were obtained by vapour diffusion of pentane in a concentrated THF solution of **Ni-2**.

**$^{19}$F{$^{1}$H} NMR** (282.4 MHz, $C_6D_6$) δ -120.1

**$^{1}$H NMR** (300 MHz, $C_6D_6$) δ 10.04 (d, $J_{HH}$ = 6.0 Hz, 1H), 8.63 (d, $J_{HH}$ = 7.9 Hz, 1H), 7.99 (m, 3H), 7.22 (s, 1H), 7.09 (s, 1H), 7.03 (d, $J_{HH}$ = 8.1 Hz, 2H), 6.87 (m, 1H), 6.77 (t, $J_{HH}$ = 8.6 Hz, 2H), 6.71 (m, 1H), 4.73 (m, 1H), 4.27 (m, 1H), 2.00 (s, 3H), 1.83 (m, 1H), 0.96 (s, 9H), 0.85 (m, 9H).

***Stoichiometric studies:*** In a glovebox, a 7 mL vial equipped with a magnetic stir bar was charged with 5-TCzBN (**PC1**, 2.0 equiv, 58.6 mg), 4-$CF_3C_6H_4CO_2Na$ (2.1 mg, 0.010 mmol, 0.50 equiv), $BnBu_3NI$ (0.8 mg, 10 mol%), $KClO_4$ (1.4 mg, 0.010 mmol, 0.50 equiv), **Ni-2** (12.4 mg, 0.020 mmol, 1.0 equiv), and **2a** (14.0 mg, 0.040 mmol, 2.0 equiv). Anhydrous THF (1.0 mL) was then added via syringe, and the vial was sealed with an aluminum crimp cap. The vial was removed from the glovebox, and the reaction mixture was stirred at 850 rpm and irradiated with 451 nm light in a blue LED photoreactor at 30 °C for 16 hours. Upon completion, the mixture was filtered through a short column of silica gel using ethyl acetate as the eluent. The reaction outcome was quantified by $^{1}$H NMR and $^{19}$F NMR analysis using 1,3,5-trimethoxybenzene (13 mg) and $C_6F_6$ (10 μL) as an internal standard, 38% yield of **3g** was observed.

***Catalytic studies:*** In a glovebox, a 7 mL vial equipped with a magnetic stir bar was charged with 5-TCzBN (**PC1**, 1.5 mol%, 2.2 mg), 4-$CF_3C_6H_4CO_2Na$ (15.9 mg, 0.05 mmol, 0.50 equiv), w/ or w/o $BnBu_3NI$ (4.2 mg, 10 mol%), $KClO_4$ (6.9 mg, 0.05 mmol, 0.50 equiv), **Ni-2** (10 mol%, 6.2 mg, 0.01 mmol), **1a** (28.7 mg, 0.10 mmol, 1.0 equiv), and **2a** (70 mg, 0.20 mmol, 2.0 equiv). Anhydrous THF (3 mL) was then added via syringe, and the vial was sealed with an aluminum crimp cap. The vial was removed from the glovebox, and the reaction mixture was stirred at 850 rpm and irradiated with 451 nm light in a blue LED photoreactor at 30 °C for 16 hours. Upon completion, the mixture was filtered through a short column of silica gel using ethyl acetate as the eluent. The reaction outcome was quantified by GC analysis using dodecane as an internal standard, 75% of **3a** was observed.

- *Photochemical Reactivity of **Ni-3** and **Ni-2**: Evidence for Light-Induced Radical Generation and Coupling*

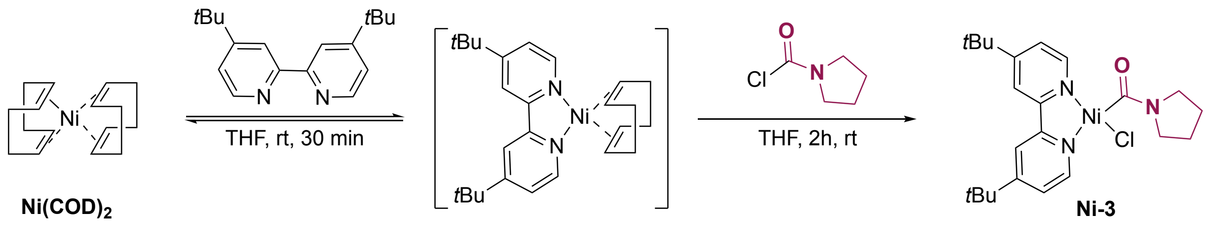


***Synthesis of complex Ni-3***: In a $N_2$-filled glovebox, both $Ni(COD)_2$ (137.5 mg, 0.50 mmol) and 4,4'-di-*tert*-butyl-2,2'-bipyridine (134.2 mg, 0.50 mmol) were dissolved in THF (2.0 mL) in an oven-dried 20 mL vial equipped with a magnetic stir bar. The reaction mixture was stirred at room temperature for 30 min, during which the solution turned deep blue. Then, a solution of pyrrolidine-1-carbonyl chloride (66.8 mg, 0.50 mmol) in THF (2.0 mL) was then added dropwise, and the resulting mixture was stirred at room temperature for an additional 2 h. The reaction mixture was filtered through a 0.45 μm PTFE syringe filter, and the solvent was removed under reduced pressure. The resulting orange solid was washed with n-pentane (2 × 5 mL), and the residual solid was dried under vacuum for 24 h. The desired complex was isolated as a pure orange amorphous solid in 78% yield (180 mg, 0.391 mmol) without further purification protocols and stored inside the glovebox at room temperature. Crystals suitable for X-ray diffraction were obtained from a concentrated THF solution at –35 °C. **$^{1}$H NMR** (400 MHz, $CD_2Cl_2$) δ 8.82 (d, $^{3}J_{HH}$ = 6.3 Hz, 1H), 8.14 (m, 1H), 7.83 (s, 2H), 7.47 (d, $^{3}J_{HH}$ = 5.8 Hz, 1H), 7.37 (m, 1H), 4.58 (m, 1H), 4.33 (m, 1H), 3.44 (m, 2H), 1.94 (m, 1H), 1.82 (m, 3H), 1.39 (m, 18H). **$^{13}$C NMR** (101 MHz, $CD_2Cl_2$) δ 184.6, 163.9, 163.0, 155.8, 152.6, 151.7, 148.9, 124.9, 123.4, 118.1, 117.1, 48.1, 46.2, 35.7, 35.7, 30.4, 30.2, 25.4, 24.9.

- *Stoichiometric photochemical studies of **Ni-3** complex with **1a***

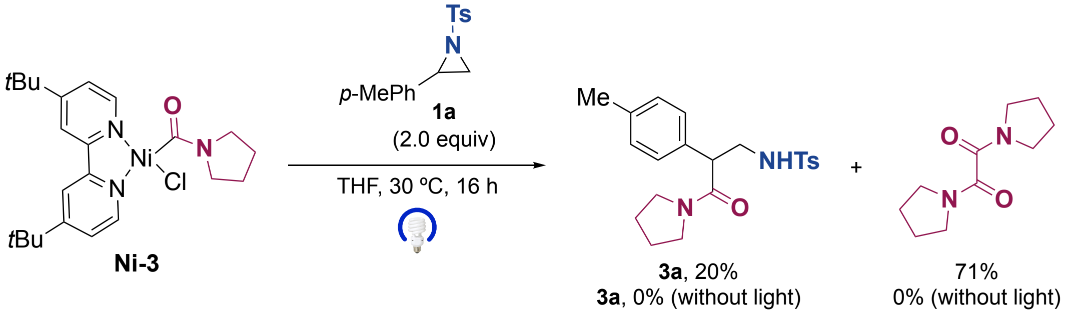


In a glovebox, a 7 mL vial equipped with a magnetic stir bar was charged with **Ni-3** (9.2 mg, 0.020 mmol, 1.0 equiv) and **1a** (11.5 mg, 0.040 mmol, 2.0 equiv). Anhydrous THF (1.0 mL) was then added via syringe, and the vial was sealed with an aluminum crimp cap. The vial was removed from the glovebox, and the reaction mixture was stirred at 850 rpm and irradiated with 451 nm light in a blue LED photoreactor at 30 °C for 16 hours. Upon completion, the reaction was quenched with 1 M HCl (1.0 mL) and extracted with ethyl acetate (3 × 2.0 mL). The mixture was quantified by GC analysis

using dodecane as an internal standard, affording the desired product **3a** in 20% yield. Additionally, quantification of the carbamoyl-carbamoyl species coming from radical recombination was performed by means of NMR using 1,3,5-trimethoxybenzene (71 % yield). Control experiment conducted in the absence of light showed that photoexcitation is required for the productive formation of product **3a**.

- *Stoichiometric photochemical studies of **Ni-3** and **Ni-2***

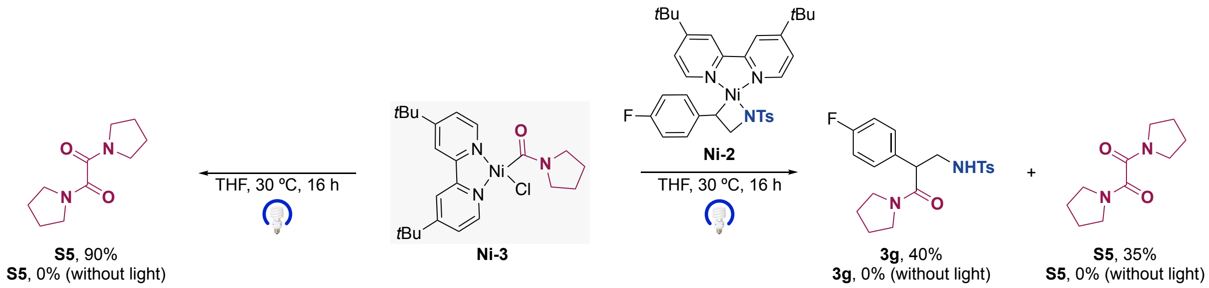


*Photochemical Cross-Coupling of **Ni-3** and **Ni-2***

To investigate the potential of **Ni-3** as a photoactive radical precursor, a stoichiometric cross-coupling reaction with **Ni-2** was carried out. In a $N_2$-filled glovebox, a 7 mL vial equipped with a magnetic stir bar was charged with the stoichiometric amount of **Ni-3** (10.0 mg, 0.022 mmol) and **Ni-2** (13.4 mg). Anhydrous THF (1.0 mL) was added, and the vial was sealed with an aluminum crimp cap. The sealed vial was removed from the glovebox and placed in a blue LED photoreactor (451 nm). The reaction mixture was irradiated at 30 ºC while stirring at 850 rpm for 16 h. Upon completion, the mixture was quenched with 1 mL of a 1 M HCl solution and extracted with ethyl acetate (3 x 2.0 mL). The combined organic layers were dried over $Na_2SO_4$, filtered, and analyzed by gas chromatography using dodecane as an internal standard. The desired product **3g** was afforded in 40% yield together with 35% of **S5** coming from radical-radical recombination. The same procedure, performed in the absence of light, resulted in no detectable formation of **3g**, confirming that light irradiation is essential for productive coupling.

*Radical Generation from **Ni-3**: Homocoupling Experiment*

To assess the ability of **Ni-3** to release radicals independently, a reaction was performed in the absence of **Ni-2**. In a $N_2$-filled glovebox, **Ni-3** (10.0 mg, 0.022 mmol) was dissolved in anhydrous THF (1.0 mL) in a 7 mL vial equipped with a magnetic stir bar and sealed with a crimp cap. The vial was removed from the glovebox and irradiated under 451 nm blue LED light at 30 °C for 16 h. Subsequently, the reaction mixture was exposed to air, followed by the addition of ethyl acetate (5.0 mL), and stirred for 1 h to ensure complete quenching. 1,3,5-Trimethoxybenzene was added as an internal standard, followed by filtration through a 0.45 µm PTFE syringe filter, concentrated under reduced pressure, and analyzed by $^1$H NMR spectroscopy. The homocoupled product **S5** was obtained in 50% yield (determined by NMR integration vs. internal standard). The same procedure performed in the absence

of light resulted in no detectable formation of **S5**, confirming that light irradiation is essential for productive coupling.

- *Stoichiometric photochemical studies of **Ni-3** with ethyl 2-phenylacrylate as radical acceptor*

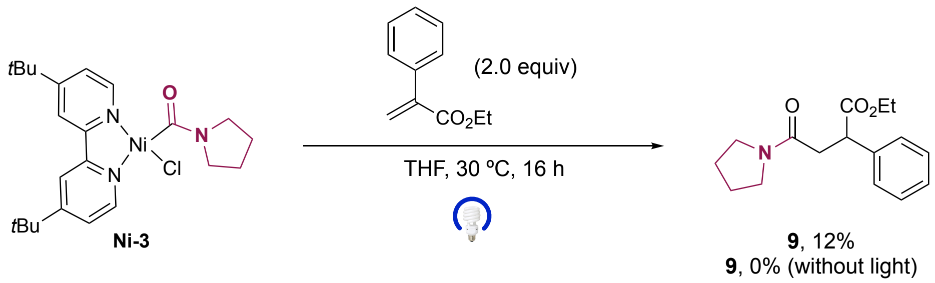


In a glovebox, a 7 mL vial equipped with a magnetic stir bar was charged with **Ni-3** (9.2 mg, 0.020 mmol, 1.0 equiv) and ethyl 2-phenylacrylate (7.1 mg, 0.040 mmol, 2.0 equiv). Anhydrous THF (1.0 mL) was then added via syringe, and the vial was sealed with an aluminum crimp cap. The vial was removed from the glovebox, and the reaction mixture was stirred at 850 rpm and irradiated with 451 nm light in a blue LED photoreactor at 30 °C for 16 hours. Upon completion, the reaction was quenched with 1 M HCl (1.0 mL) and extracted with ethyl acetate (3 × 2.0 mL). The reaction outcome was quantified by GC analysis using dodecane as an internal standard, affording the desired product **9** in 12% yield. In a control experiment performed in the absence of light, no formation of product **9** was detected.

### 6.9 Stability tests of Ni-2 complex

To assess whether the limited reactivity of **Ni-2** under stoichiometric conditions arises from instability toward components of the catalytic system, a series of control experiments were conducted. Independent mixtures of **Ni-2** (10 mg, 0.016 mmol, 1.0 equiv) with $KClO_4$, $BnBu_3NI$, 4-$CF_3C_6H_4CO_2Na$, or **PC1** (2.0 equiv each) were prepared in THF (2 mL) and stirred at room temperature for 3 days. In all cases except for the reaction with, the $^{19}F$ NMR spectra of the crude mixtures showed no evidence of decomposition (Figure S3), confirming that **Ni-2** remains intact in the presence of each additive. When the mixture containing PC1 was subsequently irradiated with blue LEDs at 30 °C, minor decomposition was detected after 24 h, with part of the initial Ni(II) complex remaining. These observations indicate that **Ni-2** is thermally and chemically robust under the reaction conditions and that its apparent stoichiometric inactivity is not due to decomposition by any component of the catalytic mixture. In contrast, under catalytic conditions **Ni-2** complex delivers the desired coupling product **3a** in 75% yield.

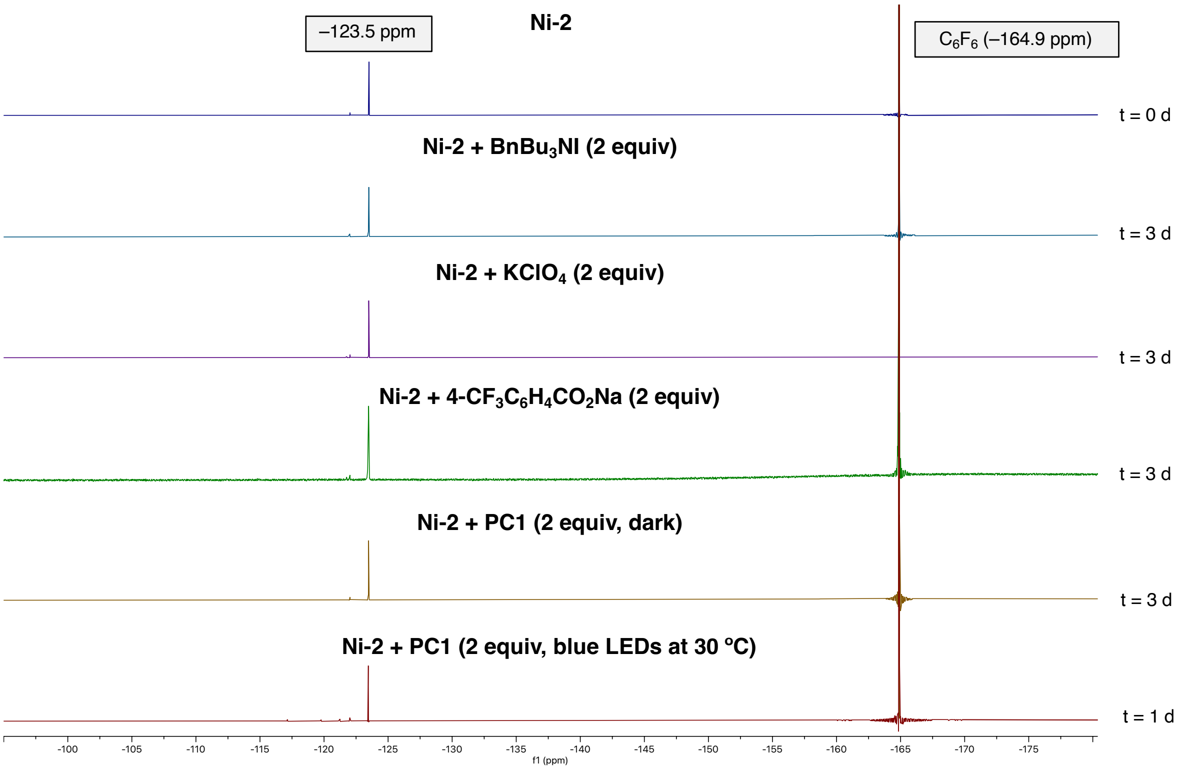


**Figure S3.** Stability assessment of **Ni-2** toward the components of the catalytic reaction, monitored by $^{19}F\{^1H\}$ NMR after 3 days of reaction. $C_6D_6$ solutions of hexafluorobenzene sealed in glass capillaries were used as external standards.

### 6.10 Discussion on the reactivity of Ni-2 under both stoichiometric and catalytic conditions

As previously discussed, stoichiometric experiments with the azanickelacyclic complex **Ni-2** and the same reaction but under catalytic conditions show different outcomes, with higher efficiency for the reaction under catalytic conditions (75% yield of **3a**). These findings indicate that the lack of reactivity does not arise from Ni(II) instability or poor radical-trapping capability. Indeed, the successful interception of carbamoyl radicals by **Ni-2** in the reaction between **Ni-2** and **Ni-3**, yielding **3g** in 40% yield, confirms that **Ni-2** is fully competent to capture carbon-centered radicals and undergo C–C bond-forming reductive elimination. Instead, the discrepancy between the stoichiometric and catalytic outcomes is most consistently explained by differences in the kinetics of radical generation. Under photocatalytic conditions, carbamoyl radicals are produced at a controlled, steady-state concentration via the photochemical homolysis of **Ni-3**, which acts as a radical reservoir. In contrast, when **Ni-2** is combined directly with Hantzsch ester (**2a**) and **PC1**, radical formation occurs too rapidly, leading to a transiently high concentration of $R^{\bullet}$. The consequent radical–radical recombination events outcompete radical capture by **Ni-2**, resulting in decomposition and minimal productive coupling. This kinetic interpretation is consistent with the observation that **Ni-2** is catalytically competent—where the radical formation is self-regulated by the photoredox cycle—but stoichiometrically inefficient when exposed to a high concentration of carbamoyl radicals. Thus, the efficiency of the system is not limited by **Ni-2**

reactivity, but by the radical generation rate and capture kinetics balance, which determines whether productive turnover or unproductive recombination predominates.

## 7. Photophysical Characterization of Ni-3: UV–Vis Spectroscopy and Quantum Yield Analysis

### 7.1 UV–Vis Spectroscopic Analysis

To evaluate the photochemical properties of **Ni-3**, UV–vis absorption spectra were recorded across a range of concentrations in anhydrous THF. As shown in Figure S4, **Ni-3** displays two major absorption bands in the UV (296 nm) and visible (488 nm) regions, with increasing absorbance intensity corresponding to increasing concentration. The linear dependence of absorbance on concentration was confirmed by Beer–Lambert plots at selected wavelengths. At 296 nm, the absorbance of **Ni-3** shows confirms linearity ($R^2$ = 0.9713) and gives ε ≈ 28394 $M^{-1}$ $cm^{-1}$ (Figure S4). At 488 nm a second calibration shows a strong linear correlation with the concentration ($R^2$ = 0.9998, Figure S4), yielding a molar extinction coefficient (ε) of 4071 $M^{-1}$ $cm^{-1}$. At 550 nm, a second calibration confirms linearity ($R^2$ = 0.9713) and gives ε ≈ 28,394 $M^{-1}$ $cm^{-1}$ (Figure S4). These values confirm that **Ni-3** effectively absorbs light at the 451 nm excitation wavelength used for reactivity and quantum yield studies.

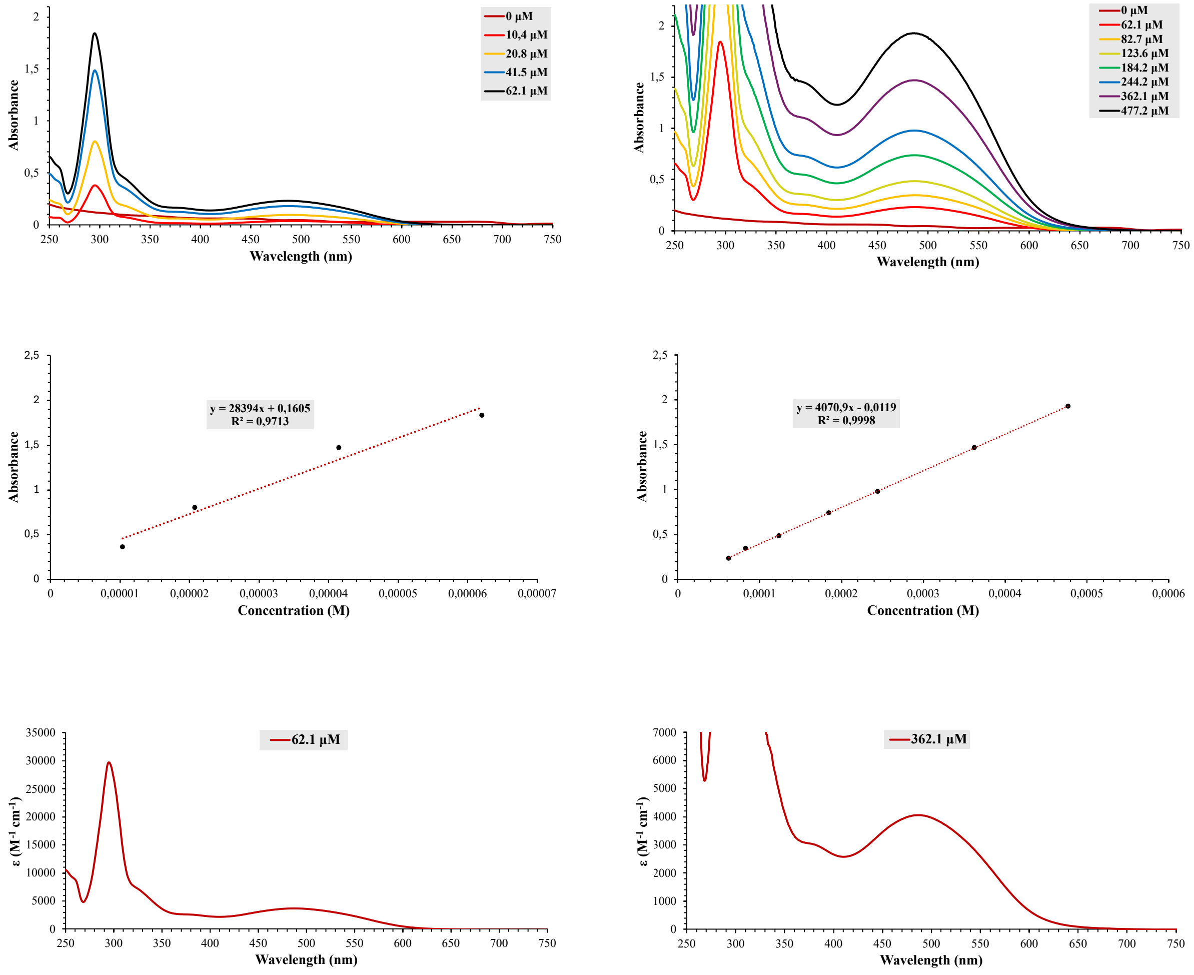

**Figure S4.** UV–vis plots in THF of **Ni-3** at different concentrations (top), Beer–Lambert plots used to determine molar extinction coefficients at 296 nm and 488 nm (middle), and the calculated molar absorption coefficients (ε) at 62.1 μM and 362.1 μM (bottom). The left panels are for absorption band at 296 nm, while the right panels are for absorption band at 488 nm.

### 7.2 Photostability and Kinetic Analysis of Ni-3 under 451 nm Irradiation

The photodegradation of **Ni-3** was investigated under the same photochemical conditions used for catalytic reactions, employing a photoreactor equipped with 451 nm OSRAM Oslon SSL 80 royal-blue LEDs (LT-1960). The photon flux and output power of the LED setup was independently characterized and used to standardize rate and quantum yield calculations. In a $N_2$-filled glovebox, 2.5 mL of a 0.36 mM solution of **Ni-3** in anhydrous THF was transferred to a screw-cap quartz cuvette and irradiated under constant stirring at 30 ºC. The absorbance at 451 nm was monitored at regular time intervals to track the degradation process. The decay profile was modelled using the exponential form of the first-order rate law (Equation S1):

$$A_x = [(A_i - A_f) \cdot e^{-k \cdot x}] + A_f$$

where $A_x$ is the absorbance at time $x$, $A_i$ and $A_f$ are the initial and final absorbances, respectively, and $k$ is the first-order rate constant. Nonlinear regression yielded a rate constant of $k$ = 0.027 $min^{-1}$, corresponding to a half-life $t_{1/2}$ = 25.7 min, with a fit quality of $R^2$ = 0.845. Decomposition proceeded cleanly over one full half-life at 30 ºC.

Attempts to linearize the data using integrated forms of zero-, first-, and second-order kinetics did not produce satisfactory linear plots, indicating that the decomposition pathway is more complex than simple unimolecular process. Possible explanations include the involvement of multiple concurrent photochemical processes or competing side pathways such as radical recombination or ligand loss. While the exponential model provides a reasonable estimate of the apparent rate constant and half-life, the kinetic behavior likely reflects a more complex mechanism.

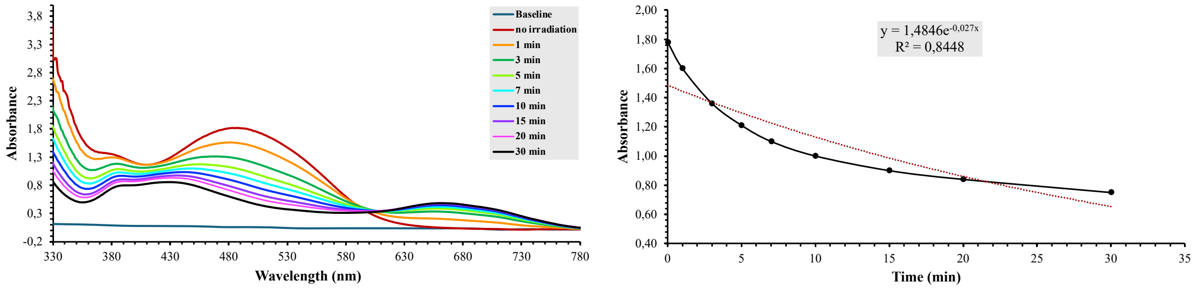


**Figure S5**. UV-Vis spectrum for the photodegradation of compound **Ni-3** and the fit to first order kinetics.

### 7.3 Quantum yield

Quantum yield (Φ) was calculated using the rate of photodegradation of **Ni-3** and the total photon flux delivered by the light source, following Equation S2. The calculation incorporates Planck's constant ($h$), the speed of light ($c$), Avogadro's constant ($N_A$), the volume of the irradiated solution ($V_{cuvette}$), the rate of degradation of the complex ($k_{complex}$), the initial concentration of **Ni-3** ($[Ni^{II}]_0$), the power of the LED ($P_{LED}$), the wavelength of the LED ($\lambda_{LED}$), and the absorbance of **Ni-3** at the irradiation wavelength ($A_{\lambda LED}$).

$$\Phi = \frac{h * c * N_A * V_{cuvette} * k_{degradation} * [Ni^{II}]_0}{P_{LED} * \lambda_{LED} * (1 - 10^{-\left(A_{\lambda LED}{}^{(Ni^{II})}\right)})}$$

For this study, we used quartz cuvettes with 1 cm path lengths and filled them with 2.5 mL solution, which results in having a side rectangular surface area of about 250 $mm^2$. The surface exposed to LED irradiation was that of the bottom of the cuvette (100 $mm^2$). As such, the measured $P_{LED}$ values reported in $mW/cm^2$ are a close approximation of the power experienced by the cuvette and by extension to that of the vials in the catalytic reactions.

**Table S2.** Values for calculating quantum yield using Equation S2 for compound **Ni-3**.

| Compound | $\lambda_{LED}$ ($10^{-9}$ m) | $k_{degradation}$ ($10^{-3}$ $min^{-1}$) | $A_{\lambda LED}{}^{Ni(II)}$ | $[Ni^{II}]_0$ (M) | $P_{LED}$ (W) | Φ (10-4) |
|---|---|---|---|---|---|---|
| **Ni-III** | 451 | 27 | 1.31 | 3.6E-4 | 3.8E-1 | 3.07 |

This low value suggests that only ~0.03% of absorbed photons lead to productive decomposition of **Ni-3** under these conditions, consistent with a slow or inefficient primary photochemical step.

### 7.4 EPR characterization of photogenerated Ni(I) species from Ni-3

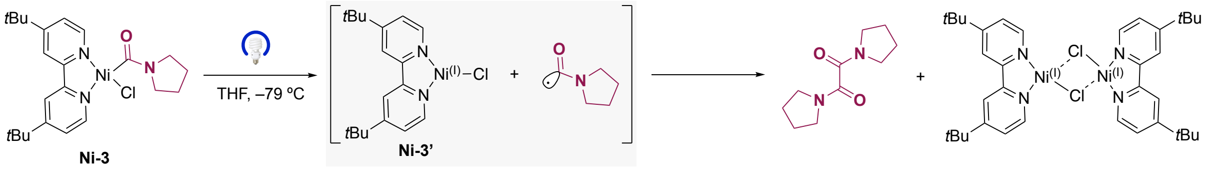


To investigate the **Ni-3'** photogeneration from **Ni-3**, time-resolved X-band CW-EPR spectroscopy was employed using a Bruker EMXmicro spectrometer (9.387 GHz, modulation amplitude: 10 G, microwave power: 0.5375 mW, time constant: 20.48 ms, 148 scans per spectrum). Thus, 0.5 mL of a 0.03 M THF solution of the **Ni-3** was loaded into a J. Young EPR tube, cooled to –79 °C in a dry ice/acetone bath, and subjected to continuous irradiation with a 456 nm Kessil lamp (Figure S5). At defined time intervals (every 10 minutes), photoirradiation was temporarily interrupted to rapidly freeze the sample in liquid nitrogen, enabling EPR analysis at 77 K. This time-resolved approach enabled direct observation of changes in the electronic structure as a function of irradiation time. Thus, after 20 minutes of photoirradiation at 456 nm, the initial **Ni-3** complex was cleanly converted to an EPR-active Ni(I) species (Figure S6), showing a rhombic signal characterized by $g_x$=2.076, $g_y$=2.075, and

$g_z$=2.295, that can be assigned to **Ni-3'**, consistent with previously reported studies by Mirica *et al.*.[12] These data support a mechanistic scenario in which, under 451 nm irradiation, the carbamoyl ligand in the **Ni-3** complex undergoes homolytic cleavage to generate a **Ni-3'** species and a free carbamoyl radical. Given that the catalytic carbamoylation of aziridines proceeds under identical photochemical conditions, it is unlikely that the carbamoyl radical ever binds to nickel in a low valent Ni(I) species. Instead, radical capture likely occurs at Ni(II) intermediate, forming a high-valent Ni(III) species that undergoes spontaneous reductive elimination to forge C–C bond coupling.

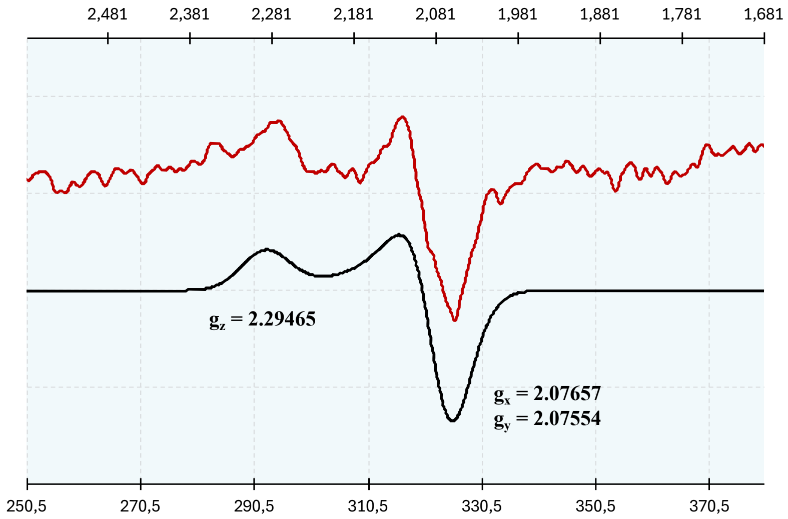


**Figure S6.** X-band EPR spectrum of photogenerated **Ni-3′** obtained after 20 min irradiation of **Ni-3** (0.03 M in THF) at 456 nm and –78 °C. The sample was frozen at 77 K in liquid nitrogen prior measurement.

## 8. Alternative Mechanistic Scenario: Proposed Ni(I)/Ni(III)/Ni(II)/Ni(III) Manifold (*path B*)

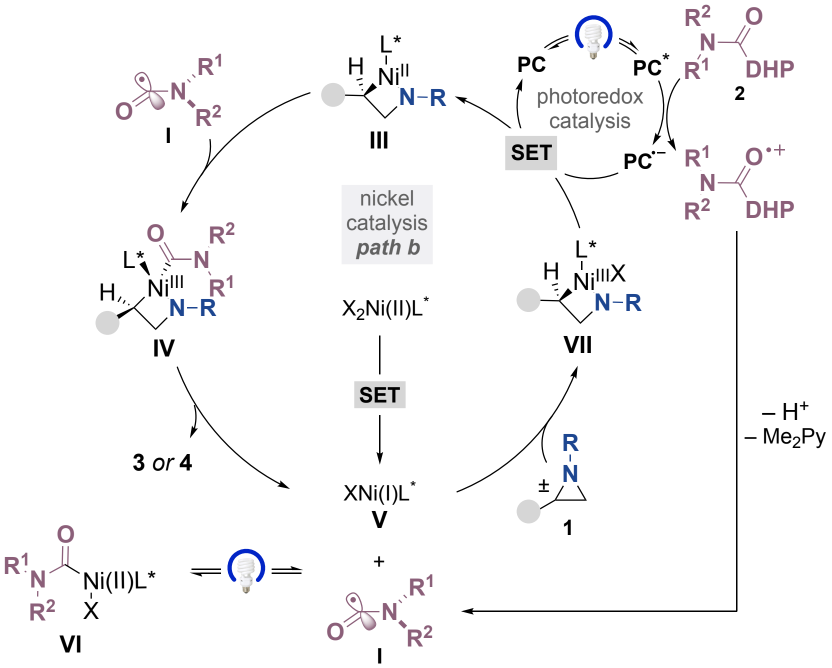


**Figure S7.** Alternative mechanistic rationale

In addition to the Ni(0)/Ni(II)/Ni(III) sequence discussed in the main text, an alternative mechanistic scenario was also considered (see Figure S7). In this pathway, Ni(I)L* species **V** generated through single-electron transfer (SET) reduction of Ni(II)$L_2$ by the photoredox catalyst engage directly in oxidative addition of aziridine **1**, generating a Ni(III) intermediate **VII**. Subsequent SET reduction generates the Ni(II) azanickelacyclic intermediate **III**, which is intercepted by the carbamoyl radical en route to the high-valent Ni(III) intermediate **IV**. Eventually, the Ni(I) species **V** is recovered upon reductive elimination, completing the cycle and productively releasing the desired products **3** or **4**.This pathway differs from Ni(0)/Ni(II)/Ni(III) sequence primarily in that aziridine activation occurs at Ni(I) rather than Ni(0). Although such a mechanism is conceptually feasible, several observations argue against it as the dominant process under the reaction conditions. Indeed, electrochemical data show that the reduction potential of the aziridine ($E_{1/2}$ = –2.65 V vs $Fc^+$/Fc) is substantially more negative than that of either the photocatalyst ($E_{1/2}$[PC/$PC^{\bullet-}$] = –1.99 V vs $Fc^+$/Fc) or the Ni(I)/Ni(0) couple for **Ni-1** (–1.89 V), making SET activation of the aziridine thermodynamically inaccessible towards potential oxidative addition reaction to **V** or radical-radical coupling with carbamoyl. Additionally, cyclic voltammetry of the isolated Ni(II) complex **Ni-1** indicates that photoinduced reduction to Ni(0) is thermodynamically favourable further supporting a Ni(0)-initiated pathway. Taken together, these results suggest that the alternate mechanism delineated in Figure S7 is less consistent with our available data but by no means should be entirely excluded.

## 9. X-Ray Crystallographic Data

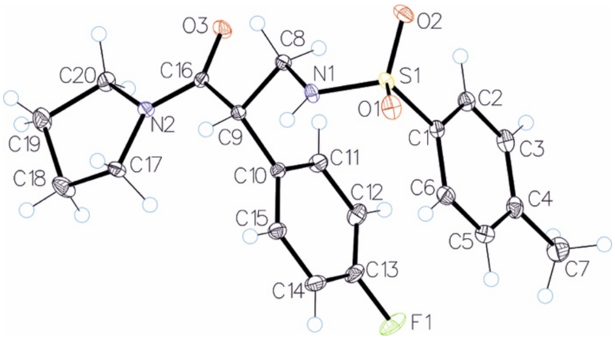


**Table S3 Crystal data and structure refinement for 3g (CCDC-2485306).**

| | |
|---|---|
| Empirical formula | $C_{20}H_{23}FN_2O_3S$ |
| Formula weight | 390.46 |
| Temperature/K | 99.90 |
| Crystal system | orthorhombic |
| Space group | $P2_12_12_1$ |
| a/Å | 5.8938(4) |
| b/Å | 13.4472(8) |
| c/Å | 23.5690(15) |
| α/° | 90 |
| β/° | 90 |
| γ/° | 90 |
| Volume/Å$^3$ | 1868.0(2) |
| Z | 4 |
| $\rho_{calc}$g/cm$^3$ | 1.388 |
| μ/mm$^{-1}$ | 0.207 |
| F(000) | 824.0 |
| Crystal size/mm$^3$ | 0.3 × 0.05 × 0.05 |
| Radiation | MoKα (λ = 0.71073) |
| 2Θ range for data collection/° | 3.456 to 63.318 |
| Index ranges | $-8 \le h \le 7$, $-19 \le k \le 14$, $-34 \le l \le 34$ |
| Reflections collected | 36899 |
| Independent reflections | 6271 [$R_{int}$ = 0.0473, $R_{sigma}$ = 0.0266] |
| Data/restraints/parameters | 6271/0/245 |
| Goodness-of-fit on $F^2$ | 1.099 |
| Final R indexes [I>=2σ (I)] | $R_1$ = 0.0281, $wR_2$ = 0.0752 |
| Final R indexes [all data] | $R_1$ = 0.0293, $wR_2$ = 0.0761 |
| Largest diff. peak/hole / e Å$^{-3}$ | 0.34/-0.34 |
| Flack parameter | 0.010(15) |

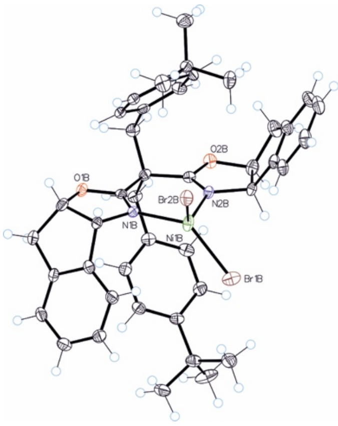


**Table S4 Crystal data and structure refinement for Ni-1 (CCDC-2485307).**

| | |
|---|---|
| Empirical formula | $C_{43}H_{46}Br_2NiO_2N_2$ |
| Formula weight | 841.35 |
| Temperature/K | 99.91 |
| Crystal system | monoclinic |
| Space group | $P2_1$ |
| a/Å | 14.4080(15) |
| b/Å | 14.7992(13) |
| c/Å | 18.9070(15) |
| α/° | 90 |
| β/° | 108.374(3) |
| γ/° | 90 |
| Volume/Å$^3$ | 3826.0(6) |
| Z | 4 |
| $\rho_{calc}$g/cm$^3$ | 1.461 |
| μ/mm$^{-1}$ | 2.634 |
| F(000) | 1728.0 |
| Crystal size/mm$^3$ | 0.4 × 0.3 × 0.1 |
| Radiation | MoKα (λ = 0.71073) |
| 2Θ range for data collection/° | 2.27 to 63.452 |
| Index ranges | $-21 \leq h \leq 21$, $-21 \leq k \leq 15$, $-24 \leq l \leq 27$ |
| Reflections collected | 73984 |
| Independent reflections | 21305 [$R_{int}$ = 0.0519, $R_{sigma}$ = 0.0588] |
| Data/restraints/parameters | 21305/97/944 |
| Goodness-of-fit on F$^2$ | 1.027 |
| Final R indexes [I>=2σ (I)] | $R_1$ = 0.0399, w$R_2$ = 0.0743 |
| Final R indexes [all data] | $R_1$ = 0.0627, w$R_2$ = 0.0816 |
| Largest diff. peak/hole / e Å$^{-3}$ | 0.98/-1.23 |
| Flack parameter | -0.014(4) |

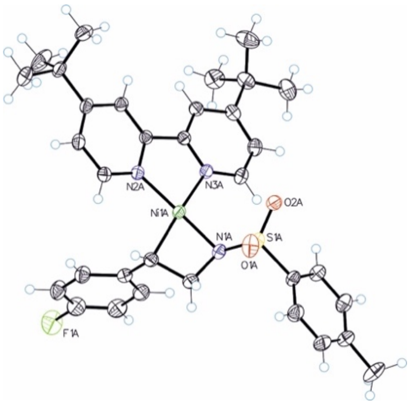


**Table S5 Crystal data and structure refinement for Ni-2 (CCDC-2486937).**

| | |
|---|---|
| Empirical formula[a] | $C_{43.80}H_{59.60}FN_3NiO_{4.70}S$ |
| Formula weight | 813.11 |
| Temperature/K | 100(2) |
| Crystal system | triclinic |
| Space group | P-1 |
| a/Å | 15.5431(3) |
| b/Å | 15.7915(3) |
| c/Å | 19.2152(3) |
| α/° | 88.2846(14) |
| β/° | 71.4216(16) |
| γ/° | 73.5265(18) |
| Volume/Å$^3$ | 4277.56(15) |
| Z | 4 |
| $\rho_{calc}$g/cm$^3$ | 1.263 |
| μ/mm$^{-1}$ | 0.552 |
| F(000) | 1736 |
| Crystal size/mm$^3$ | 0.4 × 0.2 × 0.2 |
| Radiation | MoKα (λ = 0.71073) |
| 2Θ range for data collection/° | 4.19 to 66.618 |
| Index ranges | $-23 \le h \le 23$, $-24 \le k \le 24$, $-29 \le l \le 29$ |
| Reflections collected | 48804 |
| Independent reflections | 48804 [$R_{sigma}$ = 0.0275] |
| Data/restraints/parameters | 48804/201/1054 |
| Goodness-of-fit on F$^2$ | 1.033 |
| Final R indexes [I>=2σ (I)] | $R_1$ = 0.0566, $wR_2$ = 0.1622 |
| Final R indexes [all data] | $R_1$ = 0.0742, $wR_2$ = 0.1731 |
| Largest diff. peak/hole / e Å$^{-3}$ | 1.098/-0.775 |

[a] The empirical formula contains decimal values due to fractional occupancies of disordered THF in the crystal. These values correspond to the refine crystallographic composition derived from the CIF data.

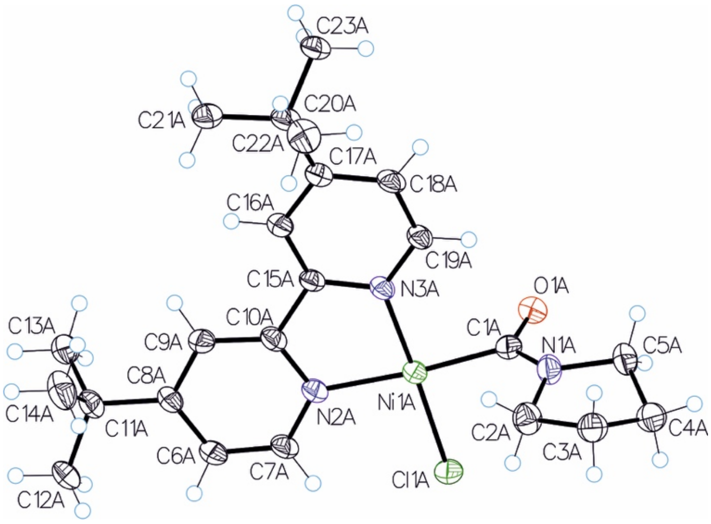


**Table S6 Crystal data and structure refinement for Ni-3 (CCDC-2485305).**

| | |
|---|---|
| Empirical formula | $C_{27}H_{40}ClN_3NiO_2$ |
| Formula weight | 532.78 |
| Temperature/K | 100(2) |
| Crystal system | Triclinic |
| Space group | P -1 |
| a/Å | 12.6581(18) |
| b/Å | 14.758(2) |
| c/Å | 15.192(2) |
| α/° | 77.251(3) |
| β/° | 85.233(3) |
| γ/° | 83.169(3) |
| Volume/Å$^3$ | 2743.6(7) |
| Z | 4 |
| $\rho_{calc}$g/cm$^3$ | 1.290 |
| μ/mm$^{-1}$ | 0.832 |
| F(000) | 1136 |
| Crystal size/mm$^3$ | 0.15 × 0.10 × 0.10 |
| Radiation | MoKα (λ = 0.71073) |
| 2Θ range for data collection/° | 2.75 to 58.60 |
| Index ranges | $-17 \leq h \leq 17$, $-19 \leq k \leq 20$, $0 \leq l \leq 20$ |
| Reflections collected | 59527 |
| Independent reflections | 14509 [$R_{sigma}$ = 0.0758] |
| Data/restraints/parameters | 14509/278/758 |
| Goodness-of-fit on F$^2$ | 1.045 |
| Final R indexes [I>=2σ (I)] | $R_1$ = 0.0697, w$R_2$ = 0.1841 |
| Final R indexes [all data] | R1 = 0.0898, w$R_2$ = 0.2033 |
| Largest diff. peak/hole / e Å$^{-3}$ | +1.419/-0.574 |

## 10. References


[1] Craig, R. A.; O'Connor, N. R.; Goldberg, A. F. G.; Stoltz, B. M. Stereoselective Lewis Acid Mediated (3+2) Cycloadditions of N -H- and N -Sulfonylaziridines with Heterocumulenes. *Chem. Eur. J.* **2014**, *20*, 4806–4813.

[2] Woods, B. P.; Orlandi, M.; Huang, C.-Y.; Sigman, M. S.; Doyle, A. G. Nickel-Catalyzed Enantioselective Reductive Cross-Coupling of Styrenyl Aziridines. *J. Am. Chem. Soc.* **2017**, *139*, 5688–5691.

[3] Huang, C.-Y. (Dennis); Doyle, A. G. Nickel-Catalyzed Negishi Alkylations of Styrenyl Aziridines. *J. Am. Chem. Soc.* **2012**, *134*, 9541–9544.

[4] Hu, X.; Cheng-Sánchez, I.; Cuesta-Galisteo, S.; Nevado, C. Nickel-Catalyzed Enantioselective Electrochemical Reductive Cross-Coupling of Aryl Aziridines with Alkenyl Bromides. *J. Am. Chem. Soc.* **2023**, *145*, 6270–6279.

[5] Lan, Y.; Han, Q.; Liao, P.; Chen, R.; Fan, F.; Zhao, X.; Liu, W. Nickel-Catalyzed Enantioselective C($sp^3$)–C($sp^3$) Cross-Electrophile Coupling of *N*-Sulfonyl Styrenyl Aziridines with Alkyl Bromides. *J. Am. Chem. Soc.* **2024**, *146*, 25426–25432.

[6] Alandini, N.; Buzzetti, L.; Favi, G.; Schulte, T.; Candish, L.; Collins, K. D.; Melchiorre, P. Amide Synthesis by Nickel/Photoredox-Catalyzed Direct Carbamoylation of (Hetero)Aryl Bromides. *Angew. Chem. Int. Ed.* **2020**, *59*, 5248–5253.

[7] Yu, Z.; Ma, S.-W.; Li, G.; Ma, L. Photocatalytic Carbamoyl Radical Transfer to Alkenyl Azaarenes. *Synlett* **2024**, *35*, 1883–1888.

[8] Pingaew, R.; Prachayasittikul, S.; Ruchirawat, S.; Prachayasittikul, V. Tungstophosphoric Acid Catalyzed Synthesis of N-Sulfonyl-1,2,3,4-Tetrahydroisoquinoline Analogs. *Chin. Chem. Lett.* **2013**, *24*, 941–944.

[9] Laha, J. K.; Sharma, S.; Dayal, N. Palladium-Catalyzed Regio- and Chemo-selective Reactions of 2-Bromobenzyl Bromides: Expanding the Scope for the Synthesis of Biaryls Fused to a Seven-Membered Sultam. *Eur. J. Org. Chem.* **2015**, *2015*, 7885–7891.

[10] (a) Luo, J.; Zhang, J. Donor–Acceptor Fluorophores for Visible-Light-Promoted Organic Synthesis: Photoredox/Ni Dual Catalytic C($Sp^3$ )–C($Sp^2$) Cross-Coupling. *ACS Catal.* **2016**, *6*, 873–877. (b) Speckmeier, E.; Fischer, T. G.; Zeitler, K. A Toolbox Approach To Construct Broadly Applicable Metal-Free Catalysts for Photoredox Chemistry: Deliberate Tuning of Redox Potentials and Importance of Halogens in Donor–Acceptor Cyanoarenes. *J. Am. Chem. Soc.* **2018**, *140*, 15353–15365.

[11] Dongbang, S.; Doyle, A. G. Ni/Photoredox-Catalyzed C($Sp^3$)–C($Sp^3$) Coupling between Aziridines and Acetals as Alcohol-Derived Alkyl Radical Precursors. *J. Am. Chem. Soc.* **2022**, *144*, 20067–20077.

[12] Westawker, L. P.; Bouley, B. S.; Vura-Weis, J.; Mirica, L. M. Photochemistry of Ni(II) Tolyl Chlorides Supported by Bidentate Ligand Frameworks. *J. Am. Chem. Soc.* **2025**, *147*, 17315–17329.

# 11. NMR Spectra

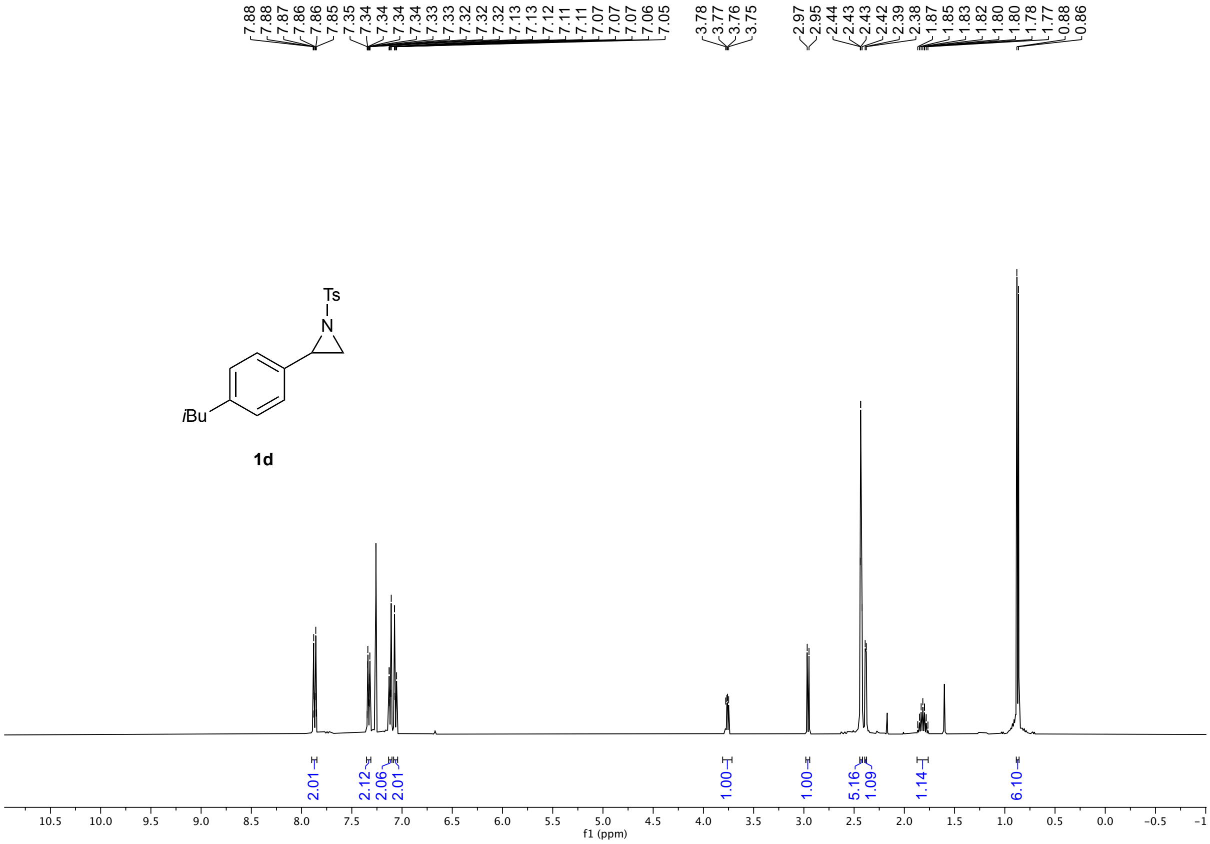


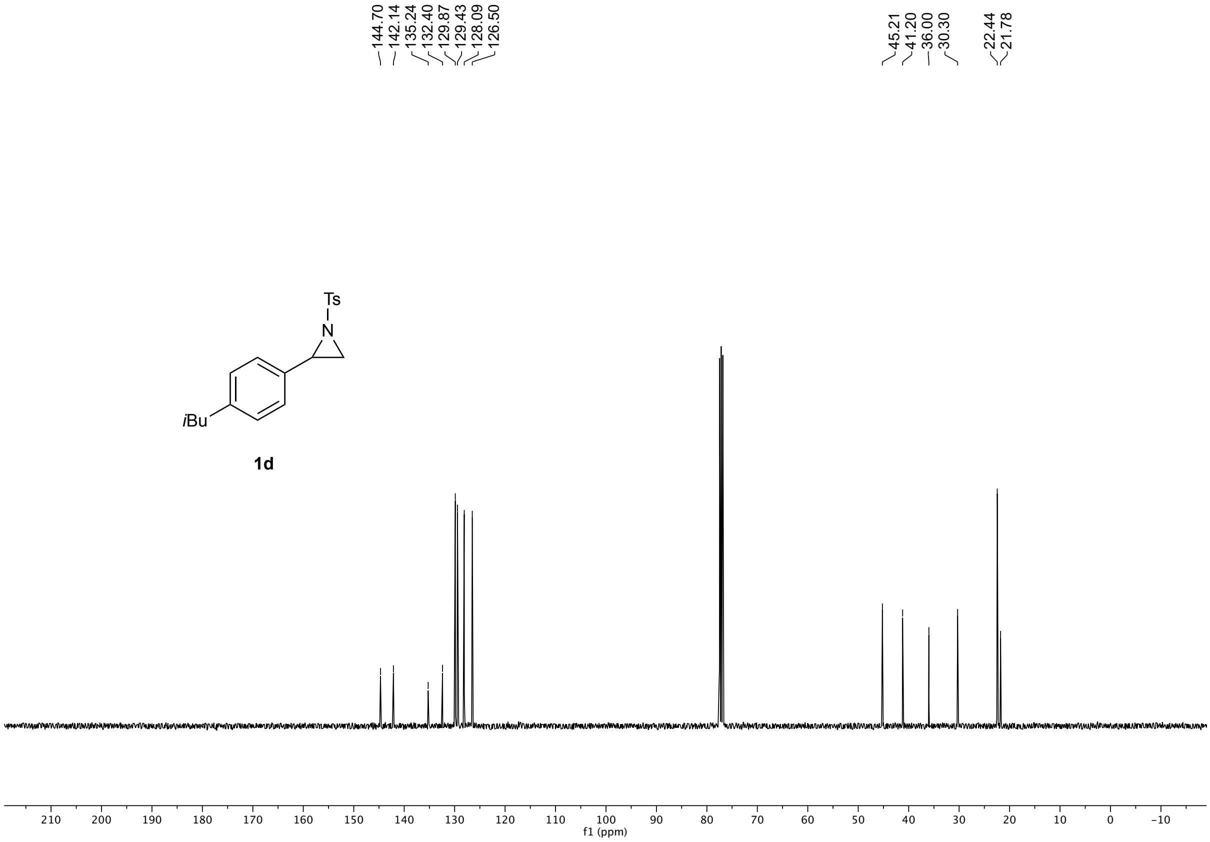

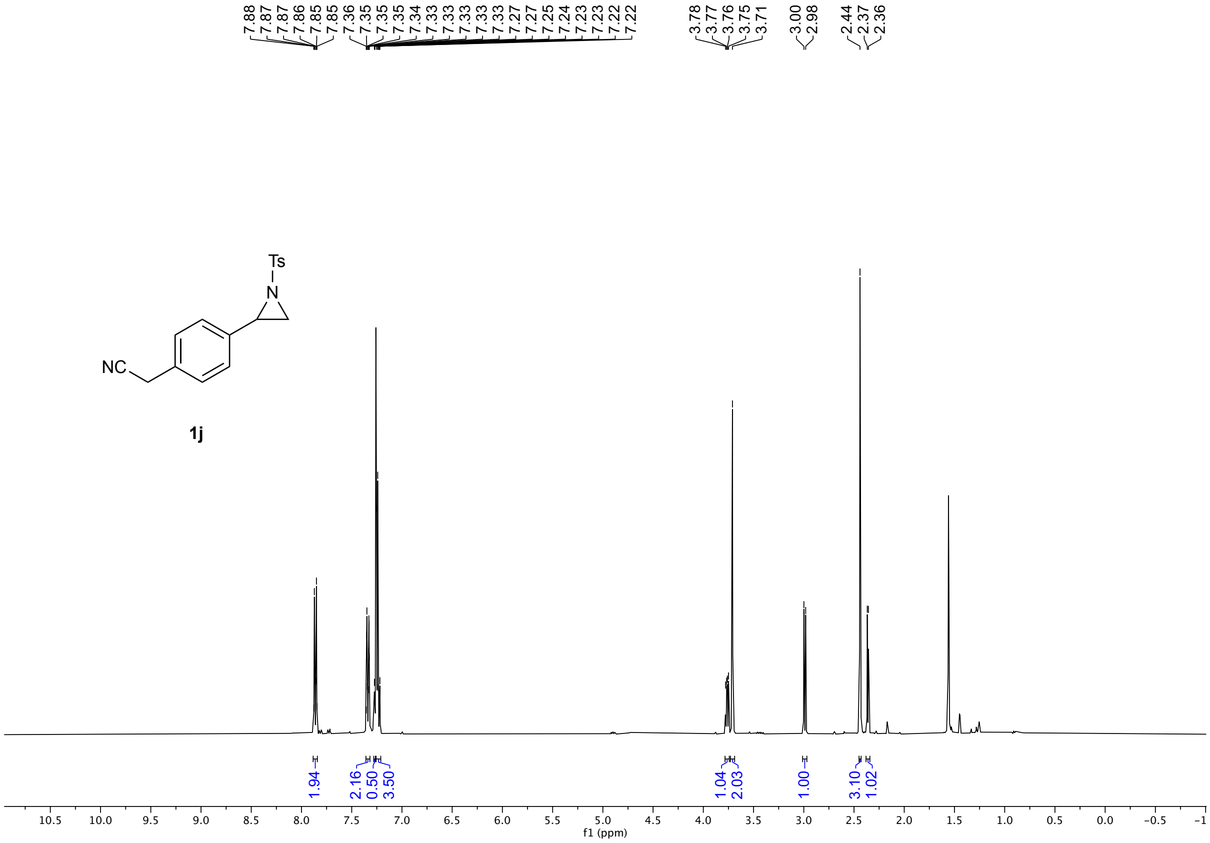
7.88
7.87
7.87
7.86
7.85
7.85
7.36
7.35
7.35
7.35
7.34
7.33
7.33
7.33
7.33
7.33
7.33
7.27
7.27
7.25
7.24
7.23
7.23
7.22
7.22
7.22
3.78
3.77
3.76
3.75
3.71
3.00
2.98
2.44
2.37
2.36
Ts
N
NC
1j
1.94
2.16
0.50
3.50
1.04
2.03
1.00
3.10
1.02
f1 (ppm)

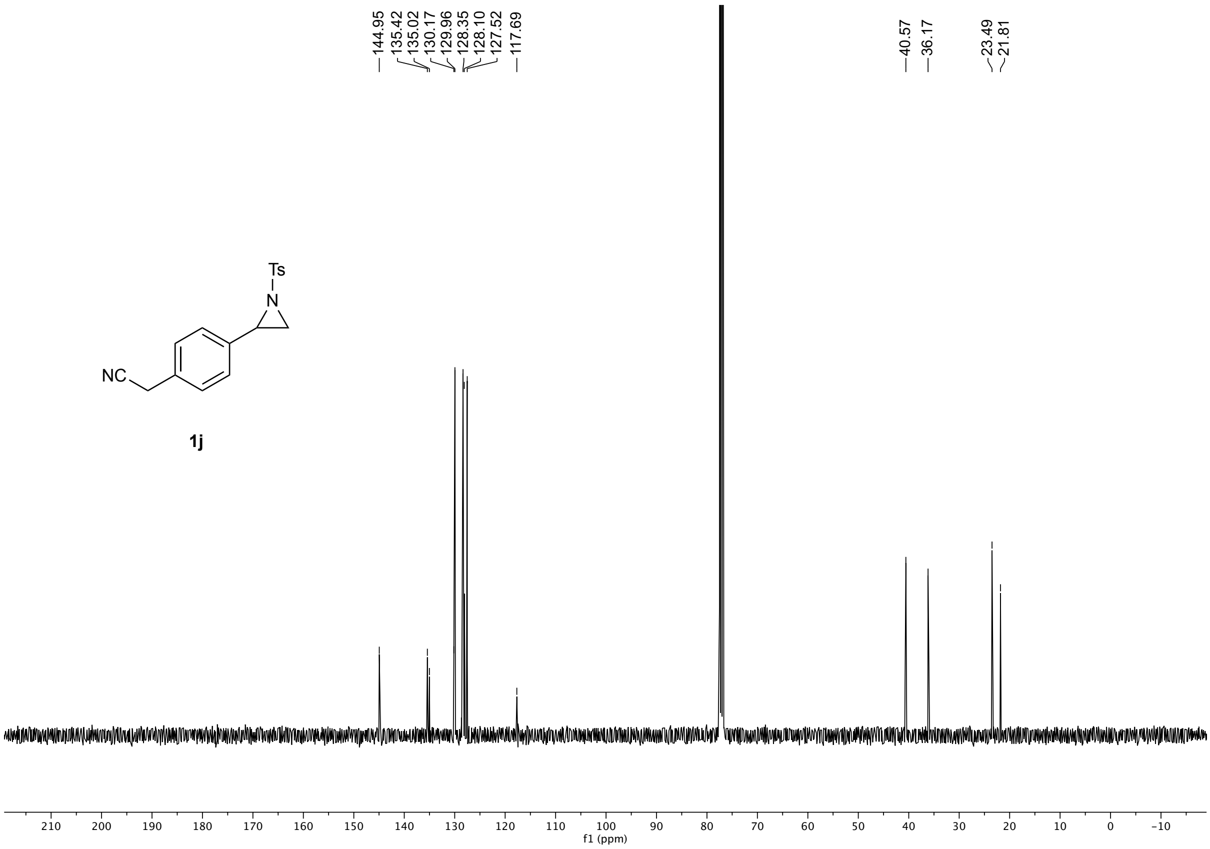
144.95
135.42
135.02
130.17
129.96
128.35
128.10
127.52
117.69
40.57
36.17
23.49
21.81
Ts
N
NC
1j
f1 (ppm)

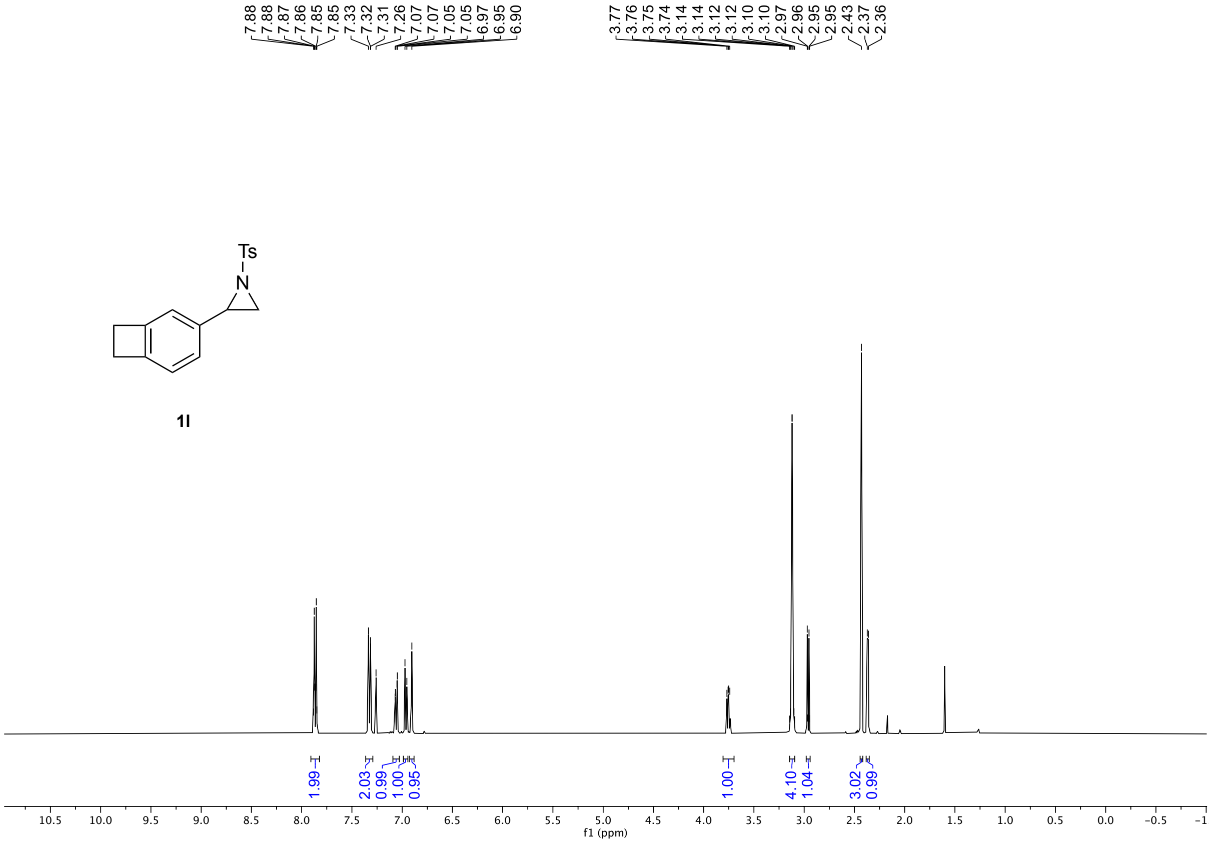
7.88 7.88 7.87 7.86 7.85 7.85 7.33 7.32 7.31 7.26 7.07 7.07 7.05 7.05 6.97 6.95 6.90
3.77 3.76 3.75 3.74 3.14 3.14 3.12 3.12 3.10 3.10 2.97 2.96 2.95 2.95 2.43 2.37 2.36
Ts
N
1l
1.99 2.03 0.99 1.00 0.95 1.00 4.10 1.04 3.02 0.99
f1 (ppm)

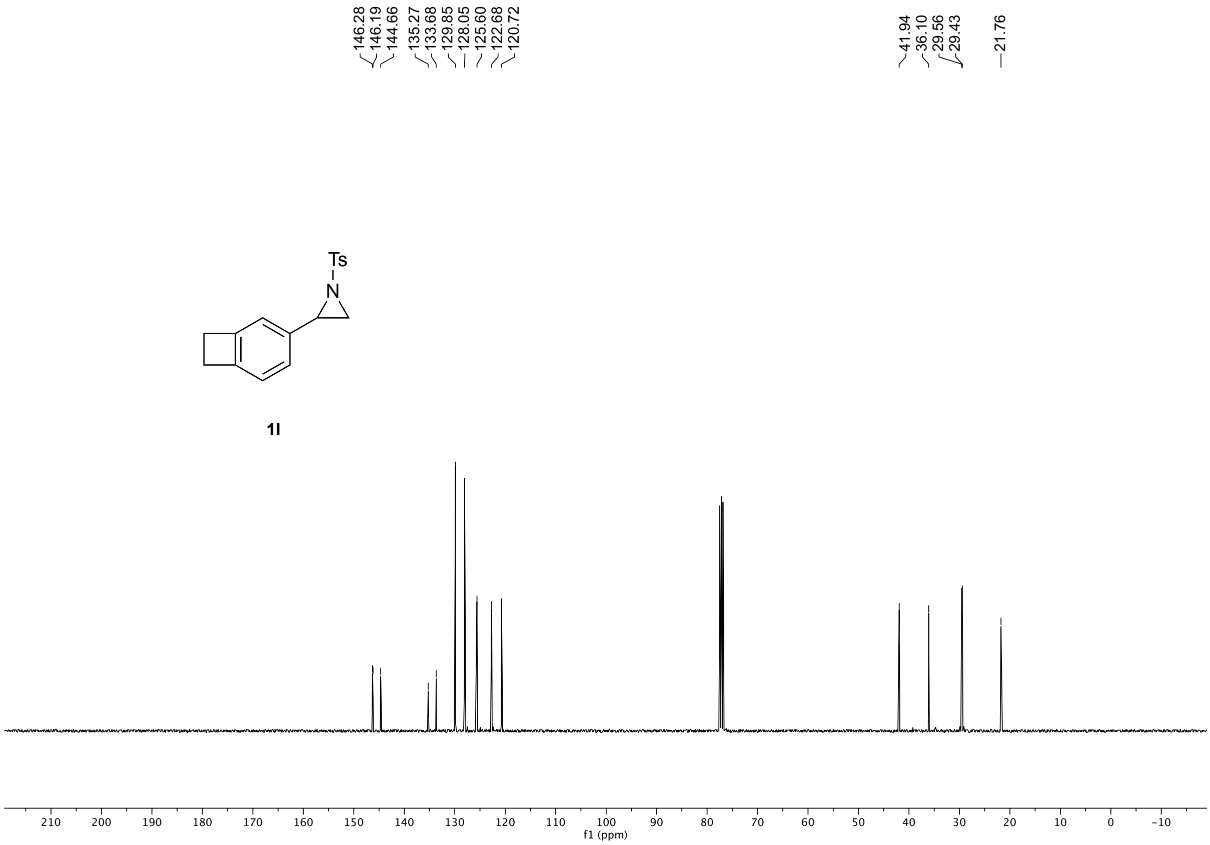
146.28 146.19 144.66 135.27 133.68 129.85 128.05 125.60 122.68 120.72
41.94 36.10 29.56 29.43 21.76
Ts
N
1l
f1 (ppm)

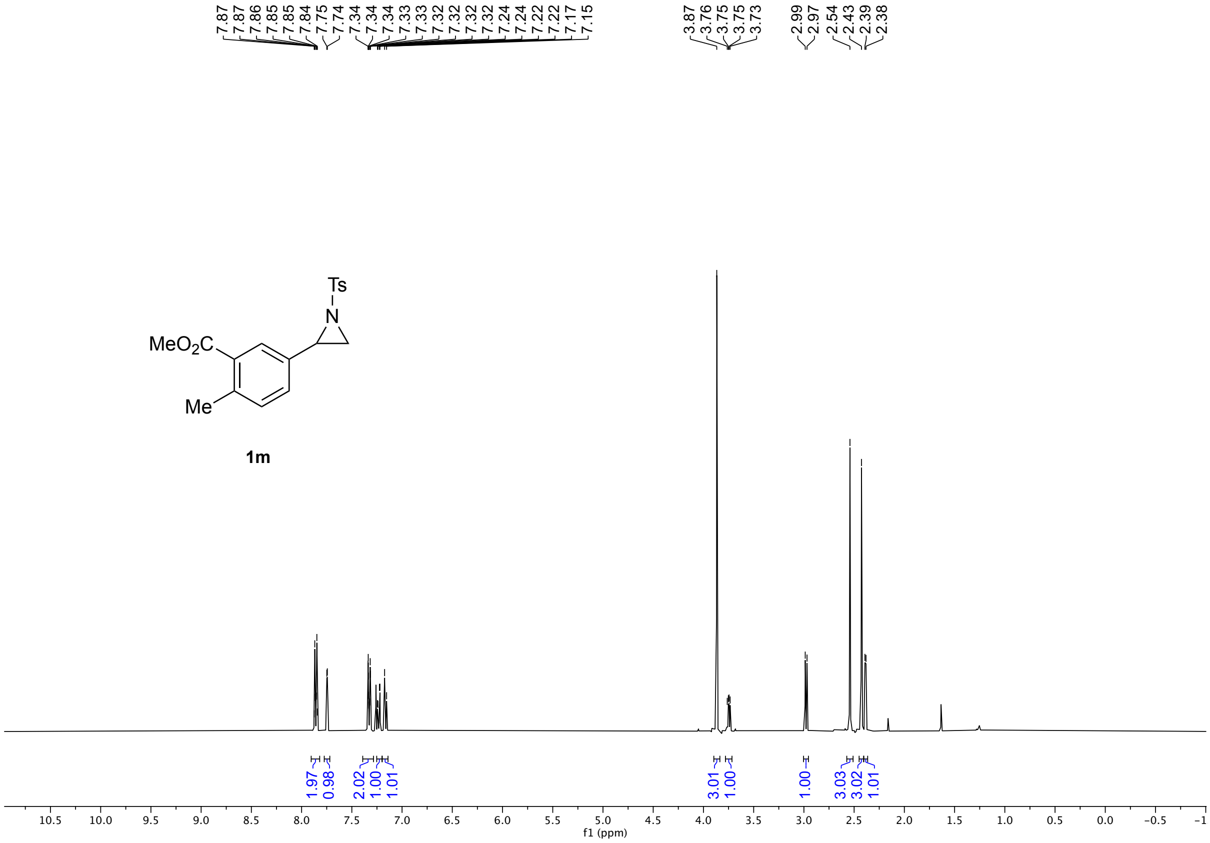
7.87
7.87
7.86
7.85
7.85
7.84
7.75
7.74
7.34
7.34
7.34
7.33
7.33
7.32
7.32
7.32
7.32
7.24
7.24
7.22
7.22
7.17
7.15
3.87
3.76
3.75
3.75
3.73
2.99
2.97
2.54
2.43
2.39
2.38
Ts
N
MeO2C
Me
1m
1.97
0.98
2.02
1.00
1.01
3.01
1.00
1.00
3.03
3.02
1.01
f1 (ppm)

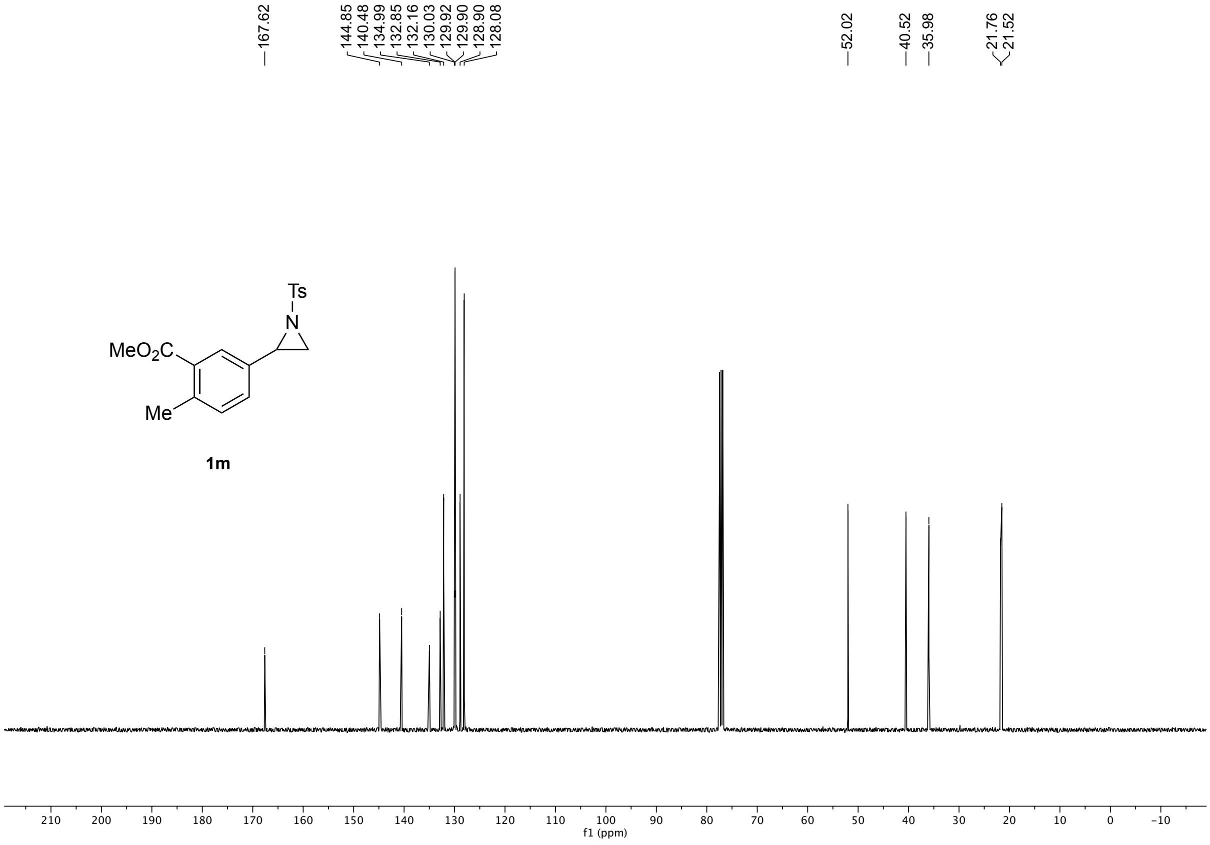
167.62
144.85
140.48
134.99
132.85
132.16
130.03
129.92
129.90
128.90
128.08
52.02
40.52
35.98
21.76
21.52
Ts
N
MeO2C
Me
1m
f1 (ppm)

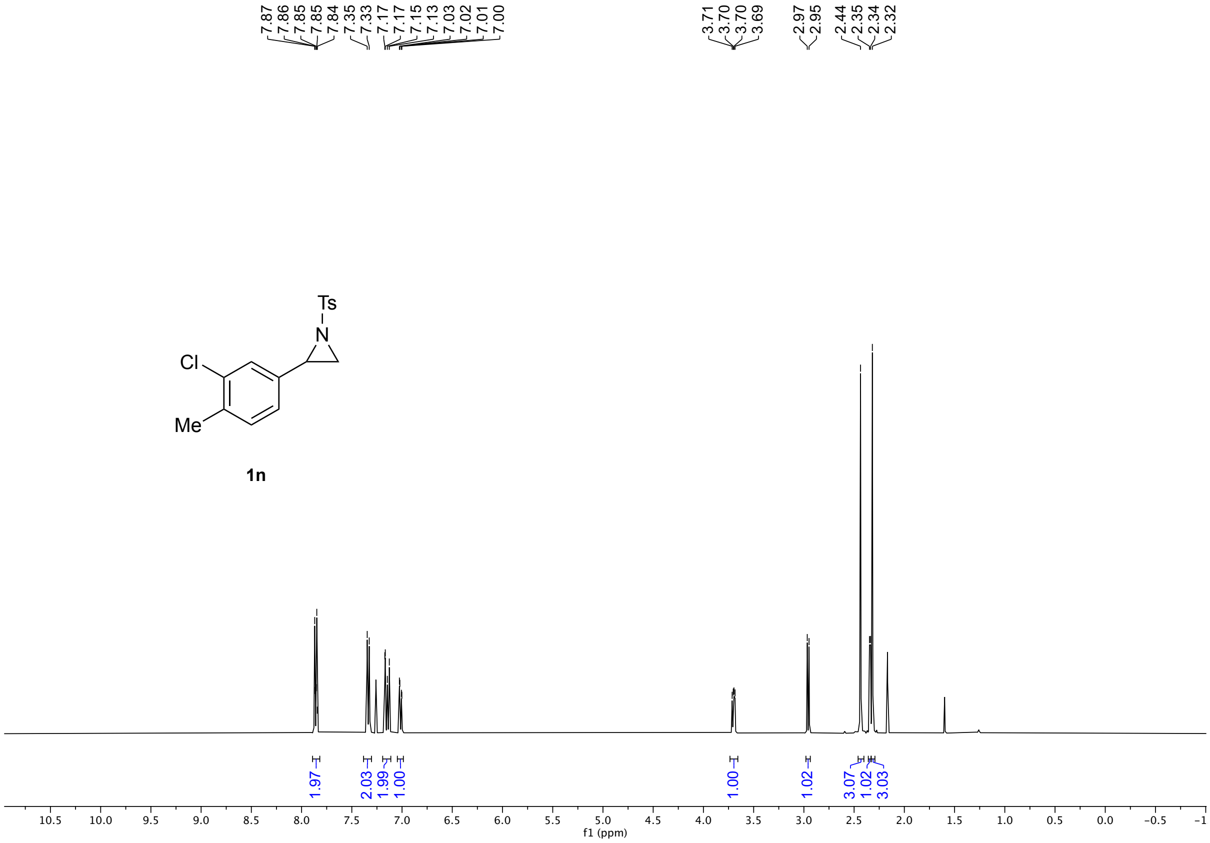
7.87
7.86
7.85
7.85
7.84
7.35
7.33
7.17
7.17
7.15
7.13
7.03
7.02
7.01
7.00
3.71
3.70
3.70
3.69
2.97
2.95
2.44
2.35
2.34
2.32
Ts
N
Cl
Me
1n
1.97
2.03
1.99
1.00
1.00
1.02
3.07
1.02
3.03
f1 (ppm)

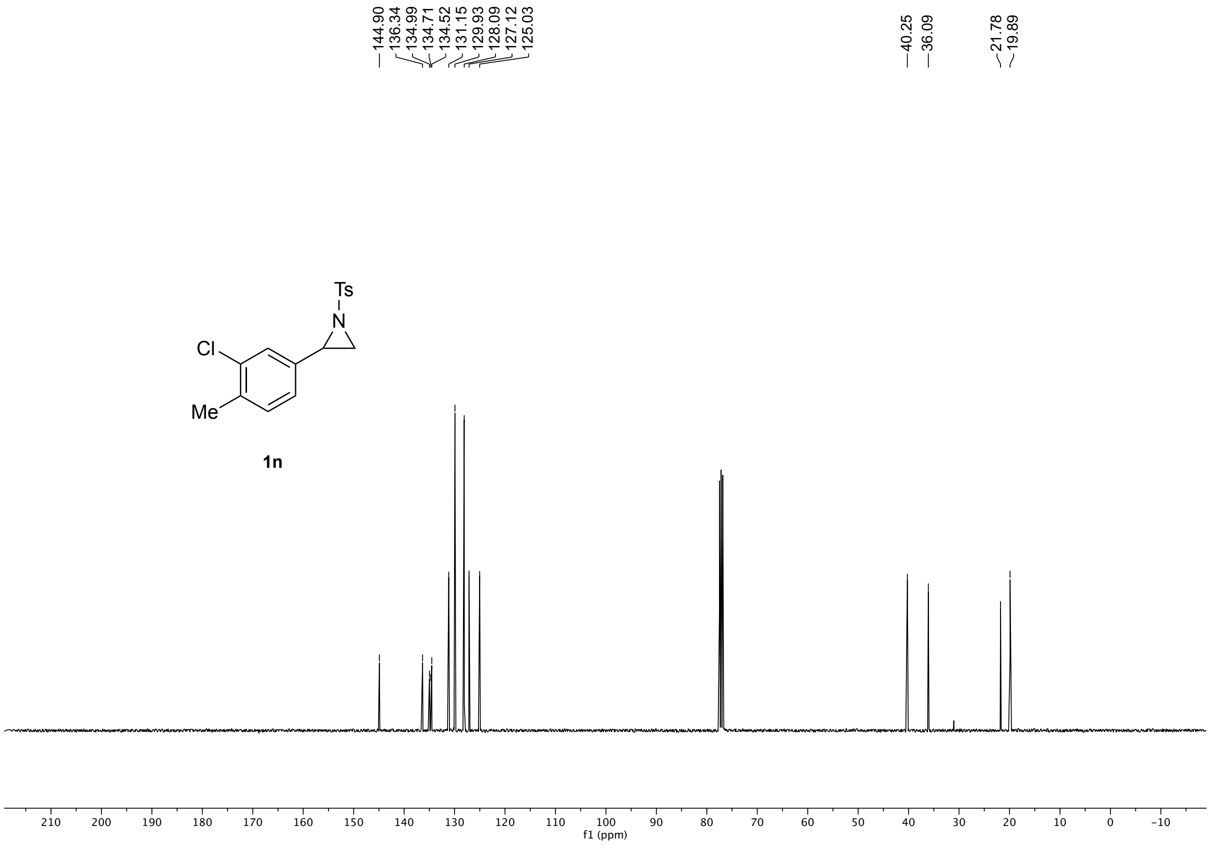
144.90
136.34
134.99
134.71
134.52
131.15
129.93
128.09
127.12
125.03
40.25
36.09
21.78
19.89
Ts
N
Cl
Me
1n
f1 (ppm)

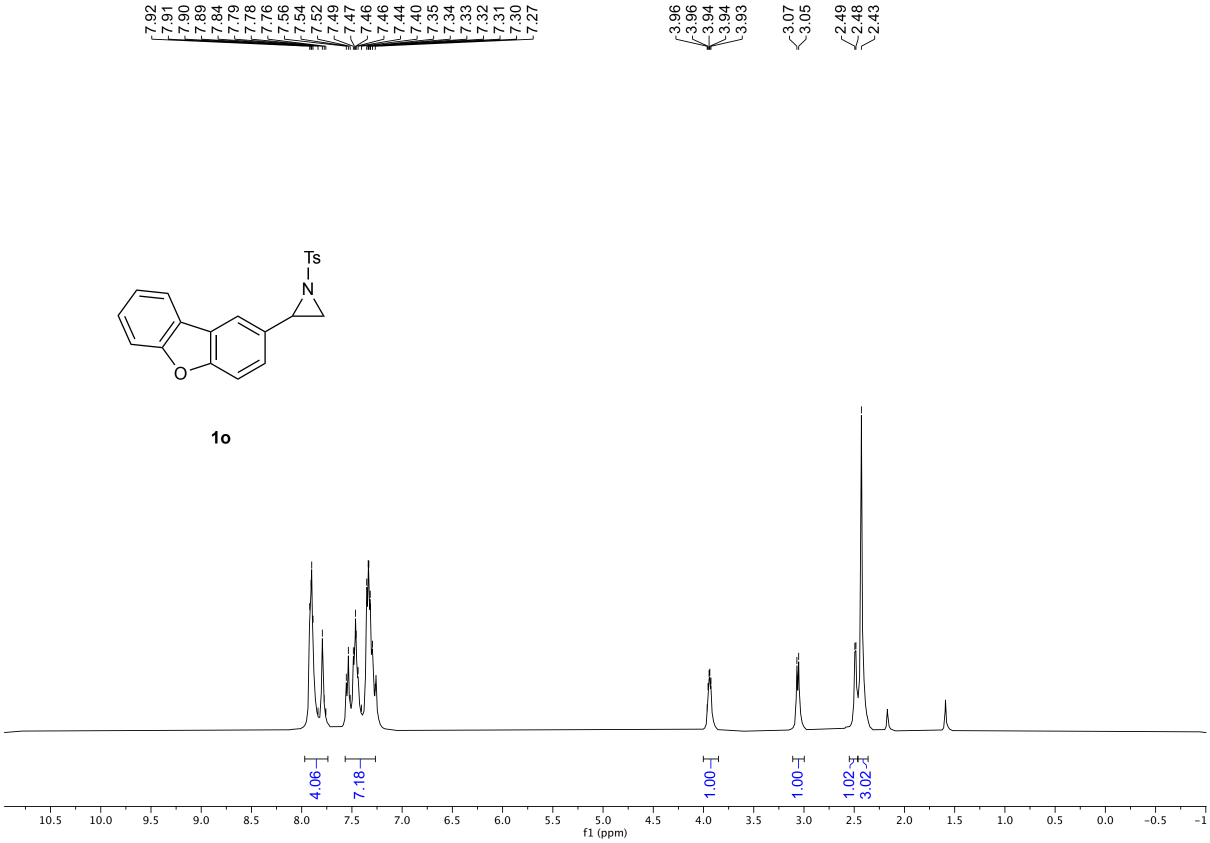
7.92 7.91 7.90 7.89 7.84 7.79 7.78 7.76 7.56 7.54 7.52 7.49 7.47 7.46 7.46 7.44 7.40 7.35 7.34 7.33 7.32 7.31 7.30 7.27
3.96 3.96 3.94 3.94 3.93
3.07 3.05
2.49 2.48 2.43
Ts
N
O
1o
4.06
7.18
1.00
1.00
1.02
3.02
f1 (ppm)

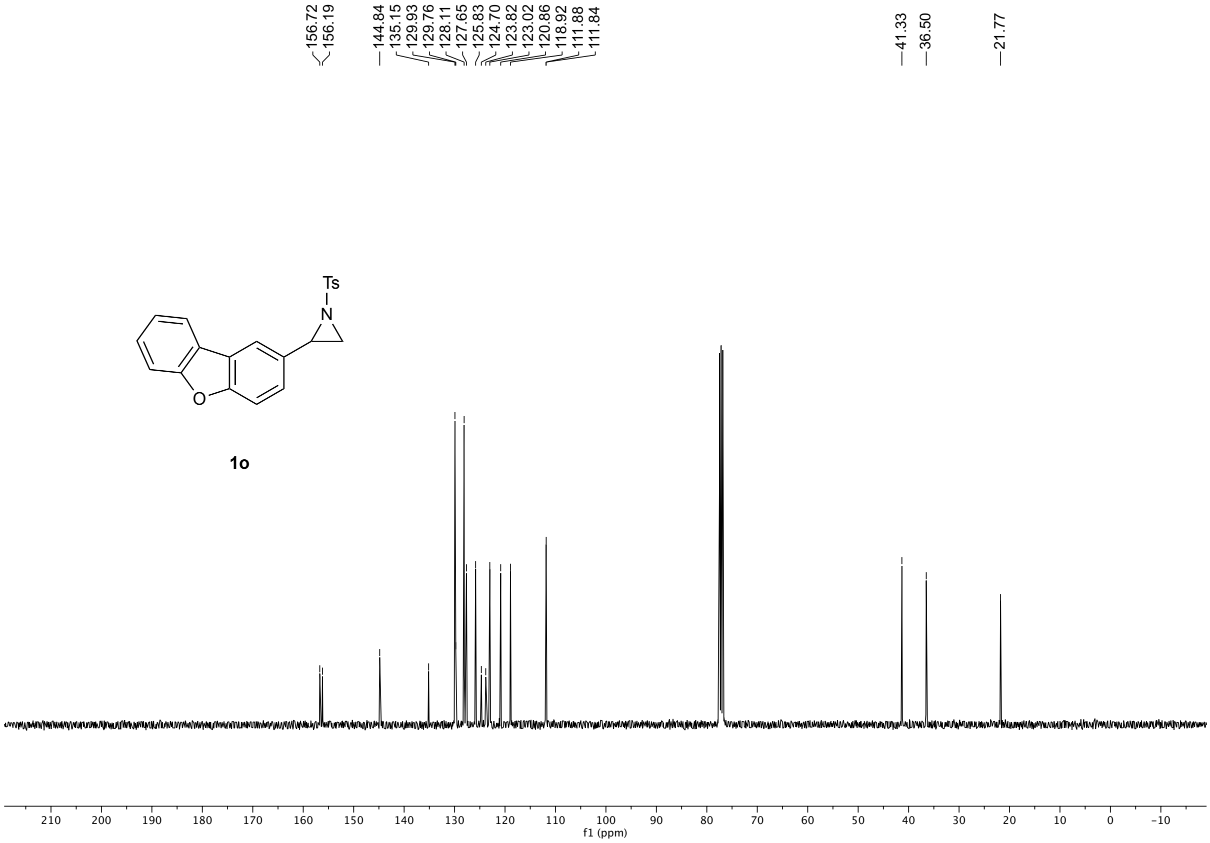
156.72 156.19
144.84 135.15 129.93 129.76 128.11 127.65 125.83 124.70 123.82 123.02 120.86 118.92 111.88 111.84
41.33
36.50
21.77
Ts
N
O
1o
f1 (ppm)

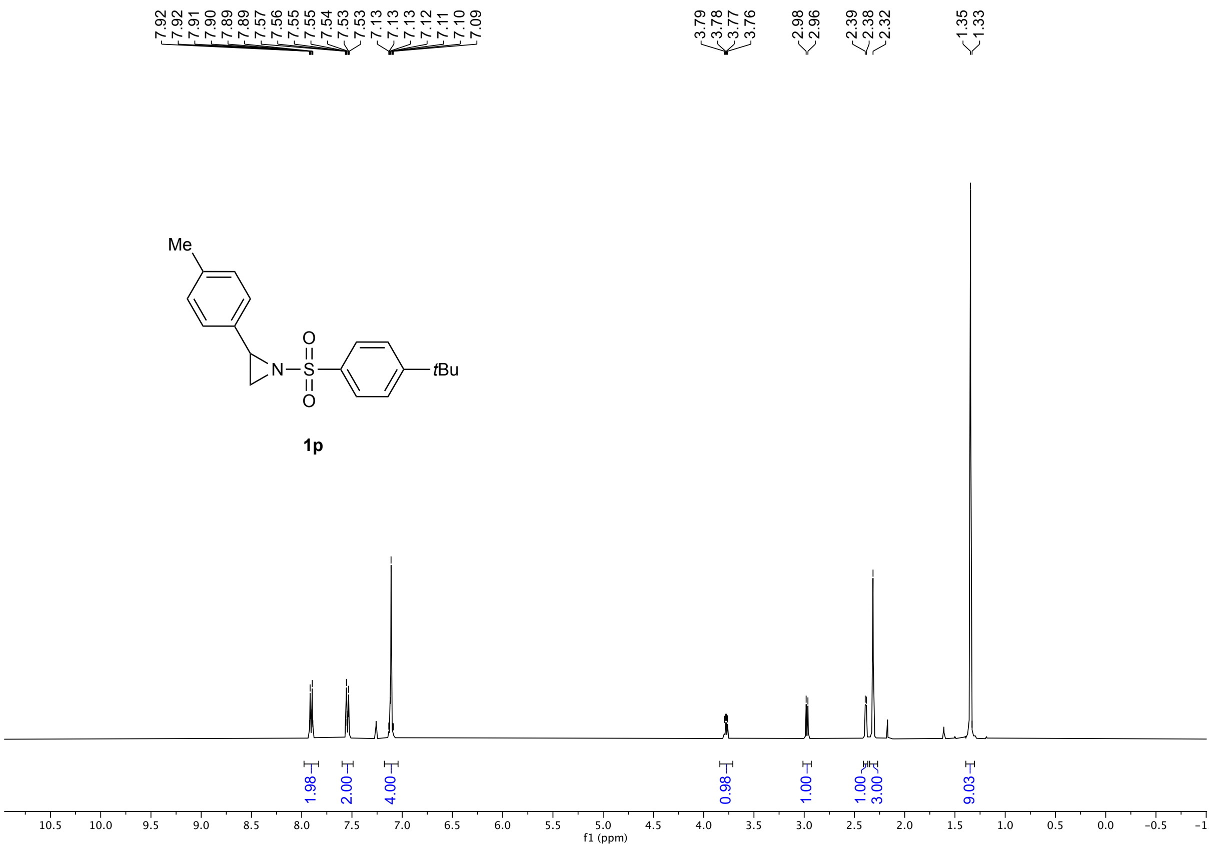
7.92
7.92
7.91
7.90
7.89
7.89
7.57
7.56
7.55
7.55
7.54
7.53
7.53
7.13
7.13
7.13
7.12
7.11
7.10
7.09
3.79
3.78
3.77
3.76
2.98
2.96
2.39
2.38
2.32
1.35
1.33
Me
N
S
O
O
tBu
1p
1.98
2.00
4.00
0.98
1.00
1.00
3.00
9.03
f1 (ppm)

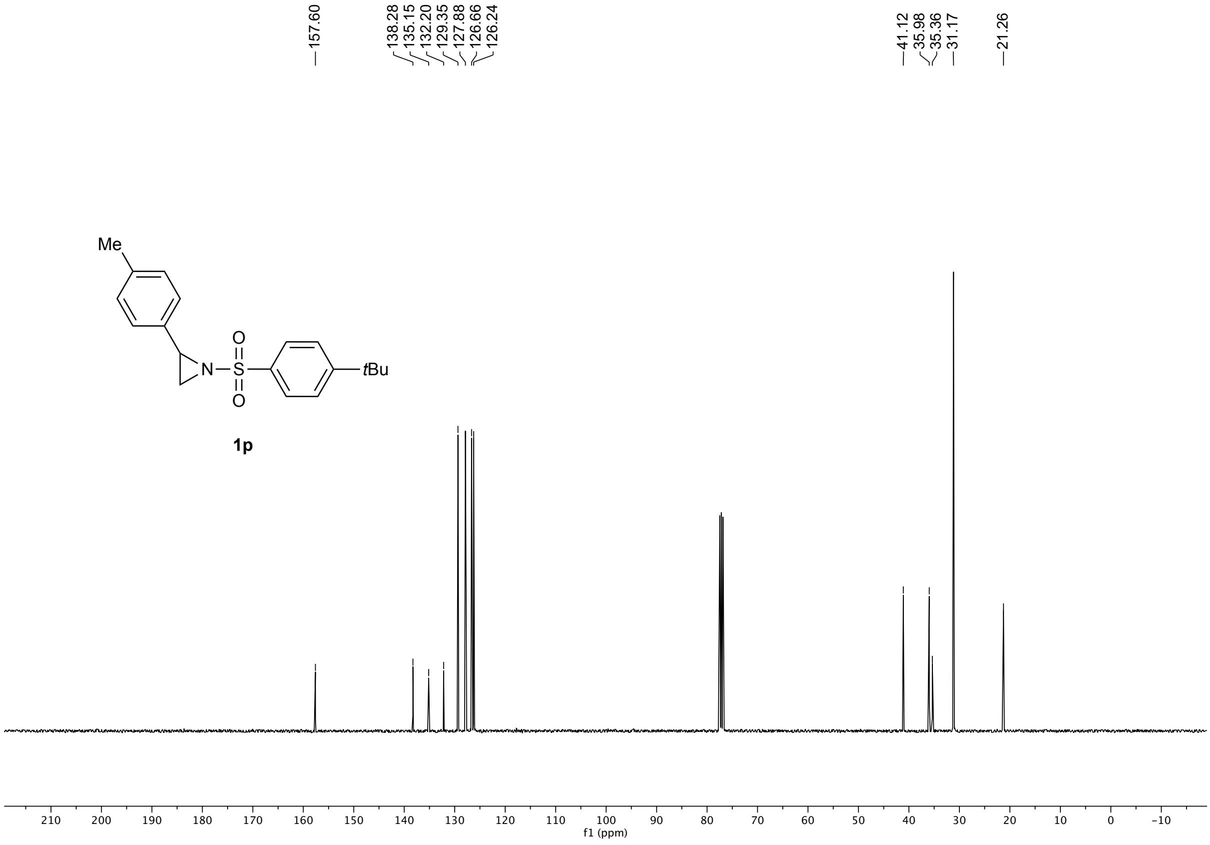
157.60
138.28
135.15
132.20
129.35
127.88
126.66
126.24
41.12
35.98
35.36
31.17
21.26
Me
N
S
O
O
tBu
1p
f1 (ppm)

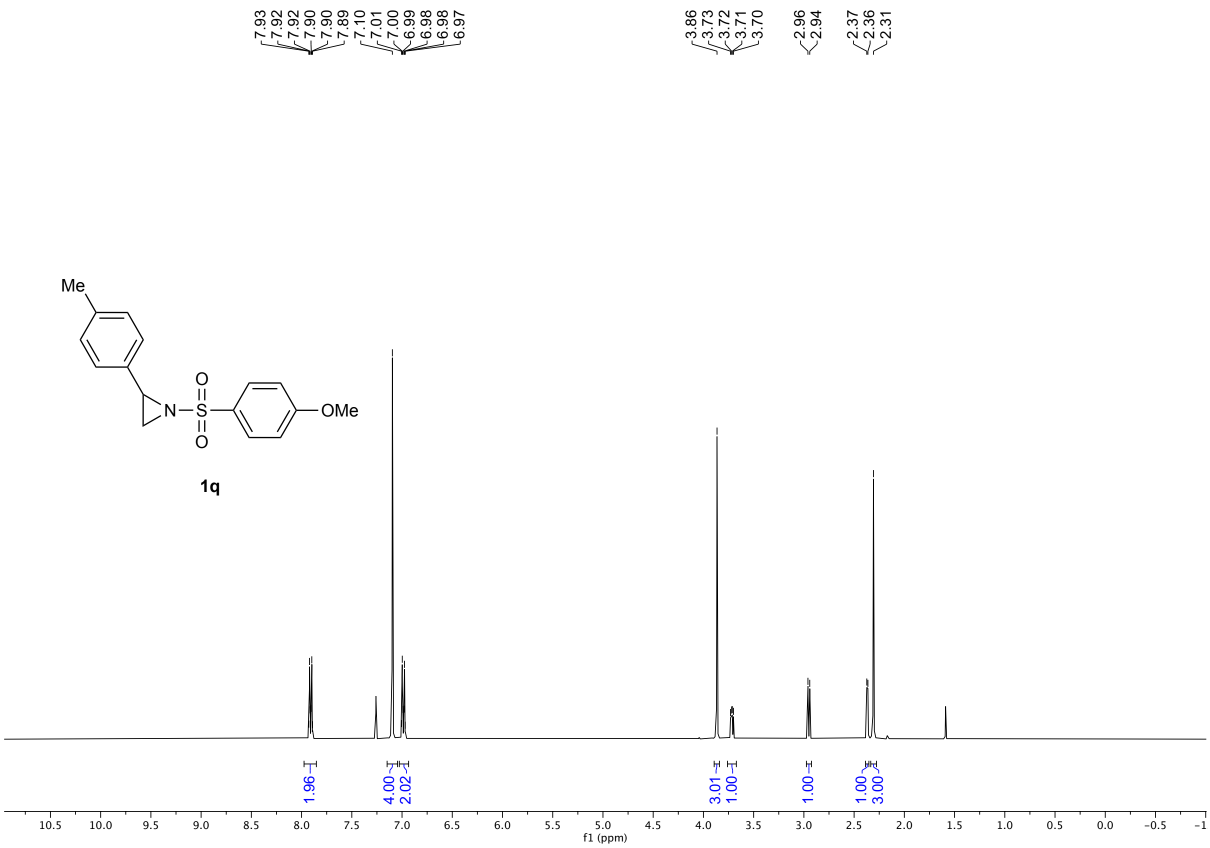
7.93 7.92 7.92 7.90 7.90 7.89 7.10 7.01 7.00 6.99 6.98 6.98 6.97
3.86 3.73 3.72 3.71 3.70
2.96 2.94
2.37 2.36 2.31
Me
O
N
S
O
OMe
1q
1.96
4.00
2.02
3.01
1.00
1.00
1.00
3.00
10.5 10.0 9.5 9.0 8.5 8.0 7.5 7.0 6.5 6.0 5.5 5.0 4.5 4.0 3.5 3.0 2.5 2.0 1.5 1.0 0.5 0.0 -0.5 -1
f1 (ppm)

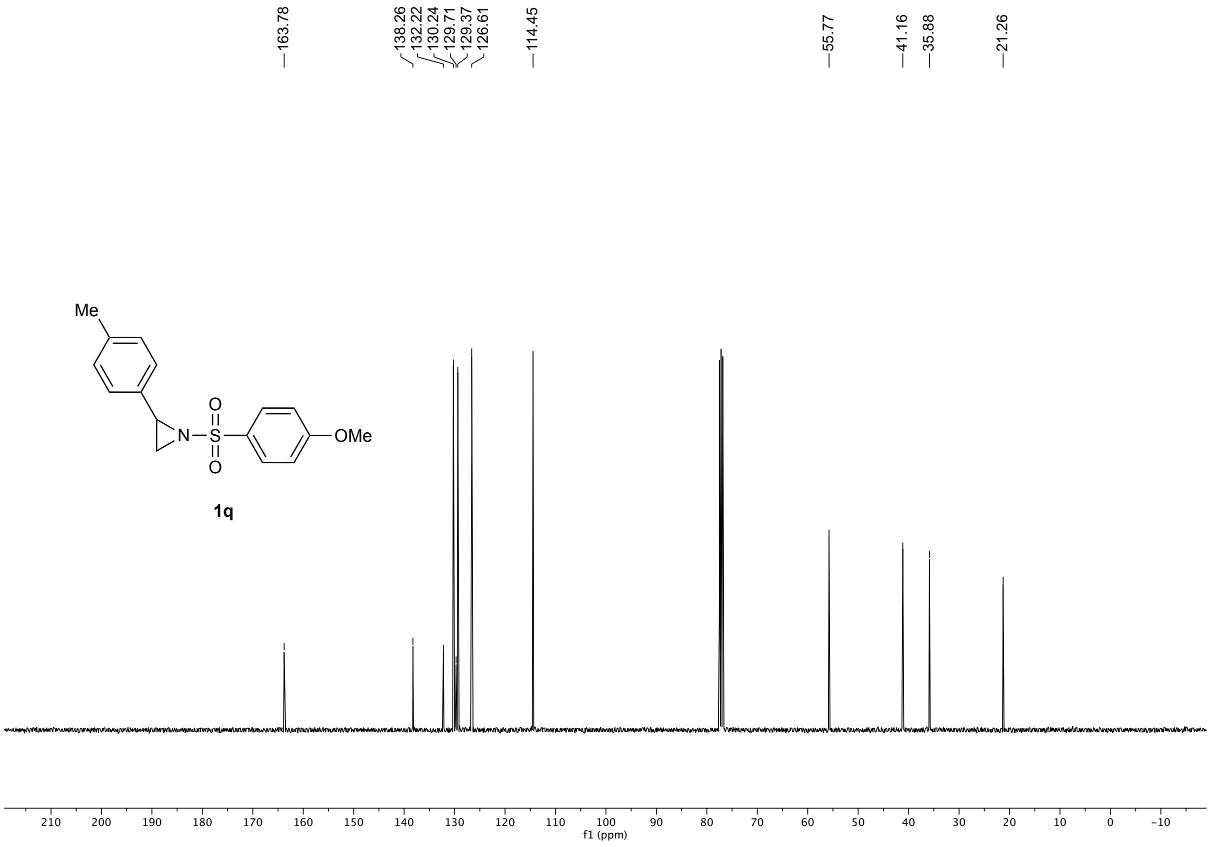
163.78
138.26 132.22 130.24 129.71 129.37 126.61
114.45
55.77
41.16
35.88
21.26
Me
O
N
S
O
OMe
1q
210 200 190 180 170 160 150 140 130 120 110 100 90 80 70 60 50 40 30 20 10 0 -10
f1 (ppm)

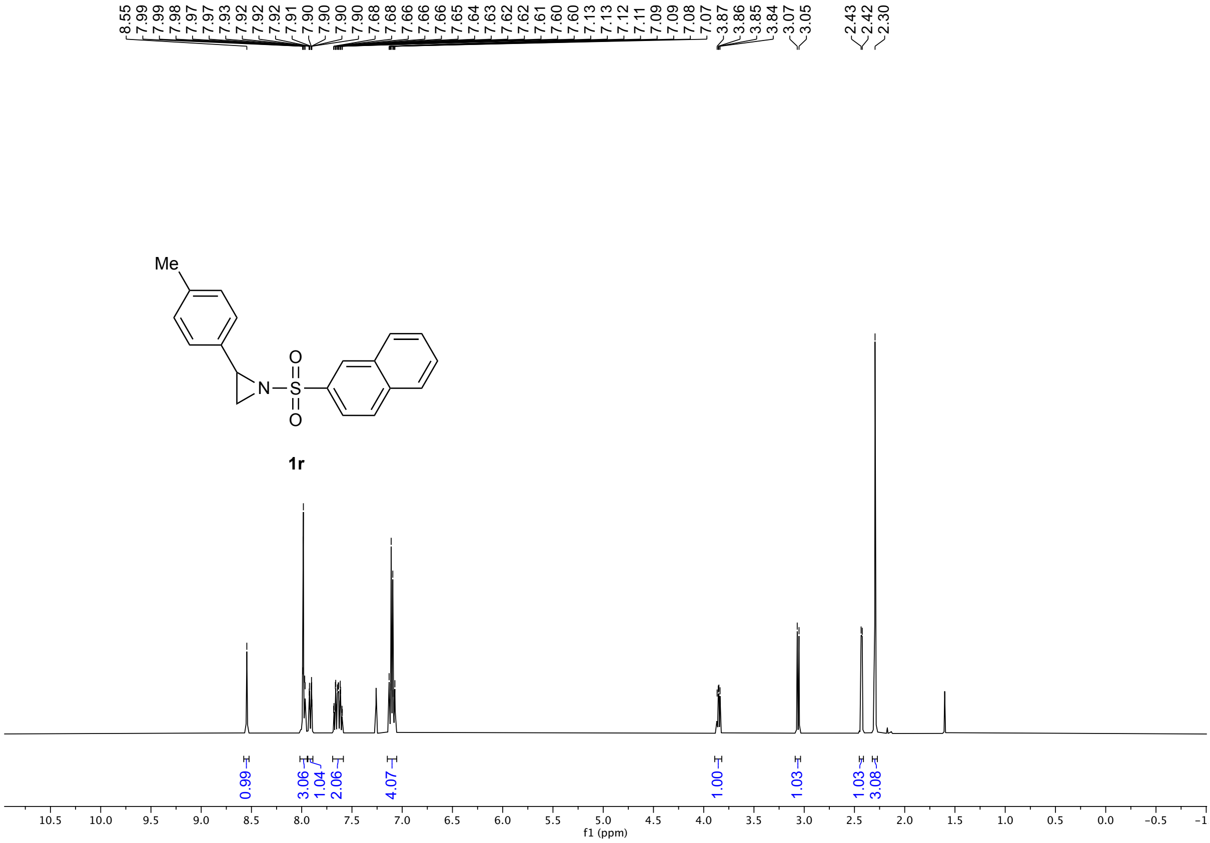
8.55
7.99
7.99
7.98
7.97
7.97
7.93
7.92
7.92
7.92
7.91
7.90
7.90
7.90
7.90
7.68
7.66
7.66
7.66
7.65
7.64
7.63
7.62
7.62
7.61
7.60
7.13
7.13
7.12
7.11
7.09
7.09
7.08
7.07
3.87
3.86
3.85
3.84
3.07
3.05
2.43
2.42
2.30
Me
N
S
O
O
1r
0.99
3.06
1.04
2.06
4.07
1.00
1.03
1.03
3.08
f1 (ppm)

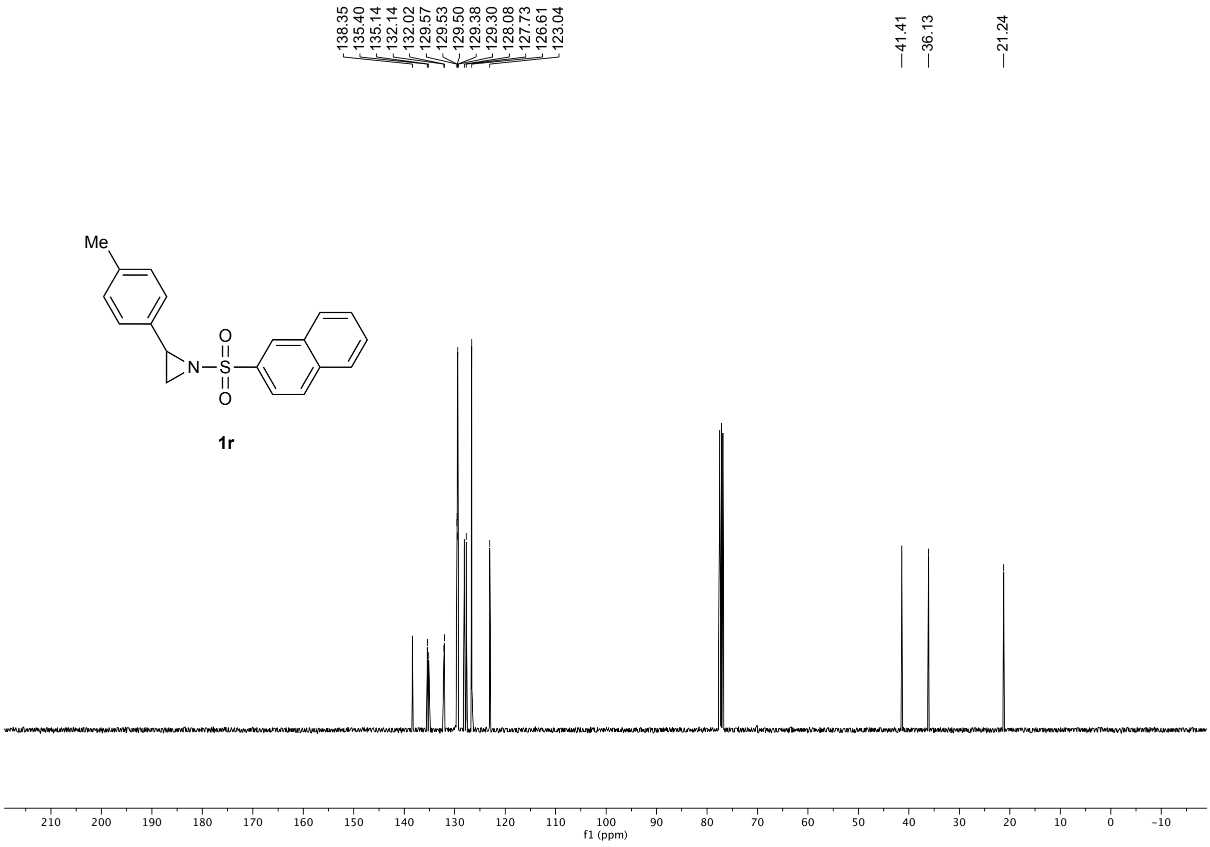
138.35
135.40
135.14
132.14
132.02
129.57
129.53
129.50
129.38
129.30
128.08
127.73
126.61
123.04
41.41
36.13
21.24
Me
N
S
O
O
1r
f1 (ppm)

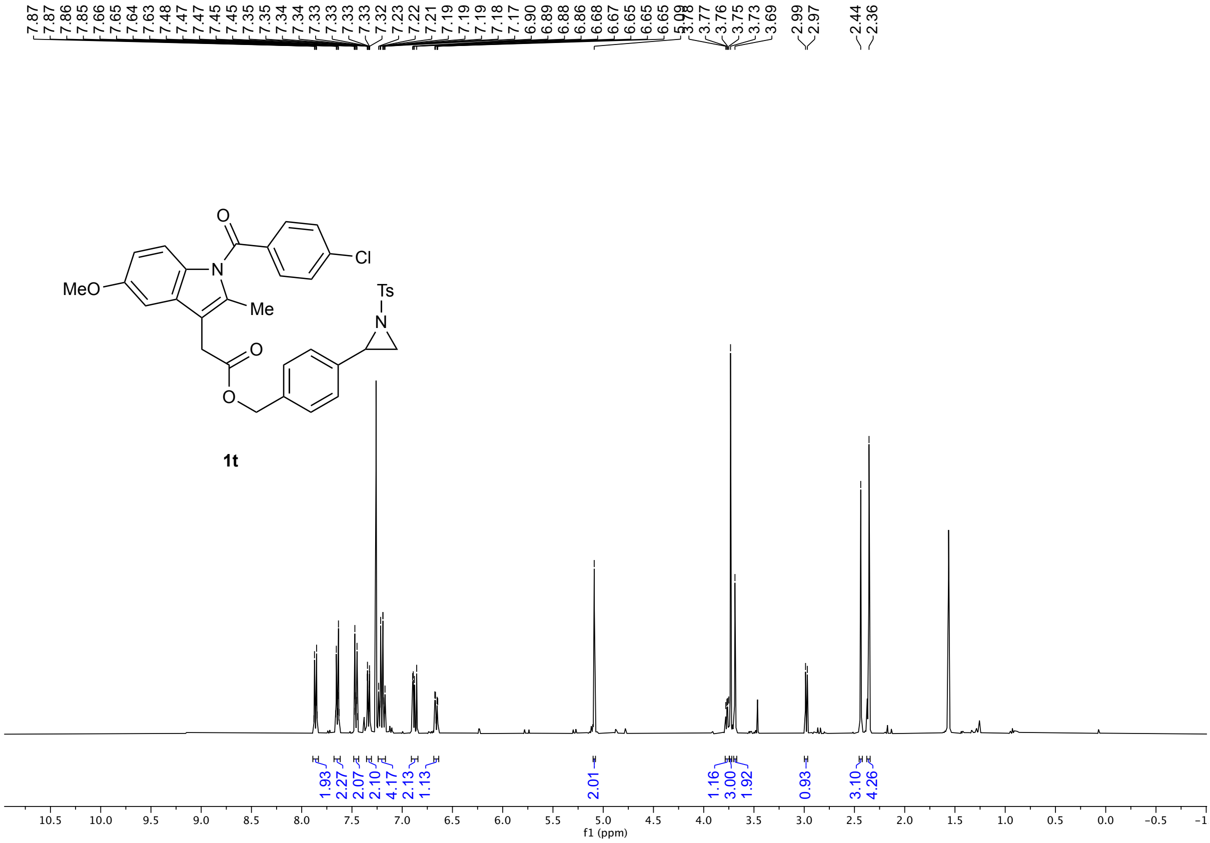
MeO
Cl
Ts
Me
1t
f1 (ppm)

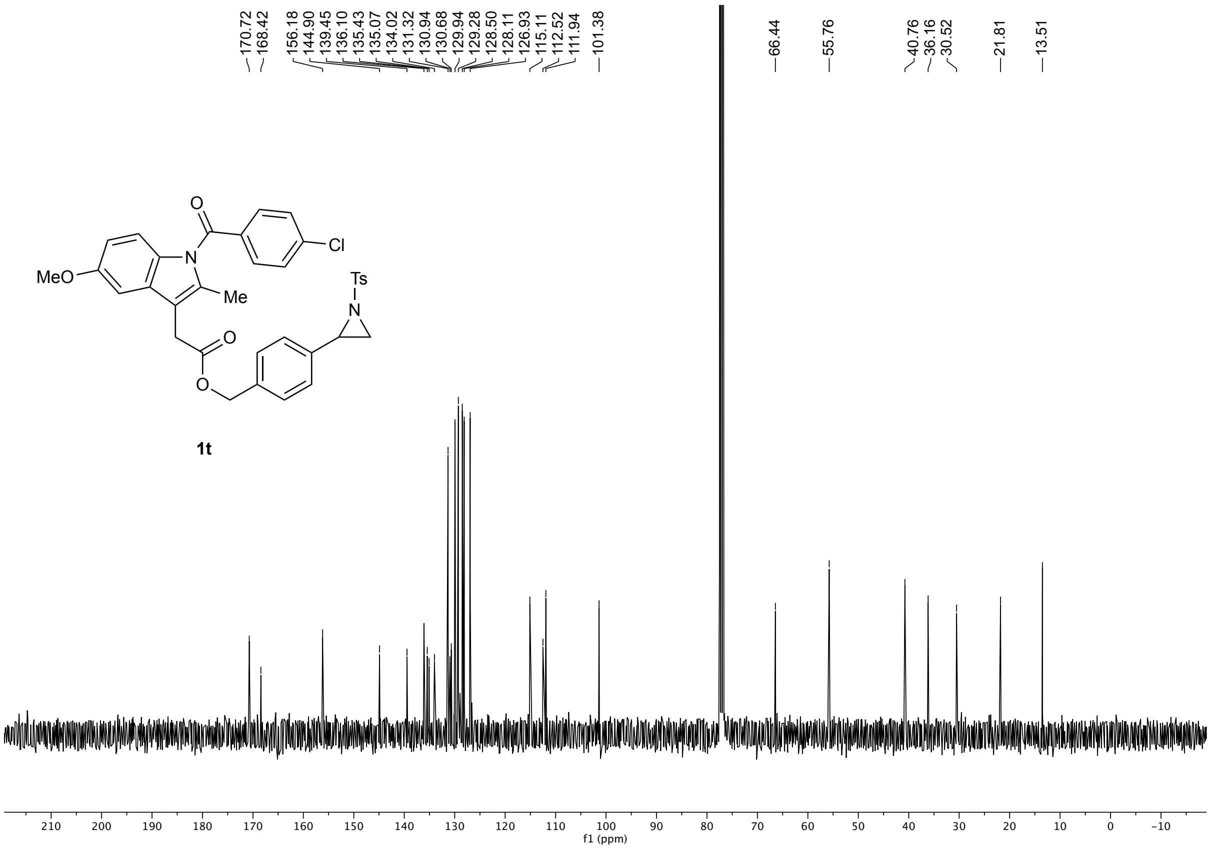
MeO
Cl
Ts
Me
1t
f1 (ppm)

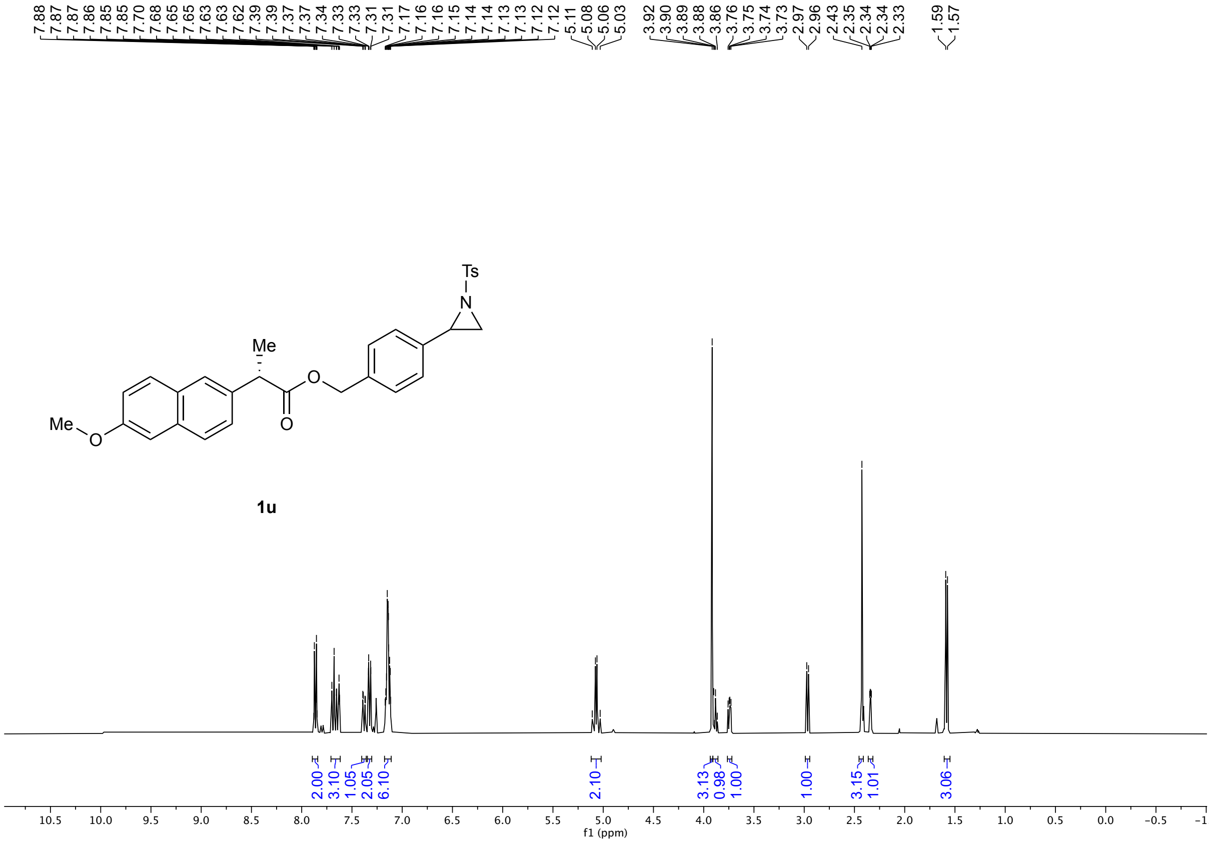
7.88
7.87
7.87
7.86
7.85
7.85
7.70
7.68
7.65
7.65
7.63
7.63
7.62
7.39
7.39
7.37
7.37
7.34
7.33
7.33
7.31
7.31
7.17
7.16
7.16
7.15
7.14
7.14
7.13
7.13
7.12
7.12
5.11
5.08
5.06
5.03
3.92
3.90
3.89
3.88
3.86
3.76
3.75
3.74
3.73
2.97
2.96
2.43
2.35
2.34
2.34
2.33
1.59
1.57
Ts
N
Me
O
O
Me
O
1u
2.00
3.10
1.05
2.05
6.10
2.10
3.13
0.98
1.00
1.00
3.15
1.01
3.06
f1 (ppm)

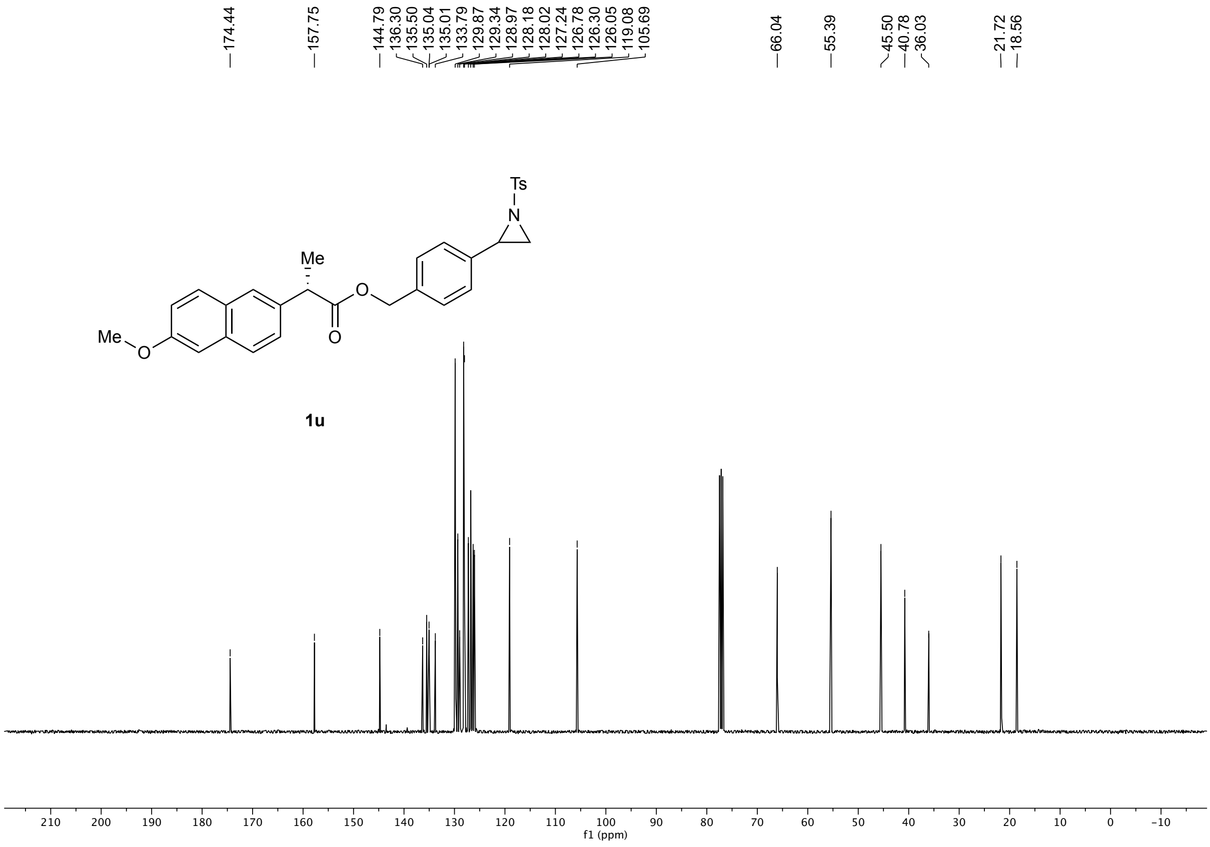
174.44
157.75
144.79
136.30
135.50
135.04
135.01
133.79
129.87
129.34
128.97
128.18
128.02
127.24
126.78
126.30
126.05
119.08
105.69
66.04
55.39
45.50
40.78
36.03
21.72
18.56
Ts
N
Me
O
O
Me
O
1u
f1 (ppm)

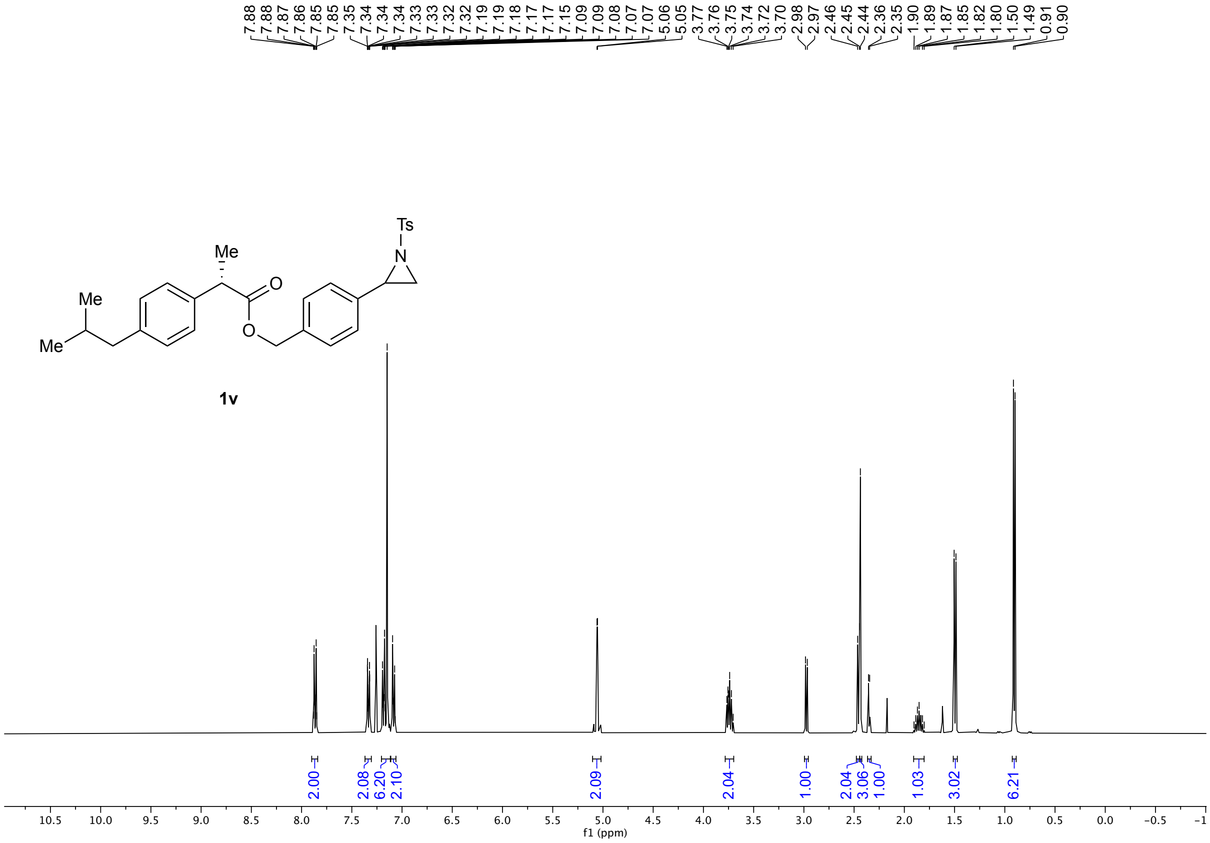
7.88 7.88 7.87 7.86 7.85 7.85 7.35 7.34 7.34 7.34 7.33 7.33 7.32 7.32 7.19 7.19 7.18 7.17 7.17 7.15 7.09 7.08 7.07 7.07 5.06 5.05 3.77 3.76 3.75 3.74 3.72 3.70 2.98 2.97 2.46 2.45 2.44 2.36 2.35 1.90 1.89 1.87 1.85 1.82 1.80 1.50 1.49 0.91 0.90
Ts
N
Me
O
Me
O
Me
1v
2.00 2.08 6.20 2.10 2.09 2.04 1.00 2.04 3.06 1.00 1.03 3.02 6.21
10.5 10.0 9.5 9.0 8.5 8.0 7.5 7.0 6.5 6.0 5.5 5.0 4.5 4.0 3.5 3.0 2.5 2.0 1.5 1.0 0.5 0.0 −0.5 −1
f1 (ppm)

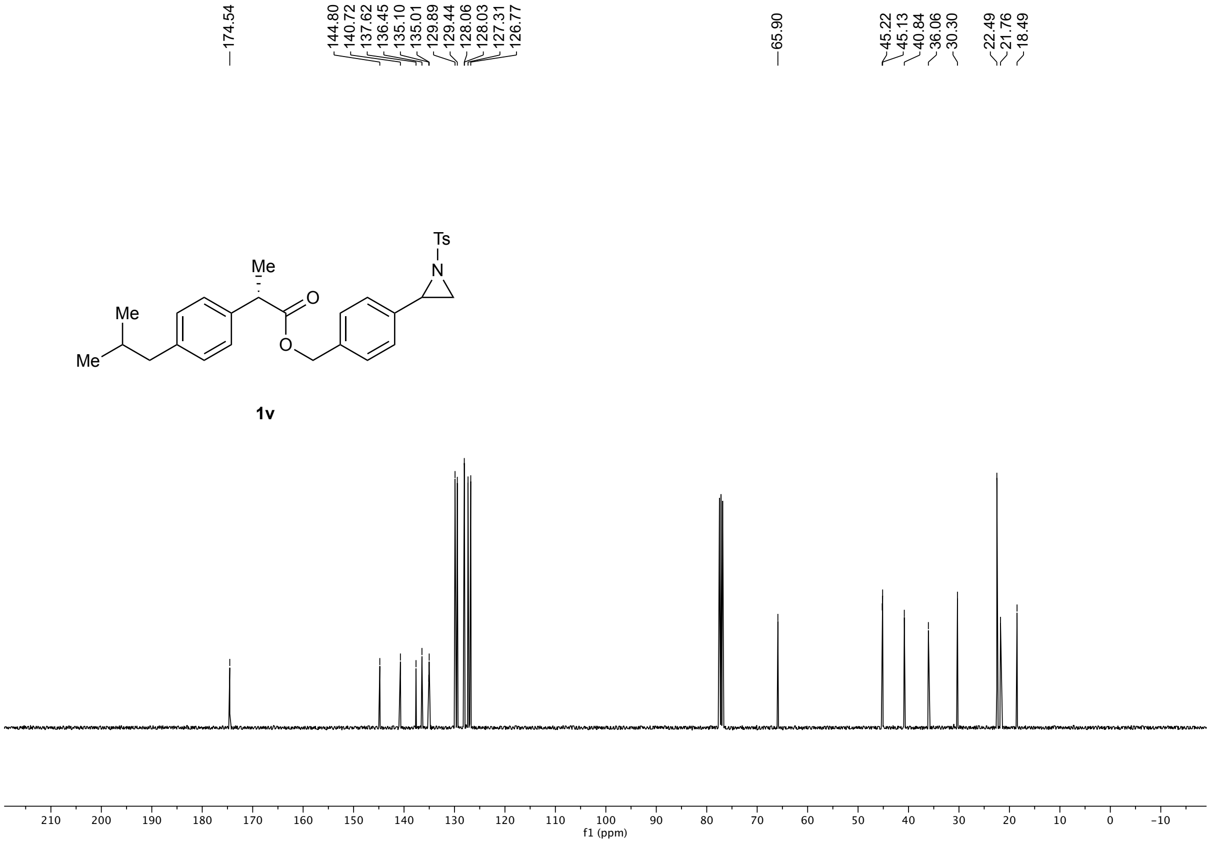
174.54
144.80 140.72 137.62 136.45 135.10 135.01 129.89 129.44 128.06 128.03 127.31 126.77
65.90
45.22 45.13 40.84 36.06 30.30 22.49 21.76 18.49
Ts
N
Me
O
Me
O
Me
1v
210 200 190 180 170 160 150 140 130 120 110 100 90 80 70 60 50 40 30 20 10 0 −10
f1 (ppm)

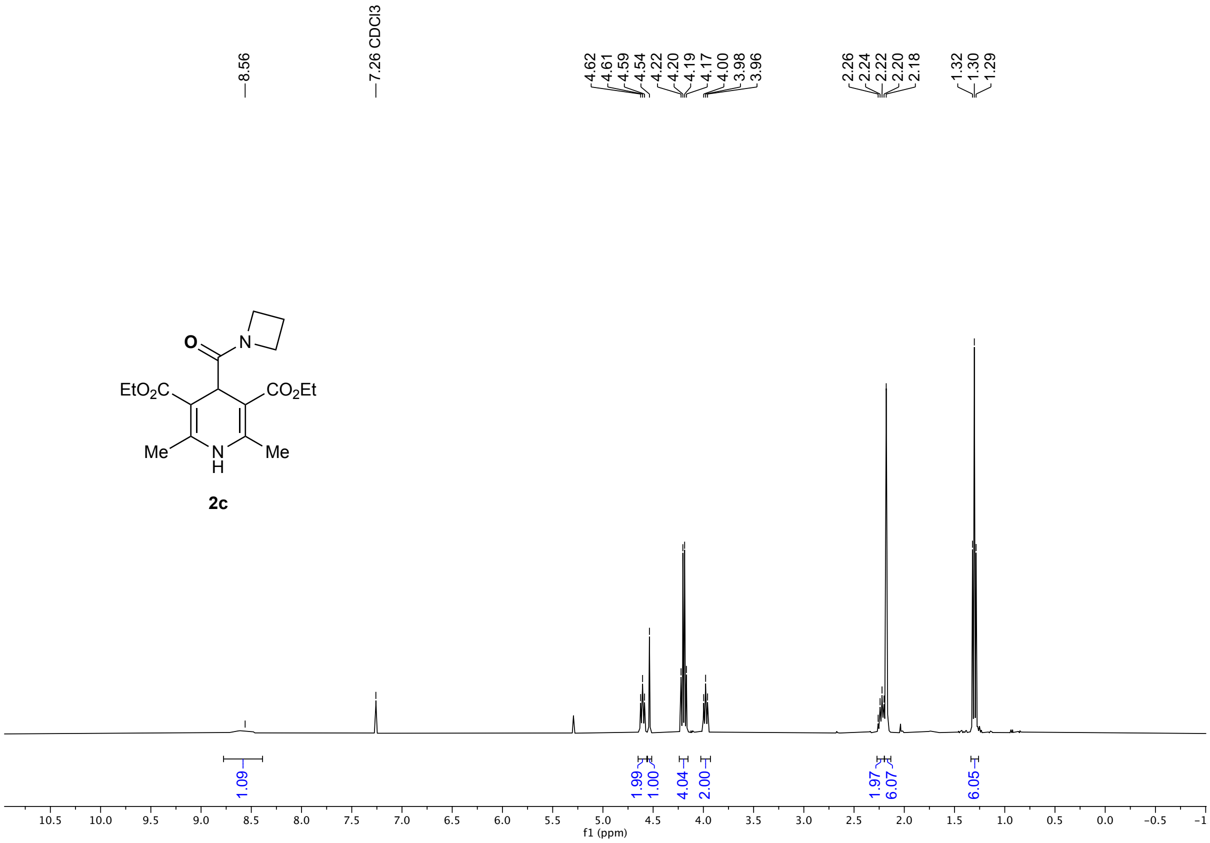
8.56
7.26 CDCl3
4.62
4.61
4.59
4.54
4.22
4.20
4.19
4.17
4.00
3.98
3.96
2.26
2.24
2.22
2.20
2.18
1.32
1.30
1.29
EtO2C
CO2Et
O
N
Me
N
H
Me
2c
1.09
1.99
1.00
4.04
2.00
1.97
6.07
6.05
f1 (ppm)

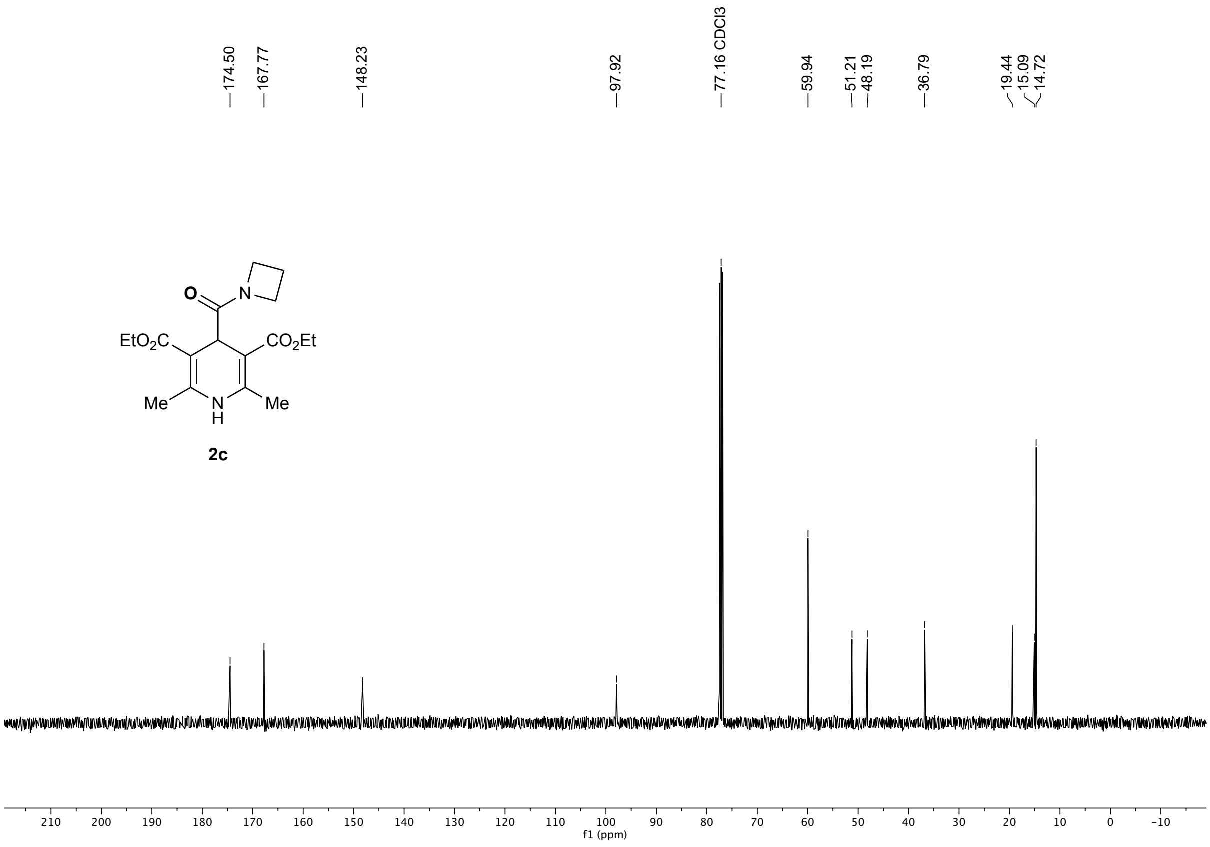
174.50
167.77
148.23
97.92
77.16 CDCl3
59.94
51.21
48.19
36.79
19.44
15.09
14.72
EtO2C
CO2Et
O
N
Me
N
H
Me
2c
f1 (ppm)

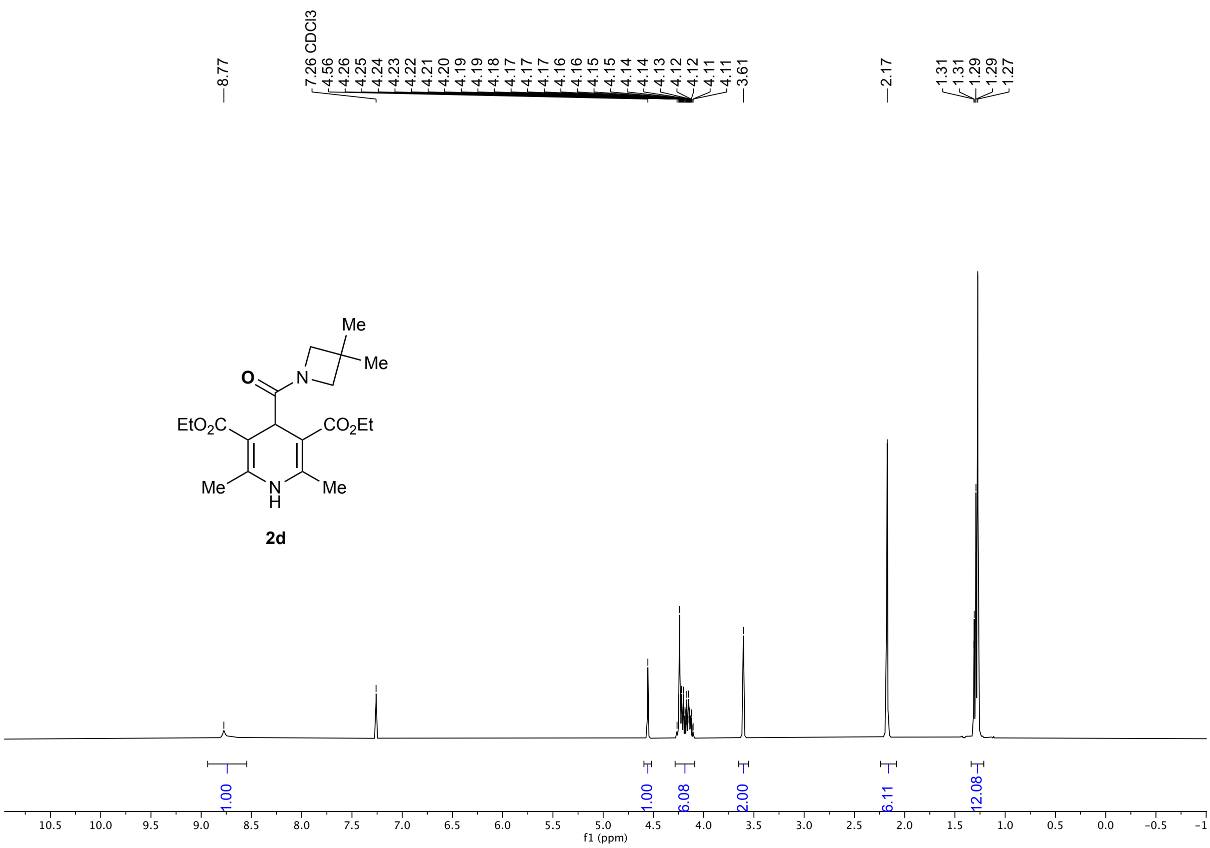
8.77
7.26 CDCl3
4.56
4.26
4.25
4.24
4.23
4.22
4.21
4.20
4.19
4.19
4.18
4.17
4.17
4.17
4.16
4.16
4.15
4.15
4.14
4.14
4.13
4.12
4.12
4.11
4.11
3.61
2.17
1.31
1.31
1.29
1.29
1.27
Me
Me
O
N
EtO2C
CO2Et
Me
N
H
Me
2d
1.00
1.00
6.08
2.00
6.11
12.08
f1 (ppm)

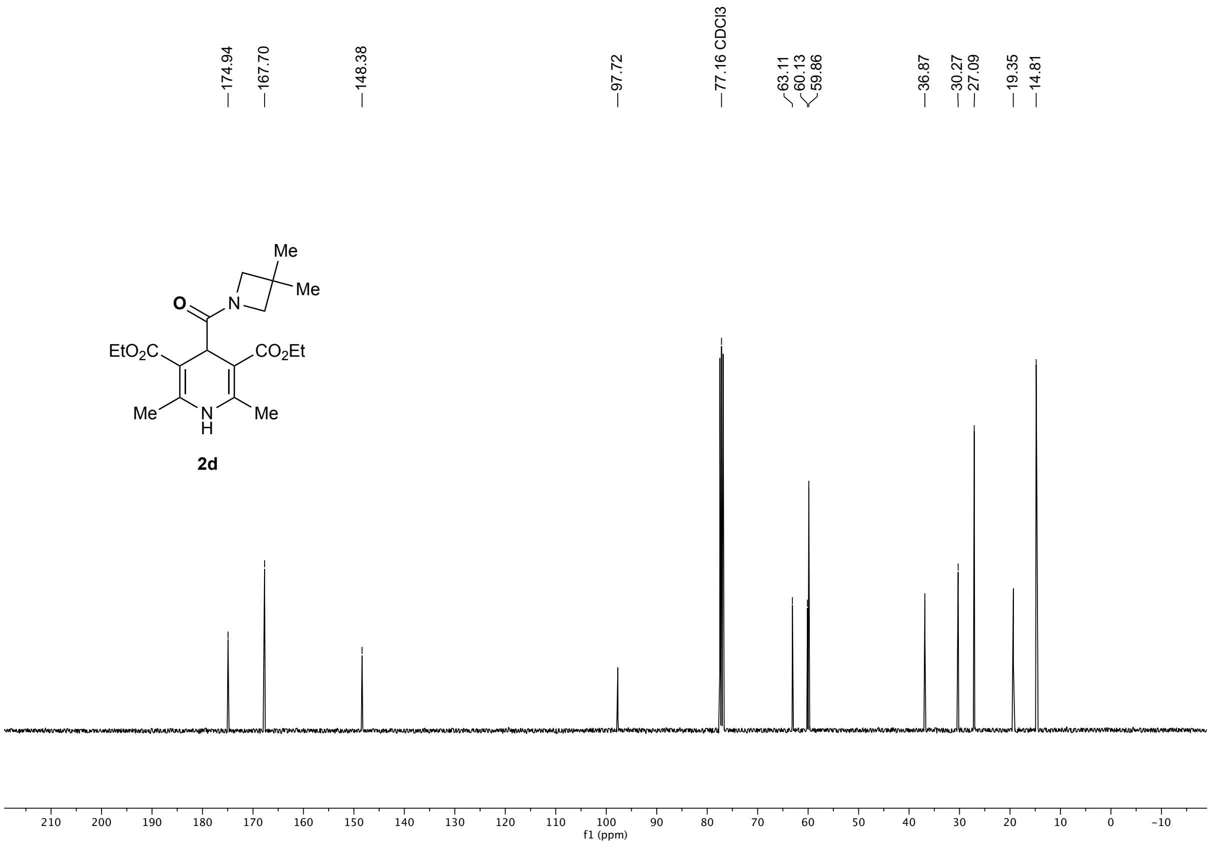
174.94
167.70
148.38
97.72
77.16 CDCl3
63.11
60.13
59.86
36.87
30.27
27.09
19.35
14.81
Me
Me
O
N
EtO2C
CO2Et
Me
N
H
Me
2d
f1 (ppm)

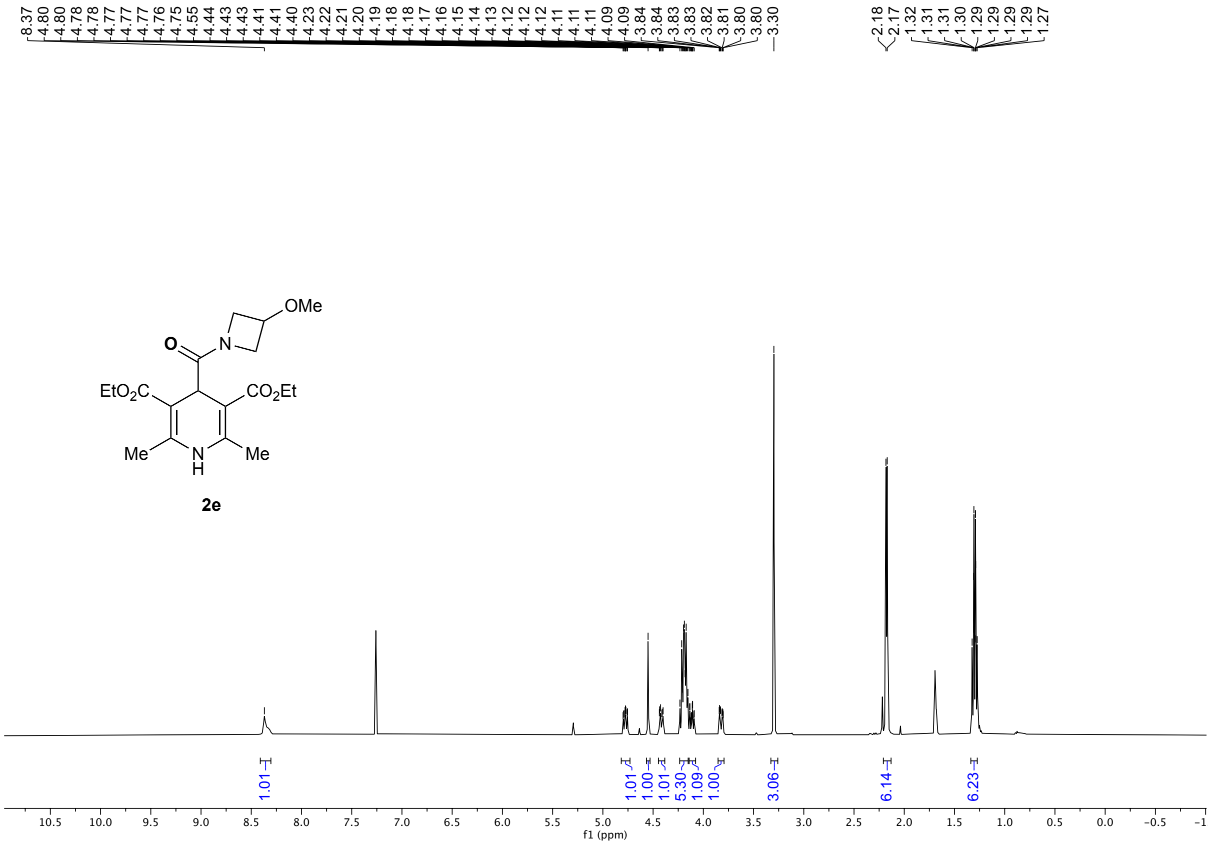
8.37 4.80 4.80 4.78 4.78 4.77 4.77 4.76 4.75 4.55 4.44 4.43 4.43 4.41 4.41 4.40 4.23 4.22 4.21 4.20 4.19 4.18 4.18 4.17 4.16 4.15 4.14 4.13 4.12 4.12 4.12 4.11 4.11 4.11 4.09 4.09 3.84 3.84 3.83 3.83 3.82 3.81 3.80 3.80 3.30
2.18 2.17 1.32 1.31 1.31 1.30 1.29 1.29 1.29 1.29 1.27
OMe
O
N
EtO2C
CO2Et
Me
N
H
Me
2e
1.01
1.01
1.00
1.01
5.30
1.09
1.00
3.06
6.14
6.23
f1 (ppm)

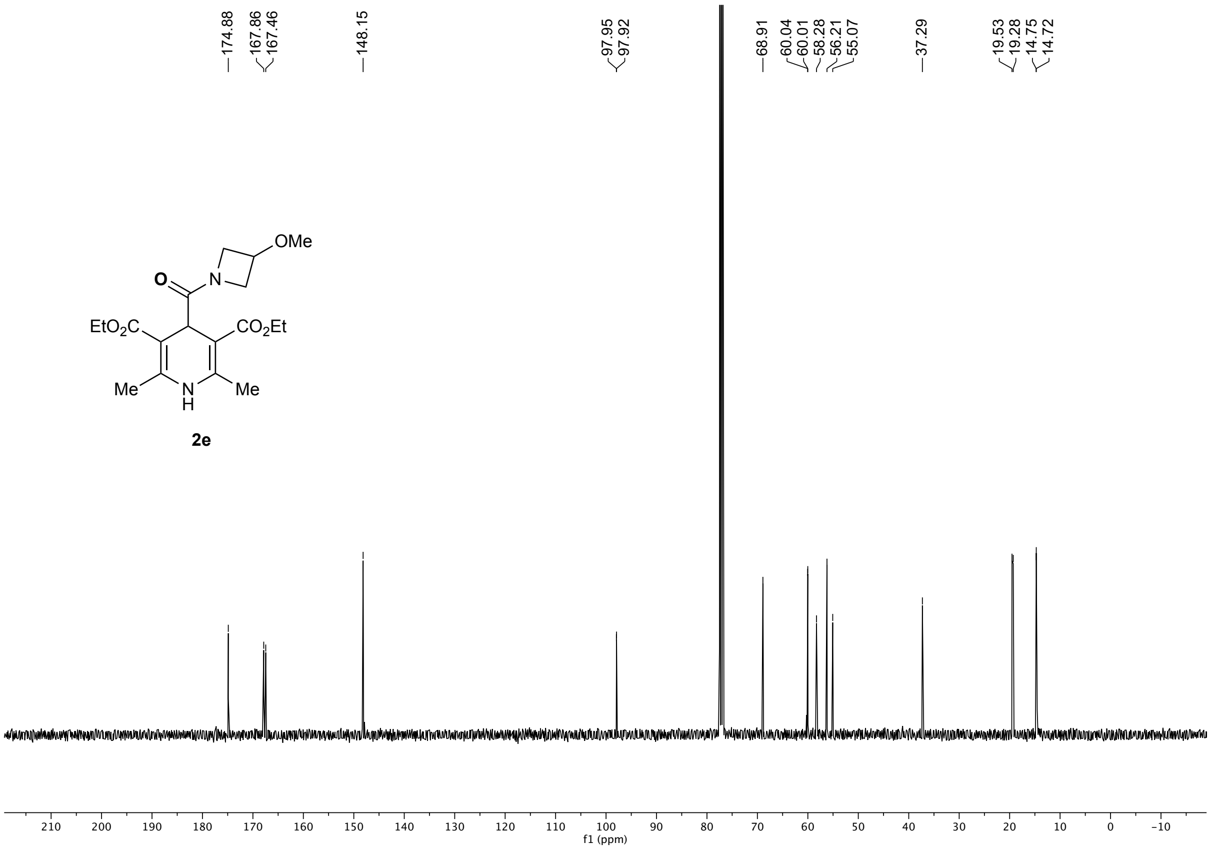
174.88
167.86 167.46
148.15
97.95 97.92
68.91
60.04 60.01 58.28 56.21 55.07
37.29
19.53 19.28 14.75 14.72
OMe
O
N
EtO2C
CO2Et
Me
N
H
Me
2e
f1 (ppm)

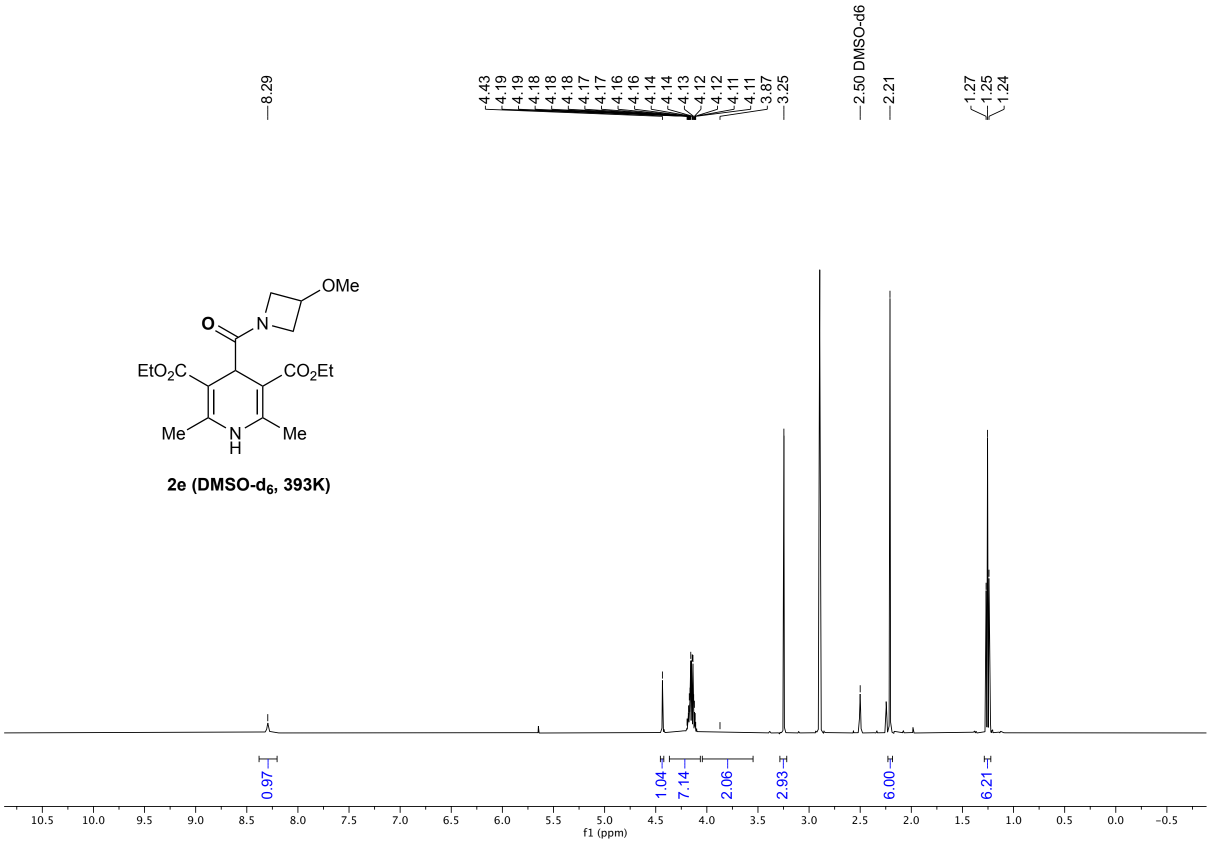
8.29
4.43
4.19
4.19
4.18
4.18
4.17
4.17
4.16
4.16
4.14
4.14
4.13
4.12
4.12
4.11
4.11
3.87
3.25
2.50 DMSO-d6
2.21
1.27
1.25
1.24
OMe
O
N
EtO2C
CO2Et
Me
N
H
Me
2e (DMSO-d6, 393K)
0.97
1.04
7.14
2.06
2.93
6.00
6.21
f1 (ppm)

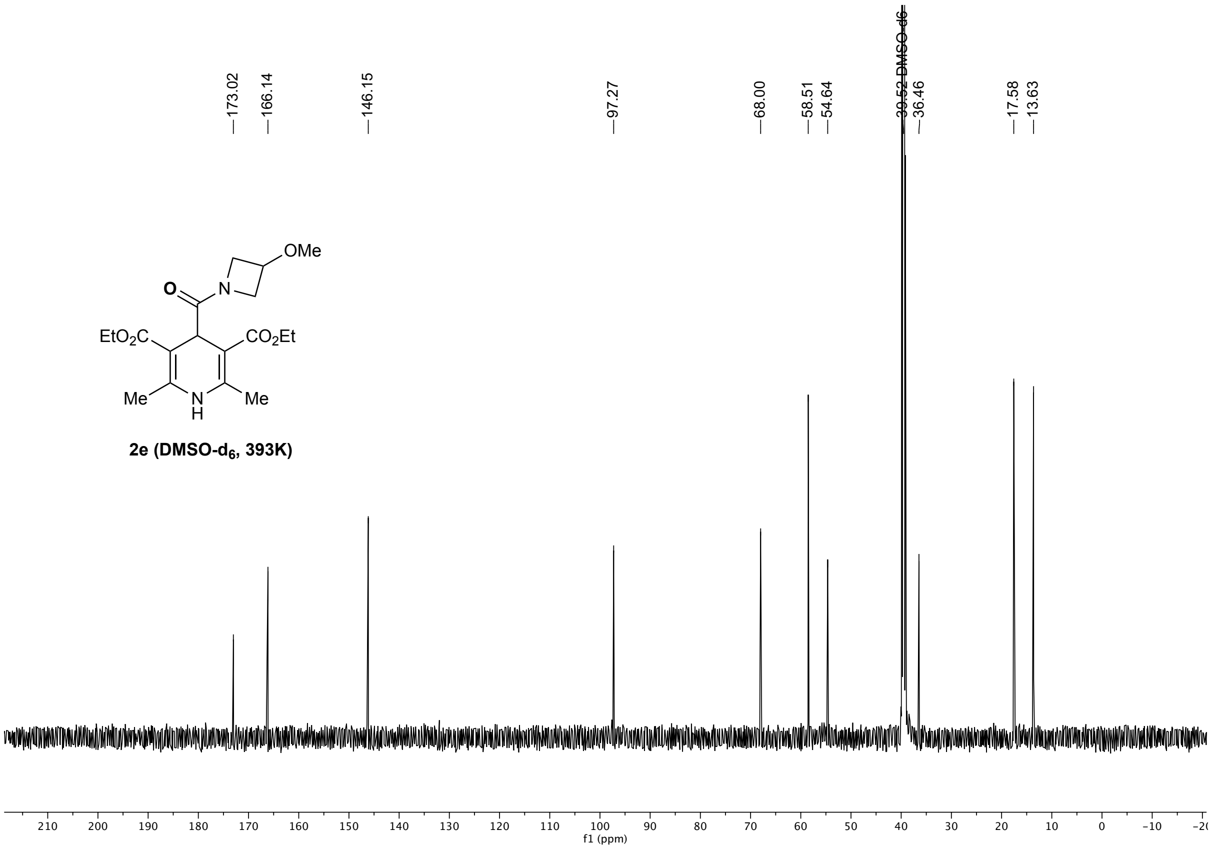
173.02
166.14
146.15
97.27
68.00
58.51
54.64
39.52 DMSO-d6
36.46
17.58
13.63
OMe
O
N
EtO2C
CO2Et
Me
N
H
Me
2e (DMSO-d6, 393K)
f1 (ppm)

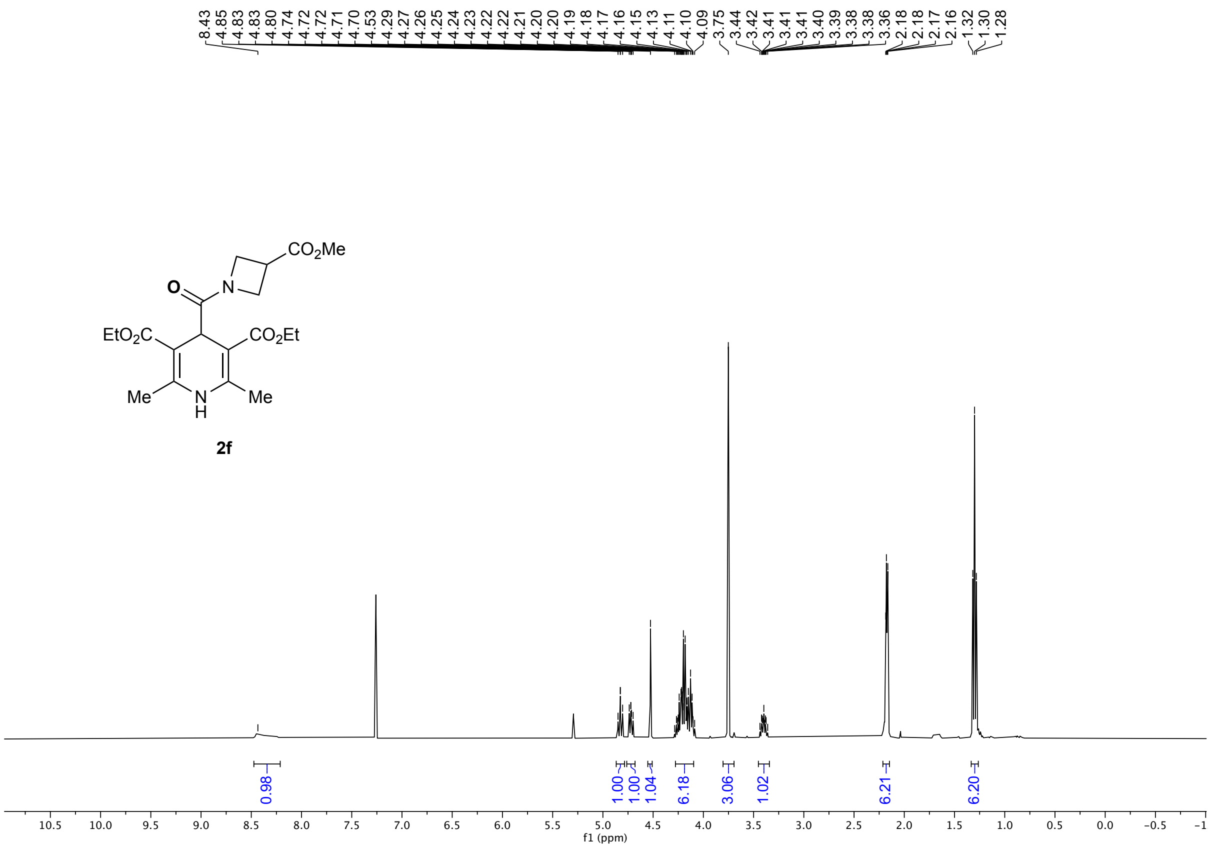
8.43
4.85
4.83
4.83
4.80
4.74
4.72
4.72
4.71
4.70
4.53
4.29
4.27
4.26
4.25
4.24
4.23
4.22
4.22
4.21
4.20
4.20
4.19
4.18
4.17
4.16
4.15
4.13
4.11
4.10
4.09
3.75
3.44
3.42
3.41
3.41
3.41
3.40
3.39
3.38
3.38
3.36
2.18
2.18
2.17
2.16
1.32
1.30
1.28
CO2Me
O
N
EtO2C
CO2Et
Me
N
H
Me
2f
0.98
1.00
1.00
1.04
6.18
3.06
1.02
6.21
6.20
f1 (ppm)

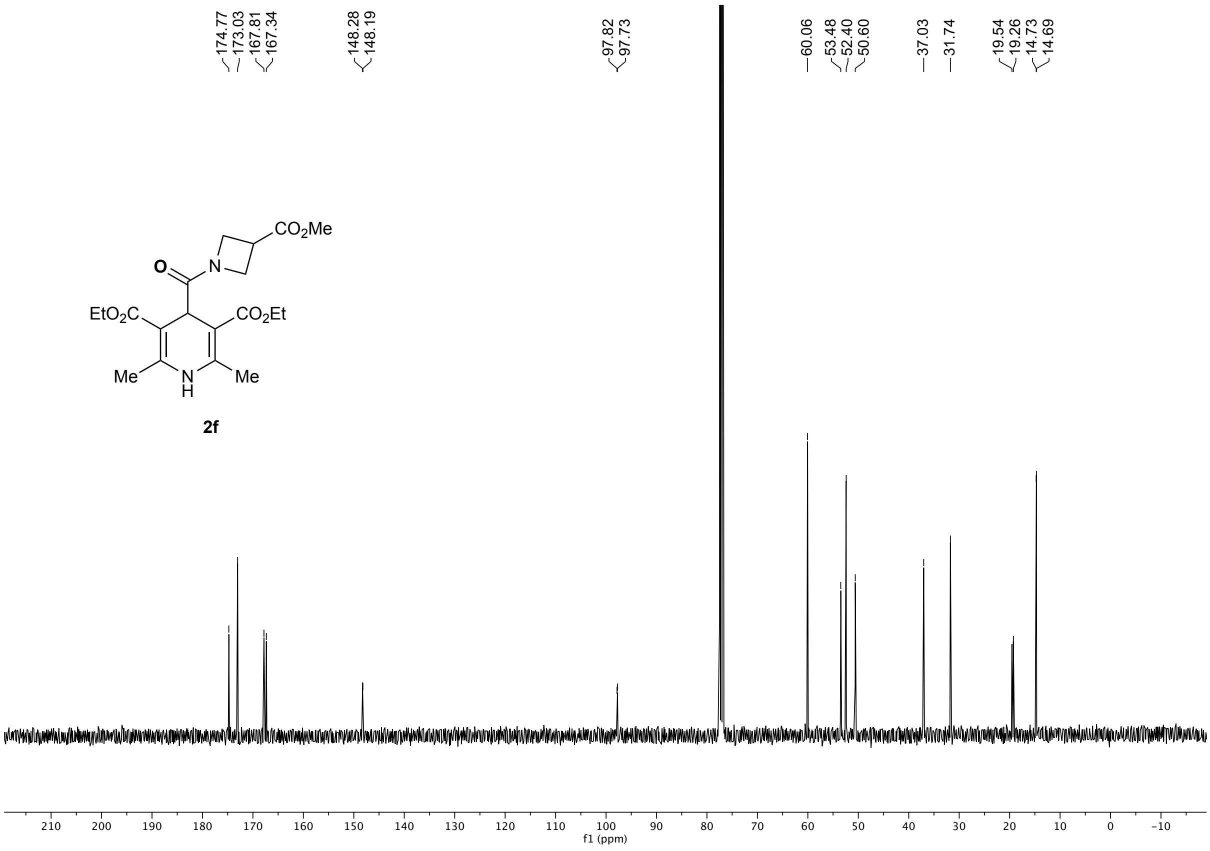
174.77
173.03
167.81
167.34
148.28
148.19
97.82
97.73
60.06
53.48
52.40
50.60
37.03
31.74
19.54
19.26
14.73
14.69
CO2Me
O
N
EtO2C
CO2Et
Me
N
H
Me
2f
f1 (ppm)

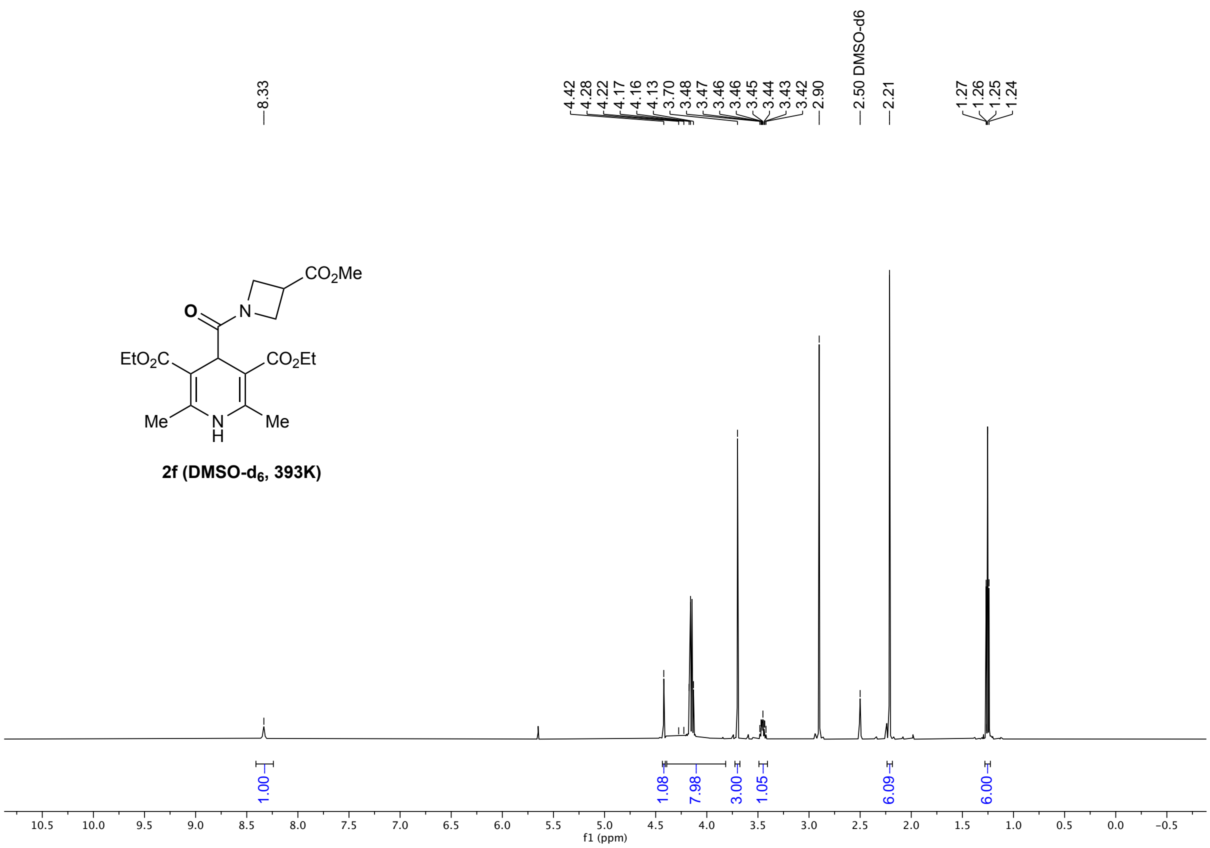
8.33
4.42
4.28
4.22
4.17
4.16
4.13
3.70
3.48
3.47
3.46
3.46
3.45
3.44
3.43
3.42
2.90
2.50 DMSO-d6
2.21
1.27
1.26
1.25
1.24
CO2Me
O
N
EtO2C
CO2Et
Me
N
H
Me
2f (DMSO-d6, 393K)
1.00
1.08
7.98
3.00
1.05
6.09
6.00
f1 (ppm)

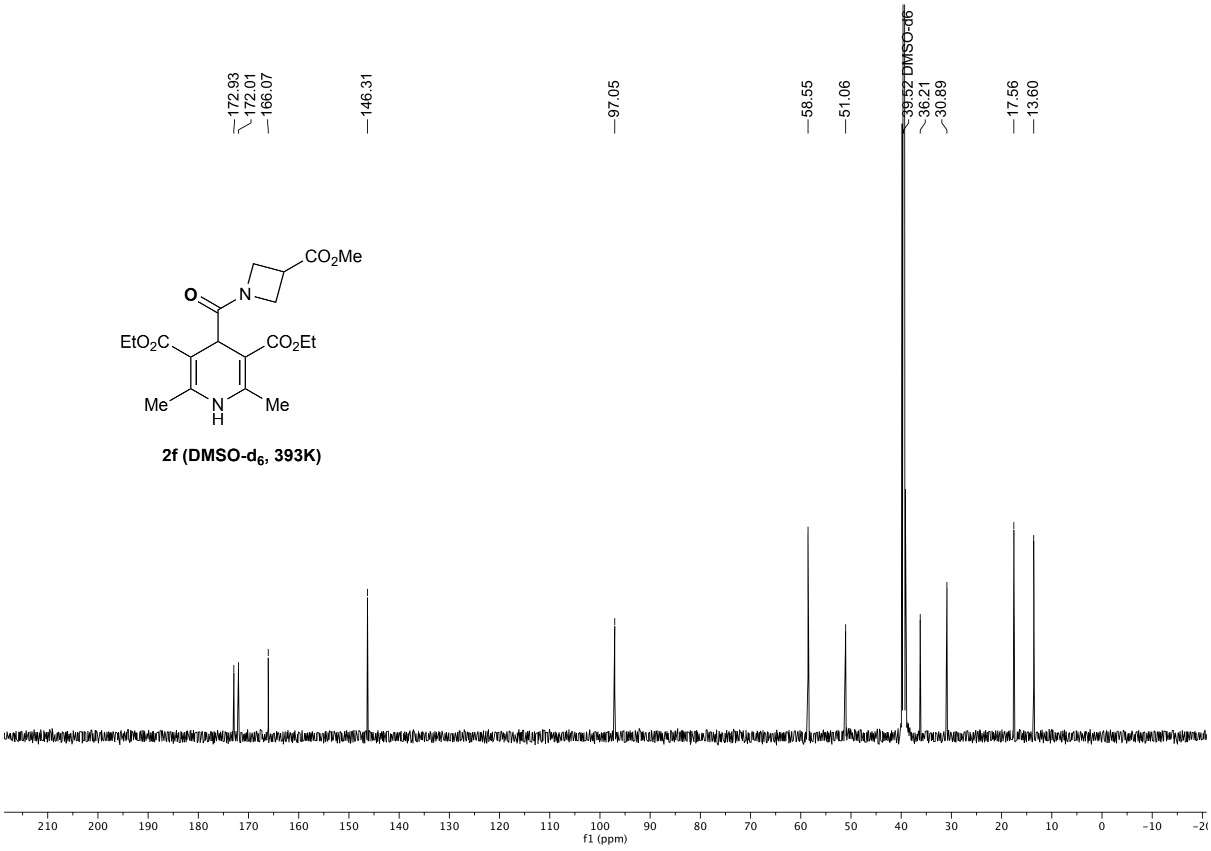
172.93
172.01
166.07
146.31
97.05
58.55
51.06
39.52 DMSO-d6
36.21
30.89
17.56
13.60
CO2Me
O
N
EtO2C
CO2Et
Me
N
H
Me
2f (DMSO-d6, 393K)
f1 (ppm)

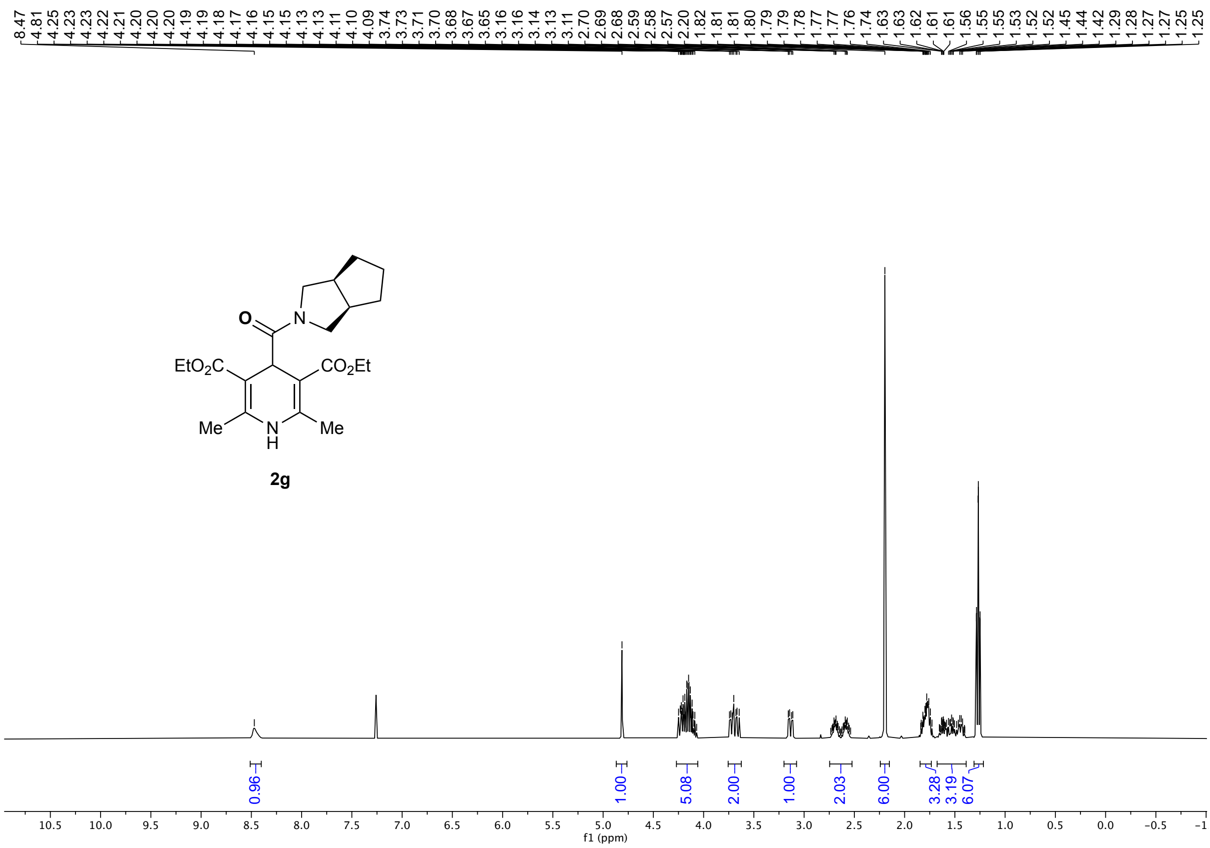
8.47 4.81 4.25 4.23 4.23 4.22 4.21 4.20 4.20 4.19 4.19 4.18 4.17 4.16 4.15 4.15 4.13 4.13 4.11 4.10 4.09 3.74 3.73 3.71 3.70 3.68 3.67 3.65 3.16 3.16 3.14 3.13 3.11 2.70 2.69 2.68 2.59 2.58 2.57 2.20 1.82 1.81 1.81 1.80 1.79 1.79 1.78 1.77 1.77 1.76 1.74 1.63 1.62 1.61 1.61 1.56 1.55 1.55 1.53 1.52 1.52 1.45 1.44 1.42 1.29 1.28 1.27 1.25 1.25
O
N
EtO2C
CO2Et
Me
N
H
Me
2g
0.96
1.00
5.08
2.00
1.00
2.03
6.00
3.28
3.19
6.07
f1 (ppm)

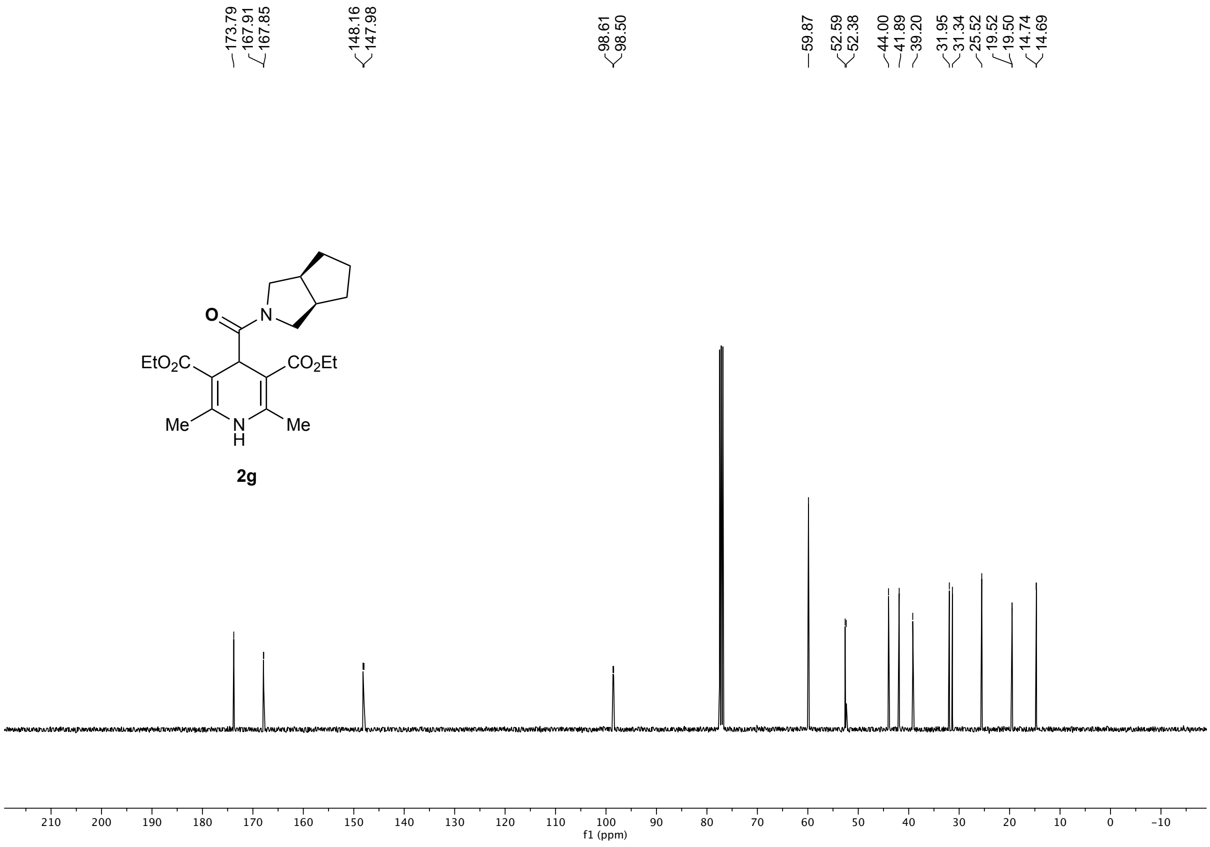
173.79 167.91 167.85
148.16 147.98
98.61 98.50
59.87 52.59 52.38 44.00 41.89 39.20 31.95 31.34 25.52 19.52 19.50 14.74 14.69
O
N
EtO2C
CO2Et
Me
N
H
Me
2g
f1 (ppm)

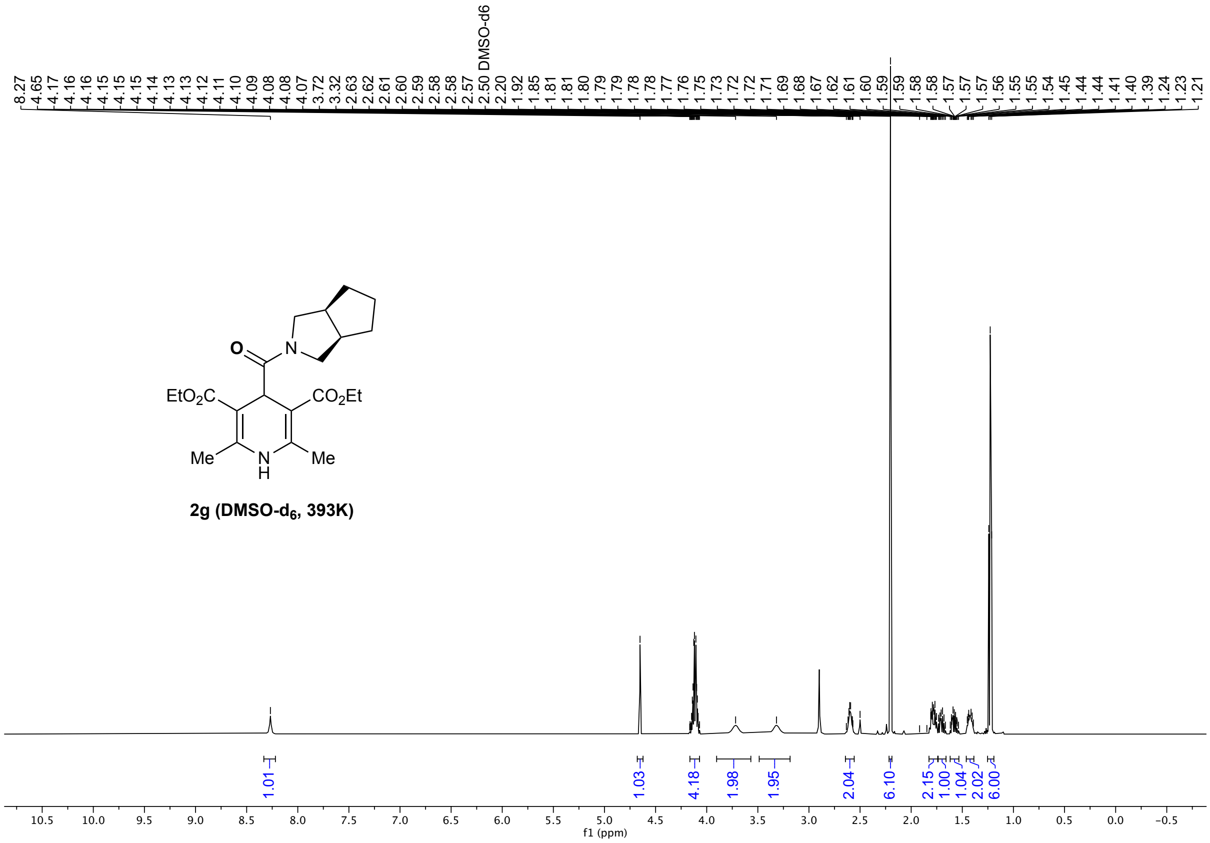
8.27 4.65 4.17 4.16 4.15 4.15 4.14 4.13 4.13 4.12 4.11 4.10 4.09 4.08 4.08 4.07 3.72 3.32 2.63 2.62 2.61 2.60 2.59 2.58 2.57 2.50 DMSO-d6 2.20 1.92 1.85 1.81 1.81 1.80 1.79 1.79 1.78 1.78 1.77 1.76 1.75 1.73 1.72 1.72 1.71 1.69 1.68 1.67 1.62 1.61 1.60 1.59 1.59 1.58 1.58 1.57 1.57 1.57 1.56 1.55 1.55 1.54 1.45 1.44 1.44 1.41 1.40 1.39 1.24 1.23 1.21
O
N
EtO2C
CO2Et
Me
N
H
Me
2g (DMSO-d6, 393K)
1.01 1.03 4.18 1.98 1.95 2.04 6.10 2.15 1.00 1.04 2.02 6.00
f1 (ppm)

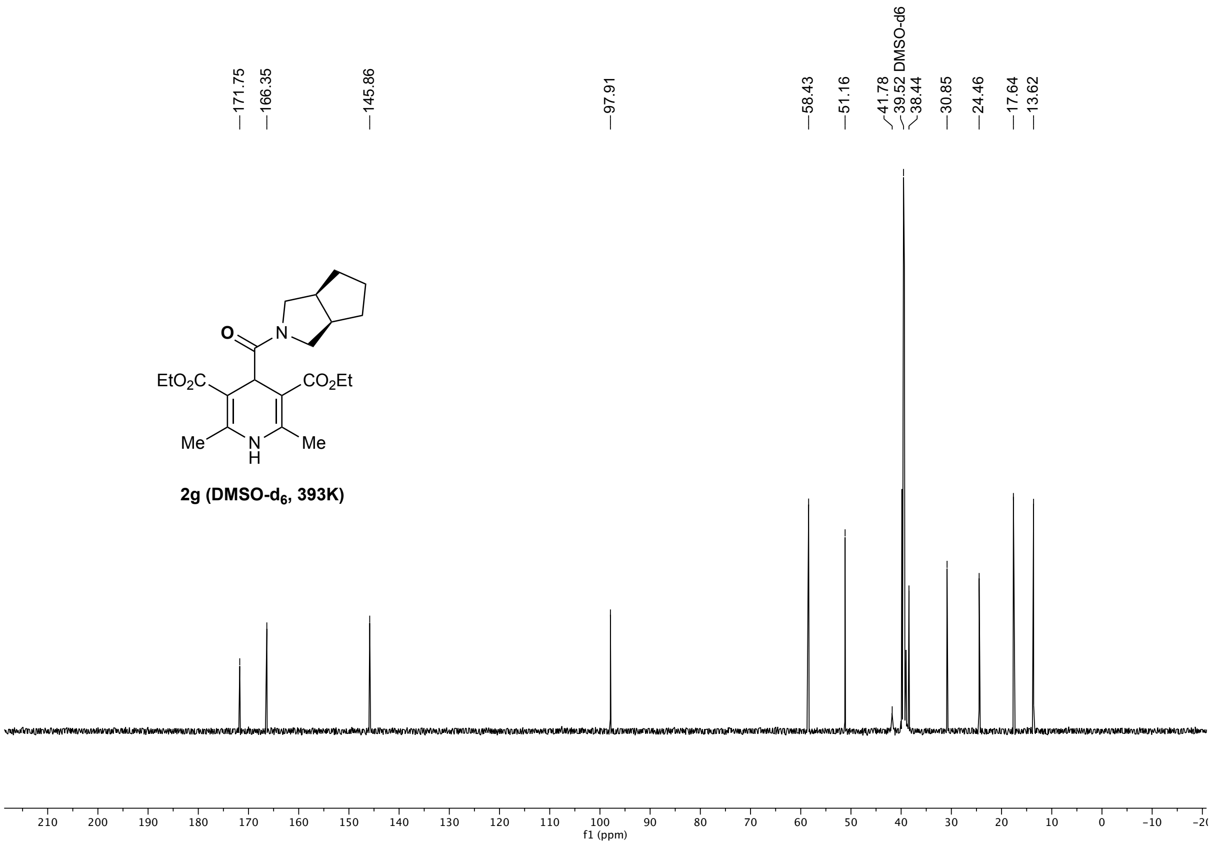
171.75 166.35 145.86 97.91 58.43 51.16 41.78 39.52 DMSO-d6 38.44 30.85 24.46 17.64 13.62
O
N
EtO2C
CO2Et
Me
N
H
Me
2g (DMSO-d6, 393K)
f1 (ppm)

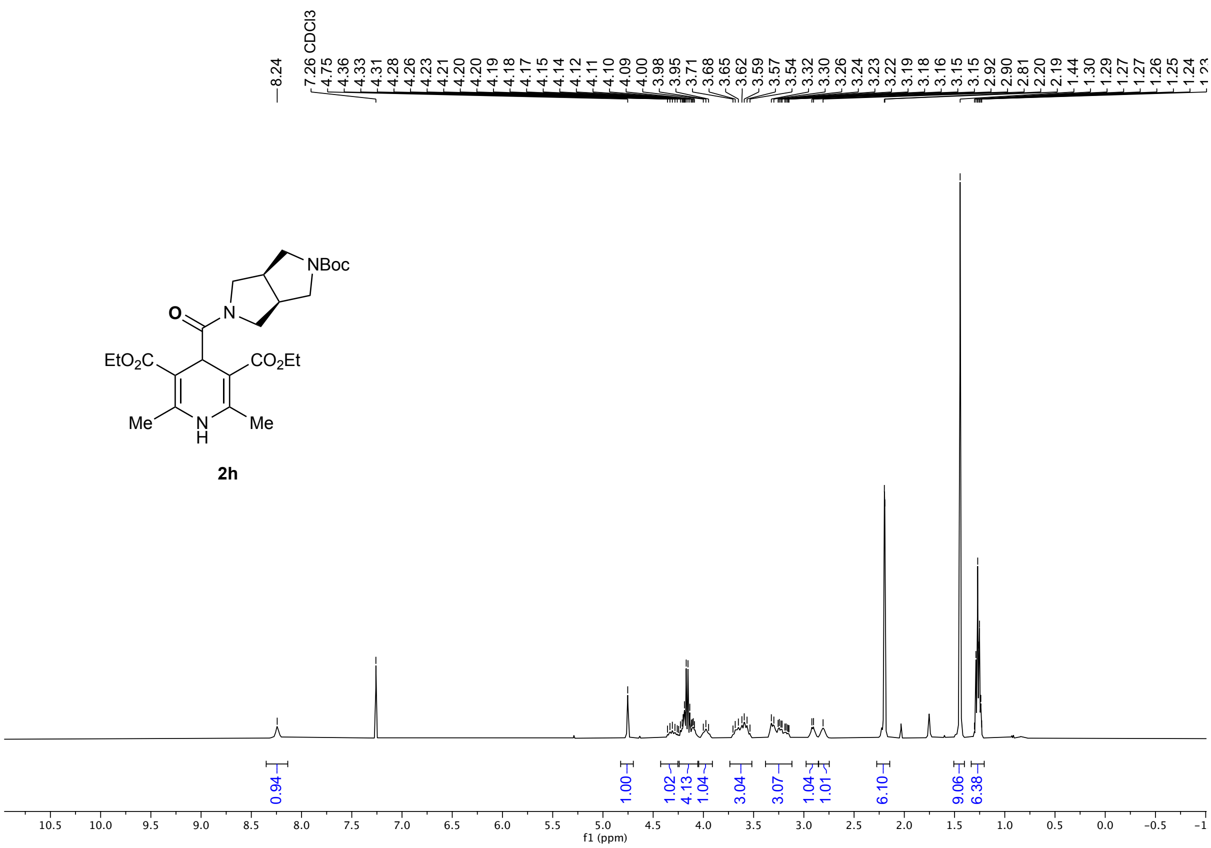
8.24
7.26 CDCl3
4.75
4.36
4.33
4.31
4.28
4.26
4.23
4.21
4.20
4.20
4.19
4.18
4.17
4.15
4.14
4.12
4.11
4.10
4.09
4.00
3.98
3.95
3.71
3.68
3.65
3.62
3.59
3.57
3.54
3.32
3.30
3.26
3.24
3.23
3.22
3.19
3.18
3.16
3.15
3.15
2.92
2.90
2.81
2.20
2.19
1.44
1.30
1.29
1.27
1.27
1.26
1.25
1.24
NBoc
O
N
EtO2C
CO2Et
Me
N
H
Me
2h
0.94
1.00
1.02
4.13
1.04
3.04
3.07
1.04
1.01
6.10
9.06
6.38
f1 (ppm)

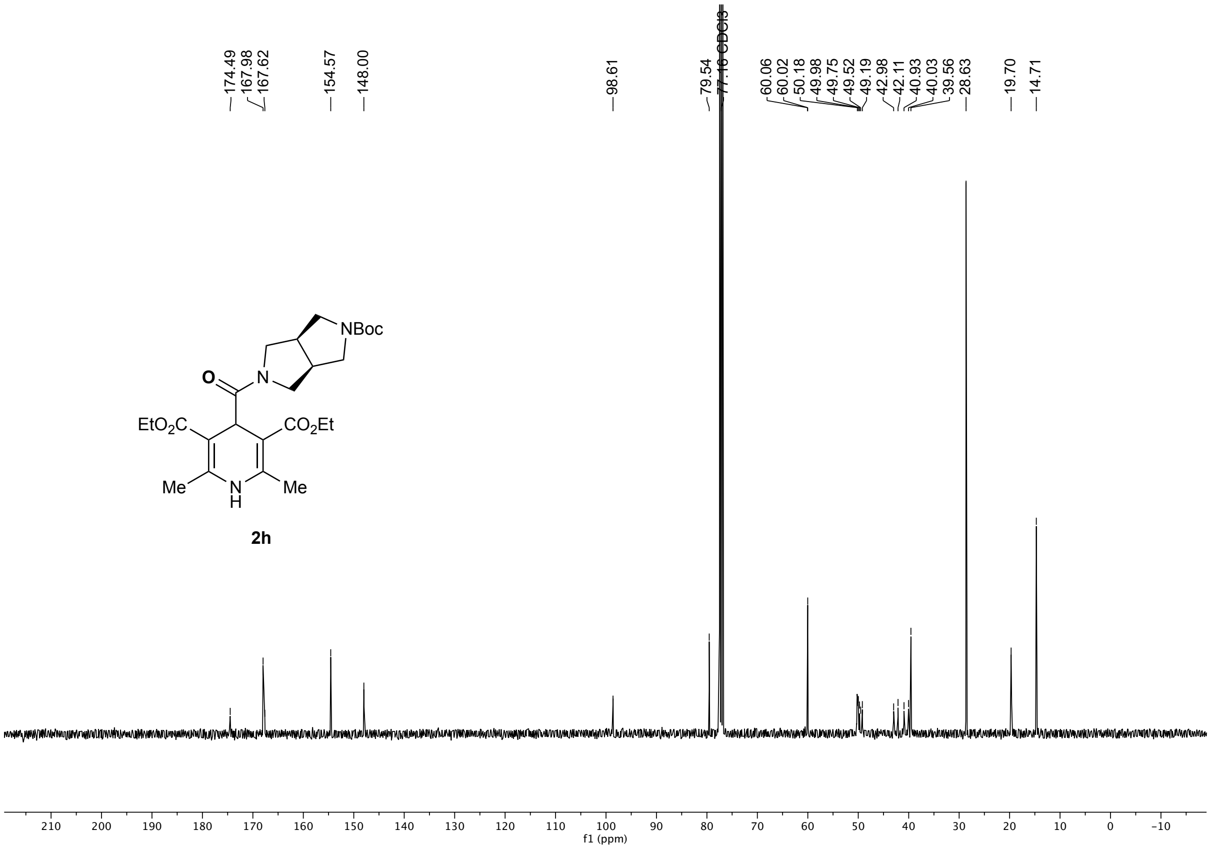
174.49
167.98
167.62
154.57
148.00
98.61
79.54
77.16 CDCl3
60.06
60.02
50.18
49.98
49.75
49.52
49.19
42.98
42.11
40.93
40.03
39.56
28.63
19.70
14.71
NBoc
O
N
EtO2C
CO2Et
Me
N
H
Me
2h
f1 (ppm)

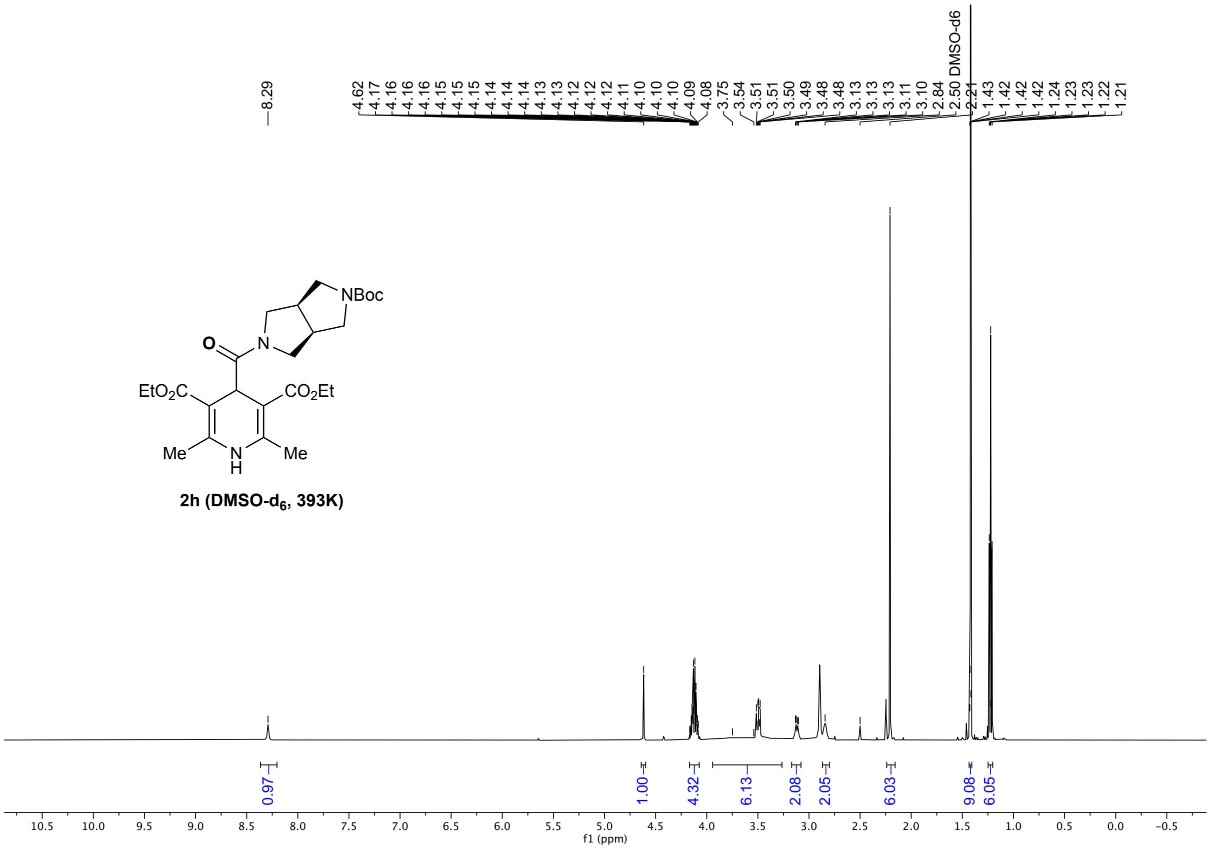


2h (DMSO-$d_6$, 393K)

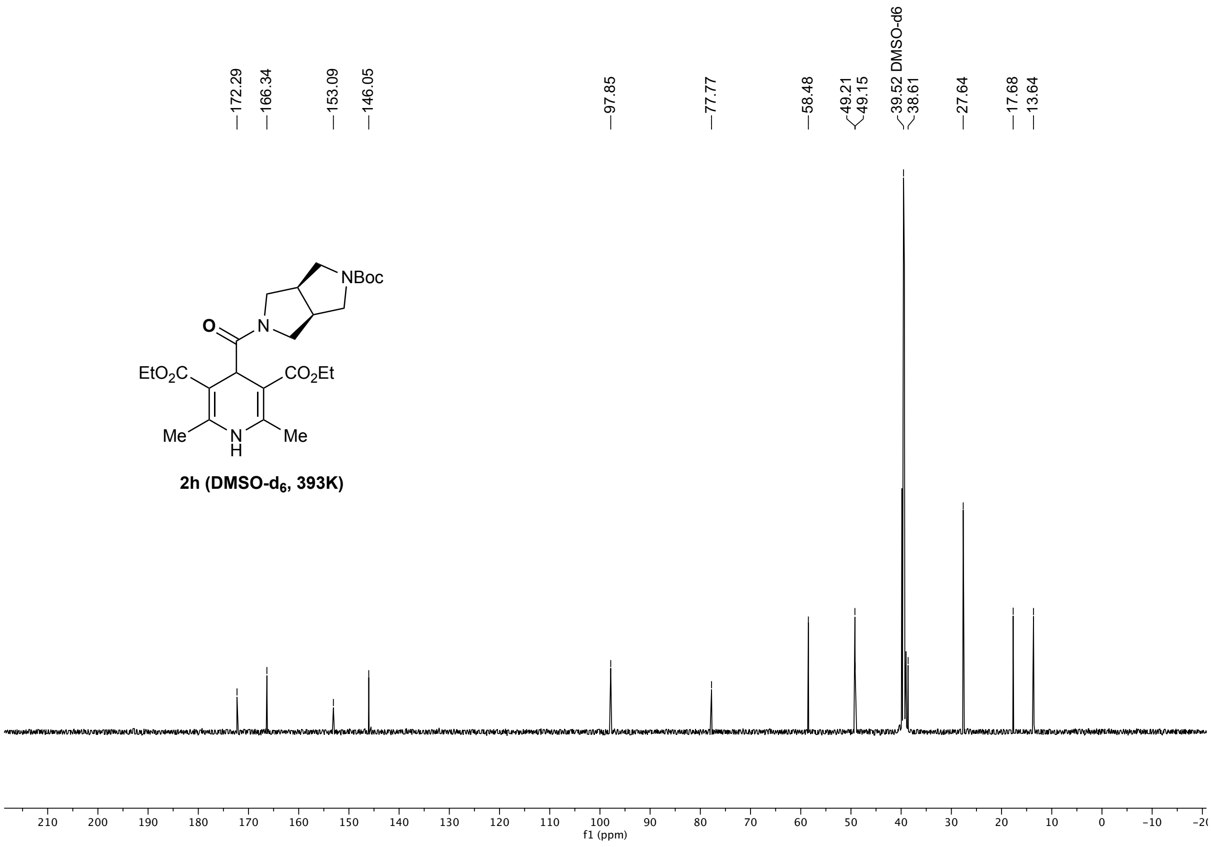


2h (DMSO-$d_6$, 393K)

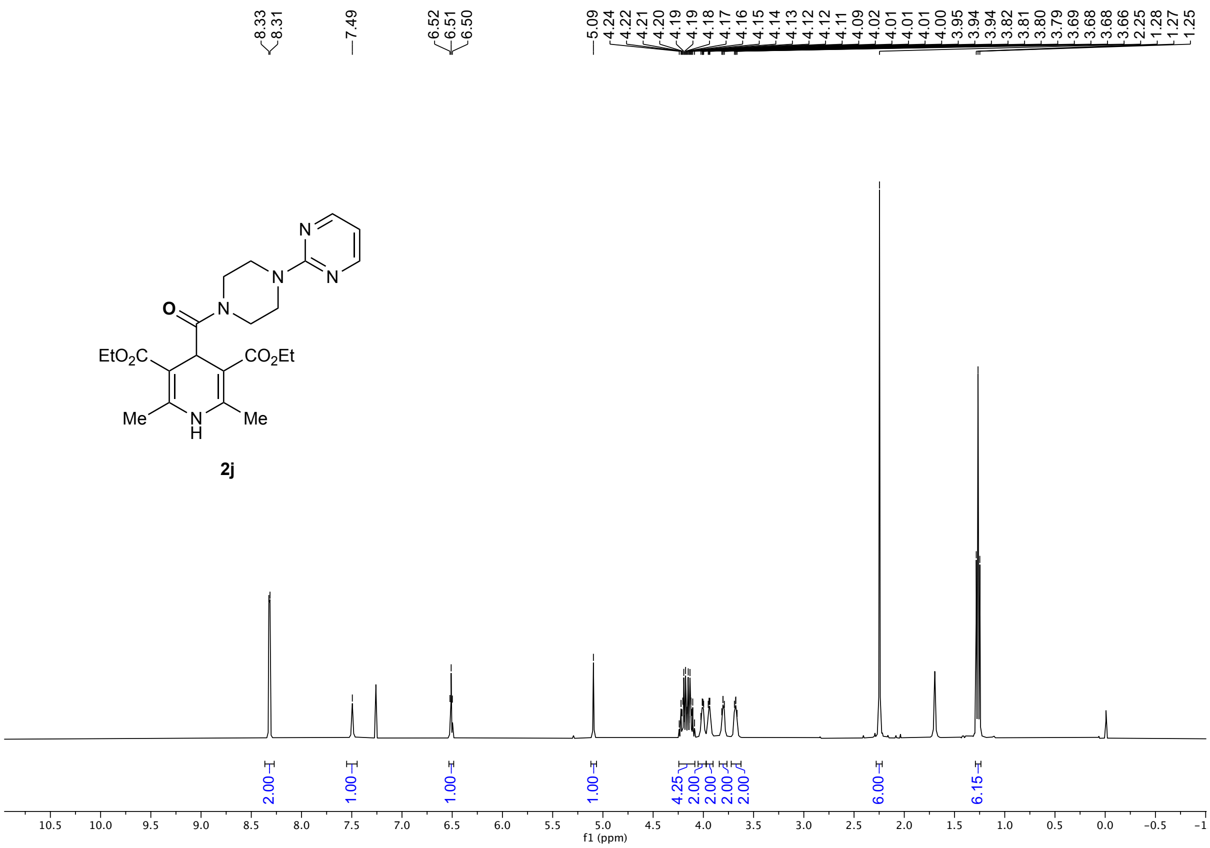
8.33
8.31
7.49
6.52
6.51
6.50
5.09
4.24
4.22
4.21
4.20
4.19
4.19
4.18
4.17
4.16
4.15
4.14
4.13
4.12
4.12
4.11
4.09
4.02
4.01
4.01
4.01
4.00
3.95
3.94
3.94
3.82
3.81
3.80
3.79
3.69
3.68
3.68
3.66
2.25
1.28
1.27
1.25
O
N
N
N
N
EtO2C
CO2Et
Me
N
H
Me
2j
2.00
1.00
1.00
1.00
4.25
2.00
2.00
2.00
2.00
6.00
6.15
10.5 10.0 9.5 9.0 8.5 8.0 7.5 7.0 6.5 6.0 5.5 5.0 4.5 4.0 3.5 3.0 2.5 2.0 1.5 1.0 0.5 0.0 −0.5 −1
f1 (ppm)

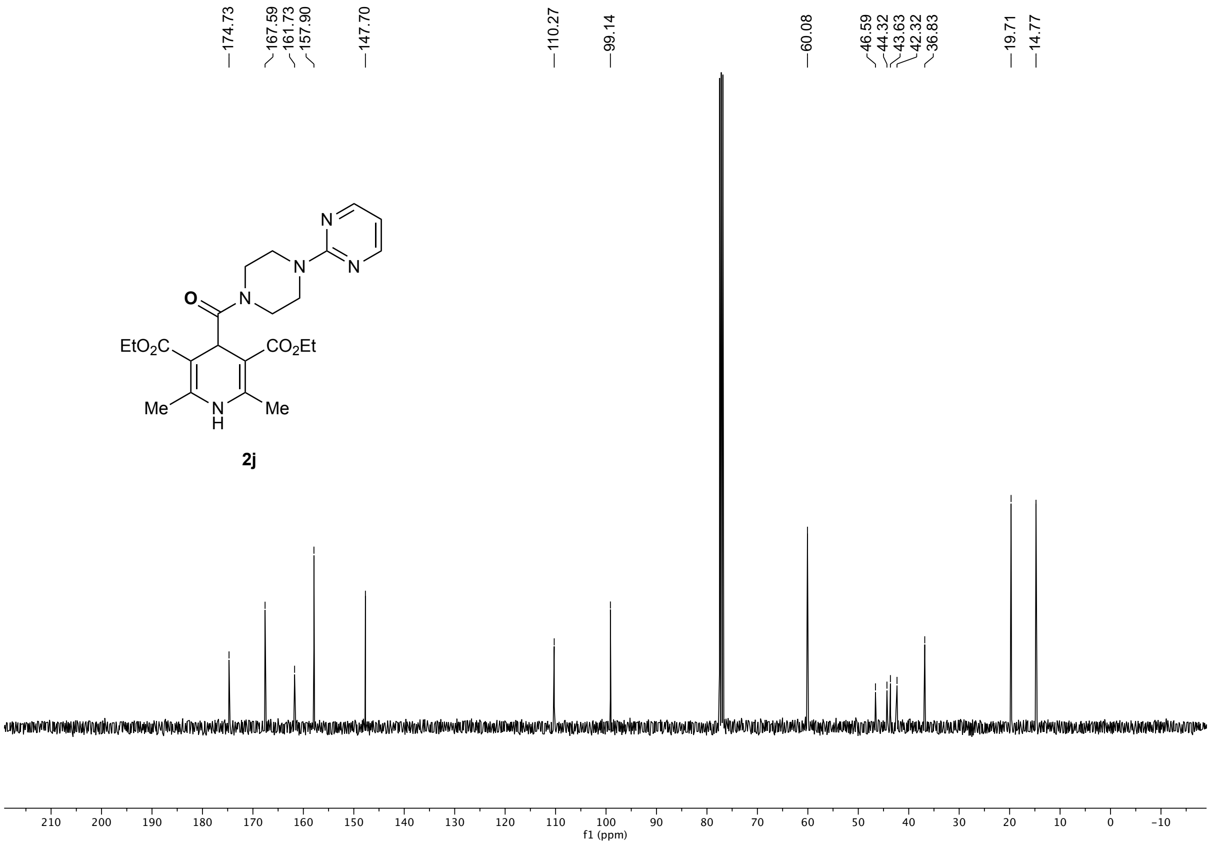
174.73
167.59
161.73
157.90
147.70
110.27
99.14
60.08
46.59
44.32
43.63
42.32
36.83
19.71
14.77
O
N
N
N
N
EtO2C
CO2Et
Me
N
H
Me
2j
210 200 190 180 170 160 150 140 130 120 110 100 90 80 70 60 50 40 30 20 10 0 −10
f1 (ppm)

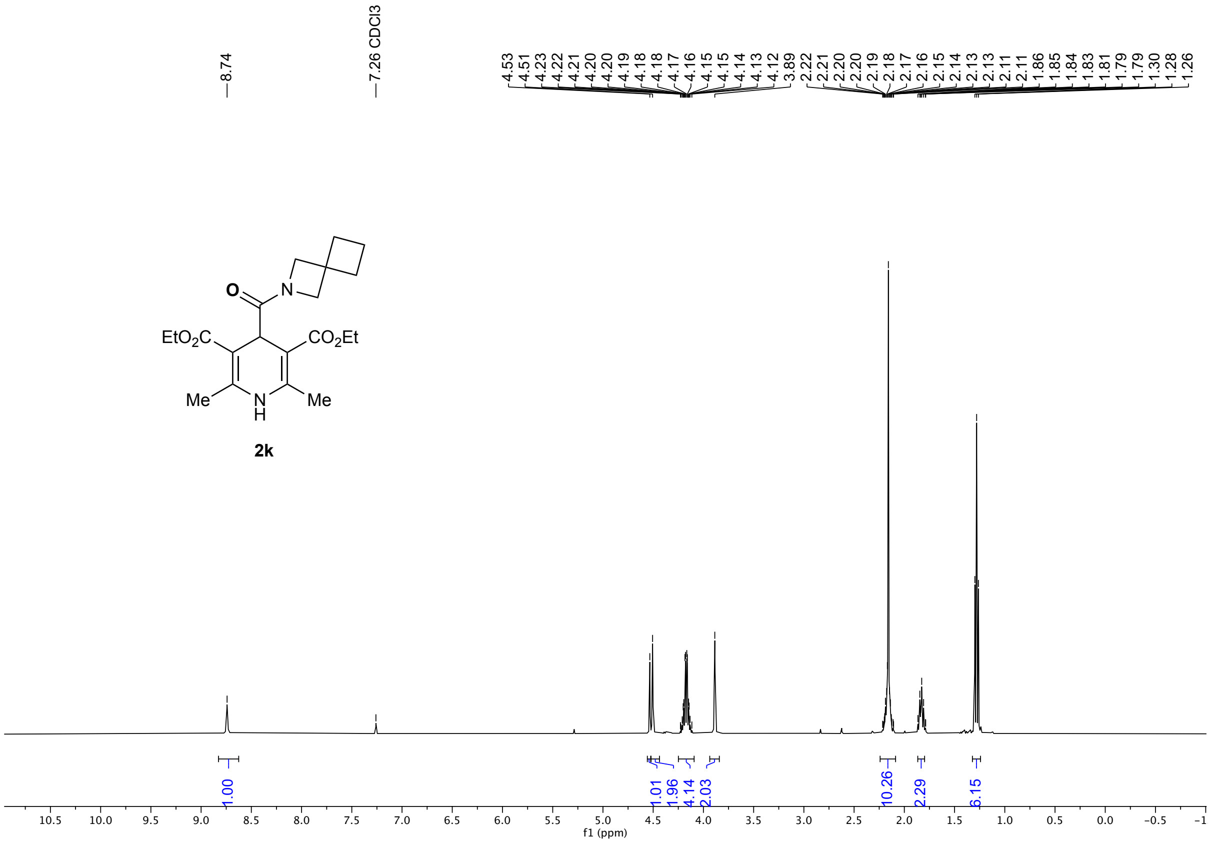
8.74
7.26 CDCl3
4.53 4.51 4.23 4.22 4.21 4.20 4.20 4.19 4.18 4.18 4.17 4.16 4.15 4.15 4.14 4.13 4.12 3.89 2.22 2.21 2.20 2.20 2.19 2.18 2.17 2.16 2.15 2.14 2.13 2.13 2.11 2.11 1.86 1.85 1.84 1.83 1.81 1.79 1.30 1.28 1.26
O
N
$EtO_2C$
$CO_2Et$
Me
N
H
Me
2k
1.00
1.01
1.96
4.14
2.03
10.26
2.29
6.15
f1 (ppm)

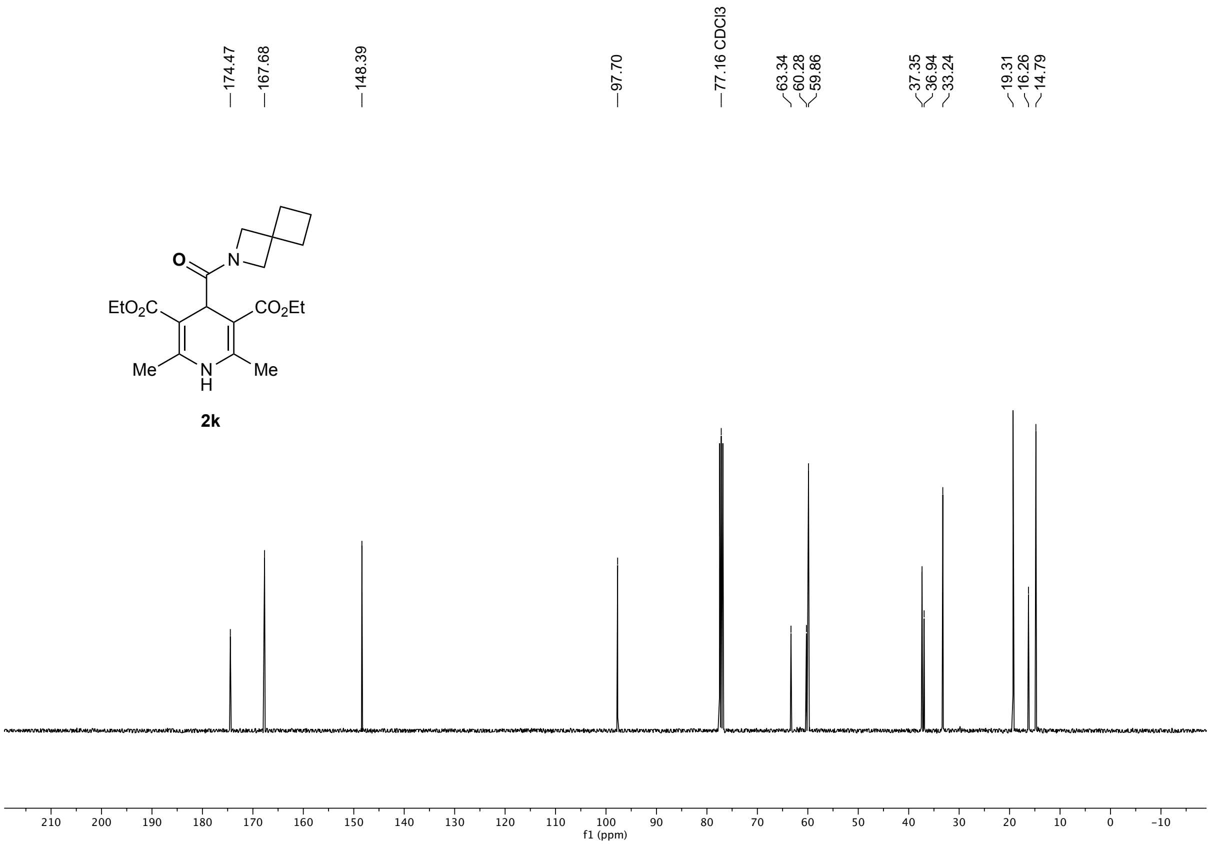
174.47
167.68
148.39
97.70
77.16 CDCl3
63.34 60.28 59.86
37.35 36.94 33.24
19.31 16.26 14.79
O
N
$EtO_2C$
$CO_2Et$
Me
N
H
Me
2k
f1 (ppm)

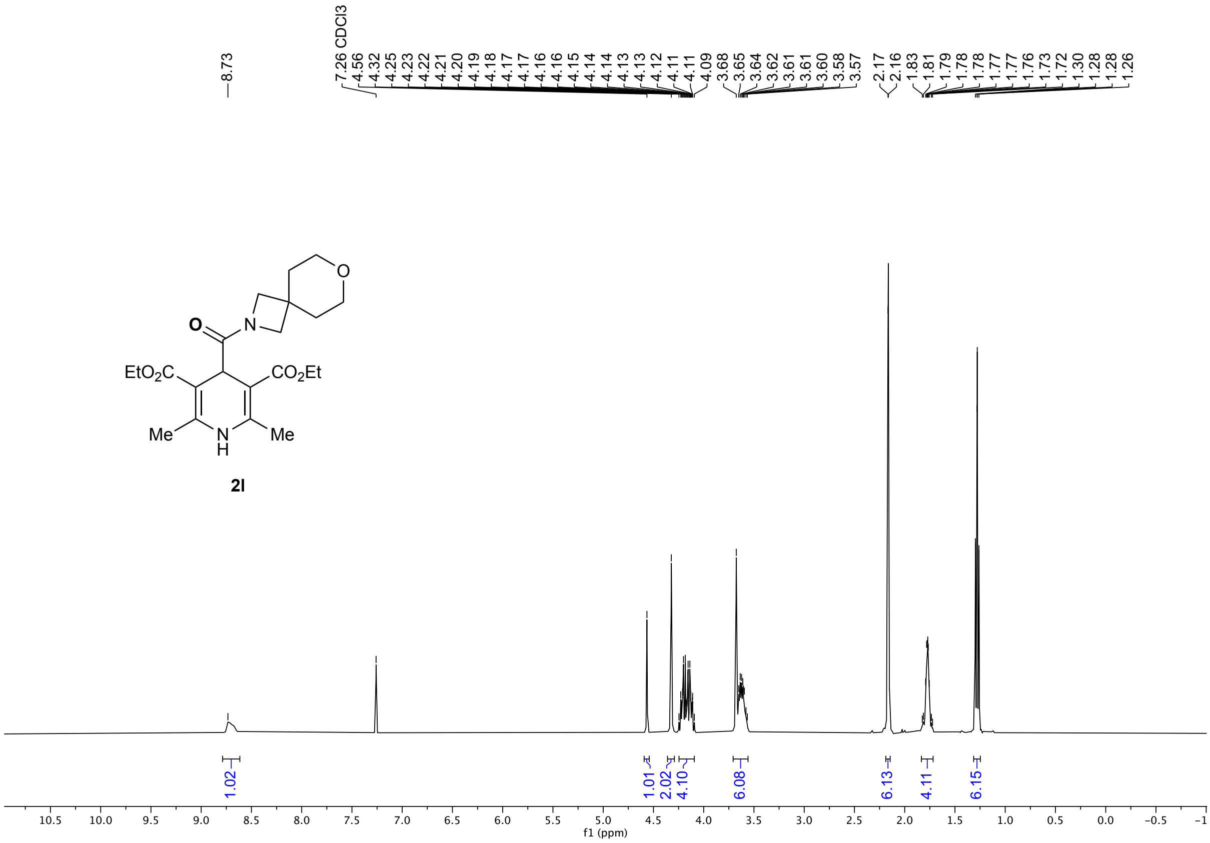

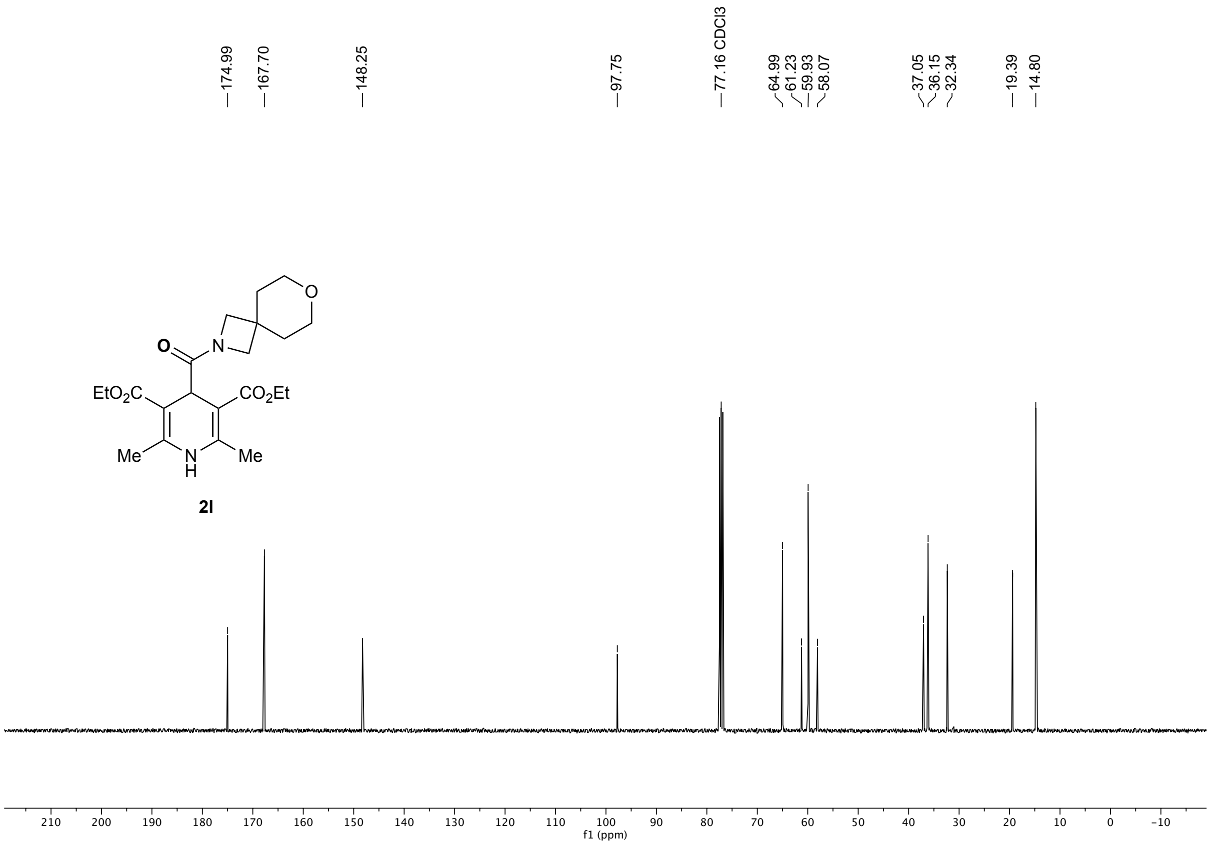

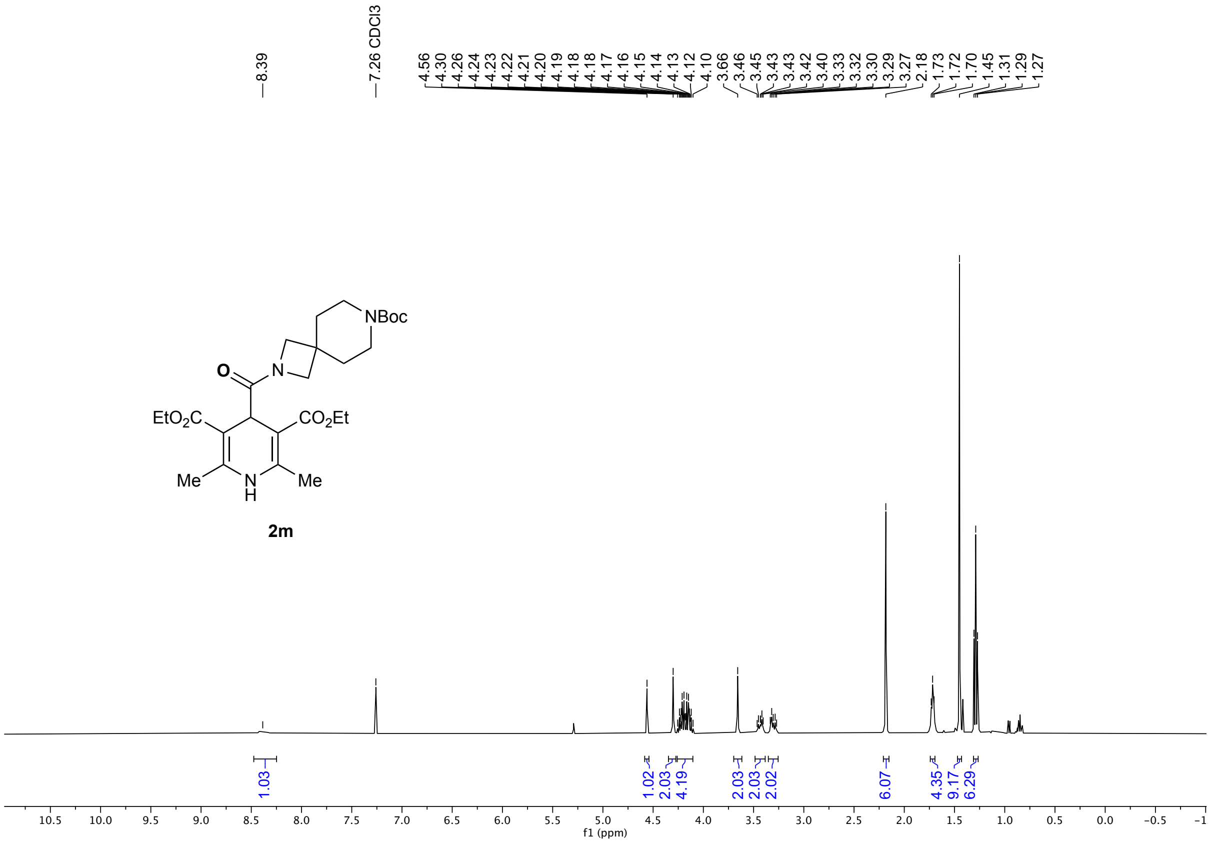
8.39
7.26 CDCl3
4.56 4.30 4.26 4.24 4.23 4.22 4.21 4.20 4.19 4.18 4.18 4.17 4.16 4.15 4.14 4.13 4.12 4.10
3.66 3.46 3.45 3.43 3.43 3.42 3.40 3.33 3.32 3.30 3.29 3.27
2.18 1.73 1.72 1.70 1.45 1.31 1.29 1.27
NBoc
O
N
$EtO_2C$
$CO_2Et$
Me
N
H
Me
2m
1.03
1.02 2.03 4.19
2.03 2.03 2.02
6.07
4.35 9.17 6.29
f1 (ppm)

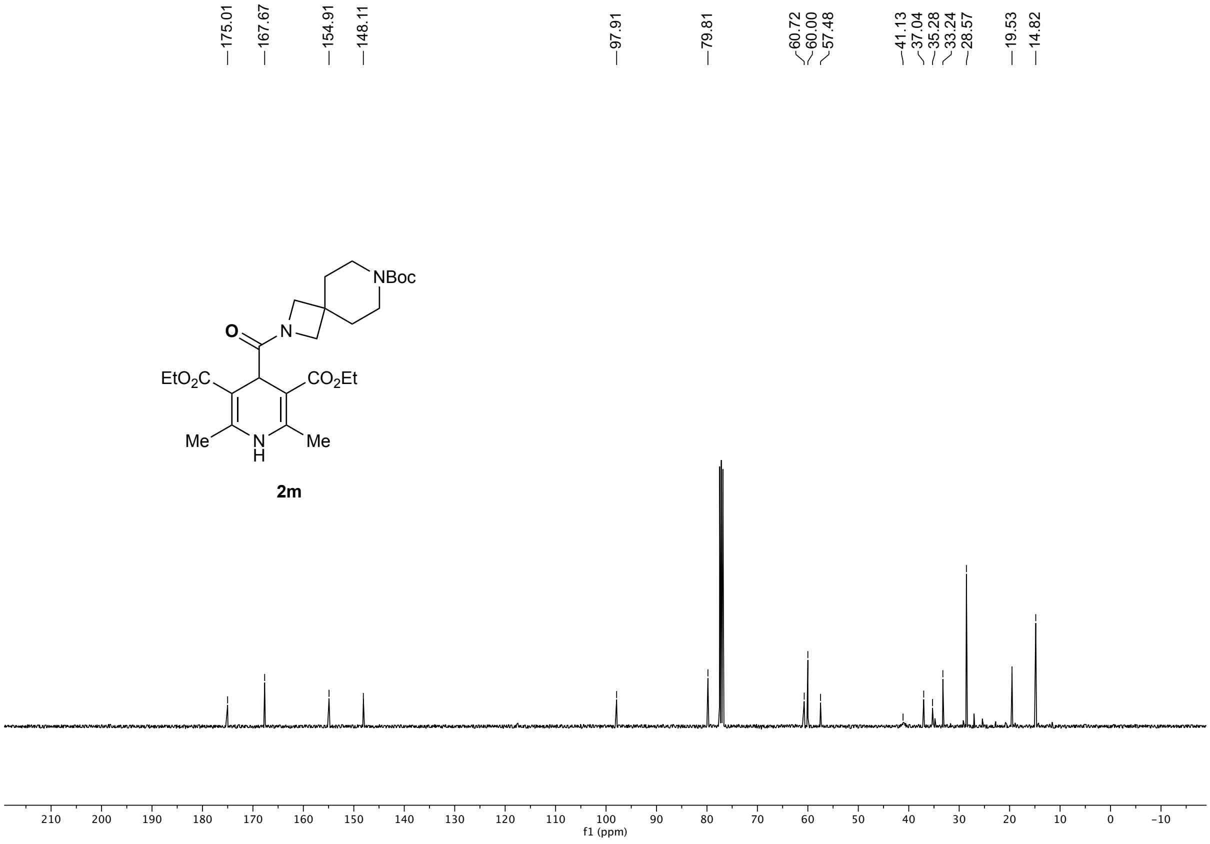
175.01 167.67 154.91 148.11
97.91
79.81
60.72 60.00 57.48
41.13 37.04 35.28 33.24 28.57
19.53 14.82
NBoc
O
N
$EtO_2C$
$CO_2Et$
Me
N
H
Me
2m
f1 (ppm)

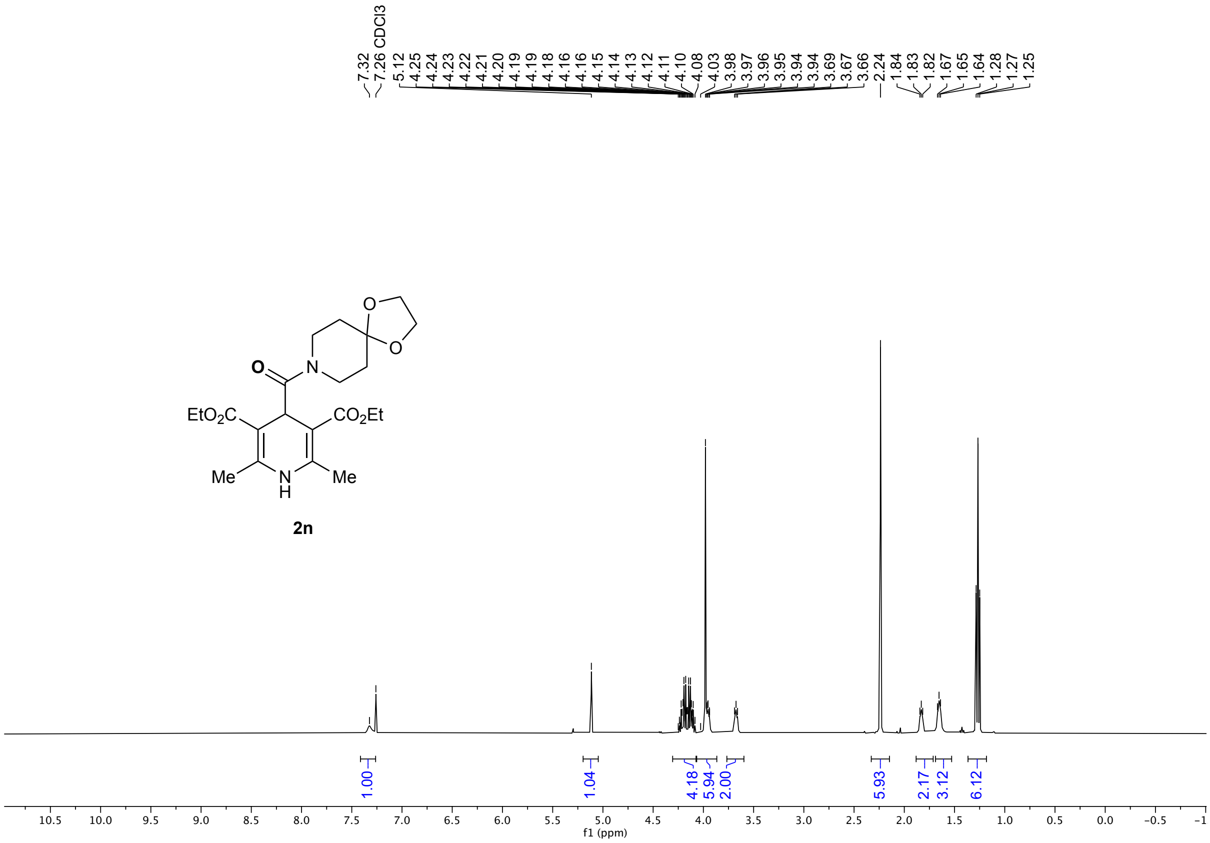
7.32
7.26 CDCl3
5.12
4.25
4.24
4.23
4.22
4.21
4.20
4.19
4.19
4.18
4.16
4.16
4.15
4.14
4.13
4.12
4.11
4.10
4.08
4.03
3.98
3.97
3.96
3.95
3.94
3.94
3.69
3.67
3.66
2.24
1.84
1.83
1.82
1.67
1.65
1.64
1.28
1.27
1.25
O
N
O
O
EtO2C
CO2Et
Me
N
H
Me
2n
1.00
1.04
4.18
5.94
2.00
5.93
2.17
3.12
6.12
f1 (ppm)

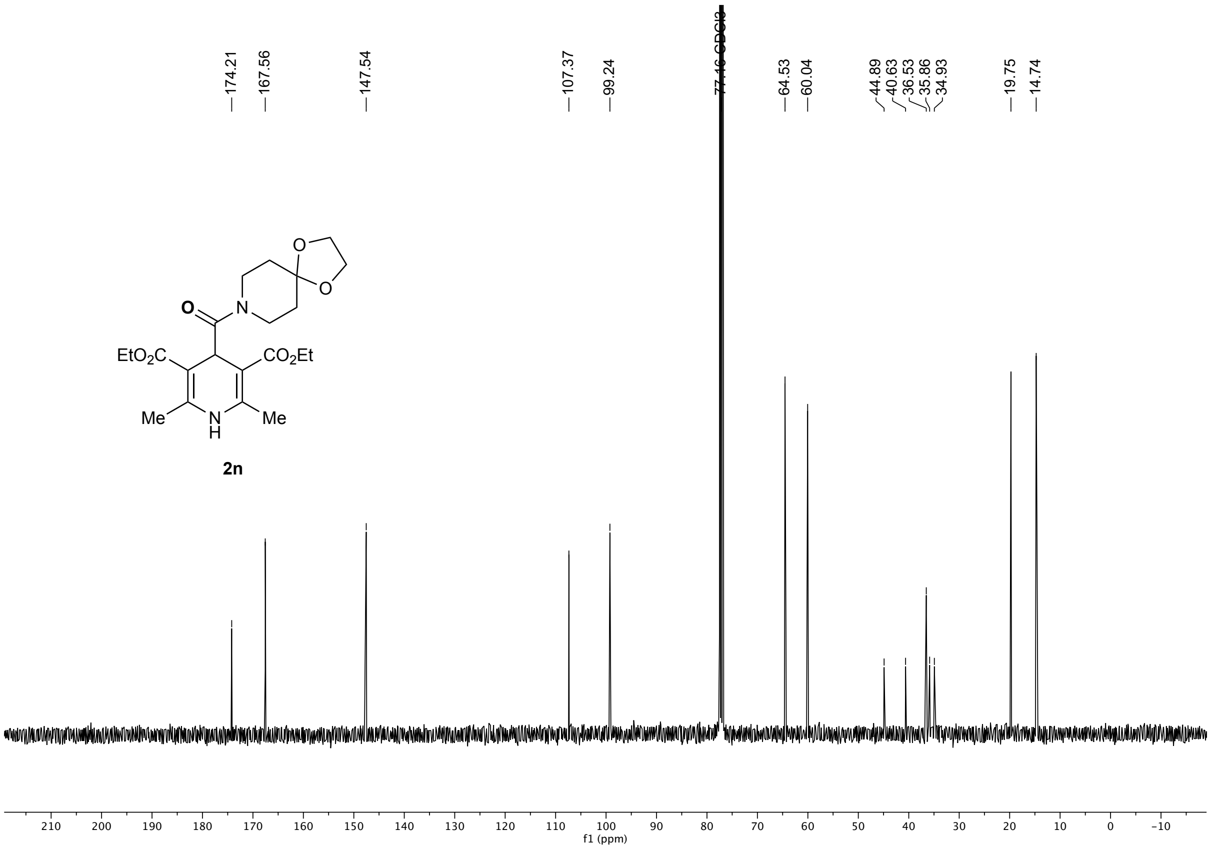
174.21
167.56
147.54
107.37
99.24
77.16 CDCl3
64.53
60.04
44.89
40.63
36.53
35.86
34.93
19.75
14.74
O
N
O
O
EtO2C
CO2Et
Me
N
H
Me
2n
f1 (ppm)

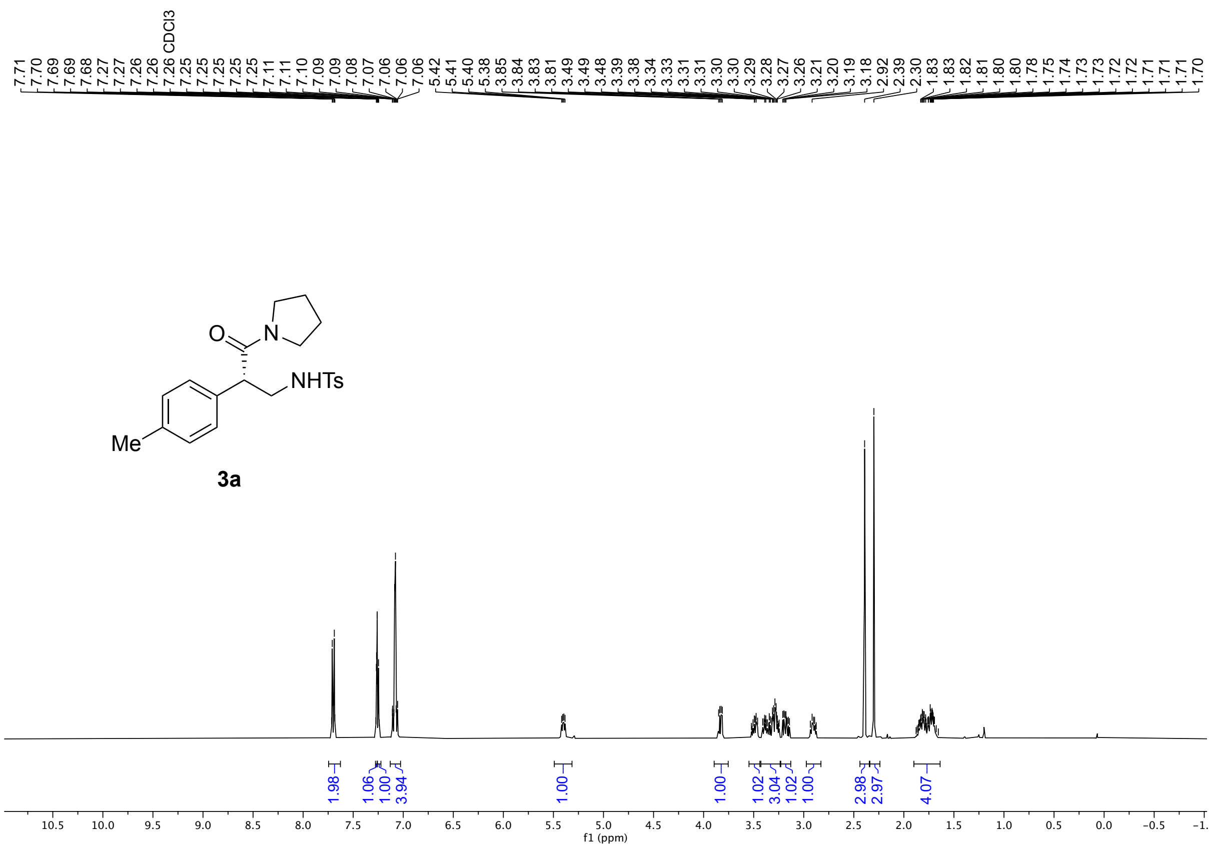
7.71
7.70
7.69
7.69
7.68
7.27
7.27
7.26
7.26 CDCl3
7.25
7.25
7.25
7.25
7.25
7.11
7.11
7.10
7.09
7.09
7.08
7.07
7.06
7.06
7.06
5.42
5.41
5.40
5.38
3.85
3.84
3.83
3.81
3.49
3.49
3.48
3.39
3.38
3.34
3.33
3.31
3.31
3.30
3.30
3.29
3.28
3.27
3.26
3.21
3.20
3.19
3.18
2.92
2.39
2.30
1.83
1.83
1.82
1.81
1.80
1.80
1.78
1.75
1.74
1.73
1.73
1.72
1.72
1.71
1.71
1.71
1.70
O
N
NHTs
Me
3a
1.98
1.06
1.00
3.94
1.00
1.00
1.02
3.04
1.02
1.00
2.98
2.97
4.07
f1 (ppm)

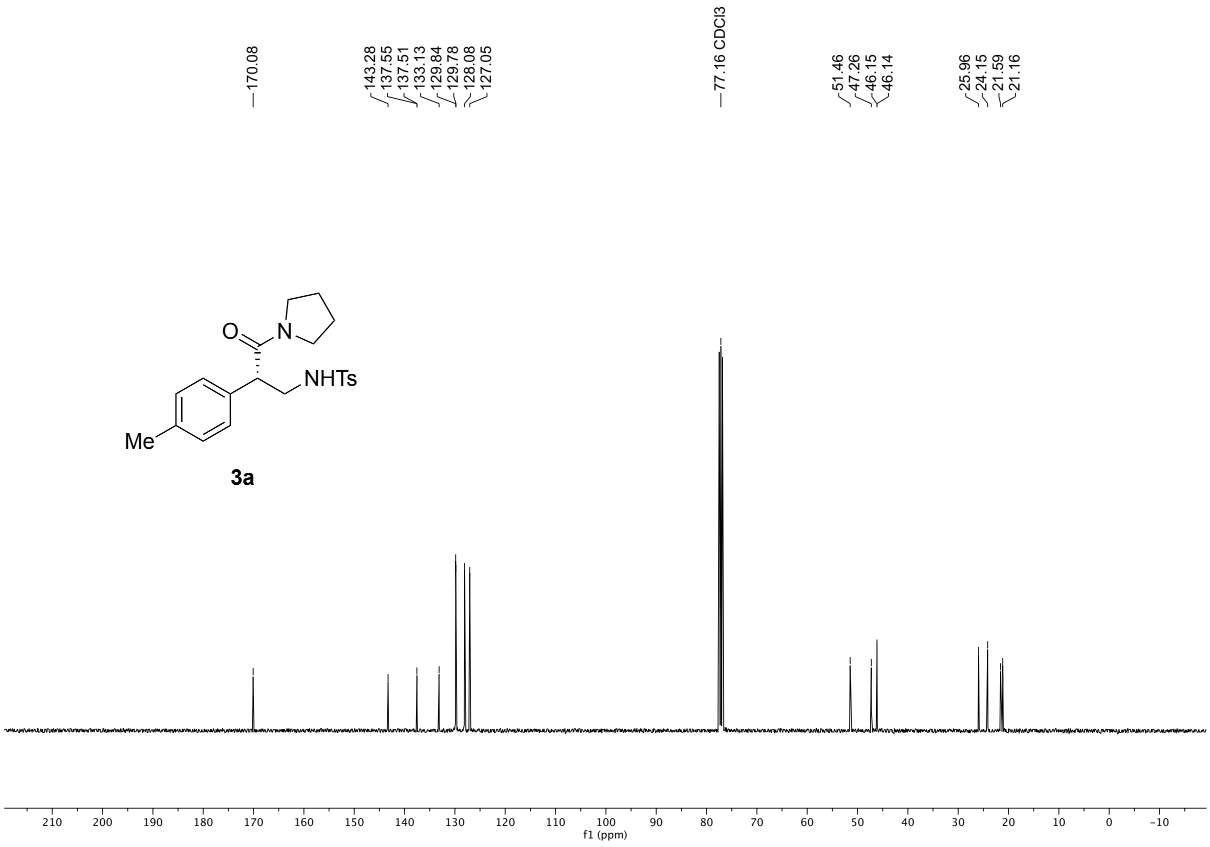
170.08
143.28
137.55
137.51
133.13
129.84
129.78
128.08
127.05
77.16 CDCl3
51.46
47.26
46.15
46.14
25.96
24.15
21.59
21.16
O
N
NHTs
Me
3a
f1 (ppm)

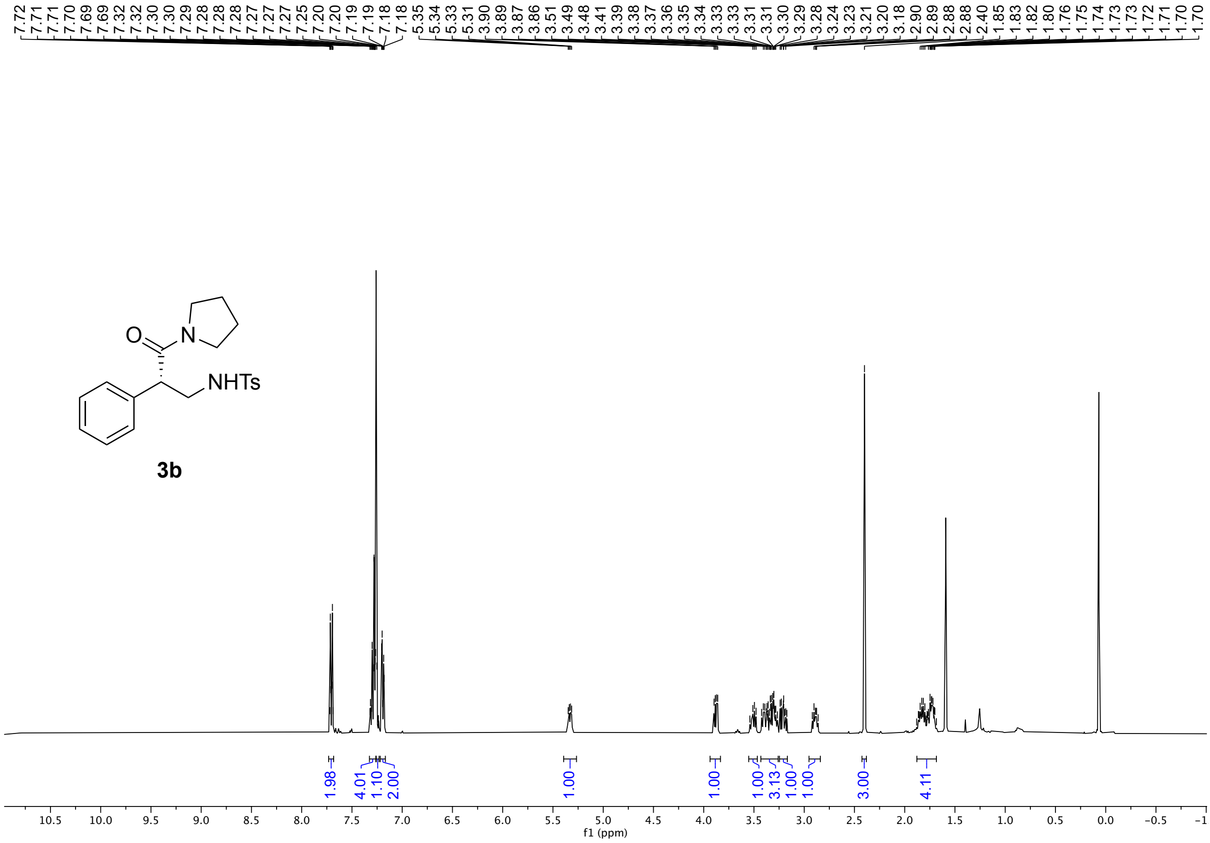
7.72 7.71 7.70 7.69 7.69 7.32 7.32 7.30 7.30 7.29 7.28 7.28 7.28 7.27 7.27 7.27 7.25 7.20 7.20 7.19 7.19 7.18 5.35 5.34 5.33 5.31 3.90 3.89 3.87 3.86 3.51 3.49 3.48 3.41 3.39 3.38 3.37 3.36 3.35 3.34 3.33 3.33 3.31 3.31 3.30 3.29 3.28 3.24 3.23 3.21 3.20 3.18 2.90 2.89 2.88 2.88 2.40 1.85 1.83 1.82 1.80 1.76 1.75 1.74 1.73 1.73 1.72 1.71 1.70
O
N
NHTs
3b
1.98
4.01
1.10
2.00
1.00
1.00
1.00
3.13
1.00
1.00
3.00
4.11
f1 (ppm)

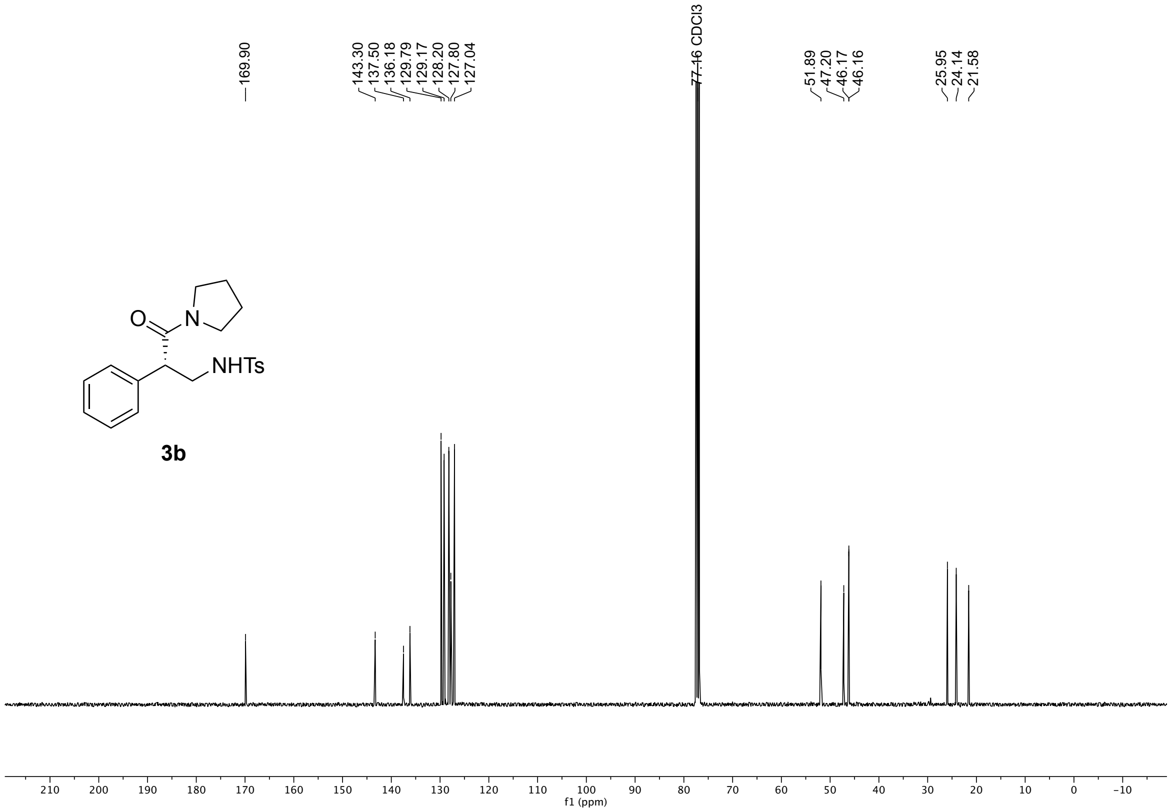
169.90
143.30 137.50 136.18 129.79 129.17 128.20 127.80 127.04
77.46 CDCl3
51.89 47.20 46.17 46.16
25.95 24.14 21.58
O
N
NHTs
3b
f1 (ppm)

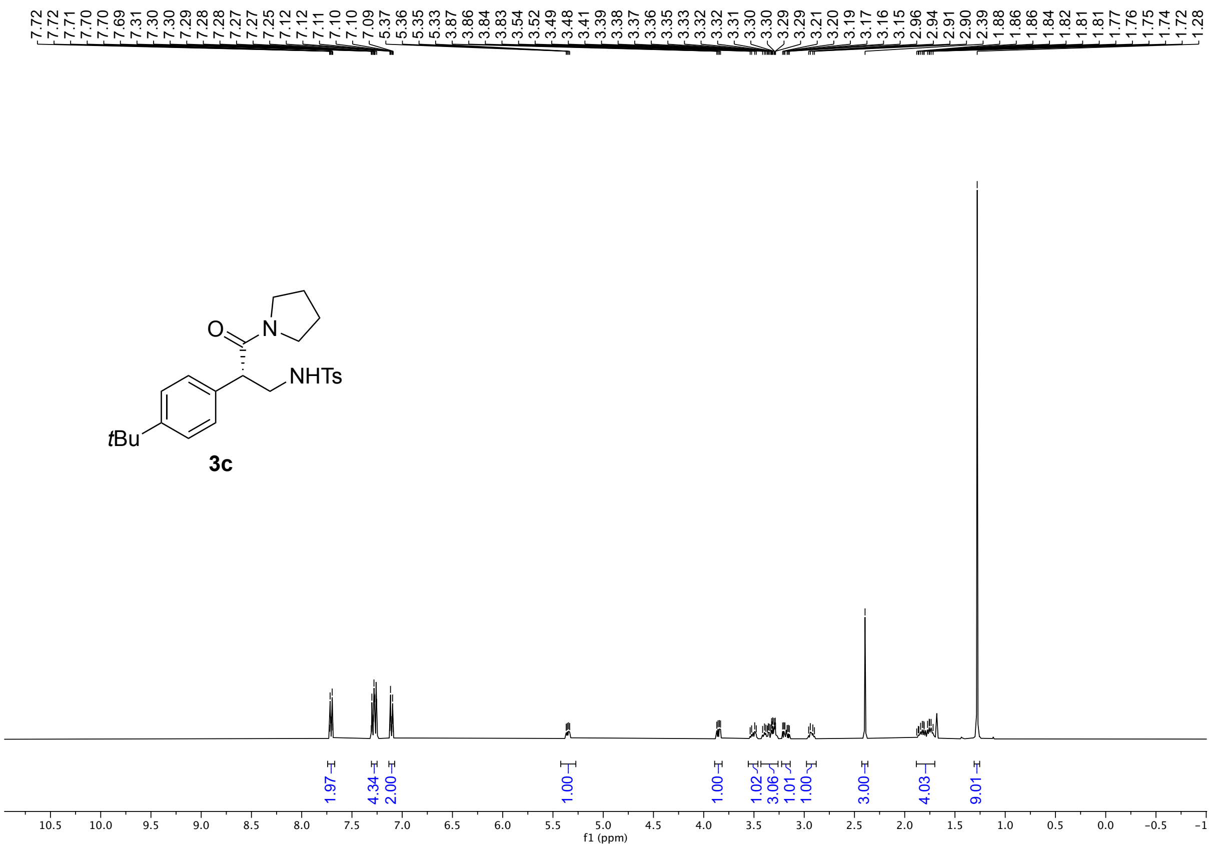
7.72 7.72 7.71 7.70 7.69 7.31 7.30 7.30 7.29 7.28 7.28 7.27 7.27 7.25 7.12 7.12 7.11 7.10 7.10 7.09 5.37 5.36 5.35 5.33 3.87 3.86 3.84 3.83 3.54 3.52 3.49 3.48 3.41 3.39 3.38 3.37 3.36 3.35 3.33 3.32 3.32 3.31 3.30 3.30 3.29 3.29 3.21 3.20 3.19 3.17 3.16 3.15 2.96 2.94 2.91 2.90 2.39 1.88 1.86 1.84 1.82 1.81 1.81 1.77 1.76 1.75 1.74 1.72 1.28
O
N
NHTs
tBu
3c
1.97
4.34
2.00
1.00
1.00
1.02
3.06
1.01
1.00
3.00
4.03
9.01
f1 (ppm)

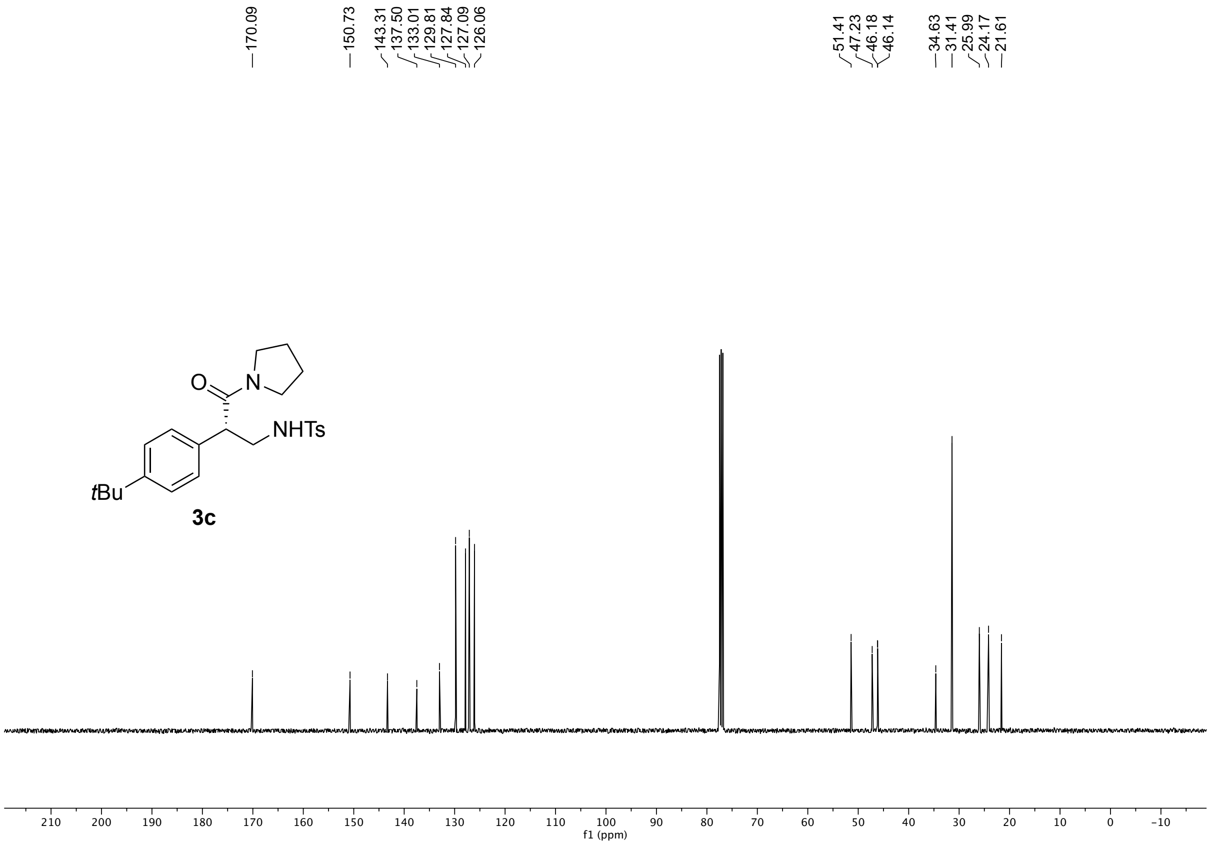
170.09
150.73
143.31 137.50 133.01 129.81 127.84 127.09 126.06
51.41 47.23 46.18 46.14
34.63
31.41
25.99 24.17
21.61
O
N
NHTs
tBu
3c
f1 (ppm)

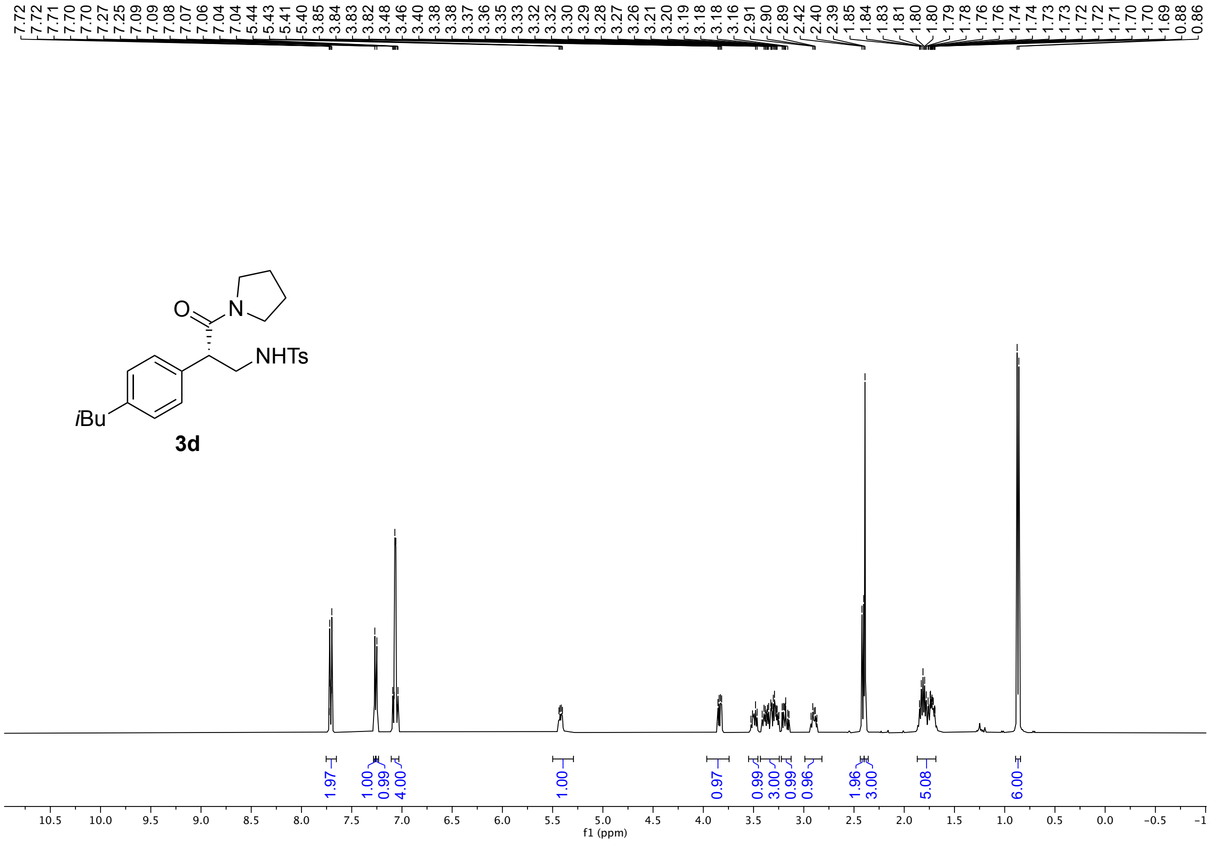
7.72 7.72 7.71 7.70 7.70 7.27 7.25 7.09 7.09 7.08 7.07 7.06 7.04 7.04 5.44 5.43 5.41 5.40 3.85 3.84 3.83 3.82 3.48 3.46 3.40 3.38 3.38 3.37 3.36 3.35 3.33 3.32 3.30 3.29 3.28 3.27 3.26 3.21 3.20 3.19 3.18 3.18 3.16 2.91 2.90 2.89 2.42 2.40 2.39 1.85 1.84 1.83 1.81 1.80 1.80 1.79 1.78 1.76 1.74 1.74 1.73 1.73 1.72 1.72 1.71 1.70 1.69 0.88 0.86
O
N
NHTs
iBu
3d
1.97 1.00 0.99 4.00 1.00 0.97 0.99 3.00 0.99 0.96 1.96 3.00 5.08 6.00
10.5 10.0 9.5 9.0 8.5 8.0 7.5 7.0 6.5 6.0 5.5 5.0 4.5 4.0 3.5 3.0 2.5 2.0 1.5 1.0 0.5 0.0 -0.5 -1
f1 (ppm)

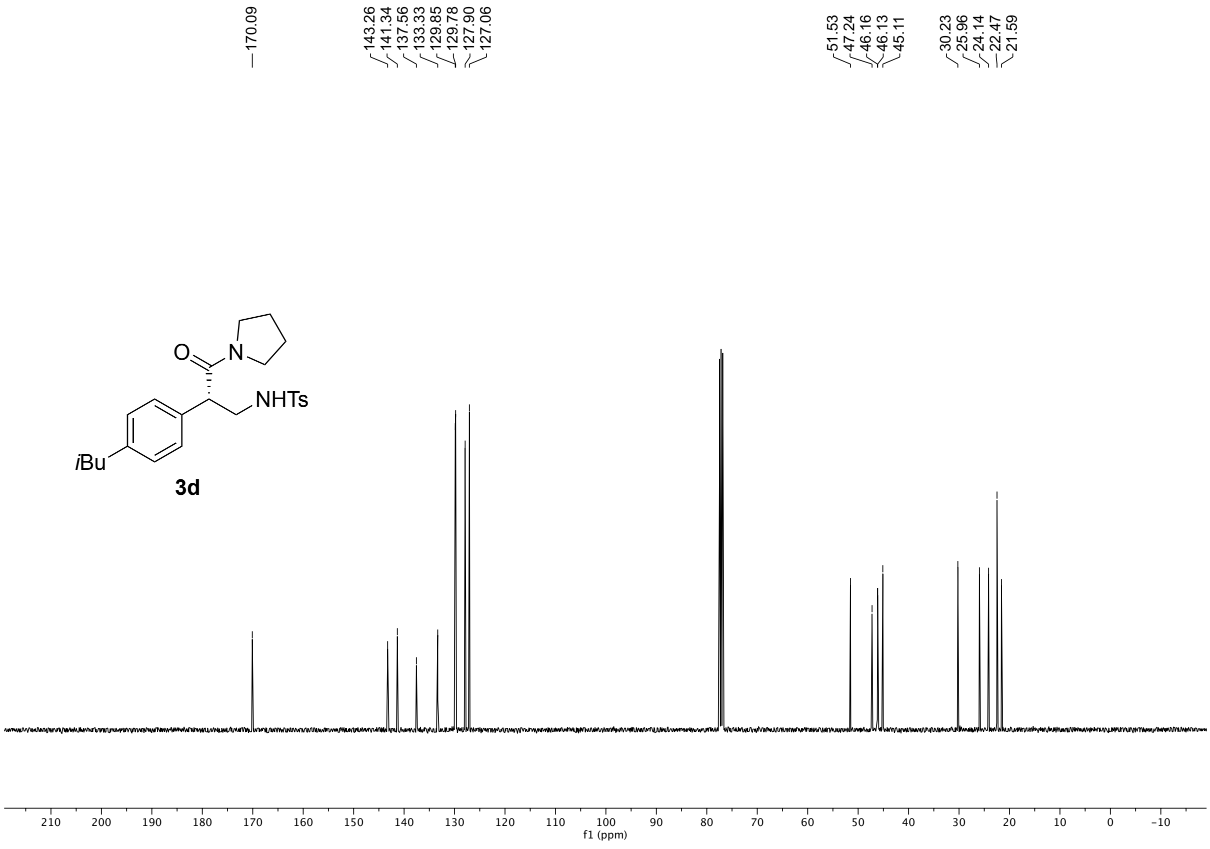
170.09
143.26 141.34 137.56 133.33 129.85 129.78 127.90 127.06
51.53 47.24 46.16 46.13 45.11
30.23 25.96 24.14 22.47 21.59
O
N
NHTs
iBu
3d
210 200 190 180 170 160 150 140 130 120 110 100 90 80 70 60 50 40 30 20 10 0 -10
f1 (ppm)

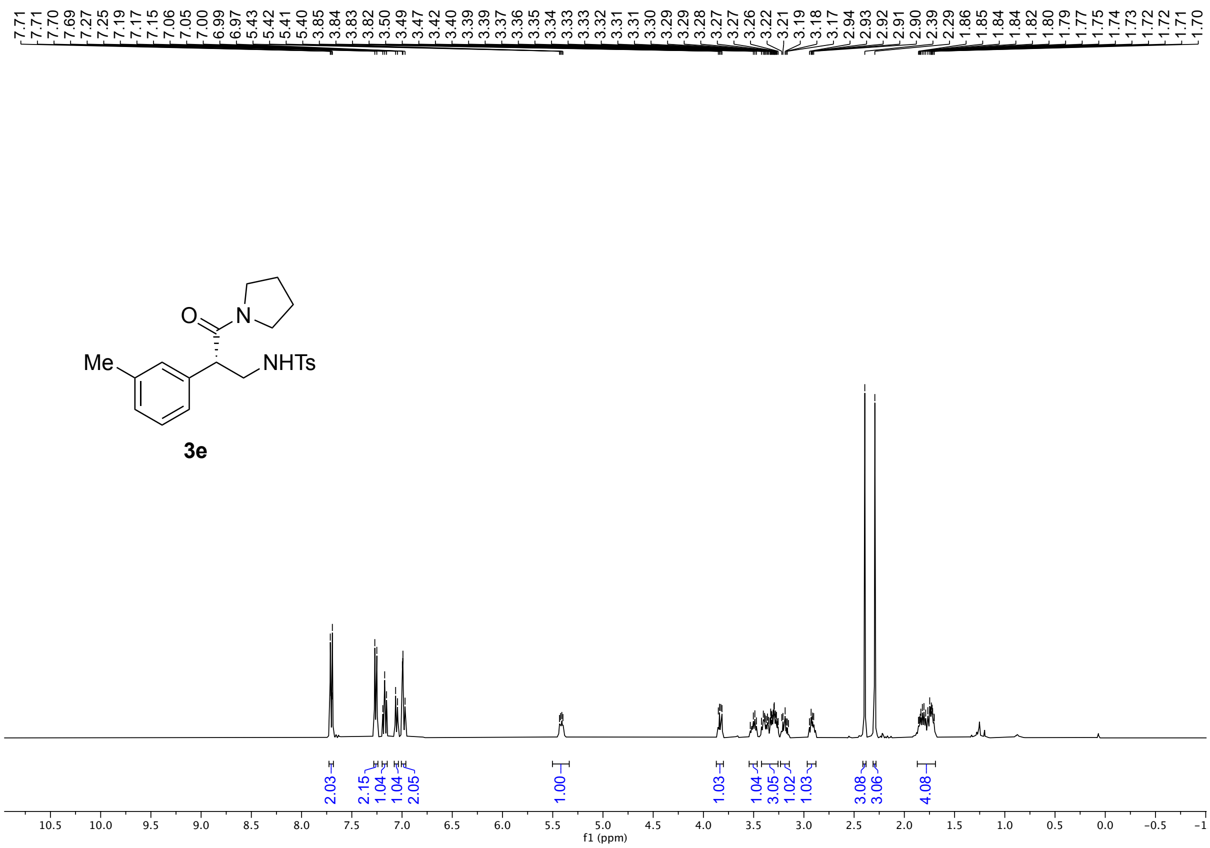
7.71 7.71 7.70 7.69 7.27 7.25 7.19 7.17 7.15 7.06 7.05 7.00 6.99 6.97 5.43 5.42 5.41 5.40 3.85 3.84 3.83 3.82 3.50 3.49 3.47 3.42 3.40 3.39 3.39 3.37 3.36 3.35 3.34 3.33 3.33 3.32 3.31 3.31 3.30 3.29 3.29 3.28 3.27 3.27 3.26 3.22 3.21 3.19 3.18 3.17 2.94 2.93 2.92 2.91 2.90 2.39 2.29 1.86 1.85 1.84 1.84 1.82 1.80 1.79 1.77 1.75 1.74 1.73 1.72 1.71 1.70
O
N
Me
NHTs
2.03 2.15 1.04 1.04 2.05 1.00 1.03 1.04 3.05 1.02 1.03 3.08 3.06 4.08
f1 (ppm)

**3e**

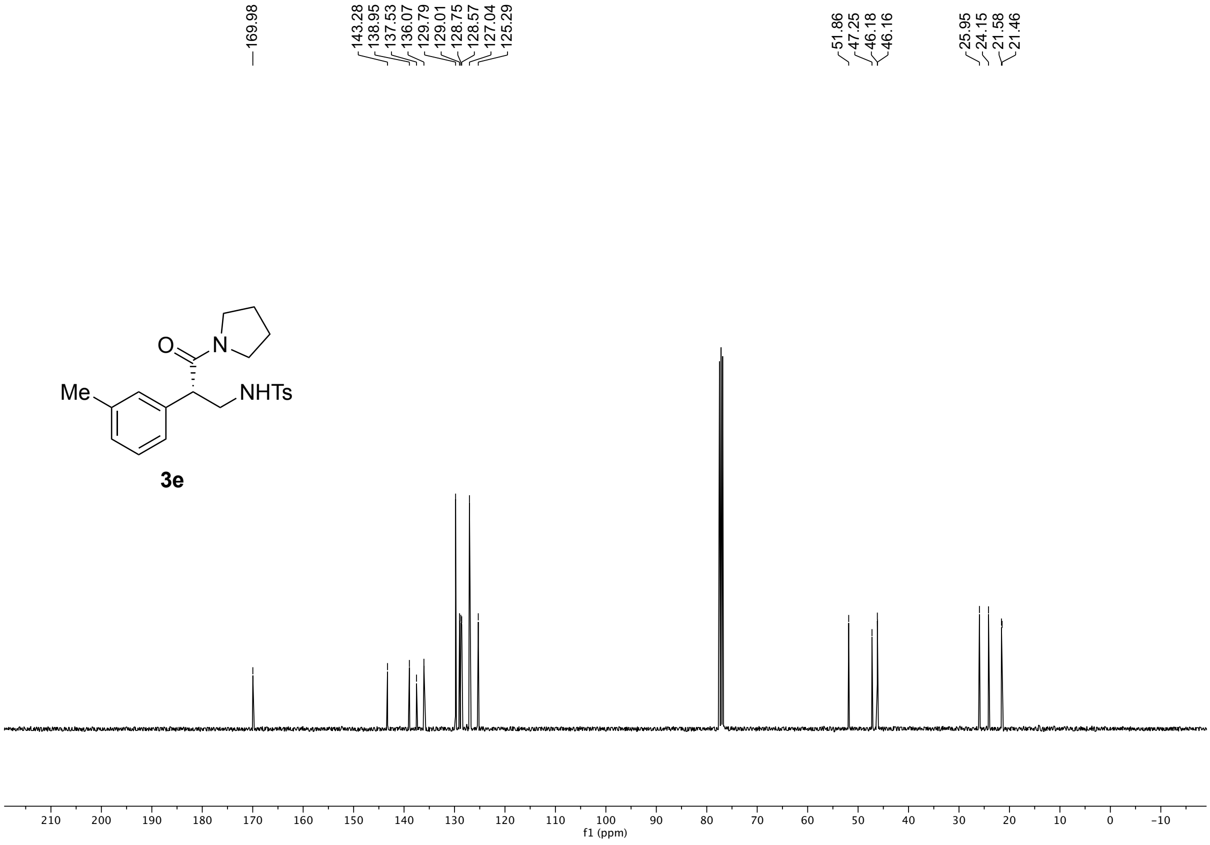
169.98
143.28 138.95 137.53 136.07 129.79 129.01 128.75 128.57 127.04 125.29
51.86 47.25 46.18 46.16
25.95 24.15 21.58 21.46
O
N
Me
NHTs
f1 (ppm)

**3e**

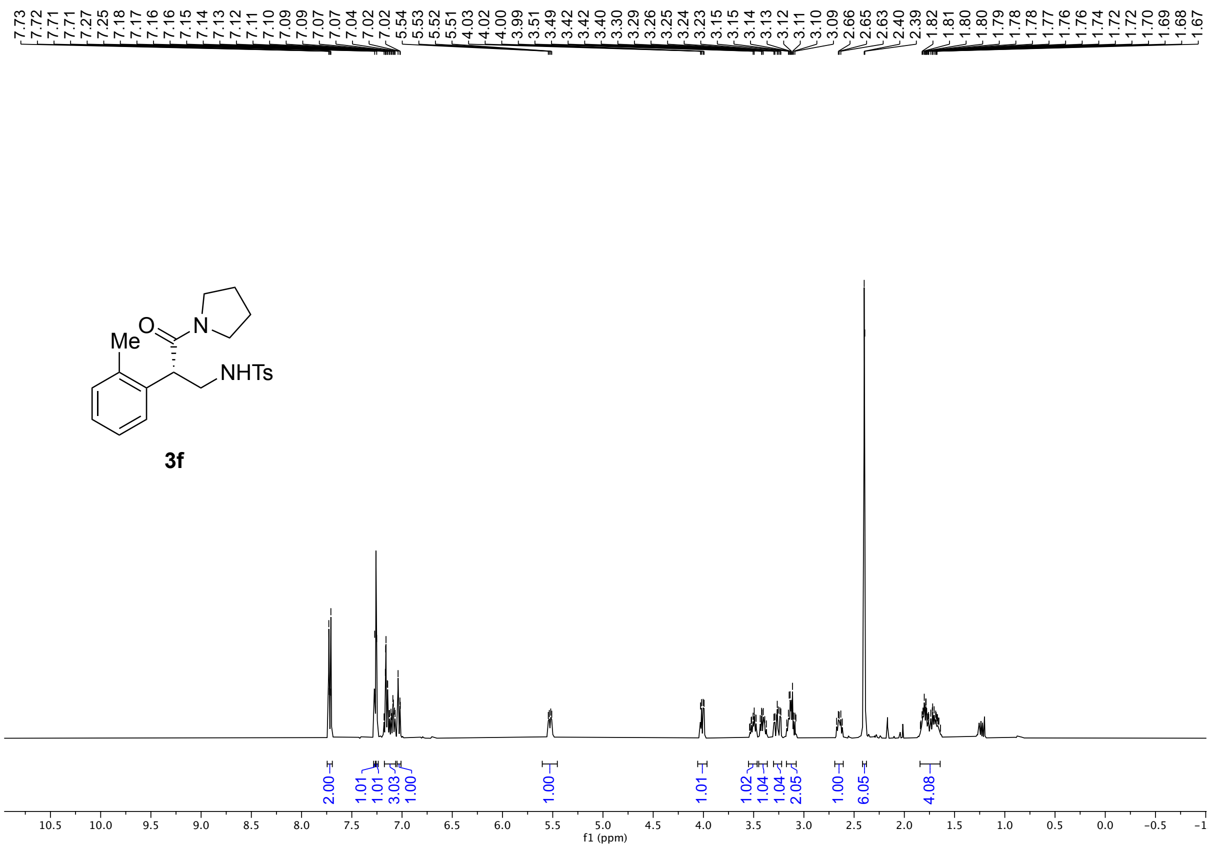
7.73 7.72 7.71 7.71 7.27 7.25 7.18 7.17 7.16 7.16 7.15 7.14 7.13 7.12 7.11 7.10 7.09 7.09 7.07 7.07 7.04 7.02 7.02 5.54 5.53 5.52 5.51 4.03 4.02 4.00 3.99 3.51 3.49 3.42 3.42 3.40 3.30 3.29 3.26 3.25 3.24 3.23 3.15 3.15 3.14 3.13 3.12 3.11 3.10 3.09 2.66 2.65 2.63 2.40 2.39 1.82 1.81 1.80 1.79 1.78 1.78 1.77 1.76 1.74 1.72 1.72 1.70 1.69 1.68 1.67
Me
O
N
NHTs
3f
2.00 1.01 1.01 3.03 1.00 1.00 1.01 1.02 1.04 1.04 2.05 1.00 6.05 4.08
f1 (ppm)

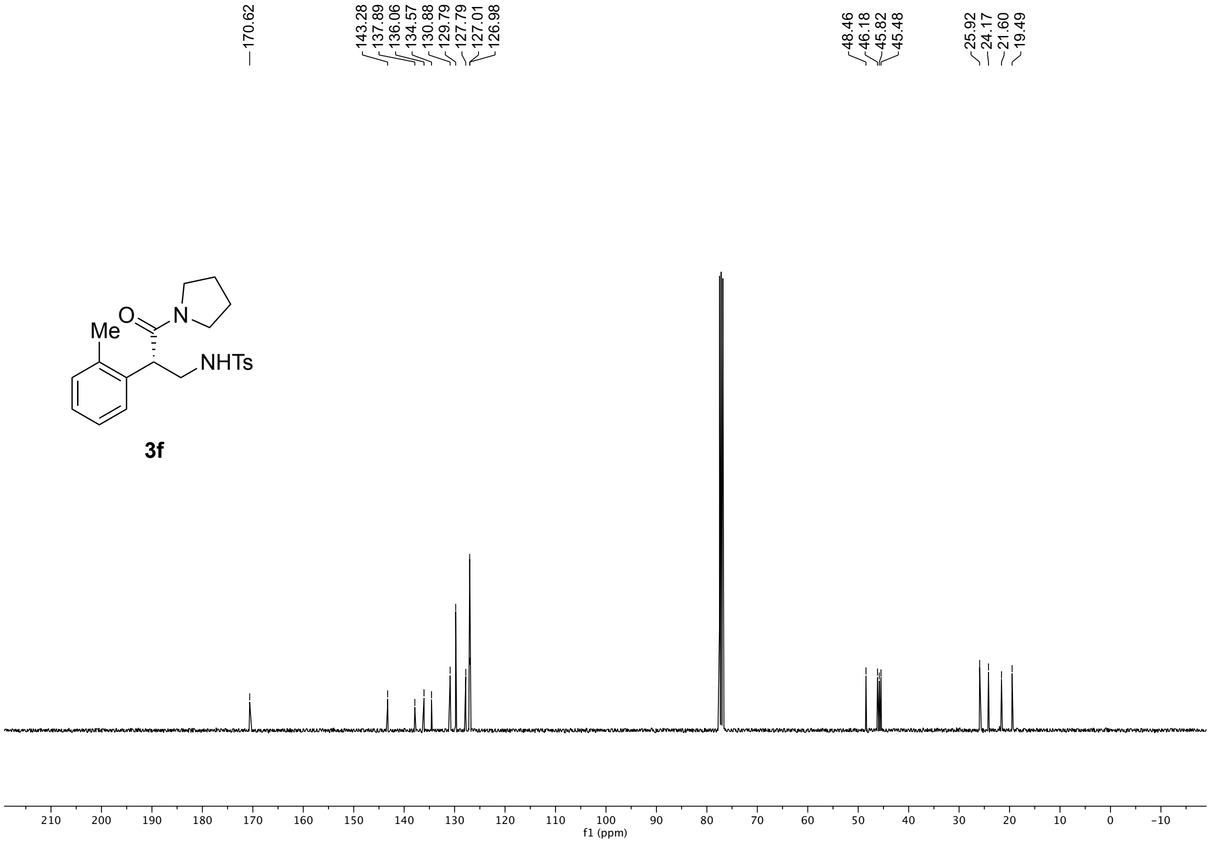
170.62
143.28 137.89 136.06 134.57 130.88 129.79 127.79 127.01 126.98
48.46 46.18 45.82 45.48
25.92 24.17 21.60 19.49
Me
O
N
NHTs
3f
f1 (ppm)

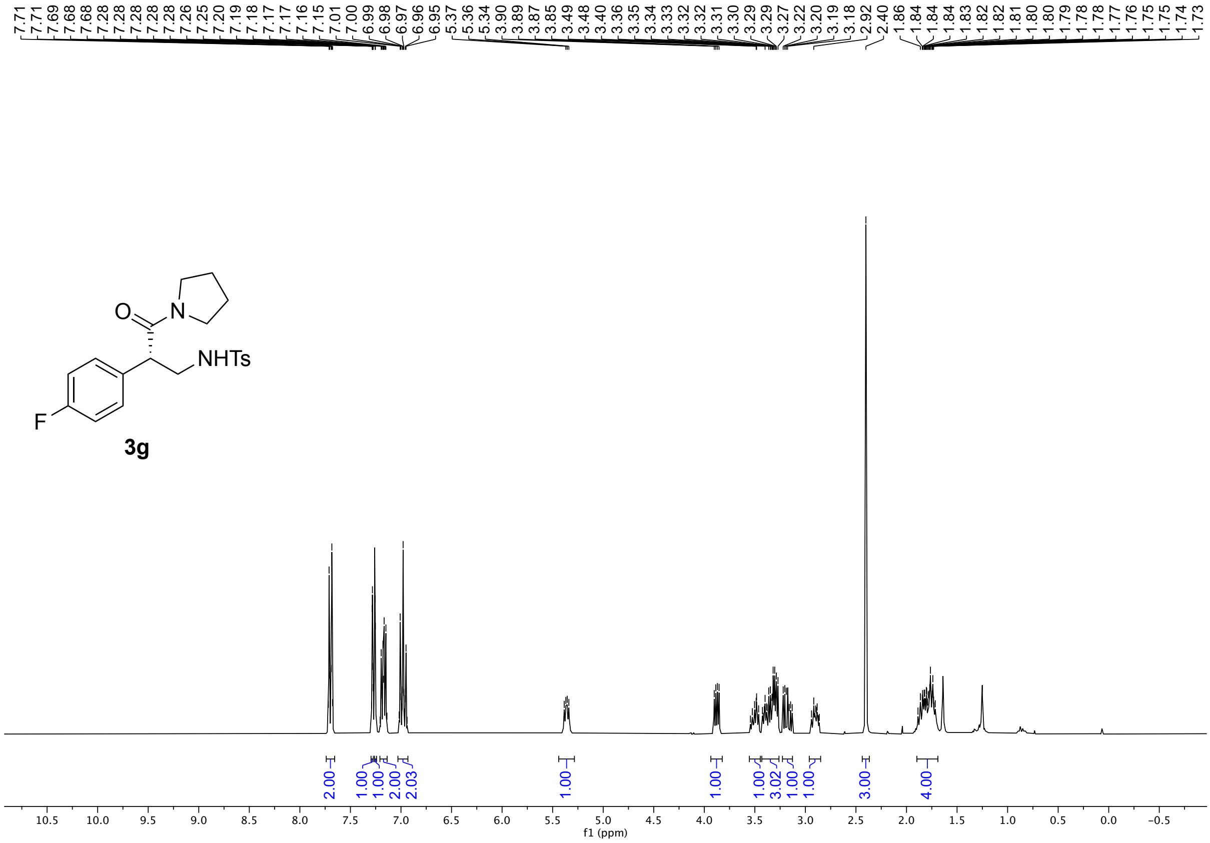
7.71 7.71 7.69 7.68 7.68 7.28 7.28 7.28 7.28 7.28 7.26 7.25 7.20 7.19 7.18 7.17 7.16 7.15 7.01 7.00 6.99 6.98 6.97 6.96 6.95 5.37 5.36 5.34 3.90 3.89 3.87 3.85 3.49 3.48 3.40 3.36 3.35 3.34 3.33 3.32 3.32 3.31 3.30 3.29 3.27 3.22 3.20 3.19 3.18 2.92 2.40 1.86 1.84 1.84 1.84 1.83 1.82 1.82 1.81 1.80 1.80 1.79 1.78 1.78 1.77 1.76 1.75 1.74 1.73
O
N
NHTs
F
3g
2.00 1.00 1.00 2.00 2.03 1.00 1.00 1.00 3.02 1.00 1.00 3.00 4.00
10.5 10.0 9.5 9.0 8.5 8.0 7.5 7.0 6.5 6.0 5.5 5.0 4.5 4.0 3.5 3.0 2.5 2.0 1.5 1.0 0.5 0.0 -0.5
f1 (ppm)

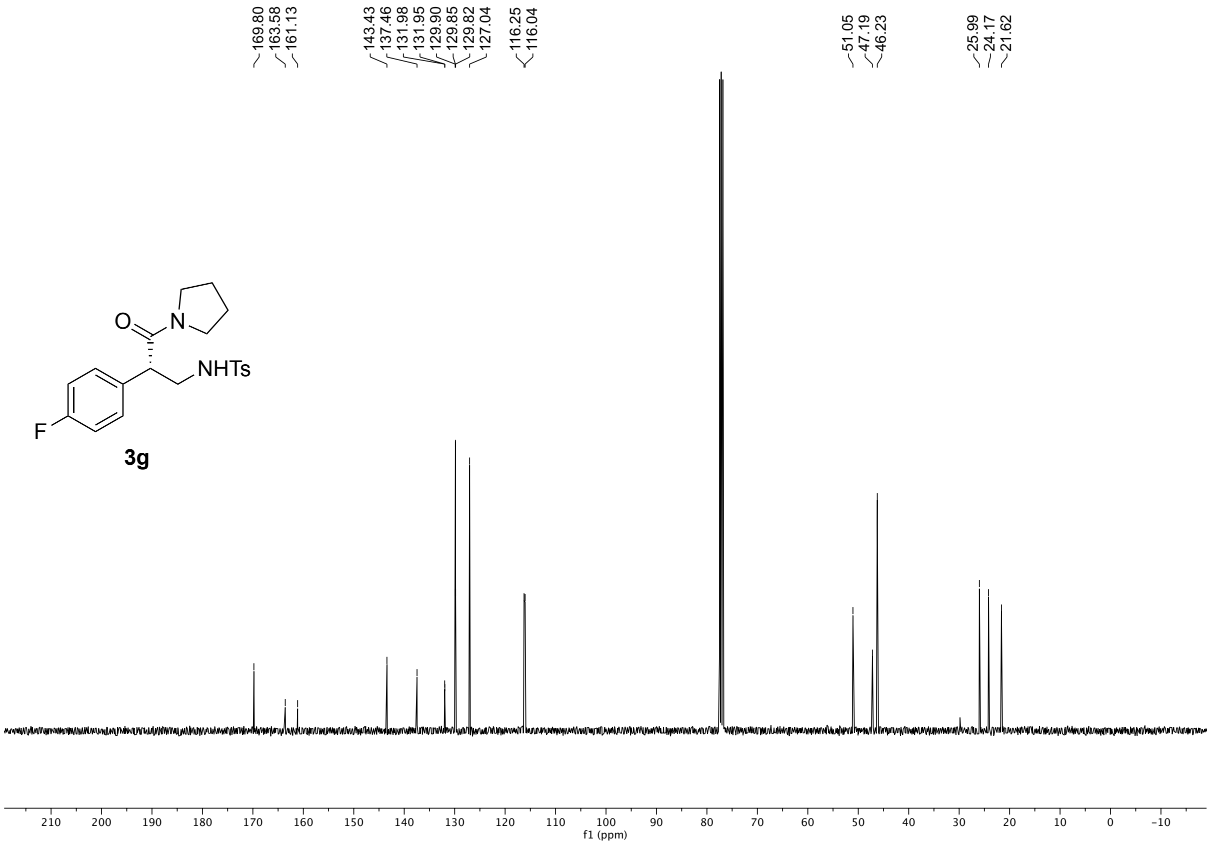
169.80 163.58 161.13 143.43 137.46 131.98 131.95 129.90 129.85 129.82 127.04 116.25 116.04 51.05 47.19 46.23 25.99 24.17 21.62
O
N
NHTs
F
3g
210 200 190 180 170 160 150 140 130 120 110 100 90 80 70 60 50 40 30 20 10 0 -10
f1 (ppm)

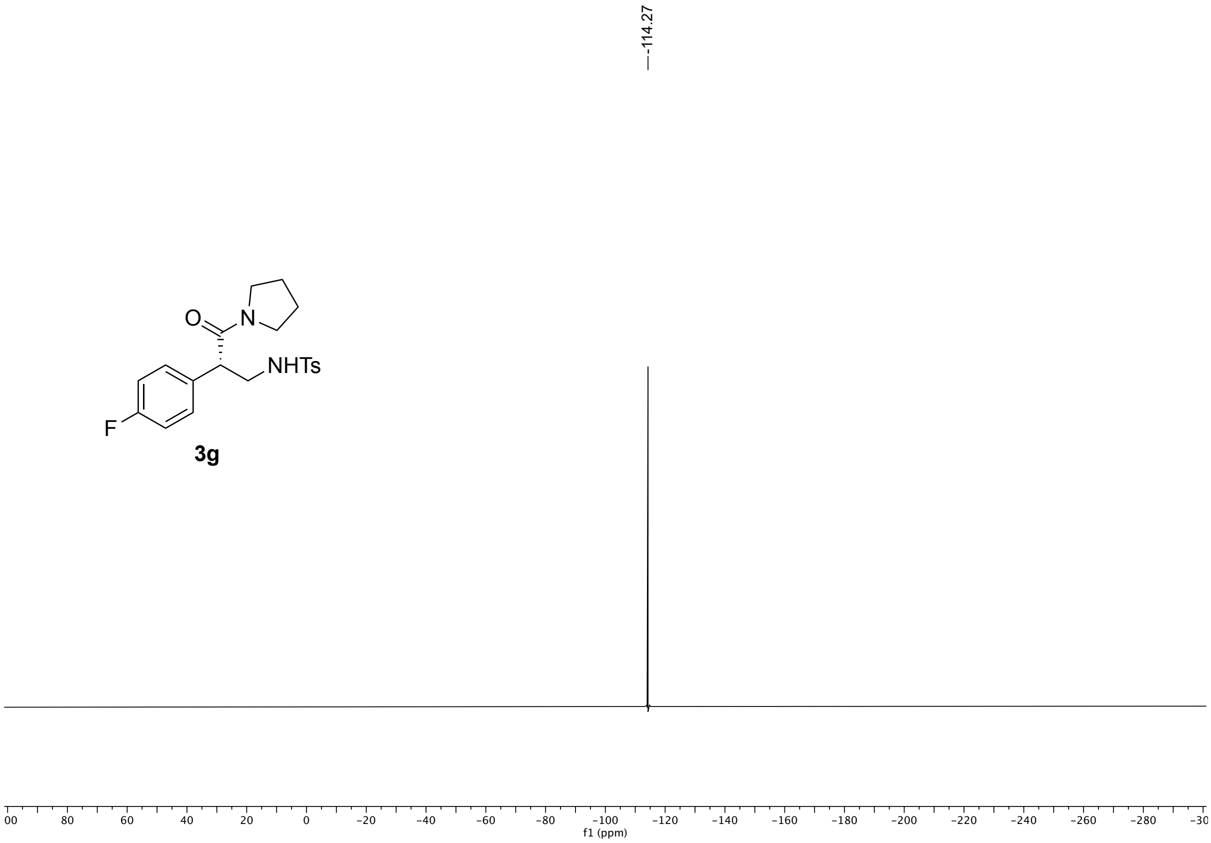

-114.27
O
N
NHTs
F
3g
00 80 60 40 20 0 -20 -40 -60 -80 -100 -120 -140 -160 -180 -200 -220 -240 -260 -280 -30
f1 (ppm)

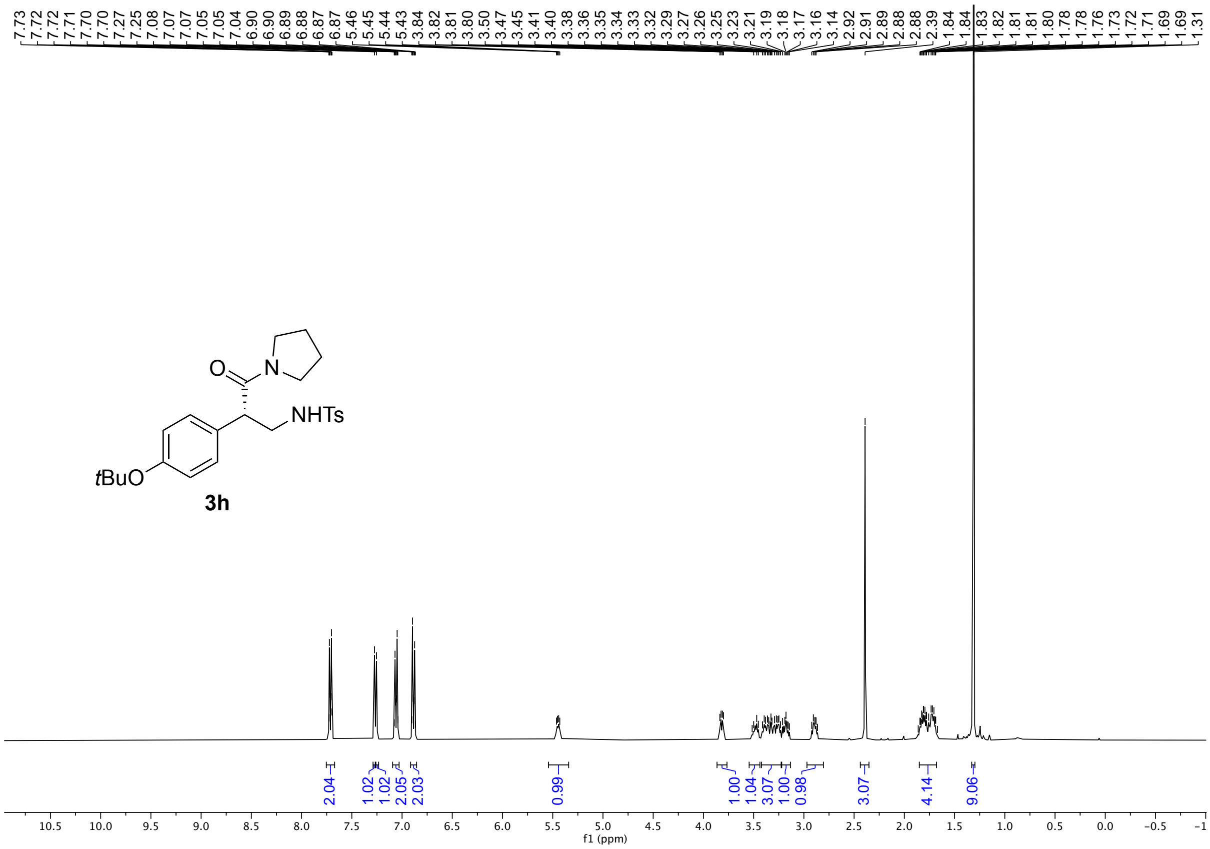
7.73 7.72 7.72 7.71 7.70 7.70 7.27 7.25 7.08 7.07 7.07 7.05 7.05 7.04 6.90 6.90 6.89 6.88 6.87 6.87 5.46 5.45 5.44 5.43 3.84 3.82 3.81 3.80 3.50 3.47 3.45 3.41 3.40 3.38 3.36 3.35 3.34 3.33 3.32 3.29 3.27 3.26 3.25 3.23 3.21 3.19 3.18 3.17 3.16 3.14 2.92 2.91 2.89 2.88 2.39 1.84 1.84 1.83 1.82 1.81 1.81 1.80 1.78 1.76 1.73 1.72 1.71 1.69 1.69 1.31
O
N
NHTs
tBuO
3h
2.04 1.02 1.02 2.05 2.03 0.99 1.00 1.04 3.07 1.00 0.98 3.07 4.14 9.06
f1 (ppm)

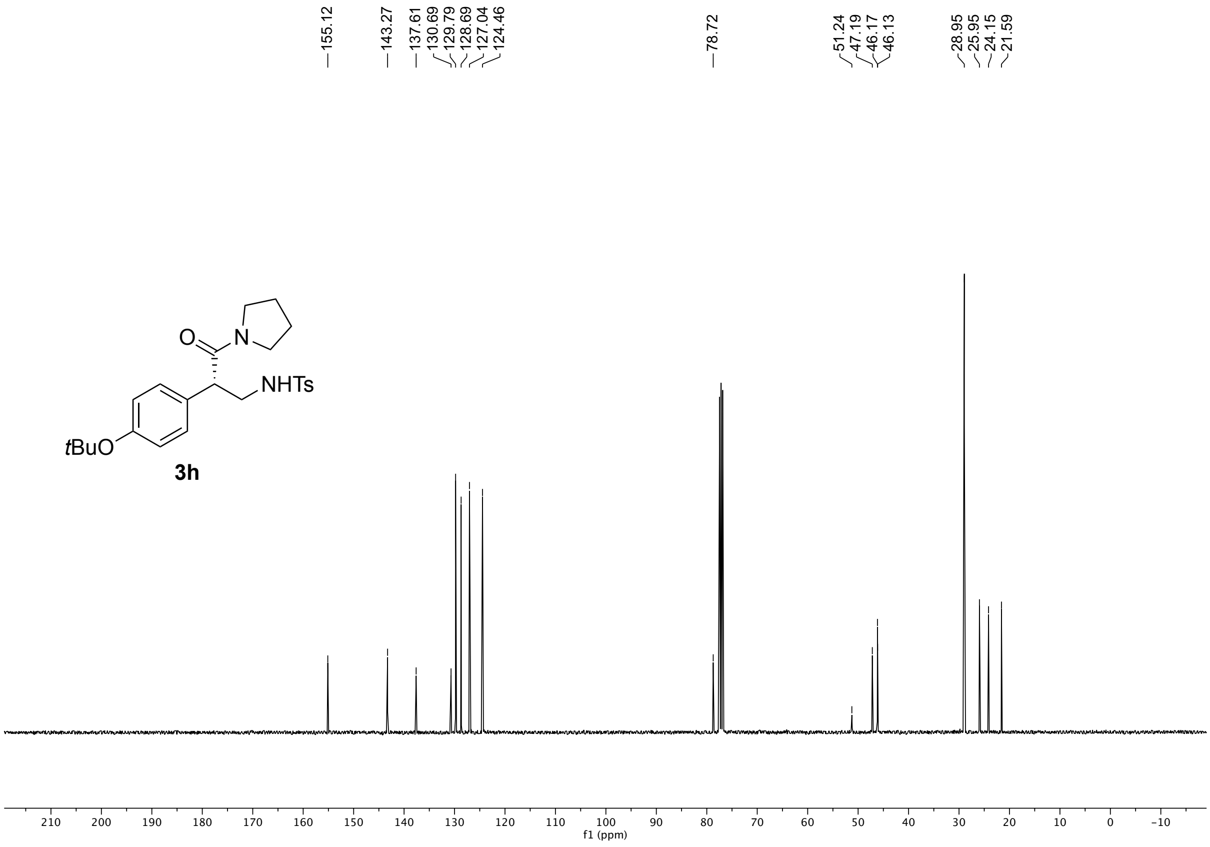
155.12 143.27 137.61 130.69 129.79 128.69 127.04 124.46 78.72 51.24 47.19 46.17 46.13 28.95 25.95 24.15 21.59
O
N
NHTs
tBuO
3h
f1 (ppm)

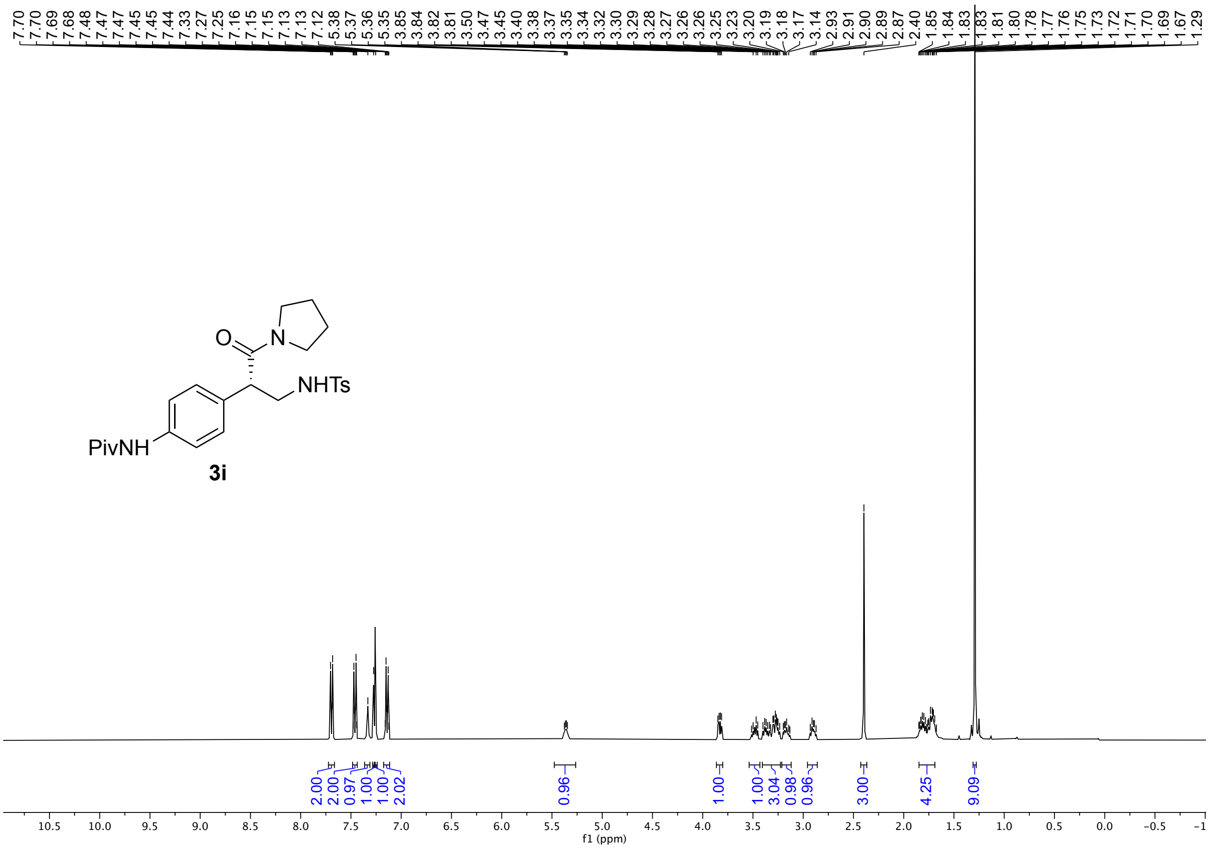
7.70 7.70 7.69 7.68 7.48 7.47 7.47 7.45 7.45 7.44 7.33 7.27 7.25 7.16 7.15 7.15 7.13 7.13 7.12 5.38 5.37 5.36 5.35 3.85 3.84 3.82 3.81 3.50 3.47 3.45 3.40 3.38 3.37 3.35 3.34 3.32 3.30 3.29 3.28 3.27 3.26 3.26 3.25 3.23 3.20 3.19 3.18 3.17 3.14 2.93 2.91 2.90 2.89 2.87 2.40 1.85 1.84 1.83 1.83 1.81 1.80 1.78 1.77 1.76 1.75 1.73 1.72 1.71 1.70 1.69 1.67 1.29
O
N
NHTs
PivNH
3i
2.00 2.00 0.97 1.00 1.00 2.02 0.96 1.00 1.00 3.04 0.98 0.96 3.00 4.25 9.09
f1 (ppm)

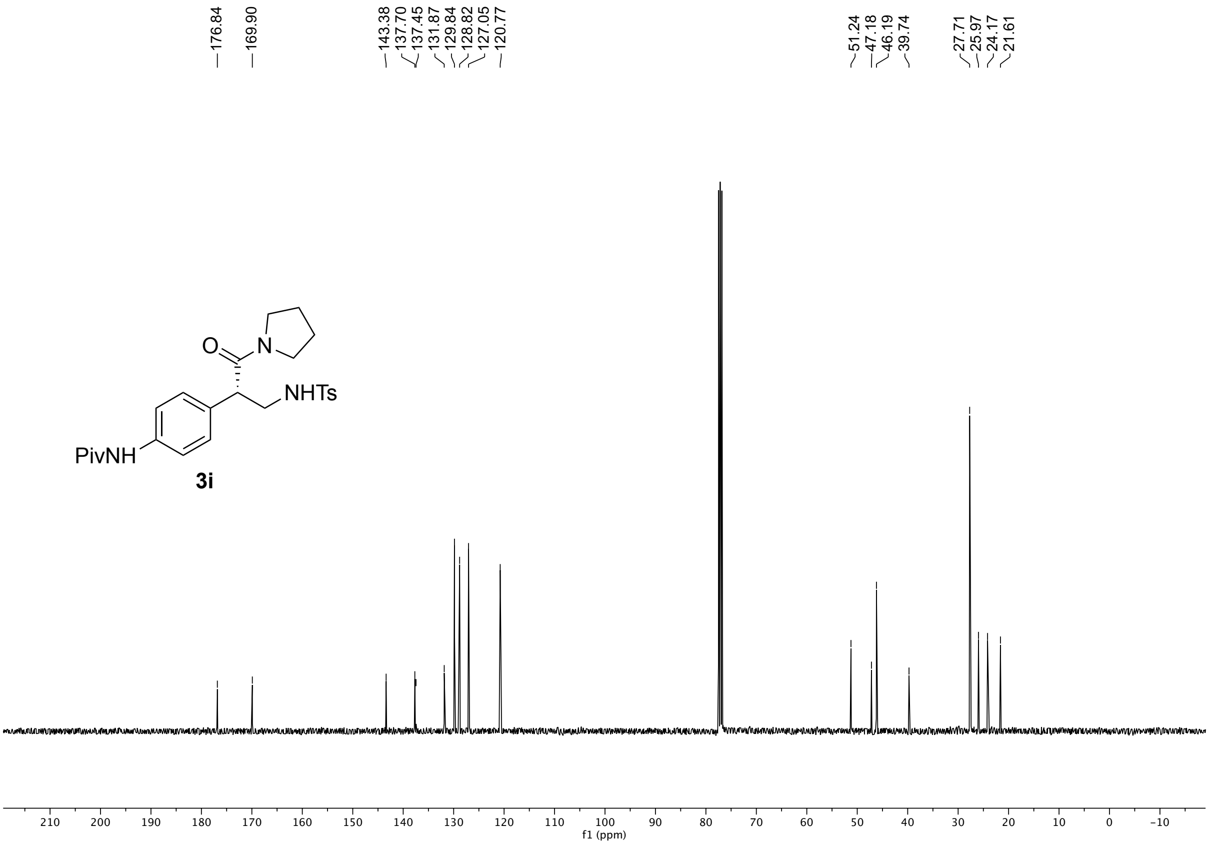
176.84 169.90 143.38 137.70 137.45 131.87 129.84 128.82 127.05 120.77 51.24 47.18 46.19 39.74 27.71 25.97 24.17 21.61
O
N
NHTs
PivNH
3i
f1 (ppm)

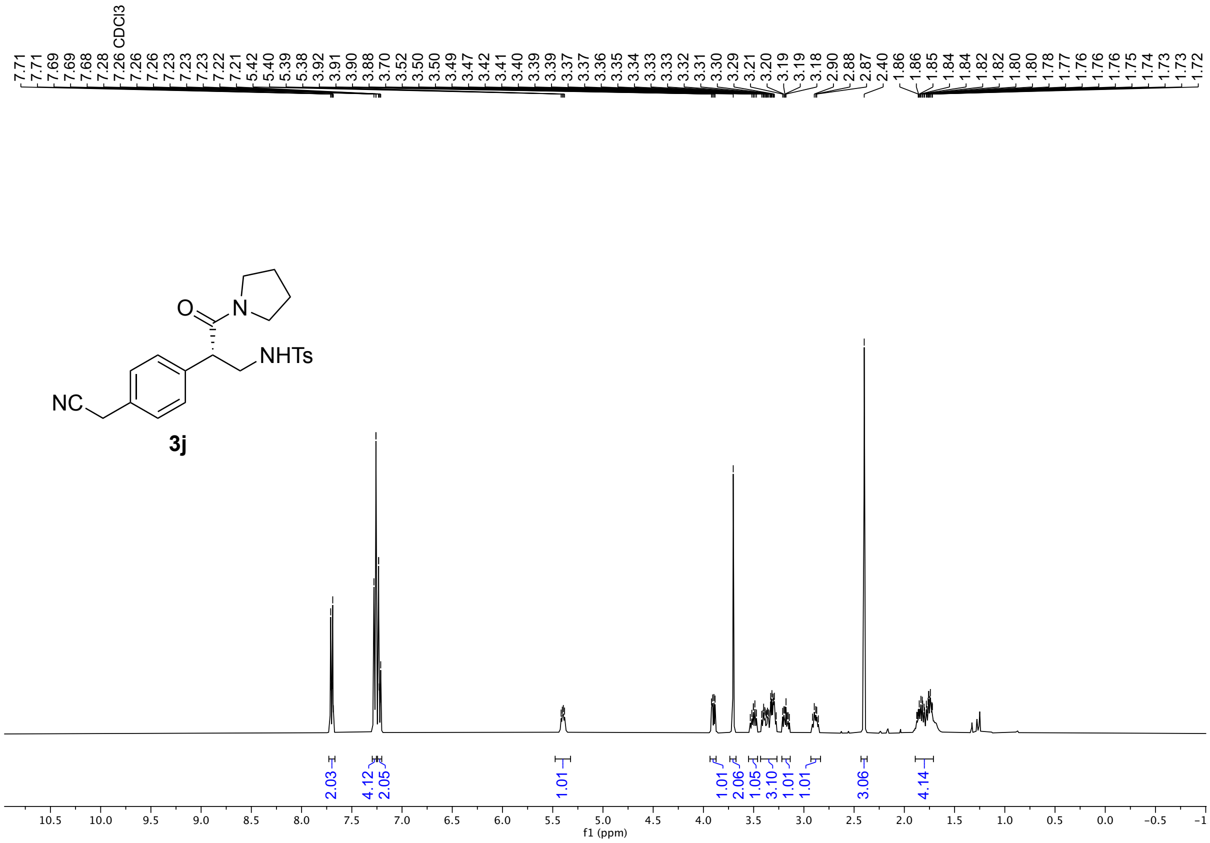
7.71 7.71 7.69 7.69 7.68 7.28 7.26 CDCl3 7.26 7.26 7.23 7.23 7.23 7.22 7.21 5.42 5.40 5.39 5.38 3.92 3.91 3.90 3.88 3.70 3.52 3.50 3.50 3.49 3.47 3.42 3.41 3.40 3.39 3.39 3.37 3.37 3.36 3.35 3.34 3.33 3.33 3.32 3.31 3.30 3.29 3.21 3.20 3.19 3.19 3.18 2.90 2.88 2.87 2.40 1.86 1.86 1.85 1.84 1.84 1.82 1.82 1.80 1.80 1.78 1.77 1.76 1.76 1.76 1.75 1.74 1.73 1.73 1.72
O
N
NHTs
NC
3j
2.03
4.12
2.05
1.01
1.01
2.06
1.05
3.10
1.01
1.01
3.06
4.14
f1 (ppm)

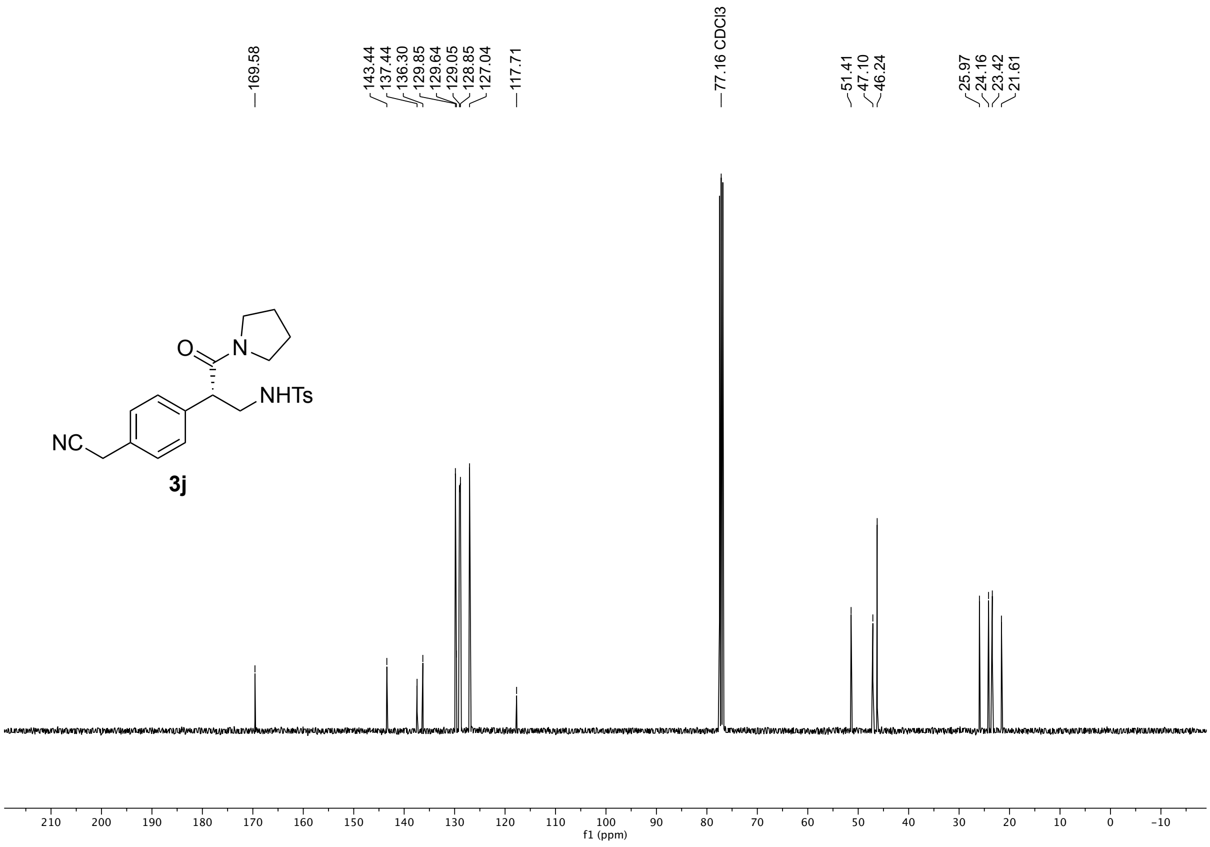
169.58
143.44 137.44 136.30 129.85 129.64 129.05 128.85 127.04
117.71
77.16 CDCl3
51.41 47.10 46.24
25.97 24.16 23.42 21.61
O
N
NHTs
NC
3j
f1 (ppm)

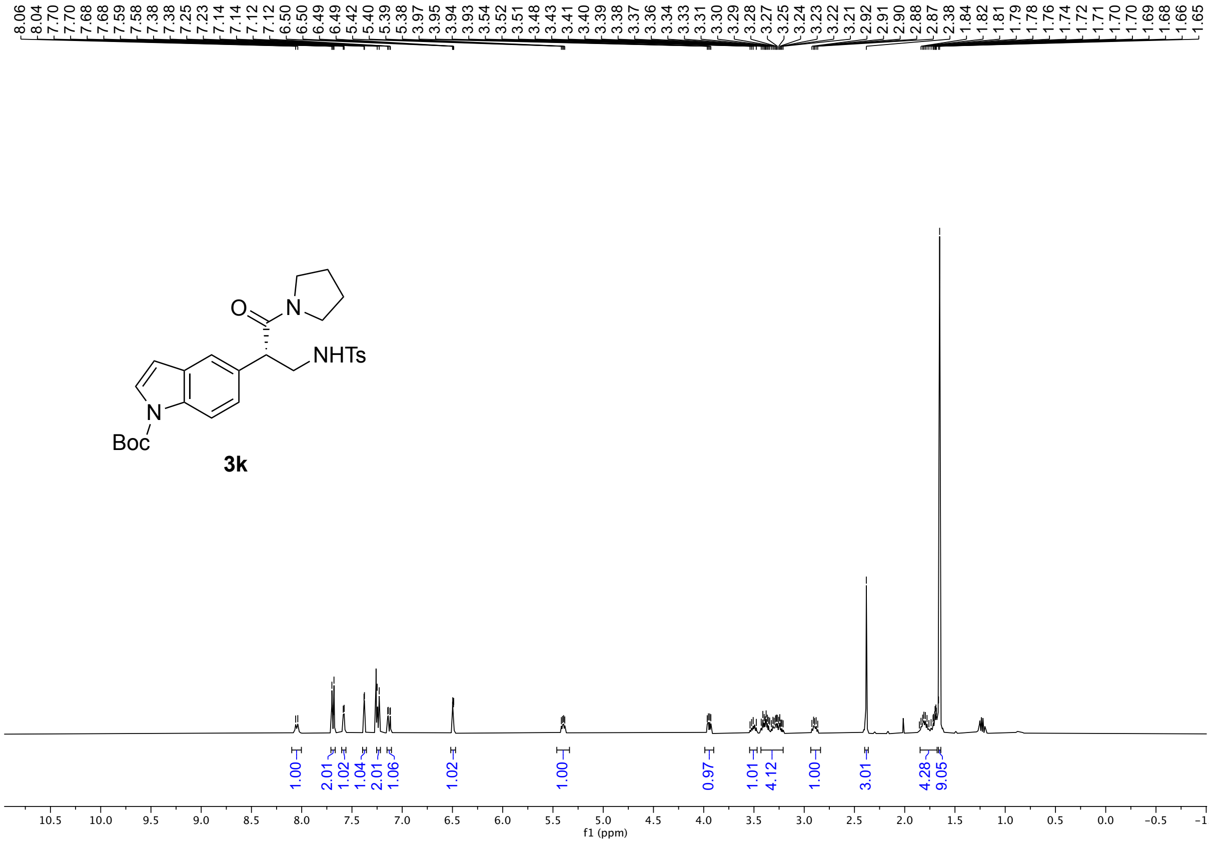
8.06 8.04 7.70 7.70 7.68 7.68 7.59 7.58 7.38 7.38 7.25 7.23 7.14 7.12 6.50 6.50 6.49 6.49 5.42 5.40 5.39 5.38 3.97 3.95 3.95 3.94 3.93 3.54 3.52 3.51 3.48 3.43 3.41 3.40 3.39 3.38 3.37 3.36 3.34 3.33 3.31 3.30 3.29 3.28 3.27 3.25 3.24 3.23 3.22 3.21 2.92 2.91 2.90 2.88 2.87 2.38 1.84 1.82 1.81 1.79 1.78 1.76 1.74 1.72 1.71 1.70 1.70 1.69 1.68 1.66 1.65
O
N
NHTs
N
Boc
3k
1.00 2.01 1.02 1.04 2.01 1.06 1.02 1.00 0.97 1.01 4.12 1.00 3.01 4.28 9.05
f1 (ppm)

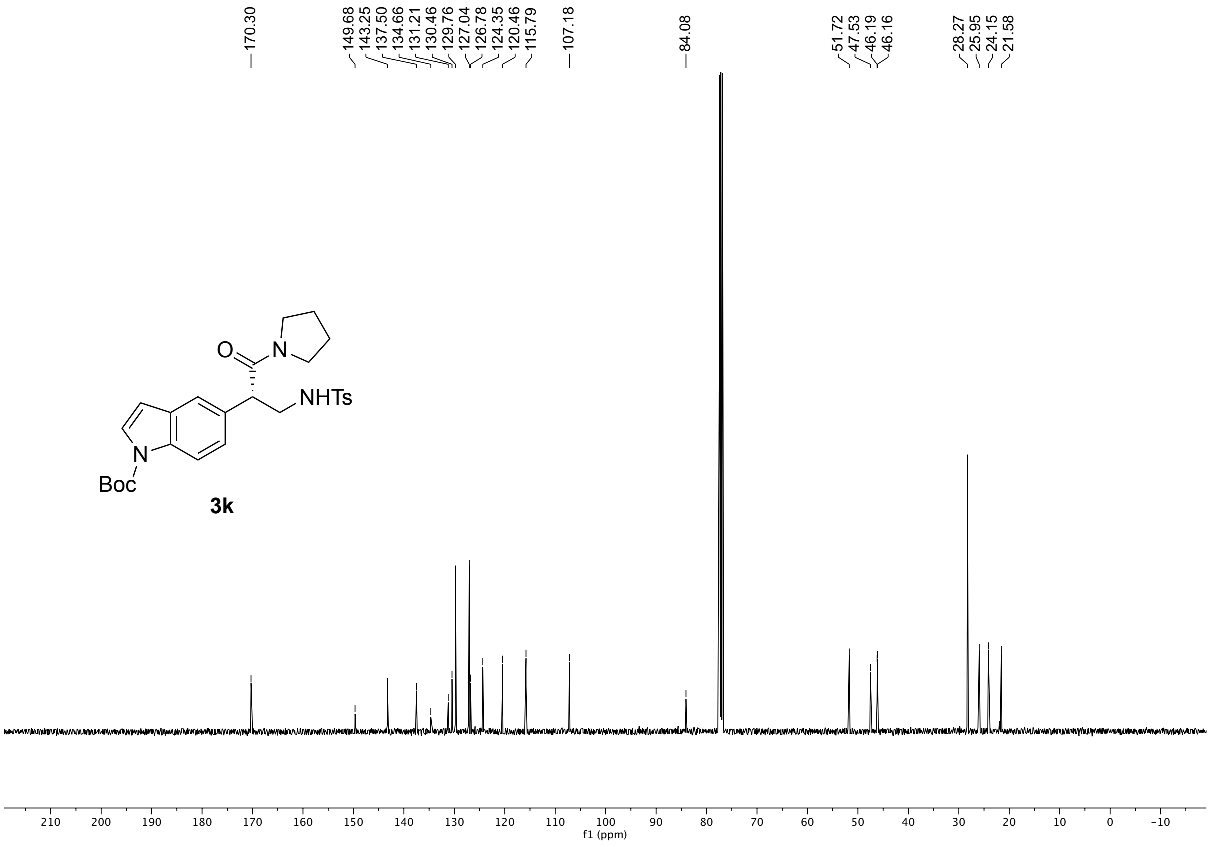
170.30
149.68 143.25 137.50 134.66 131.21 130.46 129.76 127.04 126.78 124.35 120.46 115.79
107.18
84.08
51.72 47.53 46.19 46.16
28.27 25.95 24.15 21.58
O
N
NHTs
N
Boc
3k
f1 (ppm)

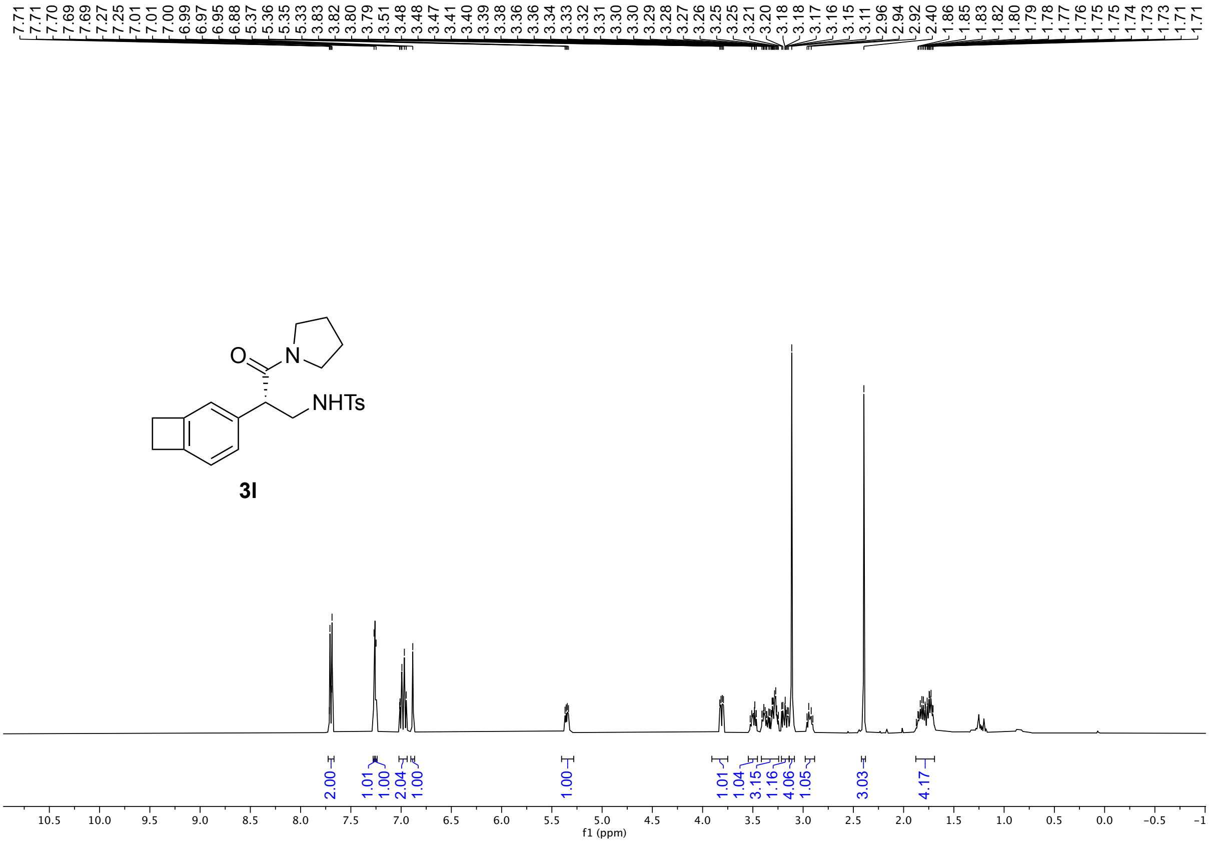
7.71 7.71 7.70 7.69 7.69 7.27 7.25 7.01 7.01 7.00 6.99 6.97 6.95 6.88 5.37 5.36 5.35 5.33 3.83 3.82 3.80 3.79 3.51 3.48 3.48 3.47 3.41 3.40 3.39 3.38 3.36 3.36 3.34 3.33 3.32 3.31 3.30 3.30 3.29 3.28 3.27 3.26 3.25 3.25 3.21 3.20 3.18 3.18 3.17 3.16 3.15 3.11 2.96 2.94 2.92 2.40 1.86 1.85 1.83 1.82 1.80 1.79 1.78 1.77 1.76 1.75 1.74 1.73 1.73 1.71 1.71
O
N
NHTs
3l
2.00 1.01 1.00 2.04 1.00 1.00 1.01 1.04 3.15 1.16 4.06 1.05 3.03 4.17
10.5 10.0 9.5 9.0 8.5 8.0 7.5 7.0 6.5 6.0 5.5 5.0 4.5 4.0 3.5 3.0 2.5 2.0 1.5 1.0 0.5 0.0 -0.5 -1
f1 (ppm)

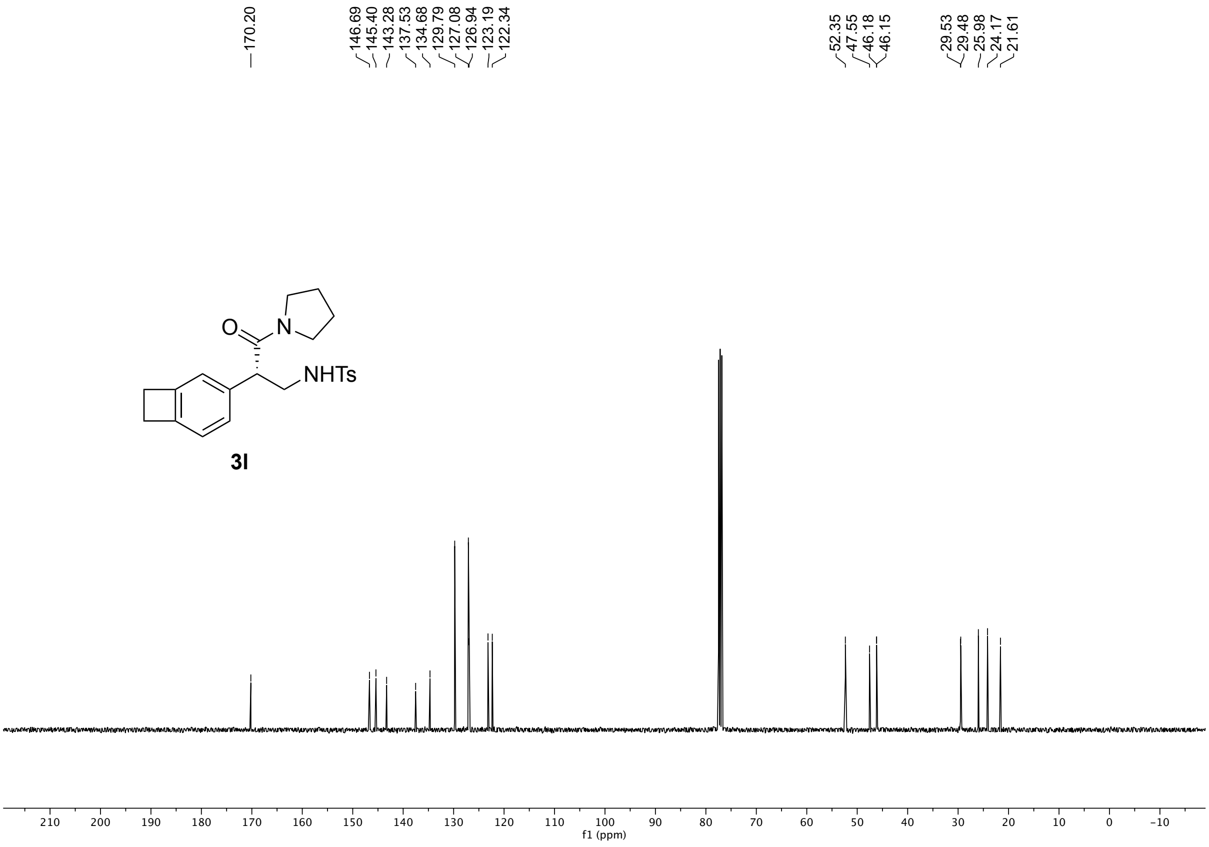
170.20
146.69 145.40 143.28 137.53 134.68 129.79 127.08 126.94 123.19 122.34
52.35 47.55 46.18 46.15
29.53 29.48 25.98 24.17 21.61
O
N
NHTs
3l
210 200 190 180 170 160 150 140 130 120 110 100 90 80 70 60 50 40 30 20 10 0 -10
f1 (ppm)

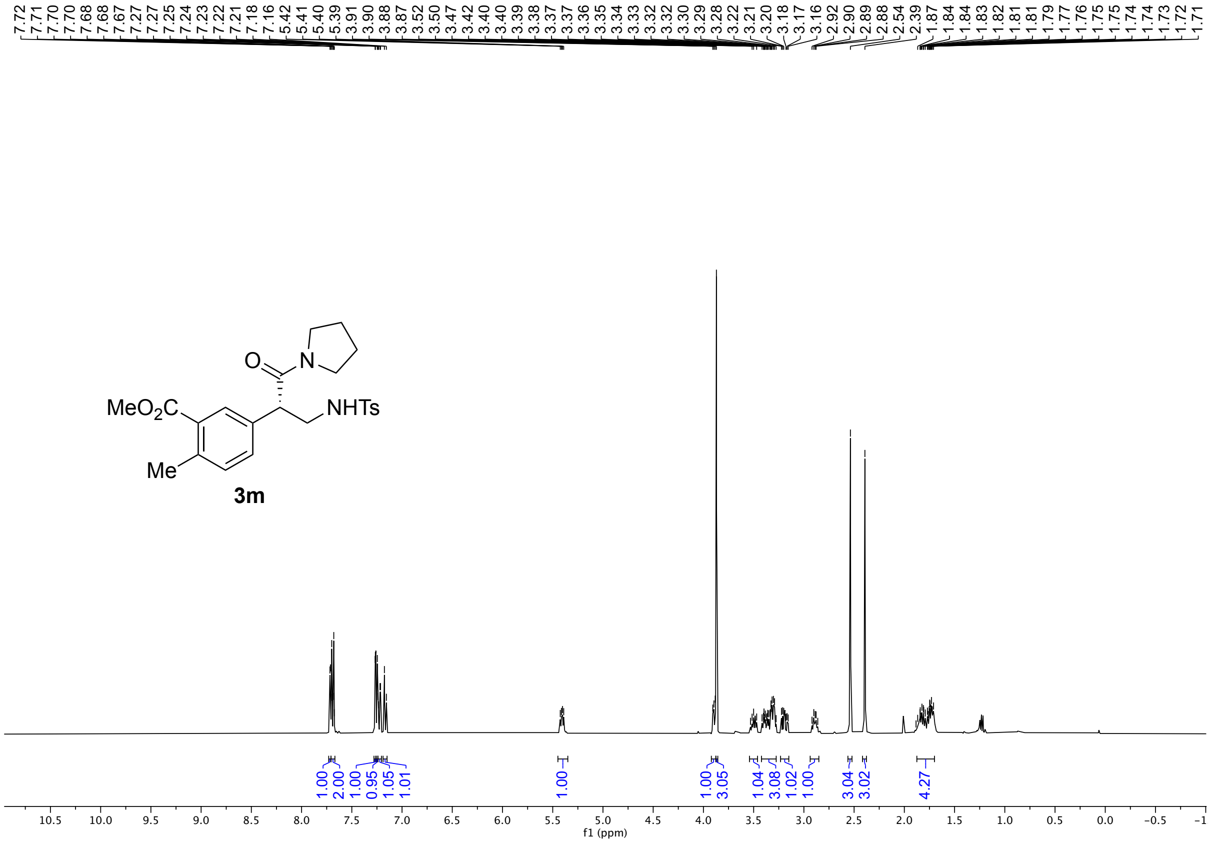
7.72 7.71 7.70 7.68 7.67 7.27 7.27 7.25 7.24 7.23 7.22 7.21 7.18 7.16 5.42 5.41 5.40 5.39 3.91 3.90 3.88 3.87 3.52 3.50 3.47 3.42 3.40 3.40 3.39 3.38 3.37 3.37 3.36 3.35 3.34 3.33 3.32 3.32 3.30 3.29 3.28 3.22 3.21 3.20 3.18 3.17 3.16 2.92 2.90 2.89 2.88 2.54 2.39 1.87 1.84 1.84 1.83 1.82 1.81 1.81 1.79 1.77 1.76 1.75 1.74 1.73 1.72 1.71
O
N
MeO2C
NHTs
Me
3m
1.00 2.00 1.00 0.95 1.05 1.01 1.00 1.00 3.05 1.04 3.08 1.02 1.00 3.04 3.02 4.27
f1 (ppm)

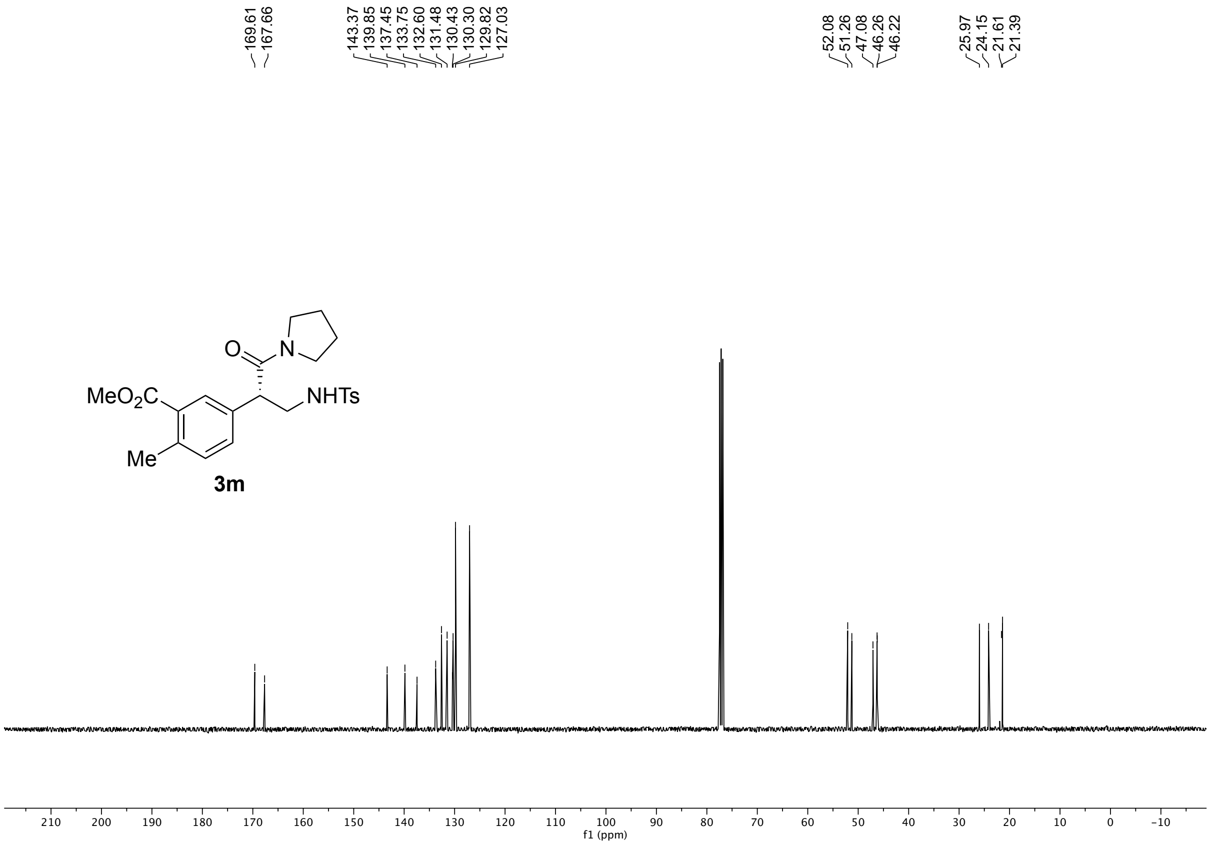
169.61 167.66 143.37 139.85 137.45 133.75 132.60 131.48 130.43 130.30 129.82 127.03 52.08 51.26 47.08 46.26 46.22 25.97 24.15 21.61 21.39
O
N
MeO2C
NHTs
Me
3m
f1 (ppm)

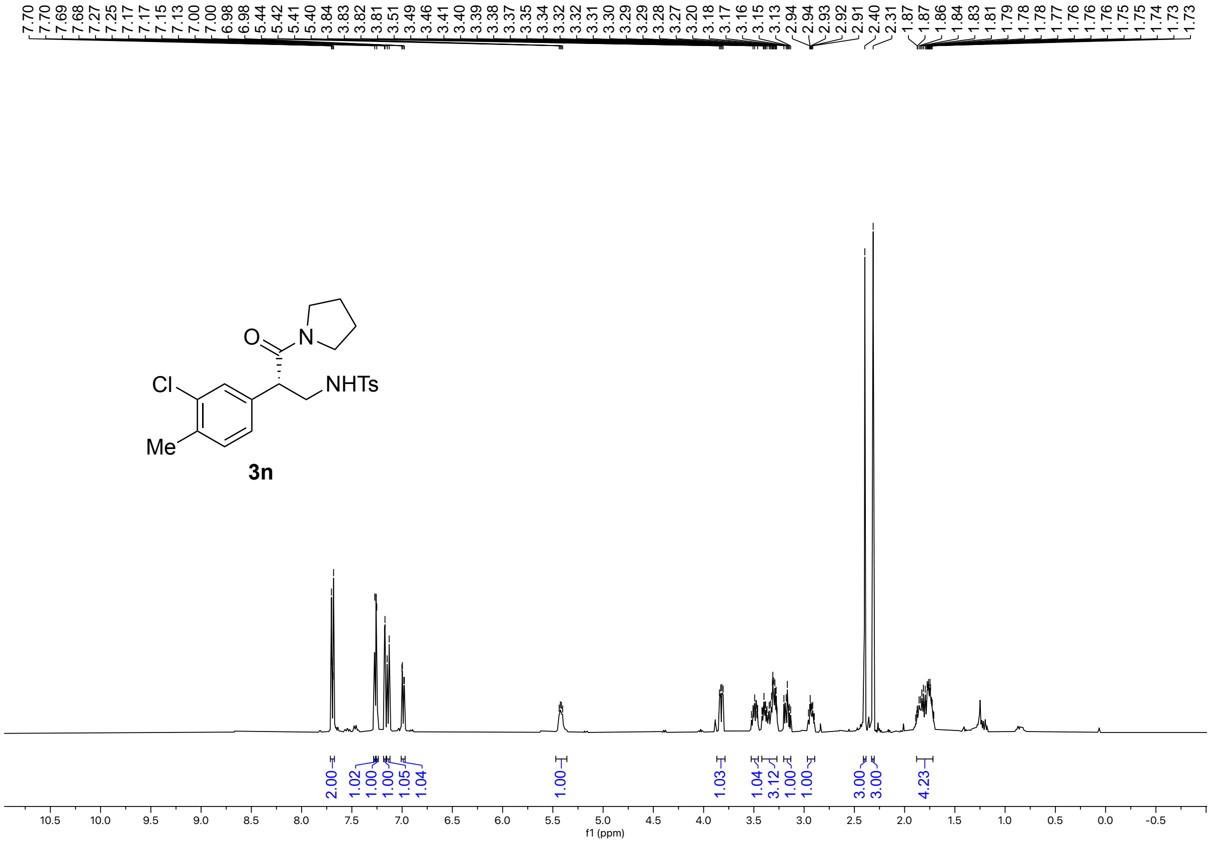
7.70
7.70
7.69
7.68
7.27
7.25
7.17
7.15
7.13
7.00
7.00
6.98
5.44
5.42
5.41
5.40
3.84
3.83
3.82
3.81
3.51
3.49
3.46
3.41
3.40
3.39
3.38
3.37
3.35
3.34
3.32
3.32
3.31
3.30
3.29
3.29
3.28
3.27
3.20
3.18
3.17
3.16
3.15
3.13
2.94
2.94
2.93
2.92
2.92
2.91
2.40
2.31
1.87
1.87
1.86
1.84
1.83
1.81
1.79
1.78
1.77
1.76
1.76
1.75
1.75
1.74
1.73
1.73
O
N
Cl
NHTs
Me
3n
2.00
1.02
1.00
1.00
1.05
1.04
1.00
1.03
1.04
3.12
1.00
1.00
3.00
3.00
4.23
f1 (ppm)

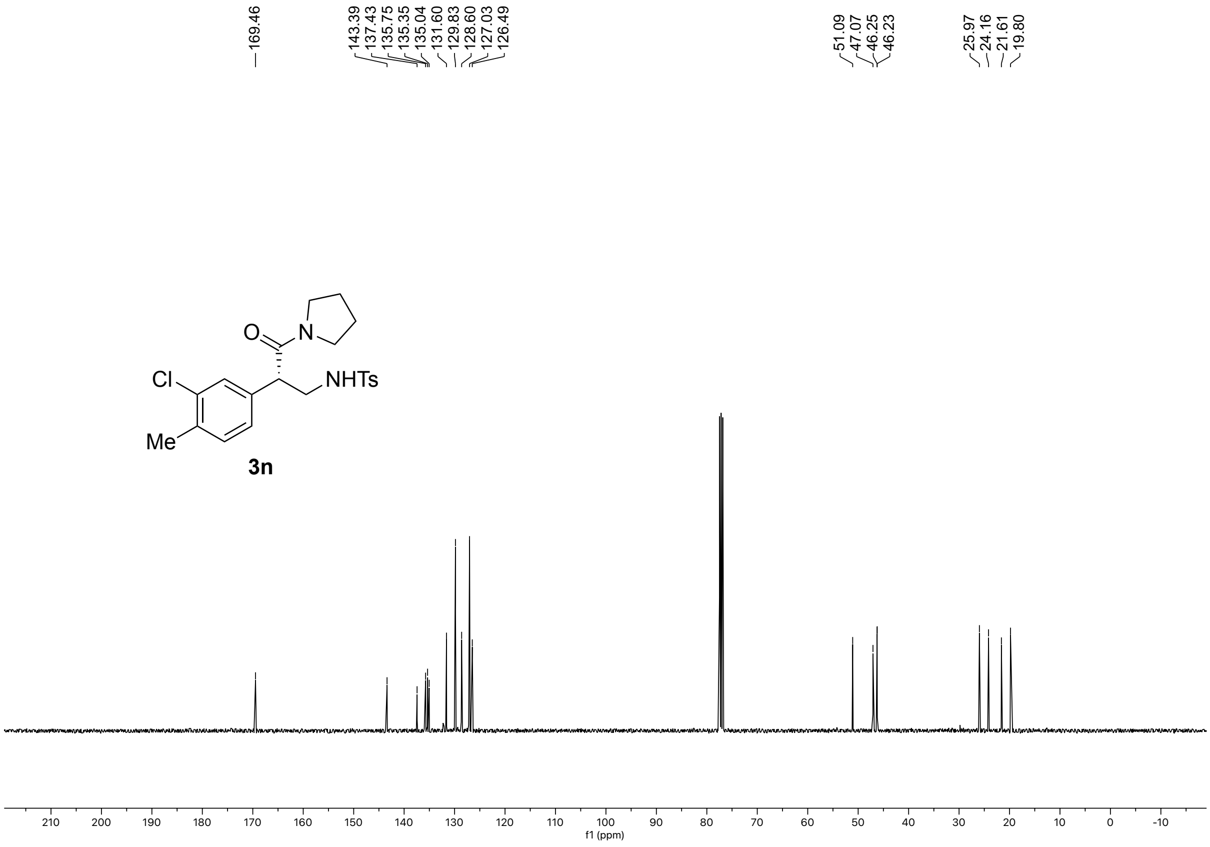
169.46
143.39
137.43
135.75
135.35
135.04
131.60
129.83
128.60
127.03
126.49
51.09
47.07
46.25
46.23
25.97
24.16
21.61
19.80
O
N
Cl
NHTs
Me
3n
f1 (ppm)

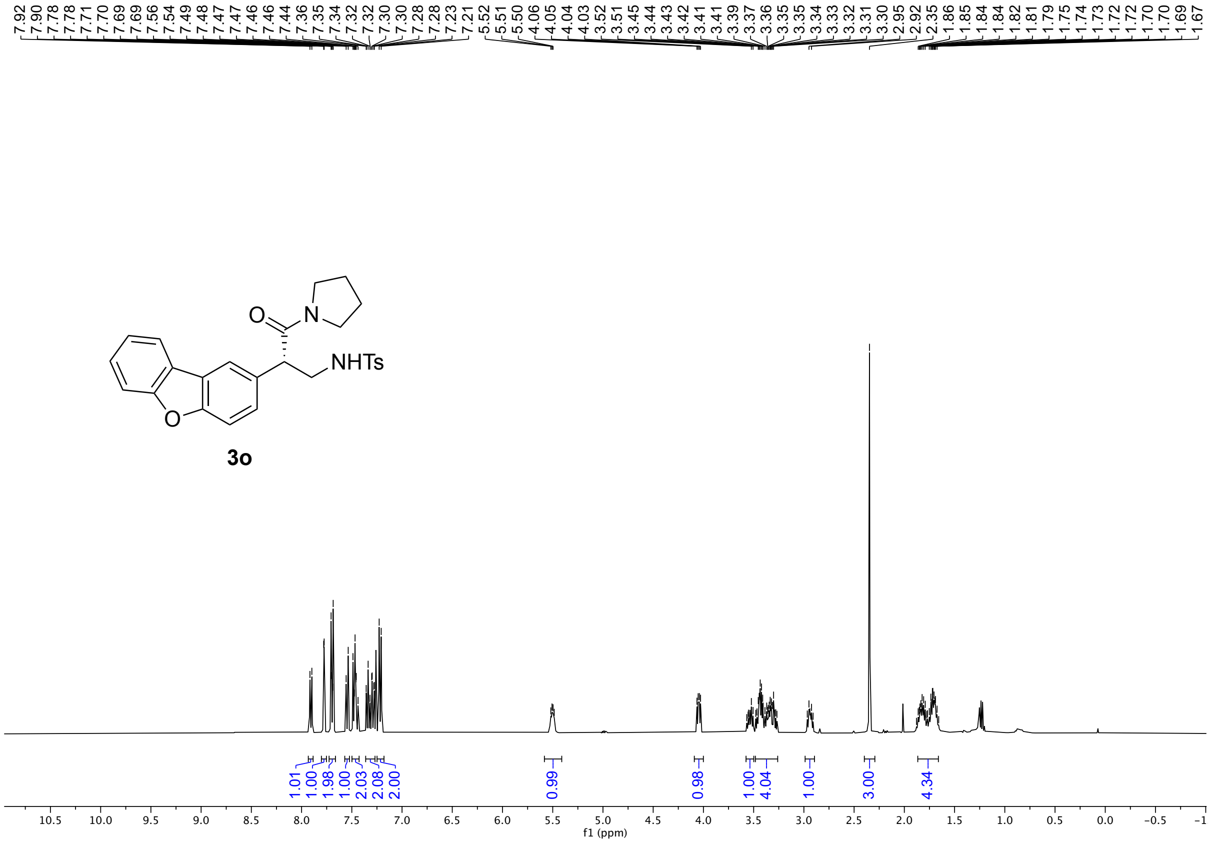
7.92 7.90 7.78 7.78 7.71 7.70 7.69 7.56 7.54 7.49 7.48 7.47 7.46 7.46 7.44 7.36 7.35 7.34 7.32 7.32 7.30 7.28 7.23 7.21 5.52 5.51 5.50 4.06 4.05 4.04 4.03 3.52 3.51 3.45 3.44 3.43 3.42 3.41 3.41 3.39 3.37 3.36 3.35 3.35 3.34 3.33 3.32 3.31 3.30 2.95 2.92 2.35 1.86 1.85 1.84 1.84 1.82 1.81 1.79 1.75 1.74 1.73 1.72 1.72 1.70 1.69 1.67
O
N
NHTs
O
3o
1.01 1.00 1.98 1.00 2.03 2.08 2.00 0.99 0.98 1.00 4.04 1.00 3.00 4.34
f1 (ppm)

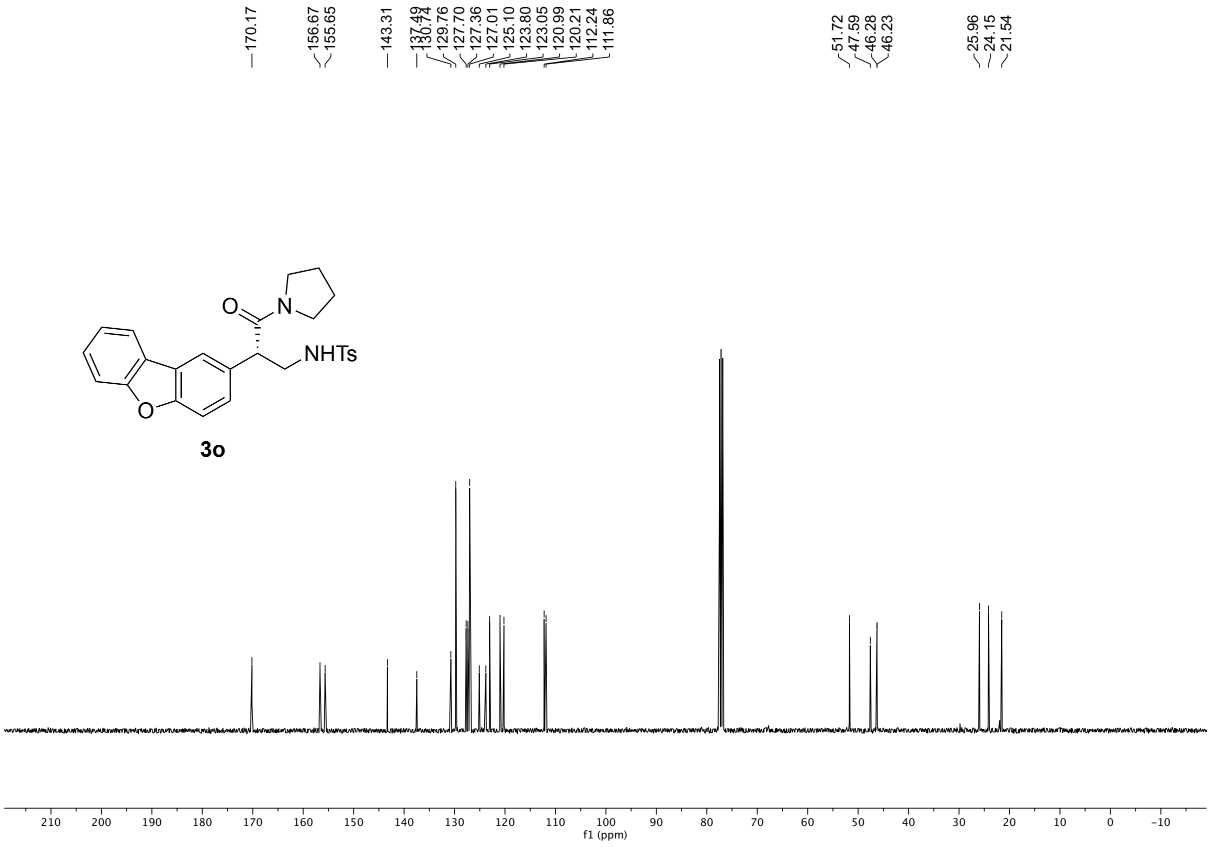
170.17 156.67 155.65 143.31 137.49 130.74 129.76 127.70 127.36 127.01 125.10 123.80 123.05 120.99 120.21 112.24 111.86 51.72 47.59 46.28 46.23 25.96 24.15 21.54
O
N
NHTs
O
3o
f1 (ppm)

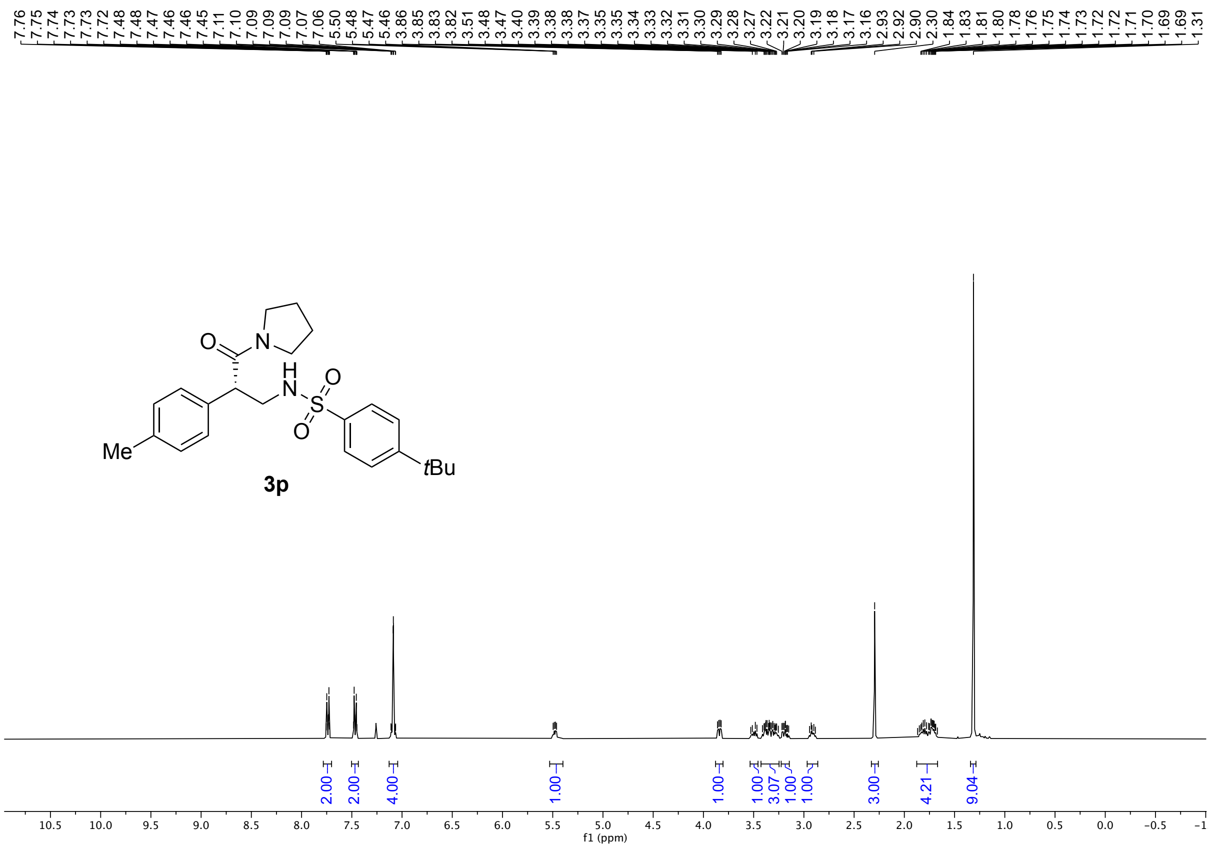
7.76 7.75 7.74 7.73 7.73 7.72 7.48 7.48 7.47 7.46 7.46 7.45 7.11 7.10 7.09 7.09 7.09 7.07 7.06 5.50 5.48 5.47 5.46 3.86 3.85 3.83 3.82 3.51 3.48 3.47 3.40 3.39 3.38 3.38 3.37 3.35 3.35 3.34 3.33 3.32 3.31 3.30 3.29 3.28 3.27 3.22 3.21 3.20 3.19 3.18 3.17 3.16 2.93 2.92 2.90 2.30 1.84 1.83 1.81 1.80 1.78 1.76 1.75 1.74 1.73 1.72 1.72 1.71 1.70 1.69 1.69 1.31
O
N
H
N
S
O
O
Me
tBu
3p
2.00
2.00
4.00
1.00
1.00
1.00
3.07
1.00
1.00
3.00
4.21
9.04
10.5 10.0 9.5 9.0 8.5 8.0 7.5 7.0 6.5 6.0 5.5 5.0 4.5 4.0 3.5 3.0 2.5 2.0 1.5 1.0 0.5 0.0 −0.5 −1
f1 (ppm)

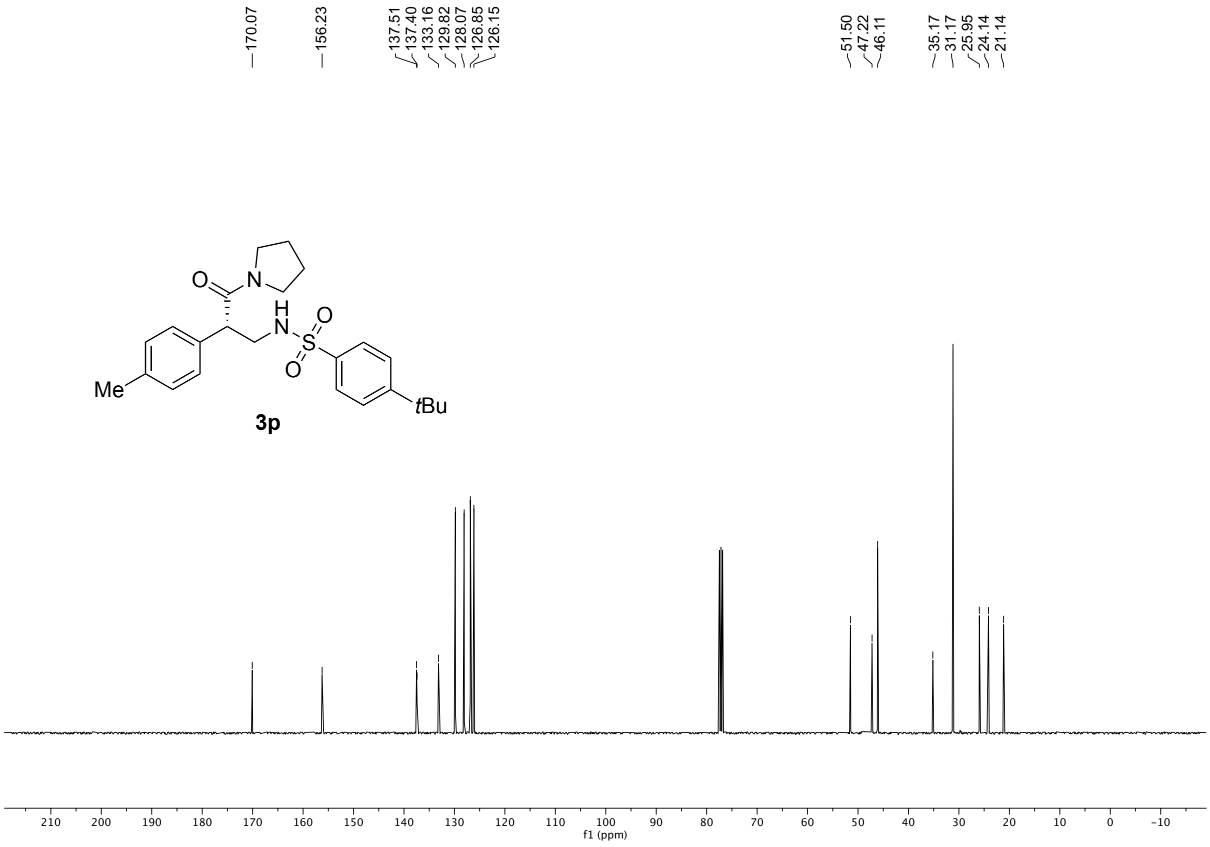
170.07
156.23
137.51 137.40 133.16 129.82 128.07 126.85 126.15
51.50 47.22 46.11
35.17 31.17 25.95 24.14 21.14
O
N
H
N
S
O
O
Me
tBu
3p
210 200 190 180 170 160 150 140 130 120 110 100 90 80 70 60 50 40 30 20 10 0 −10
f1 (ppm)

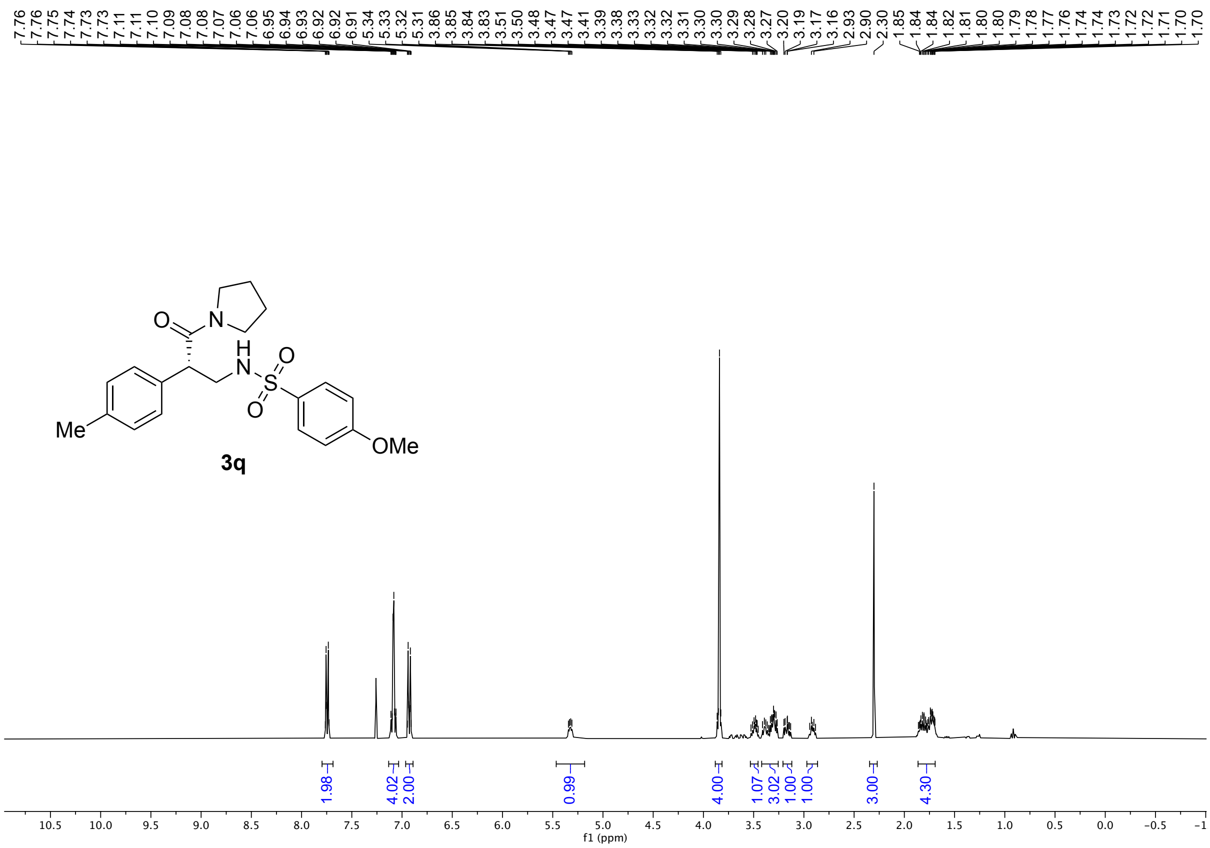
7.76 7.76 7.75 7.74 7.73 7.73 7.11 7.11 7.10 7.09 7.08 7.08 7.07 7.06 7.06 6.95 6.94 6.93 6.92 6.92 6.91 5.34 5.33 5.32 5.31 3.86 3.85 3.84 3.83 3.51 3.50 3.48 3.47 3.47 3.41 3.39 3.38 3.33 3.32 3.32 3.31 3.30 3.30 3.29 3.28 3.27 3.20 3.19 3.17 3.16 2.93 2.90 2.30 1.85 1.84 1.84 1.82 1.81 1.80 1.80 1.79 1.78 1.77 1.76 1.74 1.74 1.73 1.72 1.71 1.70 1.70
Me
OMe
3q
1.98
4.02
2.00
0.99
4.00
1.07
3.02
1.00
1.00
3.00
4.30
f1 (ppm)

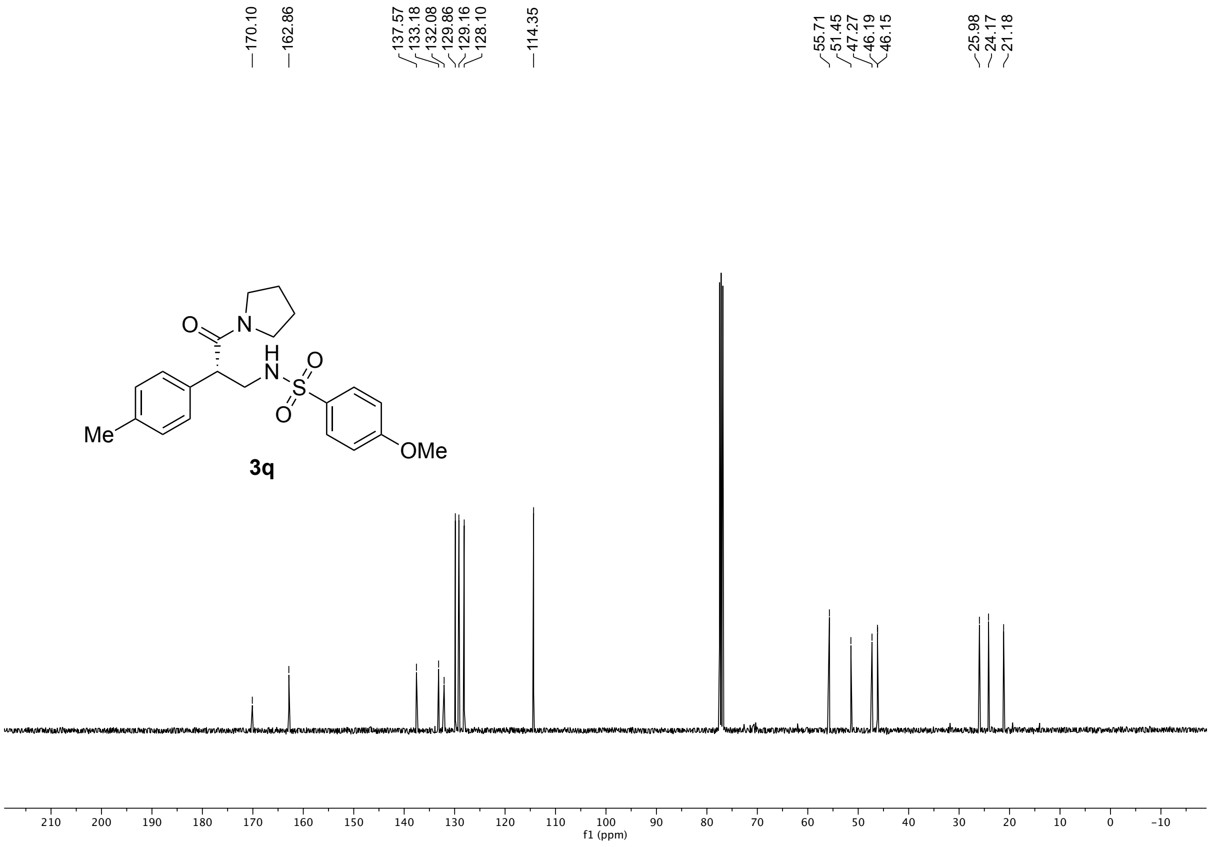
170.10 162.86 137.57 133.18 132.08 129.86 129.16 128.10 114.35 55.71 51.45 47.27 46.19 46.15 25.98 24.17 21.18
Me
OMe
3q
f1 (ppm)

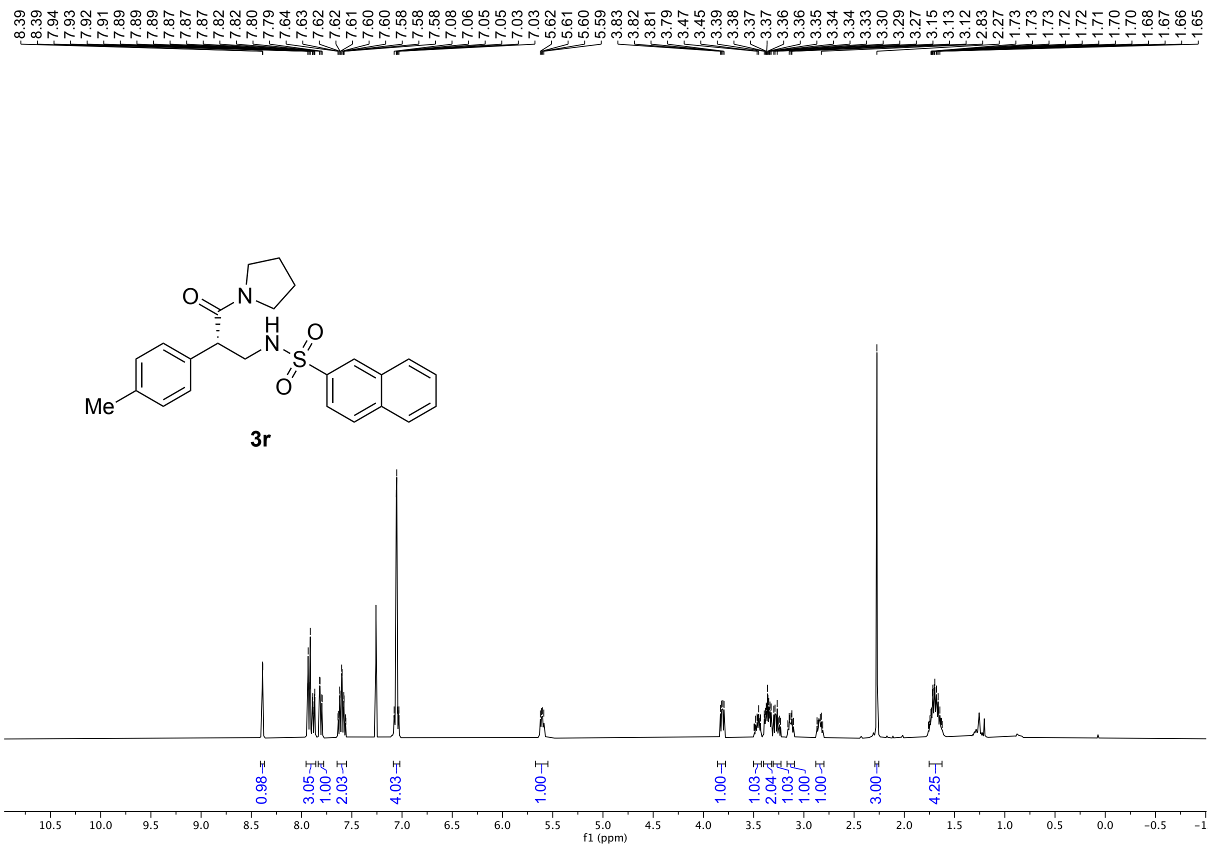
8.39 8.39 7.94 7.93 7.92 7.91 7.89 7.89 7.87 7.87 7.82 7.80 7.79 7.64 7.63 7.62 7.62 7.61 7.60 7.58 7.58 7.58 7.08 7.06 7.05 7.05 7.03 5.62 5.61 5.60 5.59 3.83 3.82 3.81 3.79 3.47 3.45 3.39 3.38 3.37 3.37 3.36 3.36 3.35 3.34 3.34 3.33 3.30 3.29 3.27 3.15 3.13 3.12 2.83 2.27 1.73 1.73 1.73 1.73 1.72 1.72 1.71 1.70 1.70 1.68 1.67 1.66 1.65
O
N
H
N
S
O
O
Me
0.98 3.05 1.00 2.03 4.03 1.00 1.00 1.03 2.04 1.03 1.00 1.00 3.00 4.25
10.5 10.0 9.5 9.0 8.5 8.0 7.5 7.0 6.5 6.0 5.5 5.0 4.5 4.0 3.5 3.0 2.5 2.0 1.5 1.0 0.5 0.0 −0.5 −1
f1 (ppm)

**3r**

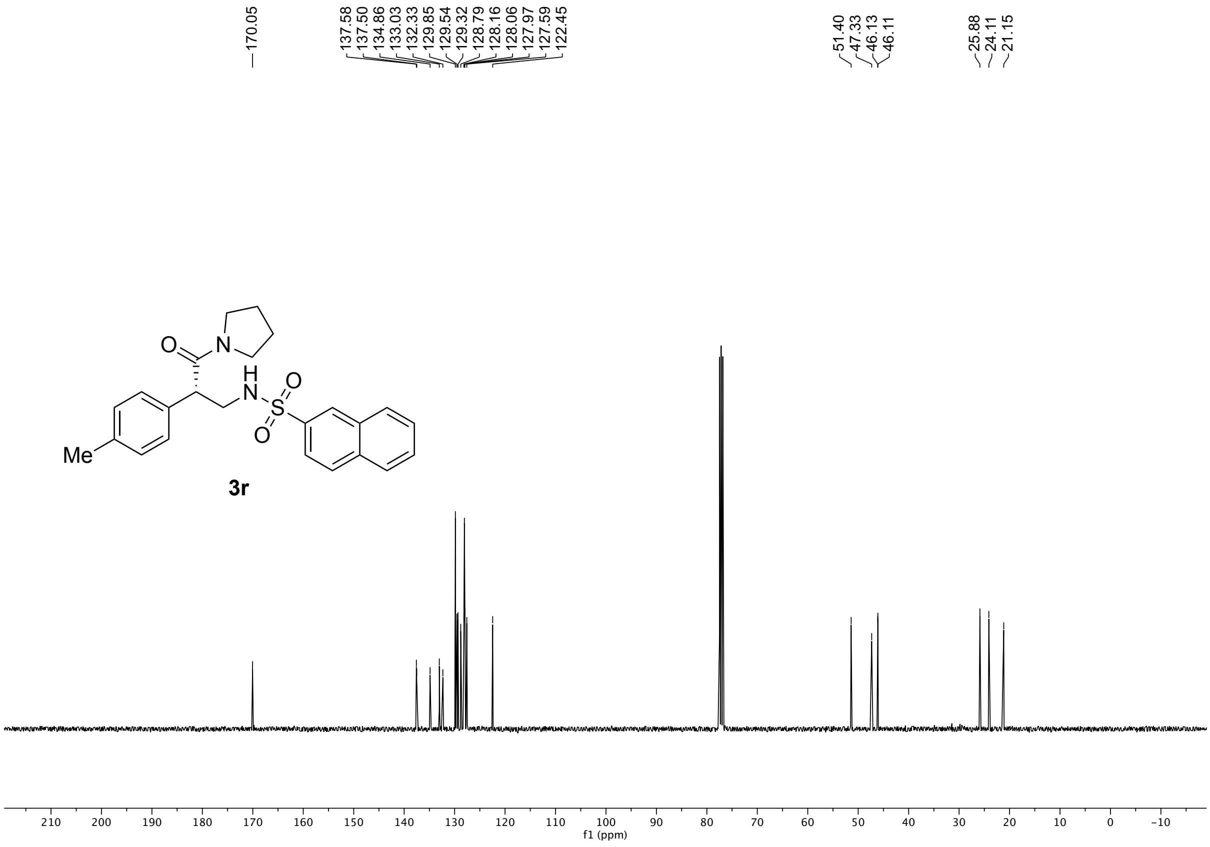
170.05
137.58 137.50 134.86 133.03 132.33 129.85 129.54 129.32 128.79 128.16 128.06 127.97 127.59 122.45
51.40 47.33 46.13 46.11
25.88 24.11 21.15
O
N
H
N
S
O
O
Me
210 200 190 180 170 160 150 140 130 120 110 100 90 80 70 60 50 40 30 20 10 0 −10
f1 (ppm)

**3r**

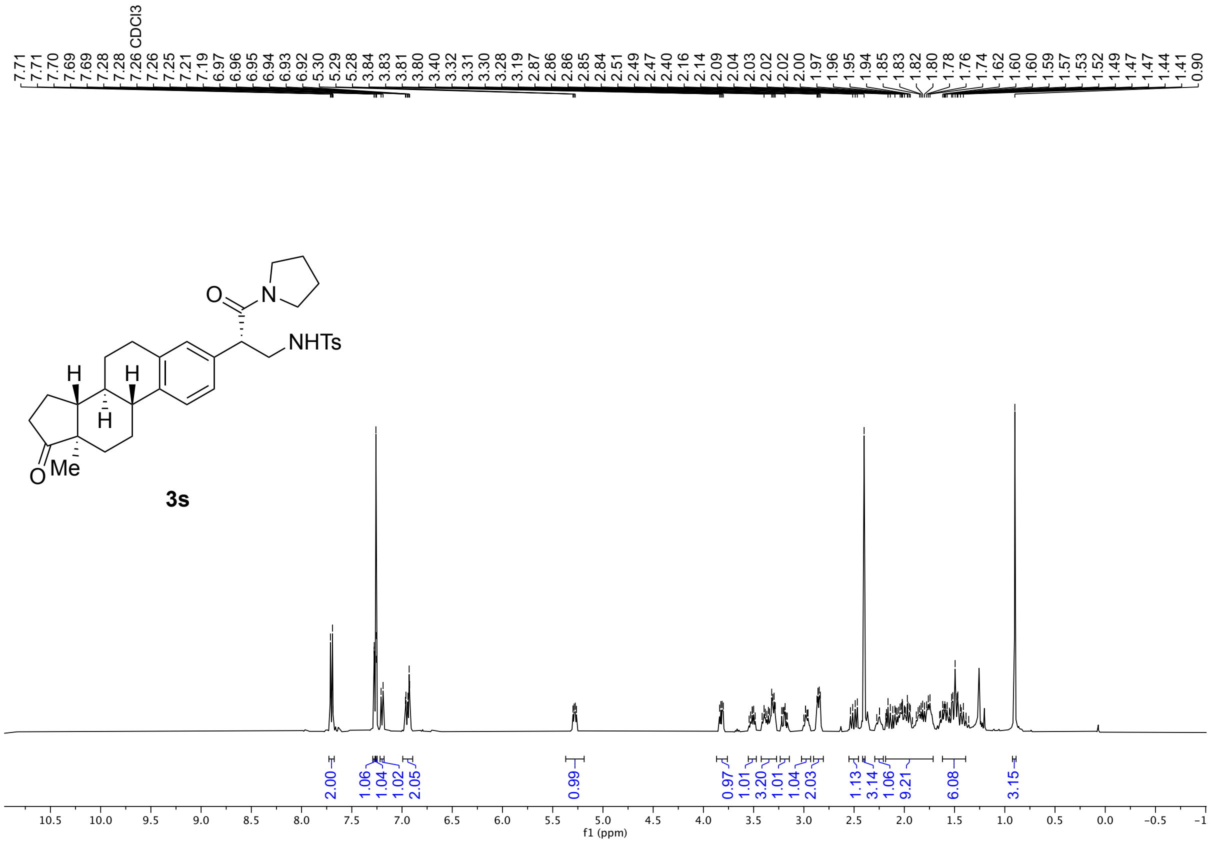
7.71 7.71 7.70 7.69 7.69 7.28 7.28 7.26 CDCl3 7.26 7.25 7.21 7.19 6.97 6.96 6.95 6.94 6.93 6.92 5.30 5.29 5.28 3.84 3.83 3.81 3.80 3.40 3.32 3.31 3.30 3.28 3.19 2.87 2.86 2.86 2.85 2.84 2.51 2.49 2.47 2.40 2.16 2.14 2.09 2.04 2.03 2.02 2.02 2.00 1.97 1.96 1.95 1.94 1.85 1.83 1.82 1.80 1.78 1.76 1.74 1.62 1.60 1.59 1.57 1.53 1.52 1.49 1.47 1.44 1.41 0.90
O
N
NHTs
H
H
H
O
Me
3s
2.00 1.06 1.04 1.02 2.05 0.99 0.97 1.01 3.20 1.01 1.04 2.03 1.13 3.14 1.06 9.21 6.08 3.15
f1 (ppm)

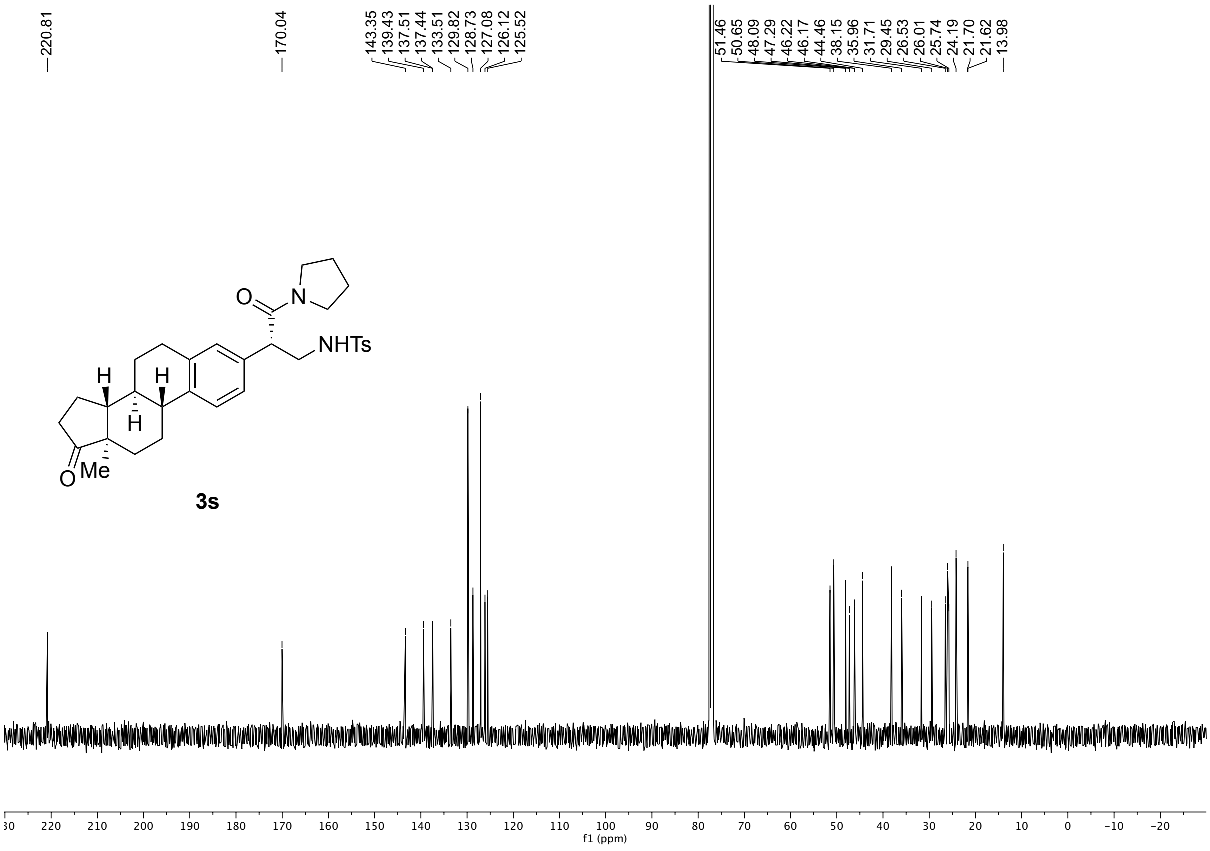
220.81
170.04
143.35 139.43 137.51 137.44 133.51 129.82 128.73 127.08 126.12 125.52
51.46 50.65 48.09 47.29 46.22 46.17 44.46 38.15 35.96 31.71 29.45 26.53 26.01 25.74 24.19 21.70 21.62 13.98
O
N
NHTs
H
H
H
O
Me
3s
f1 (ppm)

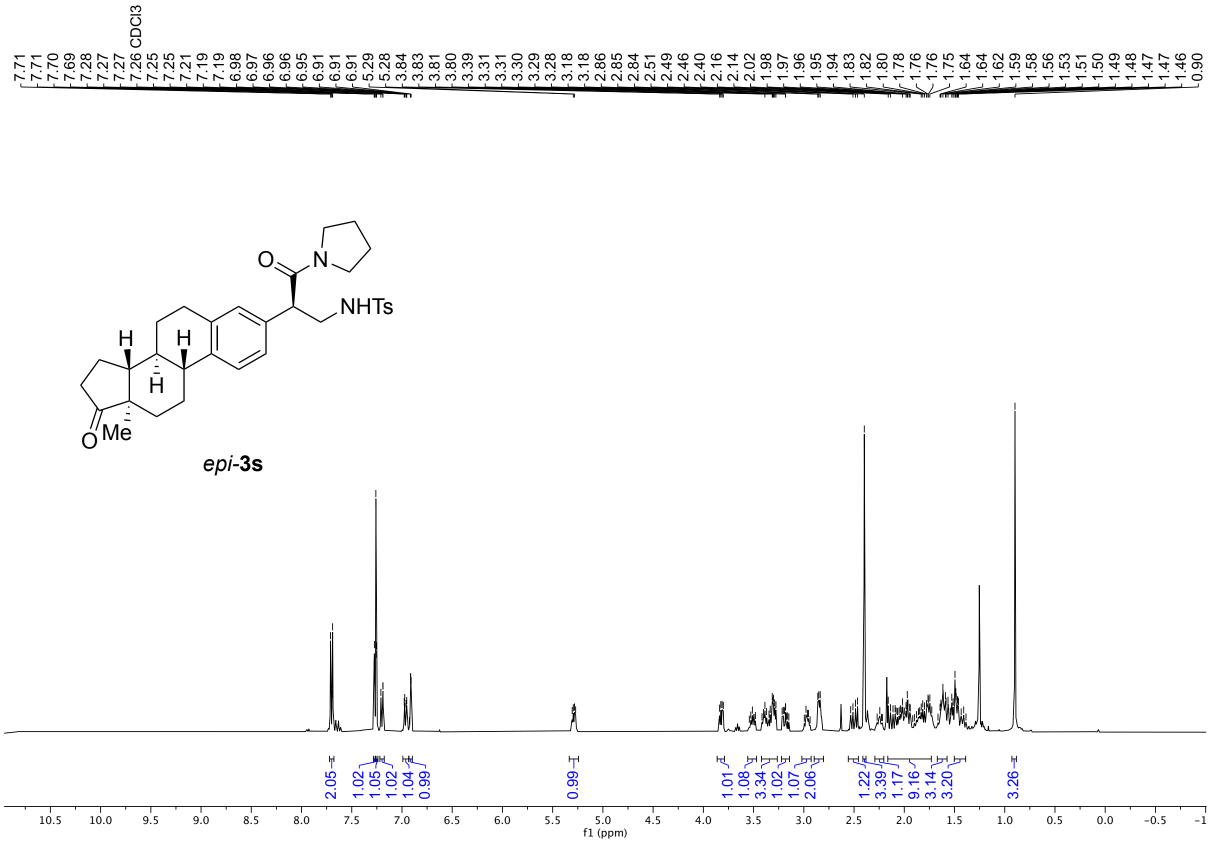
epi-3s
f1 (ppm)

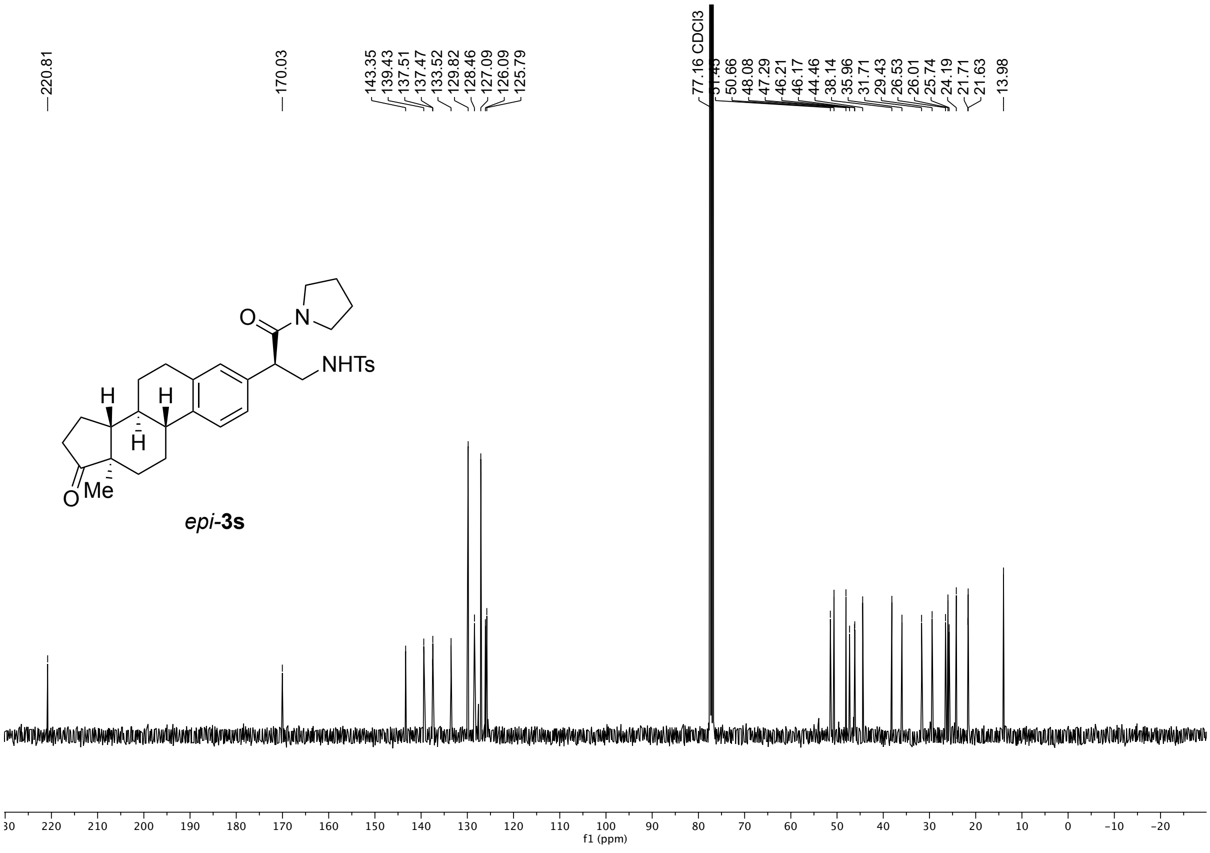
epi-3s
f1 (ppm)

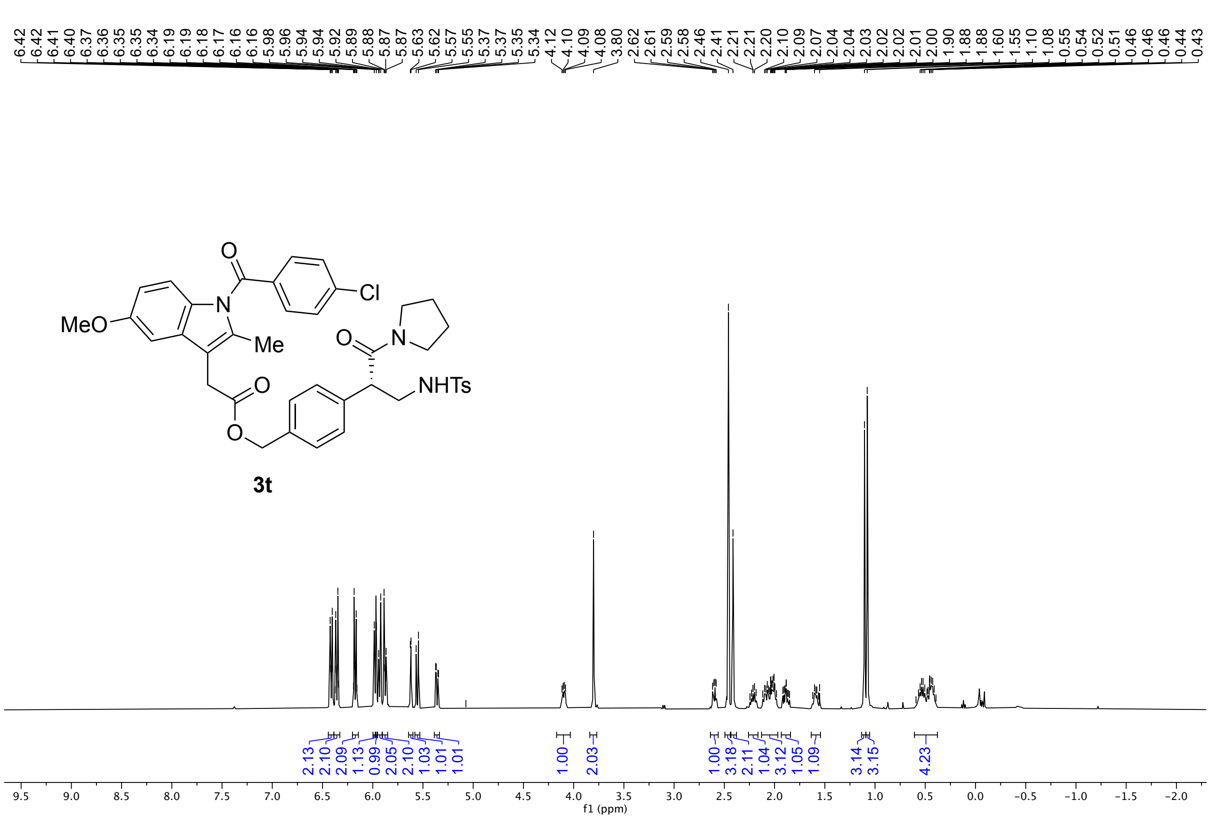
6.42 6.42 6.41 6.40 6.37 6.36 6.35 6.35 6.34 6.19 6.19 6.18 6.17 6.16 6.16 5.98 5.96 5.94 5.94 5.92 5.89 5.88 5.87 5.63 5.62 5.57 5.55 5.37 5.37 5.35 5.34 4.12 4.10 4.09 4.08 3.80 2.62 2.61 2.59 2.58 2.46 2.41 2.21 2.21 2.20 2.10 2.09 2.07 2.04 2.04 2.03 2.02 2.02 2.01 2.00 1.90 1.88 1.60 1.55 1.10 1.08 0.55 0.54 0.52 0.51 0.46 0.46 0.44 0.43
MeO
Me
NHTs
Cl
3t
2.13 2.10 2.09 1.13 0.99 2.05 2.10 1.03 1.01 1.01 1.00 2.03 1.00 3.18 2.11 1.04 3.12 1.05 1.09 3.14 3.15 4.23
f1 (ppm)

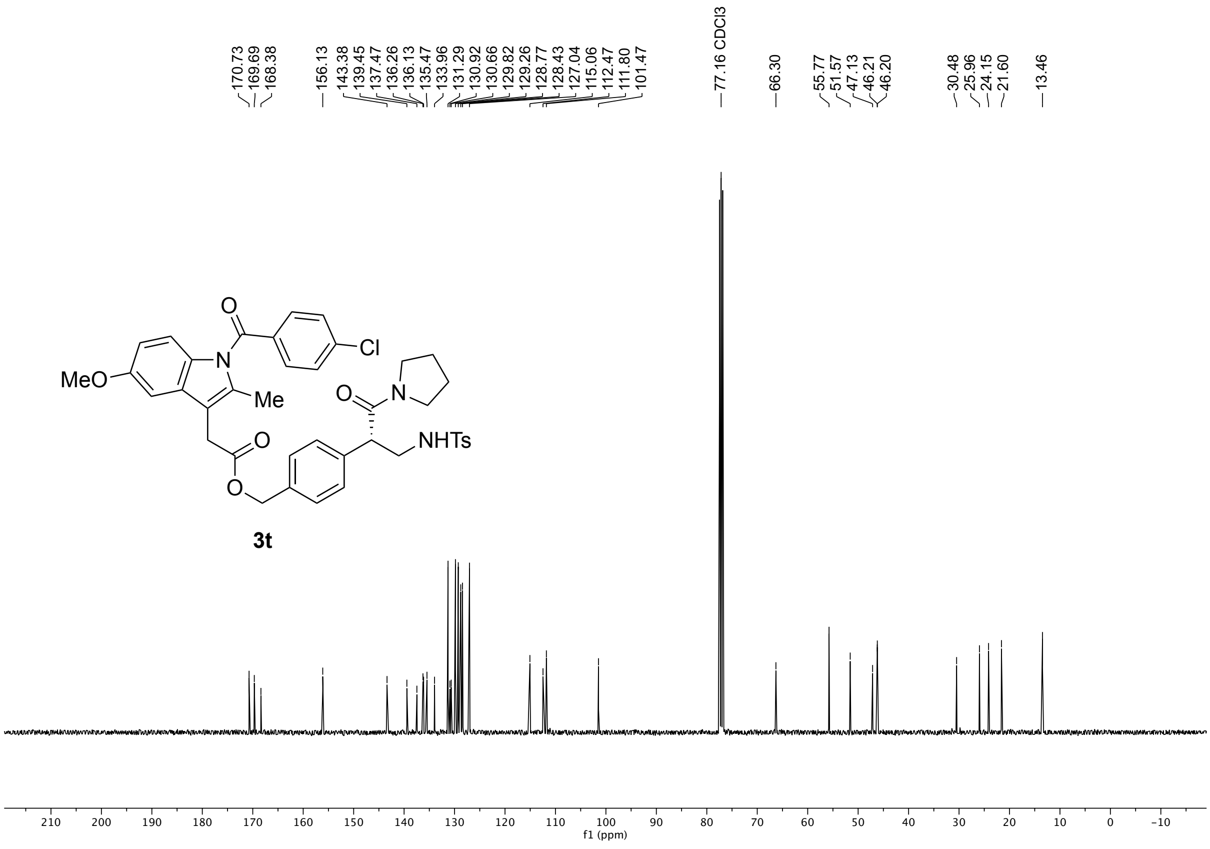
170.73 169.69 168.38 156.13 143.38 139.45 137.47 136.26 136.13 135.47 133.96 131.29 130.92 130.66 129.82 129.26 128.77 128.43 127.04 115.06 112.47 111.80 101.47
77.16 CDCl3
66.30 55.77 51.57 47.13 46.21 46.20 30.48 25.96 24.15 21.60 13.46
MeO
Me
NHTs
Cl
3t
f1 (ppm)

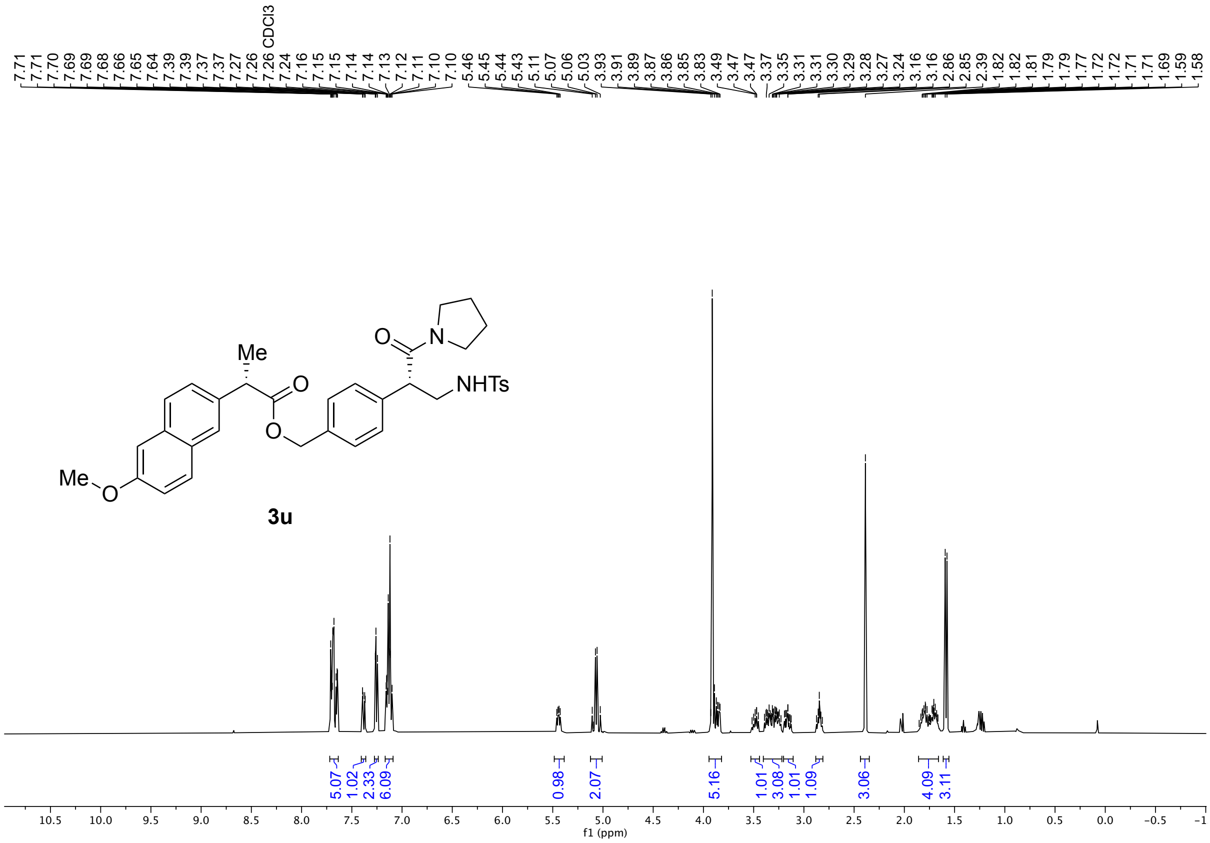
7.71
7.71
7.70
7.69
7.69
7.68
7.66
7.65
7.64
7.39
7.39
7.37
7.37
7.27
7.26 CDCl3
7.24
7.16
7.15
7.15
7.14
7.14
7.13
7.12
7.11
7.10
5.46
5.45
5.44
5.43
5.11
5.07
5.06
5.03
3.93
3.91
3.89
3.87
3.86
3.85
3.83
3.49
3.47
3.37
3.35
3.31
3.31
3.30
3.29
3.28
3.27
3.24
3.16
3.16
2.86
2.85
2.39
1.82
1.82
1.81
1.79
1.77
1.72
1.72
1.71
1.69
1.59
1.58
Me
O
N
O
NHTs
Me
O
3u
5.07
1.02
2.33
6.09
0.98
2.07
5.16
1.01
3.08
1.01
1.09
3.06
4.09
3.11
10.5 10.0 9.5 9.0 8.5 8.0 7.5 7.0 6.5 6.0 5.5 5.0 4.5 4.0 3.5 3.0 2.5 2.0 1.5 1.0 0.5 0.0 −0.5 −1
f1 (ppm)

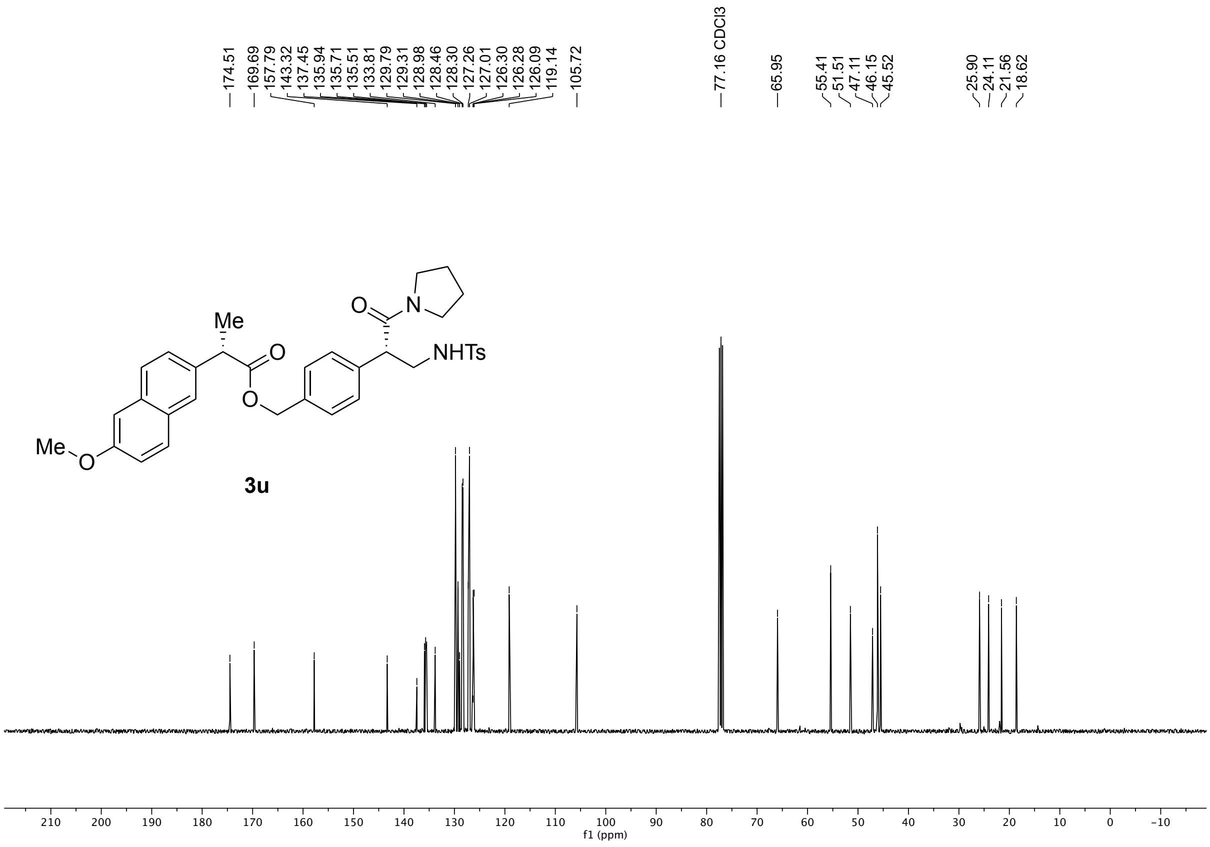
174.51
169.69
157.79
143.32
137.45
135.94
135.71
135.51
133.81
129.79
129.31
128.98
128.46
128.30
127.26
127.01
126.30
126.28
126.09
119.14
105.72
77.16 CDCl3
65.95
55.41
51.51
47.11
46.15
45.52
25.90
24.11
21.56
18.62
Me
O
N
O
NHTs
Me
O
3u
210 200 190 180 170 160 150 140 130 120 110 100 90 80 70 60 50 40 30 20 10 0 −10
f1 (ppm)

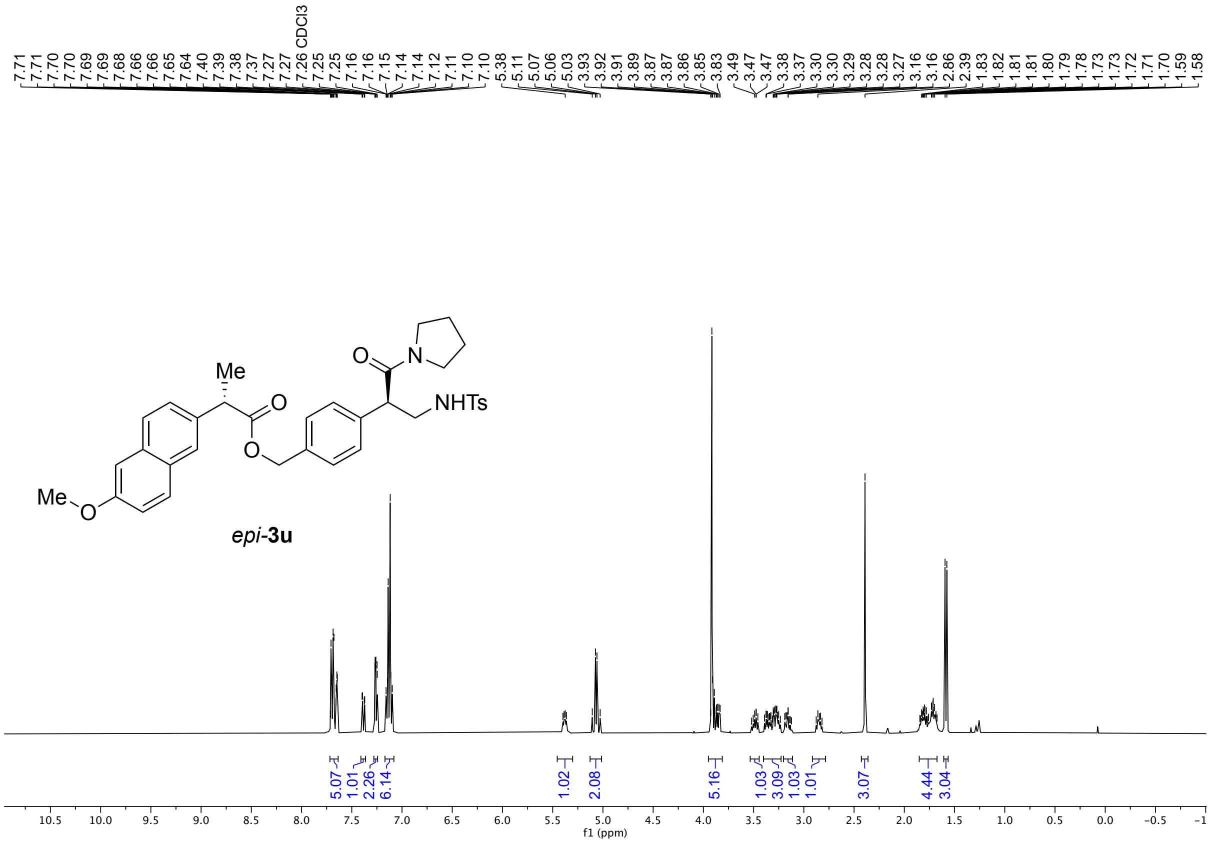

epi-3u
f1 (ppm)


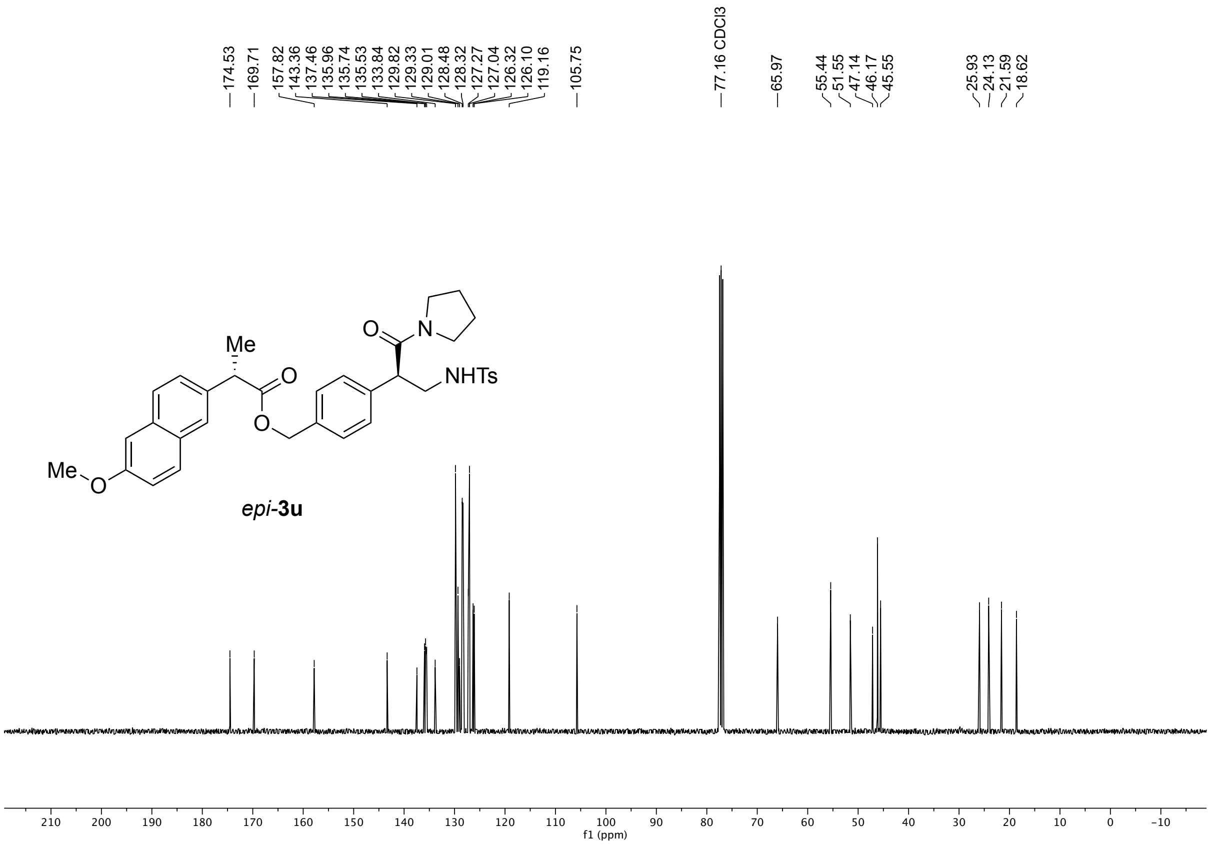

epi-3u
f1 (ppm)

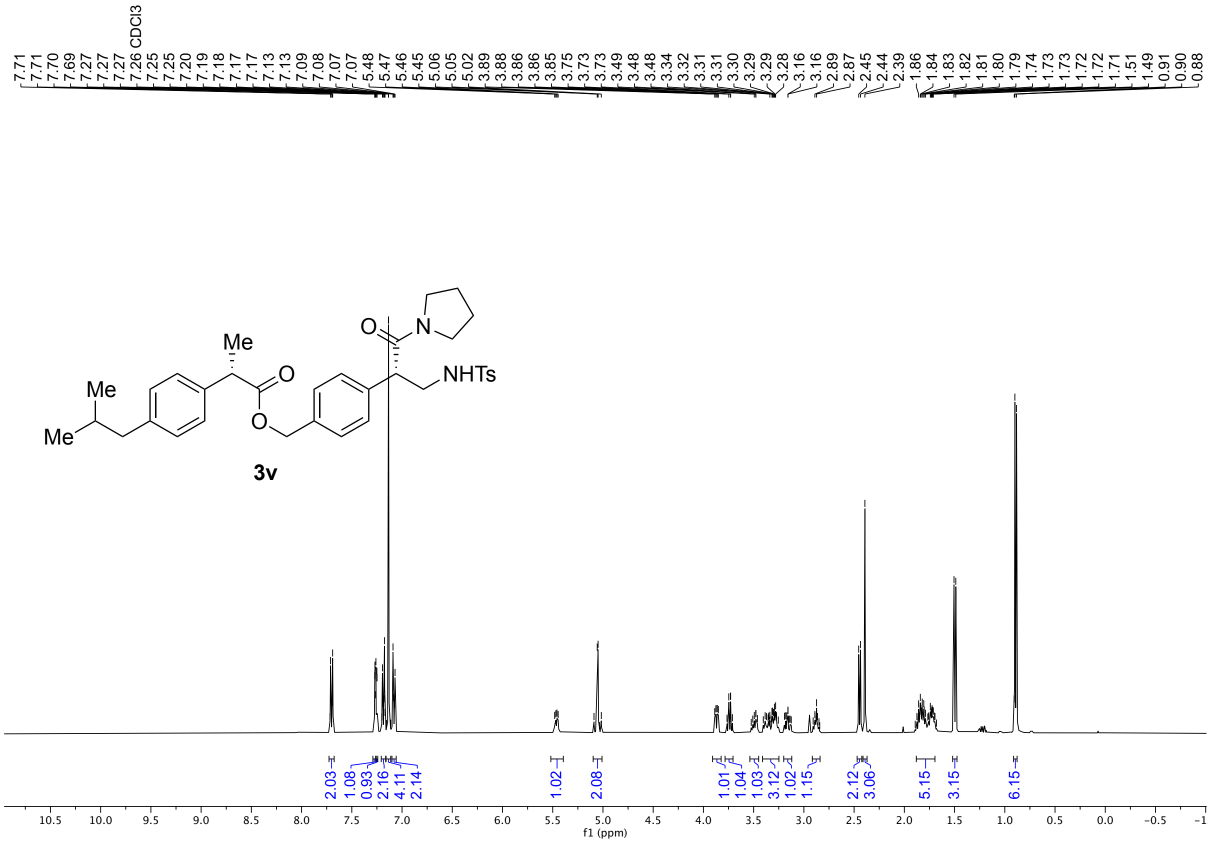
7.71 7.71 7.70 7.69 7.27 7.27 7.27 7.26 CDCl3 7.25 7.25 7.20 7.19 7.18 7.17 7.17 7.13 7.13 7.09 7.08 7.07 7.07 5.48 5.47 5.46 5.45 5.06 5.05 5.02 3.89 3.88 3.86 3.85 3.75 3.73 3.73 3.49 3.48 3.34 3.32 3.31 3.31 3.30 3.29 3.29 3.28 3.16 3.16 2.89 2.87 2.45 2.44 2.39 1.86 1.84 1.83 1.82 1.81 1.80 1.79 1.74 1.73 1.72 1.72 1.71 1.51 1.49 0.91 0.90 0.88
Me
O
Me
O
Me
O
N
NHTs
3v
2.03 1.08 0.93 2.16 4.11 2.14 1.02 2.08 1.01 1.04 1.03 3.12 1.02 1.15 2.12 3.06 5.15 3.15 6.15
f1 (ppm)

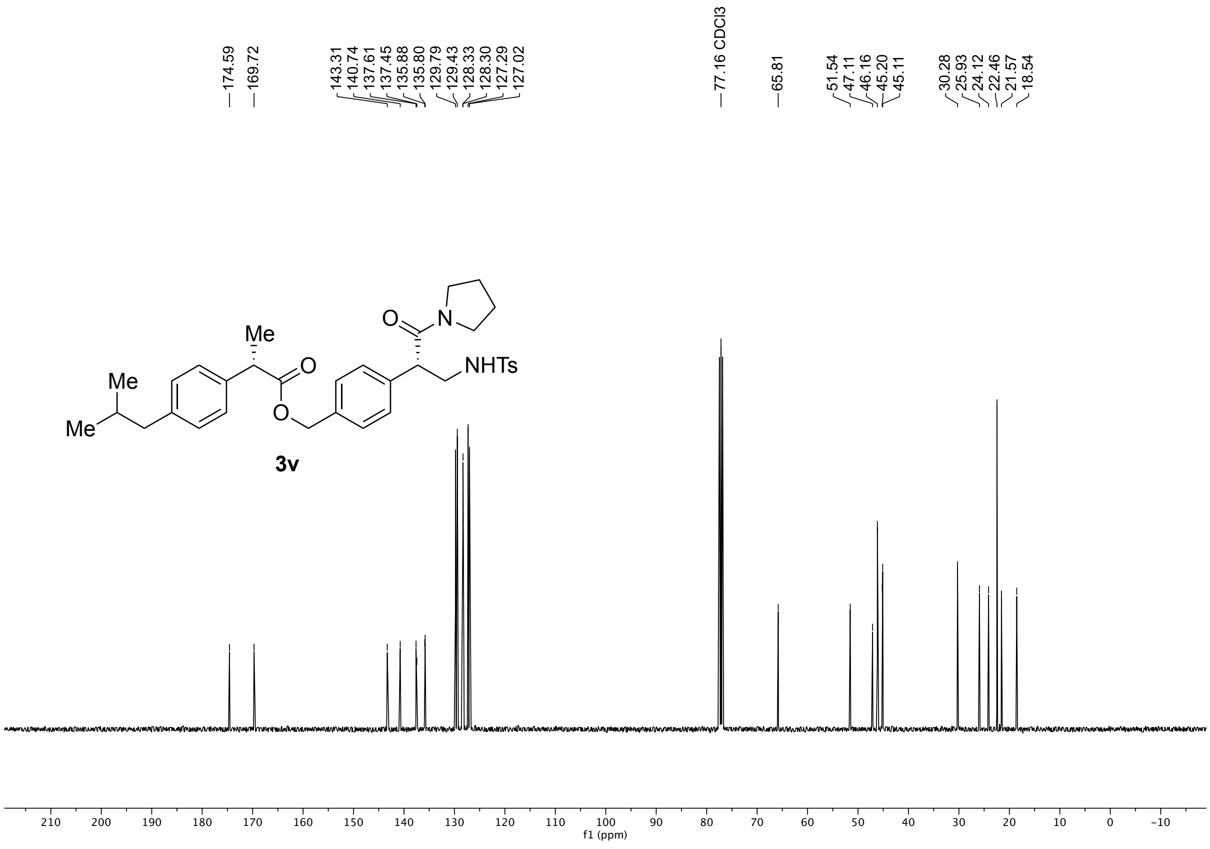
174.59 169.72 143.31 140.74 137.61 137.45 135.88 135.80 129.79 129.43 128.33 128.30 127.29 127.02 77.16 CDCl3 65.81 51.54 47.11 46.16 45.20 45.11 30.28 25.93 24.12 22.46 21.57 18.54
Me
O
Me
O
Me
O
N
NHTs
3v
f1 (ppm)

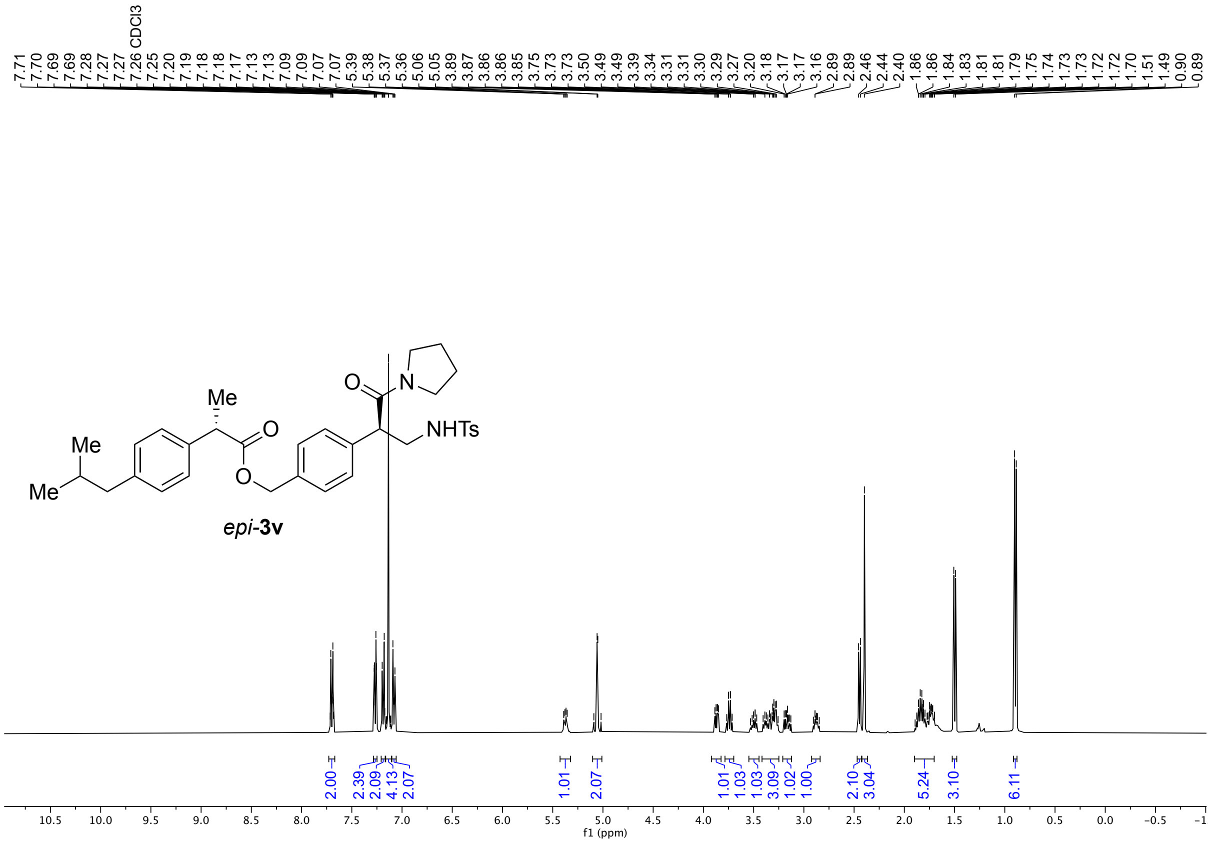

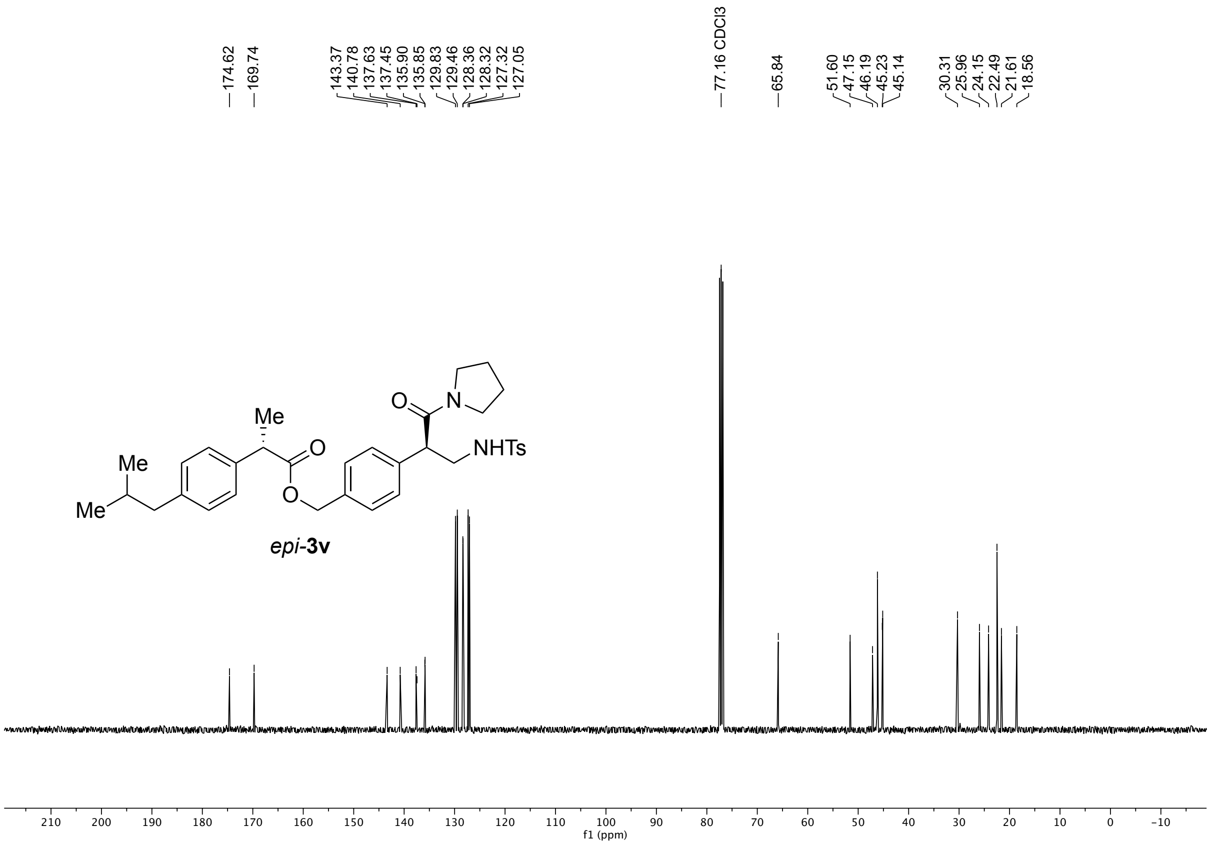

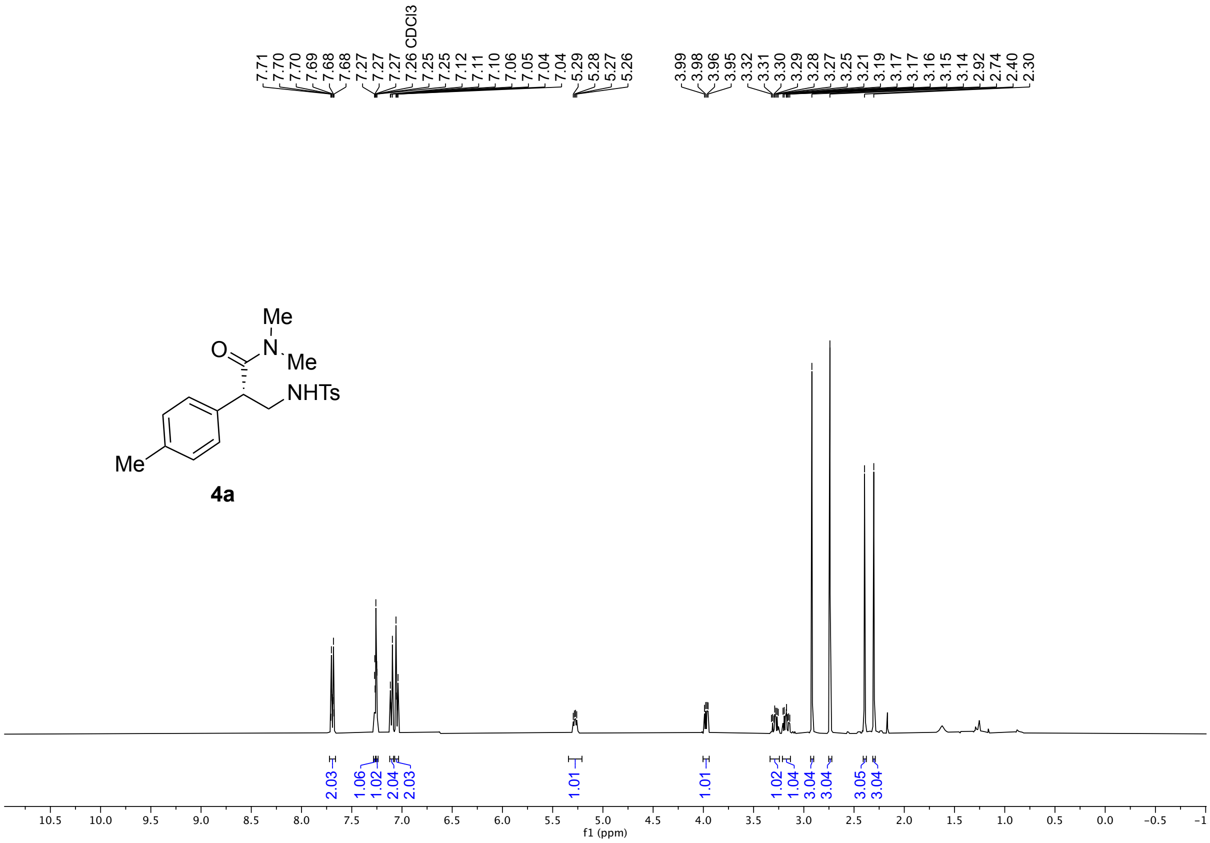
7.71
7.70
7.70
7.69
7.68
7.68
7.27
7.27
7.27
7.26 CDCl3
7.25
7.25
7.12
7.11
7.10
7.06
7.05
7.04
7.04
5.29
5.28
5.27
5.26
3.99
3.98
3.96
3.95
3.32
3.31
3.30
3.29
3.28
3.27
3.25
3.21
3.19
3.17
3.17
3.16
3.15
3.14
2.92
2.74
2.40
2.30
Me
O
N
Me
NHTs
Me
4a
2.03
1.06
1.02
2.04
2.03
1.01
1.01
1.02
1.04
3.04
3.04
3.05
3.04
f1 (ppm)

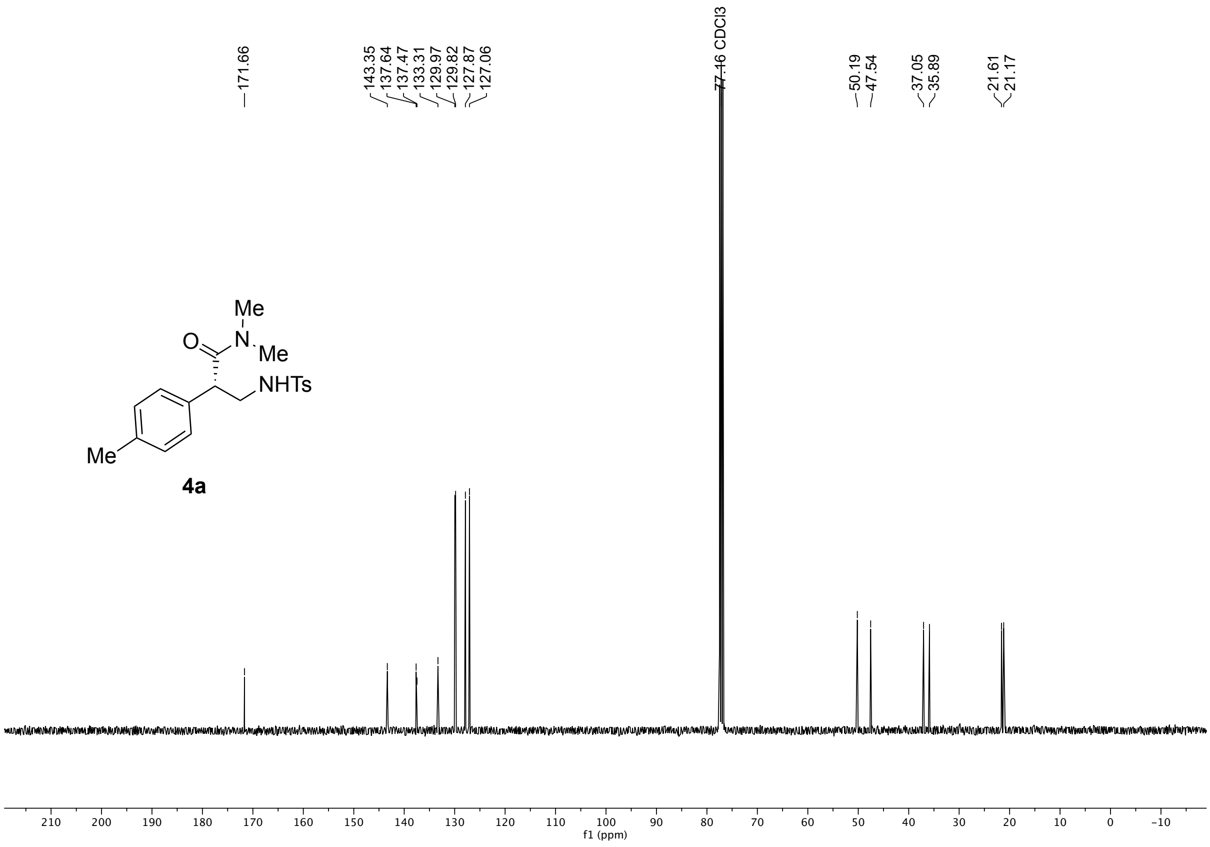
171.66
143.35
137.64
137.47
133.31
129.97
129.82
127.87
127.06
77.46 CDCl3
50.19
47.54
37.05
35.89
21.61
21.17
Me
O
N
Me
NHTs
Me
4a
f1 (ppm)

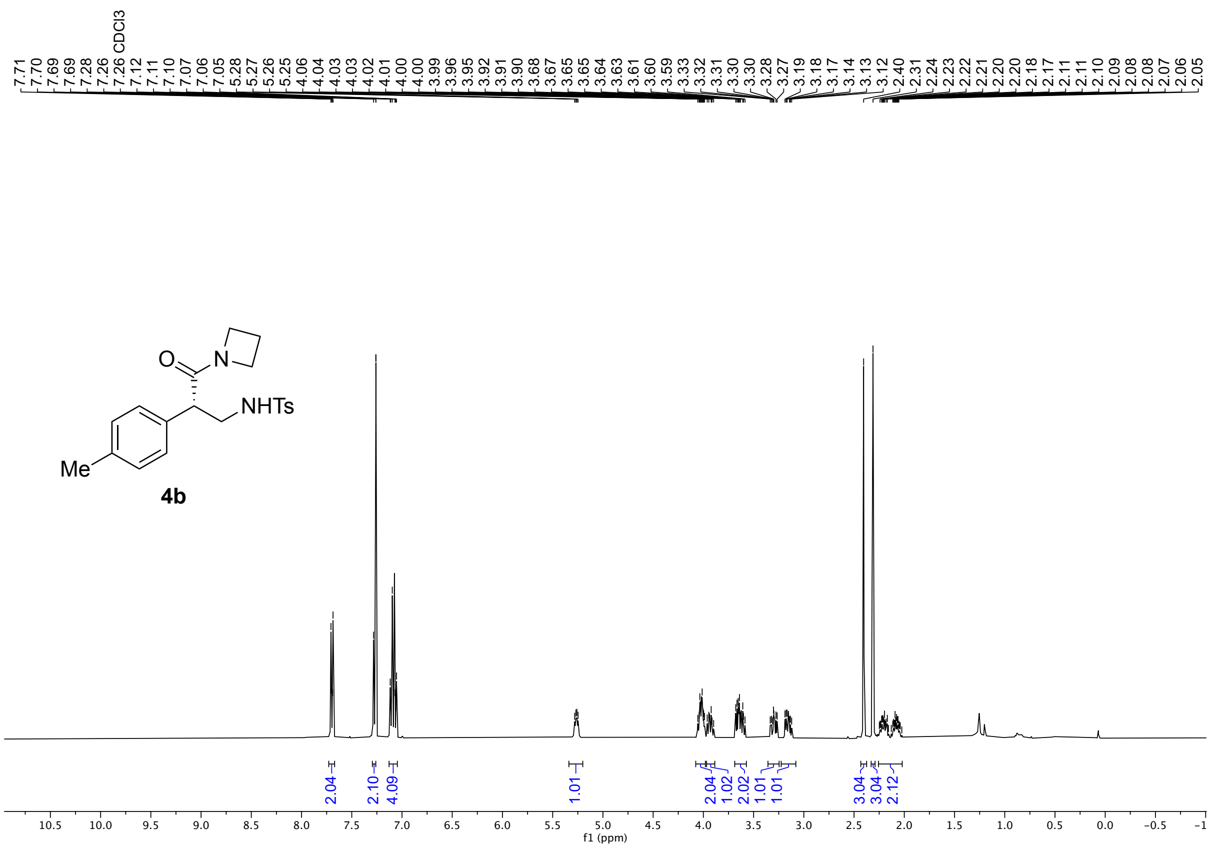
7.71
7.70
7.69
7.69
7.28
7.26 CDCl3
7.12
7.11
7.10
7.07
7.06
7.05
5.28
5.27
5.26
5.25
4.06
4.04
4.03
4.03
4.02
4.01
4.00
4.00
3.99
3.96
3.95
3.92
3.91
3.90
3.68
3.67
3.65
3.65
3.64
3.63
3.61
3.60
3.59
3.33
3.32
3.31
3.30
3.28
3.27
3.19
3.18
3.17
3.14
3.13
3.12
2.40
2.31
2.24
2.23
2.22
2.21
2.20
2.20
2.18
2.17
2.11
2.10
2.09
2.08
2.07
2.06
2.05
O
N
NHTs
Me
4b
2.04
2.10
4.09
1.01
2.04
1.02
2.02
1.01
1.01
3.04
3.04
2.12
10.5 10.0 9.5 9.0 8.5 8.0 7.5 7.0 6.5 6.0 5.5 5.0 4.5 4.0 3.5 3.0 2.5 2.0 1.5 1.0 0.5 0.0 -0.5 -1
f1 (ppm)

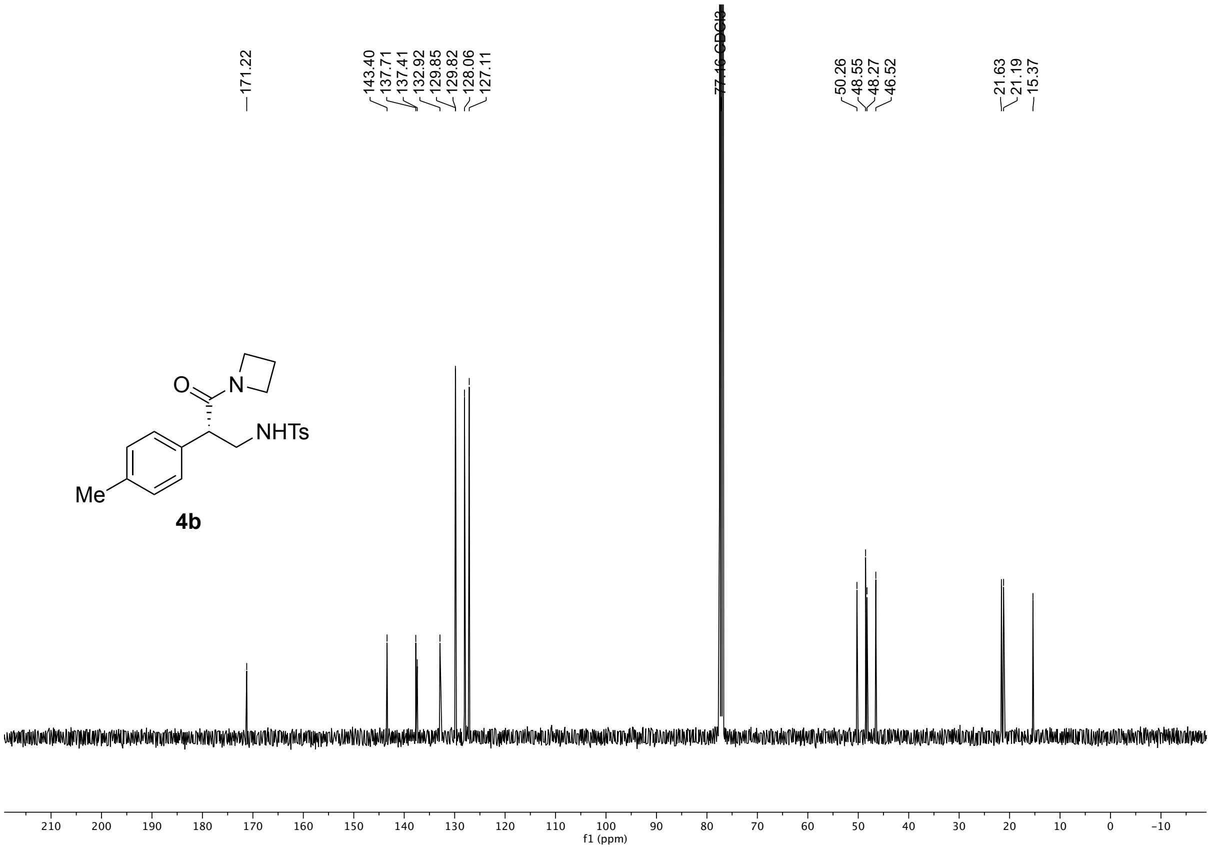
171.22
143.40
137.71
137.41
132.92
129.85
129.82
128.06
127.11
77.16 CDCl3
50.26
48.55
48.27
46.52
21.63
21.19
15.37
O
N
NHTs
Me
4b
210 200 190 180 170 160 150 140 130 120 110 100 90 80 70 60 50 40 30 20 10 0 -10
f1 (ppm)

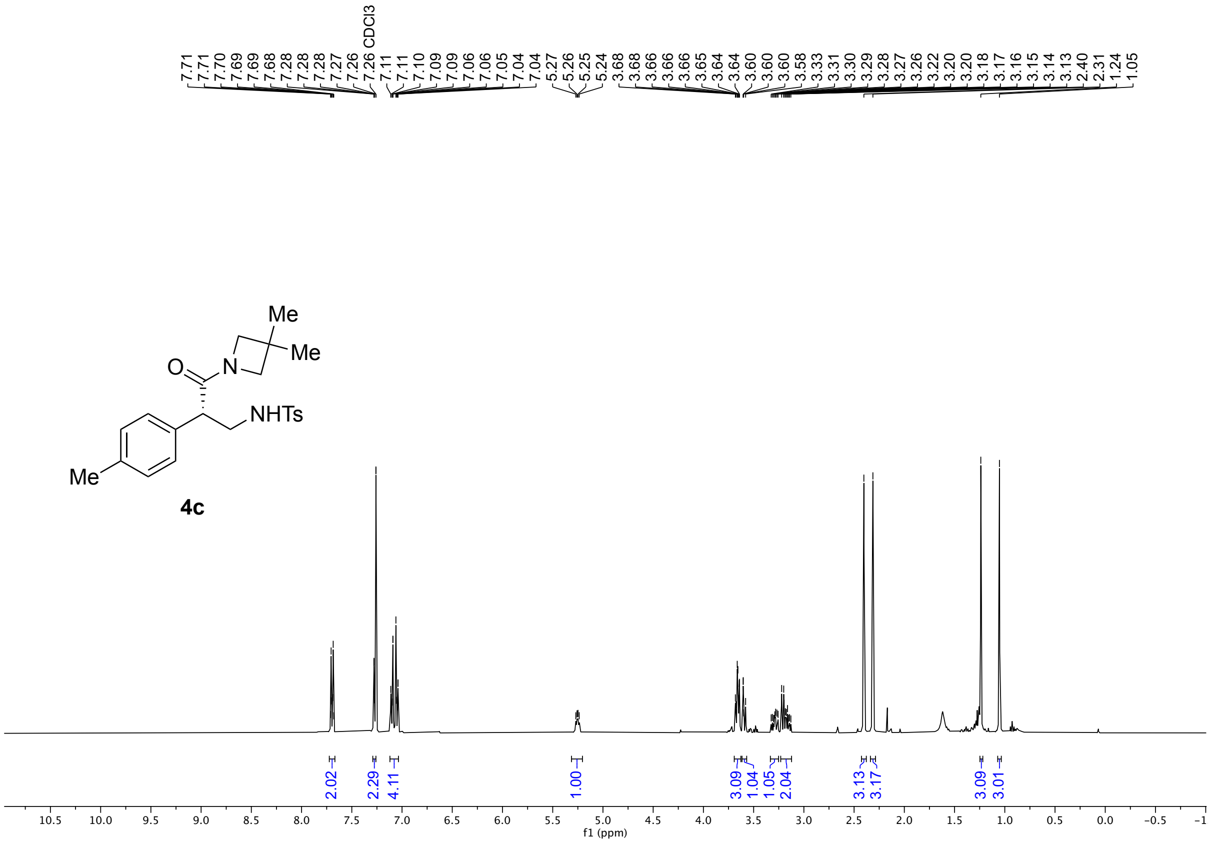
7.71 7.71 7.70 7.69 7.69 7.68 7.28 7.28 7.28 7.27 7.26 7.26 CDCl3 7.11 7.11 7.10 7.09 7.09 7.06 7.06 7.05 7.04 7.04 5.27 5.26 5.25 5.24 3.68 3.68 3.66 3.66 3.65 3.64 3.64 3.60 3.60 3.60 3.58 3.33 3.31 3.30 3.29 3.28 3.27 3.26 3.22 3.20 3.20 3.18 3.17 3.16 3.15 3.14 3.13 2.40 2.31 1.24 1.05
Me
Me
O
N
NHTs
Me
4c
2.02
2.29
4.11
1.00
3.09
1.04
1.05
2.04
3.13
3.17
3.09
3.01
f1 (ppm)

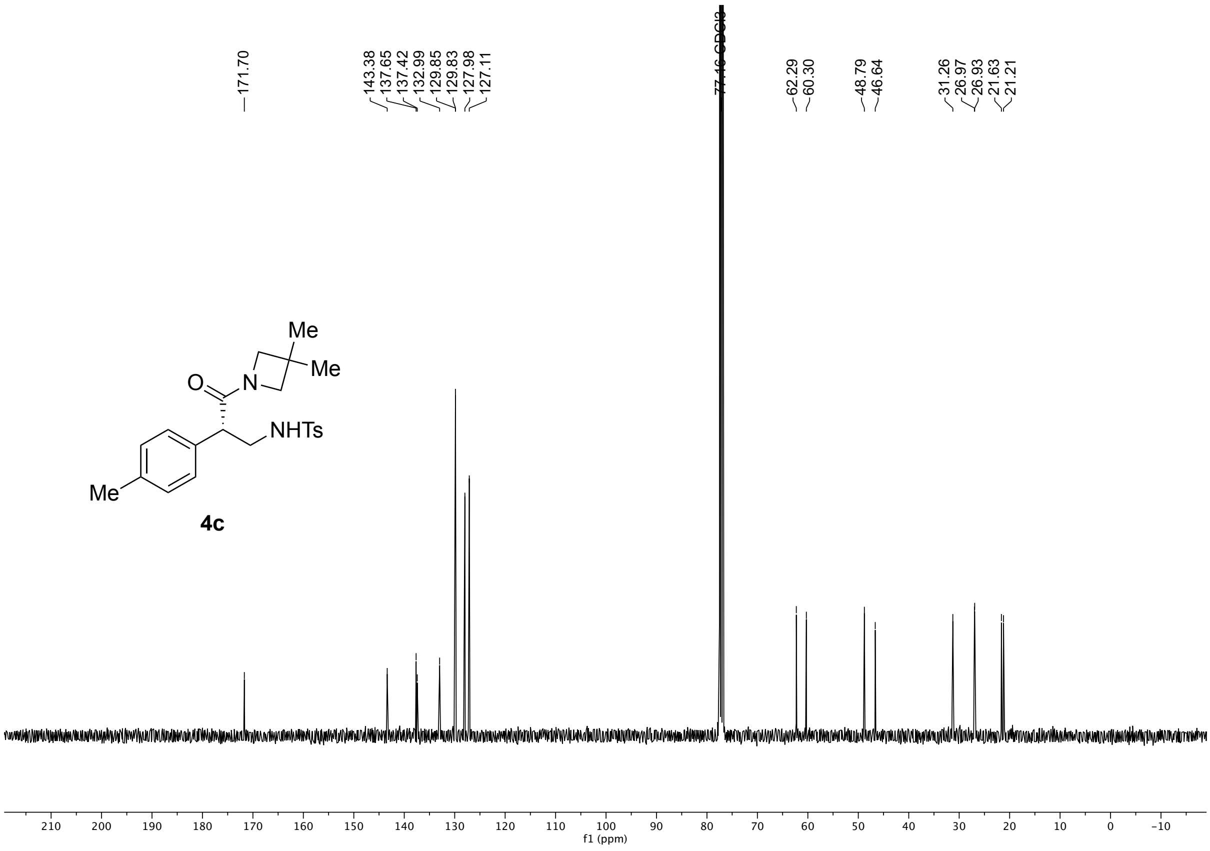
171.70
143.38 137.65 137.42 132.99 129.85 129.83 127.98 127.11
77.16 CDCl3
62.29 60.30
48.79 46.64
31.26 26.97 26.93 21.63 21.21
Me
Me
O
N
NHTs
Me
4c
f1 (ppm)

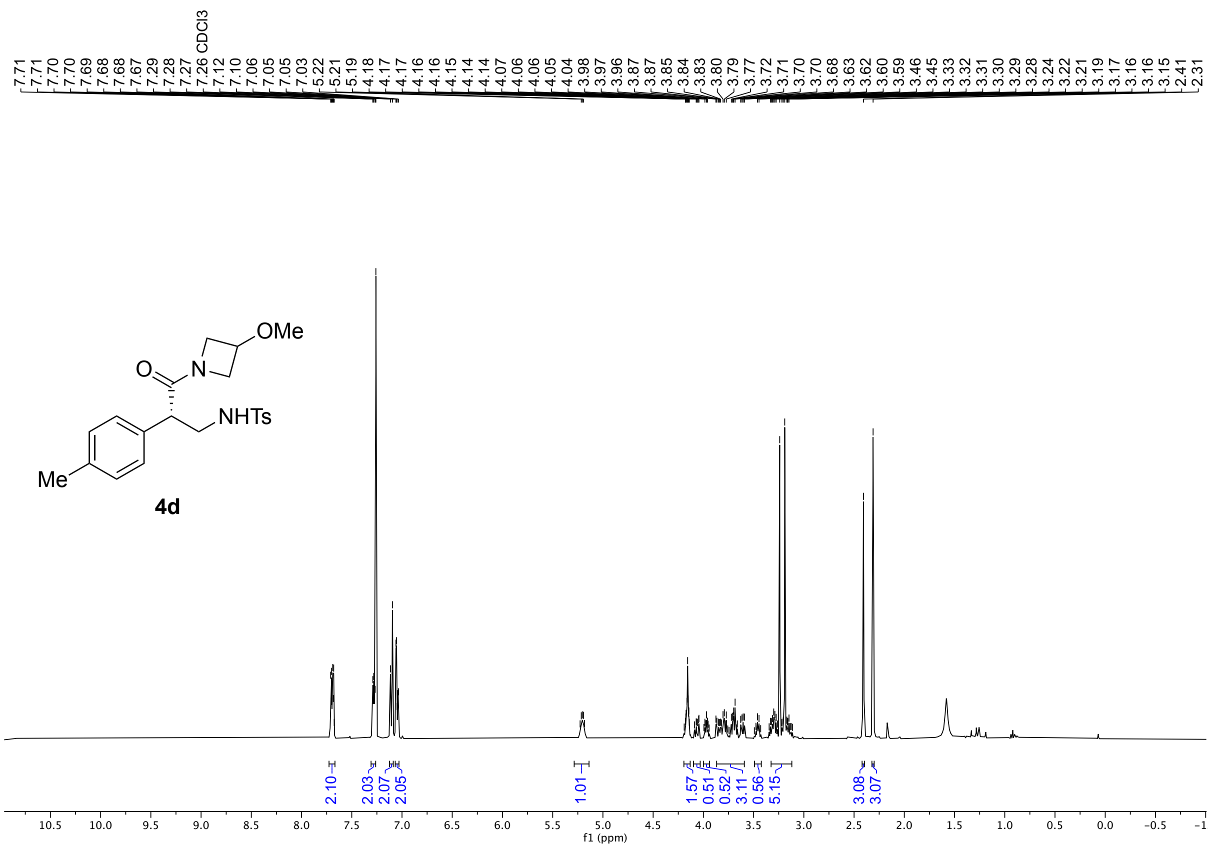

OMe
O
N
NHTs
Me
4d
CDCl3
f1 (ppm)


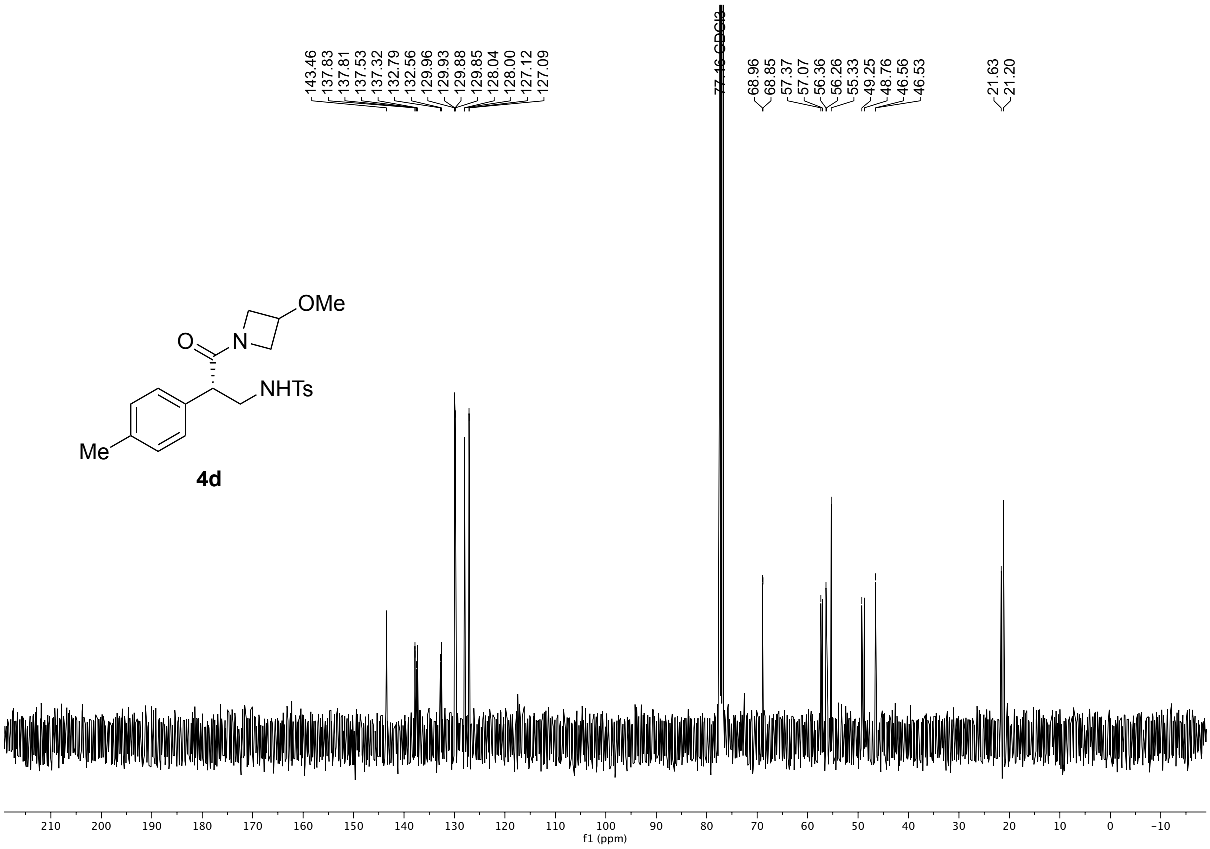

OMe
O
N
NHTs
Me
4d
77.16 CDCl3
f1 (ppm)

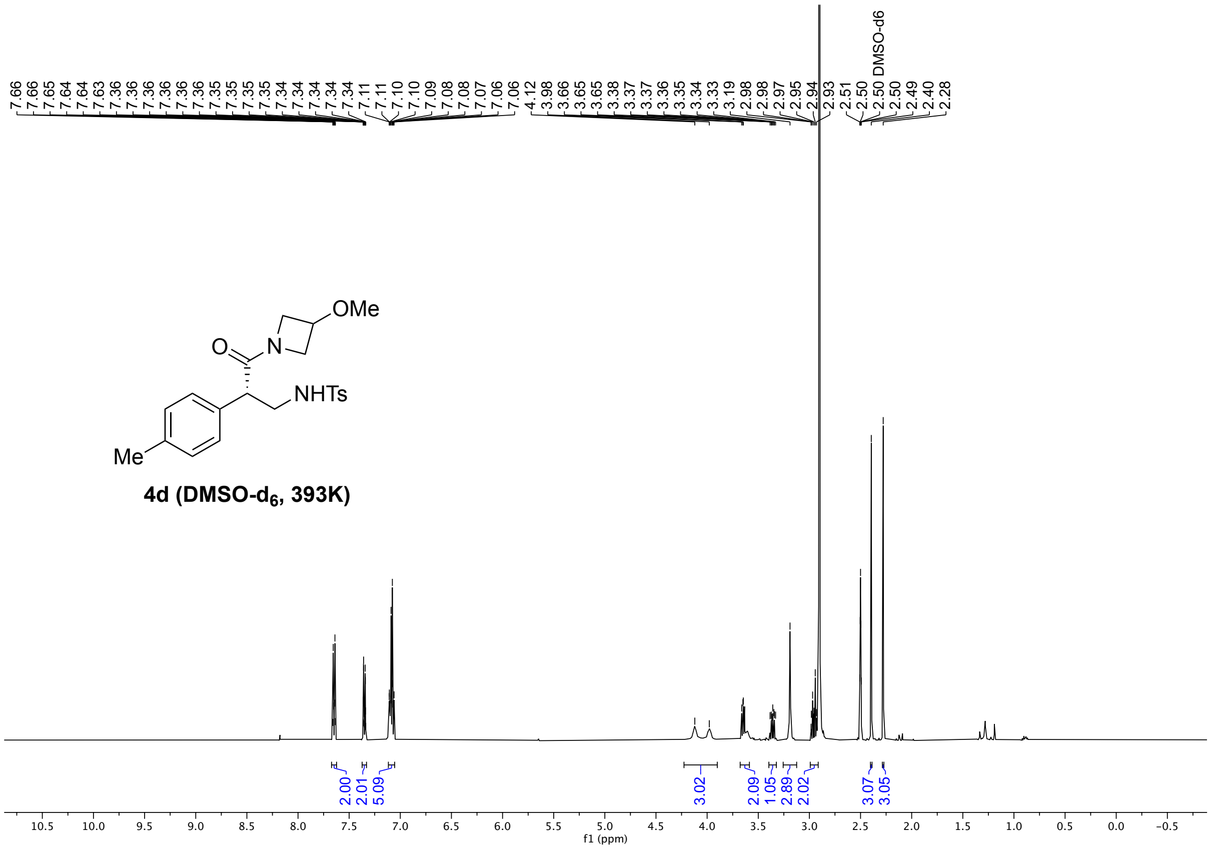


**4d (DMSO-$d_6$, 393K)**

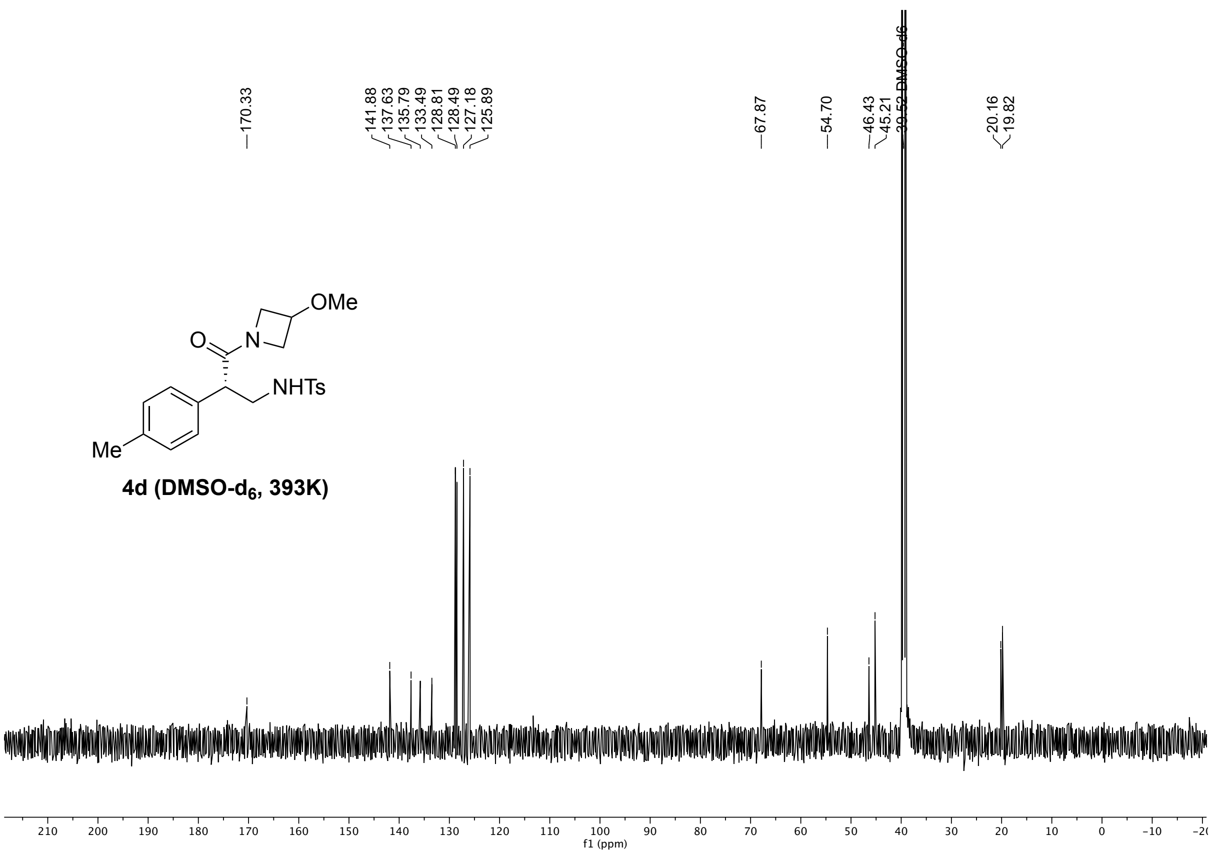


**4d (DMSO-$d_6$, 393K)**

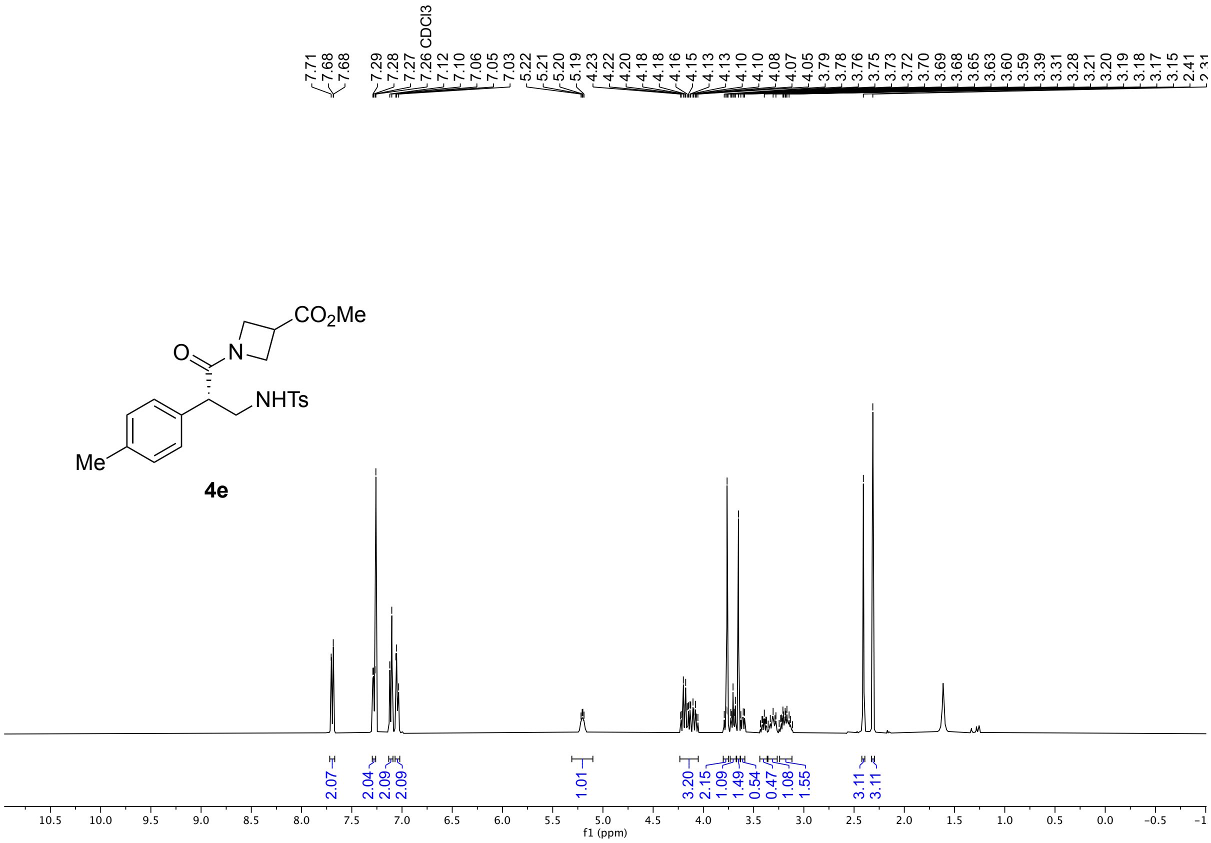
7.71
7.68
7.68
7.29
7.28
7.27
7.26 CDCl3
7.12
7.10
7.06
7.05
7.03
5.22
5.21
5.20
5.19
4.23
4.22
4.20
4.18
4.18
4.16
4.15
4.13
4.13
4.10
4.10
4.08
4.07
4.05
3.79
3.78
3.76
3.75
3.73
3.72
3.70
3.69
3.68
3.65
3.63
3.60
3.59
3.39
3.31
3.28
3.21
3.20
3.19
3.18
3.17
3.15
2.41
CO2Me
O
N
NHTs
Me
4e
2.07
2.04
2.09
2.09
1.01
3.20
2.15
1.09
1.49
0.54
0.47
1.08
1.55
3.11
3.11
f1 (ppm)

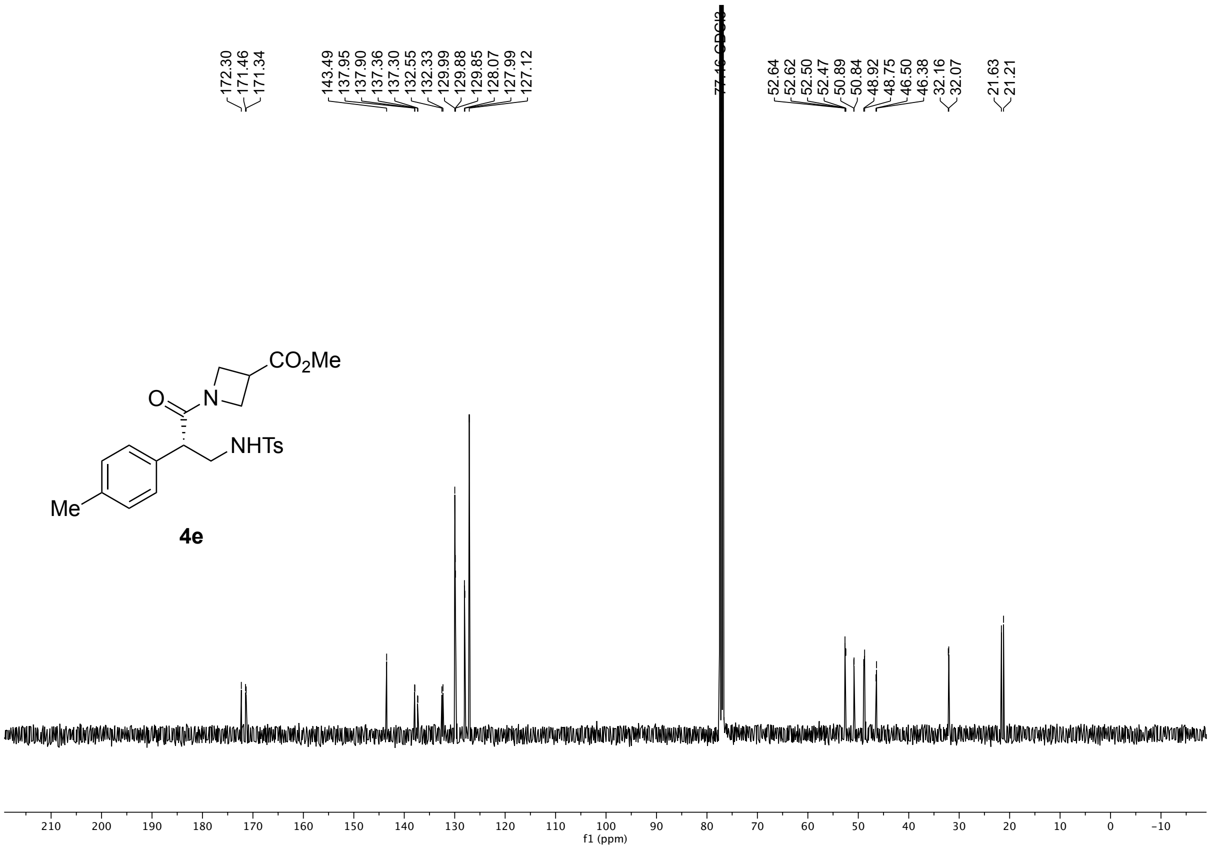
172.30
171.46
171.34
143.49
137.95
137.90
137.36
137.30
132.55
132.33
129.99
129.88
129.85
128.07
127.99
127.12
52.64
52.62
52.50
52.47
50.89
50.84
48.92
48.75
46.50
46.38
32.16
32.07
21.63
21.21
CO2Me
O
N
NHTs
Me
4e
f1 (ppm)

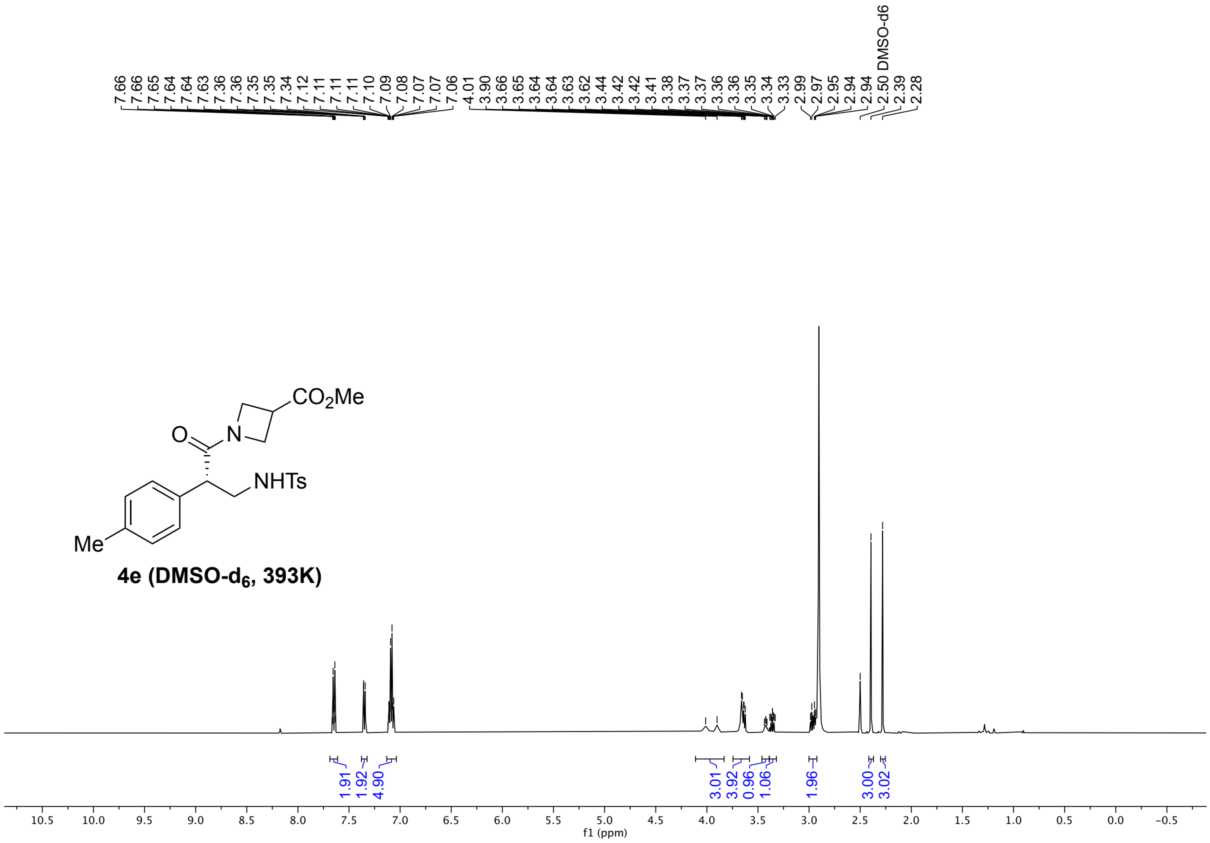
7.66 7.66 7.65 7.64 7.64 7.63 7.36 7.36 7.35 7.35 7.34 7.12 7.11 7.11 7.10 7.09 7.08 7.07 7.07 7.06 4.01 3.90 3.66 3.65 3.64 3.63 3.62 3.44 3.42 3.41 3.38 3.37 3.36 3.36 3.35 3.34 3.33 2.99 2.97 2.95 2.94 2.94 2.50 DMSO-d6 2.39 2.28
CO2Me
O
N
NHTs
Me
4e (DMSO-d6, 393K)
1.91 1.92 4.90 3.01 3.92 0.96 1.06 1.96 3.00 3.02
f1 (ppm)

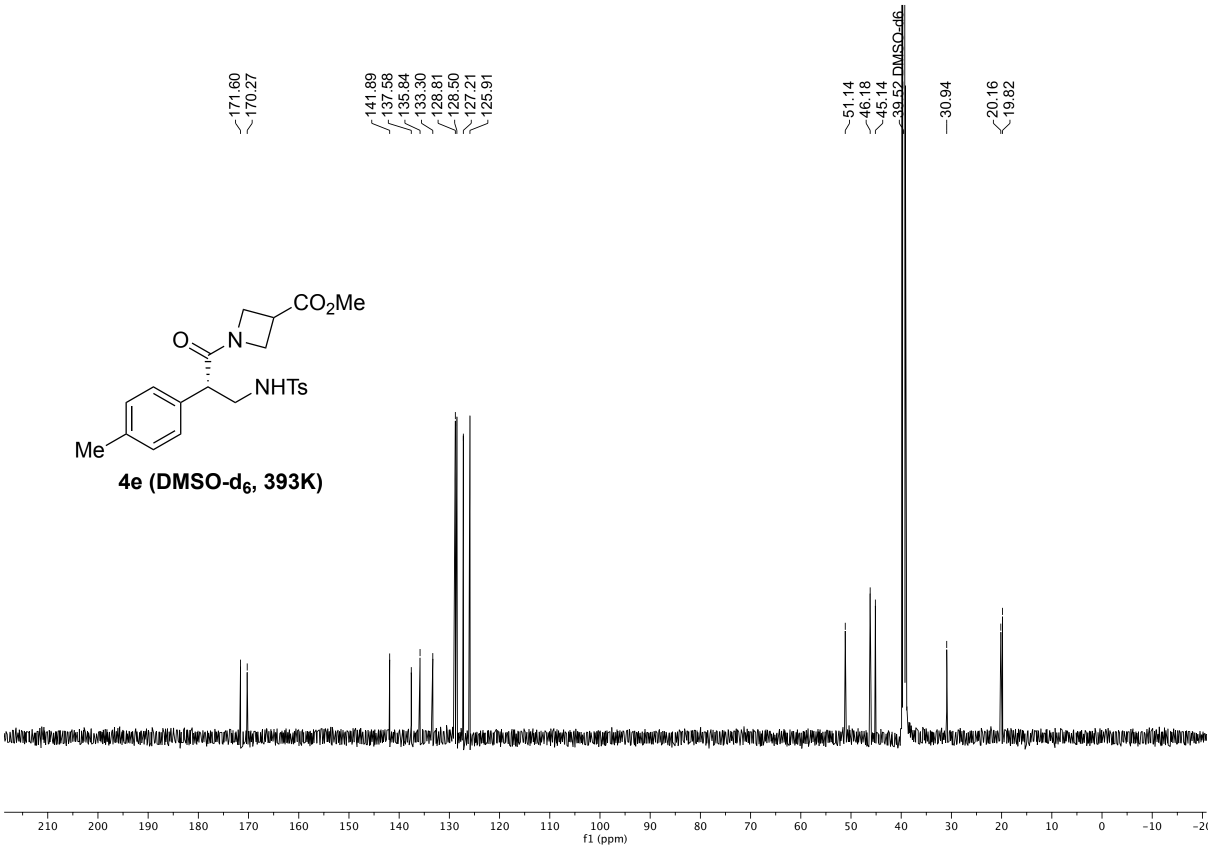
171.60 170.27
141.89 137.58 135.84 133.30 128.81 128.50 127.21 125.91
51.14 46.18 45.14 39.52 DMSO-d6
30.94
20.16 19.82
CO2Me
O
N
NHTs
Me
4e (DMSO-d6, 393K)
f1 (ppm)

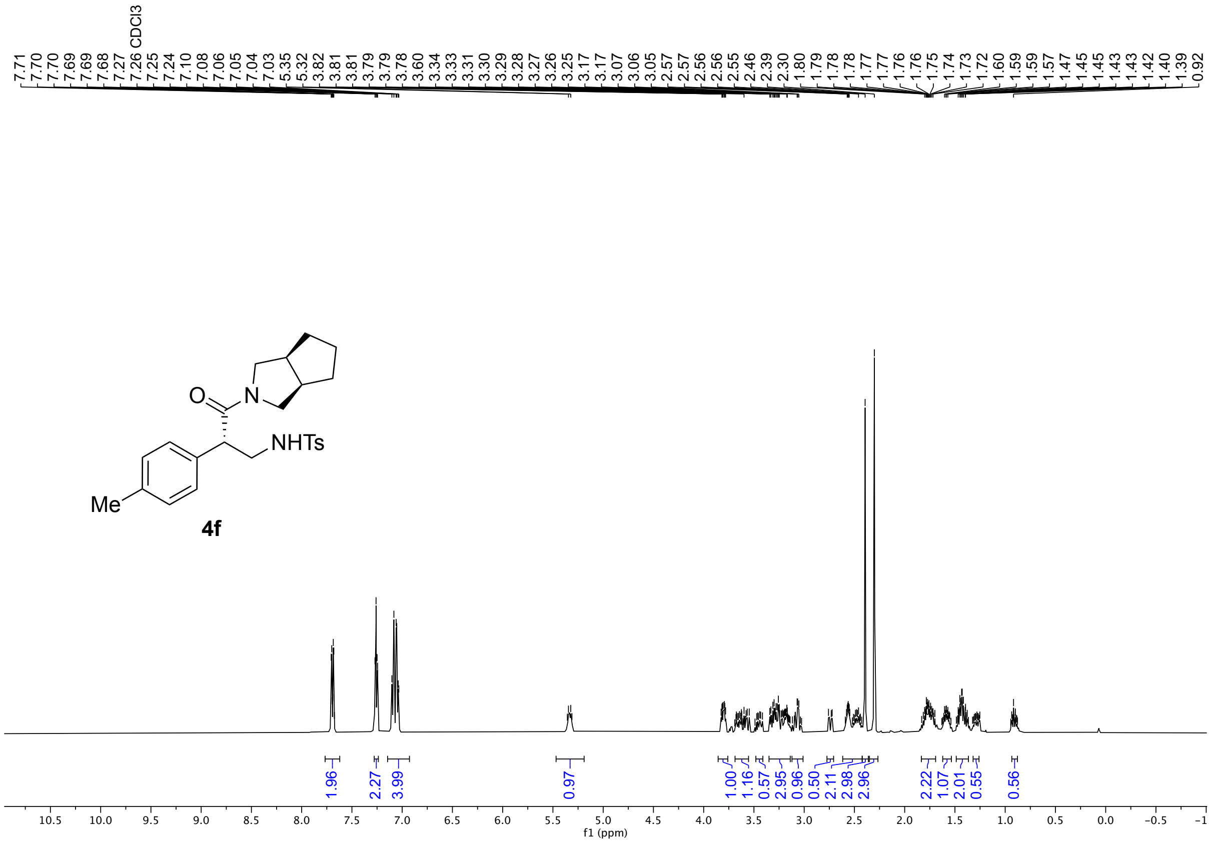

7.71 7.70 7.70 7.69 7.68 7.27 7.26 CDCl3 7.25 7.24 7.10 7.08 7.06 7.05 7.04 7.03 5.35 5.32 3.82 3.81 3.81 3.79 3.79 3.78 3.60 3.34 3.33 3.31 3.30 3.29 3.28 3.27 3.26 3.25 3.17 3.17 3.07 3.06 3.05 2.57 2.57 2.56 2.55 2.46 2.39 2.30 1.80 1.79 1.78 1.78 1.77 1.77 1.76 1.76 1.75 1.74 1.73 1.72 1.60 1.59 1.59 1.57 1.47 1.45 1.45 1.43 1.43 1.42 1.40 1.39 0.92
O
N
NHTs
Me
4f
1.96 2.27 3.99 0.97 1.00 1.16 0.57 2.95 0.96 0.50 2.11 2.98 2.96 2.22 1.07 2.01 0.55 0.56
10.5 10.0 9.5 9.0 8.5 8.0 7.5 7.0 6.5 6.0 5.5 5.0 4.5 4.0 3.5 3.0 2.5 2.0 1.5 1.0 0.5 0.0 −0.5 −1
f1 (ppm)


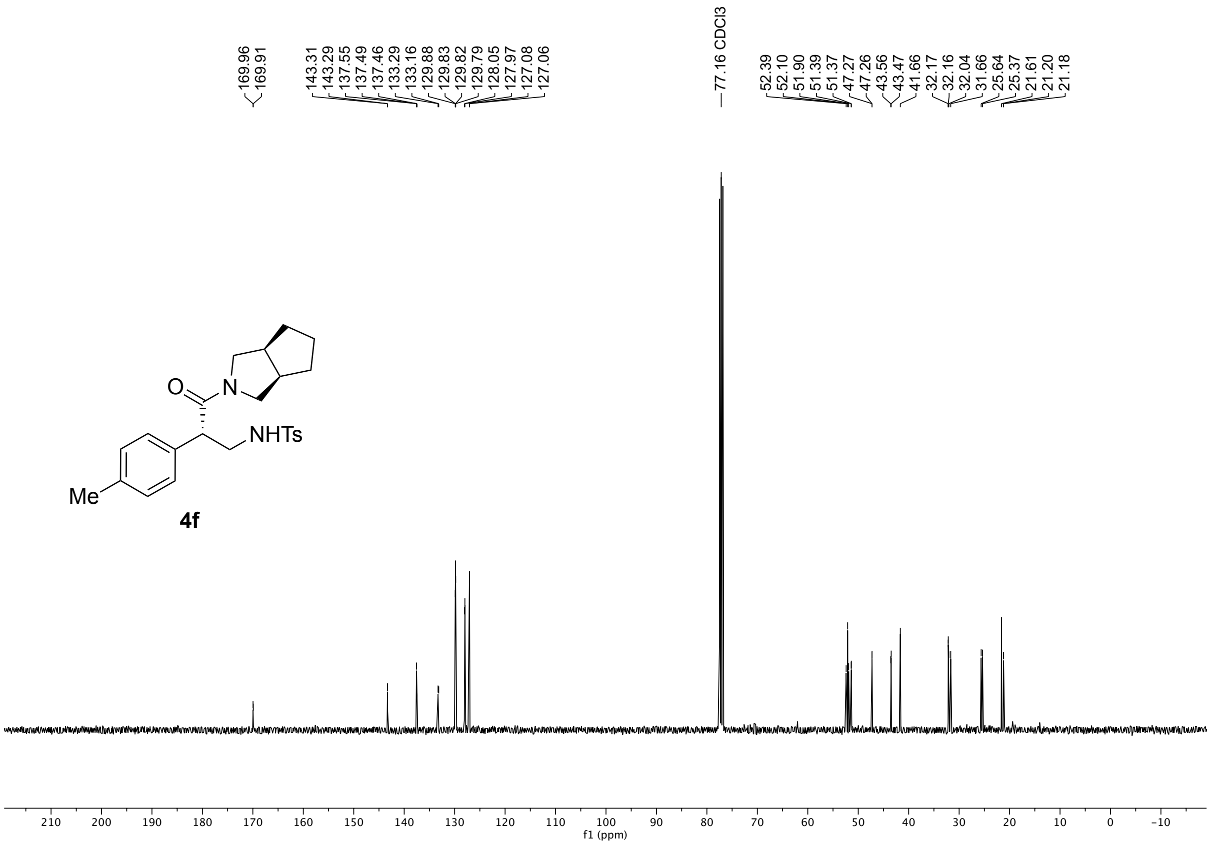

169.96 169.91 143.31 143.29 137.55 137.49 137.46 133.29 133.16 129.88 129.83 129.82 129.79 128.05 127.97 127.08 127.06 77.16 CDCl3 52.39 52.10 51.90 51.39 51.37 47.27 47.26 43.56 43.47 41.66 32.17 32.16 32.04 31.66 25.64 25.37 21.61 21.20 21.18
O
N
NHTs
Me
4f
210 200 190 180 170 160 150 140 130 120 110 100 90 80 70 60 50 40 30 20 10 0 −10
f1 (ppm)

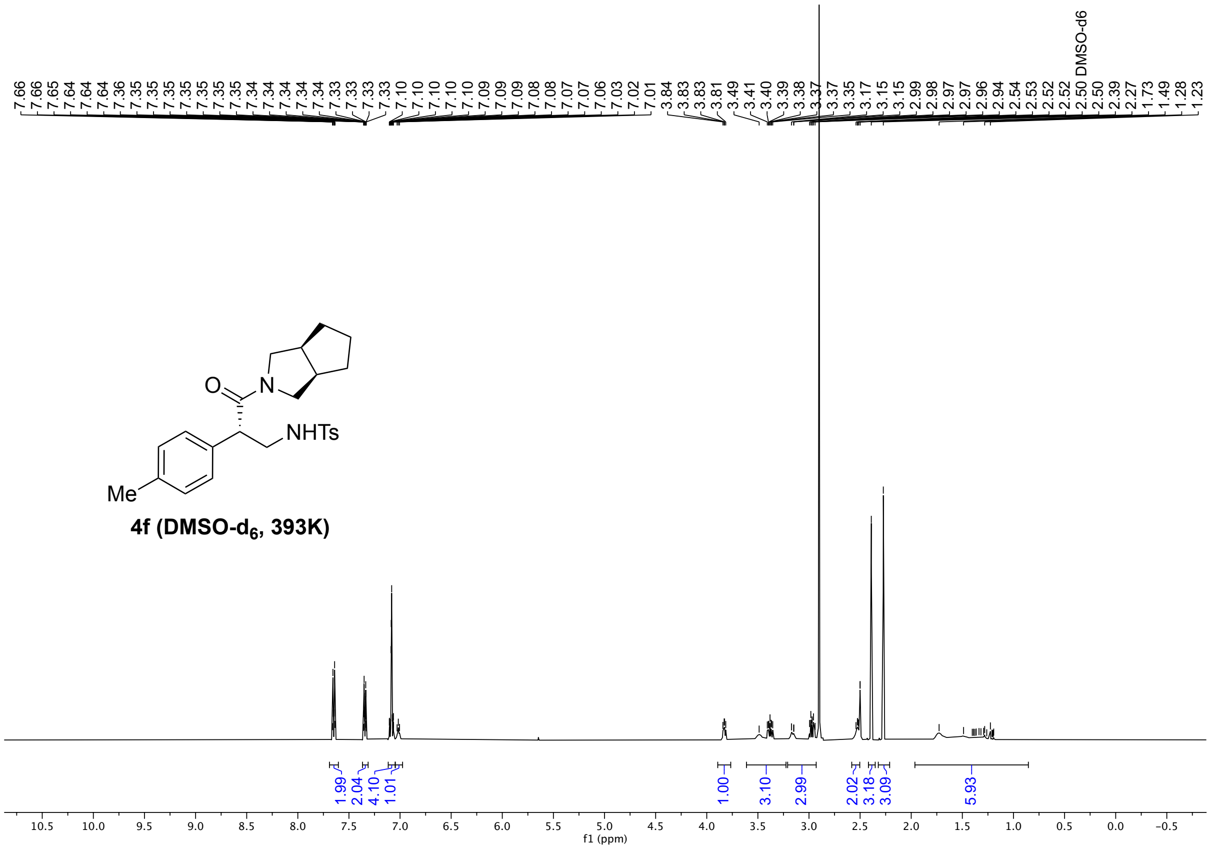
2.50 DMSO-d6
NHTs
Me
4f (DMSO-$d_6$, 393K)
f1 (ppm)

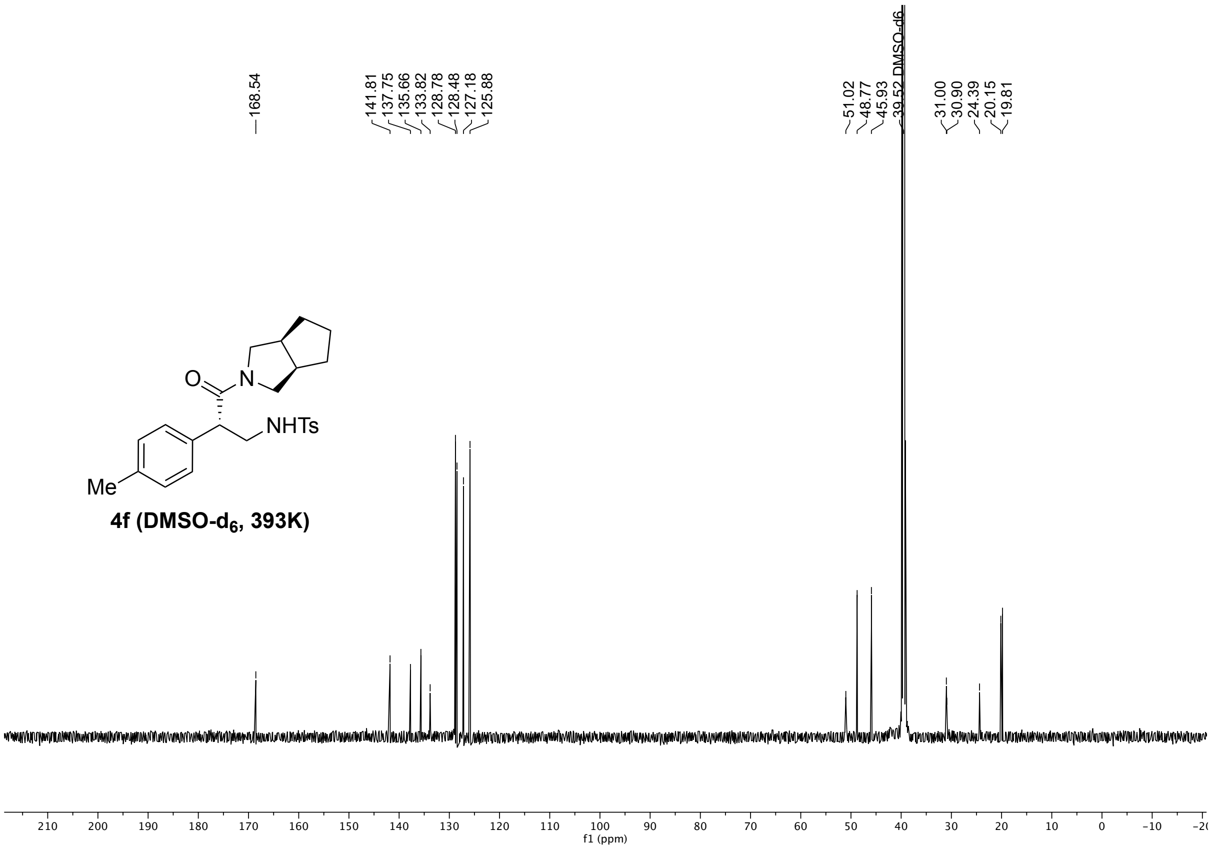
168.54
141.81
137.75
135.66
133.82
128.78
128.48
127.18
125.88
51.02
48.77
45.93
39.52 DMSO-d6
31.00
30.90
24.39
20.15
19.81
NHTs
Me
4f (DMSO-$d_6$, 393K)
f1 (ppm)

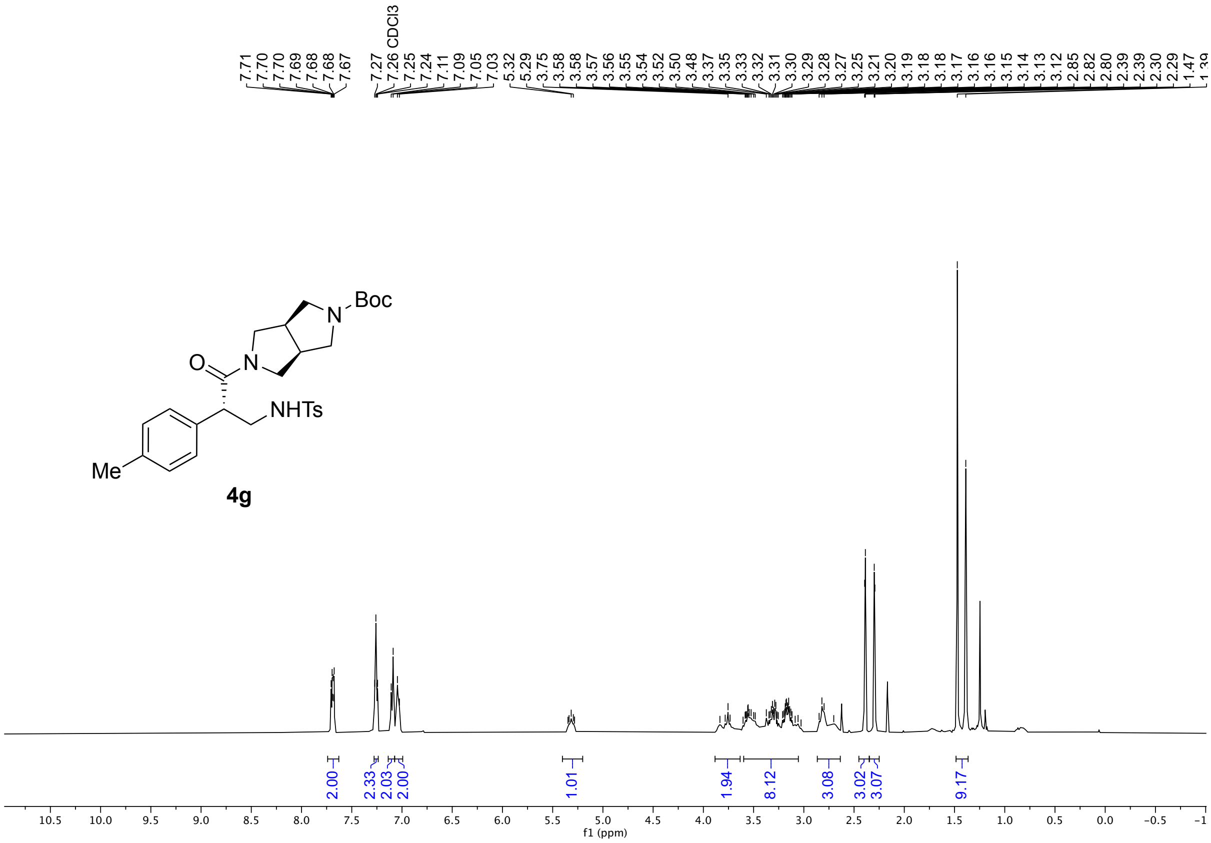

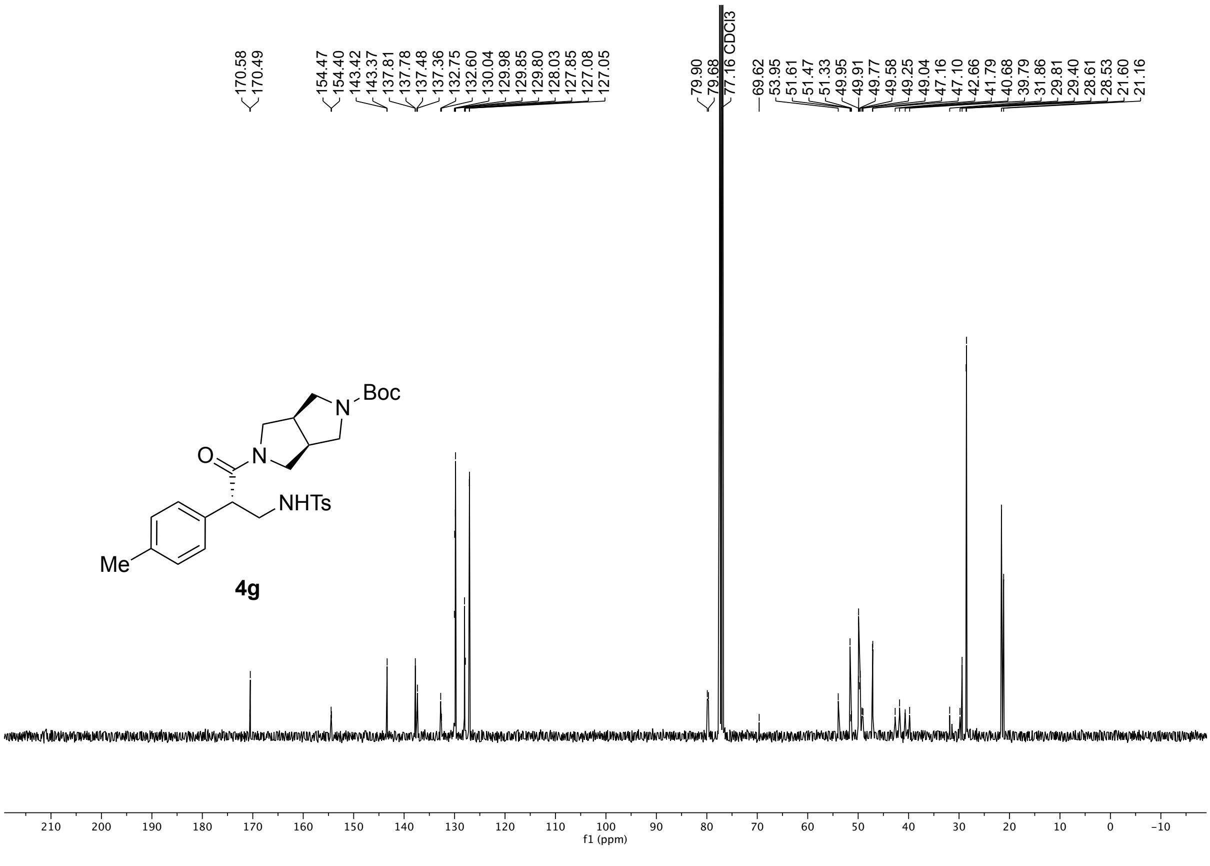

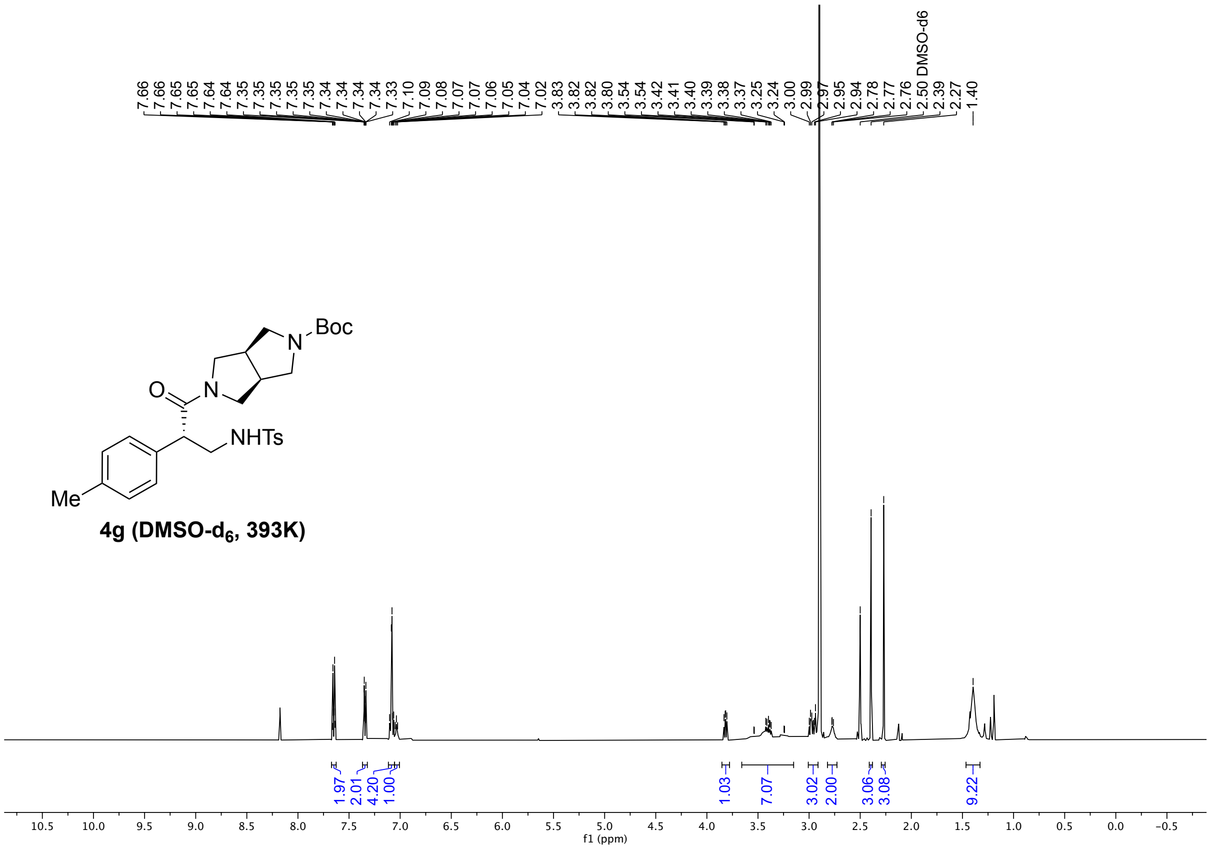
7.66 7.66 7.65 7.65 7.64 7.64 7.35 7.35 7.35 7.35 7.34 7.34 7.34 7.33 7.10 7.09 7.08 7.07 7.07 7.06 7.05 7.04 7.02 3.83 3.82 3.82 3.80 3.54 3.54 3.42 3.41 3.40 3.39 3.38 3.37 3.25 3.24 3.00 2.99 2.97 2.95 2.94 2.78 2.77 2.76 2.50 DMSO-d6 2.39 2.27 1.40
Boc
N
O
NHTs
Me
4g (DMSO-d6, 393K)
1.97 2.01 4.20 1.00 1.03 7.07 3.02 2.00 3.06 3.08 9.22
f1 (ppm)

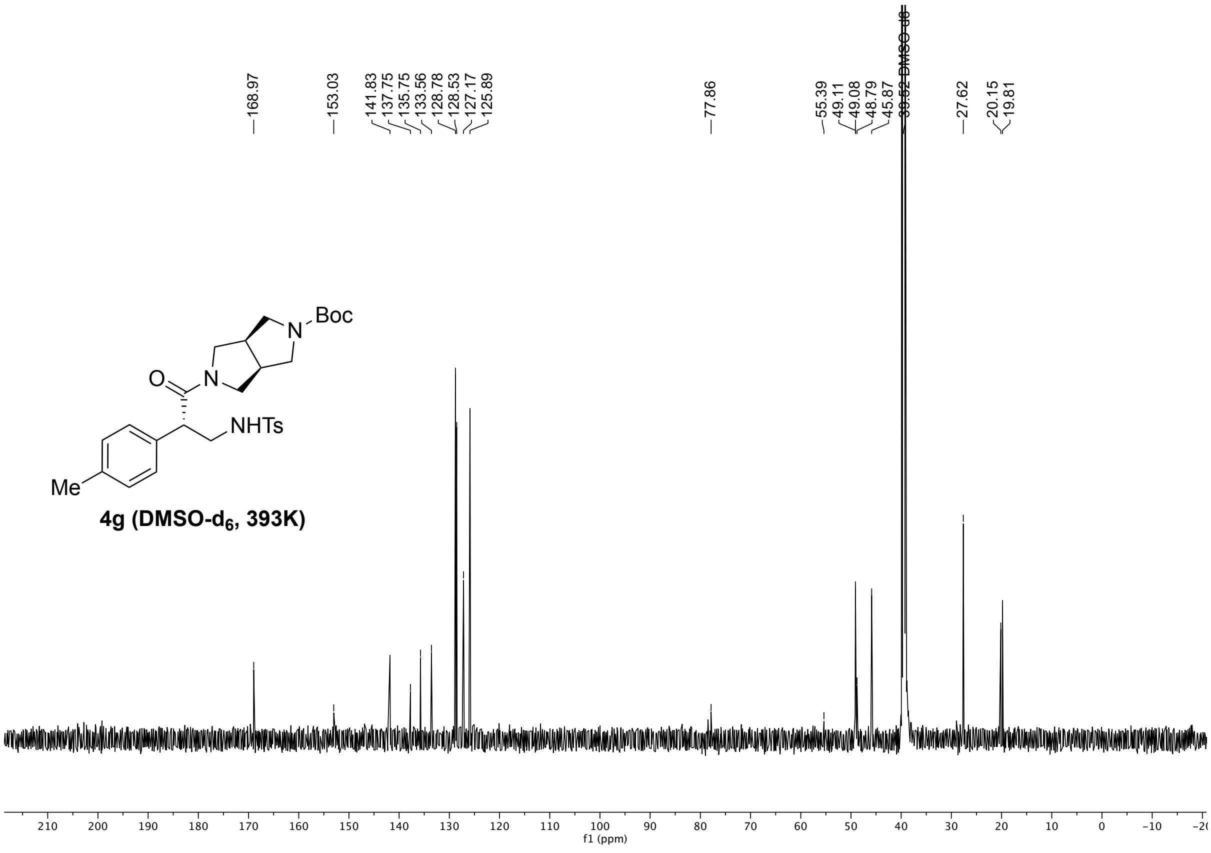
168.97 153.03 141.83 137.75 135.75 133.56 128.78 128.53 127.17 125.89 77.86 55.39 49.11 49.08 48.79 45.87 39.52 DMSO-d6 27.62 20.15 19.81
Boc
N
O
NHTs
Me
4g (DMSO-d6, 393K)
f1 (ppm)

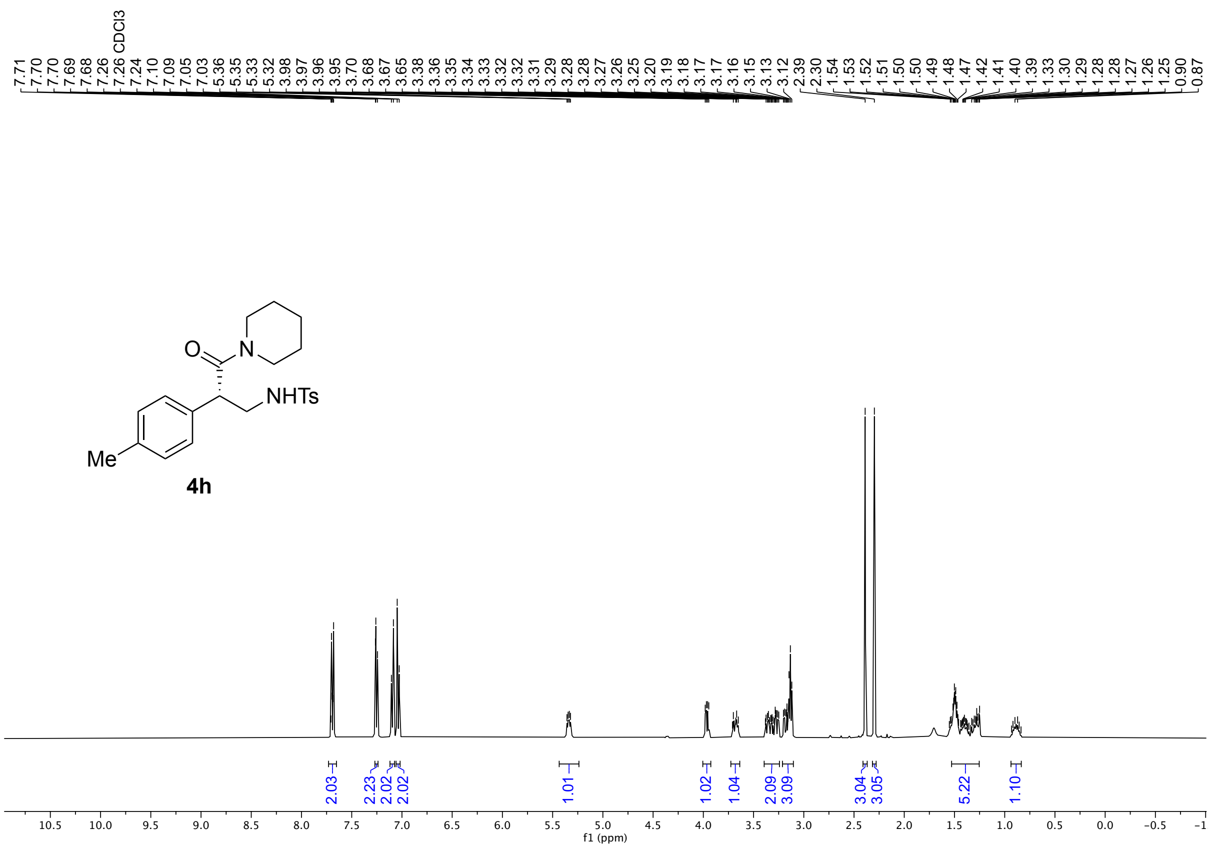
7.71 7.70 7.70 7.69 7.68 7.26 7.26 CDCl3 7.24 7.10 7.09 7.05 7.03 5.36 5.35 5.33 5.32 3.98 3.97 3.96 3.95 3.70 3.68 3.67 3.65 3.38 3.36 3.35 3.34 3.33 3.32 3.32 3.31 3.29 3.28 3.28 3.27 3.26 3.25 3.20 3.19 3.18 3.17 3.17 3.16 3.15 3.13 3.12 2.39 2.30 1.54 1.53 1.52 1.51 1.50 1.49 1.48 1.47 1.42 1.41 1.40 1.39 1.33 1.30 1.29 1.28 1.28 1.27 1.26 1.25 0.90 0.87
O
N
NHTs
Me
4h
2.03 2.23 2.02 2.02 1.01 1.02 1.04 2.09 3.09 3.04 3.05 5.22 1.10
f1 (ppm)

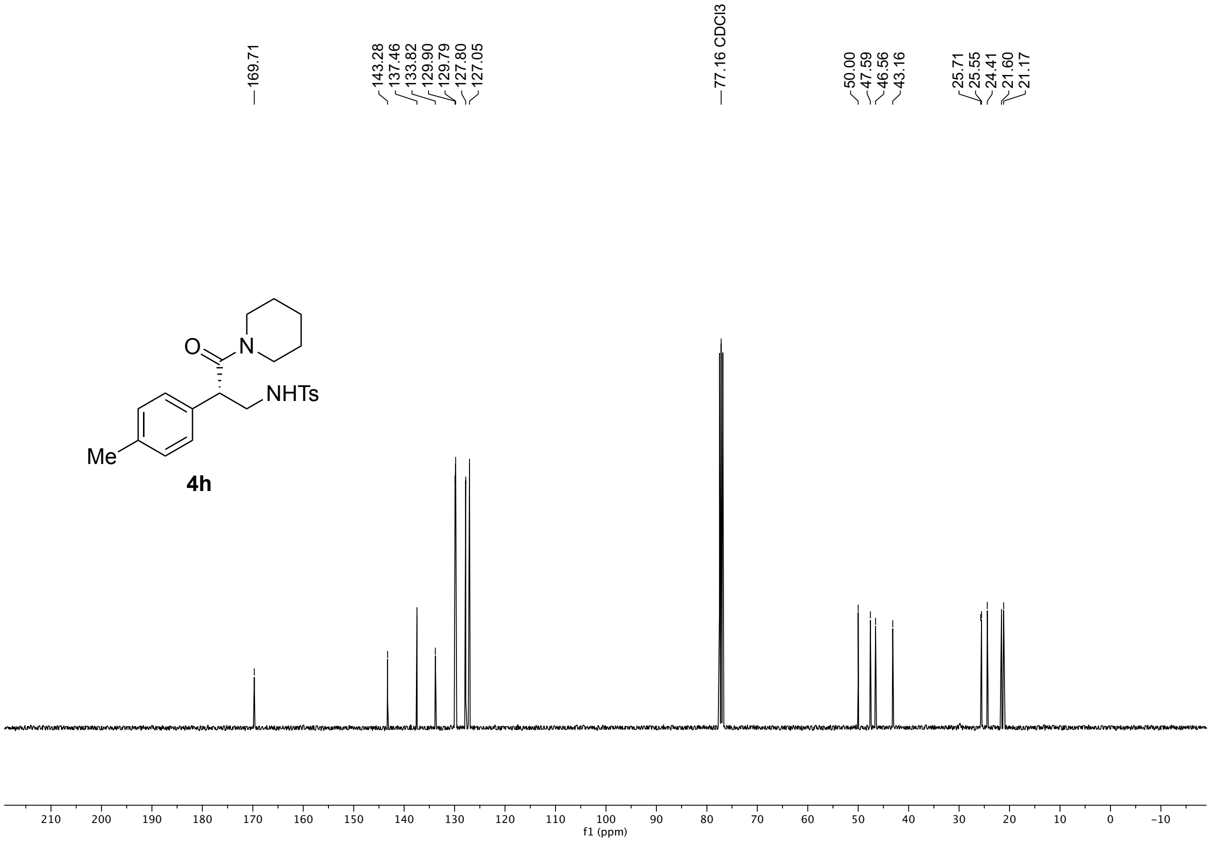
169.71
143.28 137.46 133.82 129.90 129.79 127.80 127.05
77.16 CDCl3
50.00 47.59 46.56 43.16
25.71 25.55 24.41 21.60 21.17
O
N
NHTs
Me
4h
f1 (ppm)

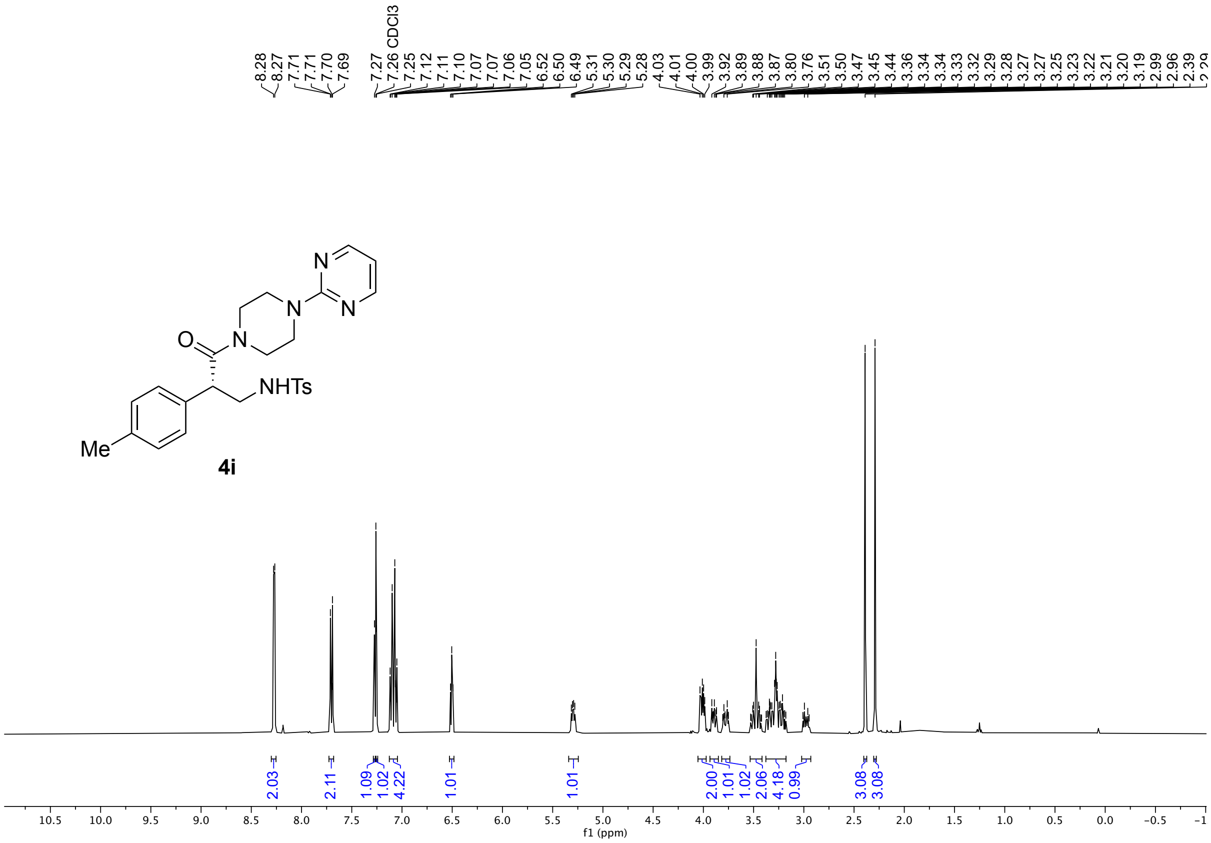

8.28
8.27
7.71
7.71
7.70
7.69
7.27
7.26 CDCl3
7.25
7.12
7.11
7.10
7.07
7.06
7.05
6.52
6.50
6.49
5.31
5.30
5.29
5.28
4.03
4.01
4.00
3.99
3.92
3.89
3.88
3.87
3.80
3.76
3.51
3.50
3.47
3.45
3.44
3.36
3.34
3.34
3.33
3.32
3.29
3.28
3.27
3.27
3.25
3.23
3.21
3.20
3.19
2.99
2.96
2.39
N
N
N
N
O
NHTs
Me
4i
2.03
2.11
1.09
1.02
4.22
1.01
1.01
2.00
1.01
1.02
2.06
4.18
0.99
3.08
3.08
f1 (ppm)


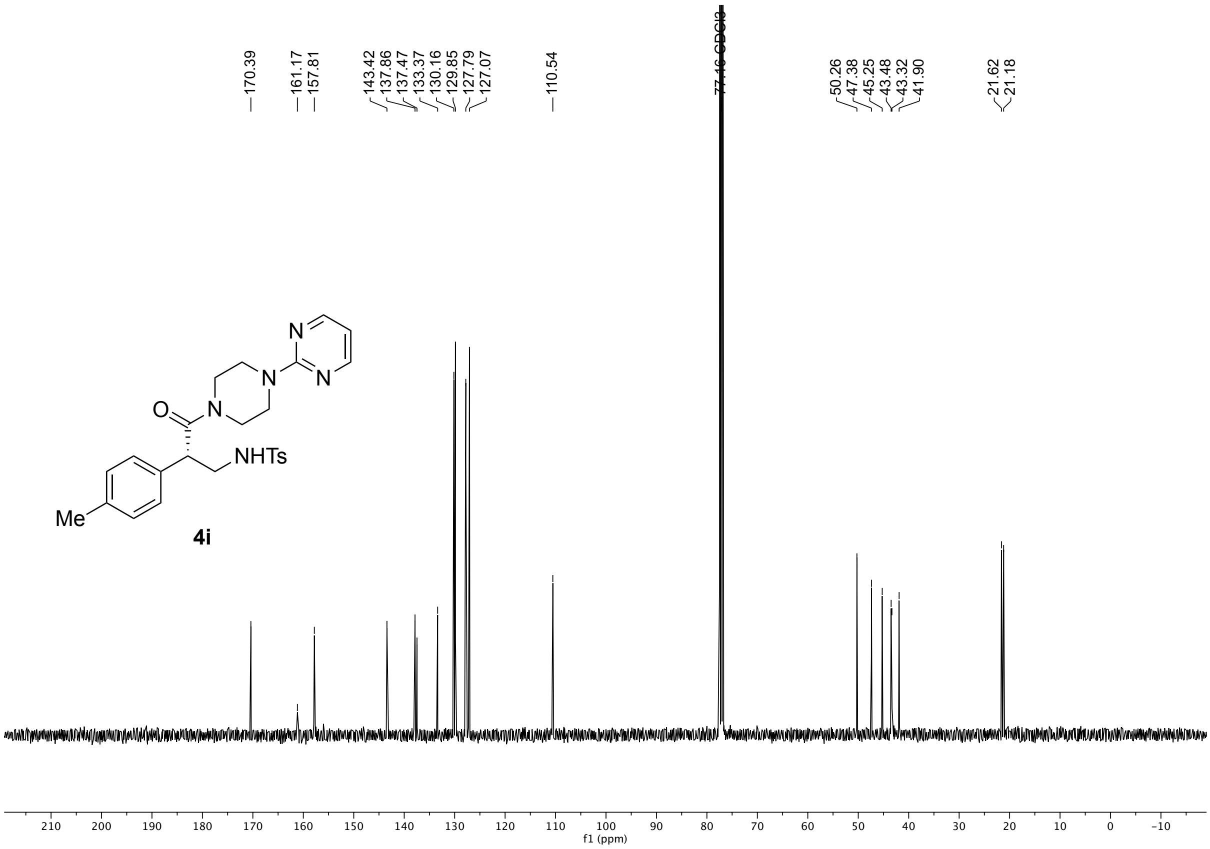

170.39
161.17
157.81
143.42
137.86
137.47
133.37
130.16
129.85
127.79
127.07
110.54
77.16 CDCl3
50.26
47.38
45.25
43.48
43.32
41.90
21.62
21.18
N
N
N
N
O
NHTs
Me
4i
f1 (ppm)

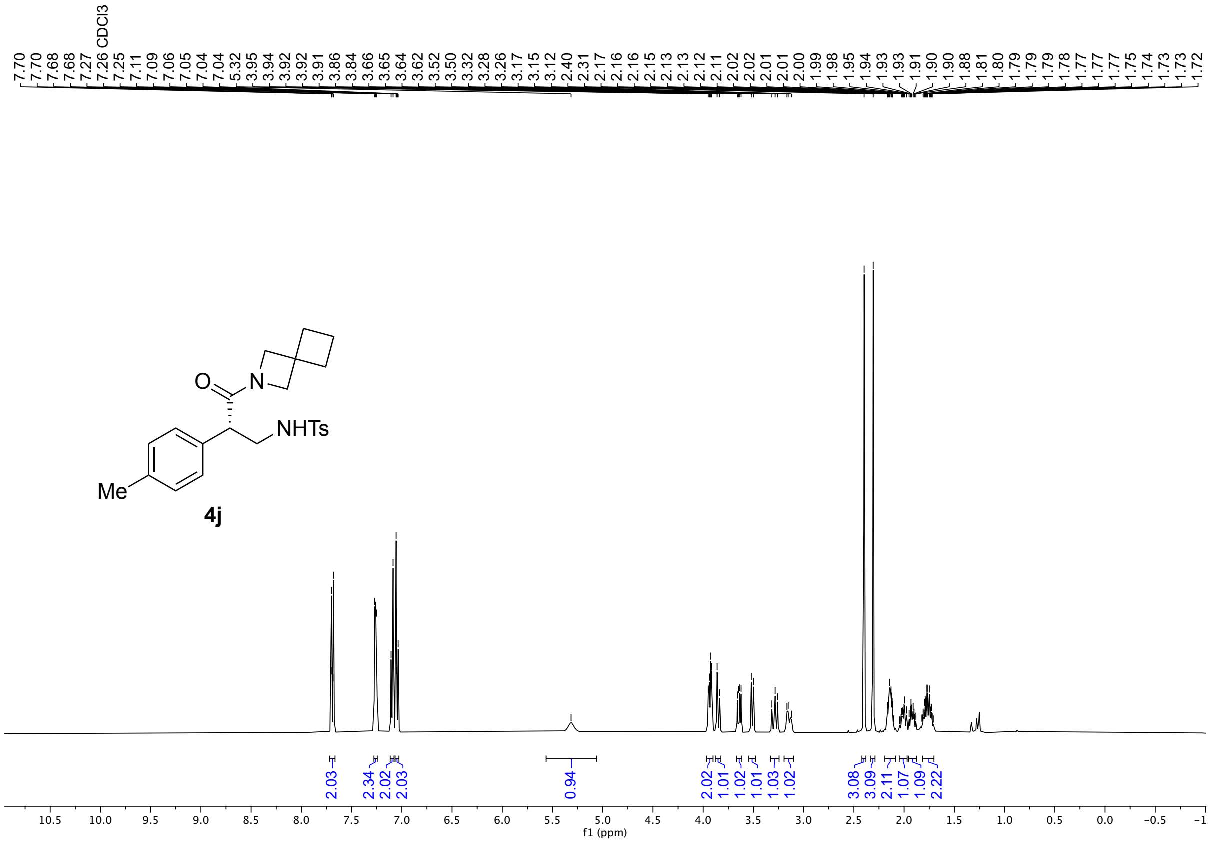
7.70
7.70
7.68
7.68
7.27
7.26 CDCl3
7.25
7.11
7.09
7.06
7.05
7.04
7.04
5.32
3.95
3.94
3.92
3.92
3.91
3.86
3.84
3.66
3.65
3.64
3.62
3.52
3.50
3.32
3.28
3.26
3.17
3.15
3.12
2.40
2.31
2.17
2.16
2.16
2.15
2.13
2.13
2.12
2.11
2.02
2.02
2.01
2.01
2.00
1.99
1.98
1.95
1.94
1.93
1.93
1.91
1.90
1.90
1.88
1.81
1.80
1.79
1.79
1.78
1.77
1.77
1.77
1.75
1.74
1.73
1.73
1.72
O
N
NHTs
Me
4j
2.03
2.34
2.02
2.03
0.94
2.02
1.01
1.02
1.01
1.03
1.02
3.08
3.09
2.11
1.07
1.09
2.22
f1 (ppm)

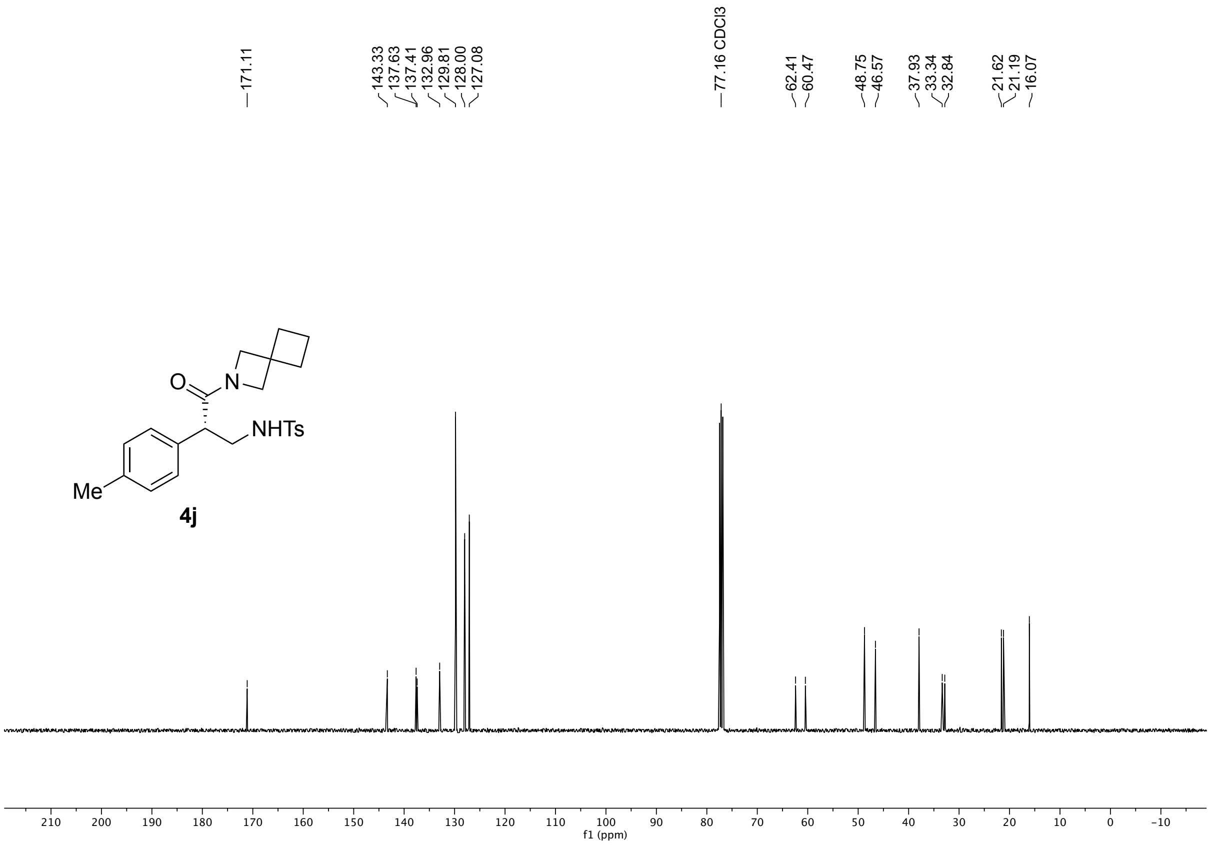
171.11
143.33
137.63
137.41
132.96
129.81
128.00
127.08
77.16 CDCl3
62.41
60.47
48.75
46.57
37.93
33.34
32.84
21.62
21.19
16.07
O
N
NHTs
Me
4j
f1 (ppm)

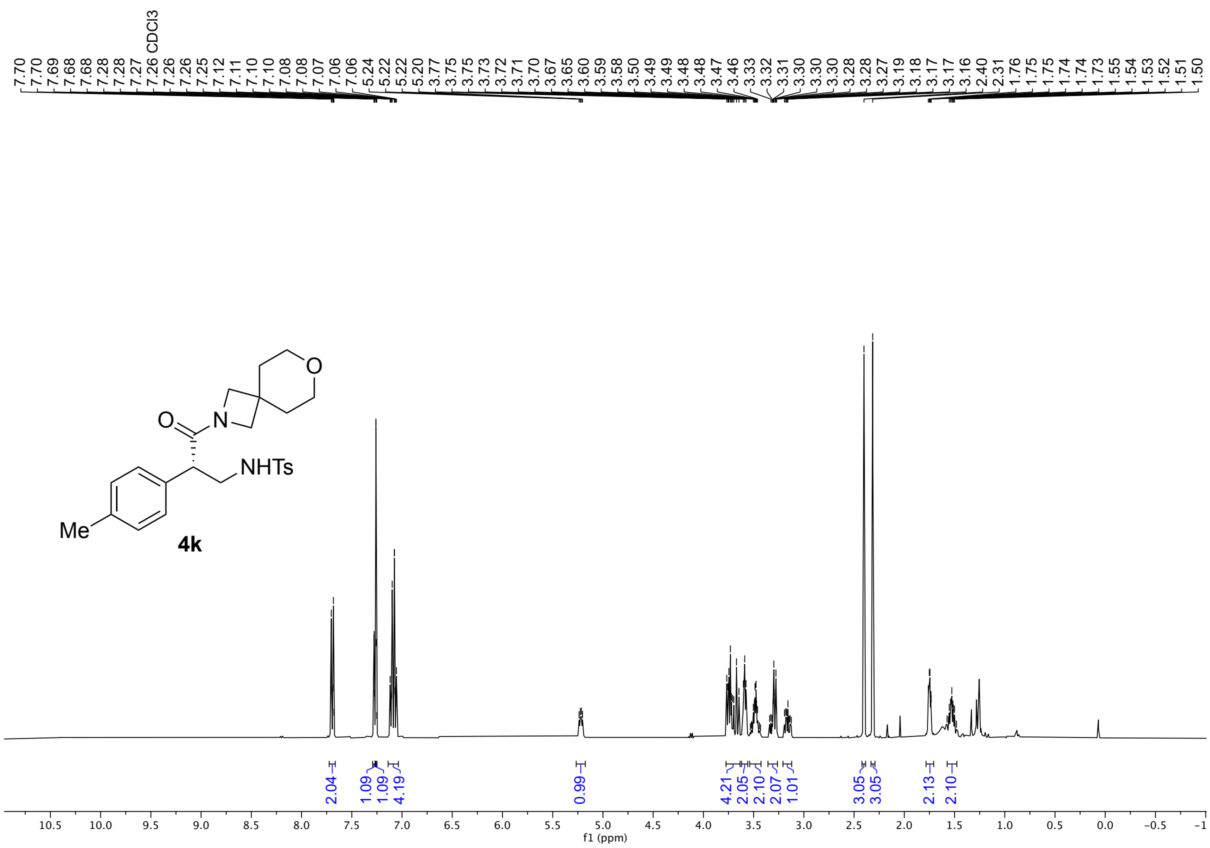
7.70
7.70
7.69
7.68
7.68
7.28
7.28
7.27
7.26 CDCl3
7.26
7.26
7.25
7.12
7.11
7.10
7.08
7.08
7.07
7.06
7.06
5.24
5.22
5.22
5.20
3.77
3.75
3.75
3.73
3.72
3.70
3.67
3.65
3.60
3.59
3.58
3.50
3.49
3.49
3.48
3.48
3.47
3.46
3.33
3.32
3.31
3.30
3.30
3.30
3.28
3.28
3.27
3.19
3.18
3.17
3.17
3.16
2.40
2.31
1.76
1.75
1.75
1.74
1.74
1.73
1.55
1.54
1.53
1.52
1.51
1.50
O
N
O
NHTs
Me
4k
2.04
1.09
1.09
4.19
0.99
4.21
2.05
2.10
2.07
1.01
3.05
3.05
2.13
2.10
10.5 10.0 9.5 9.0 8.5 8.0 7.5 7.0 6.5 6.0 5.5 5.0 4.5 4.0 3.5 3.0 2.5 2.0 1.5 1.0 0.5 0.0 -0.5 -1
f1 (ppm)

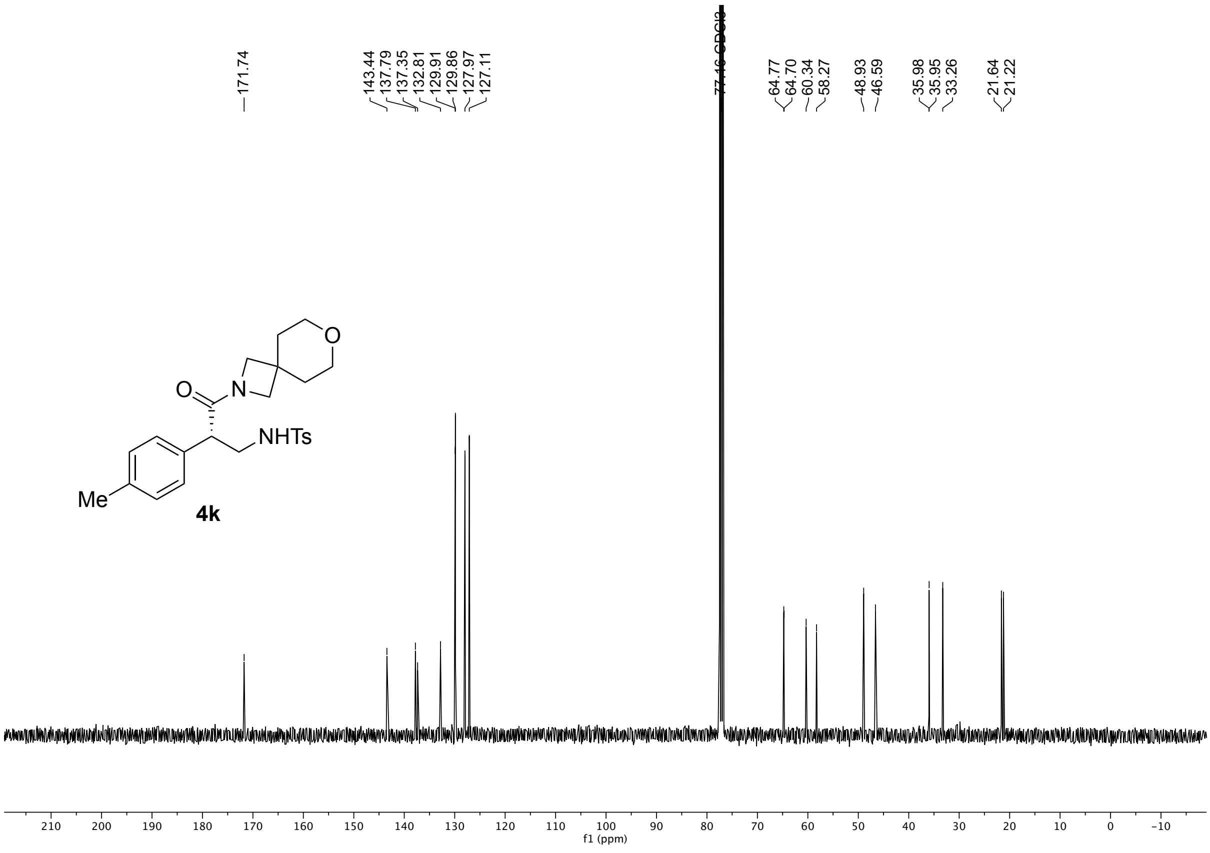
171.74
143.44
137.79
137.35
132.81
129.91
129.86
127.97
127.11
77.16 CDCl3
64.77
64.70
60.34
58.27
48.93
46.59
35.98
35.95
33.26
21.64
21.22
O
N
O
NHTs
Me
4k
210 200 190 180 170 160 150 140 130 120 110 100 90 80 70 60 50 40 30 20 10 0 -10
f1 (ppm)

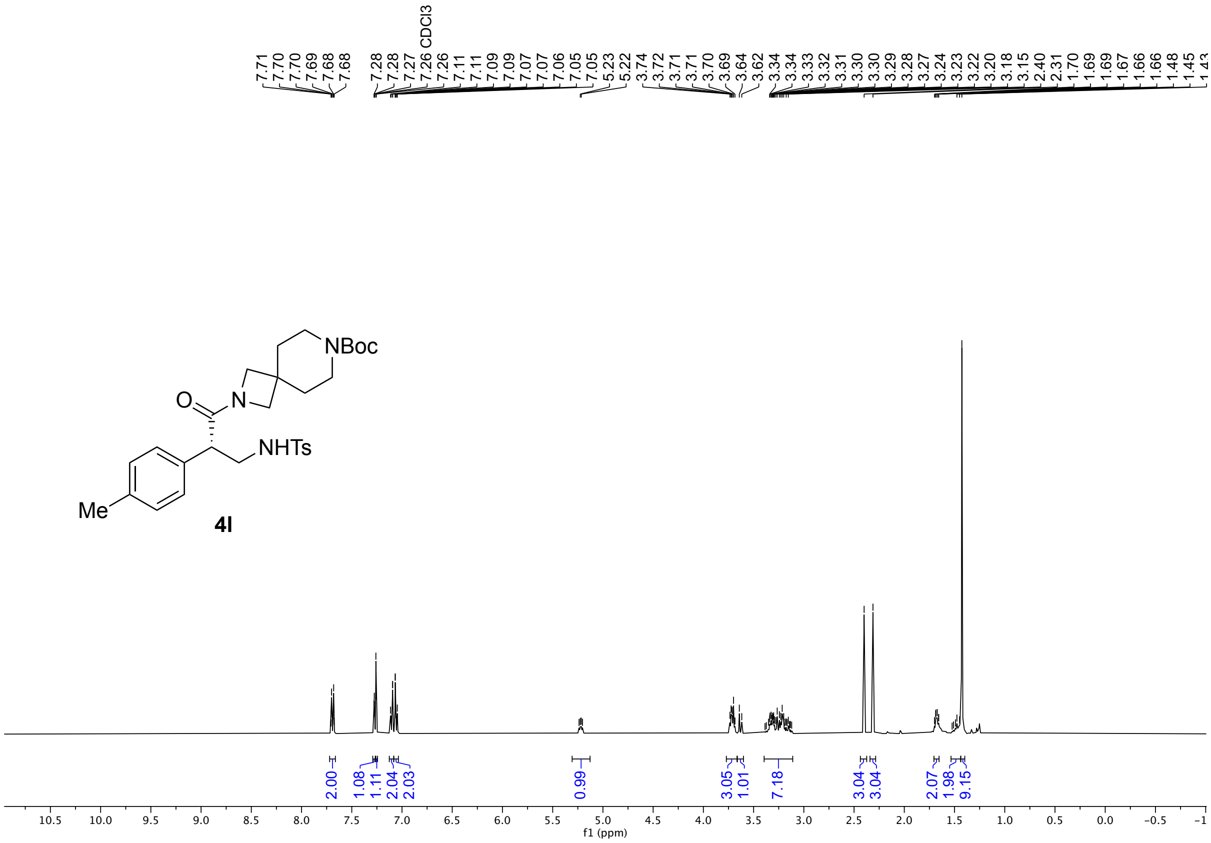
7.71
7.70
7.70
7.69
7.68
7.68
7.28
7.28
7.27
7.26 CDCl3
7.26
7.11
7.11
7.09
7.07
7.07
7.06
7.05
5.23
5.22
3.74
3.72
3.71
3.71
3.70
3.69
3.64
3.62
3.34
3.34
3.33
3.32
3.31
3.30
3.30
3.29
3.28
3.27
3.24
3.23
3.22
3.20
3.18
3.15
2.40
2.31
1.70
1.69
1.69
1.67
1.66
1.66
1.48
1.45
NBoc
O
N
NHTs
Me
4l
2.00
1.08
1.11
2.04
2.03
0.99
3.05
1.01
7.18
3.04
3.04
2.07
1.98
9.15
f1 (ppm)

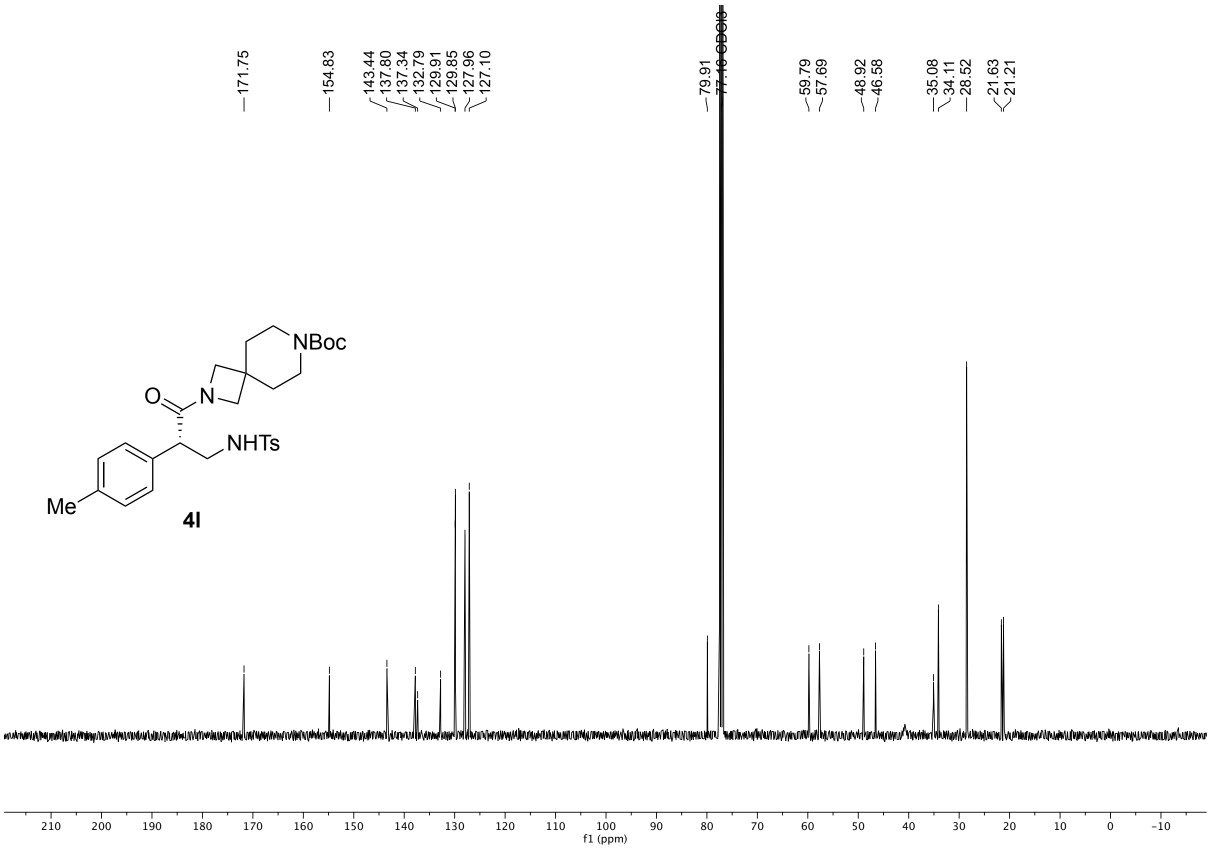
171.75
154.83
143.44
137.80
137.34
132.79
129.91
129.85
127.96
127.10
79.91
77.16 CDCl3
59.79
57.69
48.92
46.58
35.08
34.11
28.52
21.63
21.21
NBoc
O
N
NHTs
Me
4l
f1 (ppm)

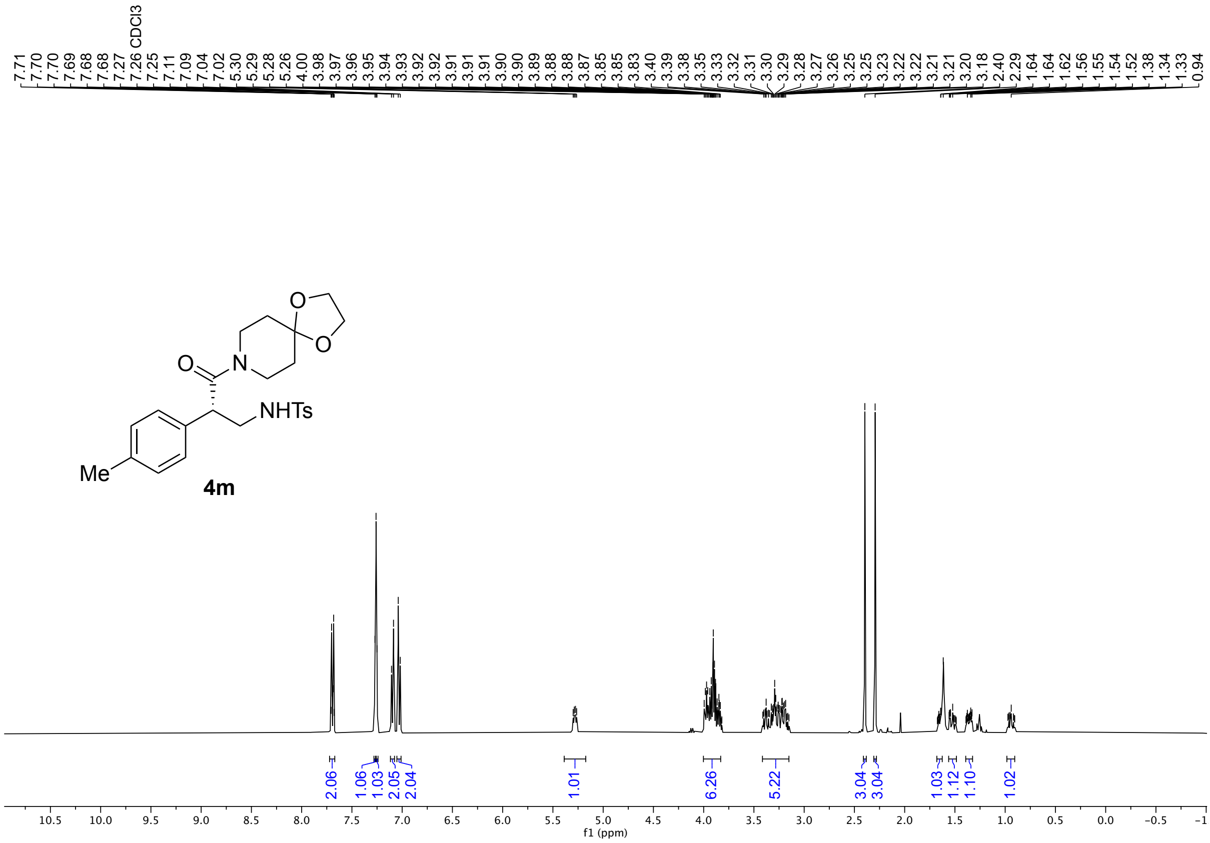
7.71 7.70 7.70 7.69 7.68 7.68 7.27 7.26 CDCl3 7.25 7.11 7.09 7.04 7.02 5.30 5.29 5.28 5.26 4.00 3.98 3.97 3.96 3.95 3.94 3.93 3.93 3.92 3.92 3.91 3.91 3.91 3.90 3.90 3.89 3.88 3.88 3.87 3.85 3.85 3.83 3.40 3.39 3.38 3.35 3.33 3.32 3.31 3.30 3.29 3.28 3.27 3.26 3.25 3.25 3.23 3.22 3.22 3.21 3.21 3.20 3.18 2.40 2.29 1.64 1.64 1.62 1.56 1.55 1.54 1.52 1.38 1.34 1.33 0.94
O
N
O
O
NHTs
Me
4m
2.06
1.06
1.03
2.05
2.04
1.01
6.26
5.22
3.04
3.04
1.03
1.12
1.10
1.02
f1 (ppm)

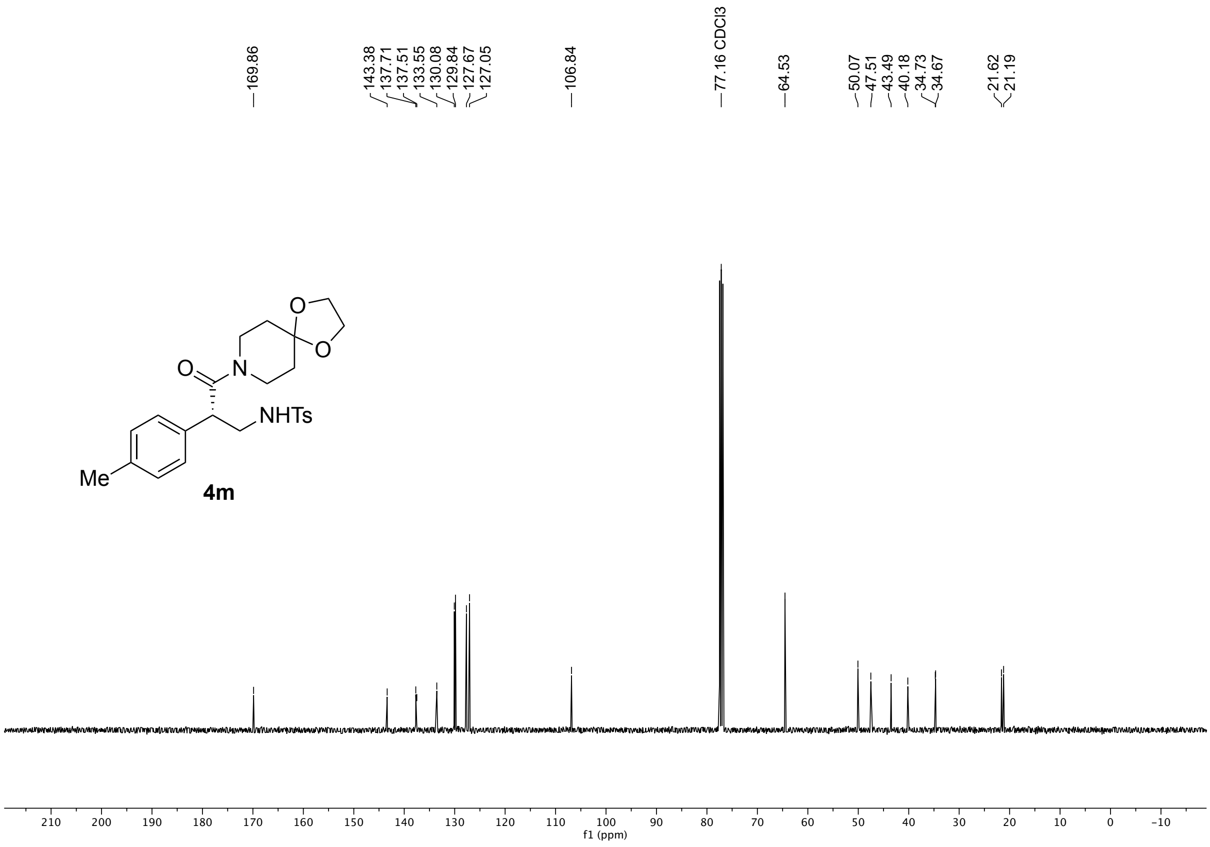
169.86
143.38 137.71 137.51 133.55 130.08 129.84 127.67 127.05
106.84
77.16 CDCl3
64.53
50.07 47.51 43.49 40.18 34.73 34.67
21.62 21.19
O
N
O
O
NHTs
Me
4m
f1 (ppm)

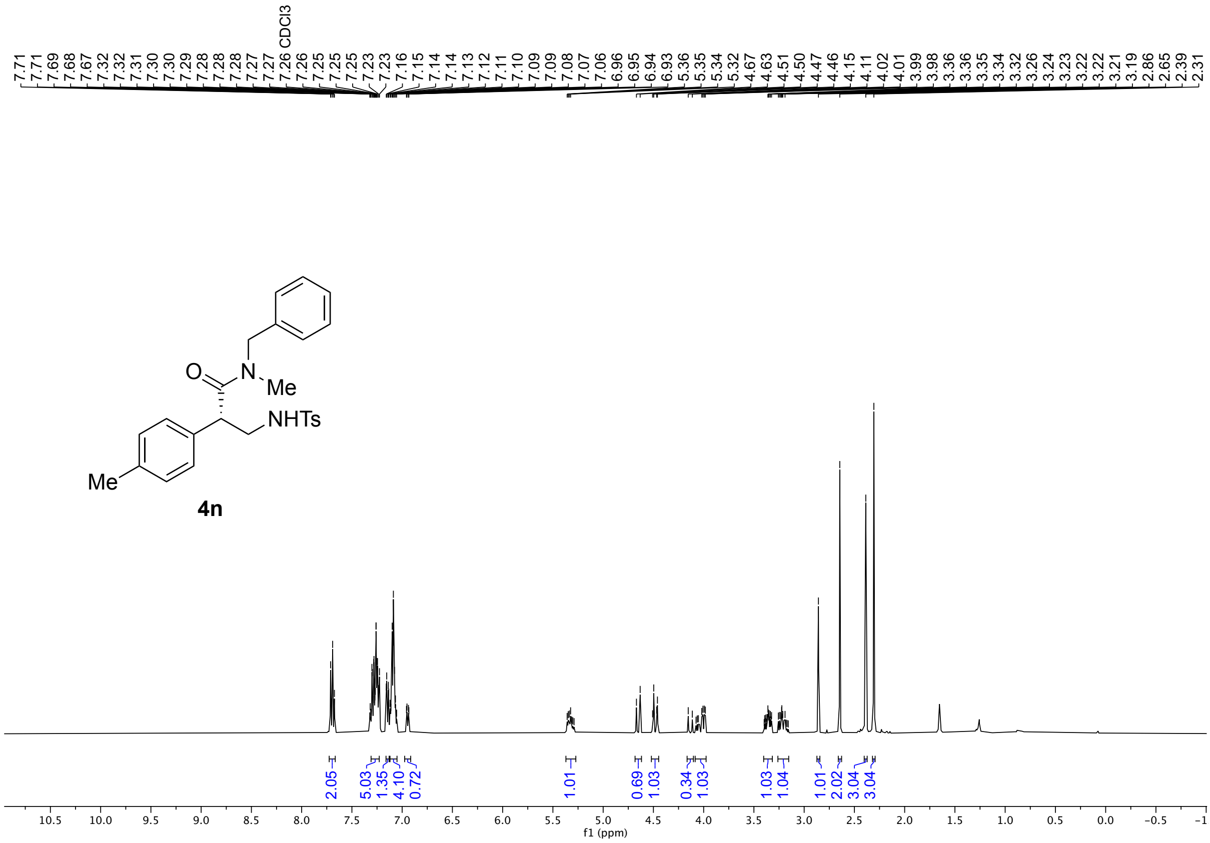
7.71 7.71 7.69 7.68 7.67 7.32 7.32 7.31 7.30 7.30 7.29 7.28 7.28 7.28 7.27 7.26 CDCl3 7.26 7.25 7.25 7.25 7.23 7.23 7.16 7.15 7.14 7.14 7.13 7.12 7.11 7.10 7.09 7.09 7.08 7.07 7.06 6.96 6.95 6.94 6.93 5.36 5.35 5.34 5.32 4.67 4.63 4.51 4.50 4.47 4.46 4.15 4.11 4.02 4.01 3.99 3.98 3.36 3.36 3.35 3.34 3.32 3.26 3.24 3.23 3.22 3.22 3.21 3.19 2.86 2.65 2.39 2.31
O
N
Me
NHTs
Me
4n
2.05 5.03 1.35 4.10 0.72 1.01 0.69 1.03 0.34 1.03 1.03 1.04 1.01 2.02 3.04 3.04
10.5 10.0 9.5 9.0 8.5 8.0 7.5 7.0 6.5 6.0 5.5 5.0 4.5 4.0 3.5 3.0 2.5 2.0 1.5 1.0 0.5 0.0 −0.5 −1
f1 (ppm)

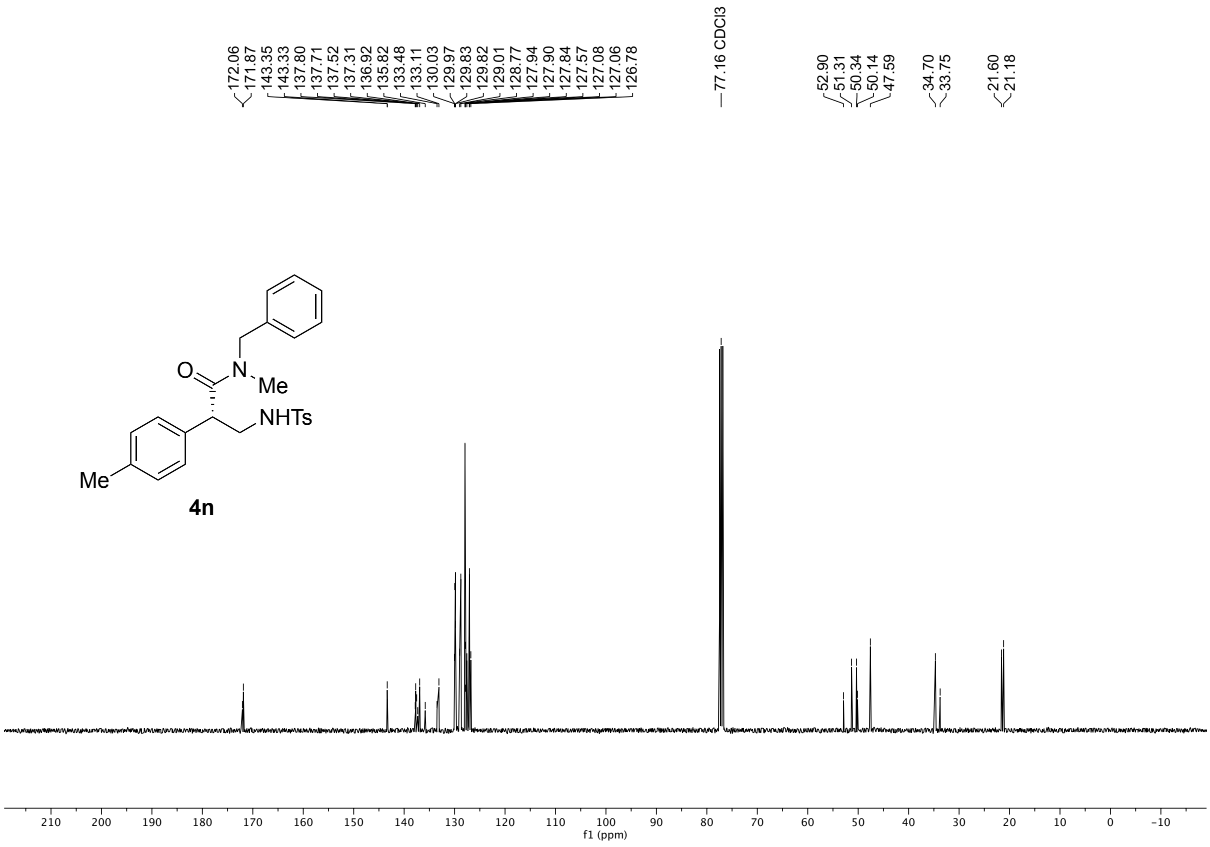
172.06 171.87 143.35 143.33 137.80 137.71 137.52 137.31 136.92 135.82 133.48 133.11 130.03 129.97 129.83 129.82 129.01 128.77 127.94 127.90 127.84 127.57 127.08 127.06 126.78
77.16 CDCl3
52.90 51.31 50.34 50.14 47.59 34.70 33.75 21.60 21.18
O
N
Me
NHTs
Me
4n
210 200 190 180 170 160 150 140 130 120 110 100 90 80 70 60 50 40 30 20 10 0 −10
f1 (ppm)

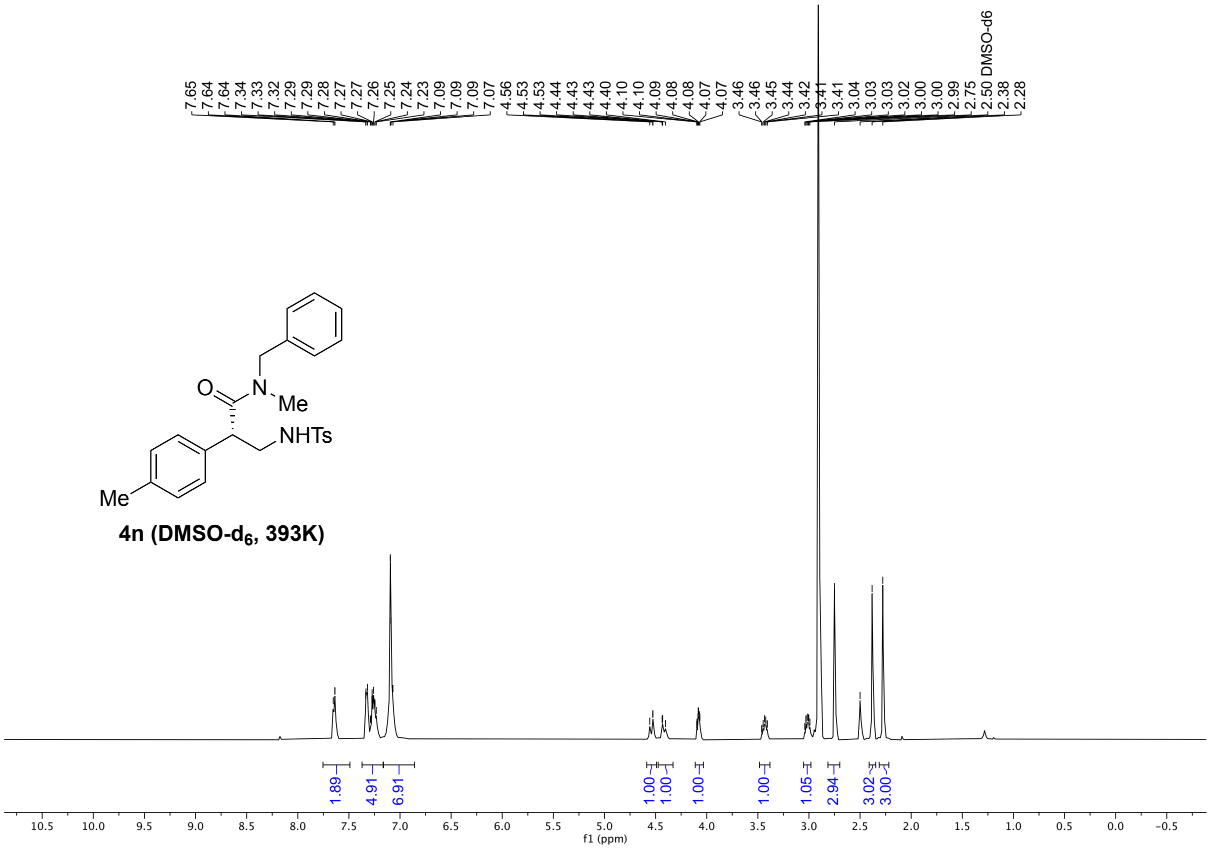
7.65 7.64 7.34 7.33 7.32 7.29 7.29 7.28 7.27 7.27 7.26 7.25 7.24 7.23 7.09 7.09 7.09 7.07 4.56 4.53 4.53 4.44 4.43 4.43 4.40 4.10 4.09 4.08 4.08 4.07 4.07 3.46 3.46 3.45 3.44 3.42 3.41 3.41 3.04 3.03 3.03 3.02 3.00 3.00 2.99 2.75 2.50 DMSO-d6 2.38 2.28
O
N
Me
NHTs
Me
4n (DMSO-d6, 393K)
1.89 4.91 6.91 1.00 1.00 1.00 1.00 1.05 2.94 3.02 3.00
f1 (ppm)

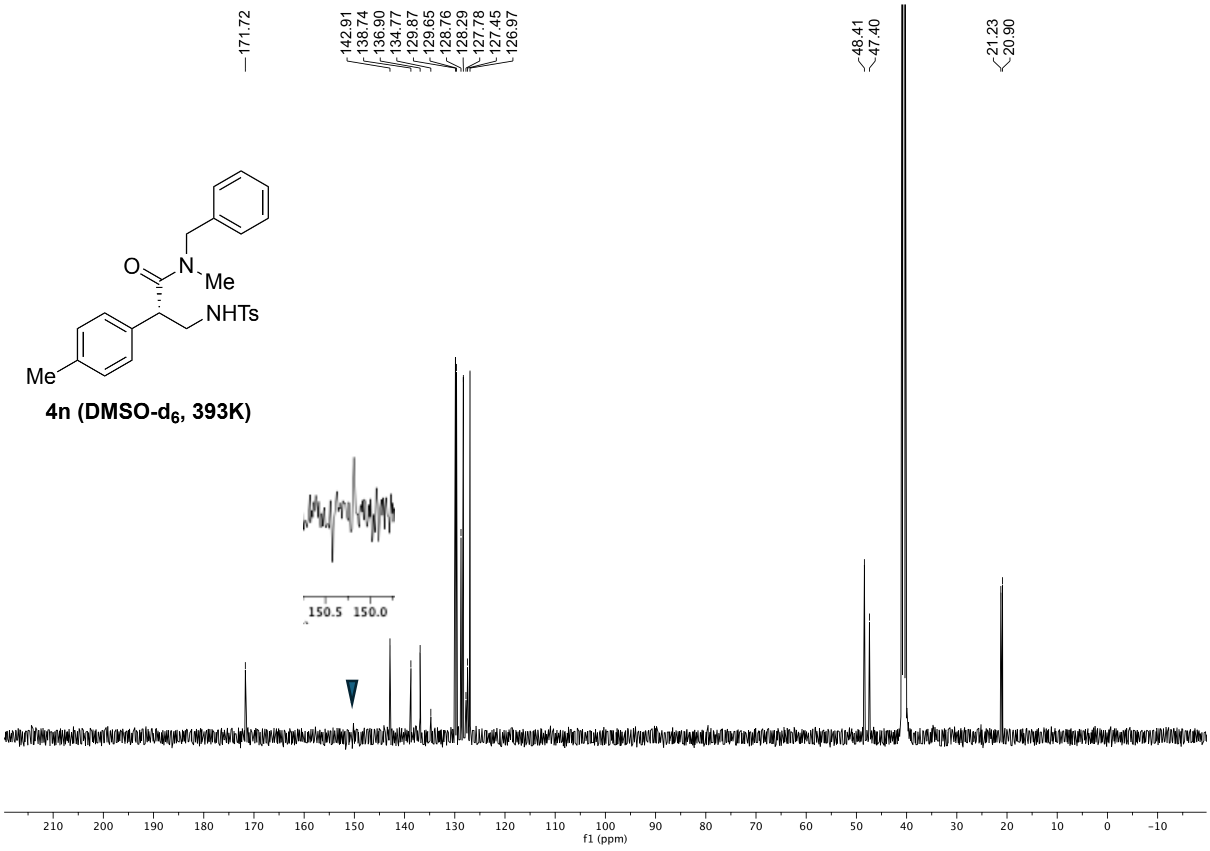
171.72
142.91 138.74 136.90 134.77 129.87 129.65 128.76 128.29 127.78 127.45 126.97
48.41 47.40
21.23 20.90
O
N
Me
NHTs
Me
4n (DMSO-d6, 393K)
150.5 150.0
f1 (ppm)

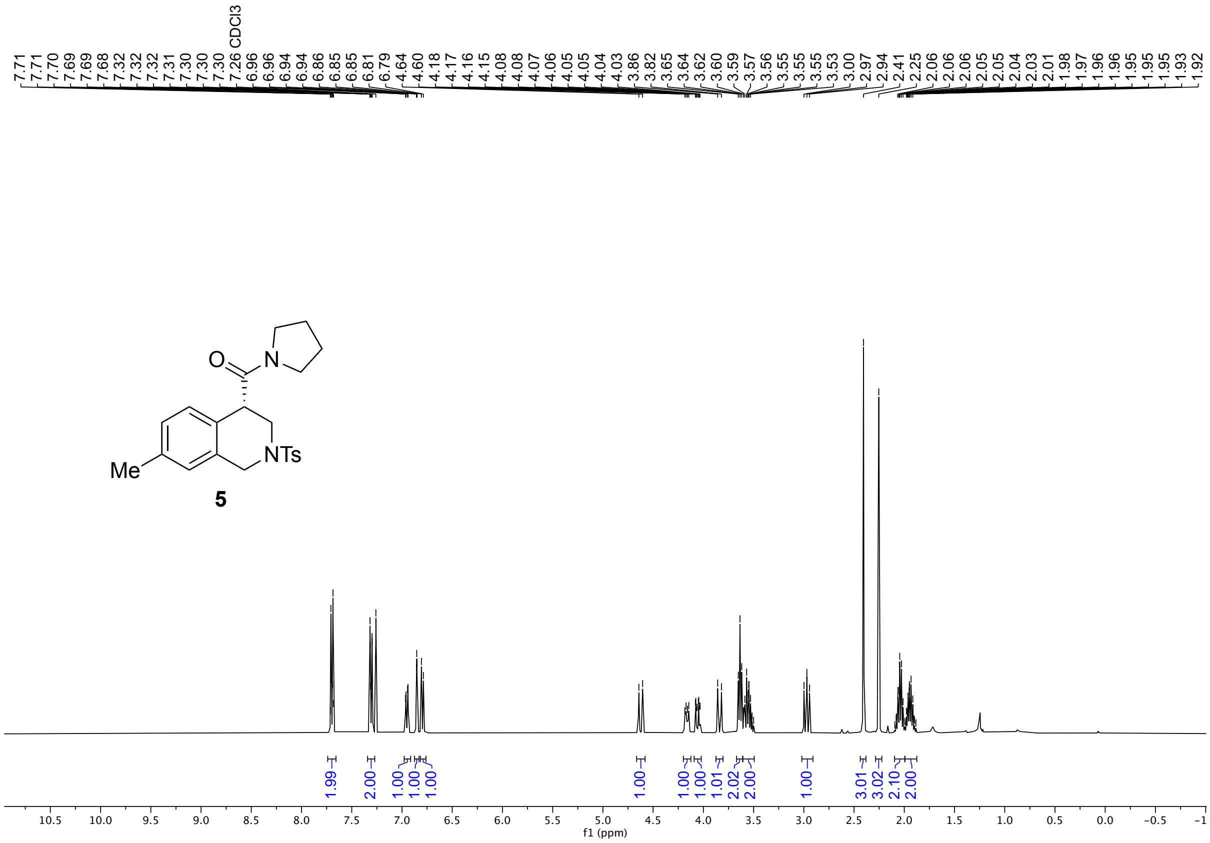
7.71 7.71 7.70 7.69 7.69 7.68 7.32 7.32 7.32 7.31 7.30 7.30 7.30 7.26 CDCl3 6.96 6.96 6.94 6.94 6.86 6.85 6.85 6.81 6.79 4.64 4.60 4.18 4.17 4.16 4.15 4.08 4.08 4.07 4.06 4.05 4.05 4.04 4.03 3.86 3.82 3.65 3.64 3.62 3.60 3.59 3.57 3.56 3.55 3.55 3.55 3.53 3.00 2.97 2.94 2.41 2.25 2.06 2.06 2.06 2.05 2.05 2.04 2.03 2.01 1.98 1.97 1.96 1.96 1.95 1.95 1.95 1.93 1.92
O
N
NTs
Me
5
1.99 2.00 1.00 1.00 1.00 1.00 1.00 1.00 1.01 2.02 2.00 1.00 3.01 3.02 2.10 2.00
10.5 10.0 9.5 9.0 8.5 8.0 7.5 7.0 6.5 6.0 5.5 5.0 4.5 4.0 3.5 3.0 2.5 2.0 1.5 1.0 0.5 0.0 −0.5 −1
f1 (ppm)

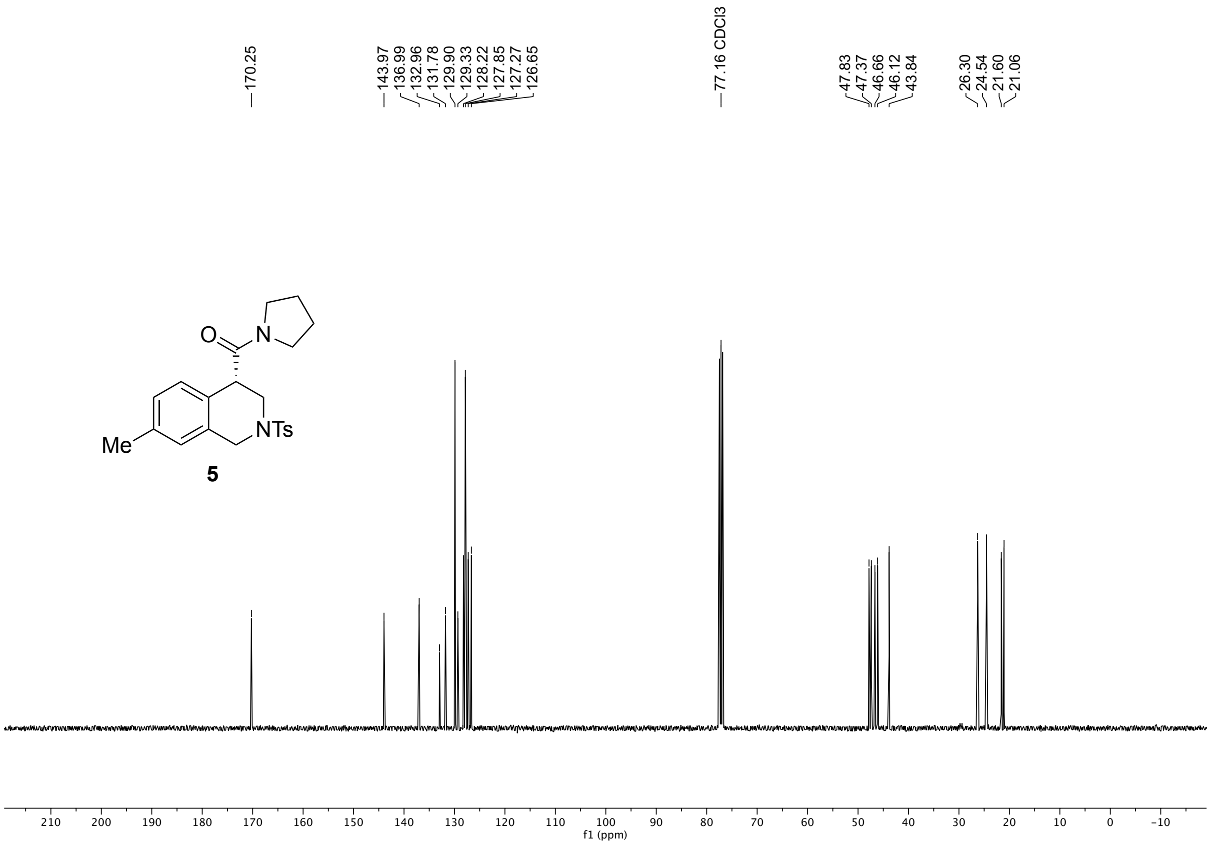
170.25
143.97 136.99 132.96 131.78 129.90 129.33 128.22 127.85 127.27 126.65
77.16 CDCl3
47.83 47.37 46.66 46.12 43.84
26.30 24.54 21.60 21.06
O
N
NTs
Me
5
210 200 190 180 170 160 150 140 130 120 110 100 90 80 70 60 50 40 30 20 10 0 −10
f1 (ppm)

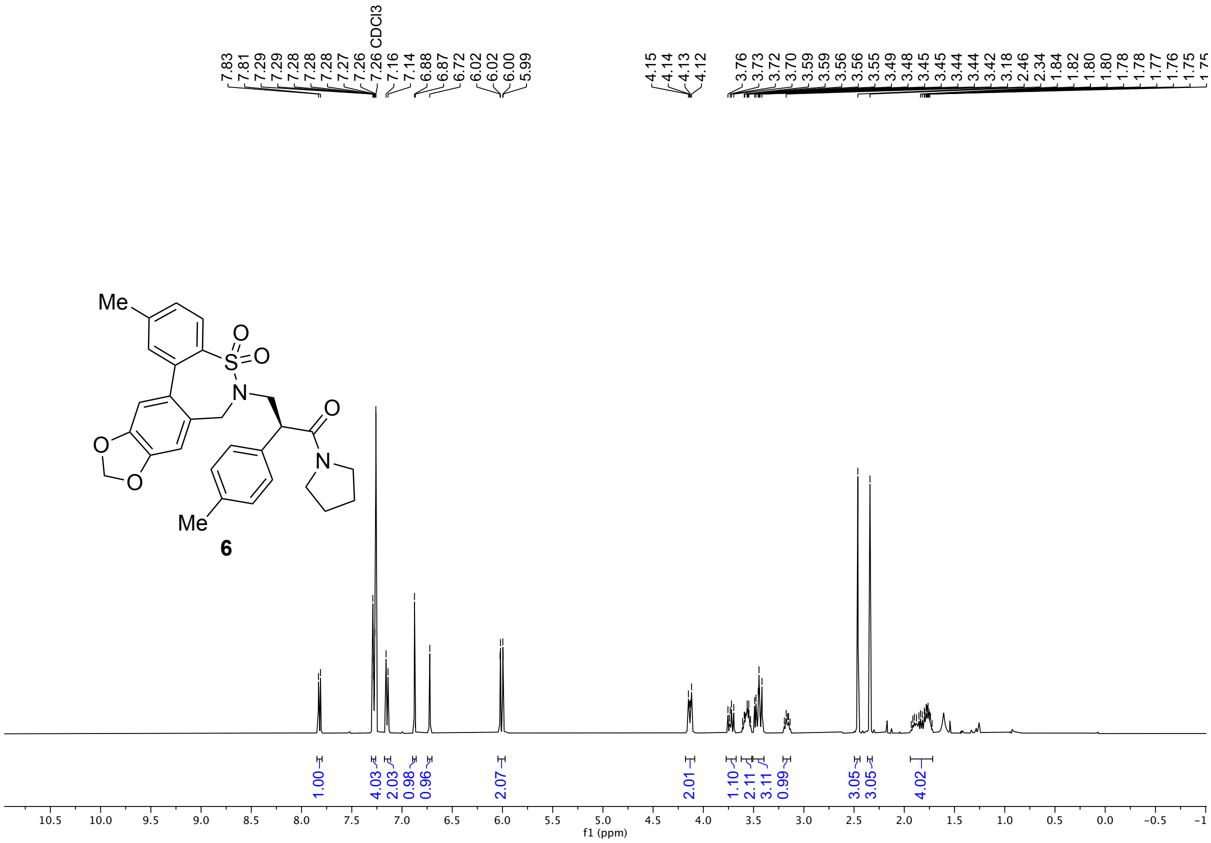
7.83
7.81
7.29
7.29
7.28
7.28
7.28
7.27
7.26 CDCl3
7.16
7.14
6.88
6.87
6.72
6.02
6.02
6.00
5.99
4.15
4.14
4.13
4.12
3.76
3.73
3.72
3.70
3.59
3.59
3.56
3.56
3.55
3.49
3.48
3.45
3.45
3.44
3.44
3.42
3.18
2.46
2.34
1.84
1.82
1.80
1.80
1.78
1.78
1.77
1.76
1.75
Me
O
S
O
N
O
O
O
N
Me
6
1.00
4.03
2.03
0.98
0.96
2.07
2.01
1.10
2.11
3.11
0.99
3.05
3.05
4.02
f1 (ppm)

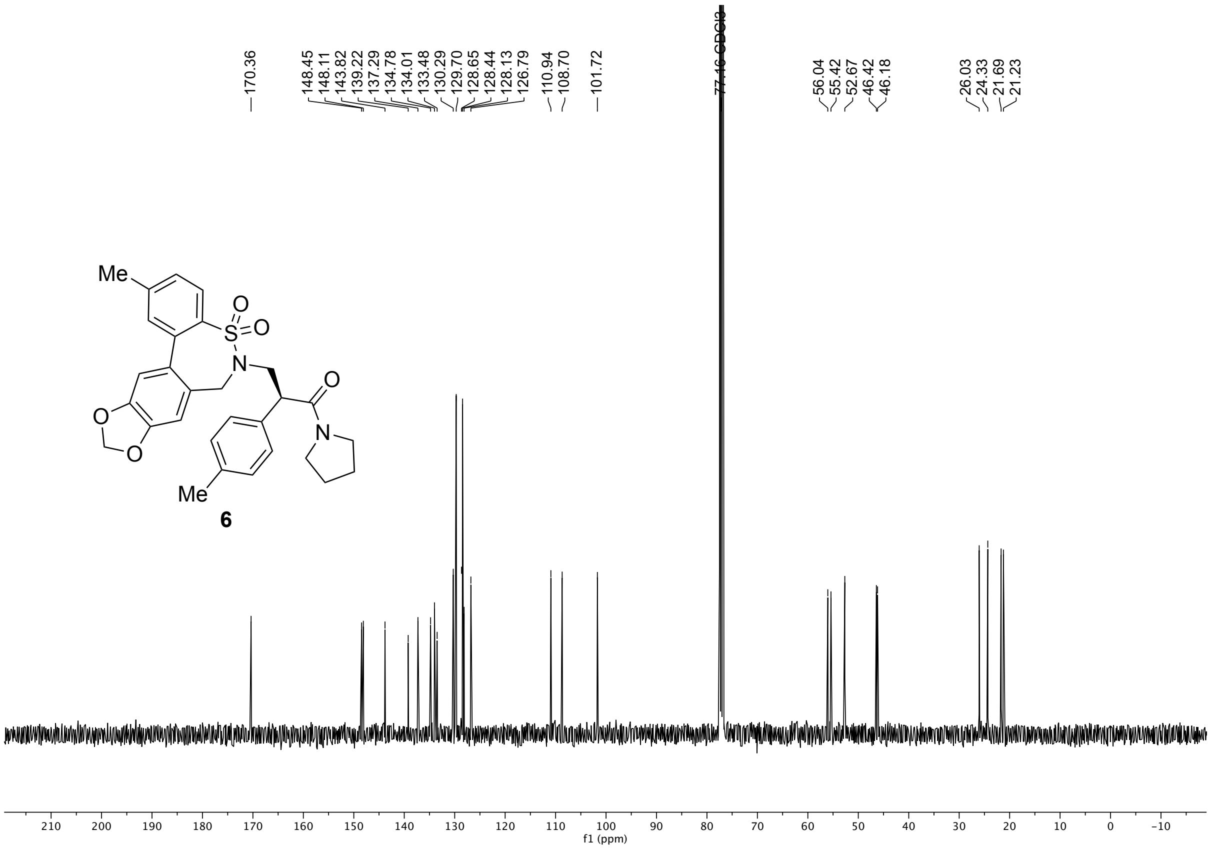
170.36
148.45
148.11
143.82
139.22
137.29
134.78
134.01
133.48
130.29
129.70
128.65
128.44
128.13
126.79
110.94
108.70
101.72
77.16 CDCl3
56.04
55.42
52.67
46.42
46.18
26.03
24.33
21.69
21.23
Me
O
S
O
N
O
O
O
N
Me
6
f1 (ppm)

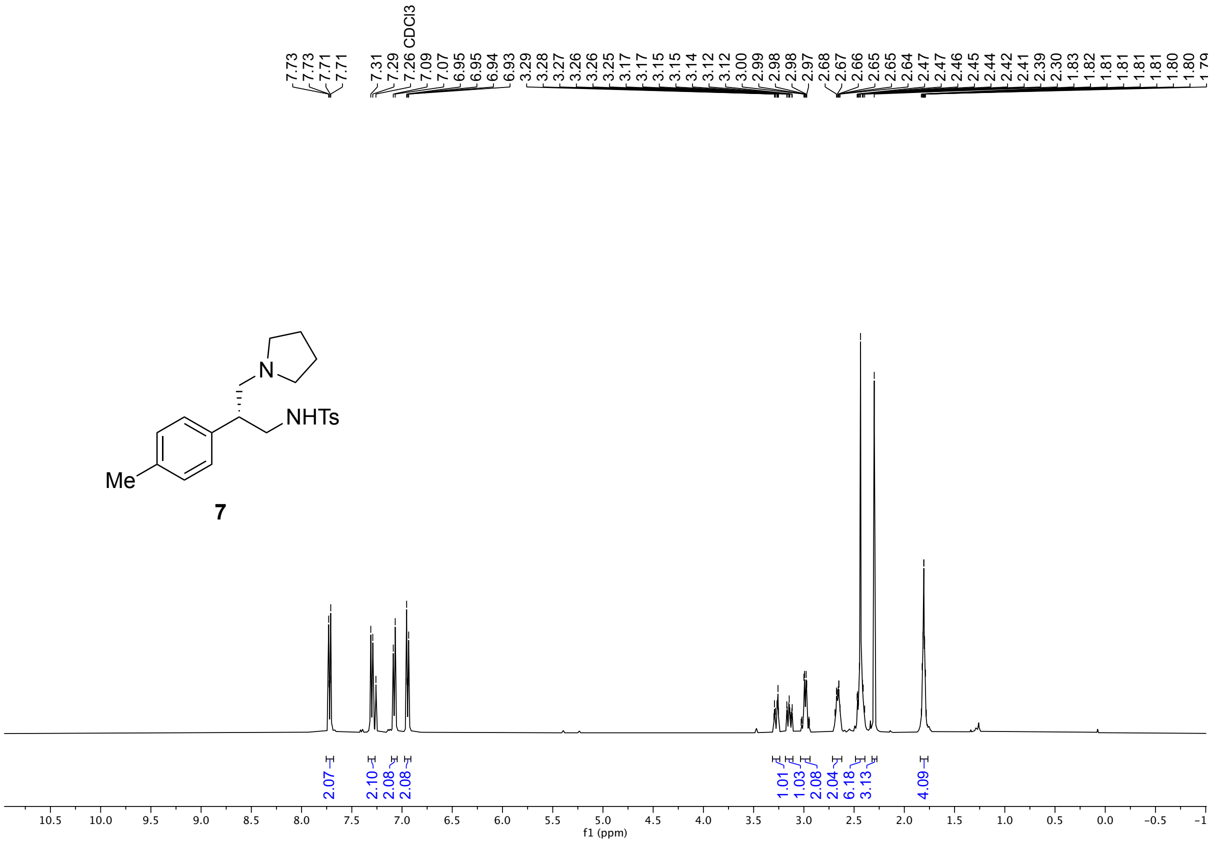
7.73 7.73 7.71 7.71 7.31 7.29 7.26 CDCl3 7.09 7.07 6.95 6.95 6.94 6.93 3.29 3.28 3.27 3.26 3.25 3.17 3.15 3.15 3.14 3.12 3.00 2.99 2.98 2.98 2.97 2.68 2.67 2.66 2.65 2.65 2.64 2.47 2.46 2.45 2.44 2.42 2.41 2.39 2.30 1.83 1.82 1.81 1.81 1.81 1.81 1.80 1.80 1.79
N
NHTs
Me
7
2.07 2.10 2.08 2.08 1.01 1.03 2.08 2.04 6.18 3.13 4.09
f1 (ppm)

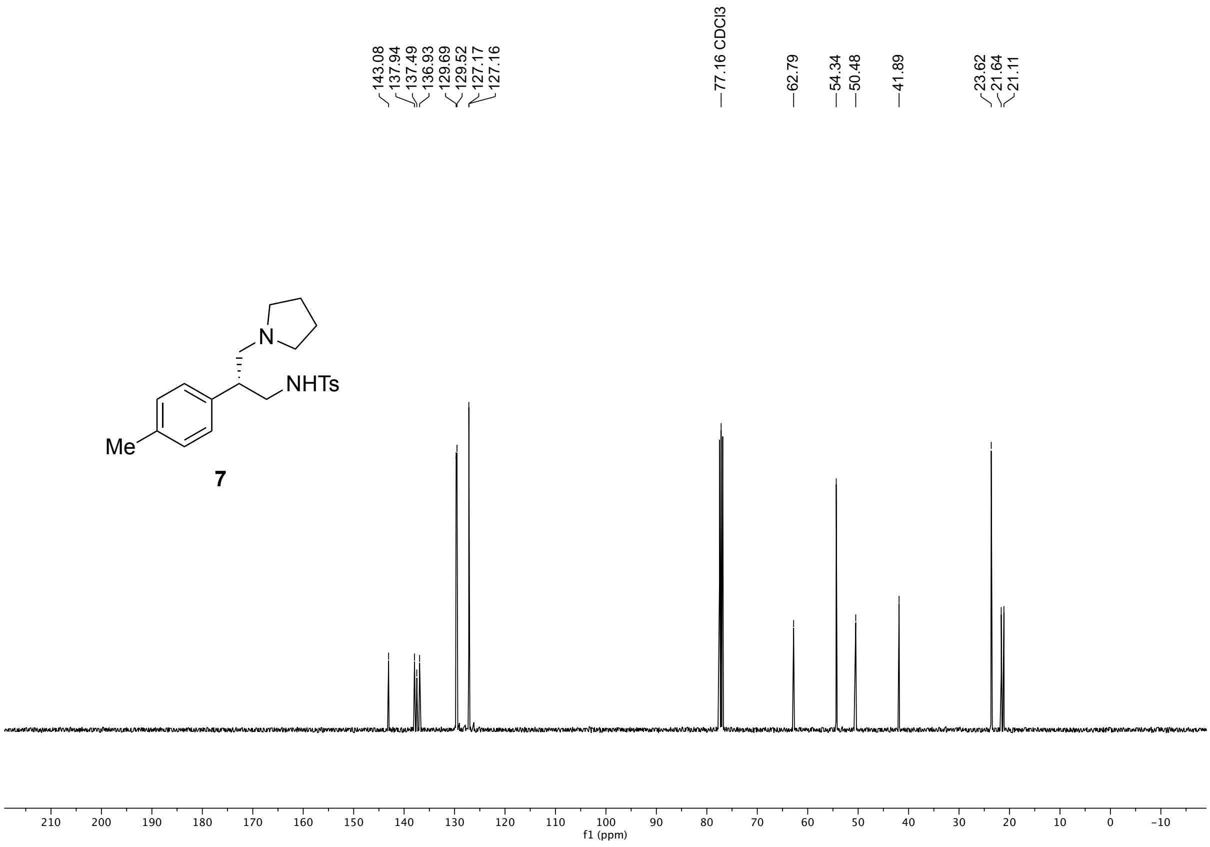
143.08 137.94 137.49 136.93 129.69 129.52 127.17 127.16
77.16 CDCl3
62.79
54.34
50.48
41.89
23.62 21.64 21.11
N
NHTs
Me
7
f1 (ppm)

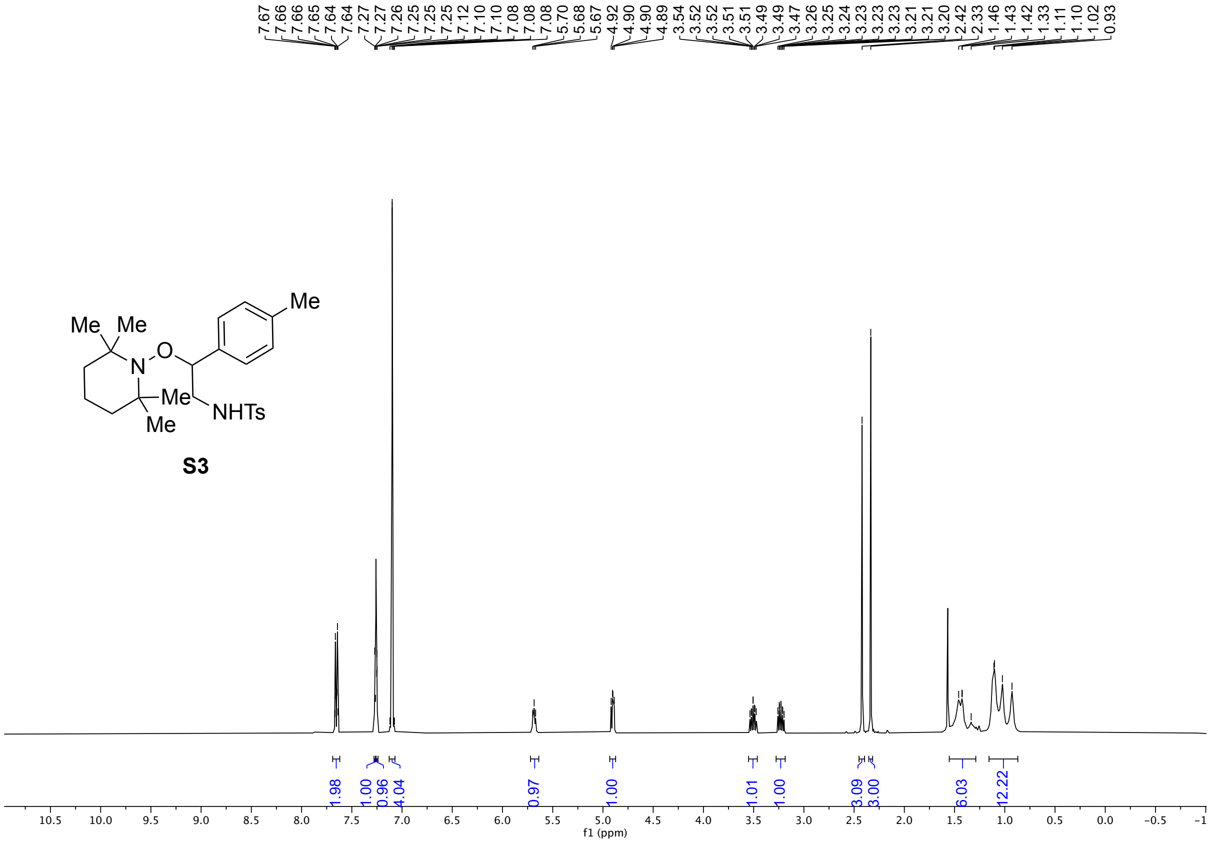
Me
Me
Me
N
O
Me
Me
NHTs
S3
f1 (ppm)

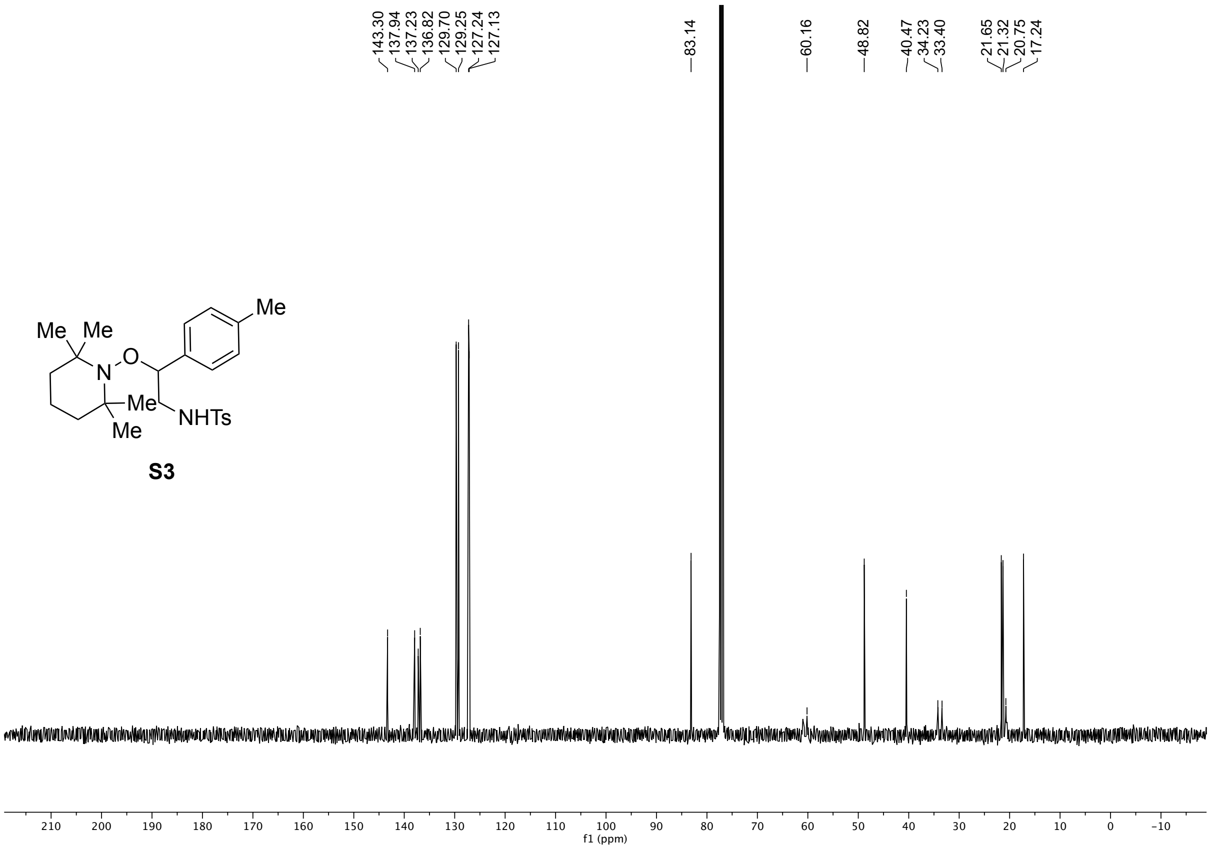
Me
Me
Me
N
O
Me
Me
NHTs
S3
f1 (ppm)

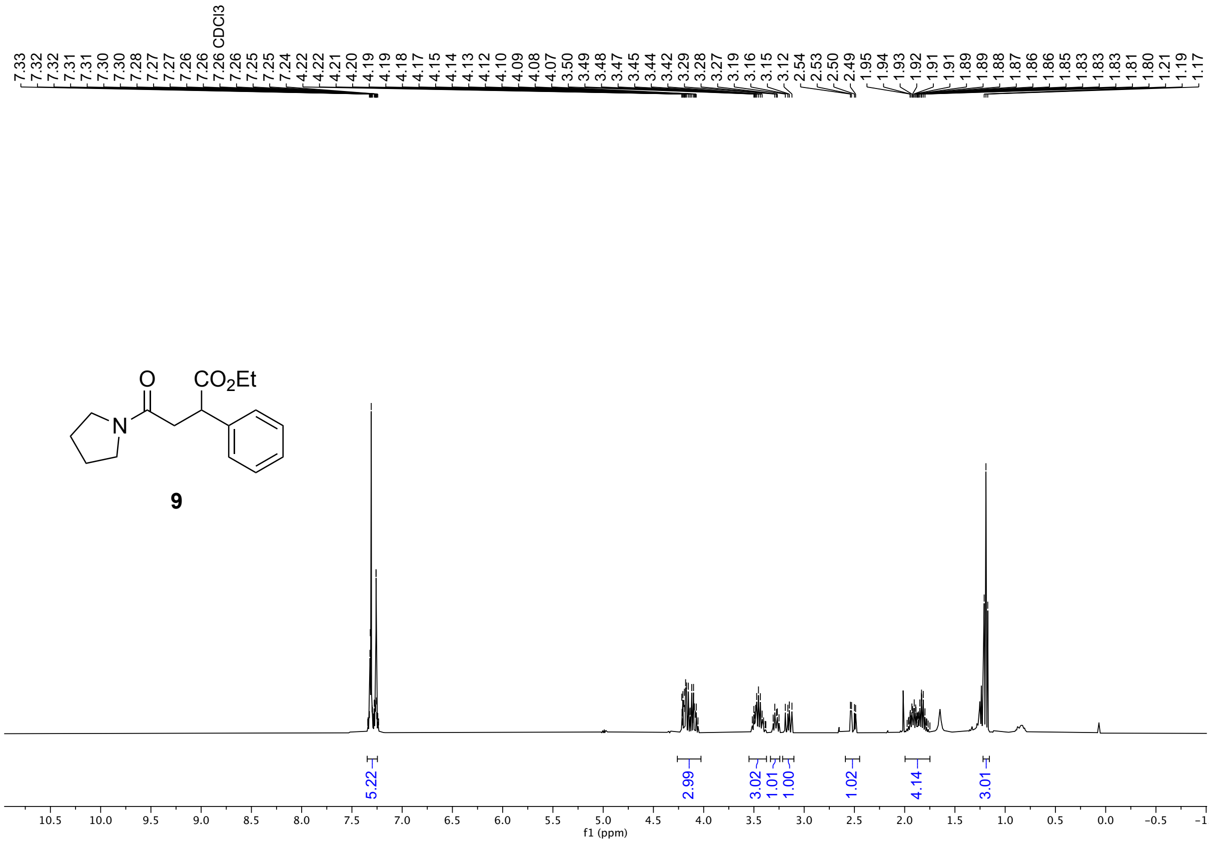

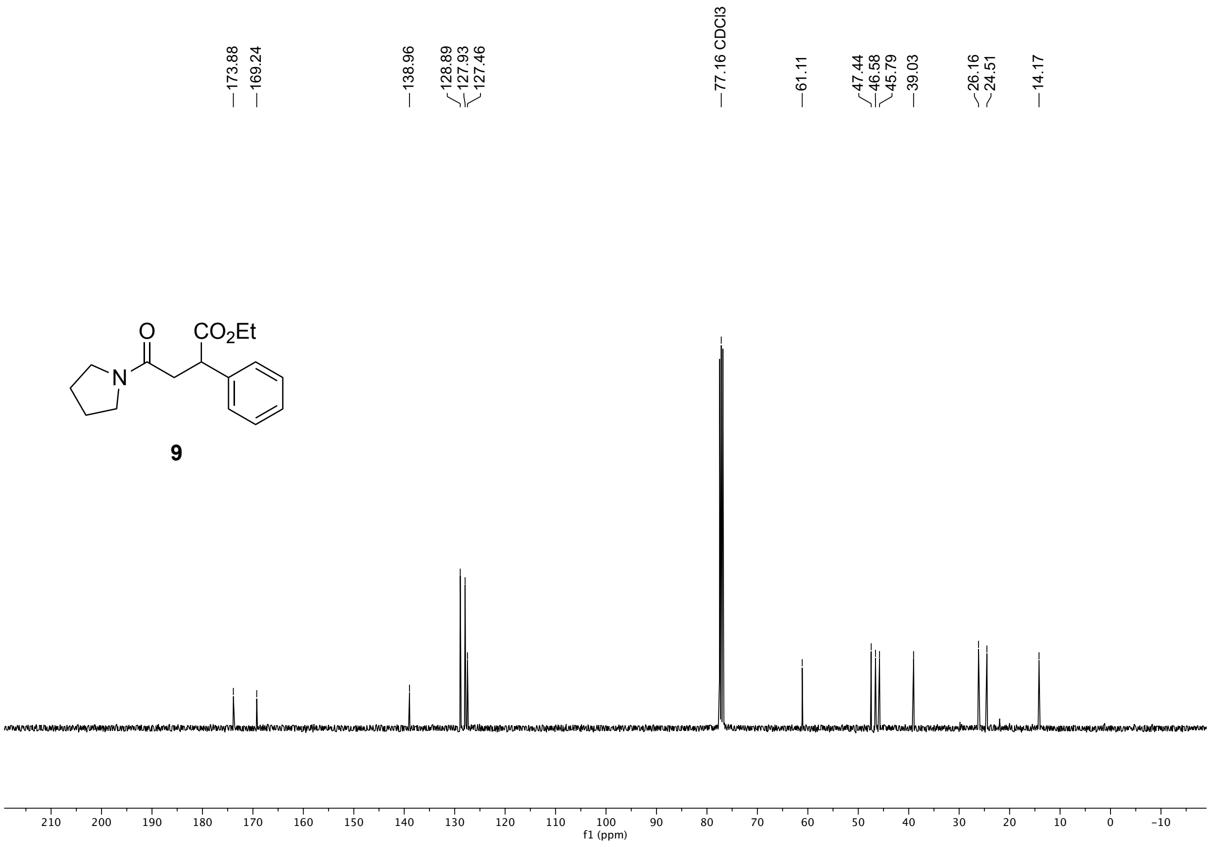

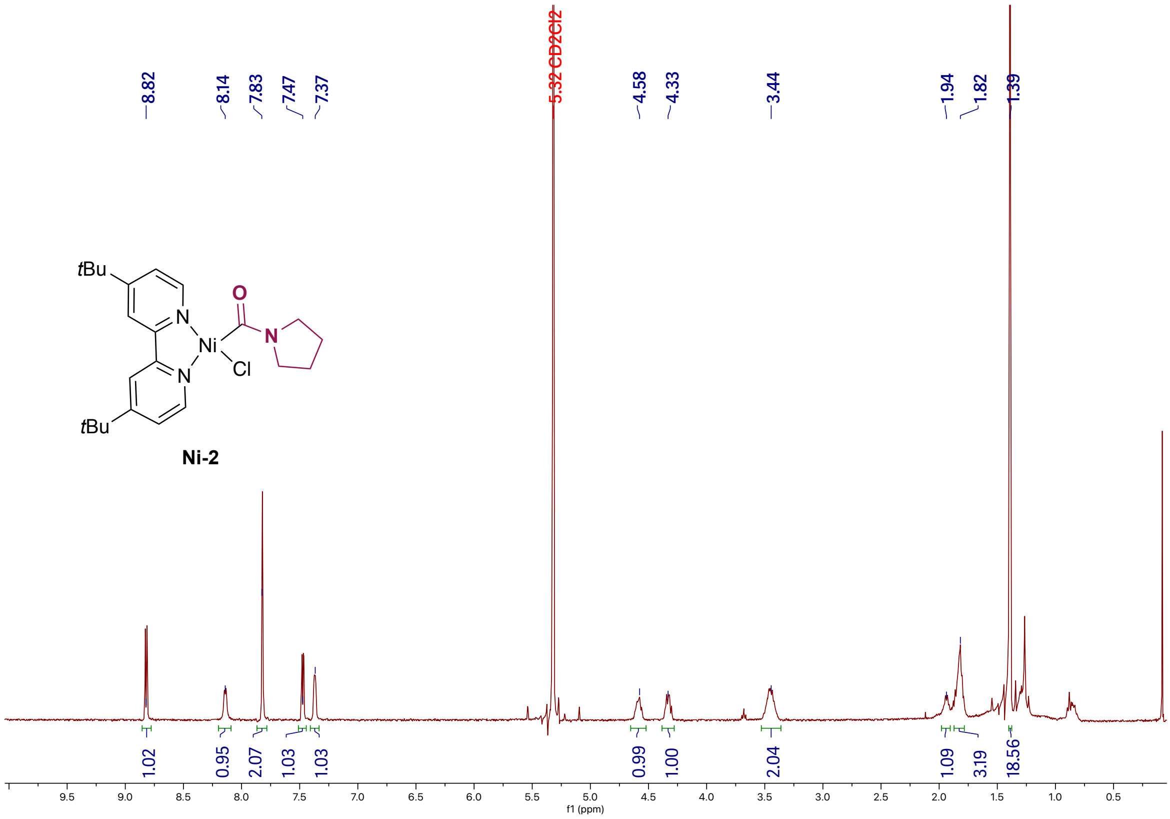
8.82
8.14
7.83
7.47
7.37
5.32 CD2Cl2
4.58
4.33
3.44
1.94
1.82
1.39
tBu
O
N
Ni
Cl
N
N
tBu
Ni-2
1.02
0.95
2.07
1.03
1.03
0.99
1.00
2.04
1.09
3.19
18.56
f1 (ppm)

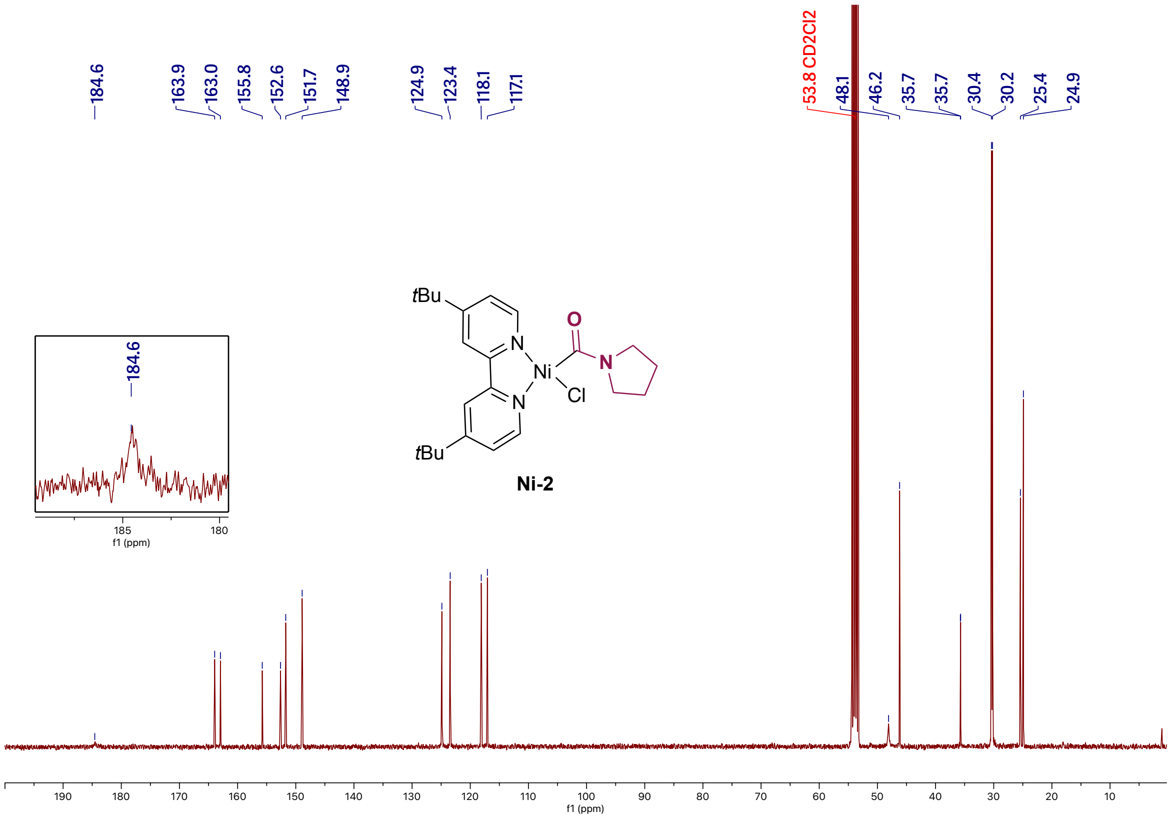
184.6
163.9
163.0
155.8
152.6
151.7
148.9
124.9
123.4
118.1
117.1
53.8 CD2Cl2
48.1
46.2
35.7
35.7
30.4
30.2
25.4
24.9
tBu
O
N
Ni
Cl
N
N
tBu
Ni-2
f1 (ppm)

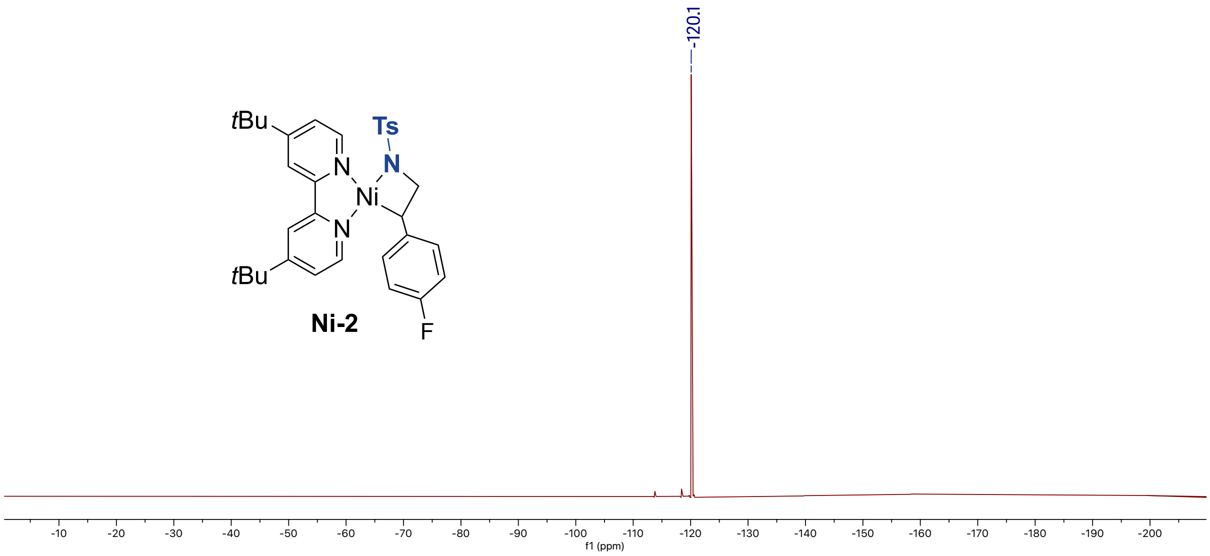
-120.1
tBu
Ts
N
Ni
N
N
tBu
Ni-2
F
f1 (ppm)

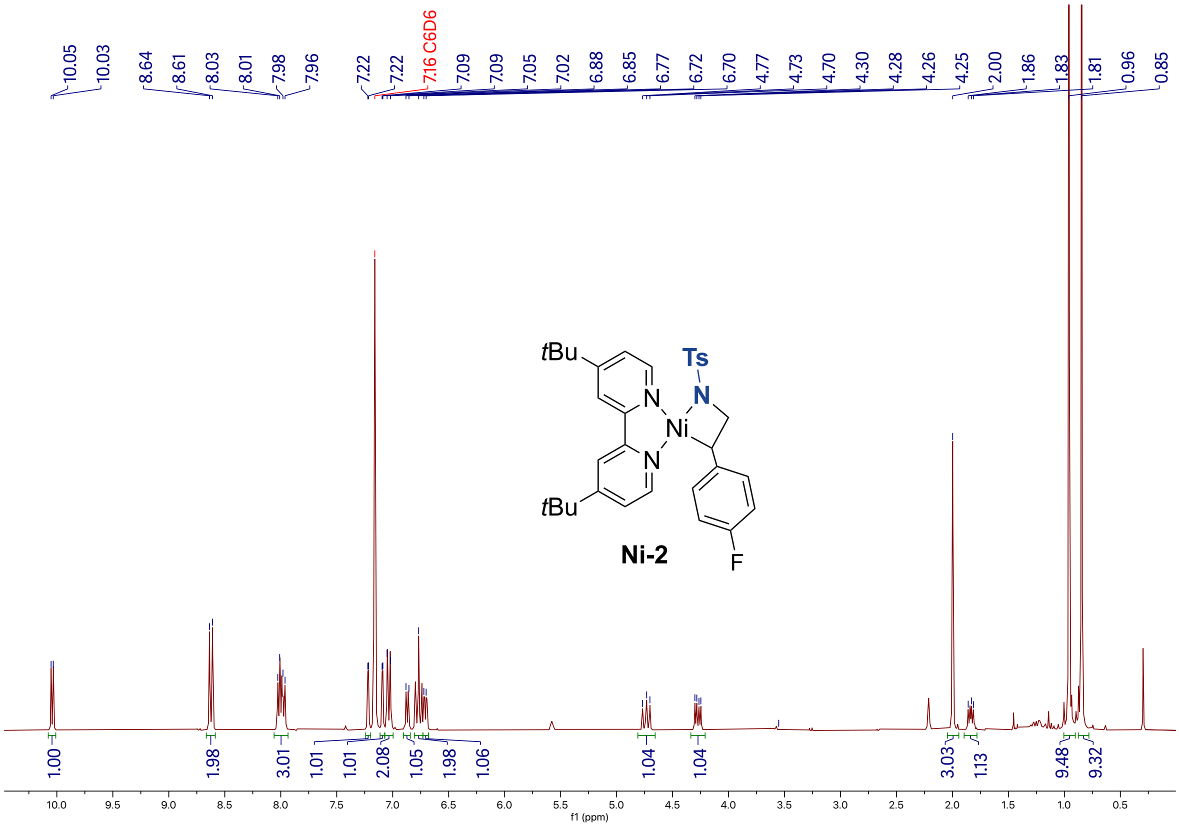
10.05
10.03
8.64
8.61
8.03
8.01
7.98
7.96
7.22
7.22
7.16 C6D6
7.09
7.09
7.05
7.02
6.88
6.85
6.77
6.72
6.70
4.77
4.73
4.70
4.30
4.28
4.26
4.25
2.00
1.86
1.83
1.81
0.96
0.85
tBu
Ts
N
Ni
N
N
tBu
Ni-2
F
1.00
1.98
3.01
1.01
1.01
2.08
1.05
1.98
1.06
1.04
1.04
3.03
1.13
9.48
9.32
f1 (ppm)

Crude **H[1] NMR** for Homocoupling Experiment

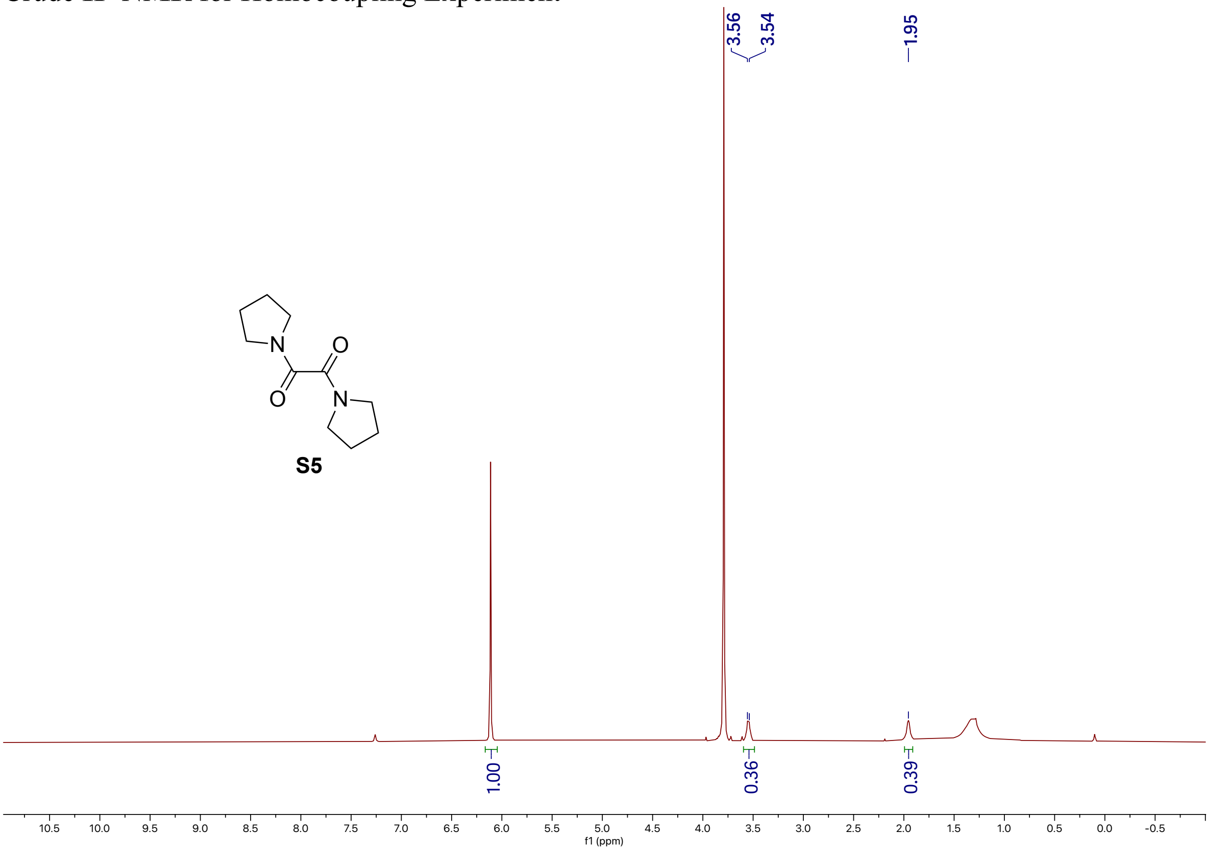


Crude $^{1}$H NMR of stoichiometric reaction between **Ni-2** and **2a**

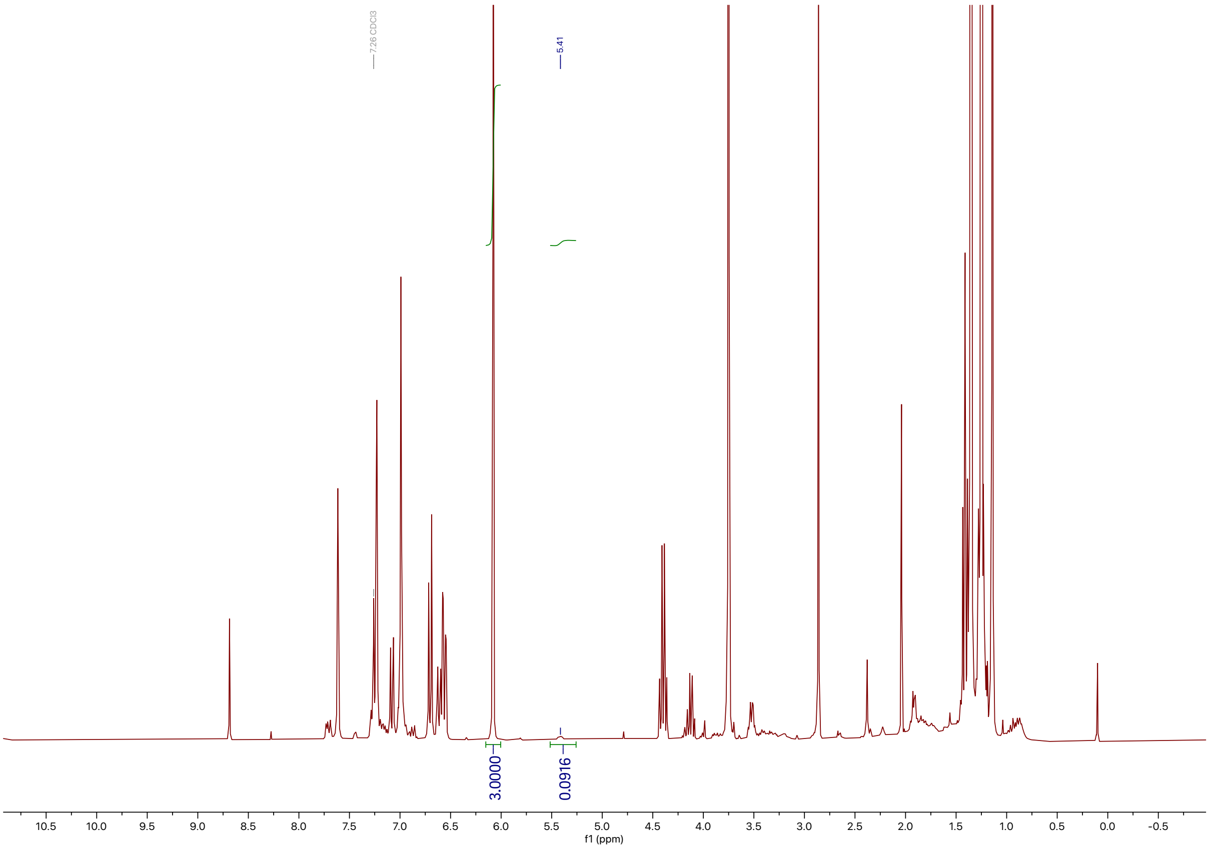


Crude $^{19}$F{$^{1}$H} NMR of stoichiometric reaction between **Ni-2** and **2a**

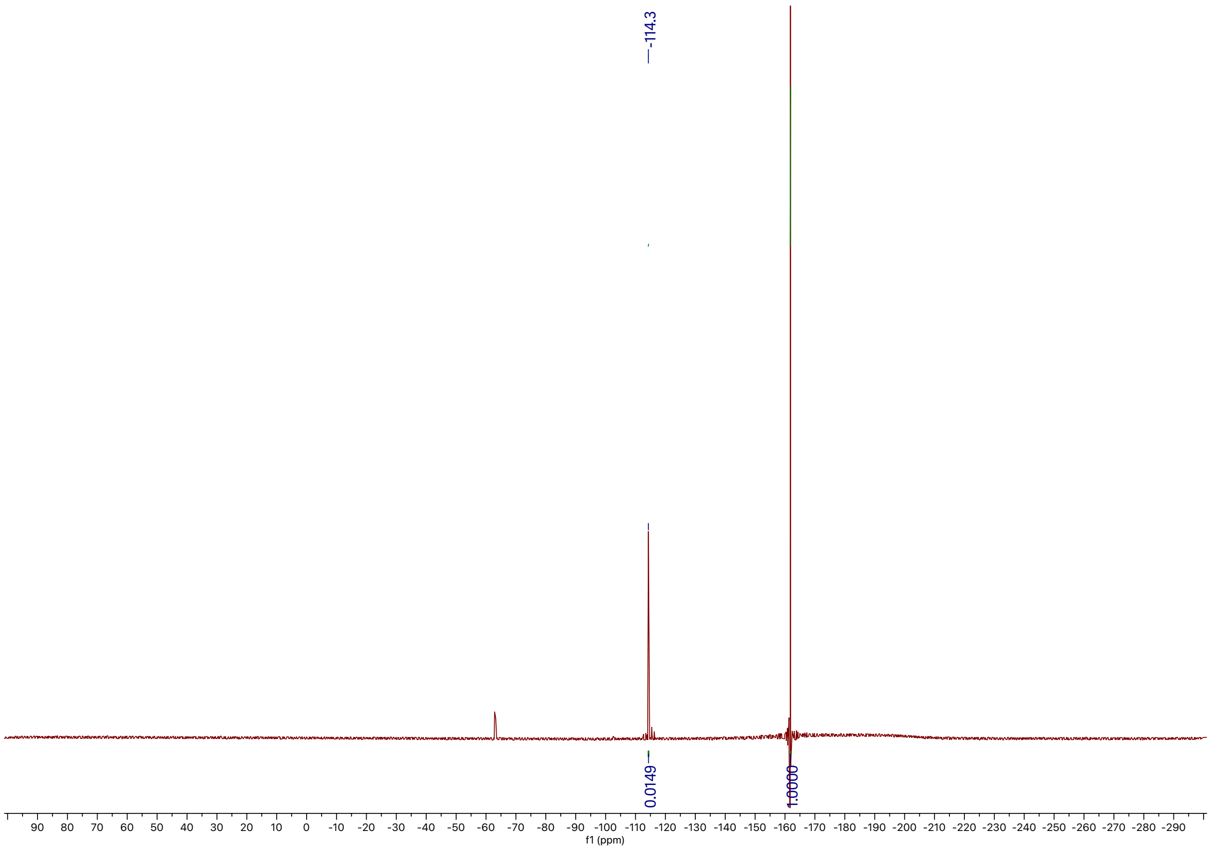
-114.3
0.0149
1.0000
90 80 70 60 50 40 30 20 10 0 -10 -20 -30 -40 -50 -60 -70 -80 -90 -100 -110 -120 -130 -140 -150 -160 -170 -180 -190 -200 -210 -220 -230 -240 -250 -260 -270 -280 -290
f1 (ppm)
